# Enabling cell-type-specific behavioral epigenetics in *Drosophila*: a modified high-yield INTACT method reveals the impact of social environment on the epigenetic landscape in dopaminergic neurons

**DOI:** 10.1186/s12915-019-0646-4

**Published:** 2019-04-10

**Authors:** Pavan Agrawal, Phuong Chung, Ulrike Heberlein, Clement Kent

**Affiliations:** 1 Janelia Research Campus, Howard Hughes Medical Institute, Ashburn, VA USA; 2 Department of Biology, York University, Toronto, Canada

## Abstract

**Background:**

Epigenetic mechanisms play fundamental roles in brain function and behavior and stressors such as social isolation can alter animal behavior via epigenetic mechanisms. However, due to cellular heterogeneity, identifying cell-type-specific epigenetic changes in the brain is challenging. Here, we report the first use of a modified isolation of nuclei tagged in specific cell type (INTACT) method in behavioral epigenetics of *Drosophila melanogaster*, a method we call mini-INTACT.

**Results:**

Using ChIP-seq on mini-INTACT purified dopaminergic nuclei, we identified epigenetic signatures in socially isolated and socially enriched *Drosophila* males. Social experience altered the epigenetic landscape in clusters of genes involved in transcription and neural function. Some of these alterations could be predicted by expression changes of four transcription factors and the prevalence of their binding sites in several clusters. These transcription factors were previously identified as activity-regulated genes, and their knockdown in dopaminergic neurons reduced the effects of social experience on sleep.

**Conclusions:**

Our work enables the use of *Drosophila* as a model for cell-type-specific behavioral epigenetics and establishes that social environment shifts the epigenetic landscape in dopaminergic neurons. Four activity-related transcription factors are required in dopaminergic neurons for the effects of social environment on sleep.

**Electronic supplementary material:**

The online version of this article (10.1186/s12915-019-0646-4) contains supplementary material, which is available to authorized users.

## Introduction

Environmental stressors have robust effects on the behavior of animals including humans, rodents, and fruit flies. Social isolation is considered a form of “passive” stress that can profoundly affect behaviors by inducing anxiety and depression-like symptoms [1–3]. For instance, solitary confinement in humans has been shown to induce depressive symptoms, increased aggression [4], and increased risk for suicide [5, 6]. In addition, social isolation is known to affect sleep quality and duration in humans [7, 8], mice [9, 10], and the fruit fly *Drosophila melanogaster* [11, 12].

Epigenetic mechanisms, including histone post-translational modifications and DNA methylation, are engaged by stressors, such as early life adversity [13, 14], reduced maternal care [15], maternal separation [16, 17], drugs of abuse [18–22], and social defeat [23], and play a key role in influencing gene expression in the brain. Efforts made in the last two decades have implicated a role for epigenetic mechanisms in social isolation in several brain regions in rodents. Social isolation has been shown to cause epigenetic changes in the midbrain of mice [24] and an increase in DNA methylation in dopaminergic neurons [25, 26]. Several studies have implicated dopaminergic neurons in encoding the effects of social isolation in rodents [17, 27, 28], and social isolation has been shown to decrease dopamine levels in flies [11] and mice [25]. Dopaminergic neurons play an important role in modulating behaviors influenced by social isolation in *Drosophila*, including aggression [29], sleep [11, 30–33], and alcohol intoxication [34]. While studies have implicated epigenetic mechanisms in subsets of brain regions and point to a role of dopaminergic neurons in social isolation, it is not known how stressors such as social isolation influence the epigenome in specific cell types of the brain and thereby affect behavior.

The brain is a highly heterogeneous tissue. This poses a challenge for epigenomic studies since ChIP-seq and RNA-seq data obtained from brain tissue are significantly more variable than data obtained from other tissue types or cells in culture [35]. This is especially challenging for small model organisms such as *Drosophila*, where manually dissecting subsets of brain regions for epigenomic analysis is not possible. Consequently, studies of behavioral epigenetics in *Drosophila* have used either mutants or flies in which the GAL4-UAS system [36] was used to modulate levels of epigenetic writers or erasers [37–48]. Studies that looked at global epigenetic changes using ChIP-seq have used either entire fly heads or whole animals after drug treatment or epigenetic mutation [18, 46, 49].

Strategies to isolate specific cell types from brains, such as laser capture microdissection [50] or manual sorting of neurons [51, 52] do not provide enough material for epigenomic analysis. A popular approach for cell-type-specific epigenomic analysis is INTACT (*i*solation of *n*uclei *ta*gged in specific *c*ell *t*ypes) [53]. INTACT allows the isolation of specific cell types using tagged nuclei that are affinity purified from a heterogeneous cell population. Recent advances in INTACT have made it possible to use this method in *Caenorhabditis elegans* [54], *Drosophila* [55], and mouse [56, 57] for epigenomic and proteomic [58] analyses. Despite its versatility, to the best of our knowledge, no studies to date have utilized INTACT for analysis of rare cell types in the field of behavioral epigenetics. INTACT in mouse has been shown to work with 1–3% of total adult neuronal nuclei [56] and epigenetic analysis with ChIP-seq required ~ 0.5–1 million purified neuronal nuclei [57]. INTACT in *Drosophila* either requires thousands of animals to access rare cell types [55] or the use of pan-neuronal or pan-glial drivers to obtain sufficient nuclei for epigenetic analysis [55, 59, 60]. This represents a significant barrier for the field of behavioral epigenetics, in which rare cell types need to be collected in restricted time windows and where collecting tissue from large number of animals would be difficult.

To address these issues, we developed a modification of the INTACT method, mini-INTACT, which uses approximately 100-fold less material. We used mini-INTACT to purify nuclei from dopaminergic neurons, which comprise less than 0.1% of fly brain neurons. We used 200–250 fly heads (10–15,000 nuclei) of socially isolated and socially enriched flies and ascertained epigenetic changes on a genome-wide scale using ChIP-seq. Comparing the enrichment profiles of six different histone modification marks with mRNA expression levels in dopaminergic neurons obtained by RNA-seq revealed clusters of genes that may contribute to the effects of social isolation and social enrichment. Our unsupervised clustering analysis followed by gene ontology (GO) analysis of these groups showed an enrichment of genes encoding readers and writers of the epigenome, cell signaling molecules, and molecules involved in neural and behavioral processes. We found that some genes encoding activity-regulated transcription factors (ARG-TFs) [61] respond to social environment in dopaminergic neurons and that knockdown of the genes encoding four of these ARG-TFs (*cabut*, *Hr38*, *stripe*, *CrebA*) reduced the effects of social experience on daytime sleep. Taken together, these data show that the epigenetic landscape of dopaminergic neurons undergoes modifications with just 4 days of social isolation in adult male flies and that ARG-TFs are part of these changes.

## Results

### mini-INTACT purifies rare cell types from adult *Drosophila* brain

**Fig. 1:**
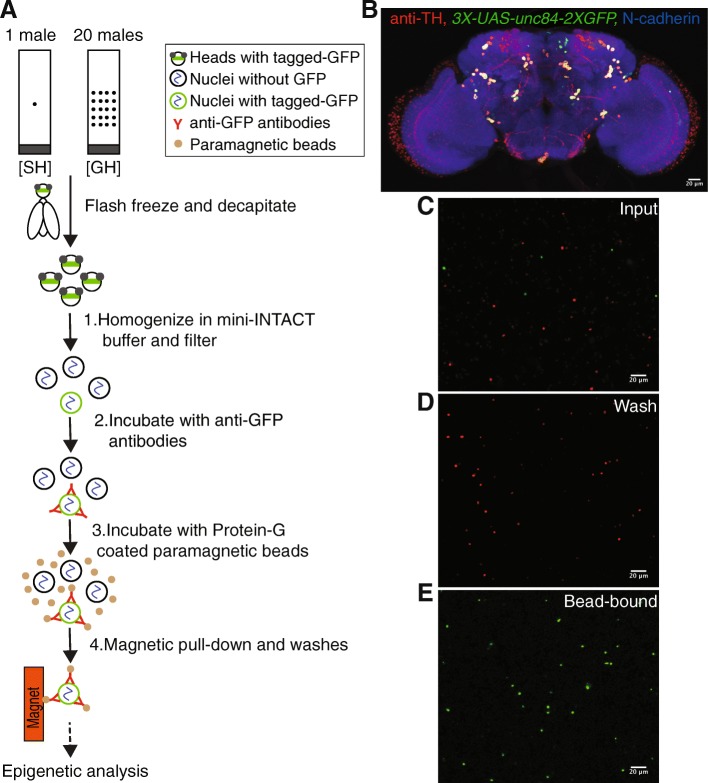
mini-INTACT method affinity purifies cell-type-specific nuclei for epigenetic analysis in *Drosophila.* **a** Schematic of mini-INTACT method to affinity purify dopaminergic neurons from heads of *Drosophila* male flies after 4 days of social isolation or social enrichment. **b** Expression of SUN-tagged GFP (*3XUAS-unc84-2XGFP*) in dopaminergic neurons driven by *TH-GAL4* in an adult male brain. **c** To assess the purity of dopaminergic nuclei, nuclei were obtained from a mixture of heads derived from flies expressing *10XUAS-unc84-tdTomfl* under the control of the pan-neuronal *elav-GAL4* driver (red) and flies expressing *3XUAS-unc84-2XGFP* under the control of the *TH-GAL4* driver (green). **d** After capturing green nuclei using bead-bound anti-GFP antibodies, red nuclei were washed away. **e** Bead-bound green nuclei were almost devoid of contaminating red nuclei

The INTACT method developed in *Drosophila melanogaster* expresses a SUN domain protein (UNC84) from *C. elegans* that localizes green fluorescent protein (GFP) to the inner nuclear membrane (*unc84-2xGFP*) [55]. While the INTACT method works well to isolate specific cell types from *Drosophila*, it requires thousands of fly heads to access rare cell types. This represents a significant challenge for the field of behavioral epigenetics, where animals need to be perturbed and collected in restricted temporal windows and where manually manipulating large number of animals is difficult. To address this issue, we modified the INTACT method [55] to isolate rare cell types (< 0.1% of adult *Drosophila* brain) from 200 to 250 fly heads; we named this modified method mini-INTACT (Fig. 1a and “Materials and methods” section).

Changes made to the INTACT protocol to achieve this ~ 50–100-fold reduction in input material include an improved homogenizer design to prevent sample loss (Additional files 1 and 2); a 20-fold reduction in homogenization and immunoprecipitation volume; the use of a single buffer system for homogenization, immunoprecipitation, and washing; and the sequential addition of anti-GFP antibodies and magnetic beads directly to the homogenate for increased binding efficiency (see the “Materials and methods” section for details).


Additional file 2:Operation of homogenizer used in mini-INTACT. Movie illustrating operation of modified homogenizer used in mini-INTACT. (MOV 52009 kb)


We expressed the INTACT transgene in dopaminergic neurons using the tyrosine hydroxylase driver, *TH-GAL4* [62], which is expressed in ~ 120 neurons in the adult brain [62–64](Fig. 1b). We compared expression of the *TH-GAL4*-driven transgene *UAS-UNC84-2XGFP* in the adult brain after varying the copy number of the upstream activator sequences (UAS) from 3X to 5X and 10X. The *3X-UAS-unc84-2XGFP* transgene most faithfully reproduced *TH*-*GAL4* expression (Fig. 1b); ectopic expression was seen when 5 or 10 copies of UAS-tagged GFP were used (Additional file 3). Therefore, we used *3X-UAS-unc84-2XGFP* for all our experiments. Counting of GFP^+^TH^+^ nuclei and GFP^+^TH^−^ nuclei in the confocal stack for Fig. 1b showed at most ~ 12% of TH^−^ nuclei among the GFP^+^ nuclei. Social isolation reduces daytime sleep when compared to group housing [11, 12]; we therefore tested for the effects of expression of the INTACT transgenes on daytime sleep using the *Drosophila* activity monitor. Expression of *3X-UAS-unc84-2XGFP* in dopaminergic neurons did not affect daytime sleep in either single-housed or group-housed male flies (Additional file 4), leading us to conclude that the expression of the transgenes had no significant effects on fly behavior.

To assess purity of the isolated nuclei, we mixed 200 heads of flies expressing *UAS-UNC84-2XGFP* driven by *TH-GAL4* with 200 heads of flies expressing *UAS-UNC84-tdTomfl* driven by the pan-neuronal driver *elav*-*GAL4*. Processing these heads using mini-INTACT resulted in an expected ratio of ~ 120 GFP-positive green to 10^5^ tdTomfl-positive red nuclei. Very few red nuclei were observed in the purified bead-bound sample as compared to green nuclei (Fig. 1c–e). Therefore, the purity obtained by mini-INTACT (~ 98%, Additional file 5) is comparable to that described for the INTACT method [55] that requires ~ 50–100 times more input material.

By manually counting various purified and diluted samples, we assessed the yield of nuclei to be in the range of 30–50% (data not shown). Therefore, from the heads of 200–250 flies, we estimated a yield of 10,000–15,000 dopaminergic nuclei for each ChIP-seq reaction. Dopaminergic neurons were obtained from *Drosophila* males that were either socially isolated or socially enriched for 4 days, hereafter referred to as single-housed (SH) and group-housed (GH) male flies, respectively. Chromatin was processed from these nuclei for ChIP-seq using six different histone modification marks as described in the “Materials and methods” section and below.

In summary, mini-INTACT allowed us to retrieve sufficient chromatin for ChIP-seq analysis of six histone marks from dopaminergic neurons of 200–250 flies for each housing condition. Each mark had two biological replicates, except for H3K4me3, which had three.

### Epigenomic profiling of dopaminergic neurons from socially isolated and socially enriched male flies

**Fig. 2:**
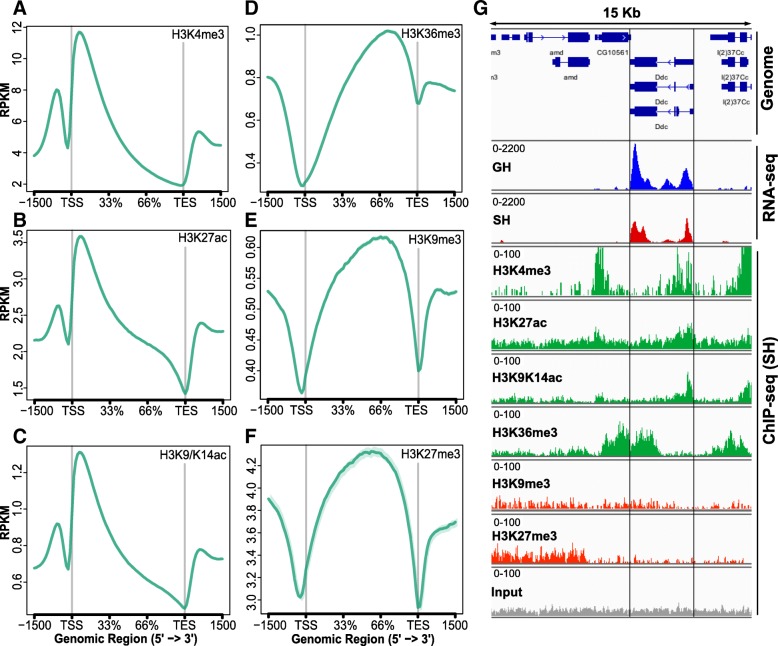
Epigenome of mini-INTACT purified dopaminergic neurons measured by ChIP-seq and RNA-seq. **a**–**f** Genome-wide profiles of the levels for the six epigenetic marks shown as ngs.plot displays. Vertical axes indicate genome-wide average of read counts per million reads. **a**–**c** Activating marks were concentrated in the promoter and immediately downstream of the TSS. **d** H3K36me3, a mark associated with transcriptional elongation, was enriched in the gene body and skewed towards the TES. **e**, **f** Two repressive marks were depleted from the TSS and TES, concentrated in the gene body, and enriched upstream of the promoter region. **g** Epigenetic and transcriptional enrichment profiles surrounding the *Ddc* gene. Representative RNA-seq panels show that *Ddc* is more strongly expressed in GH (blue) than in SH (red) males. The distribution of epigenetic marks shown is representative of the SH dataset. The four activating marks (green panels) were high in this strongly expressed gene, while the two repressive marks (red) showed low levels. An example of single “input” DNA track, which is used as a control for mark levels, is shown in the final panel

The genome-wide profiles of activating and repressive marks [65] with respect to gene bodies are shown in ngs.plot displays [66] averaged over the genome (Fig. 2a–f). As expected from previous studies with human cells [67, 68], flies [46, 69], and mouse brain [70], activating marks H3K4me3, H3K27ac, and H3K9/K14 ac were maximally enriched downstream of the transcription start site (TSS) (Fig. 2a–c), while H3K36me3, which has been associated with transcriptional elongation, is skewed towards transcription end site (TES) with enrichment in the gene body (Fig. 2d). Repressive marks H3K9me3 and H3K27me3 were depleted from TSS and TES and enriched in the central portion of the gene body (Fig. 2e, f).

As an example of transcriptional changes and epigenetic profiles at a specific locus, we depict the highly expressed *Dopa decarboxylase* (*Ddc*) gene, which is involved in dopamine synthesis. *Ddc* mRNA levels were upregulated in GH flies as compared to SH flies (fold change 33% over three replicates, *p* = 4.3 × 10^−9^, Fig. 2g), which is consistent with a previous study showing that the levels of dopamine are lower in the heads of socially isolated flies [11]. The epigenetic profile of this locus recapitulates the global profile, with marks associated with transcriptional activation (H3K4me3 and H3K27ac) centered around the TSS, H3K36me3 skewed towards the TES, and repressive marks H3K9me3 and H3K27me3 not showing enrichment as compared to input DNA. Comparative analysis of epigenetic profiles between GH and SH males using SICER [71] showed that the levels of the activating mark H3K4me3 were significantly higher in GH flies around the *Ddc* gene (normalized read count GH 35.10, SH 30.58, *p* = 0.0002, *p*_adjusted_ = 0.0004) and that the activating mark H3K27ac was similarly increased (GH 1069, SH 651, *p* = 1.5 × 10^−107^, *p*_adjusted_ < 10^−60^) in agreement with the pattern of mRNA expression. Repressive marks, which were already very low on this gene, showed no significant differences.

ChIP-seq replicates for histone modification marks were highly correlated (median Pearson’s *r* of log-transformed coverage among all pairs of biological replicates, *r* > 0.99 (Additional file 6). The genome-wide correlation between levels of activating and repressive marks with each other and with mRNA levels is shown in Table 1. All activating mark levels correlate positively with each other and with mRNA levels, while repressive marks correlate positively with each other and negatively with mRNA levels, as expected. H3K9me2 and H3K9me3 modifications are associated with heterochromatin protein 1 (HP1)-mediated heterochromatin formation and transcriptional repression [65]; however, these modifications are not strongly correlated with transcriptional repression in either human cells [68] or *Drosophila* [46]. Consistent with these findings, we find correlations of H3K9me3 to be weaker with mRNA levels and with activating marks when compared with the repressive mark H3K27me3.

**Table 1:**
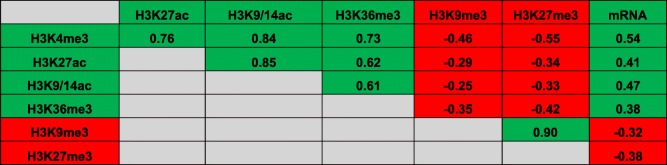
Pearson correlation coefficient values of pairwise comparisons among ChIP-seq for six histone modification marks and gene expression. Activating epigenetic marks and positive correlations are shown in green; repressive marks and negative correlation are shown in red

Analysis of ChIP-seq data using SICER [71] returned thousands of “islands” in which epigenetic mark levels were significantly different between GH and SH males (FDR < 0.001) (Additional files 7 and 8). An island as defined by SICER or DiffReps is a continuous region in which marks change in a uniform direction with significant differences in GH and SH. Typically, an island does not cover the entirety of a gene, so interpretation of SICER islands requires care. For example, an H3K4me3 island with a fold change of 1.25 was found within the body of the *foraging* gene, from position 3,622,074 to 3,656,953 bp on chromosome 2 L. This island covers the first exon of seven *foraging* transcripts, but not of the remaining six transcripts annotated in Flybase [72](www.flybase.org). By contrast, an island in *Snmp2* covers half of the first exon of all three transcripts and has H3K4me3 fold change of 1.70. Details of SICER-detected islands are in Additional file 7. We used DiffReps (Additional file 8) to characterize the portions of genes that had significant mark changes. This showed that H3K4me3 had 1143 islands with significant GH vs SH changes in gene introns, 832 in first exons, 750 in 5′UTRs, and 531 in last exons (531). By contrast, repressive mark H3K27me3 had more significant changes in last exons (398) than first exons (292), with introns (352) and 5′UTR (130). This pattern follows the overall mark levels shown in Fig. 2a–f.

In summary, when averaged over entire gene bodies, there are small but statistically significant changes in histone marks, but when examined in islands detected by SICER and DiffReps, there are much larger changes, often restricted to regions such as introns or the first exon of a gene (for activating mark H3K4me3).

### Social experience induces transcriptional changes in dopaminergic neurons

Since most of the transcripts are exported from the nucleus soon after transcription [73], nuclear RNA alone may not represent fully the transcriptional changes of the cell due to social experience. A recent study showed that considerable differences exist in the profiles of nuclear and cytosolic transcripts of individual cells [74]. Therefore, to profile the sum of nuclear and cytosolic mRNAs, we isolated dopaminergic neurons from GH and SH males using fluorescence activated cell sorting (FACS) and performed RNA-seq (see the “Materials and methods” section). Replicate concordance was assessed using Pearson’s *r* of log-transformed counts among all pairs of biological replicates (*n* = 3, *r* = 0.95 for GH and *r* = 0.91 for SH flies). These correlations are similar to those reported before for RNA-seq from dopaminergic neurons [61, 75].

We used three methods (EdgeR, CyberT, and FCros) to identify genes that are differentially expressed in dopaminergic neurons of GH and SH flies [76–78]. EdgeR and CyberT use generalizations of the between-treatment *t* test method, while FCros uses a nonparametric method based on fold changes, which is more robust to variation in mRNA counts. Using EdgeR with a FDR of 5% (see the “Materials and methods” section), we identified 16 genes upregulated in SH and 9 genes upregulated in GH males (Additional file 9). The fold change-based technique FCros identified 451 genes upregulated in SH and 466 upregulated in GH, after FDR correction of 5%. CyberT produced intermediate results. In figures and tables, we quote the FDR 0.05 obtained with FCros values, except where noted otherwise. Additional file 9 shows each gene reported as differentially expressed (DE) by any of the three methods. The methods overlapped substantially: the overlap of DE genes of any pair of methods was 59%, 63%, and 86%.

Gene ontology analysis of all differentially expressed genes (upregulated in either GH or SH males) using the DAVID GO tool [79] found two related GO groups: epigenetic (unadjusted *p* = 0.0098) and negative regulation of gene expression (*p* = 0.016). GOrilla GO analysis [80] found GO group: peptide *n*-acetyltransferase activity (*p* = 0.00044), the latter group containing genes belonging to several histone acetyltransferase complexes genes including Tip60 complex members Enhancer of Polycomb and *dom*, SAGA complex members *Taf10b* and *Taf12*, TAC1 complex members *nejire* and *Sbf*, and Enok complex members *enok*, *Gas41*, and *Ing5*. The full GOrilla and DAVID analyses are shown in Additional file 10. DAVID GO analysis of genes upregulated in SH flies revealed statistically significant clusters of ribosomal and mitochondrial genes, but DAVID analysis of genes upregulated in GH flies found no significant clusters.

Daytime activity is significantly higher in SH flies as compared to GH flies [11], suggesting that metabolic activity might be higher in SH flies. It is also known that mitochondrially encoded genes are upregulated in waking flies [81]. Consistent with these observations, in our RNA-seq dataset, we found that of 15 known mitochondrially encoded genes, 14 were higher in SH than in GH flies (*p* = 0.0005, binomial test).

In summary, transcript levels of many genes expressed in dopaminergic neurons were changed by social housing conditions, including those of many epigenetic reader and writer genes.

### Social experience alters epigenetic landscape

**Fig. 3:**
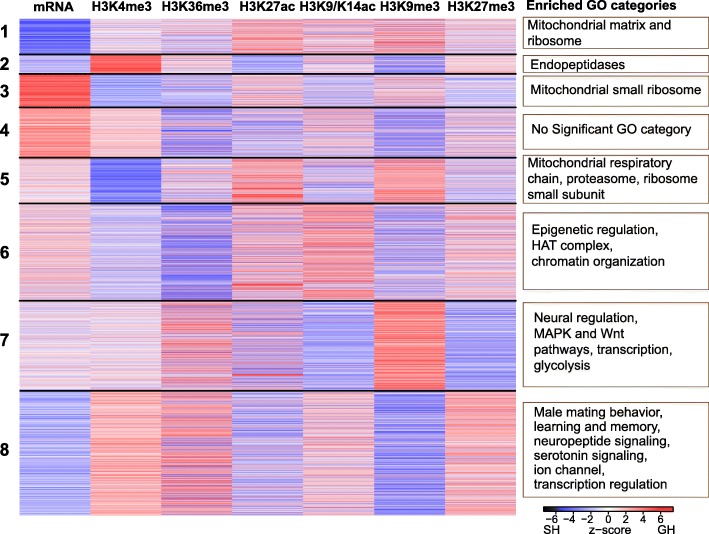
Epigenetic landscape of genes expressed in dopaminergic neurons is modulated by social experience. Heat map of eight groups identified by *k*-means clustering of the change in whole gene average mark levels and mRNA levels between GH and SH males. Red lines show genes whose marks or mRNA was higher in GH than SH males, blue lines show those that were higher in SH than GH males. Some clusters are enriched for genes with neural and regulatory functions, especially clusters 6–8. Enriched GO categories from each cluster are shown on the right. N (genes per cluster): 1:979, 2:612, 3:602, 4:413, 5:900, 6:637, 7:863, 8:544

To understand how social experience affects the epigenetic landscape of dopaminergic neurons, we focused on epigenetic changes observed in the top 40% of genes by mRNA expression levels (“expressed genes”—see the “Materials and methods” section). We clustered the *z*-score normalized differences between GH and SH flies for all six epigenetic marks and for mRNA and performed *k*-means clustering as in [115] (see the “Materials and methods” section and Additional file 11). Eight clusters provided optimal separation of genes (elbow test). These clusters, arranged in increasing order of mean mRNA expression levels, are shown as a heat map of mRNA and epigenetic mark *z*-score values in Fig. 3, with red showing marks/mRNAs that are higher in GH flies and blue showing those that are higher in SH flies.

The first five clusters are enriched for housekeeping functions and include mitochondrial, ribosomal, and proteasome genes. However, the last three clusters (6–8, containing genes with higher expression) are enriched in neural and regulatory functions.

Cluster 6 is enriched for genes with epigenetic functions, including histone acetyltransferase (HAT) genes. As noted above, HAT genes and several epigenetic regulators encode differentially expressed mRNAs; but as can be seen in the left-hand column (mRNA *z*-score), mRNA level changes are heterogeneous, as some genes in this cluster are upregulated in GH males (red) and others in SH males (blue). This is interesting considering that the *k*-means analysis grouped genes in this cluster not primarily by the direction of their mRNA change with regard to housing condition, but by their epigenetic mark changes; this cluster is enriched for readers and writers of epigenetic marks.

The seventh cluster is enriched for genes regulating neural function (some of which are members of the MAPK or WNT signaling pathways), transcription factors, and glycolysis genes. In this cluster, there is a pair of marks that show strong, anti-correlated changes: heterochromatin protein 1 (HP1)-associated H3K9me3 (higher in GH than SH) and the Polycomb repressive complex 2 (PRC2)-associated H3K27me3 mark (higher in SH than GH).

The two inhibitory marks also change in opposite directions in the final (highest expression) cluster 8, but in this cluster, the directions of change are reversed. H3K9me3 in cluster 8 is higher in SH than GH males and H3K27me3 is higher in GH than SH males. This cluster is enriched in neural function genes, including those involved in male mating behavior, learning and memory, synaptic, neuropeptide and serotonin signaling, as well as ion channels and transcription regulation genes. Genes of this cluster have on average higher expression in SH flies than in GH (*p* < 10^−15^, *t* = − 15.06, df = 1361).

In summary, there are clusters of genes whose epigenetic marks and mRNA levels respond to social experience in similar ways within each cluster, but quite differently between clusters. This suggests that different regulatory programs may be acting in each cluster. We use this putative division of genes into epigenetically distinct clusters to try to determine what the regulatory program might be in the next section.

### Social enrichment induces activity-regulated genes in dopaminergic neurons

We used the CentriMo tool [82] to search for transcription factors (TFs) whose binding sites might be enriched (occur more often than chance) in promoter-proximal regions of genes expressed in dopaminergic neurons. The eight epigenetic clusters discussed in the previous section provided us with groups of genes that had similar regulatory programs (as evidenced by their epigenetic and transcriptional response to housing conditions). We used CentriMo to search for TFs whose binding sites were enriched in genes of each cluster relative to a control group of the same number of genes randomly chosen from other clusters. The promoter-proximal region (± 500 bp from TSS) was scanned. We found a group of 24 TF motifs that were enriched in one or more of the clusters’ promoter regions. These correspond to 14 TF genes, as in many cases multiple binding motifs are documented for one TF in the motif databases used by CentriMo (Additional file 12).

We further filtered the TFs under investigation by two criteria: (1) the TF had to be in the expressed gene set and (2) the TF had to show at least a 33.3% change in transcript levels in response to housing conditions. Five TFs met our criteria: Hr38 (Hormone receptor-like in 38), Sr (Stripe), CrebA, Cbt (Cabut), and Pho (Pleiohomeotic). Interestingly, the genes encoding four of these TFs (*Hr38*, *sr*, *CrebA*, and *cbt*) are orthologs of vertebrate immediate early genes [83]. The expression of these genes was higher in GH males than in SH males. We hypothesized, consistent with another study [11], that being in the GH environment constitutes an enrichment of stimuli for male flies. In a recent study [61], dopaminergic neurons were thermogenetically stimulated by expressing dTRPA1 using the *TH-GAL4* driver and the changes in mRNA levels were measured after 60 min. Genes with large transcriptional upregulation due to neuronal stimulation were called “activity-related genes” (ARGs). We compared the change in expression levels (log fold change) of the top 50 upregulated ARGs in dopaminergic neurons found by Chen et al. [61] with the change of gene expression for the same genes in dopaminergic neurons between GH and SH males in our dataset; we found a significant positive correlation (*r* = 0.41, *p* = 0.003). That is, the size of the transcriptional response of ARGs (as defined by Chen et al. [61]) to neuronal stimulation correlates with the size of the transcriptional response to group housing in DA neurons in our experiments. Interestingly, changes in the levels of some histone marks observed between GH and SH males for these ARG genes also correlated significantly with Chen et al.’s [61] changes in expression of ARGs upon neuronal stimulation: H3K9me3 (*r* = − 0.35, *p* = 0.01), H3K27me3 (*r* = 0.46, *p* = 0.0009), and H3K4me3 (*r* = 0.32, *p* = 0.026). This result suggests that genes in dopaminergic neurons responding to short-term direct neural stimulation also respond epigenetically and transcriptionally to the long-term presumed behavioral stimulation of dopaminergic neuron due to interaction among GH flies over the course of 4 days.

**Fig. 4:**
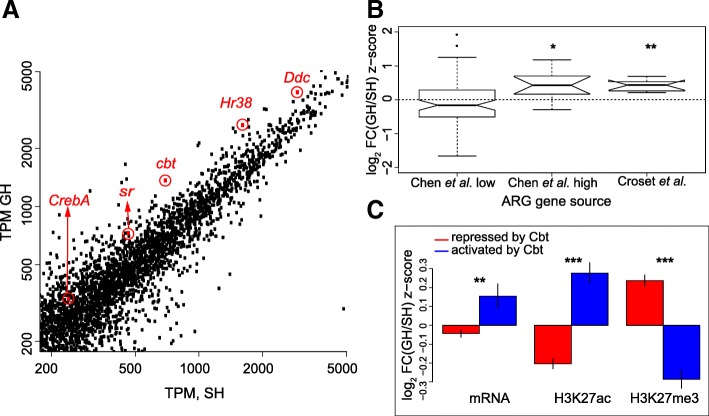
Activity-regulated genes (ARGs) are upregulated in dopaminergic neurons of GH males and correlate with transcriptional repression. **a** A zoomed in scatter plot of GH versus SH mRNA values. *Ddc* (Fig. 2) and four ARG-TFs defined as such in [61] and showing high fold change in our data are highlighted. **b** Box plots of mRNA log fold change z-scores (GH is positive; SH is negative) for groups of ARGs from two different studies. Genes with log fold change lower than 1.5 in Chen et al.’s study [61] are not over-represented in GH flies (Chen et al. low). However, the last two groups are significantly over-represented in GH flies. **c** Genes repressed (red) or activated (blue) by the ARG-TF Cbt (from Bartok et al., 2015) are shown on the same *z*-score scale as in (**b**). Genes repressed by Cbt have significantly lower mRNA and activating mark H3K27ac and significantly higher repressive mark H3K27me3. Genes activated by Cbt show the reverse pattern

Four transcription factors were among the genes that showed the largest upregulation in response to direct neuronal stimulation: *Hr38*, *sr*, *CrebA*, and *Cbt* [61]. The mRNA for all four of these TFs was also upregulated in our RNA-seq data in GH as compared to SH males (Fig. 4a), suggesting that they might regulate transcriptional responses of other genes in response to group housing. Interestingly, a recent study of gene expression in the *Drosophila* midbrain found that transcription of these ARGs was correlated across many types of neurons [84] under normal conditions—that is, there appears to be a common regulatory program across neural cell types for these genes. The epigenetic effects of social housing on marks in ARG genes were more highly correlated (by 2.4 times) among these ARGs than among all genes (*t* = 2.336, df = 20, *p* = 0.03). Of the 10 ARG genes found by Croset et al. [84], 9 were also present in our top 40% expressed genes in dopaminergic neurons (Fig. 4b). These 9 ARGs had GH/SH fold changes ranging from 1.44 to 2.11 (mean 1.70; *p* = 0.004, binomial test; Additional file 13). Similarly, genes with log fold change above 1.5 in Fig. 4 of Chen et al. [61] had high log fold changes in our data (Fig. 4b, Chen et al. high), while lower fold change genes from the same Chen et al. [61] dataset had fold changes in our data not different from zero (Fig. 4b, Chen et al. low) showing that fold change sizes in this set of genes seem to be conserved across experimenters and conditions.

In summary, several ARGs expressed in dopaminergic neurons respond similarly to 4 days of social housing and to 60 min of thermogenetic stimulation. We report below the effects of these ARG transcription factors on downstream targets using both bioinformatic analyses and by manipulating levels of these ARG TFs in dopaminergic neurons and measuring the effect on sleep.

### ARGs predict transcriptional changes due to social experience

It has been suggested that ARG expression in neurons “might be part of a homeostatic neuronal response to reduce excitability” [84]. We do not have direct measures of excitability, so we narrowed our attention and determined the effect of one of the activity-related transcription factors with the highest fold change between GH and SH, Cbt, on downstream targets. To test if the factor encoded by the ARG *cbt* is acting as a transcriptional repressor or activator in GH conditions (where its mRNA expression is increased), we compared a published dataset for *cbt* [85] with our data. In the Bartok et al. study [85], genome-wide transcriptional responses were measured upon overexpression and knockdown of *cbt* in adult male fly heads. We used mRNA expression from this study to define two sets of genes: “repressed by Cbt” and “activated by Cbt.” The repressed by Cbt set contains genes whose expression is increased upon *cbt* knockdown and decreased upon *cbt* overexpression (Additional file 14). Conversely, the activated by Cbt set contains genes whose expression is decreased upon *cbt* knockdown and increased upon *cbt* overexpression (Additional file 15, first sheet). *cbt* is upregulated by 94% in dopaminergic neurons of GH males compared to SH males in our dataset. Hence, if Cbt indeed acts as a transcriptional regulator in dopaminergic neurons, in GH males, we should see downregulation of genes repressed by Cbt and upregulation of genes activated by Cbt, when compared to SH males. To test this, we compared gene expression between the two gene sets from Bartok et al. [85] using the top 40% of expressed genes in dopaminergic neurons (Fig. 4c, first two bars, and Additional file 15, second sheet). Consistent with our hypothesis, we found reduced expression of genes repressed by Cbt in GH males compared to SH males (*p* = 0.024), and genes activated by Cbt were upregulated in GH males (*p* = 0.017, Additional file 15, second sheet). Thus, Cbt appears to act as a transcriptional regulator in dopaminergic neurons in response to social stimulation in a direction that is parallel to that observed in whole heads [85]. We note that there were more genes expressed in dopaminergic neurons in the repressed set than in the activated set (1293 vs. 376).

We next analyzed the effects of housing on the six histone marks in the two sets of Cbt-regulated genes. For each mark, the difference between the two gene sets was significant at *p* values ranging from 10^−12^ to 10^−32^ (Additional file 15, second sheet). The activating marks H3K4me3, H3K36me3, and H3K9-14 ac and the repressive mark H3K27me3 were higher in GH males in repressed by Cbt genes than in activated by Cbt genes. By contrast, the marks H3K27ac and H3K9me3 were higher in SH males in the repressed by Cbt genes than in the activated by Cbt genes. Interestingly, genes in the repressed by Cbt group were over-represented in our eighth *k*-means cluster (Fig. 3) containing genes involved in neuronal function (odds ratio 1.7:1, chi-squared 95.9, df = 1, *p* = 1.8 × 10^−22^). We present a hypothesis for this unusual pattern of mark changes (mentioned above in our discussion of cluster 8) in the “Discussion” section, but briefly, it involves possible regulation of Polycomb Repressive Complex 2 (PRC2) epigenetic marks. H3K27me3 is the classic PRC2 mark, and it replaces the H3K27ac mark; we highlight these opposing trends for these two marks in Fig. 4c.

If the four ARG-TFs (*Hr38*, *cbt*, *CrebA*, and *sr*) are acting as transcriptional regulators as shown above for *cbt* in dopaminergic neurons, there should be sets of target genes that are differentially regulated in GH versus SH flies. Experimentally determined lists of genes regulated by *Hr38*, *CrebA*, and *sr* are not available; we therefore used as a surrogate the number of TF binding sites for the ARGs near the TSS of each gene as an independent variable. We performed multi-linear regressions (see the “Materials and methods” section and Additional file 16) with expression level change (mRNA log fold change) between GH and SH males as the dependent variable and the number of TF binding motifs per gene in a 1000-bp region centered on the TSS as the independent variable. The motifs used were for the four TFs in the ARG group and for TF encoded by *pho* (associated with PRC2-mediated epigenetic regulation) [86, 87], whose binding motifs were enriched in genes of the eight clusters described above (Fig. 3) (see the “Materials and methods” section and Additional file 12).

We did the above multilinear regressions for several functional sets of genes that were (a) enriched in the three clusters containing genes expressed at medium or high levels (clusters 6, 7, and 8, Fig. 3), (b) involved in epigenetic regulation or neural function, and (c) relevant to male fly behavior. Since GO analysis is ineffective in functionally classifying small sets of genes, we manually categorized the genes in each group using their functions defined in Flybase. Genes in the following nine functional groups were identified: sleep, neuropeptide, male mating, G-protein signaling, ligand-gated ion channel, catecholamine metabolism, MAPK signaling, and certain epigenetic genes (Table 2). The writers and erasers of marks were grouped by whether their marks tend to activate or repress gene transcription.

**Table 2:** mRNA changes between GH and SH males are predicted by changes in epigenetic marks and presence of some TF binding sites. The ability to predict is given by the coefficient of multiple correlation *r*, and the *p* value from an *F* test. Full statistics are given in Additional file 16. The sign of the partial correlation coefficient is given as +, −, or blank for non-significant values. (−) indicates the coefficient was marginally significant

	Sleep-related genes	Neuropeptides and receptor genes	Male mating genes	G-protein signaling genes	Ligand-gated ion channel genes	Catecholamine metabolism genes	MAPK signaling genes	Epigenetic activation genes	Epigenetic repression genes
Group	1	2	3	4	5	6	7	8	9
*r*	0.31	0.25	0.53	0.34	0.39	0.72	0.30	0.79	0.86
*p*	0.01	0.05	0.001	0.001	0.03	0.03	.005	0.007	0.006
Hr38		–	–	(−)	–		–	–	
Cbt	–		–						+
CrebA				–					
Sr	+					–			
Pho							+	+	+

Interestingly, significant amounts of mRNA change between GH and SH flies were explained by the number of TF binding sites in these functional groups, as shown in Table 2. The table shows the *r* and *p* values for the regressions and which TF motifs were significantly different. *Hr38*, *cbt*, *CrebA*, and *sr* putative binding sites each show a significant connection to mRNA change in one or more of the six functional gene groups. Of note, in eight out of nine functional groups where ARG-TFs motifs were significantly different, the direction of the effect of *Hr38*, *cbt*, and *CrebA* binding sites was negative—that is, the more potential binding sites the TF had in a gene, the more negative the log fold change in mRNA between GH and SH flies was—that is, such genes tended to have higher expression in SH. This is consistent with the putative role of some ARGs as transcriptional repressors for certain genes in dopaminergic neurons. By contrast, *pho*, known primarily as a transcriptional repressor [88], showed a consistent positive effect on fold change between GH and SH flies.

In summary, changes in the numbers of a few putative transcription factor binding sites were sufficient to predict mRNA changes due to housing in ten functionally relevant gene groups with *r* values ranging from 0.25 to 0.86 (Table 2). This analysis is correlative, but the possible influence of ARGs on differential transcription in GH and SH males in biologically relevant gene groups led us to hypothesize that ARGs might also affect phenotypes known to vary with housing conditions.

### Regulation of social isolation-induced behavior by ARGs

**Fig. 5:**
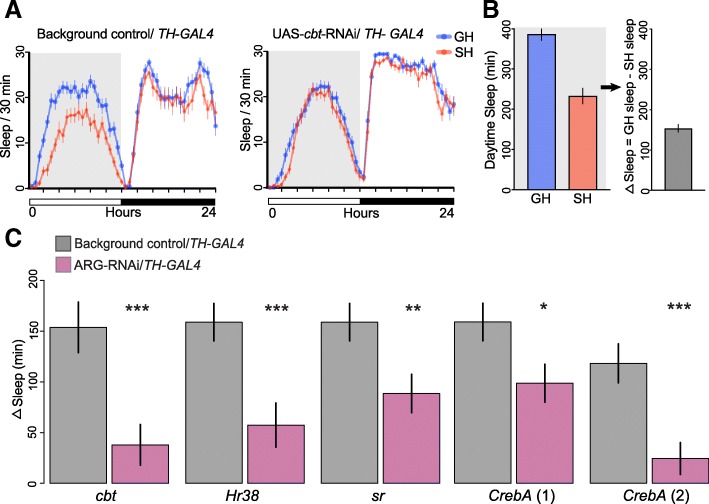
Knockdown of ARGs by RNAi affected social effects on daytime sleep. Knock-down of ARGs in dopaminergic neurons was achieved by driving RNAi transgenes with *TH-GAL4*; controls carried empty vectors without RNAi hairpin and *TH-GAL4*. **a** Example graph of sleep per 30 min over 24 h. Control single housed (SH) flies sleep less than group housed (GH) flies during the day (shaded gray area). Expressing RNAi for ARG-TF *cabut* in dopaminergic neurons significantly reduced this difference. **b** Daytime sleep was measured, and ΔSleep was compared between experimental males carrying the RNAi transgene and controls. ΔSleep is defined as minutes of daytime sleep for GH flies minus the same measure for SH flies (as described by Ganguly et al. [11]). **c** ΔSleep for controls and RNAi knockdowns. Error bars are mean ± SEM. In every case, RNAi knockdown significantly reduced the social effect on ΔSleep. Two different RNAi lines were tested for CrebA, each showing significant reductions

Social isolation has a robust influence on behavior; for example, SH flies show reduced daytime sleep when compared to GH flies [11, 12]. Having shown a potential involvement of ARG-TFs in regulating some genes differentially expressed in dopaminergic neurons of GH and SH males, we knocked down expression of these ARG-TFs in *TH-GAL4* neurons and assayed the males for their sleep patterns. Specifically, we quantified the differences in sleep between GH and SH males (or ΔSleep as described by Ganguly et al. 2006 [11]) in which these ARGs and epigenetic modifiers were downregulated in dopaminergic neurons using RNA interference. Knockdown of all four ARG-TFs (*CrebA*, *Hr38*, *cbt*, and *sr*) significantly reduced ΔSleep (Fig. 5a–c, Additional files 17 and 18). These data show that these ARG-TFs play significant roles in regulating social effects on sleep in dopaminergic neurons.

Our bioinformatic analysis suggested that these ARG-TFs act as transcriptional regulators on downstream targets. Further analysis suggested that genes repressed by Cbt [85] have reductions in H3K27ac and increases in H3K27me3 marks (Fig. 4c). *Brms1* is a member of the histone deacetylase Sin3A repressor complex that contributes to PRC2 activity by deacetylating H3K27, thus allowing H3K27me3 to increase [89], and its transcription was also upregulated in GH flies. *Brms1* knockdown also significantly reduced ΔSleep (Additional file 19), which is consistent with the effects of Cbt on ΔSleep (Fig. 5c).

## Discussion

Results from this study provide insights into how social experiences, such as social isolation and social enrichment, can affect the epigenome of a small, well-defined neural population in the adult *Drosophila* brain. Size and composition of the social group were previously shown to affect gene expression in non-neural tissues and in the brain, and signaling via cuticular hydrocarbons [11, 123–125]. We miniaturized the INTACT method, mini-INTACT, and examined epigenetic changes in a rare cell type isolated from 200 to 250 adult fly heads. We carried out ChIP-seq on mini-INTACT purified dopaminergic neuronal nuclei for six different histone modification marks and correlated it to transcriptional profiles determined by RNA-seq. We found changes in the epigenetic landscape of dopaminergic neurons upon social experience in several gene clusters. Our analysis identified four ARG-TFs [61] responding to social enrichment in dopaminergic neurons. RNAi-mediated knockdown of all four of these ARG-TFs (*cabut*, *Hr38*, *stripe*, *CrebA*) as well as an epigenetic eraser *Brms1* reduced the effects of social experience on daytime sleep (Fig. 5, Additional files 17, 18, and 19).

*K*-means clustering identified several differences in the epigenetic and transcriptional landscape that correlate with social experience. Curiously, many of the genes with the highest mean mRNA expression levels also have higher levels of the repressive H3K27me3 mark and lower levels of some activating marks (Fig. 3, cluster 8). In clusters of genes with lower expression levels, a more classical pattern of high levels of activating marks and low levels of repressive marks was found. But as the expression levels increase from these “classical” gene clusters towards the higher expression clusters (Fig. 3, cluster 8), some activating marks drop and some repressive marks rise. In fully repressed genes, repressive PRC2-related H3K27me3 levels are uniformly higher than repressive PRC1-related H3K9me3 levels. However, in the transition from the classical pattern of marks to the higher expression paradoxical pattern, H3K9me3 increases before H3K27me3. These non-classical relationships between transcription and repressive marks deserve further study. Our simplest hypothesis is that, since we sampled a population of functionally different dopaminergic neurons, there is some heterogeneity in response to social housing among these. However, since there was a small amount (12%) of off-target GFP expression in some nuclei, this may also account for some heterogeneity. This hypothesis may be tested in the future by single-neuron analyses. Alternatively, Ganguly et al. [11] showed that increased daytime sleep in GH males was associated with higher brain dopamine levels and that it could be blocked by ablation of dopaminergic neurons or loss-of-function alleles of many learning and memory genes [11]. Dopaminergic neurons in the fly brain are essential parts of circuits involved in learning and memory [90–92]. Our finding that some genes highly expressed in dopaminergic neurons are associated with an unusual pattern of epigenetic marks (Fig. 3, clusters 7 and 8) is consistent with the finding that mouse differentiated dopaminergic neurons still contain substantial numbers of genes labeled PRCa or Polycomb Repressive Complex Active, with both active transcription but also presence of repressive H3K27me3 marks [93]. Another recent study found that in embryonic stem cells such PRCa genes have a higher variability of gene expression [94]. Taken together, we suggest that some of the genes in fly dopaminergic neurons that show a change in expression between SH and GH males may be similar to those called PRCa genes in mouse dopaminergic neurons.

In the search for insect equivalents of immediate early genes, a study identified ARGs in dopaminergic neurons [61]. The fold change in response to stimulation in their top 50 ARGs correlates significantly with the fold changes we measured in mRNA in response to putative stimulation provided by group housing. The top 50 ARG fold changes in Chen et al.’s study [61] also correlate significantly with GH versus SH fold changes in H3K4me3, H3K9me3, and H3K27me3 in dopaminergic neurons in our study. Genes encoding four transcription factors (*CrebA*, *Hr38*, *sr*, and *cbt)* are in the top genes by fold change in both the Chen et al.’s study [61] and in our own data. RNAi knockdown of each of these ARG-TFs in dopaminergic neurons changed social effects on sleep behavior. However, our knockdown was chronic and developmental effects can thus not be excluded. Future studies with conditional knockdowns in single DA neurons are needed to clarify how these ARG-TFs mediate the sleep response to social housing.

*Hr38* and *stripe* have recently been shown to be activity-regulated in the honey bee and to affect dopamine pathway genes [95]. *Hr38* is a homolog of vertebrate immediate early genes NR4A1–3, and has been shown to regulate dopaminergic neuron transcription and development [96–98]. In flies, *Hr38* overexpression increases dopamine decarboxylase (*Ddc*) transcription in the larval brain [99]. In our data, *Hr38* and *Ddc* are significantly higher in GH than in SH flies (Figs. 2f and 4a). Thus, although ARGs are co-expressed in a much broader range of neural types than just dopaminergic neurons [84] in the adult fly brain, they may have specific effects in dopaminergic neurons. Further work is required to determine whether *Hr38* and *stripe* directly mediate the increased dopamine levels found by Ganguly et al. [11] in GH flies through effects on dopamine metabolism genes.

Cbt is a transcriptional repressor of some genes, for instance in adult male fly heads [85]. Its vertebrate ortholog KLF10/TIEG1 acts with epigenetic repressors such as the H3K4 demethylase JARID1/KDM5B [100] and H3K27 deacetylase BRMS1 in the Sin3A complex [101–103]. Notably, if H3K27ac is deacetylated by BRMS1, this allows for the creation of the PRC2 mark H3K27me3 [89]. We identified genes that were repressed by Cbt and were in our top 40% expression range. These genes had significant differences in social-housing effects on mRNA and epigenetic mark levels, including downregulation in GH mRNA and H3K27ac and upregulation in GH H3K27me3 marks (Fig. 4c). This is correlational evidence for a wide transcriptomic effect of Cbt in GH versus SH flies, but further study is required to delineate which genes downstream of Cbt are important to the social response.

One possible gene which may function with Cbt is *Brms1.* RNAi knockdown of *Brms1* reduced the social housing effect on daytime sleep in a similar manner as *cbt* knockdown (Additional file 19). Thus, we have a consistent picture in which genes repressed by Cbt [85] have reductions in H3K27ac and increases in H3K27me3 marks (Fig. 4c), and knockdown of the deacetylase for H3K27 produces effects on sleep similar to *cbt* (Additional file 19). Further studies are needed to elucidate possible epigenetic pathways mediated by *CrebA, Hr38,* and *stripe.* Croset et al. [84] suggest that the highly inter-correlated set of ARGs they found may have some repressive effects on transcription in various brain regions, such as the mushroom body γ lobes [84]. We found in our data that upregulation of some genes (especially *cbt* and *Hr38*) in GH males is associated with downregulation of genes in some functional gene groups. These groups fall largely into the *k*-means cluster 8 that has increased in PRC2-related marks (H3K27me3) in *TH-GAL4*-expressing dopaminergic neurons. We propose a hypothesis where group housing stimulates ARG expression, and these TFs in turn downregulate some neural function genes in part by increasing PRC2 repressive marks. Further work is required to confirm or invalidate this hypothesis.

## Conclusions

*Drosophila* has been a successful model for neurogenetics due to ease of manipulating flies, availability of a large collection of genetic tools, and the recent development of automated behavioral assays. Adaptation of cell-type-specific epigenetic methods such as mini-INTACT can help leverage this potential to comprehensively study epigenetic changes in specific neurons across several paradigms including stress, drugs of abuse, and neuro-degenerative disorders. Dopaminergic neurons modulate many behaviors, and here, we have shown that social housing changes the epigenetic and transcription landscape of these neurons in ways that may be mediated by four activity-related genes that are transcription factors.

## Materials and methods

### Fly stocks and rearing

*Drosophila melanogaster* in a Canton-S background was reared on standard fly food at 25 °C at 65% relative humidity with a 12/12-h light/dark cycle. For social isolation and group housing experiments, 24–48-h-old males of a given genotype were housed individually or in groups of 20 in standard *Drosophila* vials (2.6 cm diameter × 9.3 cm high) for 4 days containing standard fly food. *3X*-, *5X*-, and *10X-UAS-unc84-2XGFP* and *10XUAS_unc84-tdTomfl* are as described [55] and were a kind gift of Henry Gilbert (Janelia Research Campus, VA, USA), *TH-GAL4* is as described [62]. Tissue collections for genomic analysis were performed near morning activity peak, usually around ZT3-ZT5. The following TRiP RNAi lines [104] were obtained from the Bloomington Stock Center for behavioral analysis: BL36303 (*y[1] v[1]; P{y[+t7.7] = CaryP}attP2*) no insert background control vs. RNAi lines: BL29377 (*Hr38*); BL31900 (*CrebA*); BL27701 (*Sr*). BL36304 (*y[1] v[1]; P{y[+t7.7] = CaryP}attP40*) no insert background control vs. RNAi lines: BL42562 (*CrebA*); BL38276 (*cbt*) and BL42533 (*Brms1*).

### Immunostaining and imaging

Fly brains were dissected in cold 1X phosphate buffered saline (PBS) and fixed in 2% paraformaldehyde made in 1X PBS at room temperature for 1 h on a nutator, washed four times for 20 min each in PAT (1× PBS, 0.5% PBS Triton, 1% BSA) at room temperature, blocked for 1 h at room temperature with blocking buffer (PAT + 3% Normal Goat Serum) and incubated with primary antibodies, diluted in blocking buffer, overnight on a nutator at 4 °C. The primary antibodies used were Mouse-GFP (SIGMA-ALDRICH, G6539. 1:500 dilution), Rabbit-TH (EMD-Millipore, AB152, 1:200 dilution), and Rat-DN-cadherin (Hybridoma Bank DSHB, DNEX#8, 1:50 dilution). This was followed by four washes for 20 min each in PAT, and incubation overnight on a nutator at 4 °C with secondary antibodies diluted in blocking buffer. The secondary antibodies were all from Molecular Probe and used at 1:500 dilution: Alexa Fluor 488 anti-Mouse (A11029), Alexa Fluor 568 anti-Rabbit (A11036) and Alexa Fluor 633 anti-Rat (A21094). Brains were then washed four times for 20 min each in PAT at room temperature and one time for 20 min in 1× PBS and mounted with VECTASHIELD mounting medium (Vector Laboratories, H-1000). Samples were imaged on a Zeiss 800 confocal laser scanning microscope.

### mini-INTACT

Nuclei were obtained from dopaminergic neurons using INTACT [55] with modifications to enable purification of nuclei from as few as 200–250 heads per ChIP-seq for *TH*-*GAL4* which is expressed in ~ 120 neurons/brain. *Drosophila* males of *3X-UAS-unc84-2XGFP/TH-GAL4* genotype were either socially isolated or group housed and flash frozen during the morning activity peak. Frozen heads were collected over dry ice-cooled sieves from vortex-decapitated flies and added to 5 ml of mini-INTACT buffer consisting of 5 mM β-glycerophosphate pH 7.0, 2 mM MgCl2, 1× complete protease inhibitor cocktail (Roche 11873580001), 5 mM sodium butyrate, 0.6 mM spermidine, 0.2 mM spermine, 0.5% NP-40, and 0 .6mM β-mercaptoethanol. The suspension was passed over a modified “mini-INTACT” homogenizer, set at 1000 rpm, ten to 12 times. The Teflon homogenizer was modified such that the grooves at the bottom of the homogenizer helped push fly heads upward increasing the efficiency of homogenization and preventing sample loss (Additional files 1 and 2 [121]). Homogenate was filtered through a 20-μm filter (Partec CellTrics, Sysmex 25004-0042-2315) and then a 10-μm filter (Partec CellTrics, Sysmex 04-0042-2314). One microgram of anti-GFP antibody (Invitrogen G10362) was added to the filtered homogenate; tubes were gently inverted 10 times and incubated on ice for 20 min to allow binding. To this mix, 30 μl of Dynabeads Protein-G (Invitrogen 100-03D) was added and incubated at 4 °C for 30 min with constant end-over-end rotation. Beads were then collected on a magnet (Diagenode B04000003) and washed thrice using mini-INTACT buffer. Bead-bound nuclei were resuspended in 1 ml INTACT buffer and formaldehyde fixed for ChIP-seq as described in the next section.

### ChIP-Seq

For each ChIP-seq reaction, ~ 10,000–15,000 mini-INTACT isolated bead-bound nuclei were processed using Low Cell # ChIP kit (Diagenode C01010070) as per manufacturer’s instructions. In brief, nuclei were fixed in 1% formaldehyde for 2 min, immediately quenched with glycine and then lysed using nuclear lysis buffer with protease inhibitor cocktail at room temperature for 5 min. PBS was added to dilute the lysate-bead mix and loaded in AFA tubes (Covaris Inc. 520045) for sonication. Ultra-sonicator (Covaris Inc. E220) was used to sheer chromatin to ~ 200 bp length, and chromatin was recovered from the supernatant after magnetic separation. ChIP was performed using the following ChIP-seq grade antibodies: H3K4me3 (Diagenode C15410003-50, Lot A5051-001P), H3K9me3 (Diagenode C15410193, Lot A1671-001P), H3K9/K14 ac (Diagenode C15410200, Lot A1756D), H3K27me3 (Diagenode C15410195, Lot A1811-001P), H3K27ac (Diagenode C154410196, Lot A1723-0041D), and H3K36me3 (Diagenode C15410192, Lot A1895P). Two biological replicates were performed for each histone mark, and input DNA was used as the control. Libraries for sequencing were prepared using MicroPlex Library Preparation kit (Diagenode C05010012) as per manufacturer’s instruction. Single-end 60 bp sequencing reads were obtained using Illumina Hi-seq 2500.

### RNA-seq

We isolated cell bodies of dopaminergic neurons using Fluorescence Activated Cell Sorting (FACS) during the flies’ morning activity peak. The protocol was essentially as described [51] with minor modifications. In brief, brains were dissected from socially isolated or group-housed flies expressing membrane-tagged GFP and nuclear tdTomato in their dopaminergic neurons. The flies were obtained by crossing flies carrying *TH-GAL4* with a stock carrying *pJFRC105-10XUAS-IVS-nlstdTomato* in VK40 (gift of Barret D. Pfeiffer, Rubin Lab, Janelia Research Campus) and *pJFRC29-10XUAS-IVS-myr::GFP-p10* in AttP40 [105] and was found to produce better purity in FACS than other reporters [106]. To account for possible manual bias, dissectors switched their handling of group- or single-housed flies in each session. Dissected brains were digested using Liberase DH (Roche 5401054001), manually triturated using glass pipettes, and filtered using a Falcon 35 μm cell strainer (Corning 352235) before sorting. Approximately 1500 dopaminergic neurons were obtained from approximately 30 brains using a BD FACSAria II sorter (BD Biosciences, USA). Total RNA was extracted using the Arctus, PicoPure RNA Isolation Kit (Thermo Fisher Scientific 12204-01), ERCC spike-in controls were added and cDNA libraries from this material were prepared using Ovation RNA-seq System V2 (Nugen: 7102) as per manufacturer’s instructions. Three biological replicates were performed for each condition. Paired-end 100 bp sequencing reads were obtained using Illumina Hi-seq 2500.

### Sleep assay

Flies that were previously socially isolated or group housed for 4 days were anesthetized briefly with carbon dioxide and transferred into 5 mm × 65 mm transparent plastic tubes with standard cornmeal dextrose agar media. For recording locomotor activity, *Drosophila* activity monitors (Trikinetics, Waltham, USA) were kept in incubators at 25 °C with 65% relative humidity in a 12/12-h light/dark cycle. Flies were allowed one night to acclimatize to the apparatus, and activity data was collected in 1 min bins for the following 24 h as described [107]. One sleep bout was defined as 5 min of continuous inactivity [108, 109]. Statistical analysis of the sleep data was performed using Prism 7 (GraphPad software) and R scripts [110].

#### Bioinformatics

##### Sequencing analysis

All genomic procedures used release 6.02 of the *Drosophila melanogaster* genome [72]. R 3.0.3 was used in scripts and statistics [110]. Non-parametric statistical tests were used except where noted. STAR [111] was used for alignment of RNA-seq data. Total counts of de-duplicated reads were calculated at each genome position using Rsubread [112], followed by differential expression calls using edgeR [76]. We cross-checked differential expression using the CyberT [77] and FCROS [78] packages. Normalization between replicates and treatments was performed using default methods (TMM) in edgeR to correct for coverage levels. CuffDiff [113] was used to detect changes in splicing. Bowtie [114] was used to align ChIP-seq reads, and DiffReps [115] and ngs.plot [66] were used to quantify ChIP-seq reads. Changes to DiffReps and ngs.plot databases and code were required to use *Drosophila* genome release 6.02 and are included in Additional file 20. SICER [71, 116] was also run to cross-check DiffReps results (Additional file 8).

##### Clustering

Gene Ontology (GO) analysis was done using two web tools: DAVID [79] and GOrilla [80]. For mRNA differential expression analysis, genes in the top 40% of expression level were used as the background lists for both tools. The percent of expressed genes per tissue has been estimated to be between 30 and 40%, depending on the tissue and the sensitivity of detection [122]. In our own data, genes in the bottom 60% by FPKM had a within-treatment mean signal-to-noise ratio of less than 1 (as measured by the coefficient of variation), so we felt it was conservative to use the top 40% of expressed genes as our analysis cutoff. Genes in the top 40% with FCROS significant differential expression (FDR = 0.2) were analyzed. For analysis of clusters (see below), genes belonging to each cluster were compared to the appropriate background list (top 40% genes for 8-clusters). *K*-means clustering [117] was done using the *k*-means package in R. To understand the impact of social isolation on epigenetics of genes expressed in dopaminergic neurons, a dataset of the top 40% of genes by mRNA TPM expression (5372 genes) was constructed containing normalized differences between group-housed and isolated flies for mRNA and for the six epigenetic marks. Tests using an information criterion approach (BIC) were used to determine the optimal numbers of clusters, which was *k* = 8 for the 5372-gene dataset. *K*-means clustering is a stochastic process that may yield very different results each time it is run if there is no strong pattern in the data. To determine the robustness of the gene assignments to clusters, we re-ran clustering with random seed changes to create N cluster assignments. We then compared each cluster assignment to every other (1035 = *N* × (*N* − 1)/2 comparisons for eight-cluster assignments). In each comparison, we calculated the percent overlap of a cluster in assignment i with clusters in assignment j, and reported the maximum percent overlap for that cluster. We therefore generated 8280 = 8 × 1035 comparisons. Additional file 11 shows a histogram of cluster overlap percentages. For eight clusters, the median percent overlap of a cluster in one assignment to its best match in a second assignment was 94% and was > 99%, 72% of the time. Thus, we concluded that cluster identity is fairly stable, in spite of the randomness inherent in the k-means algorithm.

Cluster functional enrichment was determined using the DAVID 6.8 functional annotation tool [79] using biological, cellular, and molecular function levels 5 plus chromosome location, and using functional annotation clustering. For the gene clusters, the GO analysis by DAVID used the 5372 highest expression genes as background. Results are reported using thresholds for individual categories FDR < 0.05 and enrichment value > 2.0 for functional clusters.

##### Motif analysis

We used the MEME suite of tools [118] to find putative transcription factor binding sites in promoters of the 8 gene clusters found by k-means. CentriMo 4.12.0 [82] was used with promoter-proximal (± 500 bp from TSS) sequences of genes. We used databases of TF binding motifs from Fly Factor Survey 2014 [86, 119] supplemented by motifs determined in a recent study [87]. Promoter-proximal sequences of each gene in a cluster (“test genes”) were tested for motif enrichment using CentriMo compared to a control set of sequences from an equal number of randomly selected genes not in the cluster (“control genes”). We report a motif as “enriched” if the CentriMo’s adjusted *p*-value was < 1 × 10^−10^. We filtered TFs by a fold change of more than 33%, which was the median fold change for the top 40% of expressed genes.

To quantify the number of potential binding sites of each enriched motif in each gene, we used FIMO version 4 [120] with default parameters. Log fold changes in mRNA levels between group housed and single housed treatments were the dependent variable in multilinear regressions in which numbers of each enriched TF motifs were used as dependent variables. The “lm” program from R was used; non-significant dependent variables were removed in a step-wise manner using “stepAIC” (least significant first) until only significant variables remained; the results of these regressions are reported with multilinear r (square root of the proportion of variance explained by the regression) and F-test p-value. Full tables of regression fits are provided in Additional file 16.

## Additional files

Additional file 1: Design of homogenizer used in mini-INTACT. Diagram illustrating details of the homogenizer used in the mini-INTACT protocol. (PDF 637 kb)

Additional file 3: Comparison of tagged GFP expression in adult *Drosophila* brain. The INTACT transgene (*unc84-2XGFP*) was driven in dopaminergic neurons (*TH-GAL4*) using different copy numbers of the UAS promoter and expression of GFP was compared using the same imaging settings. (A) *3X-UAS*- (B) *5X-UAS*- and (C) *10X-UAS-unc84-2XGFP*. The *3X-UAS-unc84-2XGFP* transgene most faithfully reproduced *TH*-*GAL4* expression, while ectopic expression was observed upon further increases of the *UAS* copy numbers. Dopaminergic neurons were stained with anti-TH antibodies (red), INTACT transgene expression using anti-GFP antibodies (green), and N-cadherin (blue) was used as reference. See Fig. 1b for *3X-UAS-unc84-2XGFP* brain imaged at higher intensity. Scale bar is 20 μm. (PDF 5243 kb)

Additional file 4: *3XUAS-unc84-2XGFP* expression in dopaminergic neurons did not affect daytime sleep or activity over 24 h. (A) Daytime sleep measured over a 12 h period. GH males slept more than SH males during the daytime. No significant difference was observed due to tagged-GFP expression. (B) Total number of activity counts (beam breaks) over 24 h. GH are less active than SH flies as expected. No significant difference was observed due to tagged-GFP expression. *N* = 31–32. Unpaired t-test. (PDF 438 kb)

Additional file 5: Purity assessment of dopaminergic nuclei. The table shows the number of captured green dopaminergic nuclei using bead-bound anti-GFP antibodies. Most of the contaminating red nuclei were washed away from bead-bound affinity-purified nuclei. See Fig. 1 and main text for details (3 biological replicates). (DOCX 11 kb)

Additional file 6: Replicate concordance for ChIP-seq for various histone modifications. ChIP-seq replicate concordance is shown with Pearson’s correlation coefficient (r-values) calculated on Log (1 + ngs.plot) enrichment values for all six histone marks. (PDF 7315 kb)

Additional file 7: Differentially marked Islands called by SICER. Table of islands called as differentially marked by SICER, with genomic location, mark levels, and mark type. (XLS 3107 kb)

Additional file 8: Diffreps results. Table of regions with significant mark island differences between GH and SH males, as reported by Diffreps. (XLS 665 kb)

Additional file 9: Differentially Expressed Genes. Contains TPM values for each replicate for genes called as differentially expressed by one of edgeR, CyberT, or FCros. (XLS 1458 kb)

Additional file 10: Gorilla and DAVID functional analysis. The zip file contains top level html files which may be opened in a browser. These will give the Gorilla functional analysis and DAVID GO analyses referred to in the main text. (ZIP 919 kb)

Additional file 11: k-means cluster overlap. The figure shows a histogram of k-means cluster overlap percentages used to calculate robustness of gene assignments to clusters. For eight clusters, the median percent overlap of a cluster in one assignment to its best match in a second assignment was 94%, and was greater than 99% 72% of the time (see the “Materials and Methods” section for details). (PDF 101 kb)

Additional file 12: TFs overrepresented in k-means clusters. The tab-delimited table shows the level of overrepresentation (column 3) of genes with putative binding motifs of the TF in column 2, in each of the 8 k-means clusters (column 1). (TXT 1 kb)

Additional file 13: ARG gene fold changes. Column B gives fold changes (FC) between Group and Single housed in our experiments, for 9 genes labeled ARG in Croset et al. [84]. (XLS 30 kb)

Additional file 14: Genes repressed by cbt. Columns C-E and F-H are from Bartok et al. 2015. C-E are transcript levels in controls, F-H are transcript levels in cbt-RNAi. Columns I-O show mean normalized log2 FC between GH and SH in our experiments. (XLS 383 kb)

Additional file 15: Genes activated by cbt. Columns C-E and F-H are from Bartok et al. 2015. C-E are transcript levels in controls, F-H are transcript levels in cbt-RNAi. Columns I-O show mean normalized log2 FC between GH and SH in our experiments. Worksheet 2 gives statistics and a bar chart of the data for comparisons between repressed and activated by Cbt gene groups, for mRNA and for the 6 epigenetic marks. (XLS 145 kb)

Additional file 16: Multiple regressions on TF putative binding sites. Gives details (slope, t, F, p) of multiple regressions of the change in transcription for genes in the named group, based on independent variables the number of putative TF binding sites for the named TFs. (TXT 3 kb)

Additional file 17: ANOVA results for RNAi knockdown effects on sleep. Results of performing Type III ANOVA to detect the interaction effect of *TH-GAL4* and UAS-RNAi on Δ sleep. See detailed comments at beginning of file. (TXT 6 kb)

Additional file 18: Sleep over 24 h for UAS-ARG-RNAi and controls. (A) and (B) shows sleep per 30 min over 24 h associated with main Fig. 5c. Control single housed (SH) flies sleep less than group housed (GH) flies during the day (shaded gray area). This difference is delta-sleep. Expressing RNAi for ARG-TFs: *Hr38*, *sr*, *CrebA* in dopaminergic neurons significantly reduced this difference. RNAi hairpins against candidate genes were present in attP2 and attP40 sites respectively, driven with *TH-GAL4*. Corresponding background controls were without RNAi hairpin, but with the attP2 or attP40 inserts, driven with *TH-GAL4* (see the “Materials and Methods” section). Activity counts over 24 h are shown in (C), (D) and (E) for *UAS-RNAi* vs. corresponding controls driven by *TH-GAL4*. (C) *UAS-CrebA* (2)*-RNAi* vs. control. ****, *p* < 0.0001, GH (Student’s t-test, *n* = 45–48). (D) *UAS-ARG-RNAi* vs. control. **, *p* = 0.0059, *Hr38*-GH; ****, p < 0.0001, *sr*-GH; *, *p* = 0.0187, *CrebA*(1)-GH; *,*p* = 0.0378, *Hr38*-SH; *, *p* = 0.0103 (One way ANOVA with Dunnett’s multiple comparisons test, *n* = 44–48). (E) *UAS-cbt-RNAi* vs. control, not significant; *UAS-Brms1-RNAi* vs. control, GH, ****, *p* < 0.0001; SH, *p* = 0.0121 (One way ANOVA with Dunnett’s multiple comparisons test, *n* = 31–32). (PDF 1625 kb)

Additional file 19: Knockdown of epigenetic eraser *Brms1* by RNAi reduced social effects on daytime sleep. *Brms1* is a member of *Sin3A* histone deacetylase complex. Knockdown of *Brms1* in dopaminergic neurons was achieved by driving an RNAi transgene with *TH-GAL4*; controls carried empty vectors without RNAi hairpin and *TH-GAL4*. (A) Sleep per 30 min over 24 h for control and *Brms1* knockdown in SH and GH flies. Daytime sleep is highlighted in shaded gray area for both genotypes. (B) Expressing RNAi for *Brms1* in dopaminergic neurons reduced the social effect of sleep during the day (ΔSleep). Error bars are mean ± SEM. (PDF 743 kb)

Additional file 20: Changes for Diffreps and Ngs.plot for *Drosophila* genome 6.02. Minor changes were made in Diffreps and ngs.plot local copies to add *D. melanogaster* genome release 6.02 to the set of genomes. (ZIP 6257 kb)
