# Supplementary figures and images for: Enabling cell-type-specific behavioral epigenetics in Drosophila: a modified high-yield INTACT method reveals the impact of social environment on the epigenetic landscape in dopaminergic neurons
Source: BMC Biol. 2019 Apr 10;17:30. doi: 10.1186/s12915-019-0646-4 (PMC6456965; doi:10.1186/s12915-019-0646-4)

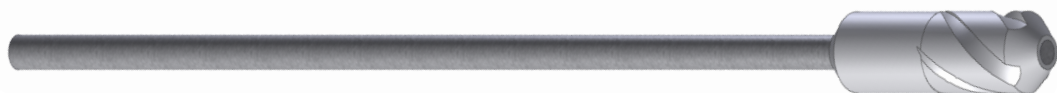

Supplement: Supplementary file 2 — Design of homogenizer used in mini-INTACT. Diagram illustrating details of the homogenizer used in the mini-INTACT protocol. (PDF 637 kb) [file 12915_2019_646_MOESM1_ESM.pdf]

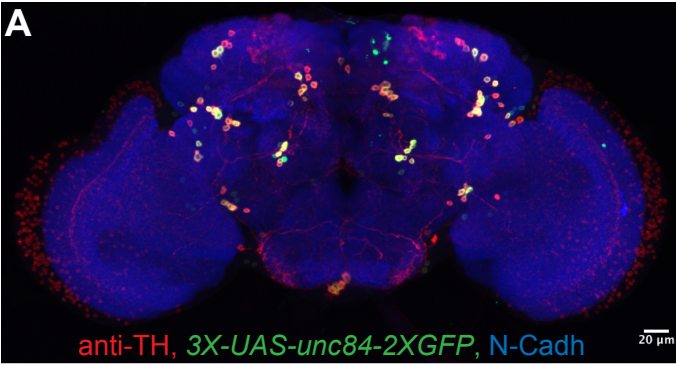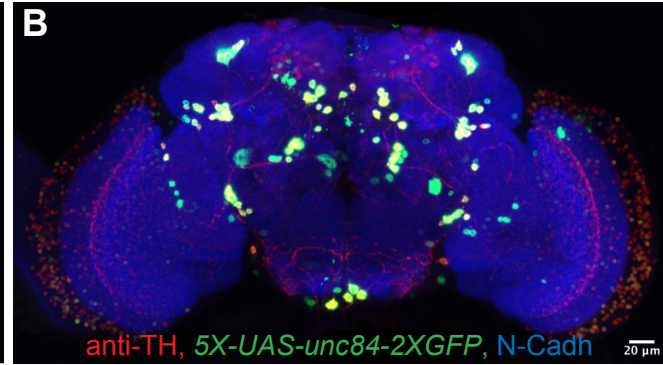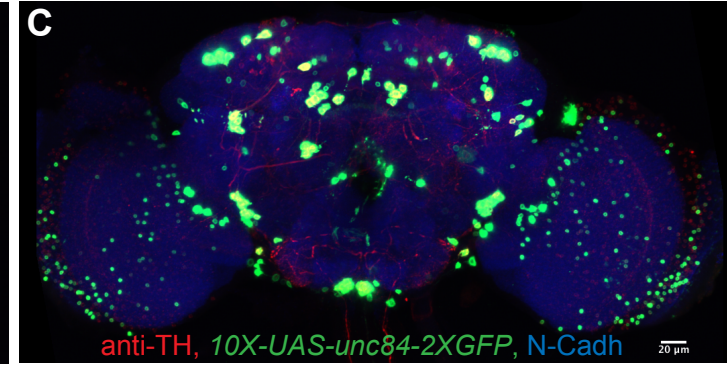

Supplement: Supplementary file 3 — Comparison of tagged GFP expression in adult Drosophila brain. The INTACT transgene (unc84-2XGFP) was driven in dopaminergic neurons (TH-GAL4) using different copy numbers of the UAS promoter and expression of GFP was compared using the same imaging settings. (A) 3X-UAS- (B) 5X-UAS- and (C) 10X-UAS-unc84-2XGFP. The 3X-UAS-unc84-2XGFP transgene most faithfully reproduced TH-GAL4 expression, while ectopic expression was observed upon further increases of the UAS copy numbers. Dopaminergic neurons were stained with anti-TH antibodies (red), INTACT transgene expression using anti-GFP antibodies (green), and N-cadherin (blue) was used as reference. See Fig. 1b for 3X-UAS-unc84-2XGFP brain imaged at higher intensity. Scale bar is 20 μm. (PDF 5243 kb) [file 12915_2019_646_MOESM3_ESM.pdf]

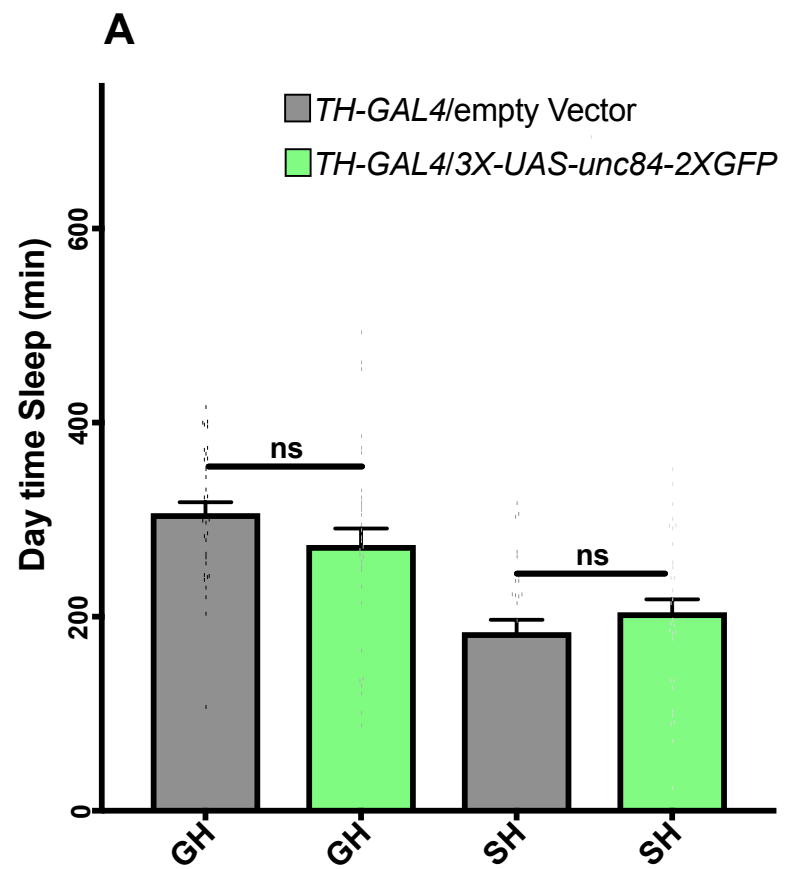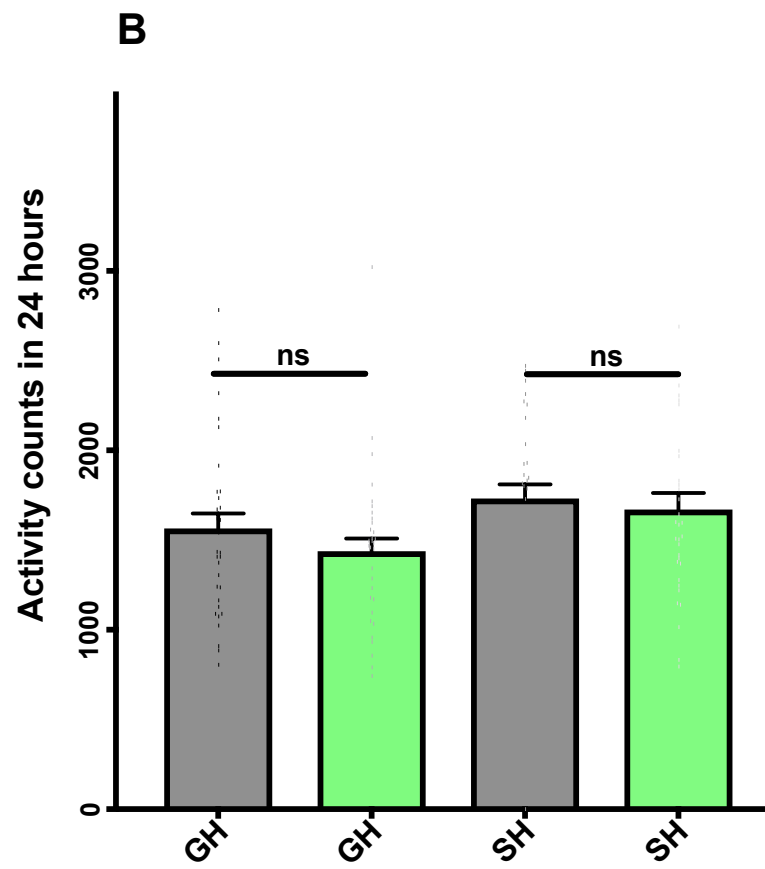

Supplement: Supplementary file 4 — 3XUAS-unc84-2XGFP expression in dopaminergic neurons did not affect daytime sleep or activity over 24 h. (A) Daytime sleep measured over a 12 h period. GH males slept more than SH males during the daytime. No significant difference was observed due to tagged-GFP expression. (B) Total number of activity counts (beam breaks) over 24 h. GH are less active than SH flies as expected. No significant difference was observed due to tagged-GFP expression. N = 31–32. Unpaired t-test. (PDF 438 kb) [file 12915_2019_646_MOESM4_ESM.pdf]

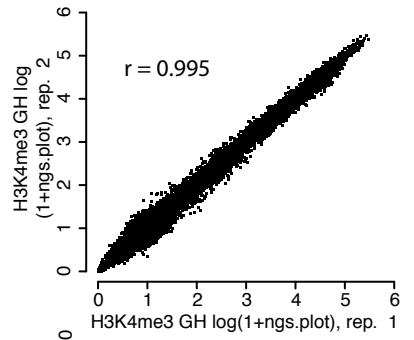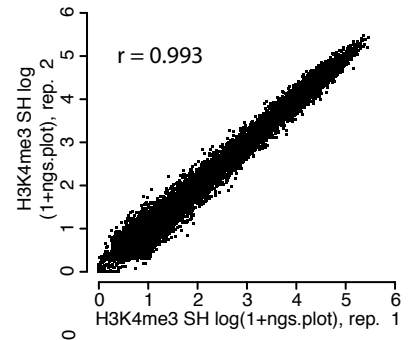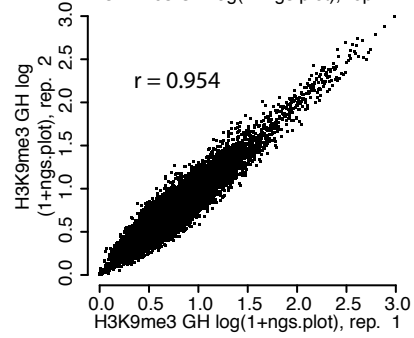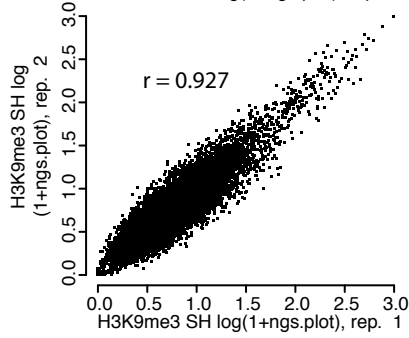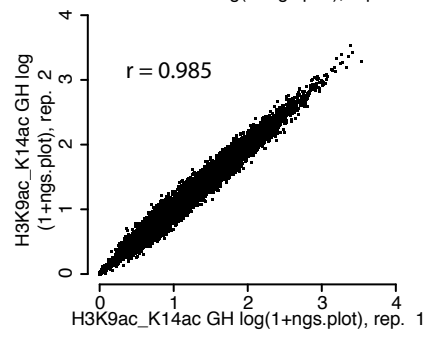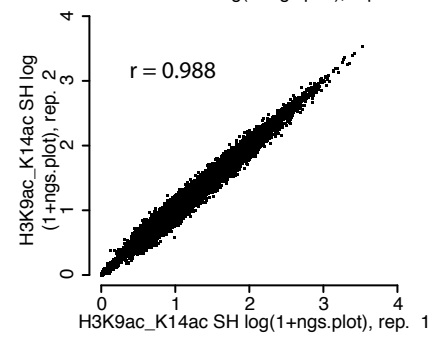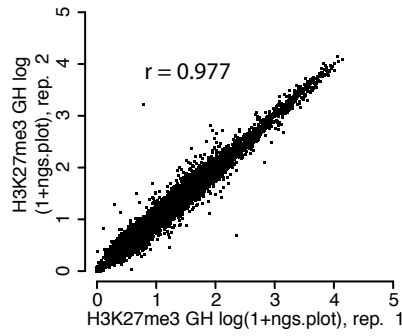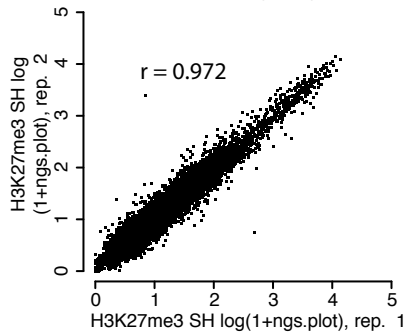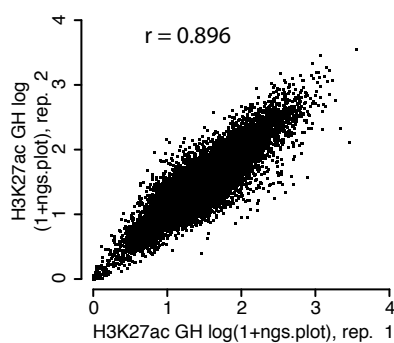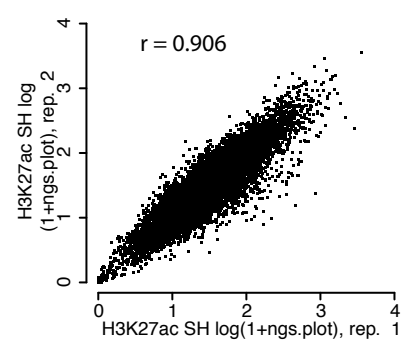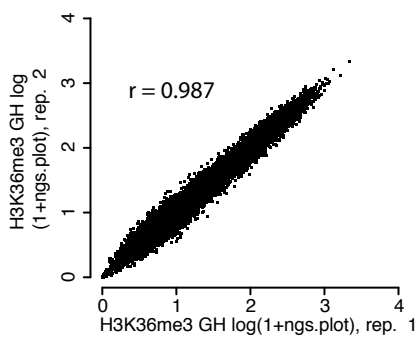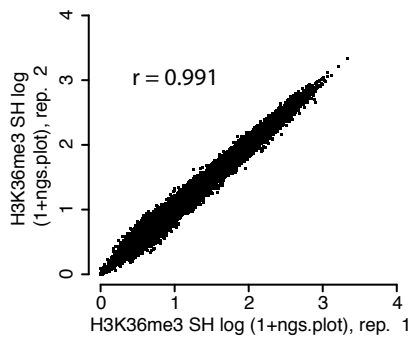

Supplement: Supplementary file 6 — Replicate concordance for ChIP-seq for various histone modifications. ChIP-seq replicate concordance is shown with Pearson’s correlation coefficient (r-values) calculated on Log (1 + ngs.plot) enrichment values for all six histone marks. (PDF 7315 kb) [file 12915_2019_646_MOESM6_ESM.pdf]

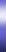

Supplement: Supplementary file 10 — Gorilla and DAVID functional analysis. The zip file contains top level html files which may be opened in a browser. These will give the Gorilla functional analysis and DAVID GO analyses referred to in the main text. (ZIP 919 kb) [file 12915_2019_646_MOESM10_ESM.zip › Additional File 10/TPM81_GOLevel5_files/two_tone_2_a.jpg]

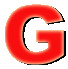

Supplement: Supplementary file 10 — Gorilla and DAVID functional analysis. The zip file contains top level html files which may be opened in a browser. These will give the Gorilla functional analysis and DAVID GO analyses referred to in the main text. (ZIP 919 kb) [file 12915_2019_646_MOESM10_ESM.zip › Additional File 10/TPM81_GOLevel5_files/G.gif]

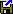

Supplement: Supplementary file 10 — Gorilla and DAVID functional analysis. The zip file contains top level html files which may be opened in a browser. These will give the Gorilla functional analysis and DAVID GO analyses referred to in the main text. (ZIP 919 kb) [file 12915_2019_646_MOESM10_ESM.zip › Additional File 10/TPM81_GOLevel5_files/download.gif]

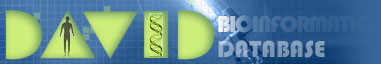

Supplement: Supplementary file 10 — Gorilla and DAVID functional analysis. The zip file contains top level html files which may be opened in a browser. These will give the Gorilla functional analysis and DAVID GO analyses referred to in the main text. (ZIP 919 kb) [file 12915_2019_646_MOESM10_ESM.zip › Additional File 10/TPM81_GOLevel5_files/david_logo2.jpg]

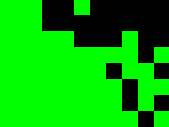

Supplement: Supplementary file 10 — Gorilla and DAVID functional analysis. The zip file contains top level html files which may be opened in a browser. These will give the Gorilla functional analysis and DAVID GO analyses referred to in the main text. (ZIP 919 kb) [file 12915_2019_646_MOESM10_ESM.zip › Additional File 10/TPM81_GOLevel5_files/2Dview.GIF]

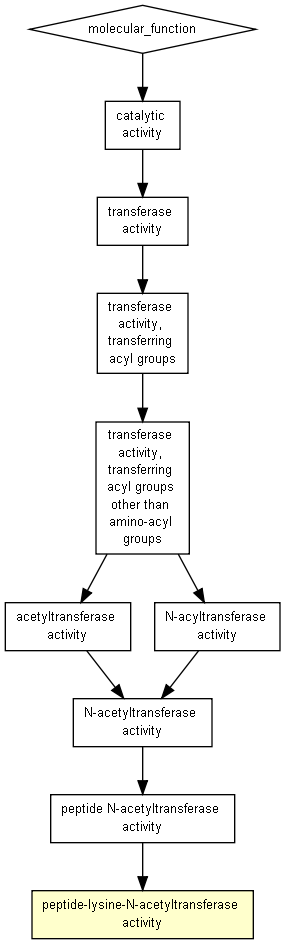

Supplement: Supplementary file 10 — Gorilla and DAVID functional analysis. The zip file contains top level html files which may be opened in a browser. These will give the Gorilla functional analysis and DAVID GO analyses referred to in the main text. (ZIP 919 kb) [file 12915_2019_646_MOESM10_ESM.zip › Additional File 10/FDR20Function_files/GOFUNCTION.png]

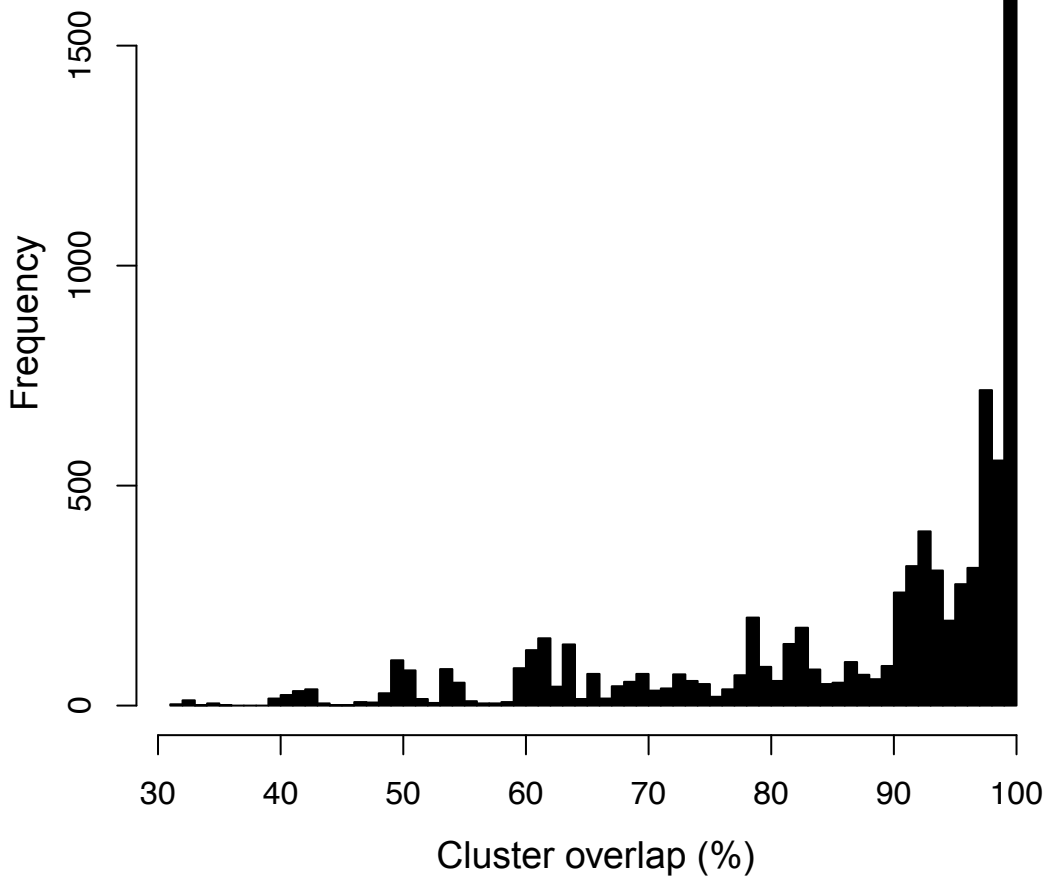

Supplement: Supplementary file 11 — k-means cluster overlap. The figure shows a histogram of k-means cluster overlap percentages used to calculate robustness of gene assignments to clusters. For eight clusters, the median percent overlap of a cluster in one assignment to its best match in a second assignment was 94%, and was greater than 99% 72% of the time (see the “Materials and Methods” section for details). (PDF 101 kb) [file 12915_2019_646_MOESM11_ESM.pdf]

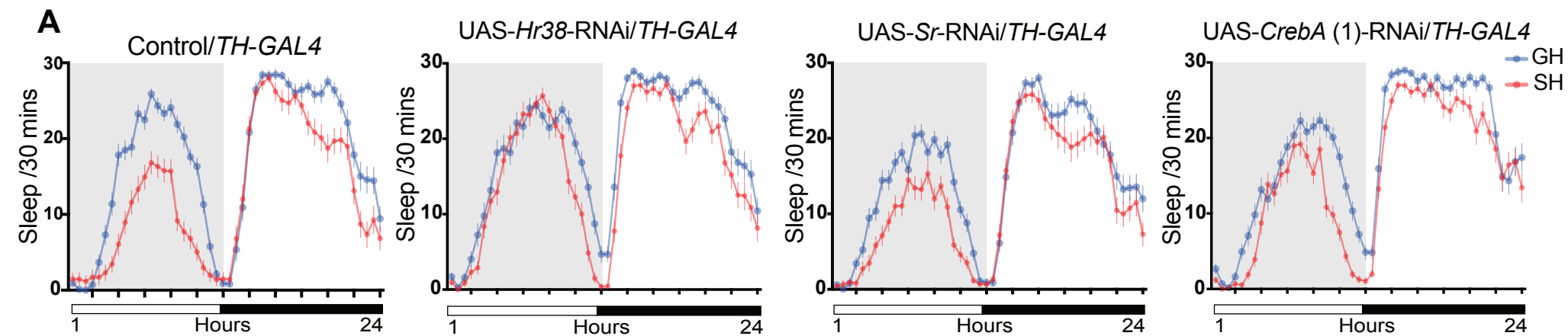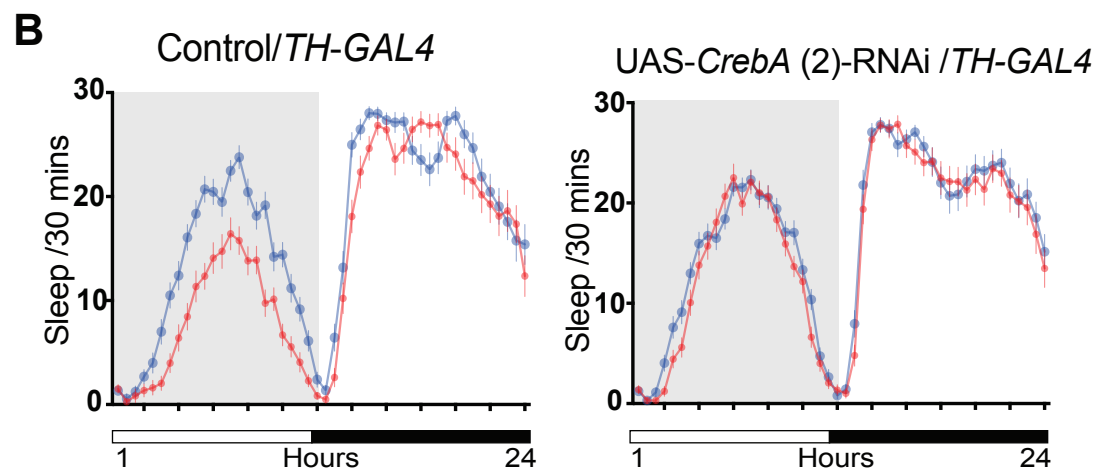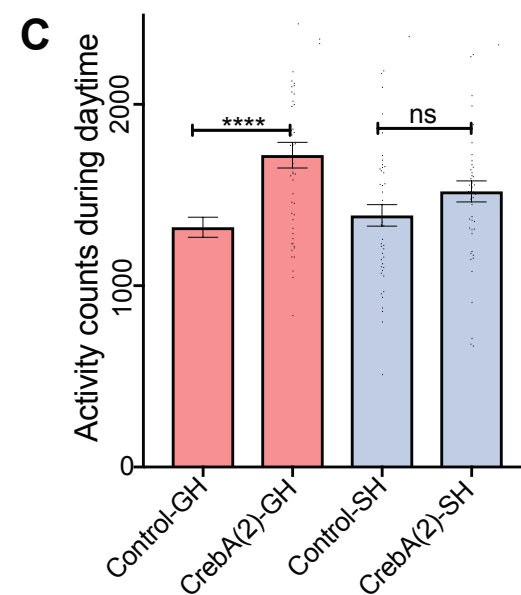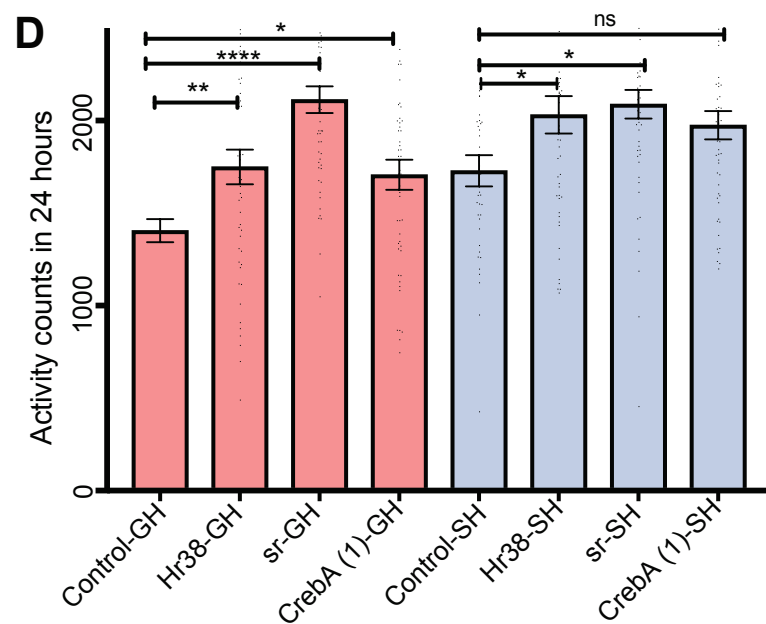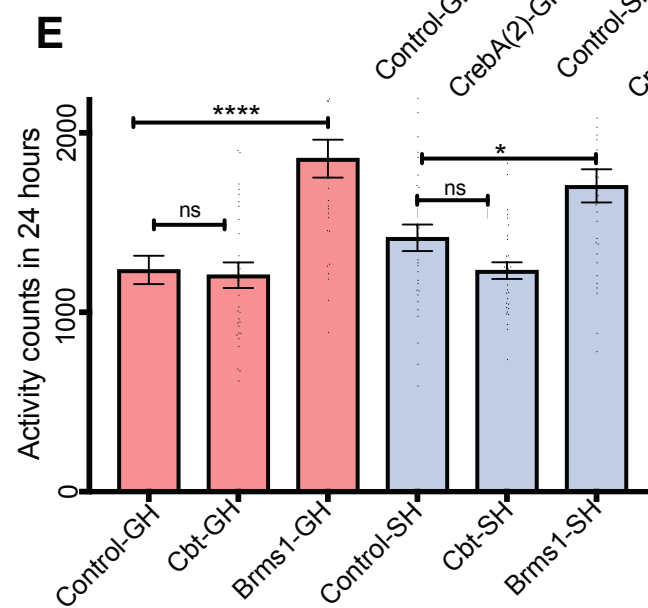

Supplement: Supplementary file 18 — Sleep over 24 h for UAS-ARG-RNAi and controls. (A) and (B) shows sleep per 30 min over 24 h associated with main Fig. 5c. Control single housed (SH) flies sleep less than group housed (GH) flies during the day (shaded gray area). This difference is delta-sleep. Expressing RNAi for ARG-TFs: Hr38, sr, CrebA in dopaminergic neurons significantly reduced this difference. RNAi hairpins against candidate genes were present in attP2 and attP40 sites respectively, driven with TH-GAL4. Corresponding background controls were without RNAi hairpin, but with the attP2 or attP40 inserts, driven with TH-GAL4 (see the “Materials and Methods” section). Activity counts over 24 h are shown in (C), (D) and (E) for UAS-RNAi vs. corresponding controls driven by TH-GAL4. (C) UAS-CrebA (2)-RNAi vs. control. ****, p < 0.0001, GH (Student’s t-test, n = 45–48). (D) UAS-ARG-RNAi vs. control. **, p = 0.0059, Hr38-GH; ****, p < 0.0001, sr-GH; *, p = 0.0187, CrebA(1)-GH; *,p = 0.0378, Hr38-SH; *, p = 0.0103 (One way ANOVA with Dunnett’s multiple comparisons test, n = 44–48). (E) UAS-cbt-RNAi vs. control, not significant; UAS-Brms1-RNAi vs. control, GH, ****, p < 0.0001; SH, p = 0.0121 (One way ANOVA with Dunnett’s multiple comparisons test, n = 31–32). (PDF 1625 kb) [file 12915_2019_646_MOESM18_ESM.pdf]

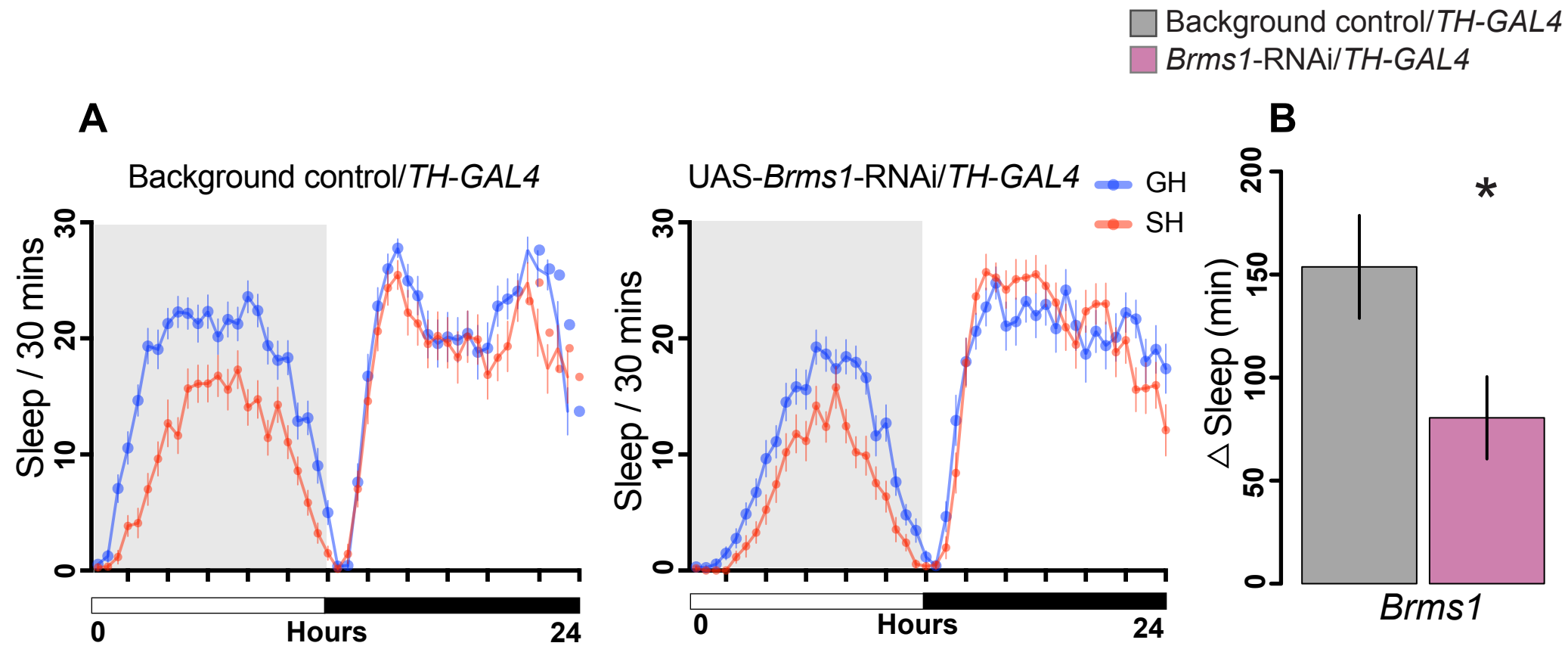

Supplement: Supplementary file 19 — Knockdown of epigenetic eraser Brms1 by RNAi reduced social effects on daytime sleep. Brms1 is a member of Sin3A histone deacetylase complex. Knockdown of Brms1 in dopaminergic neurons was achieved by driving an RNAi transgene with TH-GAL4; controls carried empty vectors without RNAi hairpin and TH-GAL4. (A) Sleep per 30 min over 24 h for control and Brms1 knockdown in SH and GH flies. Daytime sleep is highlighted in shaded gray area for both genotypes. (B) Expressing RNAi for Brms1 in dopaminergic neurons reduced the social effect of sleep during the day (ΔSleep). Error bars are mean ± SEM. (PDF 743 kb) [file 12915_2019_646_MOESM19_ESM.pdf]
