# Supplementary material for: Enabling cell-type-specific behavioral epigenetics in Drosophila: a modified high-yield INTACT method reveals the impact of social environment on the epigenetic landscape in dopaminergic neurons
Source: BMC Biol. 2019 Apr 10;17:30. doi: 10.1186/s12915-019-0646-4 (PMC6456965; doi:10.1186/s12915-019-0646-4)
Supplement: Supplementary file 5 — Purity assessment of dopaminergic nuclei. The table shows the number of captured green dopaminergic nuclei using bead-bound anti-GFP antibodies. Most of the contaminating red nuclei were washed away from bead-bound affinity-purified nuclei. See Fig. 1 and main text for details (3 biological replicates). (DOCX 11 kb) [file 12915_2019_646_MOESM5_ESM.docx]

| **Replicate** | **Green nuclei numbers** | **Red nuclei**  **numbers** | **Purity** |
| --- | --- | --- | --- |
| 1 | 482 | 8 | 98.3% |
| 2 | 502 | 12 | 97.6% |
| 3 | 698 | 12 | 98.2% |
