# Supplementary material for: Enabling cell-type-specific behavioral epigenetics in Drosophila: a modified high-yield INTACT method reveals the impact of social environment on the epigenetic landscape in dopaminergic neurons
Source: BMC Biol. 2019 Apr 10;17:30. doi: 10.1186/s12915-019-0646-4 (PMC6456965; doi:10.1186/s12915-019-0646-4)
Supplement: Supplementary file 10 — Gorilla and DAVID functional analysis. The zip file contains top level html files which may be opened in a browser. These will give the Gorilla functional analysis and DAVID GO analyses referred to in the main text. (ZIP 919 kb) [file 12915_2019_646_MOESM10_ESM.zip › Additional File 10/TPM88_GOLevel5.html]

DAVID: Database for Annotation, Visualization, and Integrated Discovery (Laboratory of Human Retrovirology and Immunoinformatics (LHRI); National Institute of Allergies and Infectious Diseases (NIAID); Leidos Biomedical Research, Inc. (LBR)


|  |  |  |  |  |  |
| --- | --- | --- | --- | --- | --- |
| DAVID Bioinformatics 6.7  |  |  |  |  |  | | --- | --- | --- | --- | --- | | |  |  |  |  | | --- | --- | --- | --- | |  | |  | | --- | | DAVID Bioinformatics Resources 6.8 | | Laboratory of Human Retrovirology and Immunoinformatics (LHRI) | | |   100%  **\*\*\* Welcome to DAVID 6.8 \*\*\* \*\*\* If you are looking for DAVID 6.7, please visit our development site. \*\*\*** |
| |  |  |  |  |  |  |  |  |  |  |  |  |  |  |  |  |  |  |  |  |  |  |  |  |  |  |  |  |  |  |  |  |  |  |  |  |  |  |  |  |  |  |  |  |  |  |  |  |  |  |  |  |  |  |  |  |  |  |  |  |  |  |  |  |  |  |  |  |  |  |  |  |  |  |  |  |  |  |  |  |  |  |  |  |  |  |  |  |  |  |  |  |  |  |  |  |  |  |  |  |  |  |  |  |  |  |  |  |  |  |  |  |  |  |  |  |  |  |  |  |  |  |  |  |  |  |  |  |  |  |  |  |  |  |  |  |  |  |  |  |  |  |  |  |  |  |  |  |  |  |  |  |  |  |  |  |  |  |  |  |  |  |  |  |  |  |  |  |  |  |  |  |  |  |  |  |  |  |  |  |  |  |  |  |  |  |  |  |  |  |  |  |  |  |  |  |  |  |  |  |  |  |  |  |  |  |  |  |  |  |  |  |  |  |  |  |  |  |  |  |  |  |  |  |  |  |  |  |  |  |  |  |  |  |  |  |  |  |  |  |  |  |  |  |  |  |  |  |  |  |  |  |  |  |  |  |  |  |  |  |  |  |  |  |  |  |  |  |  |  |  |  |  |  |  |  |  |  |  |  |  |  |  |  |  |  |  |  |  |  |  |  |  |  |  |  |  |  |  |  |  |  |  |  |  |  |  |  |  |  |  |  |  |  |  |  |  |  |  |  |  |  |  |  |  |  |  |  |  |  |  |  |  |  |  |  |  |  |  |  |  |  |  |  |  |  |  |  |  |  |  |  |  |  |  |  |  |  |  |  |  |  |  |  |  |  |  |  |  |  |  |  |  |  |  |  |  |  |  |  |  |  |  |  |  |  |  |  |  |  |  |  |  |  |  |  |  |  |  |  |  |  |  |  |  |  |  |  |  |  |  |  |  |  |  |  |  |  |  |  |  |  |  |  |  |  |  |  |  |  |  |  |  |  |  |  |  |  |  |  |  |  |  |  |  |  |  |  |  |  |  |  |  |  |  |  |  |  |  |  |  |  |  |  |  |  |  |  |  |  |  |  |  |  |  |  |  |  |  |  |  |  |  |  |  |  |  |  |  |  |  |  |  |  |  |  |  |  |  |  |  |  |  |  |  |  |  |  |  |  |  |  |  |  |  |  |  |  |  |  |  |  |  |  |  |  |  |  |  |  |  |  |  |  |  |  |  |  |  |  |  |  |  |  |  |  |  |  |  |  |  |  |  |  |  |  |  |  |  |  |  |  |  |  |  |  |  |  |  |  |  |  |  |  |  |  |  |  |  |  |  |  |  |  |  |  |  |  |  |  |  |  |  |  |  |  |  |  |  |  |  |  |  |  |  |  |  |  |  |  |  |  |  |  |  |  |  |  |  |  |  |  |  |  |  |  |  |  |  |  |  |  |  |  |  |  |  |  |  |  |  |  |  |  |  |  |  |  |  |  |  |  |  |  |  |  |  |  |  |  |  |  |  |  |  |  |  |  |  |  |  |  |  |  |  |  |  |  |  |  |  |  |  |  |  |  |  |  |  |  |  |  |  |  |  |  |  |  |  |  |  |  |  |  |  |  |  |  |  |  |  |  |  |  |  |  |  |  |  |  |  |  |  |  |  |  |  |  |  |  |  |  |  |  |  |  |  |  |  |  |  |  |  |  |  |  |  |  |  |  |  |  |  |  |  |  |  |  |  |  |  |  |  |  |  |  |  |  |  |  |  |  |  |  |  |  |  |  |  |  |  |  |  |  |  |  |  |  |  |  |  |  |  |  |  |  |  |  |  |  |  |  |  |  |  |  |  |  |  |  |  |  |  |  |  |  |  |  |  |  |  |  |  |  |  |  |  |  |  |  |  |  |  |  |  |  |  |  |  |  |  |  |  |  |  |  |  |  |  |  |  |  |  |  |  |  |  |  |  |  |  |  |  |  |  |  |  |  |  |  |  |  |  |  |  |  |  |  |  |  |  |  |  |  |  |  |  |  |  |  |  |  |  |  |  |  |  |  |  |  |  |  |  |  |  |  |  |  |  |  |  |  |  |  |  |  |  |  |  |  |  |  |  |  |  |  |  |  |  |  |  |  |  |  |  |  |  |  |  |  |  |  |  |  |  |  |  |  |  |  |  |  |  |  |  |  |  |  |  |  |  |  |  |  |  |  |  |  |  |  |  |  |  |  |  |  |  |  |  |  |  |  |  |  |  |  |  |  |  |  |  |  |  |  |  |  |  |  |  |  |  |  |  |  |  |  |  |  |  |  |  |  |  |  |  |  |  |  |  |  |  |  |  |  |  |  |  |  |  |  |  |  |  |  |  |  |  |  |  |  |  |  |  |  |  |  |  |  |  |  |  |  |  |  |  |  |  |  |  |  |  |  |  |  |  |  |  |  |  |  |  |  |  |  |  |  |  |  |  |  |  |  |  |  |  |  |  |  |  |  |  |  |  |  |  |  |  |  |  |  |  |  |  |  |  |  |  |  |  |  |  |  |  |  |  |  |  |  |  |  |  |  |  |  |  |  |  |  |  |  |  |  |  |  |  |  |  |  |  |  |  |  |  |  |  |  |  |  |  |  |  |  |  |  |  |  |  |  |  |  |  |  |  |  |  |  |  |  |  |  |  |  |  |  |  |  |  |  |  |  |  |  |  |  |  |  |  |  |  |  |  |  |  |  |  |  |  |  |  |  |  |  |  |  |  |  |  |  |  |  |  |  |  |  |  |  |  |  |  |  |  |  |  |  |  |  |  |  |  |  |  |  |  |  |  |  |  |  |  |  |  |  |  |  |  |  |  |  |  |  |  |  |  |  |  |  |  |  |  |  |  |  |  |  |  |  |  |  |  |  |  |  |  |  |  |  |  |  |  |  |  |  |  |  |  |  |  |  |  |  |  |  |  |  |  |  |  |  |  |  |  |  |  |  |  |  |  |  |  |  |  |  |  |  |  |  |  |  |  |  |  |  |  |  |  |  |  |  |  |  |  |  |  |  |  |  |  |  |  |  |  |  |  |  |  |  |  |  |  |  |  |  |  |  |  |  |  |  |  |  |  |  |  |  |  |  |  |  |  |  |  |  |  |  |  |  |  |  |  |  |  |  |  |  |  |  |  |  |  |  |  |  |  |  |  |  |  |  |  |  |  |  |  |  |  |  |  |  |  |  |  |  |  |  |  |  |  |  |  |  |  |  |  |  |  |  |  |  |  |  |  |  |  |  |  |  |  |  |  |  |  |  |  |  |  |  |  |  |  |  |  |  |  |  |  |  |  |  |  |  |  |  |  |  |  |  |  |  |  |  |  |  |  |  |  |  |  |  |  |  |  |  |  |  |  |  |  |  |  |  |  |  |  |  |  |  |  |  |  |  |  |  |  |  |  |  |  |  |  |  |  |  |  |  |  |  |  |  |  |  |  |  |  |  |  |  |  |  |  |  |  |  |  |  |  |  |  |  |  |  |  |  |  |  |  |  |  |  |  |  |  |  |  |  |  |  |  |  |  |  |  |  |  |  |  |  |  |  |  |  |  |  |  |  |  |  |  |  |  |  |  |  |  |  |  |  |  |  |  |  |  |  |  |  |  |  |  |  |  |  |  |  |  |  |  |  |  |  |  |  |  |  |  |  |  |  |  |  |  |  |  |  |  |  |  |  |  |  |  |  |  |  |  |  |  |  |  |  |  |  |  |  |  |  |  |  |  |  |  |  |  |  |  |  |  |  |  |  |  |  |  |  |  |  |  |  |  |  |  |  |  |  |  |  |  |  |  |  |  |  |  |  |  |  |  |  |  |  |  |  |  |  |  |  |  |  |  |  |  |  |  |  |  |  |  |  |  |  |  |  |  |  |  |  |  |  |  |  |  |  |  |  |  |  |  |  |  |  |  |  |  |  |  |  |  |  |  |  |  |  |  |  |  |  |  |  |  |  |  |  |  |  |  |  |  |  |  |  |  |  |  |  |  |  |  |  |  |  |  |  |  |  |  |  |  |  |  |  |  |  |  |  |  |  |  |  |  |  |  |  |  |  |  |  |  |  |  |  |  |  |  |  |  |  |  |  |  |  |  |  |  |  |  |  |  |  |  |  |  |  |  |  |  |  |  |  |  |  |  |  |  |  |  |  |  |  |  |  |  |  |  |  |  |  |  |  |  |  |  |  |  |  |  |  |  |  |  |  |  |  |  |  |  |  |  |  |  |  |  |  |  |  |  |  |  |  |  |  |  |  |  |  |  |  |  |  |  |  |  |  |  |  |  |  |  |  |  |  |  |  |  |  |  |  |  |  |  |  |  |  |  |  |  |  |  |  |  |  |  |  |  |  |  |  |  |  |  |  |  |  |  |  |  |  |  |  |  |  |  |  |  |  |  |  |  |  |  |  |  |  |  |  |  |  |  |  |  |  |  |  |  |  |  |  |  |  |  |  |  |  |  |  |  |  |  |  |  |  |  |  |  |  |  |  |  |  |  |  |  |  |  |  |  |  |  |  |  |  |  |  |  |  |  |  |  |  |  |  |  |  |  |  |  |  |  |  |  |  |  |  |  |  |  |  |  |  |  |  |  |  |  |  |  |  |  |  |  |  |  |  |  |  |  |  |  |  |  |  |  |  |  |  |  |  |  |  |  |  |  |  |  |  |  |  |  |  |  |  |  |  |  |  |  |  |  |  |  |  |  |  |  |  |  |  |  |  |  |  |  |  |  |  |  |  |  |  |  |  |  |  |  |  |  |  |  |  |  |  |  |  |  |  |  |  |  |  |  |  |  |  |  |  |  |  |  |  |  |  |  |  |  |  |  |  |  |  |  |  |  |  |  |  |  |  |  |  |  |  |  |  |  |  |  |  |  |  |  |  |  |  |  |  |  |  |  |  |  |  |  |  |  |  |  |  |  |  |  |  |  |  |  |  |  |  |  |  |  |  |  |  |  |  |  |  |  |  |  |  |  |  |  |  |  |  |  |  |  |  |  |  |  |  |  |  |  |  |  |  |  |  |  |  |  |  |  |  |  |  |  |  |  |  |  |  |  |  |  |  |  |  |  |  |  |  |  |  |  |  |  |  |  |  |  |  |  |  |  |  |  |  |  |  |  |  |  |  |  |  |  |  |  |  |  |  |  |  |  |  |  |  |  |  |  |  |  |  |  |  |  |  |  |  |  |  |  |  |  |  |  |  |  |  |  |  |  |  |  |  |  |  |  |  |  |  |  |  |  |  |  |  |  |  |  |  |  |  |  |  |  |  |  |  |  |  |  |  |  |  |  |  |  |  |  |  |  |  |  |  |  |  |  |  |  |  |  |  |  |  |  |  |  |  |  |  |  |  |  |  |  |  |  |  |  |  |  |  |  |  |  |  |  |  |  |  |  |  |  |  |  |  |  |  |  |  |  |  |  |  |  |  |  |  |  |  |  |  |  |  |  |  |  |  |  |  |  |  |  |  |  |  |  |  |  |  |  |  |  |  |  |  |  |  |  |  |  |  |  |  |  |  |  |  |  |  |  |  |  |  |  |  |  |  |  |  |  |  |  |  |  |  |  |  |  |  |  |  |  |  |  |  |  |  |  |  |  |  |  |  |  |  |  |  |  |  |  |  |  |  |  |  |  |  |  |  |  |  |  |  |  |  |  |  |  |  |  |  |  |  |  |  |  |  |  |  |  |  |  |  |  |  |  |  |  |  |  |  |  |  |  |  |  |  |  |  |  |  |  |  |  |  |  |  |  |  |  |  |  |  |  |  |  |  |  |  |  |  |  |  |  |  |  |  |  |  |  |  |  |  |  |  |  |  |  |  |  |  |  |  |  |  |  |  |  |  |  |  |  |  |  |  |  |  |  |  |  |  |  |  |  |  |  |  |  |  |  |  |  |  |  |  |  |  |  |  |  |  |  |  |  |  |  |  |  |  |  |  |  |  |  |  |  |  |  |  |  |  |  |  |  |  |  |  |  |  |  |  |  |  |  |  |  |  |  |  |  |  |  |  |  |  |  |  |  |  |  |  |  |  |  |  |  |  |  |  |  |  |  |  |  |  |  |  |  |  |  |  |  |  |  |  |  |  |  |  |  |  |  |  |  |  |  |  |  |  |  |  |  |  |  |  |  |  |  |  |  |  |  |  |  |  |  |  |  |  |  |  |  |  |  |  |  |  |  |  |  |  |  |  |  |  |  |  |  |  |  |  |  |  |  |  |  |  |  |  |  |  |  |  |  |  |  |  |  |  |  |  |  |  |  |  |  |  |  |  |  |  |  |  |  |  |  |  |  |  |  |  |  |  |  |  |  |  |  |  |  |  |  |  |  |  |  |  |  |  |  |  |  |  |  |  |  |  |  |  |  |  |  |  |  |  |  |  |  |  |  |  |  |  |  |  |  |  |  |  |  |  |  |  |  |  |  |  |  |  |  |  |  |  |  |  |  |  |  |  |  |  |  |  |  |  |  |  |  |  |  |  |  |  |  |  |  |  |  |  |  |  |  |  |  |  |  |  |  |  |  |  |  |  |  |  |  |  |  |  |  |  |  |  |  |  |  |  |  |  |  |  |  |  |  |  |  |  |  |  |  |  |  |  |  |  |  |  |  |  |  |  |  |  |  |  |  |  |  |  |  |  |  |  |  |  |  |  |  |  |  |  |  |  |  |  |  |  |  |  |  |  |  |  |  |  |  |  |  |  |  |  |  |  |  |  |  |  |  |  |  |  |  |  |  |  |  |  |  |  |  |  |  |  |  |  |  |  |  |  |  |  |  |  |  |  |  |  |  |  |  |  |  |  |  |  |  |  |  |  |  |  |  |  |  |  |  |  |  |  |  |  |  |  |  |  |  |  |  |  |  |  |  |  |  |  |  |  |  |  |  |  |  |  |  |  |  |  |  |  |  |  |  |  |  |  |  |  |  |  |  |  |  |  |  |  |  |  |  |  |  |  |  |  |  |  |  |  |  |  |  |  |  |  |  |  |  |  |  |  |  |  |  |  |  |  |  |  |  |  |  |  |  |  |  |  |  |  |  |  |  |  |  |  |  |  |  |  |  |  |  |  |  |  |  |  |  |  |  |  |  |  |  |  |  |  |  |  |  |  |  |  |  |  |  |  |  |  |  |  |  |  |  |  |  |  |  |  |  |  |  |  |  |  |  |  |  |  |  |  |  |  |  |  |  |  |  |  |  |  |  |  |  |  |  |  |  |  |  |  |  |  |  |  |  |  |  |  |  |  |  |  |  |  |  |  |  |  |  |  |  |  |  |  |  |  |  |  |  |  |  |  |  |  |  |  |  |  |  |  |  |  |  |  |  |  |  |  |  |  |  |  |  |  |  |  |  |  |  |  |  |  |  |  |  |  |  |  |  |  |  |  |  |  |  |  |  |  |  |  |  |  |  |  |  |  |  |  |  |  |  |  |  |  |  |  |  |  |  |  |  |  |  |  |  |  |  |  |  |  |  |  |  |  |  |  |  |  |  |  |  |  |  |  |  |  |  |  |  |  |  |  |  |  |  |  |  |  |  |  |  |  |  |  |  |  |  |  |  |  |  |  |  |  |  |  |  |  |  |  |  |  |  |  |  |  |  |  |  |  |  |  |  |  |  |  |  |  |  |  |  |  |  |  |  |  |  |  |  |  |  |  |  |  |  |  |  |  |  |  |  |  |  |  |  |  |  |  |  |  |  |  |  |  |  |  |  |  |  |  |  |  |  |  |  |  |  |  |  |  |  |  |  |  |  |  |  |  |  |  |  |  |  |  |  |  |  |  |  |  |  |  |  |  |  |  |  |  |  |  |  |  |  |  |  |  |  |  |  |  |  |  |  |  |  |  |  |  |  |  |  |  |  |  |  |  |  |  |  |  |  |  |  |  |  |  |  |  |  |  |  |  |  |  |  |  |  |  |  |  |  |  |  |  |  |  |  |  |  |  |  |  |  |  |  |  |  |  |  |  |  |  |  |  |  |  |  |  |  |  |  |  |  |  |  |  |  |  |  |  |  |  |  |  |  |  |  |  |  |  |  |  |  |  |  |  |  |  |  |  |  |  |  |  |  |  |  |  |  |  |  |  |  |  |  |  |  |  |  |  |  |  |  |  |  |  |  |  |  |  |  |  |  |  |  |  |  |  |  |  |  |  |  |  |  |  |  |  |  |  |  |  |  |  |  |  |  |  |  |  |  |  |  |  |  |  |  |  |  |  |  |  |  |  |  |  |  |  |  |  |  |  |  |  |  |  |  |  |  |  |  |  |  |  |  |  |  |  |  |  |  |  |  |  |  |  |  |  |  |  |  |  |  |  |  |  |  |  |  |  |  |  |  |  |  |  |  |  |  |  |  |  |  |  |  |  |  |  |  |  |  |  |  |  |  |  |  |  |  |  |  |  |  |  |  |  |  |  |  |  |  |  |  |  |  |  |  |  |  |  |  |  |  |  |  |  |  |  |  |  |  |  |  |  |  |  |  |  |  |  |  |  |  |  |  |  |  |  |  |  |  |  |  |  |  |  |  |  |  |  |  |  |  |  |  |  |  |  |  |  |  |  |  |  |  |  |  |  |  |  |  |  |  |  |  |  |  |  |  |  |  |  |  |  |  |  |  |  |  |  |  |  |  |  |  |  |  |  |  |  |  |  |  |  |  |  |  |  |  |  |  |  |  |  |  |  |  |  |  |  |  |  |  |  |  |  |  |  |  |  |  |  |  |  |  |  |  |  |  |  |  |  |  |  |  |  |  |  |  |  |  |  |  |  |  |  |  |  |  |  |  |  |  |  |  |  |  |  |  |  |  |  |  |  |  |  |  |  |  |  |  |  |  |  |  |  |  |  |  |  |  |  |  |  |  |  |  |  |  |  |  |  |  |  |  |  |  |  |  |  |  |  |  |  |  |  |  |  |  |  |  |  |  |  |  |  |  |  |  |  |  |  |  |  |  |  |  |  |  |  |  |  |  |  |  |  |  |  |  |  |  |  |  |  |  |  |  |  |  |  |  |  |  |  |  |  |  |  |  |  |  |  |  |  |  |  |  |  |  |  |  |  |  |  |  |  |  |  |  |  |  |  |  |  |  |  |  |  |  |  |  |  |  |  |  |  |  |  |  |  |  |  |  |  |  |  |  |  |  |  |  |  |  |  |  |  |  |  |  |  |  |  |  |  |  |  |  |  |  |  |  |  |  |  |  |  |  |  |  |  |  |  |  |  |  |  |  |  |  |  |  |  |  |  |  |  |  |  |  |  |  |  |  |  |  |  |  |  |  |  |  |  |  |  |  |  |  |  |  |  |  |  |  |  |  |  |  |  |  |  |  |  |  |  |  |  |  |  |  |  |  |  |  |  |  |  |  |  |  |  |  |  |  |  |  |  |  |  |  |  |  |  |  |  |  |  |  |  |  |  |  |  |  |  |  |  |  |  |  |  |  |  |  |  |  |  |  |  |  |  |  |  |  |  |  |  |  |  |  |  |  |  |  |  |  |  |  |  |  |  |  |  |  |  |  |  |  |  |  |  |  |  |  |  |  |  |  |  |  |  |  |  |  |  |  |  |  |  |  |  |  |  |  |  |  |  |  |  |  |  |  |  |  |  |  |  |  |  |  |  |  |  |  |  |  |  |  |  |  |  |  |  |  |  |  |  |  |  |  |  |  |  |  |  |  |  |  |  |  |  |  |  |  |  |  |  |  |  |  |  |  |  |  |  |  |  |  |  |  |  |  |  |  |  |  |  |  |  |  |  |  |  |  |  |  |  |  |  |  |  |  |  |  |  |  |  |  |  |  |  |  |  |  |  |  |  |  |  |  |  |  |  |  |  |  |  |  |  |  |  |  |  |  |  |  |  |  |  |  |  |  |  |  |  |  |  |  |  |  |  |  |  |  |  |  |  |  |  |  |  |  |  |  |  |  |  |  |  |  |  |  |  |  |  |  |  |  |  |  |  |  |  |  |  |  |  |  |  |  |  |  |  |  |  |  |  |  |  |  |  |  |  |  |  |  |  |  |  |  |  |  |  |  |  |  |  |  |  |  |  |  |  |  |  |  |  |  |  |  |  |  |  |  |  |  |  |  |  |  |  |  |  |  |  |  |  |  |  |  |  |  |  |  |  |  |  |  |  |  |  |  |  |  |  |  |  |  |  |  |  |  |  |  |  |  |  |  |  |  |  |  |  |  |  |  |  |  |  |  |  |  |  |  |  |  |  |  |  |  |  |  |  |  |  |  |  |  |  |  |  |  |  |  |  |  |  |  |  |  |  |  |  |  |  |  |  |  |  |  |  |  |  |  |  |  |  |  |  |  |  |  |  |  |  |  |  |  |  |  |  |  |  |  |  |  |  |  |  |  |  |  |  |  |  |  |  |  |  |  |  |  |  |  |  |  |  |  |  |  |  |  |  |  |  |  |  |  |  |  |  |  |  |  |  |  |  |  |  |  |  |  |  |  |  |  |  |  |  |  |  |  |  |  |  |  |  |  |  |  |  |  |  |  |  |  |  |  |  |  |  |  |  |  |  |  |  |  |  |  |  |  |  |  |  |  |  |  |  |  |  |  |  |  |  |  |  |  |  |  |  |  |  |  |  |  |  |  |  |  |  |  |  |  |  |  |  |  |  |  |  |  |  |  |  |  |  |  |  |  |  |  |  |  |  |  |  |  |  |  |  |  |  |  |  |  |  |  |  |  |  |  |  |  |  |  |  |  |  |  |  |  |  |  |  |  |  |  |  |  |  |  |  |  |  |  |  |  |  |  |  |  |  |  |  |  |  |  |  |  |  |  |  |  |  |  |  |  |  |  |  |  |  |  |  |  |  |  |  |  |  |  |  |  |  |  |  |  |  |  |  |  |  |  |  |  |  |  |  |  |  |  |  |  |  |  |  |  |  |  |  |  |  |  |  |  |  |  |  |  |  |  |  |  |  |  |  |  |  |  |  |  |  |  |  |  |  |  |  |  |  |  |  |  |  |  |  |  |  |  |  |  |  |  |  |  |  |  |  |  |  |  |  |  |  |  |  |  |  |  |  |  |  |  |  |  |  |  |  |  |  |  |  |  |  |  |  |  |  |  |  |  |  |  |  |  |  |  |  |  |  |  |  |  |  |  |  |  |  |  |  |  |  |  |  |  |  |  |  |  |  |  |  |  |  |  |  |  |  |  |  |  |  |  |  |  |  |  |  |  |  |  |  |  |  |  |  |  |  |  |  |  |  |  |  |  |  |  |  |  |  |  |  |  |  |  |  |  |  |  |  |  |  |  |  |  |  |  |  |  |  |  |  |  |  |  |  |  |  |  |  |  |  |  |  |  |  |  |  |  |  |  |  |  |  |  |  |  |  |  |  |  |  |  |  |  |  |  |  |  |  |  |  |  |  |  |  |  |  |  |  |  |  |  |  |  |  |  |  |  |  |  |  |  |  |  |  |  |  |  |  |  |  |  |  |  |  |  |  |  |  |  |  |  |  |  |  |  |  |  |  |  |  |  |  |  |  |  |  |  |  |  |  |  |  |  |  |  |  |  |  |  |  |  |  |  |  |  |  |  |  |  |  |  |  |  |  |  |  |  |  |  |  |  |  |  |  |  |  |  |  |  |  |  |  |  |  |  |  |  |  |  |  |  |  |  |  |  |  |  |  |  |  |  |  |  |  |  |  |  |  |  |  |  |  |  |  |  |  |  |  |  |  |  |  |  |  |  |  |  |  |  |  |  |  |  |  |  |  |  |  |  |  |  |  |  |  |  |  |  |  |  |  |  |  |  |  |  |  |  |  |  |  |  |  |  |  |  |  |  |  |  |  |  |  |  |  |  |  |  |  |  |  |  |  |  |  |  |  |  |  |  |  |  |  |  |  |  |  |  |  |  |  |  |  |  |  |  |  |  |  |  |  |  |  |  |  |  |  |  |  |  |  |  |  |  |  |  |  |  |  |  |  |  |  |  |  |  |  |  |  |  |  |  |  |  |  |  |  |  |  |  |  |  |  |  |  |  |  |  |  |  |  |  |  |  |  |  |  |  |  |  |  |  |  |  |  |  |  |  |  |  |  |  |  |  |  |  |  |  |  |  |  |  |  |  |  |  |  |  |  |  |  |  |  |  |  |  |  |  |  |  |  |  |  |  |  |  |  |  |  |  |  |  |  |  |  |  |  |  |  |  |  |  |  |  |  |  |  |  |  |  |  |  |  |  |  |  |  |  |  |  |  |  |  |  |  |  |  |  |  |  |  |  |  |  |  |  |  |  |  |  |  |  |  |  |  |  |  |  |  |  |  |  |  |  |  |  |  |  |  |  |  |  |  |  |  |  |  |  |  |  |  |  |  |  |  |  |  |  |  |  |  |  |  |  |  |  |  |  |  |  |  |  |  |  |  |  |  |  |  |  |  |  |  |  |  |  |  |  |  |  |  |  |  |  |  |  |  |  |  |  |  |  |  |  |  |  |  |  |  |  |  |  |  |  |  |  |  |  |  |  |  |  |  |  |  |  |  |  |  |  |  |  |  |  |  |  |  |  |  |  |  |  |  |  |  |  |  |  |  |  |  |  |  |  |  |  |  |  |  |  |  |  |  |  |  |  |  |  |  |  |  |  |  |  |  |  |  |  |  |  |  |  |  |  |  |  |  |  |  |  |  |  |  |  |  |  |  |  |  |  |  |  |  |  |  |  |  |  |  |  |  |  |  |  |  |  |  |  |  |  |  |  |  |  |  |  |  |  |  |  |  |  |  |  |  |  |  |  |  |  |  |  |  |  |  |  |  |  |  |  |  |  |  |  |  |  |  |  |  |  |  |  |  |  |  |  |  |  |  |  |  |  |  |  |  |  |  |  |  |  |  |  |  |  |  |  |  |  |  |  |  |  |  |  |  |  |  |  |  |  |  |  |  |  |  |  |  |  |  |  |  |  |  |  |  |  |  |  |  |  |  |  |  |  |  |  |  |  |  |  |  |  |  |  |  |  |  |  |  |  |  |  |  |  |  |  |  |  |  |  |  |  |  |  |  |  |  |  |  |  |  |  |  |  |  |  |  |  |  |  |  |  |  |  |  |  |  |  |  |  |  |  |  |  |  |  |  |  |  |  |  |  |  |  |  |  |  |  |  |  |  |  |  |  |  |  |  |  |  |  |  |  |  |  |  |  |  |  |  |  |  |  |  |  |  |  |  |  |  |  |  |  |  |  |  |  |  |  |  |  |  |  |  |  |  |  |  |  |  |  |  |  |  |  |  |  |  |  |  |  |  |  |  |  |  |  |  |  |  |  |  |  |  |  |  |  |  |  |  |  |  |  |  |  |  |  |  |  |  |  |  |  |  |  |  |  |  |  |  |  |  |  |  |  |  |  |  |  |  |  |  |  |  |  |  |  |  |  |  |  |  |  |  |  |  |  |  |  |  |  |  |  |  |  |  |  |  |  |  |  |  |  |  |  |  |  |  |  |  |  |  |  |  |  |  |  |  |  |  |  |  |  |  |  |  |  |  |  |  |  |  |  |  |  |  |  |  |  |  |  |  |  |  |  |  |  |  |  |  |  |  |  |  |  |  |  |  |  |  |  |  |  |  |  |  |  |  |  |  |  |  |  |  |  |  |  |  |  |  |  |  |  |  |  |  |  |  |  |  |  |  |  |  |  |  |  |  |  |  |  |  |  |  |  |  |  |  |  |  |  |  |  |  |  |  |  |  |  |  |  |  |  |  |  |  |  |  |  |  |  |  |  |  |  |  |  |  |  |  |  |  |  |  |  |  |  |  |  |  |  |  |  |  |  |  |  |  |  |  |  |  |  |  |  |  |  |  |  |  |  |  |  |  |  |  |  |  |  |  |  |  |  |  |  |  |  |  |  |  |  |  |  |  |  |  |  |  |  |  |  |  |  |  |  |  |  |  |  |  |  |  |  |  |  |  |  |  |  |  |  |  |  |  |  |  |  |  |  |  |  |  |  |  |  |  |  |  |  |  |  |  |  |  |  |  |  |  |  |  |  |  |  |  |  |  |  |  |  |  |  |  |  |  |  |  |  |  |  |  |  |  |  |  |  |  |  |  |  |  |  |  |  |  |  |  |  |  |  |  |  |  |  |  |  |  |  |  |  |  |  |  |  |  |  |  |  |  |  |  |  |  |  |  |  |  |  |  |  |  |  |  |  |  |  |  |  |  |  |  |  |  |  |  |  |  |  |  |  |  |  |  |  |  |  |  |  |  |  |  |  |  |  |  |  |  |  |  |  |  |  |  |  |  |  |  |  |  |  |  |  |  |  |  |  |  |  |  |  |  |  |  |  |  |  |  |  |  |  |  |  |  |  |  |  |  |  |  |  |  |  |  |  |  |  |  |  |  |  |  |  |  |  |  |  |  |  |  |  |  |  |  |  |  |  |  |  |  |  |  |  |  |  |  |  |  |  |  |  |  |  |  |  |  |  |  |  |  |  |  |  |  |  |  |  |  |  |  |  |  |  |  |  |  |  |  |  |  |  |  |  |  |  |  |  |  |  |  |  |  |  |  |  |  |  |  |  |  |  |  |  |  |  |  |  |  |  |  |  |  |  |  |  |  |  |  |  |  |  |  |  |  |  |  |  |  |  |  |  |  |  |  |  |  |  |  |  |  |  |  |  |  |  |  |  |  |  |  |  |  |  |  |  |  |  |  |  |  |  |  |  |  |  |  |  |  |  |  |  |  |  |  |  |  |  |  |  |  |  |  |  |  |  |  |  |  |  |  |  |  |  |  |  |  |  |  |  |  |  |  |  |  |  |  |  |  |  |  |  |  |  |  |  |  |  |  |  |  |  |  |  |  |  |  |  |  |  |  |  |  |  |  |  |  |  |  |  |  |  |  |  |  |  |  |  |  |  |  |  |  |  |  |  |  |  |  |  |  |  |  |  |  |  |  |  |  |  |  |  |  |  |  |  |  |  |  |  |  |  |  |  |  |  |  |  |  |  |  |  |  |  |  |  |  |  |  |  |  |  |  |  |  |  |  |  |  |  |  |  |  |  |  |  |  |  |  |  |  |  |  |  |  |  |  |  |  |  |  |  |  |  |  |  |  |  |  |  |  |  |  |  |  |  |  |  |  |  |  |  |  |  |  |  |  |  |  |  |  |  |  |  |  |  |  |  |  |  |  |  |  |  |  |  |  |  |  |  |  |  |  |  |  |  |  |  |  |  |  |  |  |  |  |  |  |  |  |  |  |  |  |  |  |  |  |  |  |  |  |  |  |  |  |  |  |  |  |  |  |  |  |  |  |  |  |  |  |  |  |  |  |  |  |  |  |  |  |  |  |  |  |  |  |  |  |  |  |  |  |  |  |  |  |  |  |  |  |  |  |  |  |  |  |  |  |  |  |  |  |  |  |  |  |  |  |  |  |  |  |  |  |  |  |  |  |  |  |  |  |  |  |  |  |  |  |  |  |  |  |  |  |  |  |  |  |  |  |  |  |  |  |  |  |  |  |  |  |  |  |  |  |  |  |  |  |  |  |  |  |  |  |  |  |  |  |  |  |  |  |  |  |  |  |  |  |  |  |  |  |  |  |  |  |  |  |  |  |  |  |  |  |  |  |  |  |  |  |  |  |  |  |  |  |  |  |  |  |  |  |  |  |  |  |  |  |  |  |  |  |  |  |  |  |  |  |  |  |  |  |  |  |  |  |  |  |  |  |  |  |  |  |  |  |  |  |  |  |  |  |  |  |  |  |  |  |  |  |  |  |  |  |  |  |  |  |  |  |  |  |  |  |  |  |  |  |  |  |  |  |  |  |  |  |  |  |  |  |  |  |  |  |  |  |  |  |  |  |  |  |  |  |  |  |  |  |  |  |  |  |  |  |  |  |  |  |  |  |  |  |  |  |  |  |  |  |  |  |  |  |  |  |  |  |  |  |  |  |  |  |  |  |  |  |  |  |  |  |  |  |  |  |  |  |  |  |  |  |  |  |  |  |  |  |  |  |  |  |  |  |  |  |  |  |  |  |  |  |  |  |  |  |  |  |  |  |  |  |  |  |  |  |  |  |  |  |  |  |  |  |  |  |  |  |  |  |  |  |  |  |  |  |  |  |  |  |  |  |  |  |  |  |  |  |  |  |  |  |  |  |  |  |  |  |  |  |  |  |  |  |  |  |  |  |  |  |  |  |  |  |  |  |  |  |  |  |  |  |  |  |  |  |  |  |  |  |  |  |  |  |  |  |  |  |  |  |  |  |  |  |  |  |  |  |  |  |  |  |  |  |  |  |  |  |  |  |  |  |  |  |  |  |  |  |  |  |  |  |  |  |  |  |  |  |  |  |  |  |  |  |  |  |  |  |  |  |  |  |  |  |  |  |  |  |  |  |  |  |  |  |  |  |  |  |  |  |  |  |  |  |  |  |  |  |  |  |  |  |  |  |  |  |  |  |  |  |  |  |  |  |  |  |  |  |  |  |  |  |  |  |  |  |  |  |  |  |  |  |  |  |  |  |  |  |  |  |  |  |  |  |  |  |  |  |  |  |  |  |  |  |  |  |  |  |  |  |  |  |  |  |  |  |  |  |  |  |  |  |  |  |  |  |  |  |  |  |  |  |  |  |  |  |  |  |  |  |  |  |  |  |  |  |  |  |  |  |  |  |  |  |  |  |  |  |  |  |  |  |  |  |  |  |  |  |  |  |  |  |  |  |  |  |  |  |  |  |  |  |  |  |  |  |  |  |  |  |  |  |  |  |  |  |  |  |  |  |  |  |  |  |  |  |  |  |  |  |  |  |  |  |  |  |  |  |  |  |  |  |  |  |  |  |  |  |  |  |  |  |  |  |  |  |  |  |  |  |  |  |  |  |  |  |  |  |  |  |  |  |  |  |  |  |  |  |  |  |  |  |  |  |  |  |  |  |  |  |  |  |  |  |  |  |  |  |  |  |  |  |  |  |  |  |  |  |  |  |  |  |  |  |  |  |  |  |  |  |  |  |  |  |  |  |  |  |  |  |  |  |  |  |  |  |  |  |  |  |  |  |  |  |  |  |  |  |  |  |  |  |  |  |  |  |  |  |  |  |  |  |  |  |  |  |  |  |  |  |  |  |  |  |  |  |  |  |  |  |  |  |  |  |  |  |  |  |  |  |  |  |  |  |  |  |  |  |  |  |  |  |  |  |  |  |  |  |  |  |  |  |  |  |  |  |  |  |  |  |  |  |  |  |  |  |  |  |  |  |  |  |  |  |  |  |  |  |  |  |  |  |  |  |  |  |  |  |  |  |  |  |  |  |  |  |  |  |  |  |  |  |  |  |  |  |  |  |  |  |  |  |  |  |  |  |  |  |  |  |  |  |  |  |  |  |  |  |  |  |  |  |  |  |  |  |  |  |  |  |  |  |  |  |  |  |  |  |  |  |  |  |  |  |  |  |  |  |  |  |  |  |  |  |  |  |  |  |  |  |  |  |  |  |  |  |  |  |  |  |  |  |  |  |  |  |  |  |  |  |  |  |  |  |  |  |  |  |  |  |  |  |  |  |  |  |  |  |  |  |  |  |  |  |  |  |  |  |  |  |  |  |  |  |  |  |  |  |  |  |  |  |  |  |  |  |  |  |  |  |  |  |  |  |  |  |  |  |  |  |  |  |  |  |  |  |  |  |  |  |  |  |  |  |  |  |  |  |  |  |  |  |  |  |  |  |  |  |  |  |  |  |  |  |  |  |  |  |  |  |  |  |  |  |  |  |  |  |  |  |  |  |  |  |  |  |  |  |  |  |  |  |  |  |  |  |  |  |  |  |  |  |  |  |  |  |  |  |  |  |  |  |  |  |  |  |  |  |  |  |  |  |  |  |  |  |  |  |  |  |  |  |  |  |  |  |  |  |  |  |  |  |  |  |  |  |  |  |  |  |  |  |  |  |  |  |  |  |  |  |  |  |  |  |  |  |  |  |  |  |  |  |  |  |  |  |  |  |  |  |  |  |  |  |  |  |  |  |  |  |  |  |  |  |  |  |  |  |  |  |  |  |  |  |  |  |  |  |  |  |  |  |  |  |  |  |  |  |  |  |  |  |  |  |  |  |  |  |  |  |  |  |  |  |  |  |  |  |  |  |  |  |  |  |  |  |  |  |  |  |  |  |  |  |  |  |  |  |  |  |  |  |  |  |  |  |  |  |  |  |  |  |  |  |  |  |  |  |  |  |  |  |  |  |  |  |  |  |  |  |  |  |  |  |  |  |  |  |  |  |  |  |  |  |  |  |  |  |  |  |  |  |  |  |  |  |  |  |  |  |  |  |  |  |  |  |  |  |  |  |  |  |  |  |  |  |  |  |  |  |  |  |  |  |  |  |  |  |  |  |  |  |  |  |  |  |  |  |  |  |  |  |  |  |  |  |  |  |  |  |  |  |  |  |  |  |  |  |  |  |  |  |  |  |  |  |  |  |  |  |  |  |  |  |  |  |  |  |  |  |  |  |  |  |  |  |  |  |  |  |  |  |  |  |  |  |  |  |  |  |  |  |  |  |  |  |  |  |  |  |  |  |  |  |  |  |  |  |  |  |  |  |  |  |  |  |  |  |  |  |  |  |  |  |  |  |  |  |  |  |  |  |  |  |  |  |  |  |  |  |  |  |  |  |  |  |  |  |  |  |  |  |  |  |  |  |  |  |  |  |  |  |  |  |  |  |  |  |  |  |  |  |  |  |  |  |  |  |  |  |  |  |  |  |  |  |  |  |  |  |  |  |  |  |  |  |  |  |  |  |  |  |  |  |  |  |  |  |  |  |  |  |  |  |  |  |  |  |  |  |  |  |  |  |  |  |  |  |  |  |  |  |  |  |  |  |  |  |  |  |  |  |  |  |  |  |  |  |  |  |  |  |  |  |  |  |  |  |  |  |  |  |  |  |  |  |  |  |  |  |  |  |  |  |  |  |  |  |  |  |  |  |  |  |  |  |  |  |  |  |  |  |  |  |  |  |  |  |  |  |  |  |  |  |  |  |  |  |  |  |  |  |  |  |  |  |  |  |  |  |  |  |  |  |  |  |  |  |  |  |  |  |  |  |  |  |  |  |  |  |  |  |  |  |  |  |  |  |  |  |  |  |  |  |  |  |  |  |  |  |  |  |  |  |  |  |  |  |  |  |  |  |  |  |  |  |  |  |  |  |  |  |  |  |  |  |  |  |  |  |  |  |  |  |  |  |  |  |  |  |  |  |  |  |  |  |  |  |  |  |  |  |  |  |  |  |  |  |  |  |  |  |  |  |  |  |  |  |  |  |  |  |  |  |  |  |  |  |  |  |  |  |  |  |  |  |  |  |  |  |  |  |  |  |  |  |  |  |  |  |  |  |  |  |  |  |  |  |  |  |  |  |  |  |  |  |  |  |  |  |  |  |  |  |  |  |  |  |  |  |  |  |  |  |  |  |  |  |  |  |  |  |  |  |  |  |  |  |  |  |  |  |  |  |  |  |  |  |  |  |  |  |  |  |  |  |  |  |  |  |  |  |  |  |  |  |  |  |  |  |  |  |  |  |  |  |  |  |  |  |  |  |  |  |  |  |  |  |  |  |  |  |  |  |  |  |  |  |  |  |  |  |  |  |  |  |  |  |  |  |  |  |  |  |  |  |  |  |  |  |  |  |  |  |  |  |  |  |  |  |  |  |  |  |  |  |  |  |  |  |  |  |  |  |  |  |  |  |  |  |  |  |  |  |  |  |  |  |  |  |  |  |  |  |  |  |  |  |  |  |  |  |  |  |  |  |  |  |  |  |  |  |  |  |  |  |  |  |  |  |  |  |  |  |  |  |  |  |  |  |  |  |  |  |  |  |  |  |  |  |  |  |  |  |  |  |  |  |  |  |  |  |  |  |  |  |  |  |  |  |  |  |  |  |  |  |  |  |  |  |  |  |  |  |  |  |  |  |  |  |  |  |  |  |  |  |  |  |  |  |  |  |  |  |  |  |  |  |  |  |  |  |  |  |  |  |  |  |  |  |  |  |  |  |  |  |  |  |  |  |  |  |  |  |  |  |  |  |  |  |  |  |  |  |  |  |  |  |  |  |  |  |  |  |  |  |  |  |  |  |  |  |  |  |  |  |  |  |  |  |  |  |  |  |  |  |  |  |  |  |  |  |  |  |  |  |  |  |  |  |  |  |  |  |  |  |  |  |  |  |  |  |  |  |  |  |  |  |  |  |  |  |  |  |  |  |  |  |  |  |  |  |  |  |  |  |  |  |  |  |  |  |  |  |  |  |  |  |  |  |  |  |  |  |  |  |  |  |  |  |  |  |  |  |  |  |  |  |  |  |  |  |  |  |  |  |  |  |  |  |  |  |  |  |  |  |  |  |  |  |  |  |  |  |  |  |  |  |  |  |  |  |  |  |  |  |  |  |  |  |  |  |  |  |  |  |  |  |  |  |  |  |  |  |  |  |  |  |  |  |  |  |  |  |  |  |  |  |  |  |  |  |  |  |  |  |  |  |  |  |  |  |  |  |  |  |  |  |  |  |  |  |  |  |  |  |  |  |  |  |  |  |  |  |  |  |  |  |  |  |  |  |  |  |  |  |  |  |  |  |  |  |  |  |  |  |  |  |  |  |  |  |  |  |  |  |  |  |  |  |  |  |  |  |  |  |  |  |  |  |  |  |  |  |  |  |  |  |  |  |  |  |  |  |  |  |  |  |  |  |  |  |  |  |  |  |  |  |  |  |  |  |  |  |  |  |  |  |  |  |  |  |  |  |  |  |  |  |  |  |  |  |  |  |  |  |  |  |  |  |  |  |  |  |  |  |  |  |  |  |  |  |  |  |  |  |  |  |  |  |  |  |  |  |  |  |  |  |  |  |  |  |  |  |  |  |  |  |  |  |  |  |  |  |  |  |  |  |  |  |  |  |  |  |  |  |  |  |  |  |  |  |  |  |  |  |  |  |  |  |  |  |  |  |  |  |  |  |  |  |  |  |  |  |  |  |  |  |  |  |  |  |  |  |  |  |  |  |  |  |  |  |  |  |  |  |  |  |  |  |  |  |  |  |  |  |  |  |  |  |  |  |  |  |  |  |  |  |  |  |  |  |  |  |  |  |  |  |  |  |  |  |  |  |  |  |  |  |  |  |  |  |  |  |  |  |  |  |  |  |  |  |  |  |  |  |  |  |  |  |  |  |  |  |  |  |  |  |  |  |  |  |  |  |  |  |  |  |  |  |  |  |  |  |  |  |  |  |  |  |  |  |  |  |  |  |  |  |  |  |  |  |  |  |  |  |  |  |  |  |  |  |  |  |  |  |  |  |  |  |  |  |  |  |  |  |  |  |  |  |  |  |  |  |  |  |  |  |  |  |  |  |  |  |  |  |  |  |  |  |  |  |  |  |  |  |  |  |  |  |  |  |  |  |  |  |  |  |  |  |  |  |  |  |  |  |  |  |  |  |  |  |  |  |  |  |  |  |  |  |  |  |  |  |  |  |  |  |  |  |  |  |  |  |  |  |  |  |  |  |  |  |  |  |  |  |  |  |  |  |  |  |  |  |  |  |  |  |  |  |  |  |  |  |  |  |  |  |  |  |  |  |  |  |  |  |  |  |  |  |  |  |  |  |  |  |  |  |  |  |  |  |  |  |  |  |  |  |  |  |  |  |  |  |  |  |  |  |  |  |  |  |  |  |  |  |  |  |  |  |  |  |  |  |  |  |  |  |  |  |  |  |  |  |  |  |  |  |  |  |  |  |  |  |  |  |  |  |  |  |  |  |  |  |  |  |  |  |  |  |  |  |  |  |  |  |  |  |  |  |  |  |  |  |  |  |  |  |  |  |  |  |  |  |  |  |  |  |  |  |  |  |  |  |  |  |  |  |  |  |  |  |  |  |  |  |  |  |  |  |  |  |  |  |  |  |  |  |  |  |  |  |  |  |  |  |  |  |  |  |  |  |  |  |  |  |  |  |  |  |  |  |  |  |  |  |  |  |  |  |  |  |  |  |  |  |  |  |  |  |  |  |  |  |  |  |  |  |  |  |  |  |  |  |  |  |  |  |  |  |  |  |  |  |  |  |  |  |  |  |  |  |  |  |  |  |  |  |  |  |  |  |  |  |  |  |  |  |  |  |  |  |  |  |  |  |  |  |  |  |  |  |  |  |  |  |  |  |  |  |  |  |  |  |  |  |  |  |  |  |  |  |  |  |  |  |  |  |  |  |  |  |  |  |  |  |  |  |  |  |  |  |  |  |  |  |  |  |  |  |  |  |  |  |  |  |  |  |  |  |  |  |  |  |  |  |  |  |  |  |  |  |  |  |  |  |  |  |  |  |  |  |  |  |  |  |  |  |  |  |  |  |  |  |  |  |  |  |  |  |  |  |  |  |  |  |  |  |  |  |  |  |  |  |  |  |  |  |  |  |  |  |  |  |  |  |  |  |  |  |  |  |  |  |  |  |  |  |  |  |  |  |  |  |  |  |  |  |  |  |  |  |  |  |  |  |  |  |  |  |  |  |  |  |  |  |  |  |  |  |  |  |  |  |  |  |  |  |  |  |  |  |  |  |  |  |  |  |  |  |  |  |  |  |  |  |  |  |  |  |  |  |  |  |  |  |  |  |  |  |  |  |  |  |  |  |  |  |  |  |  |  |  |  |  |  |  |  |  |  |  |  |  |  |  |  |  |  |  |  |  |  |  |  |  |  |  |  |  |  |  |  |  |  |  |  |  |  |  |  |  |  |  |  |  |  |  |  |  |  |  |  |  |  |  |  |  |  |  |  |  |  |  |  |  |  |  |  |  |  |  |  |  |  |  |  |  |  |  |  |  |  |  |  |  |  |  |  |  |  |  |  |  |  |  |  |  |  |  |  |  |  |  |  |  |  |  |  |  |  |  |  |  |  |  |  |  |  |  |  |  |  |  |  |  |  |  |  |  |  |  |  |  |  |  |  |  |  |  |  |  |  |  |  |  |  |  |  |  |  |  |  |  |  |  |  |  |  |  |  |  |  |  |  |  |  |  |  |  |  |  |  |  |  |  |  |  |  |  |  |  |  |  |  |  |  |  |  |  |  |  |  |  |  |  |  |  |  |  |  |  |  |  |  |  |  |  |  |  |  |  |  |  |  |  |  |  |  |  |  |  |  |  |  |  |  |  |  |  |  |  |  |  |  |  |  |  |  |  |  |  |  |  |  |  |  |  |  |  |  |  |  |  |  |  |  |  |  |  |  |  |  |  |  |  |  |  |  |  |  |  |  |  |  |  |  |  |  |  |  |  |  |  |  |  |  |  |  |  |  |  |  |  |  |  |  |  |  |  |  |  |  |  |  |  |  |  |  |  |  |  |  |  |  |  |  |  |  |  |  |  |  |  |  |  |  |  |  |  |  |  |  |  |  |  |  |  |  |  |  |  |  |  |  |  |  |  |  |  |  |  |  |  |  |  |  |  |  |  |  |  |  |  |  |  |  |  |  |  |  |  |  |  |  |  |  |  |  |  |  |  |  |  |  |  |  |  |  |  |  |  |  |  |  |  |  |  |  |  |  |  |  |  |  |  |  |  |  |  |  |  |  |  |  |  |  |  |  |  |  |  |  |  |  |  |  |  |  |  |  |  |  |  |  |  |  |  |  |  |  |  |  |  |  |  |  |  |  |  |  |  |  |  |  |  |  |  |  |  |  |  |  |  |  |  |  |  |  |  |  |  |  |  |  |  |  |  |  |  |  |  |  |  |  |  |  |  |  |  |  |  |  |  |  |  |  |  |  |  |  |  |  |  |  |  |  |  |  |  |  |  |  |  |  |  |  |  |  |  |  |  |  |  |  |  |  |  |  |  |  |  |  |  |  |  |  |  |  |  |  |  |  |  |  |  |  |  |  |  |  |  |  |  |  |  |  |  |  |  |  |  |  |  |  |  |  |  |  |  |  |  |  |  |  |  |  |  |  |  |  |  |  |  |  |  |  |  |  |  |  |  |  |  |  |  |  |  |  |  |  |  |  |  |  |  |  |  |  |  |  |  |  |  |  |  |  |  |  |  |  |  |  |  |  |  |  |  |  |  |  |  |  |  |  |  |  |  |  |  |  |  |  |  |  |  |  |  |  |  |  |  |  |  |  |  |  |  |  |  |  |  |  |  |  |  |  |  |  |  |  |  |  |  |  |  |  |  |  |  |  |  |  |  |  |  |  |  |  |  |  |  |  |  |  |  |  |  |  |  |  |  |  |  |  |  |  |  |  |  |  |  |  |  |  |  |  |  |  |  |  |  |  |  |  |  |  |  |  |  |  |  |  |  |  |  |  |  |  |  |  |  |  |  |  |  |  |  |  |  |  |  |  |  |  |  |  |  |  |  |  |  |  |  |  |  |  |  |  |  |  |  |  |  |  |  |  |  |  |  |  |  |  |  |  |  |  |  |  |  |  |  |  |  |  |  |  |  |  |  |  |  |  |  |  |  |  |  |  |  |  |  |  |  |  |  |  |  |  |  |  |  |  |  |  |  |  |  |  |  |  |  |  |  |  |  |  |  |  |  |  |  |  |  |  |  |  |  |  |  |  |  |  | | --- | --- | --- | --- | --- | --- | --- | --- | --- | --- | --- | --- | --- | --- | --- | --- | --- | --- | --- | --- | --- | --- | --- | --- | --- | --- | --- | --- | --- | --- | --- | --- | --- | --- | --- | --- | --- | --- | --- | --- | --- | --- | --- | --- | --- | --- | --- | --- | --- | --- | --- | --- | --- | --- | --- | --- | --- | --- | --- | --- | --- | --- | --- | --- | --- | --- | --- | --- | --- | --- | --- | --- | --- | --- | --- | --- | --- | --- | --- | --- | --- | --- | --- | --- | --- | --- | --- | --- | --- | --- | --- | --- | --- | --- | --- | --- | --- | --- | --- | --- | --- | --- | --- | --- | --- | --- | --- | --- | --- | --- | --- | --- | --- | --- | --- | --- | --- | --- | --- | --- | --- | --- | --- | --- | --- | --- | --- | --- | --- | --- | --- | --- | --- | --- | --- | --- | --- | --- | --- | --- | --- | --- | --- | --- | --- | --- | --- | --- | --- | --- | --- | --- | --- | --- | --- | --- | --- | --- | --- | --- | --- | --- | --- | --- | --- | --- | --- | --- | --- | --- | --- | --- | --- | --- | --- | --- | --- | --- | --- | --- | --- | --- | --- | --- | --- | --- | --- | --- | --- | --- | --- | --- | --- | --- | --- | --- | --- | --- | --- | --- | --- | --- | --- | --- | --- | --- | --- | --- | --- | --- | --- | --- | --- | --- | --- | --- | --- | --- | --- | --- | --- | --- | --- | --- | --- | --- | --- | --- | --- | --- | --- | --- | --- | --- | --- | --- | --- | --- | --- | --- | --- | --- | --- | --- | --- | --- | --- | --- | --- | --- | --- | --- | --- | --- | --- | --- | --- | --- | --- | --- | --- | --- | --- | --- | --- | --- | --- | --- | --- | --- | --- | --- | --- | --- | --- | --- | --- | --- | --- | --- | --- | --- | --- | --- | --- | --- | --- | --- | --- | --- | --- | --- | --- | --- | --- | --- | --- | --- | --- | --- | --- | --- | --- | --- | --- | --- | --- | --- | --- | --- | --- | --- | --- | --- | --- | --- | --- | --- | --- | --- | --- | --- | --- | --- | --- | --- | --- | --- | --- | --- | --- | --- | --- | --- | --- | --- | --- | --- | --- | --- | --- | --- | --- | --- | --- | --- | --- | --- | --- | --- | --- | --- | --- | --- | --- | --- | --- | --- | --- | --- | --- | --- | --- | --- | --- | --- | --- | --- | --- | --- | --- | --- | --- | --- | --- | --- | --- | --- | --- | --- | --- | --- | --- | --- | --- | --- | --- | --- | --- | --- | --- | --- | --- | --- | --- | --- | --- | --- | --- | --- | --- | --- | --- | --- | --- | --- | --- | --- | --- | --- | --- | --- | --- | --- | --- | --- | --- | --- | --- | --- | --- | --- | --- | --- | --- | --- | --- | --- | --- | --- | --- | --- | --- | --- | --- | --- | --- | --- | --- | --- | --- | --- | --- | --- | --- | --- | --- | --- | --- | --- | --- | --- | --- | --- | --- | --- | --- | --- | --- | --- | --- | --- | --- | --- | --- | --- | --- | --- | --- | --- | --- | --- | --- | --- | --- | --- | --- | --- | --- | --- | --- | --- | --- | --- | --- | --- | --- | --- | --- | --- | --- | --- | --- | --- | --- | --- | --- | --- | --- | --- | --- | --- | --- | --- | --- | --- | --- | --- | --- | --- | --- | --- | --- | --- | --- | --- | --- | --- | --- | --- | --- | --- | --- | --- | --- | --- | --- | --- | --- | --- | --- | --- | --- | --- | --- | --- | --- | --- | --- | --- | --- | --- | --- | --- | --- | --- | --- | --- | --- | --- | --- | --- | --- | --- | --- | --- | --- | --- | --- | --- | --- | --- | --- | --- | --- | --- | --- | --- | --- | --- | --- | --- | --- | --- | --- | --- | --- | --- | --- | --- | --- | --- | --- | --- | --- | --- | --- | --- | --- | --- | --- | --- | --- | --- | --- | --- | --- | --- | --- | --- | --- | --- | --- | --- | --- | --- | --- | --- | --- | --- | --- | --- | --- | --- | --- | --- | --- | --- | --- | --- | --- | --- | --- | --- | --- | --- | --- | --- | --- | --- | --- | --- | --- | --- | --- | --- | --- | --- | --- | --- | --- | --- | --- | --- | --- | --- | --- | --- | --- | --- | --- | --- | --- | --- | --- | --- | --- | --- | --- | --- | --- | --- | --- | --- | --- | --- | --- | --- | --- | --- | --- | --- | --- | --- | --- | --- | --- | --- | --- | --- | --- | --- | --- | --- | --- | --- | --- | --- | --- | --- | --- | --- | --- | --- | --- | --- | --- | --- | --- | --- | --- | --- | --- | --- | --- | --- | --- | --- | --- | --- | --- | --- | --- | --- | --- | --- | --- | --- | --- | --- | --- | --- | --- | --- | --- | --- | --- | --- | --- | --- | --- | --- | --- | --- | --- | --- | --- | --- | --- | --- | --- | --- | --- | --- | --- | --- | --- | --- | --- | --- | --- | --- | --- | --- | --- | --- | --- | --- | --- | --- | --- | --- | --- | --- | --- | --- | --- | --- | --- | --- | --- | --- | --- | --- | --- | --- | --- | --- | --- | --- | --- | --- | --- | --- | --- | --- | --- | --- | --- | --- | --- | --- | --- | --- | --- | --- | --- | --- | --- | --- | --- | --- | --- | --- | --- | --- | --- | --- | --- | --- | --- | --- | --- | --- | --- | --- | --- | --- | --- | --- | --- | --- | --- | --- | --- | --- | --- | --- | --- | --- | --- | --- | --- | --- | --- | --- | --- | --- | --- | --- | --- | --- | --- | --- | --- | --- | --- | --- | --- | --- | --- | --- | --- | --- | --- | --- | --- | --- | --- | --- | --- | --- | --- | --- | --- | --- | --- | --- | --- | --- | --- | --- | --- | --- | --- | --- | --- | --- | --- | --- | --- | --- | --- | --- | --- | --- | --- | --- | --- | --- | --- | --- | --- | --- | --- | --- | --- | --- | --- | --- | --- | --- | --- | --- | --- | --- | --- | --- | --- | --- | --- | --- | --- | --- | --- | --- | --- | --- | --- | --- | --- | --- | --- | --- | --- | --- | --- | --- | --- | --- | --- | --- | --- | --- | --- | --- | --- | --- | --- | --- | --- | --- | --- | --- | --- | --- | --- | --- | --- | --- | --- | --- | --- | --- | --- | --- | --- | --- | --- | --- | --- | --- | --- | --- | --- | --- | --- | --- | --- | --- | --- | --- | --- | --- | --- | --- | --- | --- | --- | --- | --- | --- | --- | --- | --- | --- | --- | --- | --- | --- | --- | --- | --- | --- | --- | --- | --- | --- | --- | --- | --- | --- | --- | --- | --- | --- | --- | --- | --- | --- | --- | --- | --- | --- | --- | --- | --- | --- | --- | --- | --- | --- | --- | --- | --- | --- | --- | --- | --- | --- | --- | --- | --- | --- | --- | --- | --- | --- | --- | --- | --- | --- | --- | --- | --- | --- | --- | --- | --- | --- | --- | --- | --- | --- | --- | --- | --- | --- | --- | --- | --- | --- | --- | --- | --- | --- | --- | --- | --- | --- | --- | --- | --- | --- | --- | --- | --- | --- | --- | --- | --- | --- | --- | --- | --- | --- | --- | --- | --- | --- | --- | --- | --- | --- | --- | --- | --- | --- | --- | --- | --- | --- | --- | --- | --- | --- | --- | --- | --- | --- | --- | --- | --- | --- | --- | --- | --- | --- | --- | --- | --- | --- | --- | --- | --- | --- | --- | --- | --- | --- | --- | --- | --- | --- | --- | --- | --- | --- | --- | --- | --- | --- | --- | --- | --- | --- | --- | --- | --- | --- | --- | --- | --- | --- | --- | --- | --- | --- | --- | --- | --- | --- | --- | --- | --- | --- | --- | --- | --- | --- | --- | --- | --- | --- | --- | --- | --- | --- | --- | --- | --- | --- | --- | --- | --- | --- | --- | --- | --- | --- | --- | --- | --- | --- | --- | --- | --- | --- | --- | --- | --- | --- | --- | --- | --- | --- | --- | --- | --- | --- | --- | --- | --- | --- | --- | --- | --- | --- | --- | --- | --- | --- | --- | --- | --- | --- | --- | --- | --- | --- | --- | --- | --- | --- | --- | --- | --- | --- | --- | --- | --- | --- | --- | --- | --- | --- | --- | --- | --- | --- | --- | --- | --- | --- | --- | --- | --- | --- | --- | --- | --- | --- | --- | --- | --- | --- | --- | --- | --- | --- | --- | --- | --- | --- | --- | --- | --- | --- | --- | --- | --- | --- | --- | --- | --- | --- | --- | --- | --- | --- | --- | --- | --- | --- | --- | --- | --- | --- | --- | --- | --- | --- | --- | --- | --- | --- | --- | --- | --- | --- | --- | --- | --- | --- | --- | --- | --- | --- | --- | --- | --- | --- | --- | --- | --- | --- | --- | --- | --- | --- | --- | --- | --- | --- | --- | --- | --- | --- | --- | --- | --- | --- | --- | --- | --- | --- | --- | --- | --- | --- | --- | --- | --- | --- | --- | --- | --- | --- | --- | --- | --- | --- | --- | --- | --- | --- | --- | --- | --- | --- | --- | --- | --- | --- | --- | --- | --- | --- | --- | --- | --- | --- | --- | --- | --- | --- | --- | --- | --- | --- | --- | --- | --- | --- | --- | --- | --- | --- | --- | --- | --- | --- | --- | --- | --- | --- | --- | --- | --- | --- | --- | --- | --- | --- | --- | --- | --- | --- | --- | --- | --- | --- | --- | --- | --- | --- | --- | --- | --- | --- | --- | --- | --- | --- | --- | --- | --- | --- | --- | --- | --- | --- | --- | --- | --- | --- | --- | --- | --- | --- | --- | --- | --- | --- | --- | --- | --- | --- | --- | --- | --- | --- | --- | --- | --- | --- | --- | --- | --- | --- | --- | --- | --- | --- | --- | --- | --- | --- | --- | --- | --- | --- | --- | --- | --- | --- | --- | --- | --- | --- | --- | --- | --- | --- | --- | --- | --- | --- | --- | --- | --- | --- | --- | --- | --- | --- | --- | --- | --- | --- | --- | --- | --- | --- | --- | --- | --- | --- | --- | --- | --- | --- | --- | --- | --- | --- | --- | --- | --- | --- | --- | --- | --- | --- | --- | --- | --- | --- | --- | --- | --- | --- | --- | --- | --- | --- | --- | --- | --- | --- | --- | --- | --- | --- | --- | --- | --- | --- | --- | --- | --- | --- | --- | --- | --- | --- | --- | --- | --- | --- | --- | --- | --- | --- | --- | --- | --- | --- | --- | --- | --- | --- | --- | --- | --- | --- | --- | --- | --- | --- | --- | --- | --- | --- | --- | --- | --- | --- | --- | --- | --- | --- | --- | --- | --- | --- | --- | --- | --- | --- | --- | --- | --- | --- | --- | --- | --- | --- | --- | --- | --- | --- | --- | --- | --- | --- | --- | --- | --- | --- | --- | --- | --- | --- | --- | --- | --- | --- | --- | --- | --- | --- | --- | --- | --- | --- | --- | --- | --- | --- | --- | --- | --- | --- | --- | --- | --- | --- | --- | --- | --- | --- | --- | --- | --- | --- | --- | --- | --- | --- | --- | --- | --- | --- | --- | --- | --- | --- | --- | --- | --- | --- | --- | --- | --- | --- | --- | --- | --- | --- | --- | --- | --- | --- | --- | --- | --- | --- | --- | --- | --- | --- | --- | --- | --- | --- | --- | --- | --- | --- | --- | --- | --- | --- | --- | --- | --- | --- | --- | --- | --- | --- | --- | --- | --- | --- | --- | --- | --- | --- | --- | --- | --- | --- | --- | --- | --- | --- | --- | --- | --- | --- | --- | --- | --- | --- | --- | --- | --- | --- | --- | --- | --- | --- | --- | --- | --- | --- | --- | --- | --- | --- | --- | --- | --- | --- | --- | --- | --- | --- | --- | --- | --- | --- | --- | --- | --- | --- | --- | --- | --- | --- | --- | --- | --- | --- | --- | --- | --- | --- | --- | --- | --- | --- | --- | --- | --- | --- | --- | --- | --- | --- | --- | --- | --- | --- | --- | --- | --- | --- | --- | --- | --- | --- | --- | --- | --- | --- | --- | --- | --- | --- | --- | --- | --- | --- | --- | --- | --- | --- | --- | --- | --- | --- | --- | --- | --- | --- | --- | --- | --- | --- | --- | --- | --- | --- | --- | --- | --- | --- | --- | --- | --- | --- | --- | --- | --- | --- | --- | --- | --- | --- | --- | --- | --- | --- | --- | --- | --- | --- | --- | --- | --- | --- | --- | --- | --- | --- | --- | --- | --- | --- | --- | --- | --- | --- | --- | --- | --- | --- | --- | --- | --- | --- | --- | --- | --- | --- | --- | --- | --- | --- | --- | --- | --- | --- | --- | --- | --- | --- | --- | --- | --- | --- | --- | --- | --- | --- | --- | --- | --- | --- | --- | --- | --- | --- | --- | --- | --- | --- | --- | --- | --- | --- | --- | --- | --- | --- | --- | --- | --- | --- | --- | --- | --- | --- | --- | --- | --- | --- | --- | --- | --- | --- | --- | --- | --- | --- | --- | --- | --- | --- | --- | --- | --- | --- | --- | --- | --- | --- | --- | --- | --- | --- | --- | --- | --- | --- | --- | --- | --- | --- | --- | --- | --- | --- | --- | --- | --- | --- | --- | --- | --- | --- | --- | --- | --- | --- | --- | --- | --- | --- | --- | --- | --- | --- | --- | --- | --- | --- | --- | --- | --- | --- | --- | --- | --- | --- | --- | --- | --- | --- | --- | --- | --- | --- | --- | --- | --- | --- | --- | --- | --- | --- | --- | --- | --- | --- | --- | --- | --- | --- | --- | --- | --- | --- | --- | --- | --- | --- | --- | --- | --- | --- | --- | --- | --- | --- | --- | --- | --- | --- | --- | --- | --- | --- | --- | --- | --- | --- | --- | --- | --- | --- | --- | --- | --- | --- | --- | --- | --- | --- | --- | --- | --- | --- | --- | --- | --- | --- | --- | --- | --- | --- | --- | --- | --- | --- | --- | --- | --- | --- | --- | --- | --- | --- | --- | --- | --- | --- | --- | --- | --- | --- | --- | --- | --- | --- | --- | --- | --- | --- | --- | --- | --- | --- | --- | --- | --- | --- | --- | --- | --- | --- | --- | --- | --- | --- | --- | --- | --- | --- | --- | --- | --- | --- | --- | --- | --- | --- | --- | --- | --- | --- | --- | --- | --- | --- | --- | --- | --- | --- | --- | --- | --- | --- | --- | --- | --- | --- | --- | --- | --- | --- | --- | --- | --- | --- | --- | --- | --- | --- | --- | --- | --- | --- | --- | --- | --- | --- | --- | --- | --- | --- | --- | --- | --- | --- | --- | --- | --- | --- | --- | --- | --- | --- | --- | --- | --- | --- | --- | --- | --- | --- | --- | --- | --- | --- | --- | --- | --- | --- | --- | --- | --- | --- | --- | --- | --- | --- | --- | --- | --- | --- | --- | --- | --- | --- | --- | --- | --- | --- | --- | --- | --- | --- | --- | --- | --- | --- | --- | --- | --- | --- | --- | --- | --- | --- | --- | --- | --- | --- | --- | --- | --- | --- | --- | --- | --- | --- | --- | --- | --- | --- | --- | --- | --- | --- | --- | --- | --- | --- | --- | --- | --- | --- | --- | --- | --- | --- | --- | --- | --- | --- | --- | --- | --- | --- | --- | --- | --- | --- | --- | --- | --- | --- | --- | --- | --- | --- | --- | --- | --- | --- | --- | --- | --- | --- | --- | --- | --- | --- | --- | --- | --- | --- | --- | --- | --- | --- | --- | --- | --- | --- | --- | --- | --- | --- | --- | --- | --- | --- | --- | --- | --- | --- | --- | --- | --- | --- | --- | --- | --- | --- | --- | --- | --- | --- | --- | --- | --- | --- | --- | --- | --- | --- | --- | --- | --- | --- | --- | --- | --- | --- | --- | --- | --- | --- | --- | --- | --- | --- | --- | --- | --- | --- | --- | --- | --- | --- | --- | --- | --- | --- | --- | --- | --- | --- | --- | --- | --- | --- | --- | --- | --- | --- | --- | --- | --- | --- | --- | --- | --- | --- | --- | --- | --- | --- | --- | --- | --- | --- | --- | --- | --- | --- | --- | --- | --- | --- | --- | --- | --- | --- | --- | --- | --- | --- | --- | --- | --- | --- | --- | --- | --- | --- | --- | --- | --- | --- | --- | --- | --- | --- | --- | --- | --- | --- | --- | --- | --- | --- | --- | --- | --- | --- | --- | --- | --- | --- | --- | --- | --- | --- | --- | --- | --- | --- | --- | --- | --- | --- | --- | --- | --- | --- | --- | --- | --- | --- | --- | --- | --- | --- | --- | --- | --- | --- | --- | --- | --- | --- | --- | --- | --- | --- | --- | --- | --- | --- | --- | --- | --- | --- | --- | --- | --- | --- | --- | --- | --- | --- | --- | --- | --- | --- | --- | --- | --- | --- | --- | --- | --- | --- | --- | --- | --- | --- | --- | --- | --- | --- | --- | --- | --- | --- | --- | --- | --- | --- | --- | --- | --- | --- | --- | --- | --- | --- | --- | --- | --- | --- | --- | --- | --- | --- | --- | --- | --- | --- | --- | --- | --- | --- | --- | --- | --- | --- | --- | --- | --- | --- | --- | --- | --- | --- | --- | --- | --- | --- | --- | --- | --- | --- | --- | --- | --- | --- | --- | --- | --- | --- | --- | --- | --- | --- | --- | --- | --- | --- | --- | --- | --- | --- | --- | --- | --- | --- | --- | --- | --- | --- | --- | --- | --- | --- | --- | --- | --- | --- | --- | --- | --- | --- | --- | --- | --- | --- | --- | --- | --- | --- | --- | --- | --- | --- | --- | --- | --- | --- | --- | --- | --- | --- | --- | --- | --- | --- | --- | --- | --- | --- | --- | --- | --- | --- | --- | --- | --- | --- | --- | --- | --- | --- | --- | --- | --- | --- | --- | --- | --- | --- | --- | --- | --- | --- | --- | --- | --- | --- | --- | --- | --- | --- | --- | --- | --- | --- | --- | --- | --- | --- | --- | --- | --- | --- | --- | --- | --- | --- | --- | --- | --- | --- | --- | --- | --- | --- | --- | --- | --- | --- | --- | --- | --- | --- | --- | --- | --- | --- | --- | --- | --- | --- | --- | --- | --- | --- | --- | --- | --- | --- | --- | --- | --- | --- | --- | --- | --- | --- | --- | --- | --- | --- | --- | --- | --- | --- | --- | --- | --- | --- | --- | --- | --- | --- | --- | --- | --- | --- | --- | --- | --- | --- | --- | --- | --- | --- | --- | --- | --- | --- | --- | --- | --- | --- | --- | --- | --- | --- | --- | --- | --- | --- | --- | --- | --- | --- | --- | --- | --- | --- | --- | --- | --- | --- | --- | --- | --- | --- | --- | --- | --- | --- | --- | --- | --- | --- | --- | --- | --- | --- | --- | --- | --- | --- | --- | --- | --- | --- | --- | --- | --- | --- | --- | --- | --- | --- | --- | --- | --- | --- | --- | --- | --- | --- | --- | --- | --- | --- | --- | --- | --- | --- | --- | --- | --- | --- | --- | --- | --- | --- | --- | --- | --- | --- | --- | --- | --- | --- | --- | --- | --- | --- | --- | --- | --- | --- | --- | --- | --- | --- | --- | --- | --- | --- | --- | --- | --- | --- | --- | --- | --- | --- | --- | --- | --- | --- | --- | --- | --- | --- | --- | --- | --- | --- | --- | --- | --- | --- | --- | --- | --- | --- | --- | --- | --- | --- | --- | --- | --- | --- | --- | --- | --- | --- | --- | --- | --- | --- | --- | --- | --- | --- | --- | --- | --- | --- | --- | --- | --- | --- | --- | --- | --- | --- | --- | --- | --- | --- | --- | --- | --- | --- | --- | --- | --- | --- | --- | --- | --- | --- | --- | --- | --- | --- | --- | --- | --- | --- | --- | --- | --- | --- | --- | --- | --- | --- | --- | --- | --- | --- | --- | --- | --- | --- | --- | --- | --- | --- | --- | --- | --- | --- | --- | --- | --- | --- | --- | --- | --- | --- | --- | --- | --- | --- | --- | --- | --- | --- | --- | --- | --- | --- | --- | --- | --- | --- | --- | --- | --- | --- | --- | --- | --- | --- | --- | --- | --- | --- | --- | --- | --- | --- | --- | --- | --- | --- | --- | --- | --- | --- | --- | --- | --- | --- | --- | --- | --- | --- | --- | --- | --- | --- | --- | --- | --- | --- | --- | --- | --- | --- | --- | --- | --- | --- | --- | --- | --- | --- | --- | --- | --- | --- | --- | --- | --- | --- | --- | --- | --- | --- | --- | --- | --- | --- | --- | --- | --- | --- | --- | --- | --- | --- | --- | --- | --- | --- | --- | --- | --- | --- | --- | --- | --- | --- | --- | --- | --- | --- | --- | --- | --- | --- | --- | --- | --- | --- | --- | --- | --- | --- | --- | --- | --- | --- | --- | --- | --- | --- | --- | --- | --- | --- | --- | --- | --- | --- | --- | --- | --- | --- | --- | --- | --- | --- | --- | --- | --- | --- | --- | --- | --- | --- | --- | --- | --- | --- | --- | --- | --- | --- | --- | --- | --- | --- | --- | --- | --- | --- | --- | --- | --- | --- | --- | --- | --- | --- | --- | --- | --- | --- | --- | --- | --- | --- | --- | --- | --- | --- | --- | --- | --- | --- | --- | --- | --- | --- | --- | --- | --- | --- | --- | --- | --- | --- | --- | --- | --- | --- | --- | --- | --- | --- | --- | --- | --- | --- | --- | --- | --- | --- | --- | --- | --- | --- | --- | --- | --- | --- | --- | --- | --- | --- | --- | --- | --- | --- | --- | --- | --- | --- | --- | --- | --- | --- | --- | --- | --- | --- | --- | --- | --- | --- | --- | --- | --- | --- | --- | --- | --- | --- | --- | --- | --- | --- | --- | --- | --- | --- | --- | --- | --- | --- | --- | --- | --- | --- | --- | --- | --- | --- | --- | --- | --- | --- | --- | --- | --- | --- | --- | --- | --- | --- | --- | --- | --- | --- | --- | --- | --- | --- | --- | --- | --- | --- | --- | --- | --- | --- | --- | --- | --- | --- | --- | --- | --- | --- | --- | --- | --- | --- | --- | --- | --- | --- | --- | --- | --- | --- | --- | --- | --- | --- | --- | --- | --- | --- | --- | --- | --- | --- | --- | --- | --- | --- | --- | --- | --- | --- | --- | --- | --- | --- | --- | --- | --- | --- | --- | --- | --- | --- | --- | --- | --- | --- | --- | --- | --- | --- | --- | --- | --- | --- | --- | --- | --- | --- | --- | --- | --- | --- | --- | --- | --- | --- | --- | --- | --- | --- | --- | --- | --- | --- | --- | --- | --- | --- | --- | --- | --- | --- | --- | --- | --- | --- | --- | --- | --- | --- | --- | --- | --- | --- | --- | --- | --- | --- | --- | --- | --- | --- | --- | --- | --- | --- | --- | --- | --- | --- | --- | --- | --- | --- | --- | --- | --- | --- | --- | --- | --- | --- | --- | --- | --- | --- | --- | --- | --- | --- | --- | --- | --- | --- | --- | --- | --- | --- | --- | --- | --- | --- | --- | --- | --- | --- | --- | --- | --- | --- | --- | --- | --- | --- | --- | --- | --- | --- | --- | --- | --- | --- | --- | --- | --- | --- | --- | --- | --- | --- | --- | --- | --- | --- | --- | --- | --- | --- | --- | --- | --- | --- | --- | --- | --- | --- | --- | --- | --- | --- | --- | --- | --- | --- | --- | --- | --- | --- | --- | --- | --- | --- | --- | --- | --- | --- | --- | --- | --- | --- | --- | --- | --- | --- | --- | --- | --- | --- | --- | --- | --- | --- | --- | --- | --- | --- | --- | --- | --- | --- | --- | --- | --- | --- | --- | --- | --- | --- | --- | --- | --- | --- | --- | --- | --- | --- | --- | --- | --- | --- | --- | --- | --- | --- | --- | --- | --- | --- | --- | --- | --- | --- | --- | --- | --- | --- | --- | --- | --- | --- | --- | --- | --- | --- | --- | --- | --- | --- | --- | --- | --- | --- | --- | --- | --- | --- | --- | --- | --- | --- | --- | --- | --- | --- | --- | --- | --- | --- | --- | --- | --- | --- | --- | --- | --- | --- | --- | --- | --- | --- | --- | --- | --- | --- | --- | --- | --- | --- | --- | --- | --- | --- | --- | --- | --- | --- | --- | --- | --- | --- | --- | --- | --- | --- | --- | --- | --- | --- | --- | --- | --- | --- | --- | --- | --- | --- | --- | --- | --- | --- | --- | --- | --- | --- | --- | --- | --- | --- | --- | --- | --- | --- | --- | --- | --- | --- | --- | --- | --- | --- | --- | --- | --- | --- | --- | --- | --- | --- | --- | --- | --- | --- | --- | --- | --- | --- | --- | --- | --- | --- | --- | --- | --- | --- | --- | --- | --- | --- | --- | --- | --- | --- | --- | --- | --- | --- | --- | --- | --- | --- | --- | --- | --- | --- | --- | --- | --- | --- | --- | --- | --- | --- | --- | --- | --- | --- | --- | --- | --- | --- | --- | --- | --- | --- | --- | --- | --- | --- | --- | --- | --- | --- | --- | --- | --- | --- | --- | --- | --- | --- | --- | --- | --- | --- | --- | --- | --- | --- | --- | --- | --- | --- | --- | --- | --- | --- | --- | --- | --- | --- | --- | --- | --- | --- | --- | --- | --- | --- | --- | --- | --- | --- | --- | --- | --- | --- | --- | --- | --- | --- | --- | --- | --- | --- | --- | --- | --- | --- | --- | --- | --- | --- | --- | --- | --- | --- | --- | --- | --- | --- | --- | --- | --- | --- | --- | --- | --- | --- | --- | --- | --- | --- | --- | --- | --- | --- | --- | --- | --- | --- | --- | --- | --- | --- | --- | --- | --- | --- | --- | --- | --- | --- | --- | --- | --- | --- | --- | --- | --- | --- | --- | --- | --- | --- | --- | --- | --- | --- | --- | --- | --- | --- | --- | --- | --- | --- | --- | --- | --- | --- | --- | --- | --- | --- | --- | --- | --- | --- | --- | --- | --- | --- | --- | --- | --- | --- | --- | --- | --- | --- | --- | --- | --- | --- | --- | --- | --- | --- | --- | --- | --- | --- | --- | --- | --- | --- | --- | --- | --- | --- | --- | --- | --- | --- | --- | --- | --- | --- | --- | --- | --- | --- | --- | --- | --- | --- | --- | --- | --- | --- | --- | --- | --- | --- | --- | --- | --- | --- | --- | --- | --- | --- | --- | --- | --- | --- | --- | --- | --- | --- | --- | --- | --- | --- | --- | --- | --- | --- | --- | --- | --- | --- | --- | --- | --- | --- | --- | --- | --- | --- | --- | --- | --- | --- | --- | --- | --- | --- | --- | --- | --- | --- | --- | --- | --- | --- | --- | --- | --- | --- | --- | --- | --- | --- | --- | --- | --- | --- | --- | --- | --- | --- | --- | --- | --- | --- | --- | --- | --- | --- | --- | --- | --- | --- | --- | --- | --- | --- | --- | --- | --- | --- | --- | --- | --- | --- | --- | --- | --- | --- | --- | --- | --- | --- | --- | --- | --- | --- | --- | --- | --- | --- | --- | --- | --- | --- | --- | --- | --- | --- | --- | --- | --- | --- | --- | --- | --- | --- | --- | --- | --- | --- | --- | --- | --- | --- | --- | --- | --- | --- | --- | --- | --- | --- | --- | --- | --- | --- | --- | --- | --- | --- | --- | --- | --- | --- | --- | --- | --- | --- | --- | --- | --- | --- | --- | --- | --- | --- | --- | --- | --- | --- | --- | --- | --- | --- | --- | --- | --- | --- | --- | --- | --- | --- | --- | --- | --- | --- | --- | --- | --- | --- | --- | --- | --- | --- | --- | --- | --- | --- | --- | --- | --- | --- | --- | --- | --- | --- | --- | --- | --- | --- | --- | --- | --- | --- | --- | --- | --- | --- | --- | --- | --- | --- | --- | --- | --- | --- | --- | --- | --- | --- | --- | --- | --- | --- | --- | --- | --- | --- | --- | --- | --- | --- | --- | --- | --- | --- | --- | --- | --- | --- | --- | --- | --- | --- | --- | --- | --- | --- | --- | --- | --- | --- | --- | --- | --- | --- | --- | --- | --- | --- | --- | --- | --- | --- | --- | --- | --- | --- | --- | --- | --- | --- | --- | --- | --- | --- | --- | --- | --- | --- | --- | --- | --- | --- | --- | --- | --- | --- | --- | --- | --- | --- | --- | --- | --- | --- | --- | --- | --- | --- | --- | --- | --- | --- | --- | --- | --- | --- | --- | --- | --- | --- | --- | --- | --- | --- | --- | --- | --- | --- | --- | --- | --- | --- | --- | --- | --- | --- | --- | --- | --- | --- | --- | --- | --- | --- | --- | --- | --- | --- | --- | --- | --- | --- | --- | --- | --- | --- | --- | --- | --- | --- | --- | --- | --- | --- | --- | --- | --- | --- | --- | --- | --- | --- | --- | --- | --- | --- | --- | --- | --- | --- | --- | --- | --- | --- | --- | --- | --- | --- | --- | --- | --- | --- | --- | --- | --- | --- | --- | --- | --- | --- | --- | --- | --- | --- | --- | --- | --- | --- | --- | --- | --- | --- | --- | --- | --- | --- | --- | --- | --- | --- | --- | --- | --- | --- | --- | --- | --- | --- | --- | --- | --- | --- | --- | --- | --- | --- | --- | --- | --- | --- | --- | --- | --- | --- | --- | --- | --- | --- | --- | --- | --- | --- | --- | --- | --- | --- | --- | --- | --- | --- | --- | --- | --- | --- | --- | --- | --- | --- | --- | --- | --- | --- | --- | --- | --- | --- | --- | --- | --- | --- | --- | --- | --- | --- | --- | --- | --- | --- | --- | --- | --- | --- | --- | --- | --- | --- | --- | --- | --- | --- | --- | --- | --- | --- | --- | --- | --- | --- | --- | --- | --- | --- | --- | --- | --- | --- | --- | --- | --- | --- | --- | --- | --- | --- | --- | --- | --- | --- | --- | --- | --- | --- | --- | --- | --- | --- | --- | --- | --- | --- | --- | --- | --- | --- | --- | --- | --- | --- | --- | --- | --- | --- | --- | --- | --- | --- | --- | --- | --- | --- | --- | --- | --- | --- | --- | --- | --- | --- | --- | --- | --- | --- | --- | --- | --- | --- | --- | --- | --- | --- | --- | --- | --- | --- | --- | --- | --- | --- | --- | --- | --- | --- | --- | --- | --- | --- | --- | --- | --- | --- | --- | --- | --- | --- | --- | --- | --- | --- | --- | --- | --- | --- | --- | --- | --- | --- | --- | --- | --- | --- | --- | --- | --- | --- | --- | --- | --- | --- | --- | --- | --- | --- | --- | --- | --- | --- | --- | --- | --- | --- | --- | --- | --- | --- | --- | --- | --- | --- | --- | --- | --- | --- | --- | --- | --- | --- | --- | --- | --- | --- | --- | --- | --- | --- | --- | --- | --- | --- | --- | --- | --- | --- | --- | --- | --- | --- | --- | --- | --- | --- | --- | --- | --- | --- | --- | --- | --- | --- | --- | --- | --- | --- | --- | --- | --- | --- | --- | --- | --- | --- | --- | --- | --- | --- | --- | --- | --- | --- | --- | --- | --- | --- | --- | --- | --- | --- | --- | --- | --- | --- | --- | --- | --- | --- | --- | --- | --- | --- | --- | --- | --- | --- | --- | --- | --- | --- | --- | --- | --- | --- | --- | --- | --- | --- | --- | --- | --- | --- | --- | --- | --- | --- | --- | --- | --- | --- | --- | --- | --- | --- | --- | --- | --- | --- | --- | --- | --- | --- | --- | --- | --- | --- | --- | --- | --- | --- | --- | --- | --- | --- | --- | --- | --- | --- | --- | --- | --- | --- | --- | --- | --- | --- | --- | --- | --- | --- | --- | --- | --- | --- | --- | --- | --- | --- | --- | --- | --- | --- | --- | --- | --- | --- | --- | --- | --- | --- | --- | --- | --- | --- | --- | --- | --- | --- | --- | --- | --- | --- | --- | --- | --- | --- | --- | --- | --- | --- | --- | --- | --- | --- | --- | --- | --- | --- | --- | --- | --- | --- | --- | --- | --- | --- | --- | --- | --- | --- | --- | --- | --- | --- | --- | --- | --- | --- | --- | --- | --- | --- | --- | --- | --- | --- | --- | --- | --- | --- | --- | --- | --- | --- | --- | --- | --- | --- | --- | --- | --- | --- | --- | --- | --- | --- | --- | --- | --- | --- | --- | --- | --- | --- | --- | --- | --- | --- | --- | --- | --- | --- | --- | --- | --- | --- | --- | --- | --- | --- | --- | --- | --- | --- | --- | --- | --- | --- | --- | --- | --- | --- | --- | --- | --- | --- | --- | --- | --- | --- | --- | --- | --- | --- | --- | --- | --- | --- | --- | --- | --- | --- | --- | --- | --- | --- | --- | --- | --- | --- | --- | --- | --- | --- | --- | --- | --- | --- | --- | --- | --- | --- | --- | --- | --- | --- | --- | --- | --- | --- | --- | --- | --- | --- | --- | --- | --- | --- | --- | --- | --- | --- | --- | --- | --- | --- | --- | --- | --- | --- | --- | --- | --- | --- | --- | --- | --- | --- | --- | --- | --- | --- | --- | --- | --- | --- | --- | --- | --- | --- | --- | --- | --- | --- | --- | --- | --- | --- | --- | --- | --- | --- | --- | --- | --- | --- | --- | --- | --- | --- | --- | --- | --- | --- | --- | --- | --- | --- | --- | --- | --- | --- | --- | --- | --- | --- | --- | --- | --- | --- | --- | --- | --- | --- | --- | --- | --- | --- | --- | --- | --- | --- | --- | --- | --- | --- | --- | --- | --- | --- | --- | --- | --- | --- | --- | --- | --- | --- | --- | --- | --- | --- | --- | --- | --- | --- | --- | --- | --- | --- | --- | --- | --- | --- | --- | --- | --- | --- | --- | --- | --- | --- | --- | --- | --- | --- | --- | --- | --- | --- | --- | --- | --- | --- | --- | --- | --- | --- | --- | --- | --- | --- | --- | --- | --- | --- | --- | --- | --- | --- | --- | --- | --- | --- | --- | --- | --- | --- | --- | --- | --- | --- | --- | --- | --- | --- | --- | --- | --- | --- | --- | --- | --- | --- | --- | --- | --- | --- | --- | --- | --- | --- | --- | --- | --- | --- | --- | --- | --- | --- | --- | --- | --- | --- | --- | --- | --- | --- | --- | --- | --- | --- | --- | --- | --- | --- | --- | --- | --- | --- | --- | --- | --- | --- | --- | --- | --- | --- | --- | --- | --- | --- | --- | --- | --- | --- | --- | --- | --- | --- | --- | --- | --- | --- | --- | --- | --- | --- | --- | --- | --- | --- | --- | --- | --- | --- | --- | --- | --- | --- | --- | --- | --- | --- | --- | --- | --- | --- | --- | --- | --- | --- | --- | --- | --- | --- | --- | --- | --- | --- | --- | --- | --- | --- | --- | --- | --- | --- | --- | --- | --- | --- | --- | --- | --- | --- | --- | --- | --- | --- | --- | --- | --- | --- | --- | --- | --- | --- | --- | --- | --- | --- | --- | --- | --- | --- | --- | --- | --- | --- | --- | --- | --- | --- | --- | --- | --- | --- | --- | --- | --- | --- | --- | --- | --- | --- | --- | --- | --- | --- | --- | --- | --- | --- | --- | --- | --- | --- | --- | --- | --- | --- | --- | --- | --- | --- | --- | --- | --- | --- | --- | --- | --- | --- | --- | --- | --- | --- | --- | --- | --- | --- | --- | --- | --- | --- | --- | --- | --- | --- | --- | --- | --- | --- | --- | --- | --- | --- | --- | --- | --- | --- | --- | --- | --- | --- | --- | --- | --- | --- | --- | --- | --- | --- | --- | --- | --- | --- | --- | --- | --- | --- | --- | --- | --- | --- | --- | --- | --- | --- | --- | --- | --- | --- | --- | --- | --- | --- | --- | --- | --- | --- | --- | --- | --- | --- | --- | --- | --- | --- | --- | --- | --- | --- | --- | --- | --- | --- | --- | --- | --- | --- | --- | --- | --- | --- | --- | --- | --- | --- | --- | --- | --- | --- | --- | --- | --- | --- | --- | --- | --- | --- | --- | --- | --- | --- | --- | --- | --- | --- | --- | --- | --- | --- | --- | --- | --- | --- | --- | --- | --- | --- | --- | --- | --- | --- | --- | --- | --- | --- | --- | --- | --- | --- | --- | --- | --- | --- | --- | --- | --- | --- | --- | --- | --- | --- | --- | --- | --- | --- | --- | --- | --- | --- | --- | --- | --- | --- | --- | --- | --- | --- | --- | --- | --- | --- | --- | --- | --- | --- | --- | --- | --- | --- | --- | --- | --- | --- | --- | --- | --- | --- | --- | --- | --- | --- | --- | --- | --- | --- | --- | --- | --- | --- | --- | --- | --- | --- | --- | --- | --- | --- | --- | --- | --- | --- | --- | --- | --- | --- | --- | --- | --- | --- | --- | --- | --- | --- | --- | --- | --- | --- | --- | --- | --- | --- | --- | --- | --- | --- | --- | --- | --- | --- | --- | --- | --- | --- | --- | --- | --- | --- | --- | --- | --- | --- | --- | --- | --- | --- | --- | --- | --- | --- | --- | --- | --- | --- | --- | --- | --- | --- | --- | --- | --- | --- | --- | --- | --- | --- | --- | --- | --- | --- | --- | --- | --- | --- | --- | --- | --- | --- | --- | --- | --- | --- | --- | --- | --- | --- | --- | --- | --- | --- | --- | --- | --- | --- | --- | --- | --- | --- | --- | --- | --- | --- | --- | --- | --- | --- | --- | --- | --- | --- | --- | --- | --- | --- | --- | --- | --- | --- | --- | --- | --- | --- | --- | --- | --- | --- | --- | --- | --- | --- | --- | --- | --- | --- | --- | --- | --- | --- | --- | --- | --- | --- | --- | --- | --- | --- | --- | --- | --- | --- | --- | --- | --- | --- | --- | --- | --- | --- | --- | --- | --- | --- | --- | --- | --- | --- | --- | --- | --- | --- | --- | --- | --- | --- | --- | --- | --- | --- | --- | --- | --- | --- | --- | --- | --- | --- | --- | --- | --- | --- | --- | --- | --- | --- | --- | --- | --- | --- | --- | --- | --- | --- | --- | --- | --- | --- | --- | --- | --- | --- | --- | --- | --- | --- | --- | --- | --- | --- | --- | --- | --- | --- | --- | --- | --- | --- | --- | --- | --- | --- | --- | --- | --- | --- | --- | --- | --- | --- | --- | --- | --- | --- | --- | --- | --- | --- | --- | --- | --- | --- | --- | --- | --- | --- | --- | --- | --- | --- | --- | --- | --- | --- | --- | --- | --- | --- | --- | --- | --- | --- | --- | --- | --- | --- | --- | --- | --- | --- | --- | --- | --- | --- | --- | --- | --- | --- | --- | --- | --- | --- | --- | --- | --- | --- | --- | --- | --- | --- | --- | --- | --- | --- | --- | --- | --- | --- | --- | --- | --- | --- | --- | --- | --- | --- | --- | --- | --- | --- | --- | --- | --- | --- | --- | --- | --- | --- | --- | --- | --- | --- | --- | --- | --- | --- | --- | --- | --- | --- | --- | --- | --- | --- | --- | --- | --- | --- | --- | --- | --- | --- | --- | --- | --- | --- | --- | --- | --- | --- | --- | --- | --- | --- | --- | --- | --- | --- | --- | --- | --- | --- | --- | --- | --- | --- | --- | --- | --- | --- | --- | --- | --- | --- | --- | --- | --- | --- | --- | --- | --- | --- | --- | --- | --- | --- | --- | --- | --- | --- | --- | --- | --- | --- | --- | --- | --- | --- | --- | --- | --- | --- | --- | --- | --- | --- | --- | --- | --- | --- | --- | --- | --- | --- | --- | --- | --- | --- | --- | --- | --- | --- | --- | --- | --- | --- | --- | --- | --- | --- | --- | --- | --- | --- | --- | --- | --- | --- | --- | --- | --- | --- | --- | --- | --- | --- | --- | --- | --- | --- | --- | --- | --- | --- | --- | --- | --- | --- | --- | --- | --- | --- | --- | --- | --- | --- | --- | --- | --- | --- | --- | --- | --- | --- | --- | --- | --- | --- | --- | --- | --- | --- | --- | --- | --- | --- | --- | --- | --- | --- | --- | --- | --- | --- | --- | --- | --- | --- | --- | --- | --- | --- | --- | --- | --- | --- | --- | --- | --- | --- | --- | --- | --- | --- | --- | --- | --- | --- | --- | --- | --- | --- | --- | --- | --- | --- | --- | --- | --- | --- | --- | --- | --- | --- | --- | --- | --- | --- | --- | --- | --- | --- | --- | --- | --- | --- | --- | --- | --- | --- | --- | --- | --- | --- | --- | --- | --- | --- | --- | --- | --- | --- | --- | --- | --- | --- | --- | --- | --- | --- | --- | --- | --- | --- | --- | --- | --- | --- | --- | --- | --- | --- | --- | --- | --- | --- | --- | --- | --- | --- | --- | --- | --- | --- | --- | --- | --- | --- | --- | --- | --- | --- | --- | --- | --- | --- | --- | --- | --- | --- | --- | --- | --- | --- | --- | --- | --- | --- | --- | --- | --- | --- | --- | --- | --- | --- | --- | --- | --- | --- | --- | --- | --- | --- | --- | --- | --- | --- | --- | --- | --- | --- | --- | --- | --- | --- | --- | --- | --- | --- | --- | --- | --- | --- | --- | --- | --- | --- | --- | --- | --- | --- | --- | --- | --- | --- | --- | --- | --- | --- | --- | --- | --- | --- | --- | --- | --- | --- | --- | --- | --- | --- | --- | --- | --- | --- | --- | --- | --- | --- | --- | --- | --- | --- | --- | --- | --- | --- | --- | --- | --- | --- | --- | --- | --- | --- | --- | --- | --- | --- | --- | --- | --- | --- | --- | --- | --- | --- | --- | --- | --- | --- | --- | --- | --- | --- | --- | --- | --- | --- | --- | --- | --- | --- | --- | --- | --- | --- | --- | --- | --- | --- | --- | --- | --- | --- | --- | --- | --- | --- | --- | --- | --- | --- | --- | --- | --- | --- | --- | --- | --- | --- | --- | --- | --- | --- | --- | --- | --- | --- | --- | --- | --- | --- | --- | --- | --- | --- | --- | --- | --- | --- | --- | --- | --- | --- | --- | --- | --- | --- | --- | --- | --- | --- | --- | --- | --- | --- | --- | --- | --- | --- | --- | --- | --- | --- | --- | --- | --- | --- | --- | --- | --- | --- | --- | --- | --- | --- | --- | --- | --- | --- | --- | --- | --- | --- | --- | --- | --- | --- | --- | --- | --- | --- | --- | --- | --- | --- | --- | --- | --- | --- | --- | --- | --- | --- | --- | --- | --- | --- | --- | --- | --- | --- | --- | --- | --- | --- | --- | --- | --- | --- | --- | --- | --- | --- | --- | --- | --- | --- | --- | --- | --- | --- | --- | --- | --- | --- | --- | --- | --- | --- | --- | --- | --- | --- | --- | --- | --- | --- | --- | --- | --- | --- | --- | --- | --- | --- | --- | --- | --- | --- | --- | --- | --- | --- | --- | --- | --- | --- | --- | --- | --- | --- | --- | --- | --- | --- | --- | --- | --- | --- | --- | --- | --- | --- | --- | --- | --- | --- | --- | --- | --- | --- | --- | --- | --- | --- | --- | --- | --- | --- | --- | --- | --- | --- | --- | --- | --- | --- | --- | --- | --- | --- | --- | --- | --- | --- | --- | --- | --- | --- | --- | --- | --- | --- | --- | --- | --- | --- | --- | --- | --- | --- | --- | --- | --- | --- | --- | --- | --- | --- | --- | --- | --- | --- | --- | --- | --- | --- | --- | --- | --- | --- | --- | --- | --- | --- | --- | --- | --- | --- | --- | --- | --- | --- | --- | --- | --- | --- | --- | --- | --- | --- | --- | --- | --- | --- | --- | --- | --- | --- | --- | --- | --- | --- | --- | --- | --- | --- | --- | --- | --- | --- | --- | --- | --- | --- | --- | --- | --- | --- | --- | --- | --- | --- | --- | --- | --- | --- | --- | --- | --- | --- | --- | --- | --- | --- | --- | --- | --- | --- | --- | --- | --- | --- | --- | --- | --- | --- | --- | --- | --- | --- | --- | --- | --- | --- | --- | --- | --- | --- | --- | --- | --- | --- | --- | --- | --- | --- | --- | --- | --- | --- | --- | --- | --- | --- | --- | --- | --- | --- | --- | --- | --- | --- | --- | --- | --- | --- | --- | --- | --- | --- | --- | --- | --- | --- | --- | --- | --- | --- | --- | --- | --- | --- | --- | --- | --- | --- | --- | --- | --- | --- | --- | --- | --- | --- | --- | --- | --- | --- | --- | --- | --- | --- | --- | --- | --- | --- | --- | --- | --- | --- | --- | --- | --- | --- | --- | --- | --- | --- | --- | --- | --- | --- | --- | --- | --- | --- | --- | --- | --- | --- | --- | --- | --- | --- | --- | --- | --- | --- | --- | --- | --- | --- | --- | --- | --- | --- | --- | --- | --- | --- | --- | --- | --- | --- | --- | --- | --- | --- | --- | --- | --- | --- | --- | --- | --- | --- | --- | --- | --- | --- | --- | --- | --- | --- | --- | --- | --- | --- | --- | --- | --- | --- | --- | --- | --- | --- | --- | --- | --- | --- | --- | --- | --- | --- | --- | --- | --- | --- | --- | --- | --- | --- | --- | --- | --- | --- | --- | --- | --- | --- | --- | --- | --- | --- | --- | --- | --- | --- | --- | --- | --- | --- | --- | --- | --- | --- | --- | --- | --- | --- | --- | --- | --- | --- | --- | --- | --- | --- | --- | --- | --- | --- | --- | --- | --- | --- | --- | --- | --- | --- | --- | --- | --- | --- | --- | --- | --- | --- | --- | --- | --- | --- | --- | --- | --- | --- | --- | --- | --- | --- | --- | --- | --- | --- | --- | --- | --- | --- | --- | --- | --- | --- | --- | --- | --- | --- | --- | --- | --- | --- | --- | --- | --- | --- | --- | --- | --- | --- | --- | --- | --- | --- | --- | --- | --- | --- | --- | --- | --- | --- | --- | --- | --- | --- | --- | --- | --- | --- | --- | --- | --- | --- | --- | --- | --- | --- | --- | --- | --- | --- | --- | --- | --- | --- | --- | --- | --- | --- | --- | --- | --- | --- | --- | --- | --- | --- | --- | --- | --- | --- | --- | --- | --- | --- | --- | --- | --- | --- | --- | --- | --- | --- | --- | --- | --- | --- | --- | --- | --- | --- | --- | --- | --- | --- | --- | --- | --- | --- | --- | --- | --- | --- | --- | --- | --- | --- | --- | --- | --- | --- | --- | --- | --- | --- | --- | --- | --- | --- | --- | --- | --- | --- | --- | --- | --- | --- | --- | --- | --- | --- | --- | --- | --- | --- | --- | --- | --- | --- | --- | --- | --- | --- | --- | --- | --- | --- | --- | --- | --- | --- | --- | --- | --- | --- | --- | --- | --- | --- | --- | --- | --- | --- | --- | --- | --- | --- | --- | --- | --- | --- | --- | --- | --- | --- | --- | --- | --- | --- | --- | --- | --- | --- | --- | --- | --- | --- | --- | --- | --- | --- | --- | --- | --- | --- | --- | --- | --- | --- | --- | --- | --- | --- | --- | --- | --- | --- | --- | --- | --- | --- | --- | --- | --- | --- | --- | --- | --- | --- | --- | --- | --- | --- | --- | --- | --- | --- | --- | --- | --- | --- | --- | --- | --- | --- | --- | --- | --- | --- | --- | --- | --- | --- | --- | --- | --- | --- | --- | --- | --- | --- | --- | --- | --- | --- | --- | --- | --- | --- | --- | --- | --- | --- | --- | --- | --- | --- | --- | --- | --- | --- | --- | --- | --- | --- | --- | --- | --- | --- | --- | --- | --- | --- | --- | --- | --- | --- | --- | --- | --- | --- | --- | --- | --- | --- | --- | --- | --- | --- | --- | --- | --- | --- | --- | --- | --- | --- | --- | --- | --- | --- | --- | --- | --- | --- | --- | --- | --- | --- | --- | --- | --- | --- | --- | --- | --- | --- | --- | --- | --- | --- | --- | --- | --- | --- | --- | --- | --- | --- | --- | --- | --- | --- | --- | --- | --- | --- | --- | --- | --- | --- | --- | --- | --- | --- | --- | --- | --- | --- | --- | --- | --- | --- | --- | --- | --- | --- | --- | --- | --- | --- | --- | --- | --- | --- | --- | --- | --- | --- | --- | --- | --- | --- | --- | --- | --- | --- | --- | --- | --- | --- | --- | --- | --- | --- | --- | --- | --- | --- | --- | --- | --- | --- | --- | --- | --- | --- | --- | --- | --- | --- | --- | --- | --- | --- | --- | --- | --- | --- | --- | --- | --- | --- | --- | --- | --- | --- | --- | --- | --- | --- | --- | --- | --- | --- | --- | --- | --- | --- | --- | --- | --- | --- | --- | --- | --- | --- | --- | --- | --- | --- | --- | --- | --- | --- | --- | --- | --- | --- | --- | --- | --- | --- | --- | --- | --- | --- | --- | --- | --- | --- | --- | --- | --- | --- | --- | --- | --- | --- | --- | --- | --- | --- | --- | --- | --- | --- | --- | --- | --- | --- | --- | --- | --- | --- | --- | --- | --- | --- | --- | --- | --- | --- | --- | --- | --- | --- | --- | --- | --- | --- | --- | --- | --- | --- | --- | --- | --- | --- | --- | --- | --- | --- | --- | --- | --- | --- | --- | --- | --- | --- | --- | --- | --- | --- | --- | --- | --- | --- | --- | --- | --- | --- | --- | --- | --- | --- | --- | --- | --- | --- | --- | --- | --- | --- | --- | --- | --- | --- | --- | --- | --- | --- | --- | --- | --- | --- | --- | --- | --- | --- | --- | --- | --- | --- | --- | --- | --- | --- | --- | --- | --- | --- | --- | --- | --- | --- | --- | --- | --- | --- | --- | --- | --- | --- | --- | --- | --- | --- | --- | --- | --- | --- | --- | --- | --- | --- | --- | --- | --- | --- | --- | --- | --- | --- | --- | --- | --- | --- | --- | --- | --- | --- | --- | --- | --- | --- | --- | --- | --- | --- | --- | --- | --- | --- | --- | --- | --- | --- | --- | --- | --- | --- | --- | --- | --- | --- | --- | --- | --- | --- | --- | --- | --- | --- | --- | --- | --- | --- | --- | --- | --- | --- | --- | --- | --- | --- | --- | --- | --- | --- | --- | --- | --- | --- | --- | --- | --- | --- | --- | --- | --- | --- | --- | --- | --- | --- | --- | --- | --- | --- | --- | --- | --- | --- | --- | --- | --- | --- | --- | --- | --- | --- | --- | --- | --- | --- | --- | --- | --- | --- | --- | --- | --- | --- | --- | --- | --- | --- | --- | --- | --- | --- | --- | --- | --- | --- | --- | --- | --- | --- | --- | --- | --- | --- | --- | --- | --- | --- | --- | --- | --- | --- | --- | --- | --- | --- | --- | --- | --- | --- | --- | --- | --- | --- | --- | --- | --- | --- | --- | --- | --- | --- | --- | --- | --- | --- | --- | --- | --- | --- | --- | --- | --- | --- | --- | --- | --- | --- | --- | --- | --- | --- | --- | --- | --- | --- | --- | --- | --- | --- | --- | --- | --- | --- | --- | --- | --- | --- | --- | --- | --- | --- | --- | --- | --- | --- | --- | --- | --- | --- | --- | --- | --- | --- | --- | --- | --- | --- | --- | --- | --- | --- | --- | --- | --- | --- | --- | --- | --- | --- | --- | --- | --- | --- | --- | --- | --- | --- | --- | --- | --- | --- | --- | --- | --- | --- | --- | --- | --- | --- | --- | --- | --- | --- | --- | --- | --- | --- | --- | --- | --- | --- | --- | --- | --- | --- | --- | --- | --- | --- | --- | --- | --- | --- | --- | --- | --- | --- | --- | --- | --- | --- | --- | --- | --- | --- | --- | --- | --- | --- | --- | --- | --- | --- | --- | --- | --- | --- | --- | --- | --- | --- | --- | --- | --- | --- | --- | --- | --- | --- | --- | --- | --- | --- | --- | --- | --- | --- | --- | --- | --- | --- | --- | --- | --- | --- | --- | --- | --- | --- | --- | --- | --- | --- | --- | --- | --- | --- | --- | --- | --- | --- | --- | --- | --- | --- | --- | --- | --- | --- | --- | --- | --- | --- | --- | --- | --- | --- | --- | --- | --- | --- | --- | --- | --- | --- | --- | --- | --- | --- | --- | --- | --- | --- | --- | --- | --- | --- | --- | --- | --- | --- | --- | --- | --- | --- | --- | --- | --- | --- | --- | --- | --- | --- | --- | --- | --- | --- | --- | --- | --- | --- | --- | --- | --- | --- | --- | --- | --- | --- | --- | --- | --- | --- | --- | --- | --- | --- | --- | --- | --- | --- | --- | --- | --- | --- | --- | --- | --- | --- | --- | --- | --- | --- | --- | --- | --- | --- | --- | --- | --- | --- | --- | --- | --- | --- | --- | --- | --- | --- | --- | --- | --- | --- | --- | --- | --- | --- | --- | --- | --- | --- | --- | --- | --- | --- | --- | --- | --- | --- | --- | --- | --- | --- | --- | --- | --- | --- | --- | --- | --- | --- | --- | --- | --- | --- | --- | --- | --- | --- | --- | --- | --- | --- | --- | --- | --- | --- | --- | --- | --- | --- | --- | --- | --- | --- | --- | --- | --- | --- | --- | --- | --- | --- | --- | --- | --- | --- | --- | --- | --- | --- | --- | --- | --- | --- | --- | --- | --- | --- | --- | --- | --- | --- | --- | --- | --- | --- | --- | --- | --- | --- | --- | --- | --- | --- | --- | --- | --- | --- | --- | --- | --- | --- | --- | --- | --- | --- | --- | --- | --- | --- | --- | --- | --- | --- | --- | --- | --- | --- | --- | --- | --- | --- | --- | --- | --- | --- | --- | --- | --- | --- | --- | --- | --- | --- | --- | --- | --- | --- | --- | --- | --- | --- | --- | --- | --- | --- | --- | --- | --- | --- | --- | --- | --- | --- | --- | --- | --- | --- | --- | --- | --- | --- | --- | --- | --- | --- | --- | --- | --- | --- | --- | --- | --- | --- | --- | --- | --- | --- | --- | --- | --- | --- | --- | --- | --- | --- | --- | --- | --- | --- | --- | --- | --- | --- | --- | --- | --- | --- | --- | --- | --- | --- | --- | --- | --- | --- | --- | --- | --- | --- | --- | --- | --- | --- | --- | --- | --- | --- | --- | --- | --- | --- | --- | --- | --- | --- | --- | --- | --- | --- | --- | --- | --- | --- | --- | --- | --- | --- | --- | --- | --- | --- | --- | --- | --- | --- | --- | --- | --- | --- | --- | --- | --- | --- | --- | --- | --- | --- | --- | --- | --- | --- | --- | --- | --- | --- | --- | --- | --- | --- | --- | --- | --- | --- | --- | --- | --- | --- | --- | --- | --- | --- | --- | --- | --- | --- | --- | --- | --- | --- | --- | --- | --- | --- | --- | --- | --- | --- | --- | --- | --- | --- | --- | --- | --- | --- | --- | --- | --- | --- | --- | --- | --- | --- | --- | --- | --- | --- | --- | --- | --- | --- | --- | --- | --- | --- | --- | --- | --- | --- | --- | --- | --- | --- | --- | --- | --- | --- | --- | --- | --- | --- | --- | --- | --- | --- | --- | --- | --- | --- | --- | --- | --- | --- | --- | --- | --- | --- | --- | --- | --- | --- | --- | --- | --- | --- | --- | --- | --- | --- | --- | --- | --- | --- | --- | --- | --- | --- | --- | --- | --- | --- | --- | --- | --- | --- | --- | --- | --- | --- | --- | --- | --- | --- | --- | --- | --- | --- | --- | --- | --- | --- | --- | --- | --- | --- | --- | --- | --- | --- | --- | --- | --- | --- | --- | --- | --- | --- | --- | --- | --- | --- | --- | --- | --- | --- | --- | --- | --- | --- | --- | --- | --- | --- | --- | --- | --- | --- | --- | --- | --- | --- | --- | --- | --- | --- | --- | --- | --- | --- | --- | --- | --- | --- | --- | --- | --- | --- | --- | --- | --- | --- | --- | --- | --- | --- | --- | --- | --- | --- | --- | --- | --- | --- | --- | --- | --- | --- | --- | --- | --- | --- | --- | --- | --- | --- | --- | --- | --- | --- | --- | --- | --- | --- | --- | --- | --- | --- | --- | --- | --- | --- | --- | --- | --- | --- | --- | --- | --- | --- | --- | --- | --- | --- | --- | --- | --- | --- | --- | --- | --- | --- | --- | --- | --- | --- | --- | --- | --- | --- | --- | --- | --- | --- | --- | --- | --- | --- | --- | --- | --- | --- | --- | --- | --- | --- | --- | --- | --- | --- | --- | --- | --- | --- | --- | --- | --- | --- | --- | --- | --- | --- | --- | --- | --- | --- | --- | --- | --- | --- | --- | --- | --- | --- | --- | --- | --- | --- | --- | --- | --- | --- | --- | --- | --- | --- | --- | --- | --- | --- | --- | --- | --- | --- | --- | --- | --- | --- | --- | --- | --- | --- | --- | --- | --- | --- | --- | --- | --- | --- | --- | --- | --- | --- | --- | --- | --- | --- | --- | --- | --- | --- | --- | --- | --- | --- | --- | --- | --- | --- | --- | --- | --- | --- | --- | --- | --- | --- | --- | --- | --- | --- | --- | --- | --- | --- | --- | --- | --- | --- | --- | --- | --- | --- | --- | --- | --- | --- | --- | --- | --- | --- | --- | --- | --- | --- | --- | --- | --- | --- | --- | --- | --- | --- | --- | --- | --- | --- | --- | --- | --- | --- | --- | --- | --- | --- | --- | --- | --- | --- | --- | --- | --- | --- | --- | --- | --- | --- | --- | --- | --- | --- | --- | --- | --- | --- | --- | --- | --- | --- | --- | --- | --- | --- | --- | --- | --- | --- | --- | --- | --- | --- | --- | --- | --- | --- | --- | --- | --- | --- | --- | --- | --- | --- | --- | --- | --- | --- | --- | --- | --- | --- | --- | --- | --- | --- | --- | --- | --- | --- | --- | --- | --- | --- | --- | --- | --- | --- | --- | --- | --- | --- | --- | --- | --- | --- | --- | --- | --- | --- | --- | --- | --- | --- | --- | --- | --- | --- | --- | --- | --- | --- | --- | --- | --- | --- | --- | --- | --- | --- | --- | --- | --- | --- | --- | --- | --- | --- | --- | --- | --- | --- | --- | --- | --- | --- | --- | --- | --- | --- | --- | --- | --- | --- | --- | --- | --- | --- | --- | --- | --- | --- | --- | --- | --- | --- | --- | --- | --- | --- | --- | --- | --- | --- | --- | --- | --- | --- | --- | --- | --- | --- | --- | --- | --- | --- | --- | --- | --- | --- | --- | --- | --- | --- | --- | --- | --- | --- | --- | --- | --- | --- | --- | --- | --- | --- | --- | --- | --- | --- | --- | --- | --- | --- | --- | --- | --- | --- | --- | --- | --- | --- | --- | --- | --- | --- | --- | --- | --- | --- | --- | --- | --- | --- | --- | --- | --- | --- | --- | --- | --- | --- | --- | --- | --- | --- | --- | --- | --- | --- | --- | --- | --- | --- | --- | --- | --- | --- | --- | --- | --- | --- | --- | --- | --- | --- | --- | --- | --- | --- | --- | --- | --- | --- | --- | --- | --- | --- | --- | --- | --- | --- | --- | --- | --- | --- | --- | --- | --- | --- | --- | --- | --- | --- | --- | --- | --- | --- | --- | --- | --- | --- | --- | --- | --- | --- | --- | --- | --- | --- | --- | --- | --- | --- | --- | --- | --- | --- | --- | --- | --- | --- | --- | --- | --- | --- | --- | --- | --- | --- | --- | --- | --- | --- | --- | --- | --- | --- | --- | --- | --- | --- | --- | --- | --- | --- | --- | --- | --- | --- | --- | --- | --- | --- | --- | --- | --- | --- | --- | --- | --- | --- | --- | --- | --- | --- | --- | --- | --- | --- | --- | --- | --- | --- | --- | --- | --- | --- | --- | --- | --- | --- | --- | --- | --- | --- | --- | --- | --- | --- | --- | --- | --- | --- | --- | --- | --- | --- | --- | --- | --- | --- | --- | --- | --- | --- | --- | --- | --- | --- | --- | --- | --- | --- | --- | --- | --- | --- | --- | --- | --- | --- | --- | --- | --- | --- | --- | --- | --- | --- | --- | --- | --- | --- | --- | --- | --- | --- | --- | --- | --- | --- | --- | --- | --- | --- | --- | --- | --- | --- | --- | --- | --- | --- | --- | --- | --- | --- | --- | --- | --- | --- | --- | --- | --- | --- | --- | --- | --- | --- | --- | --- | --- | --- | --- | --- | --- | --- | --- | --- | --- | --- | --- | --- | --- | --- | --- | --- | --- | --- | --- | --- | --- | --- | --- | --- | --- | --- | --- | --- | --- | --- | --- | --- | --- | --- | --- | --- | --- | --- | --- | --- | --- | --- | --- | --- | --- | --- | --- | --- | --- | --- | --- | --- | --- | --- | --- | --- | --- | --- | --- | --- | --- | --- | --- | --- | --- | --- | --- | --- | --- | --- | --- | --- | --- | --- | --- | --- | --- | --- | --- | --- | --- | --- | --- | --- | --- | --- | --- | --- | --- | --- | --- | --- | --- | --- | --- | --- | --- | --- | --- | --- | --- | --- | --- | --- | --- | --- | --- | --- | --- | --- | --- | --- | --- | --- | --- | --- | --- | --- | --- | --- | --- | --- | --- | --- | --- | --- | --- | --- | --- | --- | --- | --- | --- | --- | --- | --- | --- | --- | --- | --- | --- | --- | --- | --- | --- | --- | --- | --- | --- | --- | --- | --- | --- | --- | --- | --- | --- | --- | --- | --- | --- | --- | --- | --- | --- | --- | --- | --- | --- | --- | --- | --- | --- | --- | --- | --- | --- | --- | --- | --- | --- | --- | --- | --- | --- | --- | --- | --- | --- | --- | --- | --- | --- | --- | --- | --- | --- | --- | --- | --- | --- | --- | --- | --- | --- | --- | --- | --- | --- | --- | --- | --- | --- | --- | --- | --- | --- | --- | --- | --- | --- | --- | --- | --- | --- | --- | --- | --- | --- | --- | --- | --- | --- | --- | --- | --- | --- | --- | --- | --- | --- | --- | --- | --- | --- | --- | --- | --- | --- | --- | --- | --- | --- | --- | --- | --- | --- | --- | --- | --- | --- | --- | --- | --- | --- | --- | --- | --- | --- | --- | --- | --- | --- | --- | --- | --- | --- | --- | --- | --- | --- | --- | --- | --- | --- | --- | --- | --- | --- | --- | --- | --- | --- | --- | --- | --- | --- | --- | --- | --- | --- | --- | --- | --- | --- | --- | --- | --- | --- | --- | --- | --- | --- | --- | --- | --- | --- | --- | --- | --- | --- | --- | --- | --- | --- | --- | --- | --- | --- | --- | --- | --- | --- | --- | --- | --- | --- | --- | --- | --- | --- | --- | --- | --- | --- | --- | --- | --- | --- | --- | --- | --- | --- | --- | --- | --- | --- | --- | --- | --- | --- | --- | --- | --- | --- | --- | --- | --- | --- | --- | --- | --- | --- | --- | --- | --- | --- | --- | --- | --- | --- | --- | --- | --- | --- | --- | --- | --- | --- | --- | --- | --- | --- | --- | --- | --- | --- | --- | --- | --- | --- | --- | --- | --- | --- | --- | --- | --- | --- | --- | --- | --- | --- | --- | --- | --- | --- | --- | --- | --- | --- | --- | --- | --- | --- | --- | --- | --- | --- | --- | --- | --- | --- | --- | --- | --- | --- | --- | --- | --- | --- | --- | --- | --- | --- | --- | --- | --- | --- | --- | --- | --- | --- | --- | --- | --- | --- | --- | --- | --- | --- | --- | --- | --- | --- | --- | --- | --- | --- | --- | --- | --- | --- | --- | --- | --- | --- | --- | --- | --- | --- | --- | --- | --- | --- | --- | --- | --- | --- | --- | --- | --- | --- | --- | --- | --- | --- | --- | --- | --- | --- | --- | --- | --- | --- | --- | --- | --- | --- | --- | --- | --- | --- | --- | --- | --- | --- | --- | --- | --- | --- | --- | --- | --- | --- | --- | --- | --- | --- | --- | --- | --- | --- | --- | --- | --- | --- | --- | --- | --- | --- | --- | --- | --- | --- | --- | --- | --- | --- | --- | --- | --- | --- | --- | --- | --- | --- | --- | --- | --- | --- | --- | --- | --- | --- | --- | --- | --- | --- | --- | --- | --- | --- | --- | --- | --- | --- | --- | --- | --- | --- | --- | --- | --- | --- | --- | --- | --- | --- | --- | --- | --- | --- | --- | --- | --- | --- | --- | --- | --- | --- | --- | --- | --- | --- | --- | --- | --- | --- | --- | --- | --- | --- | --- | --- | --- | --- | --- | --- | --- | --- | --- | --- | --- | --- | --- | --- | --- | --- | --- | --- | --- | --- | --- | --- | --- | --- | --- | --- | --- | --- | --- | --- | --- | --- | --- | --- | --- | --- | --- | --- | --- | --- | --- | --- | --- | --- | --- | --- | --- | --- | --- | --- | --- | --- | --- | --- | --- | --- | --- | --- | --- | --- | --- | --- | --- | --- | --- | --- | --- | --- | --- | --- | --- | --- | --- | --- | --- | --- | --- | --- | --- | --- | --- | --- | --- | --- | --- | --- | --- | --- | --- | --- | --- | --- | --- | --- | --- | --- | --- | --- | --- | --- | --- | --- | --- | --- | --- | --- | --- | --- | --- | --- | --- | --- | --- | --- | --- | --- | --- | --- | --- | --- | --- | --- | --- | --- | --- | --- | --- | --- | --- | --- | --- | --- | --- | --- | --- | --- | --- | --- | --- | --- | --- | --- | --- | --- | --- | --- | --- | --- | --- | --- | --- | --- | --- | --- | --- | --- | --- | --- | --- | --- | --- | --- | --- | --- | --- | --- | --- | --- | --- | --- | --- | --- | --- | --- | --- | --- | --- | --- | --- | --- | --- | --- | --- | --- | --- | --- | --- | --- | --- | --- | --- | --- | --- | --- | --- | --- | --- | --- | --- | --- | --- | --- | --- | --- | --- | --- | --- | --- | --- | --- | --- | --- | --- | --- | --- | --- | --- | --- | --- | --- | --- | --- | --- | --- | --- | --- | --- | --- | --- | --- | --- | --- | --- | --- | --- | --- | --- | --- | --- | --- | --- | --- | --- | --- | --- | --- | --- | --- | --- | --- | --- | --- | --- | --- | --- | --- | --- | --- | --- | --- | --- | --- | --- | --- | --- | --- | --- | --- | --- | --- | --- | --- | --- | --- | --- | --- | --- | --- | --- | --- | --- | --- | --- | --- | --- | --- | --- | --- | --- | --- | --- | --- | --- | --- | --- | --- | --- | --- | --- | --- | --- | --- | --- | --- | --- | --- | --- | --- | --- | --- | --- | --- | --- | --- | --- | --- | --- | --- | --- | --- | --- | --- | --- | --- | --- | --- | --- | --- | --- | --- | --- | --- | --- | --- | --- | --- | --- | --- | --- | --- | --- | --- | --- | --- | --- | --- | --- | --- | --- | --- | --- | --- | --- | --- | --- | --- | --- | --- | --- | --- | --- | --- | --- | --- | --- | --- | --- | --- | --- | --- | --- | --- | --- | --- | --- | --- | --- | --- | --- | --- | --- | --- | --- | --- | --- | --- | --- | --- | --- | --- | --- | --- | --- | --- | --- | --- | --- | --- | --- | --- | --- | --- | --- | --- | --- | --- | --- | --- | --- | --- | --- | --- | --- | --- | --- | --- | --- | --- | --- | --- | --- | --- | --- | --- | --- | --- | --- | --- | --- | --- | --- | --- | --- | --- | --- | --- | --- | --- | --- | --- | --- | --- | --- | --- | --- | --- | --- | --- | --- | --- | --- | --- | --- | --- | --- | --- | --- | --- | --- | --- | --- | --- | --- | --- | --- | --- | --- | --- | --- | --- | --- | --- | --- | --- | --- | --- | --- | --- | --- | --- | --- | --- | --- | --- | --- | --- | --- | --- | --- | --- | --- | --- | --- | --- | --- | --- | --- | --- | --- | --- | --- | --- | --- | --- | --- | --- | --- | --- | --- | --- | --- | --- | --- | --- | --- | --- | --- | --- | --- | --- | --- | --- | --- | --- | --- | --- | --- | --- | --- | --- | --- | --- | --- | --- | --- | --- | --- | --- | --- | --- | --- | --- | --- | --- | --- | --- | --- | --- | --- | --- | --- | --- | --- | --- | --- | --- | --- | --- | --- | --- | --- | --- | --- | --- | --- | --- | --- | --- | --- | --- | --- | --- | --- | --- | --- | --- | --- | --- | --- | --- | --- | --- | --- | --- | --- | --- | --- | --- | --- | --- | --- | --- | --- | --- | --- | --- | --- | --- | --- | --- | --- | --- | --- | --- | --- | --- | --- | --- | --- | --- | --- | --- | --- | --- | --- | --- | --- | --- | --- | --- | --- | --- | --- | --- | --- | --- | --- | --- | --- | --- | --- | --- | --- | --- | --- | --- | --- | --- | --- | --- | --- | --- | --- | --- | --- | --- | --- | --- | --- | --- | --- | --- | --- | --- | --- | --- | --- | --- | --- | --- | --- | --- | --- | --- | --- | --- | --- | --- | --- | --- | --- | --- | --- | --- | --- | --- | --- | --- | --- | --- | --- | --- | --- | --- | --- | --- | --- | --- | --- | --- | --- | --- | --- | --- | --- | --- | --- | --- | --- | --- | --- | --- | --- | --- | --- | --- | --- | --- | --- | --- | --- | --- | --- | --- | --- | --- | --- | --- | --- | --- | --- | --- | --- | --- | --- | --- | --- | --- | --- | --- | --- | --- | --- | --- | --- | --- | --- | --- | --- | --- | --- | --- | --- | --- | --- | --- | --- | --- | --- | --- | --- | --- | --- | --- | --- | --- | --- | --- | --- | --- | --- | --- | --- | --- | --- | --- | --- | --- | --- | --- | --- | --- | --- | --- | --- | --- | --- | --- | --- | --- | --- | --- | --- | --- | --- | --- | --- | --- | --- | --- | --- | --- | --- | --- | --- | --- | --- | --- | --- | --- | --- | --- | --- | --- | --- | --- | --- | --- | --- | --- | --- | --- | --- | --- | --- | --- | --- | --- | --- | --- | --- | --- | --- | --- | --- | --- | --- | --- | --- | --- | --- | --- | --- | --- | --- | --- | --- | --- | --- | --- | --- | --- | --- | --- | --- | --- | --- | --- | --- | --- | --- | --- | --- | --- | --- | --- | --- | --- | --- | --- | --- | --- | --- | --- | --- | --- | --- | --- | --- | --- | --- | --- | --- | --- | --- | --- | --- | --- | --- | --- | --- | --- | --- | --- | --- | --- | --- | --- | --- | --- | --- | --- | --- | --- | --- | --- | --- | --- | --- | --- | --- | --- | --- | --- | --- | --- | --- | --- | --- | --- | --- | --- | --- | --- | --- | --- | --- | --- | --- | --- | --- | --- | --- | --- | --- | --- | --- | --- | --- | --- | --- | --- | --- | --- | --- | --- | --- | --- | --- | --- | --- | --- | --- | --- | --- | --- | --- | --- | --- | --- | --- | --- | --- | --- | --- | --- | --- | --- | --- | --- | --- | --- | --- | --- | --- | --- | --- | --- | --- | --- | --- | --- | --- | --- | --- | --- | --- | --- | --- | --- | --- | --- | --- | --- | --- | --- | --- | --- | --- | --- | --- | --- | --- | --- | --- | --- | --- | --- | --- | --- | --- | --- | --- | --- | --- | --- | --- | --- | --- | --- | --- | --- | --- | --- | --- | --- | --- | --- | --- | --- | --- | --- | --- | --- | --- | --- | --- | --- | --- | --- | --- | --- | --- | --- | --- | --- | --- | --- | --- | --- | --- | --- | --- | --- | --- | --- | --- | --- | --- | --- | --- | --- | --- | --- | --- | --- | --- | --- | --- | --- | --- | --- | --- | --- | --- | --- | --- | --- | --- | --- | --- | --- | --- | --- | --- | --- | --- | --- | --- | --- | --- | --- | --- | --- | --- | --- | --- | --- | --- | --- | --- | --- | --- | --- | --- | --- | --- | --- | --- | --- | --- | --- | --- | --- | --- | --- | --- | --- | --- | --- | --- | --- | --- | --- | --- | --- | --- | --- | --- | --- | --- | --- | --- | --- | --- | --- | --- | --- | --- | --- | --- | --- | --- | --- | --- | --- | --- | --- | --- | --- | --- | --- | --- | --- | --- | --- | --- | --- | --- | --- | --- | --- | --- | --- | --- | --- | --- | --- | --- | --- | --- | --- | --- | --- | --- | --- | --- | --- | --- | --- | --- | --- | --- | --- | --- | --- | --- | --- | --- | --- | --- | --- | --- | --- | --- | --- | --- | --- | --- | --- | --- | --- | --- | --- | --- | --- | --- | --- | --- | --- | --- | --- | --- | --- | --- | --- | --- | --- | --- | --- | --- | --- | --- | --- | --- | --- | --- | --- | --- | --- | --- | --- | --- | --- | --- | --- | --- | --- | --- | --- | --- | --- | --- | --- | --- | --- | --- | | DAVID Functional Annotation Clustering      |  | | --- | | Functional Annotation Clustering | | Help and Manual | | Current Gene List: DavidMay18\_TPM88 | | Current Background: DavidMay18\_TPM8\_bg | | 1361 DAVID IDs |  - Options          Classification Stringency    Custom   Lowest   Low   Medium   High   Highest   - |  |  |  |     | --- | --- | --- |     | Kappa Similarity | Similarity Term Overlap 3 4 5 6 7 8 9 10 | Similarity Threshold 0.20 0.25 0.30 0.35 0.40 0.45 0.50 0.55 0.60 0.65 0.70 0.75 0.80 0.85 0.90 0.95 1.00 |     | Classification | Initial Group Membership 2 3 4 5 6 7 8 9 10 | Final Group Membership 2 3 4 5 6 7 8 9 10 | Multiple Linkage Threshold 0.00 0.05 0.10 0.15 0.20 0.25 0.30 0.35 0.40 0.45 0.50 0.55 0.60 0.65 0.70 0.75 0.80 0.85 0.90 0.95 1.00 |  |     | Enrichment Thresholds | EASE |  |  |  |     |     | Display | Fold Change | Bonferroni | Benjamini | FDR | LT,PH,PT |  |  |  |  | | --- | --- | --- | |  |  |  |     |  |  | | --- | --- | | 181 Cluster(s) | Download File |  | Annotation Cluster 1 | | Enrichment Score: 37.97 |  |  | Count | P\_Value | Benjamini | | --- | --- | --- | --- | --- | --- | --- | --- | |  | GOTERM\_CC\_5 | plasma membrane part | **RT** |  | 207 | 2.2E-39 | 1.1E-36 | |  | GOTERM\_CC\_5 | intrinsic component of plasma membrane | **RT** |  | 151 | 1.1E-38 | 2.5E-36 | |  | GOTERM\_CC\_5 | integral component of plasma membrane | **RT** |  | 147 | 5.2E-38 | 8.1E-36 | | Annotation Cluster 2 | | Enrichment Score: 11.93 |  |  | Count | P\_Value | Benjamini | | --- | --- | --- | --- | --- | --- | --- | --- | |  | GOTERM\_BP\_5 | G-protein coupled receptor signaling pathway | **RT** |  | 78 | 5.9E-16 | 1.3E-12 | |  | GOTERM\_MF\_5 | G-protein coupled receptor activity | **RT** |  | 48 | 3.1E-11 | 2.9E-9 | |  | GOTERM\_BP\_5 | G-protein coupled receptor signaling pathway, coupled to cyclic nucleotide second messenger | **RT** |  | 28 | 8.6E-11 | 4.1E-8 | | Annotation Cluster 3 | | Enrichment Score: 8.16 |  |  | Count | P\_Value | Benjamini | | --- | --- | --- | --- | --- | --- | --- | --- | |  | GOTERM\_BP\_5 | modulation of synaptic transmission | **RT** |  | 49 | 1.2E-11 | 1.4E-8 | |  | GOTERM\_BP\_5 | synaptic signaling | **RT** |  | 102 | 1.3E-10 | 3.9E-8 | |  | GOTERM\_BP\_5 | trans-synaptic signaling | **RT** |  | 102 | 1.3E-10 | 3.9E-8 | |  | GOTERM\_BP\_5 | neurotransmitter transport | **RT** |  | 40 | 1.1E-2 | 2.2E-1 | | Annotation Cluster 4 | | Enrichment Score: 7.31 |  |  | Count | P\_Value | Benjamini | | --- | --- | --- | --- | --- | --- | --- | --- | |  | GOTERM\_BP\_5 | axon guidance | **RT** |  | 95 | 1.2E-11 | 9.6E-9 | |  | GOTERM\_BP\_5 | neuron differentiation | **RT** |  | 218 | 1.7E-11 | 1.0E-8 | |  | GOTERM\_BP\_5 | neuron projection guidance | **RT** |  | 95 | 1.0E-10 | 4.0E-8 | |  | GOTERM\_BP\_5 | neuron development | **RT** |  | 196 | 1.0E-10 | 3.5E-8 | |  | GOTERM\_BP\_5 | axon development | **RT** |  | 110 | 2.1E-10 | 5.0E-8 | |  | GOTERM\_BP\_5 | cell morphogenesis involved in neuron differentiation | **RT** |  | 138 | 5.9E-9 | 1.3E-6 | |  | GOTERM\_BP\_5 | neuron projection development | **RT** |  | 162 | 1.3E-8 | 2.7E-6 | |  | GOTERM\_BP\_5 | cell projection morphogenesis | **RT** |  | 157 | 4.4E-8 | 8.2E-6 | |  | GOTERM\_BP\_5 | neuron projection morphogenesis | **RT** |  | 153 | 4.5E-8 | 7.6E-6 | |  | GOTERM\_BP\_5 | cell part morphogenesis | **RT** |  | 158 | 1.1E-7 | 1.5E-5 | |  | GOTERM\_BP\_5 | cell morphogenesis involved in differentiation | **RT** |  | 142 | 1.1E-7 | 1.5E-5 | |  | GOTERM\_BP\_5 | cell morphogenesis | **RT** |  | 182 | 1.6E-6 | 1.6E-4 | |  | GOTERM\_BP\_5 | nervous system development | **RT** |  | 315 | 5.5E-6 | 4.7E-4 | |  | GOTERM\_BP\_5 | cell development | **RT** |  | 290 | 4.6E-5 | 2.8E-3 | |  | GOTERM\_BP\_5 | neurogenesis | **RT** |  | 265 | 1.4E-4 | 6.8E-3 | |  | GOTERM\_BP\_5 | dendrite development | **RT** |  | 56 | 2.3E-2 | 3.4E-1 | | Annotation Cluster 5 | | Enrichment Score: 6.55 |  |  | Count | P\_Value | Benjamini | | --- | --- | --- | --- | --- | --- | --- | --- | |  | GOTERM\_CC\_5 | ion channel complex | **RT** |  | 39 | 6.9E-14 | 8.2E-12 | |  | GOTERM\_MF\_5 | substrate-specific channel activity | **RT** |  | 57 | 2.0E-12 | 5.8E-10 | |  | GOTERM\_MF\_5 | ion channel activity | **RT** |  | 57 | 2.0E-12 | 5.8E-10 | |  | GOTERM\_CC\_5 | transmembrane transporter complex | **RT** |  | 42 | 5.4E-12 | 5.1E-10 | |  | GOTERM\_MF\_5 | gated channel activity | **RT** |  | 46 | 2.3E-11 | 3.3E-9 | |  | GOTERM\_CC\_5 | cation channel complex | **RT** |  | 23 | 2.1E-8 | 1.7E-6 | |  | GOTERM\_BP\_5 | ion transport | **RT** |  | 93 | 9.2E-8 | 1.4E-5 | |  | GOTERM\_CC\_5 | plasma membrane protein complex | **RT** |  | 45 | 1.0E-6 | 4.3E-5 | |  | GOTERM\_MF\_5 | ligand-gated ion channel activity | **RT** |  | 25 | 2.1E-6 | 1.2E-4 | |  | GOTERM\_BP\_5 | cation transport | **RT** |  | 69 | 2.4E-6 | 2.3E-4 | |  | GOTERM\_MF\_5 | cation transmembrane transporter activity | **RT** |  | 67 | 5.9E-5 | 2.8E-3 | |  | GOTERM\_BP\_5 | transmembrane transport | **RT** |  | 74 | 1.6E-2 | 2.7E-1 | |  | GOTERM\_BP\_5 | ion transmembrane transport | **RT** |  | 29 | 1.5E-1 | 8.3E-1 | |  | GOTERM\_BP\_5 | cation transmembrane transport | **RT** |  | 26 | 2.0E-1 | 8.8E-1 | |  | GOTERM\_BP\_5 | inorganic ion transmembrane transport | **RT** |  | 22 | 5.2E-1 | 9.9E-1 | | Annotation Cluster 6 | | Enrichment Score: 6.04 |  |  | Count | P\_Value | Benjamini | | --- | --- | --- | --- | --- | --- | --- | --- | |  | GOTERM\_CC\_5 | plasma membrane region | **RT** |  | 53 | 5.6E-8 | 3.7E-6 | |  | GOTERM\_CC\_5 | synaptic membrane | **RT** |  | 27 | 1.6E-7 | 8.6E-6 | |  | GOTERM\_CC\_5 | postsynaptic membrane | **RT** |  | 22 | 7.8E-7 | 3.7E-5 | |  | GOTERM\_MF\_5 | ligand-gated ion channel activity | **RT** |  | 25 | 2.1E-6 | 1.2E-4 | |  | GOTERM\_CC\_5 | plasma membrane receptor complex | **RT** |  | 16 | 4.3E-5 | 1.5E-3 | | Annotation Cluster 7 | | Enrichment Score: 5.17 |  |  | Count | P\_Value | Benjamini | | --- | --- | --- | --- | --- | --- | --- | --- | |  | GOTERM\_BP\_5 | organ morphogenesis | **RT** |  | 196 | 5.9E-8 | 9.4E-6 | |  | GOTERM\_BP\_5 | sensory organ development | **RT** |  | 120 | 3.4E-7 | 4.3E-5 | |  | GOTERM\_BP\_5 | eye development | **RT** |  | 97 | 2.8E-6 | 2.5E-4 | |  | GOTERM\_BP\_5 | compound eye development | **RT** |  | 90 | 2.2E-5 | 1.7E-3 | |  | GOTERM\_BP\_5 | sensory organ morphogenesis | **RT** |  | 78 | 2.2E-5 | 1.7E-3 | |  | GOTERM\_BP\_5 | eye morphogenesis | **RT** |  | 78 | 2.2E-5 | 1.7E-3 | |  | GOTERM\_BP\_5 | photoreceptor cell development | **RT** |  | 33 | 1.1E-3 | 3.5E-2 | | Annotation Cluster 8 | | Enrichment Score: 4.82 |  |  | Count | P\_Value | Benjamini | | --- | --- | --- | --- | --- | --- | --- | --- | |  | GOTERM\_MF\_5 | G-protein coupled receptor activity | **RT** |  | 48 | 3.1E-11 | 2.9E-9 | |  | GOTERM\_BP\_5 | neuropeptide signaling pathway | **RT** |  | 19 | 6.7E-4 | 2.4E-2 | |  | GOTERM\_MF\_5 | G-protein coupled peptide receptor activity | **RT** |  | 19 | 1.4E-3 | 3.9E-2 | |  | GOTERM\_MF\_5 | neuropeptide receptor activity | **RT** |  | 18 | 1.9E-3 | 4.7E-2 | | Annotation Cluster 9 | | Enrichment Score: 4.42 |  |  | Count | P\_Value | Benjamini | | --- | --- | --- | --- | --- | --- | --- | --- | |  | GOTERM\_MF\_5 | G-protein coupled amine receptor activity | **RT** |  | 16 | 1.6E-7 | 1.2E-5 | |  | GOTERM\_MF\_5 | G-protein coupled serotonin receptor activity | **RT** |  | 9 | 1.7E-4 | 5.9E-3 | |  | GOTERM\_MF\_5 | serotonin receptor activity | **RT** |  | 9 | 1.7E-4 | 5.9E-3 | |  | GOTERM\_BP\_5 | serotonin receptor signaling pathway | **RT** |  | 8 | 4.8E-4 | 1.9E-2 | | Annotation Cluster 10 | | Enrichment Score: 4 |  |  | Count | P\_Value | Benjamini | | --- | --- | --- | --- | --- | --- | --- | --- | |  | GOTERM\_BP\_5 | organ morphogenesis | **RT** |  | 196 | 5.9E-8 | 9.4E-6 | |  | GOTERM\_BP\_5 | imaginal disc-derived appendage development | **RT** |  | 98 | 1.6E-5 | 1.3E-3 | |  | GOTERM\_BP\_5 | post-embryonic organ development | **RT** |  | 119 | 3.2E-5 | 2.3E-3 | |  | GOTERM\_BP\_5 | imaginal disc-derived appendage morphogenesis | **RT** |  | 95 | 4.4E-5 | 2.8E-3 | |  | GOTERM\_BP\_5 | appendage morphogenesis | **RT** |  | 95 | 4.4E-5 | 2.8E-3 | |  | GOTERM\_BP\_5 | animal organ development | **RT** |  | 258 | 4.9E-5 | 2.9E-3 | |  | GOTERM\_BP\_5 | post-embryonic appendage morphogenesis | **RT** |  | 93 | 5.8E-5 | 3.4E-3 | |  | GOTERM\_BP\_5 | imaginal disc-derived wing morphogenesis | **RT** |  | 86 | 6.5E-5 | 3.7E-3 | |  | GOTERM\_BP\_5 | post-embryonic organ morphogenesis | **RT** |  | 105 | 7.5E-5 | 4.2E-3 | |  | GOTERM\_BP\_5 | imaginal disc morphogenesis | **RT** |  | 105 | 7.5E-5 | 4.2E-3 | |  | GOTERM\_BP\_5 | morphogenesis of an epithelium | **RT** |  | 136 | 1.1E-4 | 5.9E-3 | |  | GOTERM\_BP\_5 | wing disc morphogenesis | **RT** |  | 87 | 1.3E-4 | 6.5E-3 | |  | GOTERM\_BP\_5 | imaginal disc development | **RT** |  | 136 | 2.1E-4 | 9.7E-3 | |  | GOTERM\_BP\_5 | epithelial tube morphogenesis | **RT** |  | 113 | 2.2E-4 | 1.0E-2 | |  | GOTERM\_BP\_5 | instar larval or pupal morphogenesis | **RT** |  | 119 | 6.0E-4 | 2.2E-2 | |  | GOTERM\_BP\_5 | wing disc development | **RT** |  | 106 | 8.2E-4 | 2.8E-2 | |  | GOTERM\_BP\_5 | tube morphogenesis | **RT** |  | 115 | 8.5E-4 | 2.8E-2 | |  | GOTERM\_BP\_5 | post-embryonic morphogenesis | **RT** |  | 119 | 1.4E-3 | 4.2E-2 | |  | GOTERM\_BP\_5 | instar larval or pupal development | **RT** |  | 128 | 3.8E-3 | 1.0E-1 | | Annotation Cluster 11 | | Enrichment Score: 3.73 |  |  | Count | P\_Value | Benjamini | | --- | --- | --- | --- | --- | --- | --- | --- | |  | GOTERM\_BP\_5 | male mating behavior | **RT** |  | 28 | 7.6E-5 | 4.1E-3 | |  | GOTERM\_BP\_5 | male courtship behavior | **RT** |  | 26 | 1.4E-4 | 6.8E-3 | |  | GOTERM\_BP\_5 | courtship behavior | **RT** |  | 29 | 1.9E-4 | 9.0E-3 | |  | GOTERM\_BP\_5 | mating behavior | **RT** |  | 34 | 2.5E-4 | 1.1E-2 | |  | GOTERM\_BP\_5 | mating | **RT** |  | 34 | 4.6E-4 | 1.9E-2 | | Annotation Cluster 12 | | Enrichment Score: 3.12 |  |  | Count | P\_Value | Benjamini | | --- | --- | --- | --- | --- | --- | --- | --- | |  | GOTERM\_CC\_5 | axon | **RT** |  | 56 | 1.5E-7 | 8.9E-6 | |  | GOTERM\_CC\_5 | axon terminus | **RT** |  | 22 | 1.3E-3 | 3.5E-2 | |  | GOTERM\_CC\_5 | neuron projection terminus | **RT** |  | 22 | 1.3E-3 | 3.5E-2 | |  | GOTERM\_CC\_5 | terminal bouton | **RT** |  | 20 | 3.5E-3 | 8.9E-2 | |  | GOTERM\_CC\_5 | axon part | **RT** |  | 27 | 3.8E-3 | 8.9E-2 | |  | GOTERM\_CC\_5 | type I terminal bouton | **RT** |  | 8 | 5.7E-2 | 5.1E-1 | | Annotation Cluster 13 | | Enrichment Score: 2.94 |  |  | Count | P\_Value | Benjamini | | --- | --- | --- | --- | --- | --- | --- | --- | |  | GOTERM\_BP\_5 | negative regulation of cell communication | **RT** |  | 79 | 4.0E-5 | 2.7E-3 | |  | GOTERM\_BP\_5 | cell surface receptor signaling pathway | **RT** |  | 129 | 8.2E-4 | 2.8E-2 | |  | GOTERM\_BP\_5 | negative regulation of signal transduction | **RT** |  | 68 | 1.9E-3 | 5.4E-2 | |  | GOTERM\_BP\_5 | regulation of signal transduction | **RT** |  | 145 | 2.7E-2 | 3.7E-1 | | Annotation Cluster 14 | | Enrichment Score: 2.92 |  |  | Count | P\_Value | Benjamini | | --- | --- | --- | --- | --- | --- | --- | --- | |  | GOTERM\_BP\_5 | learning or memory | **RT** |  | 53 | 4.8E-4 | 1.9E-2 | |  | GOTERM\_BP\_5 | associative learning | **RT** |  | 26 | 1.3E-3 | 3.9E-2 | |  | GOTERM\_BP\_5 | olfactory learning | **RT** |  | 23 | 2.9E-3 | 8.0E-2 | | Annotation Cluster 15 | | Enrichment Score: 2.56 |  |  | Count | P\_Value | Benjamini | | --- | --- | --- | --- | --- | --- | --- | --- | |  | GOTERM\_BP\_5 | detection of stimulus involved in sensory perception | **RT** |  | 26 | 3.1E-4 | 1.3E-2 | |  | GOTERM\_BP\_5 | detection of temperature stimulus involved in sensory perception of pain | **RT** |  | 16 | 5.7E-3 | 1.4E-1 | |  | GOTERM\_BP\_5 | detection of temperature stimulus involved in sensory perception | **RT** |  | 16 | 5.7E-3 | 1.4E-1 | |  | GOTERM\_BP\_5 | sensory perception of temperature stimulus | **RT** |  | 16 | 5.7E-3 | 1.4E-1 | | Annotation Cluster 16 | | Enrichment Score: 2.41 |  |  | Count | P\_Value | Benjamini | | --- | --- | --- | --- | --- | --- | --- | --- | |  | GOTERM\_MF\_5 | sequence-specific DNA binding | **RT** |  | 62 | 3.6E-4 | 1.1E-2 | |  | GOTERM\_MF\_5 | regulatory region DNA binding | **RT** |  | 37 | 6.2E-3 | 1.2E-1 | |  | GOTERM\_MF\_5 | double-stranded DNA binding | **RT** |  | 28 | 2.6E-2 | 3.4E-1 | | Annotation Cluster 17 | | Enrichment Score: 2.34 |  |  | Count | P\_Value | Benjamini | | --- | --- | --- | --- | --- | --- | --- | --- | |  | GOTERM\_BP\_5 | regulation of nervous system development | **RT** |  | 84 | 2.4E-4 | 1.1E-2 | |  | GOTERM\_BP\_5 | regulation of neuron differentiation | **RT** |  | 45 | 2.1E-3 | 6.0E-2 | |  | GOTERM\_BP\_5 | regulation of neurogenesis | **RT** |  | 55 | 2.5E-3 | 6.8E-2 | |  | GOTERM\_BP\_5 | regulation of axonogenesis | **RT** |  | 17 | 6.1E-3 | 1.4E-1 | |  | GOTERM\_BP\_5 | regulation of cell morphogenesis involved in differentiation | **RT** |  | 27 | 1.1E-2 | 2.1E-1 | |  | GOTERM\_BP\_5 | regulation of neuron projection development | **RT** |  | 31 | 1.2E-2 | 2.2E-1 | |  | GOTERM\_BP\_5 | regulation of cell morphogenesis | **RT** |  | 48 | 4.5E-2 | 4.9E-1 | | Annotation Cluster 18 | | Enrichment Score: 2.32 |  |  | Count | P\_Value | Benjamini | | --- | --- | --- | --- | --- | --- | --- | --- | |  | GOTERM\_BP\_5 | stem cell differentiation | **RT** |  | 20 | 5.0E-4 | 1.9E-2 | |  | GOTERM\_BP\_5 | stem cell fate determination | **RT** |  | 8 | 9.2E-3 | 1.9E-1 | |  | GOTERM\_BP\_5 | stem cell fate commitment | **RT** |  | 10 | 2.4E-2 | 3.4E-1 | | Annotation Cluster 19 | | Enrichment Score: 2.06 |  |  | Count | P\_Value | Benjamini | | --- | --- | --- | --- | --- | --- | --- | --- | |  | GOTERM\_BP\_5 | intracellular signal transduction | **RT** |  | 148 | 2.0E-4 | 9.5E-3 | |  | GOTERM\_BP\_5 | small GTPase mediated signal transduction | **RT** |  | 64 | 2.3E-3 | 6.3E-2 | |  | GOTERM\_BP\_5 | regulation of small GTPase mediated signal transduction | **RT** |  | 36 | 2.2E-2 | 3.3E-1 | |  | GOTERM\_BP\_5 | regulation of intracellular signal transduction | **RT** |  | 63 | 5.7E-1 | 1.0E0 | | Annotation Cluster 20 | | Enrichment Score: 1.99 |  |  | Count | P\_Value | Benjamini | | --- | --- | --- | --- | --- | --- | --- | --- | |  | GOTERM\_BP\_5 | establishment of ommatidial planar polarity | **RT** |  | 20 | 1.8E-3 | 5.1E-2 | |  | GOTERM\_BP\_5 | establishment of planar polarity | **RT** |  | 25 | 8.9E-3 | 1.9E-1 | |  | GOTERM\_BP\_5 | morphogenesis of a polarized epithelium | **RT** |  | 27 | 1.3E-2 | 2.5E-1 | |  | GOTERM\_BP\_5 | ommatidial rotation | **RT** |  | 10 | 5.0E-2 | 5.2E-1 | | Annotation Cluster 21 | | Enrichment Score: 1.96 |  |  | Count | P\_Value | Benjamini | | --- | --- | --- | --- | --- | --- | --- | --- | |  | GOTERM\_BP\_5 | synapse assembly | **RT** |  | 60 | 4.2E-5 | 2.8E-3 | |  | GOTERM\_BP\_5 | regulation of nervous system development | **RT** |  | 84 | 2.4E-4 | 1.1E-2 | |  | GOTERM\_BP\_5 | synaptic growth at neuromuscular junction | **RT** |  | 37 | 6.3E-3 | 1.4E-1 | |  | GOTERM\_BP\_5 | regulation of synapse assembly | **RT** |  | 37 | 6.3E-3 | 1.4E-1 | |  | GOTERM\_BP\_5 | regulation of neuromuscular junction development | **RT** |  | 35 | 1.4E-2 | 2.5E-1 | |  | GOTERM\_BP\_5 | negative regulation of nervous system development | **RT** |  | 32 | 1.9E-2 | 3.0E-1 | |  | GOTERM\_BP\_5 | regulation of synaptic growth at neuromuscular junction | **RT** |  | 33 | 2.6E-2 | 3.6E-1 | |  | GOTERM\_BP\_5 | negative regulation of developmental growth | **RT** |  | 23 | 3.5E-2 | 4.3E-1 | |  | GOTERM\_BP\_5 | negative regulation of neuromuscular junction development | **RT** |  | 14 | 1.4E-1 | 8.0E-1 | |  | GOTERM\_BP\_5 | negative regulation of synapse assembly | **RT** |  | 14 | 1.4E-1 | 8.0E-1 | |  | GOTERM\_BP\_5 | negative regulation of synaptic growth at neuromuscular junction | **RT** |  | 14 | 1.4E-1 | 8.0E-1 | | Annotation Cluster 22 | | Enrichment Score: 1.84 |  |  | Count | P\_Value | Benjamini | | --- | --- | --- | --- | --- | --- | --- | --- | |  | GOTERM\_BP\_5 | cell fate commitment | **RT** |  | 93 | 3.5E-4 | 1.4E-2 | |  | GOTERM\_BP\_5 | cell-cell signaling involved in cell fate commitment | **RT** |  | 38 | 9.5E-2 | 7.1E-1 | |  | GOTERM\_BP\_5 | lateral inhibition | **RT** |  | 38 | 9.5E-2 | 7.1E-1 | | Annotation Cluster 23 | | Enrichment Score: 1.78 |  |  | Count | P\_Value | Benjamini | | --- | --- | --- | --- | --- | --- | --- | --- | |  | GOTERM\_BP\_5 | detection of light stimulus | **RT** |  | 22 | 9.8E-4 | 3.2E-2 | |  | GOTERM\_BP\_5 | phototransduction | **RT** |  | 20 | 1.2E-3 | 3.8E-2 | |  | GOTERM\_BP\_5 | detection of visible light | **RT** |  | 12 | 5.2E-3 | 1.3E-1 | |  | GOTERM\_BP\_5 | phototransduction, visible light | **RT** |  | 9 | 1.3E-2 | 2.4E-1 | |  | GOTERM\_BP\_5 | cellular response to light stimulus | **RT** |  | 12 | 1.9E-2 | 3.0E-1 | |  | GOTERM\_BP\_5 | rhodopsin mediated signaling pathway | **RT** |  | 8 | 2.9E-2 | 3.8E-1 | |  | GOTERM\_BP\_5 | deactivation of rhodopsin mediated signaling | **RT** |  | 7 | 4.0E-2 | 4.7E-1 | |  | GOTERM\_BP\_5 | regulation of rhodopsin mediated signaling pathway | **RT** |  | 7 | 6.3E-2 | 5.9E-1 | |  | GOTERM\_BP\_5 | regulation of G-protein coupled receptor protein signaling pathway | **RT** |  | 8 | 9.3E-2 | 7.0E-1 | |  | GOTERM\_BP\_5 | metarhodopsin inactivation | **RT** |  | 3 | 1.7E-1 | 8.4E-1 | | Annotation Cluster 24 | | Enrichment Score: 1.74 |  |  | Count | P\_Value | Benjamini | | --- | --- | --- | --- | --- | --- | --- | --- | |  | GOTERM\_BP\_5 | regulation of neurotransmitter secretion | **RT** |  | 16 | 1.9E-6 | 1.9E-4 | |  | GOTERM\_BP\_5 | regulation of neurotransmitter transport | **RT** |  | 16 | 1.9E-6 | 1.9E-4 | |  | GOTERM\_BP\_5 | regulation of secretion | **RT** |  | 33 | 1.2E-5 | 9.7E-4 | |  | GOTERM\_BP\_5 | regulation of secretion by cell | **RT** |  | 31 | 3.2E-5 | 2.3E-3 | |  | GOTERM\_BP\_5 | neurotransmitter transport | **RT** |  | 40 | 1.1E-2 | 2.2E-1 | |  | GOTERM\_BP\_5 | signal release | **RT** |  | 42 | 1.7E-2 | 2.7E-1 | |  | GOTERM\_BP\_5 | secretion | **RT** |  | 58 | 2.4E-2 | 3.4E-1 | |  | GOTERM\_BP\_5 | secretion by cell | **RT** |  | 56 | 2.9E-2 | 3.8E-1 | |  | GOTERM\_CC\_5 | secretory vesicle | **RT** |  | 24 | 3.0E-2 | 3.9E-1 | |  | GOTERM\_CC\_5 | exocytic vesicle | **RT** |  | 22 | 3.1E-2 | 3.9E-1 | |  | GOTERM\_CC\_5 | synaptic vesicle | **RT** |  | 21 | 3.4E-2 | 4.0E-1 | |  | GOTERM\_CC\_5 | transport vesicle | **RT** |  | 26 | 4.7E-2 | 4.6E-1 | |  | GOTERM\_BP\_5 | neurotransmitter secretion | **RT** |  | 33 | 5.4E-2 | 5.4E-1 | |  | GOTERM\_BP\_5 | signal release from synapse | **RT** |  | 33 | 5.4E-2 | 5.4E-1 | |  | GOTERM\_BP\_5 | synaptic vesicle exocytosis | **RT** |  | 19 | 8.7E-2 | 6.9E-1 | |  | GOTERM\_BP\_5 | synaptic vesicle localization | **RT** |  | 35 | 1.0E-1 | 7.2E-1 | |  | GOTERM\_BP\_5 | exocytosis | **RT** |  | 26 | 1.2E-1 | 7.7E-1 | |  | GOTERM\_BP\_5 | regulated exocytosis | **RT** |  | 19 | 1.2E-1 | 7.7E-1 | |  | GOTERM\_CC\_5 | cytoplasmic, membrane-bounded vesicle | **RT** |  | 35 | 1.3E-1 | 7.5E-1 | |  | GOTERM\_BP\_5 | establishment of synaptic vesicle localization | **RT** |  | 33 | 1.5E-1 | 8.3E-1 | |  | GOTERM\_BP\_5 | synaptic vesicle transport | **RT** |  | 33 | 1.5E-1 | 8.3E-1 | |  | GOTERM\_CC\_5 | intracellular vesicle | **RT** |  | 36 | 1.8E-1 | 8.4E-1 | |  | GOTERM\_CC\_5 | cytoplasmic vesicle | **RT** |  | 36 | 1.8E-1 | 8.4E-1 | |  | GOTERM\_BP\_5 | vesicle mediated transport in synapse | **RT** |  | 29 | 2.4E-1 | 9.2E-1 | |  | GOTERM\_BP\_5 | establishment of vesicle localization | **RT** |  | 33 | 2.7E-1 | 9.4E-1 | |  | GOTERM\_BP\_5 | synaptic vesicle recycling | **RT** |  | 13 | 6.7E-1 | 1.0E0 | | Annotation Cluster 25 | | Enrichment Score: 1.68 |  |  | Count | P\_Value | Benjamini | | --- | --- | --- | --- | --- | --- | --- | --- | |  | GOTERM\_CC\_5 | acetylcholine-gated channel complex | **RT** |  | 7 | 6.8E-3 | 1.4E-1 | |  | GOTERM\_BP\_5 | response to alkaloid | **RT** |  | 12 | 1.9E-2 | 3.0E-1 | |  | GOTERM\_BP\_5 | response to nicotine | **RT** |  | 6 | 3.2E-2 | 4.0E-1 | |  | GOTERM\_MF\_5 | acetylcholine receptor activity | **RT** |  | 5 | 4.6E-2 | 4.6E-1 | | Annotation Cluster 26 | | Enrichment Score: 1.62 |  |  | Count | P\_Value | Benjamini | | --- | --- | --- | --- | --- | --- | --- | --- | |  | GOTERM\_BP\_5 | regulation of synaptic vesicle transport | **RT** |  | 9 | 3.5E-3 | 9.3E-2 | |  | GOTERM\_BP\_5 | regulation of receptor-mediated endocytosis | **RT** |  | 9 | 2.1E-2 | 3.2E-1 | |  | GOTERM\_BP\_5 | regulation of synaptic vesicle recycling | **RT** |  | 4 | 1.8E-1 | 8.6E-1 | | Annotation Cluster 27 | | Enrichment Score: 1.55 |  |  | Count | P\_Value | Benjamini | | --- | --- | --- | --- | --- | --- | --- | --- | |  | GOTERM\_BP\_5 | regulation of axonogenesis | **RT** |  | 17 | 6.1E-3 | 1.4E-1 | |  | GOTERM\_BP\_5 | regulation of cell morphogenesis involved in differentiation | **RT** |  | 27 | 1.1E-2 | 2.1E-1 | |  | GOTERM\_BP\_5 | regulation of neuron projection development | **RT** |  | 31 | 1.2E-2 | 2.2E-1 | |  | GOTERM\_BP\_5 | neuron projection extension | **RT** |  | 20 | 1.6E-2 | 2.6E-1 | |  | GOTERM\_BP\_5 | axon extension | **RT** |  | 19 | 2.6E-2 | 3.6E-1 | |  | GOTERM\_BP\_5 | developmental cell growth | **RT** |  | 23 | 3.5E-2 | 4.3E-1 | |  | GOTERM\_BP\_5 | regulation of axon extension | **RT** |  | 10 | 3.5E-2 | 4.3E-1 | |  | GOTERM\_BP\_5 | regulation of extent of cell growth | **RT** |  | 10 | 3.5E-2 | 4.3E-1 | |  | GOTERM\_BP\_5 | regulation of axon guidance | **RT** |  | 9 | 4.9E-2 | 5.1E-1 | |  | GOTERM\_BP\_5 | regulation of cell size | **RT** |  | 20 | 4.2E-1 | 9.9E-1 | | Annotation Cluster 28 | | Enrichment Score: 1.46 |  |  | Count | P\_Value | Benjamini | | --- | --- | --- | --- | --- | --- | --- | --- | |  | GOTERM\_MF\_5 | microtubule binding | **RT** |  | 26 | 9.4E-3 | 1.6E-1 | |  | GOTERM\_CC\_5 | polymeric cytoskeletal fiber | **RT** |  | 28 | 5.1E-2 | 4.8E-1 | |  | GOTERM\_CC\_5 | microtubule | **RT** |  | 20 | 8.5E-2 | 6.4E-1 | | Annotation Cluster 29 | | Enrichment Score: 1.44 |  |  | Count | P\_Value | Benjamini | | --- | --- | --- | --- | --- | --- | --- | --- | |  | GOTERM\_CC\_5 | cytoplasmic side of plasma membrane | **RT** |  | 14 | 5.8E-3 | 1.2E-1 | |  | GOTERM\_CC\_5 | extrinsic component of cytoplasmic side of plasma membrane | **RT** |  | 10 | 3.7E-2 | 4.1E-1 | |  | GOTERM\_CC\_5 | extrinsic component of plasma membrane | **RT** |  | 12 | 4.2E-2 | 4.4E-1 | |  | GOTERM\_CC\_5 | heterotrimeric G-protein complex | **RT** |  | 5 | 1.9E-1 | 8.4E-1 | | Annotation Cluster 30 | | Enrichment Score: 1.41 |  |  | Count | P\_Value | Benjamini | | --- | --- | --- | --- | --- | --- | --- | --- | |  | GOTERM\_BP\_5 | cell fate commitment involved in pattern specification | **RT** |  | 11 | 4.0E-3 | 1.0E-1 | |  | GOTERM\_BP\_5 | formation of organ boundary | **RT** |  | 14 | 1.5E-2 | 2.6E-1 | |  | GOTERM\_BP\_5 | formation of anatomical boundary | **RT** |  | 15 | 2.8E-2 | 3.7E-1 | |  | GOTERM\_BP\_5 | organ formation | **RT** |  | 14 | 3.7E-2 | 4.5E-1 | |  | GOTERM\_BP\_5 | sensory organ boundary specification | **RT** |  | 7 | 4.0E-2 | 4.7E-1 | |  | GOTERM\_BP\_5 | sensory organ precursor cell fate determination | **RT** |  | 6 | 8.9E-2 | 6.9E-1 | |  | GOTERM\_BP\_5 | cell fate determination involved in pattern specification | **RT** |  | 6 | 8.9E-2 | 6.9E-1 | |  | GOTERM\_BP\_5 | salivary gland boundary specification | **RT** |  | 7 | 2.6E-1 | 9.3E-1 | | Annotation Cluster 31 | | Enrichment Score: 1.37 |  |  | Count | P\_Value | Benjamini | | --- | --- | --- | --- | --- | --- | --- | --- | |  | GOTERM\_BP\_5 | circadian sleep/wake cycle process | **RT** |  | 11 | 1.1E-2 | 2.2E-1 | |  | GOTERM\_BP\_5 | circadian sleep/wake cycle, sleep | **RT** |  | 9 | 3.3E-2 | 4.1E-1 | |  | GOTERM\_BP\_5 | positive regulation of circadian sleep/wake cycle, sleep | **RT** |  | 7 | 6.3E-2 | 5.9E-1 | |  | GOTERM\_BP\_5 | regulation of circadian sleep/wake cycle, sleep | **RT** |  | 10 | 1.4E-1 | 8.1E-1 | | Annotation Cluster 32 | | Enrichment Score: 1.34 |  |  | Count | P\_Value | Benjamini | | --- | --- | --- | --- | --- | --- | --- | --- | |  | GOTERM\_BP\_5 | dopamine receptor signaling pathway | **RT** |  | 6 | 3.2E-2 | 4.0E-1 | |  | GOTERM\_MF\_5 | dopamine neurotransmitter receptor activity | **RT** |  | 6 | 3.5E-2 | 3.8E-1 | |  | GOTERM\_MF\_5 | dopamine neurotransmitter receptor activity, coupled via Gs | **RT** |  | 5 | 8.6E-2 | 6.5E-1 | | Annotation Cluster 33 | | Enrichment Score: 1.32 |  |  | Count | P\_Value | Benjamini | | --- | --- | --- | --- | --- | --- | --- | --- | |  | GOTERM\_BP\_5 | positive regulation of cyclase activity | **RT** |  | 9 | 3.5E-3 | 9.3E-2 | |  | GOTERM\_BP\_5 | positive regulation of lyase activity | **RT** |  | 9 | 3.5E-3 | 9.3E-2 | |  | GOTERM\_BP\_5 | regulation of adenylate cyclase activity | **RT** |  | 10 | 5.4E-3 | 1.3E-1 | |  | GOTERM\_BP\_5 | ribose phosphate biosynthetic process | **RT** |  | 26 | 6.9E-3 | 1.6E-1 | |  | GOTERM\_BP\_5 | regulation of nucleotide biosynthetic process | **RT** |  | 11 | 7.0E-3 | 1.6E-1 | |  | GOTERM\_BP\_5 | positive regulation of nucleotide biosynthetic process | **RT** |  | 9 | 7.1E-3 | 1.6E-1 | |  | GOTERM\_BP\_5 | positive regulation of nucleotide metabolic process | **RT** |  | 9 | 1.3E-2 | 2.4E-1 | |  | GOTERM\_BP\_5 | purine-containing compound biosynthetic process | **RT** |  | 25 | 1.4E-2 | 2.6E-1 | |  | GOTERM\_BP\_5 | nucleotide biosynthetic process | **RT** |  | 28 | 1.6E-2 | 2.6E-1 | |  | GOTERM\_BP\_5 | regulation of nucleotide metabolic process | **RT** |  | 12 | 1.9E-2 | 3.0E-1 | |  | GOTERM\_BP\_5 | nucleoside phosphate biosynthetic process | **RT** |  | 28 | 1.9E-2 | 3.0E-1 | |  | GOTERM\_BP\_5 | organophosphate biosynthetic process | **RT** |  | 34 | 1.3E-1 | 7.9E-1 | |  | GOTERM\_BP\_5 | ribonucleotide metabolic process | **RT** |  | 33 | 8.6E-1 | 1.0E0 | |  | GOTERM\_BP\_5 | ribose phosphate metabolic process | **RT** |  | 33 | 8.7E-1 | 1.0E0 | |  | GOTERM\_BP\_5 | purine nucleotide metabolic process | **RT** |  | 32 | 8.7E-1 | 1.0E0 | |  | GOTERM\_BP\_5 | nucleotide metabolic process | **RT** |  | 37 | 9.1E-1 | 1.0E0 | |  | GOTERM\_BP\_5 | nucleoside phosphate metabolic process | **RT** |  | 37 | 9.2E-1 | 1.0E0 | |  | GOTERM\_BP\_5 | nucleobase-containing small molecule metabolic process | **RT** |  | 38 | 9.7E-1 | 1.0E0 | | Annotation Cluster 34 | | Enrichment Score: 1.29 |  |  | Count | P\_Value | Benjamini | | --- | --- | --- | --- | --- | --- | --- | --- | |  | GOTERM\_BP\_5 | negative regulation of neuron differentiation | **RT** |  | 13 | 1.4E-2 | 2.6E-1 | |  | GOTERM\_BP\_5 | negative regulation of cell development | **RT** |  | 24 | 1.5E-2 | 2.6E-1 | |  | GOTERM\_BP\_5 | negative regulation of cell morphogenesis involved in differentiation | **RT** |  | 9 | 3.3E-2 | 4.1E-1 | |  | GOTERM\_BP\_5 | negative regulation of neuron projection development | **RT** |  | 10 | 3.5E-2 | 4.3E-1 | |  | GOTERM\_BP\_5 | negative regulation of neurogenesis | **RT** |  | 20 | 4.0E-2 | 4.6E-1 | |  | GOTERM\_BP\_5 | negative regulation of cell projection organization | **RT** |  | 10 | 9.0E-2 | 6.9E-1 | |  | GOTERM\_BP\_5 | negative regulation of dendrite morphogenesis | **RT** |  | 5 | 2.5E-1 | 9.2E-1 | |  | GOTERM\_BP\_5 | negative regulation of dendrite development | **RT** |  | 5 | 2.5E-1 | 9.2E-1 | | Annotation Cluster 35 | | Enrichment Score: 1.25 |  |  | Count | P\_Value | Benjamini | | --- | --- | --- | --- | --- | --- | --- | --- | |  | GOTERM\_BP\_5 | reproductive system development | **RT** |  | 16 | 4.6E-2 | 4.9E-1 | |  | GOTERM\_BP\_5 | reproductive structure development | **RT** |  | 16 | 4.6E-2 | 4.9E-1 | |  | GOTERM\_BP\_5 | gonad development | **RT** |  | 10 | 6.8E-2 | 6.1E-1 | |  | GOTERM\_BP\_5 | development of primary sexual characteristics | **RT** |  | 10 | 6.8E-2 | 6.1E-1 | | Annotation Cluster 36 | | Enrichment Score: 1.24 |  |  | Count | P\_Value | Benjamini | | --- | --- | --- | --- | --- | --- | --- | --- | |  | GOTERM\_CC\_5 | spindle pole | **RT** |  | 13 | 5.8E-3 | 1.3E-1 | |  | GOTERM\_CC\_5 | spindle | **RT** |  | 25 | 3.2E-2 | 3.9E-1 | |  | GOTERM\_CC\_5 | microtubule organizing center | **RT** |  | 20 | 2.2E-1 | 8.7E-1 | |  | GOTERM\_CC\_5 | centrosome | **RT** |  | 19 | 2.6E-1 | 9.1E-1 | | Annotation Cluster 37 | | Enrichment Score: 1.24 |  |  | Count | P\_Value | Benjamini | | --- | --- | --- | --- | --- | --- | --- | --- | |  | GOTERM\_BP\_5 | heart development | **RT** |  | 21 | 5.5E-2 | 5.5E-1 | |  | GOTERM\_BP\_5 | circulatory system development | **RT** |  | 21 | 5.5E-2 | 5.5E-1 | |  | GOTERM\_BP\_5 | cardiovascular system development | **RT** |  | 21 | 5.5E-2 | 5.5E-1 | |  | GOTERM\_BP\_5 | cardiocyte differentiation | **RT** |  | 7 | 6.3E-2 | 5.9E-1 | | Annotation Cluster 38 | | Enrichment Score: 1.24 |  |  | Count | P\_Value | Benjamini | | --- | --- | --- | --- | --- | --- | --- | --- | |  | GOTERM\_BP\_5 | regulation of regulated secretory pathway | **RT** |  | 9 | 3.5E-3 | 9.3E-2 | |  | GOTERM\_BP\_5 | regulation of synaptic vesicle transport | **RT** |  | 9 | 3.5E-3 | 9.3E-2 | |  | GOTERM\_BP\_5 | regulation of exocytosis | **RT** |  | 11 | 7.0E-3 | 1.6E-1 | |  | GOTERM\_BP\_5 | regulation of synaptic vesicle exocytosis | **RT** |  | 5 | 4.2E-2 | 4.8E-1 | |  | GOTERM\_BP\_5 | synaptic vesicle exocytosis | **RT** |  | 19 | 8.7E-2 | 6.9E-1 | |  | GOTERM\_BP\_5 | exocytosis | **RT** |  | 26 | 1.2E-1 | 7.7E-1 | |  | GOTERM\_BP\_5 | regulated exocytosis | **RT** |  | 19 | 1.2E-1 | 7.7E-1 | |  | GOTERM\_BP\_5 | vesicle fusion | **RT** |  | 12 | 3.6E-1 | 9.7E-1 | |  | GOTERM\_BP\_5 | organelle membrane fusion | **RT** |  | 12 | 4.3E-1 | 9.9E-1 | |  | GOTERM\_BP\_5 | single-organism membrane fusion | **RT** |  | 12 | 6.1E-1 | 1.0E0 | | Annotation Cluster 39 | | Enrichment Score: 1.18 |  |  | Count | P\_Value | Benjamini | | --- | --- | --- | --- | --- | --- | --- | --- | |  | GOTERM\_CC\_5 | cell cortex region | **RT** |  | 15 | 8.1E-3 | 1.5E-1 | |  | GOTERM\_CC\_5 | cell cortex part | **RT** |  | 16 | 9.5E-2 | 6.6E-1 | |  | GOTERM\_CC\_5 | cytoplasmic region | **RT** |  | 34 | 1.0E-1 | 6.7E-1 | |  | GOTERM\_CC\_5 | apical cortex | **RT** |  | 10 | 1.3E-1 | 7.5E-1 | |  | GOTERM\_CC\_5 | cell cortex | **RT** |  | 30 | 1.3E-1 | 7.5E-1 | | Annotation Cluster 40 | | Enrichment Score: 1.16 |  |  | Count | P\_Value | Benjamini | | --- | --- | --- | --- | --- | --- | --- | --- | |  | GOTERM\_CC\_5 | ionotropic glutamate receptor complex | **RT** |  | 5 | 1.2E-2 | 2.0E-1 | |  | GOTERM\_MF\_5 | transmitter-gated ion channel activity | **RT** |  | 7 | 7.1E-2 | 5.9E-1 | |  | GOTERM\_MF\_5 | transmitter-gated channel activity | **RT** |  | 7 | 7.1E-2 | 5.9E-1 | |  | GOTERM\_MF\_5 | glutamate receptor activity | **RT** |  | 6 | 1.4E-1 | 7.9E-1 | |  | GOTERM\_MF\_5 | ionotropic glutamate receptor activity | **RT** |  | 5 | 2.0E-1 | 8.6E-1 | | Annotation Cluster 41 | | Enrichment Score: 1.12 |  |  | Count | P\_Value | Benjamini | | --- | --- | --- | --- | --- | --- | --- | --- | |  | GOTERM\_MF\_5 | carboxylic acid transmembrane transporter activity | **RT** |  | 16 | 3.3E-3 | 6.9E-2 | |  | GOTERM\_BP\_5 | amino acid transmembrane transport | **RT** |  | 7 | 1.3E-1 | 7.9E-1 | |  | GOTERM\_BP\_5 | organic acid transmembrane transport | **RT** |  | 7 | 1.7E-1 | 8.4E-1 | |  | GOTERM\_BP\_5 | carboxylic acid transmembrane transport | **RT** |  | 7 | 1.7E-1 | 8.4E-1 | |  | GOTERM\_BP\_5 | organic acid transport | **RT** |  | 7 | 2.1E-1 | 8.9E-1 | | Annotation Cluster 42 | | Enrichment Score: 1.12 |  |  | Count | P\_Value | Benjamini | | --- | --- | --- | --- | --- | --- | --- | --- | |  | GOTERM\_BP\_5 | stem cell differentiation | **RT** |  | 20 | 5.0E-4 | 1.9E-2 | |  | GOTERM\_BP\_5 | renal filtration cell differentiation | **RT** |  | 7 | 1.6E-3 | 4.9E-2 | |  | GOTERM\_BP\_5 | renal system development | **RT** |  | 17 | 8.7E-3 | 1.8E-1 | |  | GOTERM\_BP\_5 | urogenital system development | **RT** |  | 17 | 8.7E-3 | 1.8E-1 | |  | GOTERM\_BP\_5 | digestive tract development | **RT** |  | 19 | 3.3E-2 | 4.1E-1 | |  | GOTERM\_BP\_5 | digestive system development | **RT** |  | 19 | 3.3E-2 | 4.1E-1 | |  | GOTERM\_BP\_5 | cardiocyte differentiation | **RT** |  | 7 | 6.3E-2 | 5.9E-1 | |  | GOTERM\_BP\_5 | cardioblast differentiation | **RT** |  | 5 | 1.3E-1 | 7.9E-1 | |  | GOTERM\_BP\_5 | Malpighian tubule development | **RT** |  | 12 | 1.3E-1 | 7.9E-1 | |  | GOTERM\_BP\_5 | renal tubule development | **RT** |  | 12 | 1.3E-1 | 7.9E-1 | |  | GOTERM\_BP\_5 | embryonic hindgut morphogenesis | **RT** |  | 9 | 2.7E-1 | 9.3E-1 | |  | GOTERM\_BP\_5 | cardiac cell fate commitment | **RT** |  | 4 | 3.4E-1 | 9.7E-1 | |  | GOTERM\_BP\_5 | epithelial cell proliferation involved in Malpighian tubule morphogenesis | **RT** |  | 3 | 3.9E-1 | 9.8E-1 | |  | GOTERM\_BP\_5 | hindgut development | **RT** |  | 9 | 4.4E-1 | 9.9E-1 | |  | GOTERM\_BP\_5 | hindgut morphogenesis | **RT** |  | 9 | 4.4E-1 | 9.9E-1 | |  | GOTERM\_BP\_5 | Malpighian tubule morphogenesis | **RT** |  | 7 | 4.7E-1 | 9.9E-1 | |  | GOTERM\_BP\_5 | renal tubule morphogenesis | **RT** |  | 7 | 4.7E-1 | 9.9E-1 | |  | GOTERM\_BP\_5 | digestive tract morphogenesis | **RT** |  | 10 | 5.4E-1 | 9.9E-1 | | Annotation Cluster 43 | | Enrichment Score: 1.03 |  |  | Count | P\_Value | Benjamini | | --- | --- | --- | --- | --- | --- | --- | --- | |  | GOTERM\_BP\_5 | negative regulation of Wnt signaling pathway | **RT** |  | 13 | 1.4E-2 | 2.6E-1 | |  | GOTERM\_BP\_5 | regulation of Wnt signaling pathway | **RT** |  | 22 | 1.0E-1 | 7.2E-1 | |  | GOTERM\_BP\_5 | cell surface receptor signaling pathway involved in cell-cell signaling | **RT** |  | 28 | 1.5E-1 | 8.2E-1 | |  | GOTERM\_BP\_5 | Wnt signaling pathway | **RT** |  | 27 | 1.7E-1 | 8.5E-1 | |  | GOTERM\_BP\_5 | cell-cell signaling by wnt | **RT** |  | 27 | 1.9E-1 | 8.7E-1 | | Annotation Cluster 44 | | Enrichment Score: 1.01 |  |  | Count | P\_Value | Benjamini | | --- | --- | --- | --- | --- | --- | --- | --- | |  | GOTERM\_CC\_5 | cell projection membrane | **RT** |  | 7 | 8.4E-2 | 6.4E-1 | |  | GOTERM\_CC\_5 | neuron projection membrane | **RT** |  | 6 | 8.9E-2 | 6.4E-1 | |  | GOTERM\_CC\_5 | leading edge membrane | **RT** |  | 6 | 1.2E-1 | 7.4E-1 | | Annotation Cluster 45 | | Enrichment Score: 0.99 |  |  | Count | P\_Value | Benjamini | | --- | --- | --- | --- | --- | --- | --- | --- | |  | GOTERM\_BP\_5 | regulation of ion transport | **RT** |  | 17 | 7.3E-4 | 2.6E-2 | |  | GOTERM\_BP\_5 | regulation of transporter activity | **RT** |  | 6 | 3.2E-2 | 4.0E-1 | |  | GOTERM\_BP\_5 | regulation of transmembrane transporter activity | **RT** |  | 6 | 3.2E-2 | 4.0E-1 | |  | GOTERM\_BP\_5 | regulation of ion transmembrane transporter activity | **RT** |  | 6 | 3.2E-2 | 4.0E-1 | |  | GOTERM\_BP\_5 | regulation of transmembrane transport | **RT** |  | 6 | 8.9E-2 | 6.9E-1 | |  | GOTERM\_BP\_5 | regulation of ion transmembrane transport | **RT** |  | 6 | 8.9E-2 | 6.9E-1 | |  | GOTERM\_BP\_5 | positive regulation of ion transport | **RT** |  | 6 | 8.9E-2 | 6.9E-1 | |  | GOTERM\_BP\_5 | positive regulation of ion transmembrane transporter activity | **RT** |  | 4 | 1.1E-1 | 7.6E-1 | |  | GOTERM\_BP\_5 | positive regulation of transporter activity | **RT** |  | 4 | 1.1E-1 | 7.6E-1 | |  | GOTERM\_BP\_5 | regulation of cation transmembrane transport | **RT** |  | 5 | 1.8E-1 | 8.6E-1 | |  | GOTERM\_BP\_5 | negative regulation of ion transport | **RT** |  | 4 | 1.8E-1 | 8.6E-1 | |  | GOTERM\_BP\_5 | positive regulation of transmembrane transport | **RT** |  | 4 | 2.6E-1 | 9.3E-1 | |  | GOTERM\_BP\_5 | positive regulation of ion transmembrane transport | **RT** |  | 4 | 2.6E-1 | 9.3E-1 | |  | GOTERM\_BP\_5 | negative regulation of transporter activity | **RT** |  | 3 | 2.8E-1 | 9.4E-1 | |  | GOTERM\_BP\_5 | negative regulation of transmembrane transport | **RT** |  | 3 | 2.8E-1 | 9.4E-1 | |  | GOTERM\_BP\_5 | negative regulation of ion transmembrane transporter activity | **RT** |  | 3 | 2.8E-1 | 9.4E-1 | |  | GOTERM\_BP\_5 | negative regulation of ion transmembrane transport | **RT** |  | 3 | 2.8E-1 | 9.4E-1 | |  | GOTERM\_BP\_5 | positive regulation of cation transmembrane transport | **RT** |  | 3 | 4.9E-1 | 9.9E-1 | | Annotation Cluster 46 | | Enrichment Score: 0.9 |  |  | Count | P\_Value | Benjamini | | --- | --- | --- | --- | --- | --- | --- | --- | |  | GOTERM\_BP\_5 | regulation of receptor-mediated endocytosis | **RT** |  | 9 | 2.1E-2 | 3.2E-1 | |  | GOTERM\_BP\_5 | positive regulation of clathrin-mediated endocytosis | **RT** |  | 5 | 7.9E-2 | 6.5E-1 | |  | GOTERM\_BP\_5 | positive regulation of receptor-mediated endocytosis | **RT** |  | 6 | 1.3E-1 | 7.9E-1 | |  | GOTERM\_BP\_5 | regulation of endocytosis | **RT** |  | 14 | 2.2E-1 | 8.9E-1 | |  | GOTERM\_BP\_5 | positive regulation of synaptic vesicle transport | **RT** |  | 3 | 2.8E-1 | 9.4E-1 | |  | GOTERM\_BP\_5 | positive regulation of endocytosis | **RT** |  | 8 | 2.9E-1 | 9.5E-1 | | Annotation Cluster 47 | | Enrichment Score: 0.85 |  |  | Count | P\_Value | Benjamini | | --- | --- | --- | --- | --- | --- | --- | --- | |  | GOTERM\_CC\_5 | cytoskeleton of presynaptic active zone | **RT** |  | 4 | 8.6E-2 | 6.4E-1 | |  | GOTERM\_CC\_5 | presynaptic active zone cytoplasmic component | **RT** |  | 4 | 8.6E-2 | 6.4E-1 | |  | GOTERM\_CC\_5 | presynaptic cytoskeleton | **RT** |  | 4 | 8.6E-2 | 6.4E-1 | |  | GOTERM\_CC\_5 | cortical cytoskeleton | **RT** |  | 4 | 6.1E-1 | 1.0E0 | | Annotation Cluster 48 | | Enrichment Score: 0.85 |  |  | Count | P\_Value | Benjamini | | --- | --- | --- | --- | --- | --- | --- | --- | |  | GOTERM\_BP\_5 | embryo development ending in birth or egg hatching | **RT** |  | 42 | 4.3E-2 | 4.8E-1 | |  | GOTERM\_BP\_5 | embryonic morphogenesis | **RT** |  | 44 | 6.9E-2 | 6.1E-1 | |  | GOTERM\_BP\_5 | dorsal closure | **RT** |  | 22 | 2.8E-1 | 9.4E-1 | |  | GOTERM\_BP\_5 | morphogenesis of embryonic epithelium | **RT** |  | 22 | 4.9E-1 | 9.9E-1 | | Annotation Cluster 49 | | Enrichment Score: 0.85 |  |  | Count | P\_Value | Benjamini | | --- | --- | --- | --- | --- | --- | --- | --- | |  | GOTERM\_BP\_5 | imaginal disc pattern formation | **RT** |  | 23 | 1.5E-2 | 2.6E-1 | |  | GOTERM\_BP\_5 | wing disc pattern formation | **RT** |  | 16 | 1.0E-1 | 7.3E-1 | |  | GOTERM\_BP\_5 | dorsal/ventral pattern formation, imaginal disc | **RT** |  | 11 | 3.4E-1 | 9.7E-1 | |  | GOTERM\_BP\_5 | dorsal/ventral pattern formation | **RT** |  | 20 | 7.7E-1 | 1.0E0 | | Annotation Cluster 50 | | Enrichment Score: 0.84 |  |  | Count | P\_Value | Benjamini | | --- | --- | --- | --- | --- | --- | --- | --- | |  | GOTERM\_BP\_5 | regulation of cell fate specification | **RT** |  | 9 | 6.8E-2 | 6.1E-1 | |  | GOTERM\_BP\_5 | compound eye cone cell differentiation | **RT** |  | 6 | 1.3E-1 | 7.9E-1 | |  | GOTERM\_BP\_5 | compound eye cone cell fate commitment | **RT** |  | 4 | 1.8E-1 | 8.6E-1 | |  | GOTERM\_BP\_5 | regulation of compound eye cone cell fate specification | **RT** |  | 3 | 2.8E-1 | 9.4E-1 | | Annotation Cluster 51 | | Enrichment Score: 0.83 |  |  | Count | P\_Value | Benjamini | | --- | --- | --- | --- | --- | --- | --- | --- | |  | GOTERM\_BP\_5 | hormone transport | **RT** |  | 9 | 2.1E-2 | 3.2E-1 | |  | GOTERM\_BP\_5 | regulation of hormone secretion | **RT** |  | 9 | 2.1E-2 | 3.2E-1 | |  | GOTERM\_BP\_5 | hormone secretion | **RT** |  | 9 | 2.1E-2 | 3.2E-1 | |  | GOTERM\_BP\_5 | peptide transport | **RT** |  | 8 | 4.6E-2 | 4.9E-1 | |  | GOTERM\_BP\_5 | peptide secretion | **RT** |  | 8 | 4.6E-2 | 4.9E-1 | |  | GOTERM\_BP\_5 | amide transport | **RT** |  | 8 | 6.7E-2 | 6.1E-1 | |  | GOTERM\_BP\_5 | regulation of peptide transport | **RT** |  | 7 | 9.3E-2 | 7.0E-1 | |  | GOTERM\_BP\_5 | regulation of peptide secretion | **RT** |  | 7 | 9.3E-2 | 7.0E-1 | |  | GOTERM\_BP\_5 | regulation of peptide hormone secretion | **RT** |  | 6 | 1.8E-1 | 8.5E-1 | |  | GOTERM\_BP\_5 | negative regulation of hormone secretion | **RT** |  | 3 | 2.8E-1 | 9.4E-1 | |  | GOTERM\_BP\_5 | negative regulation of peptide hormone secretion | **RT** |  | 3 | 2.8E-1 | 9.4E-1 | |  | GOTERM\_BP\_5 | negative regulation of peptide secretion | **RT** |  | 3 | 2.8E-1 | 9.4E-1 | |  | GOTERM\_BP\_5 | regulation of insulin secretion | **RT** |  | 3 | 3.9E-1 | 9.8E-1 | |  | GOTERM\_BP\_5 | positive regulation of hormone secretion | **RT** |  | 4 | 4.2E-1 | 9.9E-1 | |  | GOTERM\_BP\_5 | negative regulation of secretion by cell | **RT** |  | 3 | 5.8E-1 | 1.0E0 | |  | GOTERM\_BP\_5 | negative regulation of secretion | **RT** |  | 3 | 6.6E-1 | 1.0E0 | |  | GOTERM\_BP\_5 | positive regulation of peptide secretion | **RT** |  | 3 | 6.6E-1 | 1.0E0 | |  | GOTERM\_BP\_5 | regulation of protein secretion | **RT** |  | 5 | 7.7E-1 | 1.0E0 | | Annotation Cluster 52 | | Enrichment Score: 0.82 |  |  | Count | P\_Value | Benjamini | | --- | --- | --- | --- | --- | --- | --- | --- | |  | GOTERM\_BP\_5 | negative regulation of smoothened signaling pathway | **RT** |  | 8 | 6.7E-2 | 6.1E-1 | |  | GOTERM\_BP\_5 | regulation of smoothened signaling pathway | **RT** |  | 9 | 2.3E-1 | 9.0E-1 | |  | GOTERM\_BP\_5 | smoothened signaling pathway | **RT** |  | 13 | 2.3E-1 | 9.1E-1 | | Annotation Cluster 53 | | Enrichment Score: 0.8 |  |  | Count | P\_Value | Benjamini | | --- | --- | --- | --- | --- | --- | --- | --- | |  | GOTERM\_BP\_5 | positive regulation of nervous system development | **RT** |  | 31 | 7.7E-3 | 1.7E-1 | |  | GOTERM\_BP\_5 | positive regulation of neurogenesis | **RT** |  | 18 | 1.3E-1 | 7.9E-1 | |  | GOTERM\_BP\_5 | positive regulation of neuron projection development | **RT** |  | 11 | 1.4E-1 | 8.0E-1 | |  | GOTERM\_BP\_5 | positive regulation of cell projection organization | **RT** |  | 15 | 1.5E-1 | 8.3E-1 | |  | GOTERM\_BP\_5 | positive regulation of neuron differentiation | **RT** |  | 15 | 1.8E-1 | 8.5E-1 | |  | GOTERM\_BP\_5 | positive regulation of cell morphogenesis involved in differentiation | **RT** |  | 9 | 2.3E-1 | 9.0E-1 | |  | GOTERM\_BP\_5 | positive regulation of cell development | **RT** |  | 19 | 2.8E-1 | 9.4E-1 | |  | GOTERM\_BP\_5 | regulation of dendrite morphogenesis | **RT** |  | 12 | 2.8E-1 | 9.4E-1 | |  | GOTERM\_BP\_5 | positive regulation of dendrite morphogenesis | **RT** |  | 6 | 3.4E-1 | 9.7E-1 | |  | GOTERM\_BP\_5 | positive regulation of dendrite development | **RT** |  | 6 | 4.6E-1 | 9.9E-1 | | Annotation Cluster 54 | | Enrichment Score: 0.79 |  |  | Count | P\_Value | Benjamini | | --- | --- | --- | --- | --- | --- | --- | --- | |  | GOTERM\_BP\_5 | regulation of cell fate specification | **RT** |  | 9 | 6.8E-2 | 6.1E-1 | |  | GOTERM\_BP\_5 | negative regulation of cell fate commitment | **RT** |  | 7 | 2.1E-1 | 8.9E-1 | |  | GOTERM\_BP\_5 | negative regulation of cell fate specification | **RT** |  | 6 | 2.9E-1 | 9.4E-1 | | Annotation Cluster 55 | | Enrichment Score: 0.78 |  |  | Count | P\_Value | Benjamini | | --- | --- | --- | --- | --- | --- | --- | --- | |  | GOTERM\_BP\_5 | response to acetylcholine | **RT** |  | 3 | 1.7E-1 | 8.4E-1 | |  | GOTERM\_BP\_5 | acetylcholine receptor signaling pathway | **RT** |  | 3 | 1.7E-1 | 8.4E-1 | |  | GOTERM\_BP\_5 | cellular response to acetylcholine | **RT** |  | 3 | 1.7E-1 | 8.4E-1 | |  | GOTERM\_BP\_5 | signal transduction involved in cellular response to ammonium ion | **RT** |  | 3 | 1.7E-1 | 8.4E-1 | |  | GOTERM\_BP\_5 | cellular response to ammonium ion | **RT** |  | 3 | 1.7E-1 | 8.4E-1 | |  | GOTERM\_BP\_5 | G-protein coupled acetylcholine receptor signaling pathway | **RT** |  | 3 | 1.7E-1 | 8.4E-1 | | Annotation Cluster 56 | | Enrichment Score: 0.77 |  |  | Count | P\_Value | Benjamini | | --- | --- | --- | --- | --- | --- | --- | --- | |  | GOTERM\_BP\_5 | positive regulation of synapse assembly | **RT** |  | 13 | 5.0E-2 | 5.2E-1 | |  | GOTERM\_BP\_5 | positive regulation of neuromuscular junction development | **RT** |  | 10 | 2.1E-1 | 8.9E-1 | |  | GOTERM\_BP\_5 | positive regulation of synaptic growth at neuromuscular junction | **RT** |  | 10 | 2.1E-1 | 8.9E-1 | |  | GOTERM\_BP\_5 | positive regulation of developmental growth | **RT** |  | 22 | 3.8E-1 | 9.8E-1 | | Annotation Cluster 57 | | Enrichment Score: 0.74 |  |  | Count | P\_Value | Benjamini | | --- | --- | --- | --- | --- | --- | --- | --- | |  | GOTERM\_BP\_5 | muscle cell differentiation | **RT** |  | 26 | 2.1E-2 | 3.1E-1 | |  | GOTERM\_BP\_5 | striated muscle cell differentiation | **RT** |  | 22 | 7.4E-2 | 6.3E-1 | |  | GOTERM\_BP\_5 | muscle cell development | **RT** |  | 12 | 1.0E-1 | 7.4E-1 | |  | GOTERM\_BP\_5 | striated muscle cell development | **RT** |  | 11 | 1.6E-1 | 8.4E-1 | |  | GOTERM\_BP\_5 | actomyosin structure organization | **RT** |  | 13 | 2.0E-1 | 8.8E-1 | |  | GOTERM\_BP\_5 | sarcomere organization | **RT** |  | 7 | 2.1E-1 | 8.9E-1 | |  | GOTERM\_BP\_5 | myofibril assembly | **RT** |  | 8 | 2.4E-1 | 9.2E-1 | |  | GOTERM\_BP\_5 | myoblast fusion | **RT** |  | 10 | 3.3E-1 | 9.6E-1 | |  | GOTERM\_BP\_5 | syncytium formation | **RT** |  | 11 | 3.8E-1 | 9.8E-1 | |  | GOTERM\_BP\_5 | syncytium formation by plasma membrane fusion | **RT** |  | 11 | 3.8E-1 | 9.8E-1 | |  | GOTERM\_BP\_5 | cellular component assembly involved in morphogenesis | **RT** |  | 20 | 5.6E-1 | 1.0E0 | | Annotation Cluster 58 | | Enrichment Score: 0.69 |  |  | Count | P\_Value | Benjamini | | --- | --- | --- | --- | --- | --- | --- | --- | |  | GOTERM\_BP\_5 | regulation of microtubule polymerization or depolymerization | **RT** |  | 8 | 4.6E-2 | 4.9E-1 | |  | GOTERM\_BP\_5 | negative regulation of protein depolymerization | **RT** |  | 6 | 8.9E-2 | 6.9E-1 | |  | GOTERM\_BP\_5 | negative regulation of protein complex disassembly | **RT** |  | 6 | 8.9E-2 | 6.9E-1 | |  | GOTERM\_BP\_5 | microtubule polymerization or depolymerization | **RT** |  | 10 | 1.8E-1 | 8.5E-1 | |  | GOTERM\_BP\_5 | regulation of protein depolymerization | **RT** |  | 6 | 2.9E-1 | 9.4E-1 | |  | GOTERM\_BP\_5 | protein complex disassembly | **RT** |  | 10 | 5.0E-1 | 9.9E-1 | |  | GOTERM\_BP\_5 | negative regulation of cytoskeleton organization | **RT** |  | 7 | 5.2E-1 | 9.9E-1 | |  | GOTERM\_BP\_5 | negative regulation of organelle organization | **RT** |  | 13 | 7.0E-1 | 1.0E0 | | Annotation Cluster 59 | | Enrichment Score: 0.68 |  |  | Count | P\_Value | Benjamini | | --- | --- | --- | --- | --- | --- | --- | --- | |  | GOTERM\_BP\_5 | formation of primary germ layer | **RT** |  | 7 | 9.3E-2 | 7.0E-1 | |  | GOTERM\_BP\_5 | mesoderm morphogenesis | **RT** |  | 7 | 9.3E-2 | 7.0E-1 | |  | GOTERM\_BP\_5 | mesoderm formation | **RT** |  | 6 | 1.3E-1 | 7.9E-1 | |  | GOTERM\_BP\_5 | mesoderm migration involved in gastrulation | **RT** |  | 4 | 2.6E-1 | 9.3E-1 | |  | GOTERM\_BP\_5 | gastrulation | **RT** |  | 12 | 3.6E-1 | 9.7E-1 | |  | GOTERM\_BP\_5 | cell migration involved in gastrulation | **RT** |  | 5 | 3.8E-1 | 9.8E-1 | |  | GOTERM\_BP\_5 | mesodermal cell migration | **RT** |  | 4 | 4.2E-1 | 9.9E-1 | | Annotation Cluster 60 | | Enrichment Score: 0.68 |  |  | Count | P\_Value | Benjamini | | --- | --- | --- | --- | --- | --- | --- | --- | |  | GOTERM\_MF\_5 | acetylgalactosaminyltransferase activity | **RT** |  | 7 | 1.4E-2 | 2.0E-1 | |  | GOTERM\_BP\_5 | oligosaccharide biosynthetic process | **RT** |  | 5 | 4.2E-2 | 4.8E-1 | |  | GOTERM\_BP\_5 | carbohydrate biosynthetic process | **RT** |  | 11 | 1.1E-1 | 7.5E-1 | |  | GOTERM\_BP\_5 | glycoprotein biosynthetic process | **RT** |  | 22 | 1.3E-1 | 7.9E-1 | |  | GOTERM\_BP\_5 | glycoprotein metabolic process | **RT** |  | 22 | 1.3E-1 | 7.9E-1 | |  | GOTERM\_BP\_5 | macromolecule glycosylation | **RT** |  | 19 | 1.6E-1 | 8.3E-1 | |  | GOTERM\_BP\_5 | glycosylation | **RT** |  | 19 | 1.6E-1 | 8.3E-1 | |  | GOTERM\_CC\_5 | Golgi stack | **RT** |  | 13 | 2.6E-1 | 9.1E-1 | |  | GOTERM\_CC\_5 | Golgi membrane | **RT** |  | 24 | 3.0E-1 | 9.3E-1 | |  | GOTERM\_BP\_5 | oligosaccharide metabolic process | **RT** |  | 6 | 4.0E-1 | 9.8E-1 | |  | GOTERM\_CC\_5 | organelle subcompartment | **RT** |  | 17 | 4.7E-1 | 9.9E-1 | |  | GOTERM\_CC\_5 | Golgi cisterna | **RT** |  | 7 | 5.0E-1 | 9.9E-1 | |  | GOTERM\_CC\_5 | Golgi subcompartment | **RT** |  | 16 | 5.1E-1 | 9.9E-1 | |  | GOTERM\_CC\_5 | Golgi apparatus part | **RT** |  | 32 | 6.8E-1 | 1.0E0 | |  | GOTERM\_CC\_5 | Golgi apparatus | **RT** |  | 43 | 8.6E-1 | 1.0E0 | | Annotation Cluster 61 | | Enrichment Score: 0.68 |  |  | Count | P\_Value | Benjamini | | --- | --- | --- | --- | --- | --- | --- | --- | |  | GOTERM\_BP\_5 | neuron projection extension involved in neuron projection guidance | **RT** |  | 8 | 2.9E-2 | 3.8E-1 | |  | GOTERM\_BP\_5 | negative regulation of cell morphogenesis involved in differentiation | **RT** |  | 9 | 3.3E-2 | 4.1E-1 | |  | GOTERM\_BP\_5 | regulation of axon guidance | **RT** |  | 9 | 4.9E-2 | 5.1E-1 | |  | GOTERM\_BP\_5 | regulation of axon extension involved in axon guidance | **RT** |  | 6 | 8.9E-2 | 6.9E-1 | |  | GOTERM\_BP\_5 | positive regulation of cell migration | **RT** |  | 5 | 1.3E-1 | 7.9E-1 | |  | GOTERM\_BP\_5 | positive regulation of cell motility | **RT** |  | 5 | 1.3E-1 | 7.9E-1 | |  | GOTERM\_BP\_5 | positive regulation of cellular component movement | **RT** |  | 7 | 1.7E-1 | 8.4E-1 | |  | GOTERM\_BP\_5 | mesenchyme development | **RT** |  | 4 | 1.8E-1 | 8.6E-1 | |  | GOTERM\_BP\_5 | negative regulation of axonogenesis | **RT** |  | 4 | 1.8E-1 | 8.6E-1 | |  | GOTERM\_BP\_5 | semaphorin-plexin signaling pathway | **RT** |  | 4 | 2.6E-1 | 9.3E-1 | |  | GOTERM\_BP\_5 | negative regulation of axon extension involved in axon guidance | **RT** |  | 3 | 2.8E-1 | 9.4E-1 | |  | GOTERM\_BP\_5 | negative regulation of axon guidance | **RT** |  | 3 | 2.8E-1 | 9.4E-1 | |  | GOTERM\_BP\_5 | negative regulation of chemotaxis | **RT** |  | 3 | 2.8E-1 | 9.4E-1 | |  | GOTERM\_BP\_5 | neural crest cell development | **RT** |  | 3 | 2.8E-1 | 9.4E-1 | |  | GOTERM\_BP\_5 | neural crest cell migration | **RT** |  | 3 | 2.8E-1 | 9.4E-1 | |  | GOTERM\_BP\_5 | negative regulation of cell growth | **RT** |  | 6 | 2.9E-1 | 9.4E-1 | |  | GOTERM\_BP\_5 | negative regulation of axon extension | **RT** |  | 3 | 3.9E-1 | 9.8E-1 | |  | GOTERM\_BP\_5 | mesenchymal cell development | **RT** |  | 3 | 3.9E-1 | 9.8E-1 | |  | GOTERM\_BP\_5 | mesenchymal cell differentiation | **RT** |  | 3 | 3.9E-1 | 9.8E-1 | |  | GOTERM\_BP\_5 | negative regulation of cellular component movement | **RT** |  | 3 | 7.2E-1 | 1.0E0 | |  | GOTERM\_BP\_5 | regulation of cell motility | **RT** |  | 7 | 7.4E-1 | 1.0E0 | |  | GOTERM\_BP\_5 | regulation of cell migration | **RT** |  | 7 | 7.4E-1 | 1.0E0 | | Annotation Cluster 62 | | Enrichment Score: 0.65 |  |  | Count | P\_Value | Benjamini | | --- | --- | --- | --- | --- | --- | --- | --- | |  | GOTERM\_BP\_5 | negative regulation of RNA biosynthetic process | **RT** |  | 52 | 8.9E-2 | 6.9E-1 | |  | GOTERM\_BP\_5 | negative regulation of transcription, DNA-templated | **RT** |  | 52 | 8.9E-2 | 6.9E-1 | |  | GOTERM\_BP\_5 | negative regulation of RNA metabolic process | **RT** |  | 55 | 1.1E-1 | 7.5E-1 | |  | GOTERM\_BP\_5 | negative regulation of nucleobase-containing compound metabolic process | **RT** |  | 58 | 1.1E-1 | 7.5E-1 | |  | GOTERM\_BP\_5 | negative regulation of gene expression | **RT** |  | 79 | 1.4E-1 | 8.1E-1 | |  | GOTERM\_BP\_5 | negative regulation of cellular macromolecule biosynthetic process | **RT** |  | 65 | 1.4E-1 | 8.1E-1 | |  | GOTERM\_BP\_5 | negative regulation of macromolecule biosynthetic process | **RT** |  | 65 | 1.4E-1 | 8.1E-1 | |  | GOTERM\_BP\_5 | negative regulation of cellular biosynthetic process | **RT** |  | 66 | 2.0E-1 | 8.8E-1 | |  | GOTERM\_BP\_5 | negative regulation of macromolecule metabolic process | **RT** |  | 92 | 4.4E-1 | 9.9E-1 | |  | GOTERM\_BP\_5 | transcription, DNA-templated | **RT** |  | 134 | 8.1E-1 | 1.0E0 | |  | GOTERM\_BP\_5 | regulation of cellular macromolecule biosynthetic process | **RT** |  | 166 | 8.4E-1 | 1.0E0 | |  | GOTERM\_BP\_5 | regulation of macromolecule biosynthetic process | **RT** |  | 166 | 8.6E-1 | 1.0E0 | | Annotation Cluster 63 | | Enrichment Score: 0.65 |  |  | Count | P\_Value | Benjamini | | --- | --- | --- | --- | --- | --- | --- | --- | |  | GOTERM\_BP\_5 | ovarian fusome organization | **RT** |  | 6 | 3.2E-2 | 4.0E-1 | |  | GOTERM\_BP\_5 | ovarian nurse cell to oocyte transport | **RT** |  | 9 | 3.1E-1 | 9.6E-1 | |  | GOTERM\_BP\_5 | germline ring canal formation | **RT** |  | 5 | 3.1E-1 | 9.6E-1 | |  | GOTERM\_BP\_5 | female germline ring canal formation | **RT** |  | 4 | 4.2E-1 | 9.9E-1 | |  | GOTERM\_BP\_5 | karyosome formation | **RT** |  | 5 | 4.5E-1 | 9.9E-1 | | Annotation Cluster 64 | | Enrichment Score: 0.64 |  |  | Count | P\_Value | Benjamini | | --- | --- | --- | --- | --- | --- | --- | --- | |  | GOTERM\_CC\_5 | type I terminal bouton | **RT** |  | 8 | 5.7E-2 | 5.1E-1 | |  | GOTERM\_CC\_5 | type Is terminal bouton | **RT** |  | 3 | 4.3E-1 | 9.8E-1 | |  | GOTERM\_CC\_5 | type Ib terminal bouton | **RT** |  | 3 | 5.1E-1 | 9.9E-1 | | Annotation Cluster 65 | | Enrichment Score: 0.59 |  |  | Count | P\_Value | Benjamini | | --- | --- | --- | --- | --- | --- | --- | --- | |  | GOTERM\_BP\_5 | positive regulation of secretion | **RT** |  | 11 | 1.6E-1 | 8.4E-1 | |  | GOTERM\_BP\_5 | positive regulation of secretion by cell | **RT** |  | 10 | 2.5E-1 | 9.2E-1 | |  | GOTERM\_BP\_5 | positive regulation of hormone secretion | **RT** |  | 4 | 4.2E-1 | 9.9E-1 | | Annotation Cluster 66 | | Enrichment Score: 0.58 |  |  | Count | P\_Value | Benjamini | | --- | --- | --- | --- | --- | --- | --- | --- | |  | GOTERM\_BP\_5 | leg disc development | **RT** |  | 18 | 2.1E-1 | 8.9E-1 | |  | GOTERM\_BP\_5 | leg disc morphogenesis | **RT** |  | 15 | 2.9E-1 | 9.5E-1 | |  | GOTERM\_BP\_5 | imaginal disc-derived leg morphogenesis | **RT** |  | 15 | 2.9E-1 | 9.5E-1 | | Annotation Cluster 67 | | Enrichment Score: 0.58 |  |  | Count | P\_Value | Benjamini | | --- | --- | --- | --- | --- | --- | --- | --- | |  | GOTERM\_BP\_5 | ion homeostasis | **RT** |  | 25 | 1.2E-1 | 7.8E-1 | |  | GOTERM\_BP\_5 | cellular ion homeostasis | **RT** |  | 19 | 3.3E-1 | 9.6E-1 | |  | GOTERM\_BP\_5 | cellular chemical homeostasis | **RT** |  | 19 | 4.5E-1 | 9.9E-1 | | Annotation Cluster 68 | | Enrichment Score: 0.57 |  |  | Count | P\_Value | Benjamini | | --- | --- | --- | --- | --- | --- | --- | --- | |  | GOTERM\_BP\_5 | exocrine system development | **RT** |  | 36 | 1.4E-1 | 8.1E-1 | |  | GOTERM\_BP\_5 | salivary gland development | **RT** |  | 36 | 1.4E-1 | 8.1E-1 | |  | GOTERM\_BP\_5 | gland development | **RT** |  | 43 | 1.7E-1 | 8.4E-1 | |  | GOTERM\_BP\_5 | salivary gland morphogenesis | **RT** |  | 29 | 2.2E-1 | 9.0E-1 | |  | GOTERM\_BP\_5 | gland morphogenesis | **RT** |  | 29 | 2.2E-1 | 9.0E-1 | |  | GOTERM\_BP\_5 | salivary gland cell autophagic cell death | **RT** |  | 17 | 4.3E-1 | 9.9E-1 | |  | GOTERM\_BP\_5 | autophagic cell death | **RT** |  | 17 | 4.3E-1 | 9.9E-1 | |  | GOTERM\_BP\_5 | salivary gland histolysis | **RT** |  | 17 | 4.7E-1 | 9.9E-1 | |  | GOTERM\_BP\_5 | programmed cell death involved in cell development | **RT** |  | 25 | 5.5E-1 | 1.0E0 | | Annotation Cluster 69 | | Enrichment Score: 0.51 |  |  | Count | P\_Value | Benjamini | | --- | --- | --- | --- | --- | --- | --- | --- | |  | GOTERM\_CC\_5 | integral component of synaptic vesicle membrane | **RT** |  | 4 | 8.6E-2 | 6.4E-1 | |  | GOTERM\_CC\_5 | intrinsic component of synaptic vesicle membrane | **RT** |  | 4 | 8.6E-2 | 6.4E-1 | |  | GOTERM\_CC\_5 | exocytic vesicle membrane | **RT** |  | 5 | 2.4E-1 | 8.9E-1 | |  | GOTERM\_CC\_5 | synaptic vesicle membrane | **RT** |  | 5 | 2.4E-1 | 8.9E-1 | |  | GOTERM\_CC\_5 | cytoplasmic vesicle part | **RT** |  | 12 | 5.5E-1 | 9.9E-1 | |  | GOTERM\_CC\_5 | cytoplasmic vesicle membrane | **RT** |  | 11 | 5.9E-1 | 9.9E-1 | |  | GOTERM\_CC\_5 | transport vesicle membrane | **RT** |  | 6 | 6.9E-1 | 1.0E0 | |  | GOTERM\_CC\_5 | integral component of organelle membrane | **RT** |  | 6 | 9.1E-1 | 1.0E0 | | Annotation Cluster 70 | | Enrichment Score: 0.5 |  |  | Count | P\_Value | Benjamini | | --- | --- | --- | --- | --- | --- | --- | --- | |  | GOTERM\_BP\_5 | regulation of signal transduction | **RT** |  | 145 | 2.7E-2 | 3.7E-1 | |  | GOTERM\_BP\_5 | positive regulation of cell communication | **RT** |  | 60 | 4.8E-1 | 9.9E-1 | |  | GOTERM\_BP\_5 | regulation of intracellular signal transduction | **RT** |  | 63 | 5.7E-1 | 1.0E0 | |  | GOTERM\_BP\_5 | positive regulation of intracellular signal transduction | **RT** |  | 29 | 6.5E-1 | 1.0E0 | |  | GOTERM\_BP\_5 | positive regulation of signal transduction | **RT** |  | 52 | 6.5E-1 | 1.0E0 | | Annotation Cluster 71 | | Enrichment Score: 0.5 |  |  | Count | P\_Value | Benjamini | | --- | --- | --- | --- | --- | --- | --- | --- | |  | GOTERM\_BP\_5 | glial cell development | **RT** |  | 7 | 2.1E-1 | 8.9E-1 | |  | GOTERM\_BP\_5 | response to fibroblast growth factor | **RT** |  | 5 | 3.1E-1 | 9.6E-1 | |  | GOTERM\_BP\_5 | fibroblast growth factor receptor signaling pathway | **RT** |  | 5 | 3.1E-1 | 9.6E-1 | |  | GOTERM\_BP\_5 | cellular response to growth factor stimulus | **RT** |  | 14 | 4.8E-1 | 9.9E-1 | | Annotation Cluster 72 | | Enrichment Score: 0.5 |  |  | Count | P\_Value | Benjamini | | --- | --- | --- | --- | --- | --- | --- | --- | |  | GOTERM\_BP\_5 | positive regulation of hydrolase activity | **RT** |  | 25 | 1.4E-1 | 8.1E-1 | |  | GOTERM\_MF\_5 | GTPase activator activity | **RT** |  | 20 | 4.7E-1 | 9.9E-1 | |  | GOTERM\_BP\_5 | regulation of GTPase activity | **RT** |  | 21 | 4.8E-1 | 9.9E-1 | | Annotation Cluster 73 | | Enrichment Score: 0.49 |  |  | Count | P\_Value | Benjamini | | --- | --- | --- | --- | --- | --- | --- | --- | |  | GOTERM\_BP\_5 | cell-cell junction assembly | **RT** |  | 12 | 1.5E-1 | 8.3E-1 | |  | GOTERM\_BP\_5 | adherens junction organization | **RT** |  | 8 | 3.8E-1 | 9.8E-1 | |  | GOTERM\_BP\_5 | adherens junction assembly | **RT** |  | 4 | 5.7E-1 | 1.0E0 | | Annotation Cluster 74 | | Enrichment Score: 0.48 |  |  | Count | P\_Value | Benjamini | | --- | --- | --- | --- | --- | --- | --- | --- | |  | GOTERM\_BP\_5 | proximal/distal pattern formation | **RT** |  | 5 | 3.1E-1 | 9.6E-1 | |  | GOTERM\_BP\_5 | proximal/distal pattern formation, imaginal disc | **RT** |  | 4 | 3.4E-1 | 9.7E-1 | |  | GOTERM\_BP\_5 | leg disc pattern formation | **RT** |  | 4 | 3.4E-1 | 9.7E-1 | | Annotation Cluster 75 | | Enrichment Score: 0.47 |  |  | Count | P\_Value | Benjamini | | --- | --- | --- | --- | --- | --- | --- | --- | |  | GOTERM\_BP\_5 | positive regulation of neurotransmitter transport | **RT** |  | 3 | 2.8E-1 | 9.4E-1 | |  | GOTERM\_BP\_5 | positive regulation of neurotransmitter secretion | **RT** |  | 3 | 2.8E-1 | 9.4E-1 | |  | GOTERM\_BP\_5 | positive regulation of synaptic transmission | **RT** |  | 4 | 5.0E-1 | 9.9E-1 | | Annotation Cluster 76 | | Enrichment Score: 0.42 |  |  | Count | P\_Value | Benjamini | | --- | --- | --- | --- | --- | --- | --- | --- | |  | GOTERM\_BP\_5 | head segmentation | **RT** |  | 4 | 1.8E-1 | 8.6E-1 | |  | GOTERM\_BP\_5 | specification of segmental identity, head | **RT** |  | 3 | 3.9E-1 | 9.8E-1 | |  | GOTERM\_BP\_5 | segment specification | **RT** |  | 5 | 7.7E-1 | 1.0E0 | | Annotation Cluster 77 | | Enrichment Score: 0.41 |  |  | Count | P\_Value | Benjamini | | --- | --- | --- | --- | --- | --- | --- | --- | |  | GOTERM\_BP\_5 | regulation of heart contraction | **RT** |  | 8 | 2.9E-1 | 9.5E-1 | |  | GOTERM\_BP\_5 | positive regulation of heart contraction | **RT** |  | 3 | 3.9E-1 | 9.8E-1 | |  | GOTERM\_BP\_5 | positive regulation of blood circulation | **RT** |  | 3 | 3.9E-1 | 9.8E-1 | |  | GOTERM\_BP\_5 | heart contraction | **RT** |  | 6 | 5.2E-1 | 9.9E-1 | | Annotation Cluster 78 | | Enrichment Score: 0.4 |  |  | Count | P\_Value | Benjamini | | --- | --- | --- | --- | --- | --- | --- | --- | |  | GOTERM\_BP\_5 | regulation of ERBB signaling pathway | **RT** |  | 14 | 3.8E-1 | 9.8E-1 | |  | GOTERM\_BP\_5 | regulation of epidermal growth factor receptor signaling pathway | **RT** |  | 14 | 3.8E-1 | 9.8E-1 | |  | GOTERM\_BP\_5 | negative regulation of epidermal growth factor receptor signaling pathway | **RT** |  | 10 | 4.1E-1 | 9.8E-1 | |  | GOTERM\_BP\_5 | negative regulation of ERBB signaling pathway | **RT** |  | 10 | 4.1E-1 | 9.8E-1 | | Annotation Cluster 79 | | Enrichment Score: 0.4 |  |  | Count | P\_Value | Benjamini | | --- | --- | --- | --- | --- | --- | --- | --- | |  | GOTERM\_BP\_5 | cilium organization | **RT** |  | 7 | 2.1E-1 | 8.9E-1 | |  | GOTERM\_BP\_5 | cilium assembly | **RT** |  | 6 | 3.4E-1 | 9.7E-1 | |  | GOTERM\_BP\_5 | cilium morphogenesis | **RT** |  | 6 | 3.4E-1 | 9.7E-1 | |  | GOTERM\_BP\_5 | microtubule bundle formation | **RT** |  | 4 | 5.7E-1 | 1.0E0 | |  | GOTERM\_BP\_5 | axoneme assembly | **RT** |  | 3 | 7.2E-1 | 1.0E0 | | Annotation Cluster 80 | | Enrichment Score: 0.39 |  |  | Count | P\_Value | Benjamini | | --- | --- | --- | --- | --- | --- | --- | --- | |  | GOTERM\_BP\_5 | negative regulation of Notch signaling pathway | **RT** |  | 9 | 2.7E-1 | 9.3E-1 | |  | GOTERM\_BP\_5 | Notch signaling pathway | **RT** |  | 24 | 3.2E-1 | 9.6E-1 | |  | GOTERM\_BP\_5 | regulation of Notch signaling pathway | **RT** |  | 18 | 8.0E-1 | 1.0E0 | | Annotation Cluster 81 | | Enrichment Score: 0.37 |  |  | Count | P\_Value | Benjamini | | --- | --- | --- | --- | --- | --- | --- | --- | |  | GOTERM\_MF\_5 | S-acyltransferase activity | **RT** |  | 6 | 2.5E-1 | 9.1E-1 | |  | GOTERM\_MF\_5 | palmitoyltransferase activity | **RT** |  | 5 | 4.7E-1 | 9.9E-1 | |  | GOTERM\_BP\_5 | lipoprotein biosynthetic process | **RT** |  | 8 | 6.6E-1 | 1.0E0 | | Annotation Cluster 82 | | Enrichment Score: 0.37 |  |  | Count | P\_Value | Benjamini | | --- | --- | --- | --- | --- | --- | --- | --- | |  | GOTERM\_BP\_5 | regulation of cytoskeleton organization | **RT** |  | 32 | 4.3E-2 | 4.8E-1 | |  | GOTERM\_BP\_5 | regulation of actin cytoskeleton organization | **RT** |  | 19 | 3.6E-1 | 9.7E-1 | |  | GOTERM\_BP\_5 | regulation of actin filament length | **RT** |  | 11 | 5.8E-1 | 1.0E0 | |  | GOTERM\_BP\_5 | regulation of protein polymerization | **RT** |  | 11 | 5.8E-1 | 1.0E0 | |  | GOTERM\_BP\_5 | regulation of actin polymerization or depolymerization | **RT** |  | 11 | 5.8E-1 | 1.0E0 | |  | GOTERM\_BP\_5 | regulation of actin filament polymerization | **RT** |  | 8 | 8.6E-1 | 1.0E0 | |  | GOTERM\_BP\_5 | actin filament polymerization | **RT** |  | 5 | 9.8E-1 | 1.0E0 | | Annotation Cluster 83 | | Enrichment Score: 0.34 |  |  | Count | P\_Value | Benjamini | | --- | --- | --- | --- | --- | --- | --- | --- | |  | GOTERM\_BP\_5 | tube fusion | **RT** |  | 6 | 2.3E-1 | 9.1E-1 | |  | GOTERM\_BP\_5 | branch fusion, open tracheal system | **RT** |  | 6 | 2.3E-1 | 9.1E-1 | |  | GOTERM\_BP\_5 | branching involved in open tracheal system development | **RT** |  | 11 | 7.1E-1 | 1.0E0 | |  | GOTERM\_BP\_5 | morphogenesis of a branching epithelium | **RT** |  | 11 | 7.1E-1 | 1.0E0 | |  | GOTERM\_BP\_5 | branching morphogenesis of an epithelial tube | **RT** |  | 11 | 7.1E-1 | 1.0E0 | | Annotation Cluster 84 | | Enrichment Score: 0.33 |  |  | Count | P\_Value | Benjamini | | --- | --- | --- | --- | --- | --- | --- | --- | |  | GOTERM\_MF\_5 | sequence-specific DNA binding | **RT** |  | 62 | 3.6E-4 | 1.1E-2 | |  | GOTERM\_BP\_5 | nucleobase-containing compound biosynthetic process | **RT** |  | 182 | 6.1E-1 | 1.0E0 | |  | GOTERM\_BP\_5 | regulation of transcription, DNA-templated | **RT** |  | 148 | 6.3E-1 | 1.0E0 | |  | GOTERM\_BP\_5 | regulation of RNA biosynthetic process | **RT** |  | 148 | 6.4E-1 | 1.0E0 | |  | GOTERM\_BP\_5 | regulation of RNA metabolic process | **RT** |  | 161 | 6.8E-1 | 1.0E0 | |  | GOTERM\_BP\_5 | RNA biosynthetic process | **RT** |  | 154 | 7.3E-1 | 1.0E0 | |  | GOTERM\_BP\_5 | transcription, DNA-templated | **RT** |  | 134 | 8.1E-1 | 1.0E0 | |  | GOTERM\_BP\_5 | regulation of cellular macromolecule biosynthetic process | **RT** |  | 166 | 8.4E-1 | 1.0E0 | |  | GOTERM\_BP\_5 | regulation of macromolecule biosynthetic process | **RT** |  | 166 | 8.6E-1 | 1.0E0 | |  | GOTERM\_BP\_5 | regulation of gene expression | **RT** |  | 185 | 8.7E-1 | 1.0E0 | |  | GOTERM\_BP\_5 | RNA metabolic process | **RT** |  | 203 | 1.0E0 | 1.0E0 | |  | GOTERM\_BP\_5 | nucleic acid metabolic process | **RT** |  | 215 | 1.0E0 | 1.0E0 | |  | GOTERM\_CC\_5 | nucleus | **RT** |  | 261 | 1.0E0 | 1.0E0 | |  | GOTERM\_BP\_5 | cellular macromolecule biosynthetic process | **RT** |  | 213 | 1.0E0 | 1.0E0 | | Annotation Cluster 85 | | Enrichment Score: 0.33 |  |  | Count | P\_Value | Benjamini | | --- | --- | --- | --- | --- | --- | --- | --- | |  | GOTERM\_BP\_5 | regionalization | **RT** |  | 78 | 7.4E-2 | 6.3E-1 | |  | GOTERM\_BP\_5 | establishment or maintenance of microtubule cytoskeleton polarity | **RT** |  | 12 | 1.0E-1 | 7.4E-1 | |  | GOTERM\_BP\_5 | blastoderm segmentation | **RT** |  | 34 | 1.6E-1 | 8.3E-1 | |  | GOTERM\_BP\_5 | embryonic pattern specification | **RT** |  | 38 | 1.7E-1 | 8.4E-1 | |  | GOTERM\_BP\_5 | segmentation | **RT** |  | 40 | 1.8E-1 | 8.5E-1 | |  | GOTERM\_BP\_5 | oocyte microtubule cytoskeleton organization | **RT** |  | 9 | 2.3E-1 | 9.0E-1 | |  | GOTERM\_BP\_5 | establishment or maintenance of cytoskeleton polarity | **RT** |  | 13 | 2.3E-1 | 9.1E-1 | |  | GOTERM\_BP\_5 | anterior/posterior pattern specification | **RT** |  | 26 | 3.1E-1 | 9.6E-1 | |  | GOTERM\_BP\_5 | oocyte microtubule cytoskeleton polarization | **RT** |  | 6 | 4.0E-1 | 9.8E-1 | |  | GOTERM\_BP\_5 | tripartite regional subdivision | **RT** |  | 21 | 4.6E-1 | 9.9E-1 | |  | GOTERM\_BP\_5 | anterior/posterior axis specification | **RT** |  | 21 | 4.6E-1 | 9.9E-1 | |  | GOTERM\_BP\_5 | embryonic axis specification | **RT** |  | 22 | 4.6E-1 | 9.9E-1 | |  | GOTERM\_BP\_5 | cell maturation | **RT** |  | 33 | 5.2E-1 | 9.9E-1 | |  | GOTERM\_BP\_5 | bicoid mRNA localization | **RT** |  | 3 | 7.2E-1 | 1.0E0 | |  | GOTERM\_BP\_5 | axis specification | **RT** |  | 28 | 7.2E-1 | 1.0E0 | |  | GOTERM\_BP\_5 | oocyte anterior/posterior axis specification | **RT** |  | 12 | 8.1E-1 | 1.0E0 | |  | GOTERM\_BP\_5 | oocyte construction | **RT** |  | 18 | 8.5E-1 | 1.0E0 | |  | GOTERM\_BP\_5 | oocyte axis specification | **RT** |  | 16 | 9.2E-1 | 1.0E0 | |  | GOTERM\_BP\_5 | intracellular mRNA localization involved in anterior/posterior axis specification | **RT** |  | 7 | 9.2E-1 | 1.0E0 | |  | GOTERM\_BP\_5 | oocyte development | **RT** |  | 19 | 9.2E-1 | 1.0E0 | |  | GOTERM\_BP\_5 | pole plasm oskar mRNA localization | **RT** |  | 5 | 9.3E-1 | 1.0E0 | |  | GOTERM\_BP\_5 | oocyte differentiation | **RT** |  | 21 | 9.4E-1 | 1.0E0 | |  | GOTERM\_BP\_5 | intracellular mRNA localization involved in pattern specification process | **RT** |  | 7 | 9.4E-1 | 1.0E0 | |  | GOTERM\_BP\_5 | pole plasm assembly | **RT** |  | 7 | 9.6E-1 | 1.0E0 | |  | GOTERM\_BP\_5 | pole plasm RNA localization | **RT** |  | 5 | 9.6E-1 | 1.0E0 | |  | GOTERM\_BP\_5 | pole plasm mRNA localization | **RT** |  | 5 | 9.6E-1 | 1.0E0 | | Annotation Cluster 86 | | Enrichment Score: 0.33 |  |  | Count | P\_Value | Benjamini | | --- | --- | --- | --- | --- | --- | --- | --- | |  | GOTERM\_BP\_5 | establishment of imaginal disc-derived wing hair orientation | **RT** |  | 6 | 4.0E-1 | 9.8E-1 | |  | GOTERM\_BP\_5 | imaginal disc-derived wing hair organization | **RT** |  | 9 | 4.9E-1 | 9.9E-1 | |  | GOTERM\_BP\_5 | epidermal cell differentiation | **RT** |  | 9 | 5.3E-1 | 9.9E-1 | | Annotation Cluster 87 | | Enrichment Score: 0.32 |  |  | Count | P\_Value | Benjamini | | --- | --- | --- | --- | --- | --- | --- | --- | |  | GOTERM\_CC\_5 | basal cortex | **RT** |  | 3 | 2.3E-1 | 8.9E-1 | |  | GOTERM\_BP\_5 | asymmetric stem cell division | **RT** |  | 12 | 4.7E-1 | 9.9E-1 | |  | GOTERM\_BP\_5 | somatic stem cell division | **RT** |  | 9 | 5.7E-1 | 1.0E0 | |  | GOTERM\_BP\_5 | neuroblast division | **RT** |  | 8 | 6.2E-1 | 1.0E0 | |  | GOTERM\_BP\_5 | asymmetric neuroblast division | **RT** |  | 8 | 6.2E-1 | 1.0E0 | | Annotation Cluster 88 | | Enrichment Score: 0.32 |  |  | Count | P\_Value | Benjamini | | --- | --- | --- | --- | --- | --- | --- | --- | |  | GOTERM\_BP\_5 | regulation of microtubule polymerization or depolymerization | **RT** |  | 8 | 4.6E-2 | 4.9E-1 | |  | GOTERM\_BP\_5 | regulation of microtubule polymerization | **RT** |  | 3 | 1.7E-1 | 8.4E-1 | |  | GOTERM\_BP\_5 | positive regulation of microtubule polymerization | **RT** |  | 3 | 1.7E-1 | 8.4E-1 | |  | GOTERM\_BP\_5 | regulation of protein polymerization | **RT** |  | 11 | 5.8E-1 | 1.0E0 | |  | GOTERM\_BP\_5 | positive regulation of protein polymerization | **RT** |  | 6 | 7.1E-1 | 1.0E0 | |  | GOTERM\_BP\_5 | regulation of actin filament polymerization | **RT** |  | 8 | 8.6E-1 | 1.0E0 | |  | GOTERM\_BP\_5 | positive regulation of cytoskeleton organization | **RT** |  | 6 | 8.9E-1 | 1.0E0 | |  | GOTERM\_BP\_5 | positive regulation of protein complex assembly | **RT** |  | 7 | 8.9E-1 | 1.0E0 | |  | GOTERM\_BP\_5 | positive regulation of actin filament polymerization | **RT** |  | 3 | 9.7E-1 | 1.0E0 | |  | GOTERM\_BP\_5 | positive regulation of organelle organization | **RT** |  | 13 | 9.8E-1 | 1.0E0 | |  | GOTERM\_BP\_5 | actin filament polymerization | **RT** |  | 5 | 9.8E-1 | 1.0E0 | | Annotation Cluster 89 | | Enrichment Score: 0.31 |  |  | Count | P\_Value | Benjamini | | --- | --- | --- | --- | --- | --- | --- | --- | |  | GOTERM\_BP\_5 | cellular response to organic cyclic compound | **RT** |  | 14 | 1.6E-1 | 8.4E-1 | |  | GOTERM\_BP\_5 | cellular response to lipid | **RT** |  | 8 | 5.3E-1 | 9.9E-1 | |  | GOTERM\_BP\_5 | hormone-mediated signaling pathway | **RT** |  | 9 | 5.3E-1 | 9.9E-1 | |  | GOTERM\_BP\_5 | cellular response to hormone stimulus | **RT** |  | 17 | 5.9E-1 | 1.0E0 | |  | GOTERM\_BP\_5 | steroid hormone mediated signaling pathway | **RT** |  | 6 | 6.2E-1 | 1.0E0 | |  | GOTERM\_BP\_5 | response to steroid hormone | **RT** |  | 6 | 6.2E-1 | 1.0E0 | |  | GOTERM\_BP\_5 | cellular response to steroid hormone stimulus | **RT** |  | 6 | 6.2E-1 | 1.0E0 | | Annotation Cluster 90 | | Enrichment Score: 0.3 |  |  | Count | P\_Value | Benjamini | | --- | --- | --- | --- | --- | --- | --- | --- | |  | GOTERM\_BP\_5 | heterochromatin organization involved in chromatin silencing | **RT** |  | 4 | 3.4E-1 | 9.7E-1 | |  | GOTERM\_BP\_5 | chromatin organization involved in regulation of transcription | **RT** |  | 4 | 5.0E-1 | 9.9E-1 | |  | GOTERM\_BP\_5 | heterochromatin organization | **RT** |  | 6 | 7.1E-1 | 1.0E0 | | Annotation Cluster 91 | | Enrichment Score: 0.29 |  |  | Count | P\_Value | Benjamini | | --- | --- | --- | --- | --- | --- | --- | --- | |  | GOTERM\_BP\_5 | establishment of protein localization to plasma membrane | **RT** |  | 8 | 1.2E-1 | 7.8E-1 | |  | GOTERM\_BP\_5 | Golgi to plasma membrane protein transport | **RT** |  | 3 | 3.9E-1 | 9.8E-1 | |  | GOTERM\_BP\_5 | protein localization to cell periphery | **RT** |  | 9 | 5.3E-1 | 9.9E-1 | |  | GOTERM\_BP\_5 | protein localization to plasma membrane | **RT** |  | 8 | 5.7E-1 | 1.0E0 | |  | GOTERM\_BP\_5 | establishment of protein localization to membrane | **RT** |  | 11 | 6.1E-1 | 1.0E0 | |  | GOTERM\_BP\_5 | plasma membrane organization | **RT** |  | 10 | 6.8E-1 | 1.0E0 | |  | GOTERM\_BP\_5 | protein localization to membrane | **RT** |  | 14 | 7.7E-1 | 1.0E0 | |  | GOTERM\_BP\_5 | protein targeting to membrane | **RT** |  | 4 | 9.5E-1 | 1.0E0 | | Annotation Cluster 92 | | Enrichment Score: 0.28 |  |  | Count | P\_Value | Benjamini | | --- | --- | --- | --- | --- | --- | --- | --- | |  | GOTERM\_CC\_5 | cation-transporting ATPase complex | **RT** |  | 3 | 2.3E-1 | 8.9E-1 | |  | GOTERM\_CC\_5 | ATPase dependent transmembrane transport complex | **RT** |  | 3 | 2.3E-1 | 8.9E-1 | |  | GOTERM\_CC\_5 | sodium:potassium-exchanging ATPase complex | **RT** |  | 3 | 2.3E-1 | 8.9E-1 | |  | GOTERM\_MF\_5 | active ion transmembrane transporter activity | **RT** |  | 10 | 9.0E-1 | 1.0E0 | |  | GOTERM\_MF\_5 | ATPase coupled ion transmembrane transporter activity | **RT** |  | 6 | 9.9E-1 | 1.0E0 | |  | GOTERM\_MF\_5 | ATPase activity, coupled to transmembrane movement of substances | **RT** |  | 9 | 1.0E0 | 1.0E0 | |  | GOTERM\_MF\_5 | P-P-bond-hydrolysis-driven transmembrane transporter activity | **RT** |  | 10 | 1.0E0 | 1.0E0 | | Annotation Cluster 93 | | Enrichment Score: 0.28 |  |  | Count | P\_Value | Benjamini | | --- | --- | --- | --- | --- | --- | --- | --- | |  | GOTERM\_BP\_5 | positive regulation of cell morphogenesis involved in differentiation | **RT** |  | 9 | 2.3E-1 | 9.0E-1 | |  | GOTERM\_BP\_5 | positive regulation of axon extension | **RT** |  | 3 | 6.6E-1 | 1.0E0 | |  | GOTERM\_BP\_5 | positive regulation of axonogenesis | **RT** |  | 3 | 6.6E-1 | 1.0E0 | |  | GOTERM\_BP\_5 | positive regulation of cell growth | **RT** |  | 7 | 8.0E-1 | 1.0E0 | | Annotation Cluster 94 | | Enrichment Score: 0.27 |  |  | Count | P\_Value | Benjamini | | --- | --- | --- | --- | --- | --- | --- | --- | |  | GOTERM\_BP\_5 | ovarian nurse cell to oocyte transport | **RT** |  | 9 | 3.1E-1 | 9.6E-1 | |  | GOTERM\_BP\_5 | cytoplasmic transport, nurse cell to oocyte | **RT** |  | 4 | 5.7E-1 | 1.0E0 | |  | GOTERM\_BP\_5 | cytosolic transport | **RT** |  | 7 | 8.5E-1 | 1.0E0 | | Annotation Cluster 95 | | Enrichment Score: 0.26 |  |  | Count | P\_Value | Benjamini | | --- | --- | --- | --- | --- | --- | --- | --- | |  | GOTERM\_BP\_5 | adult chitin-containing cuticle pigmentation | **RT** |  | 5 | 3.8E-1 | 9.8E-1 | |  | GOTERM\_BP\_5 | chitin-based cuticle sclerotization | **RT** |  | 6 | 5.7E-1 | 1.0E0 | |  | GOTERM\_BP\_5 | cuticle pigmentation | **RT** |  | 6 | 7.5E-1 | 1.0E0 | | Annotation Cluster 96 | | Enrichment Score: 0.25 |  |  | Count | P\_Value | Benjamini | | --- | --- | --- | --- | --- | --- | --- | --- | |  | GOTERM\_BP\_5 | regulation of neuron death | **RT** |  | 10 | 2.5E-1 | 9.2E-1 | |  | GOTERM\_BP\_5 | negative regulation of neuron death | **RT** |  | 7 | 4.7E-1 | 9.9E-1 | |  | GOTERM\_BP\_5 | negative regulation of neuron apoptotic process | **RT** |  | 4 | 5.7E-1 | 1.0E0 | |  | GOTERM\_BP\_5 | regulation of neuron apoptotic process | **RT** |  | 4 | 6.9E-1 | 1.0E0 | |  | GOTERM\_BP\_5 | neuron apoptotic process | **RT** |  | 4 | 7.4E-1 | 1.0E0 | |  | GOTERM\_BP\_5 | negative regulation of apoptotic process | **RT** |  | 12 | 9.2E-1 | 1.0E0 | | Annotation Cluster 97 | | Enrichment Score: 0.24 |  |  | Count | P\_Value | Benjamini | | --- | --- | --- | --- | --- | --- | --- | --- | |  | GOTERM\_BP\_5 | regulation of cell maturation | **RT** |  | 9 | 3.1E-1 | 9.6E-1 | |  | GOTERM\_BP\_5 | oocyte microtubule cytoskeleton polarization | **RT** |  | 6 | 4.0E-1 | 9.8E-1 | |  | GOTERM\_BP\_5 | regulation of embryonic pattern specification | **RT** |  | 6 | 5.7E-1 | 1.0E0 | |  | GOTERM\_BP\_5 | regulation of oocyte development | **RT** |  | 5 | 7.2E-1 | 1.0E0 | |  | GOTERM\_BP\_5 | regulation of pole plasm oskar mRNA localization | **RT** |  | 5 | 7.2E-1 | 1.0E0 | |  | GOTERM\_BP\_5 | regulation of embryonic development | **RT** |  | 10 | 9.1E-1 | 1.0E0 | | Annotation Cluster 98 | | Enrichment Score: 0.22 |  |  | Count | P\_Value | Benjamini | | --- | --- | --- | --- | --- | --- | --- | --- | |  | GOTERM\_BP\_5 | positive regulation of developmental growth | **RT** |  | 22 | 3.8E-1 | 9.8E-1 | |  | GOTERM\_BP\_5 | positive regulation of multicellular organism growth | **RT** |  | 9 | 4.9E-1 | 9.9E-1 | |  | GOTERM\_BP\_5 | regulation of multicellular organism growth | **RT** |  | 11 | 5.0E-1 | 9.9E-1 | |  | GOTERM\_BP\_5 | positive regulation of cell size | **RT** |  | 5 | 9.0E-1 | 1.0E0 | |  | GOTERM\_BP\_5 | positive regulation of organ growth | **RT** |  | 3 | 9.3E-1 | 1.0E0 | | Annotation Cluster 99 | | Enrichment Score: 0.22 |  |  | Count | P\_Value | Benjamini | | --- | --- | --- | --- | --- | --- | --- | --- | |  | GOTERM\_BP\_5 | tyrosine metabolic process | **RT** |  | 3 | 3.9E-1 | 9.8E-1 | |  | GOTERM\_BP\_5 | aromatic amino acid family metabolic process | **RT** |  | 3 | 6.6E-1 | 1.0E0 | |  | GOTERM\_BP\_5 | alpha-amino acid metabolic process | **RT** |  | 8 | 8.6E-1 | 1.0E0 | | Annotation Cluster 100 | | Enrichment Score: 0.22 |  |  | Count | P\_Value | Benjamini | | --- | --- | --- | --- | --- | --- | --- | --- | |  | GOTERM\_BP\_5 | regulation of imaginal disc growth | **RT** |  | 7 | 5.7E-1 | 1.0E0 | |  | GOTERM\_BP\_5 | negative regulation of imaginal disc growth | **RT** |  | 3 | 5.8E-1 | 1.0E0 | |  | GOTERM\_BP\_5 | imaginal disc growth | **RT** |  | 7 | 6.6E-1 | 1.0E0 | | Annotation Cluster 101 | | Enrichment Score: 0.21 |  |  | Count | P\_Value | Benjamini | | --- | --- | --- | --- | --- | --- | --- | --- | |  | GOTERM\_BP\_5 | glycosaminoglycan metabolic process | **RT** |  | 5 | 5.7E-1 | 1.0E0 | |  | GOTERM\_BP\_5 | proteoglycan metabolic process | **RT** |  | 5 | 5.7E-1 | 1.0E0 | |  | GOTERM\_BP\_5 | aminoglycan biosynthetic process | **RT** |  | 4 | 6.4E-1 | 1.0E0 | |  | GOTERM\_BP\_5 | chondroitin sulfate proteoglycan biosynthetic process | **RT** |  | 3 | 6.6E-1 | 1.0E0 | |  | GOTERM\_BP\_5 | chondroitin sulfate metabolic process | **RT** |  | 3 | 6.6E-1 | 1.0E0 | | Annotation Cluster 102 | | Enrichment Score: 0.2 |  |  | Count | P\_Value | Benjamini | | --- | --- | --- | --- | --- | --- | --- | --- | |  | GOTERM\_BP\_5 | regulation of transmembrane receptor protein serine/threonine kinase signaling pathway | **RT** |  | 11 | 3.4E-1 | 9.7E-1 | |  | GOTERM\_BP\_5 | response to BMP | **RT** |  | 8 | 4.3E-1 | 9.9E-1 | |  | GOTERM\_BP\_5 | cellular response to growth factor stimulus | **RT** |  | 14 | 4.8E-1 | 9.9E-1 | |  | GOTERM\_BP\_5 | BMP signaling pathway | **RT** |  | 7 | 5.7E-1 | 1.0E0 | |  | GOTERM\_BP\_5 | regulation of BMP signaling pathway | **RT** |  | 7 | 6.2E-1 | 1.0E0 | |  | GOTERM\_BP\_5 | transforming growth factor beta receptor signaling pathway | **RT** |  | 4 | 7.9E-1 | 1.0E0 | |  | GOTERM\_BP\_5 | response to transforming growth factor beta | **RT** |  | 4 | 8.6E-1 | 1.0E0 | |  | GOTERM\_BP\_5 | negative regulation of BMP signaling pathway | **RT** |  | 3 | 9.1E-1 | 1.0E0 | |  | GOTERM\_BP\_5 | negative regulation of transmembrane receptor protein serine/threonine kinase signaling pathway | **RT** |  | 3 | 9.6E-1 | 1.0E0 | | Annotation Cluster 103 | | Enrichment Score: 0.2 |  |  | Count | P\_Value | Benjamini | | --- | --- | --- | --- | --- | --- | --- | --- | |  | GOTERM\_BP\_5 | induction of programmed cell death | **RT** |  | 5 | 2.5E-1 | 9.2E-1 | |  | GOTERM\_BP\_5 | positive regulation of cell death | **RT** |  | 14 | 4.5E-1 | 9.9E-1 | |  | GOTERM\_BP\_5 | cellular response to lipid | **RT** |  | 8 | 5.3E-1 | 9.9E-1 | |  | GOTERM\_BP\_5 | positive regulation of programmed cell death | **RT** |  | 12 | 5.4E-1 | 1.0E0 | |  | GOTERM\_BP\_5 | response to sterol | **RT** |  | 8 | 6.9E-1 | 1.0E0 | |  | GOTERM\_BP\_5 | response to ecdysone | **RT** |  | 8 | 6.9E-1 | 1.0E0 | |  | GOTERM\_BP\_5 | positive regulation of apoptotic process | **RT** |  | 8 | 8.2E-1 | 1.0E0 | |  | GOTERM\_BP\_5 | cellular response to alcohol | **RT** |  | 4 | 8.8E-1 | 1.0E0 | |  | GOTERM\_BP\_5 | cellular response to ketone | **RT** |  | 3 | 9.3E-1 | 1.0E0 | |  | GOTERM\_BP\_5 | cellular response to sterol | **RT** |  | 3 | 9.3E-1 | 1.0E0 | | Annotation Cluster 104 | | Enrichment Score: 0.19 |  |  | Count | P\_Value | Benjamini | | --- | --- | --- | --- | --- | --- | --- | --- | |  | GOTERM\_CC\_5 | microtubule plus-end | **RT** |  | 4 | 2.8E-1 | 9.2E-1 | |  | GOTERM\_CC\_5 | microtubule end | **RT** |  | 4 | 2.8E-1 | 9.2E-1 | |  | GOTERM\_BP\_5 | mitotic spindle elongation | **RT** |  | 4 | 3.4E-1 | 9.7E-1 | |  | GOTERM\_BP\_5 | spindle elongation | **RT** |  | 4 | 3.4E-1 | 9.7E-1 | |  | GOTERM\_CC\_5 | spindle microtubule | **RT** |  | 6 | 5.1E-1 | 9.9E-1 | |  | GOTERM\_BP\_5 | mitotic spindle organization | **RT** |  | 8 | 6.2E-1 | 1.0E0 | |  | GOTERM\_BP\_5 | establishment of mitotic spindle localization | **RT** |  | 5 | 7.2E-1 | 1.0E0 | |  | GOTERM\_BP\_5 | mitotic spindle assembly | **RT** |  | 4 | 7.4E-1 | 1.0E0 | |  | GOTERM\_BP\_5 | microtubule cytoskeleton organization involved in mitosis | **RT** |  | 4 | 7.4E-1 | 1.0E0 | |  | GOTERM\_BP\_5 | spindle assembly | **RT** |  | 7 | 8.3E-1 | 1.0E0 | |  | GOTERM\_BP\_5 | establishment of spindle localization | **RT** |  | 5 | 8.6E-1 | 1.0E0 | |  | GOTERM\_BP\_5 | spindle localization | **RT** |  | 5 | 8.6E-1 | 1.0E0 | |  | GOTERM\_BP\_5 | establishment of mitotic spindle orientation | **RT** |  | 3 | 8.9E-1 | 1.0E0 | |  | GOTERM\_BP\_5 | spindle organization | **RT** |  | 8 | 9.1E-1 | 1.0E0 | |  | GOTERM\_BP\_5 | sister chromatid segregation | **RT** |  | 12 | 9.3E-1 | 1.0E0 | |  | GOTERM\_BP\_5 | establishment of spindle orientation | **RT** |  | 3 | 9.6E-1 | 1.0E0 | |  | GOTERM\_BP\_5 | mitotic sister chromatid segregation | **RT** |  | 9 | 9.8E-1 | 1.0E0 | | Annotation Cluster 105 | | Enrichment Score: 0.18 |  |  | Count | P\_Value | Benjamini | | --- | --- | --- | --- | --- | --- | --- | --- | |  | GOTERM\_BP\_5 | regulation of insulin receptor signaling pathway | **RT** |  | 6 | 4.0E-1 | 9.8E-1 | |  | GOTERM\_BP\_5 | negative regulation of insulin receptor signaling pathway | **RT** |  | 4 | 5.0E-1 | 9.9E-1 | |  | GOTERM\_BP\_5 | cellular response to hormone stimulus | **RT** |  | 17 | 5.9E-1 | 1.0E0 | |  | GOTERM\_BP\_5 | cellular response to peptide | **RT** |  | 8 | 7.3E-1 | 1.0E0 | |  | GOTERM\_BP\_5 | cellular response to peptide hormone stimulus | **RT** |  | 8 | 7.3E-1 | 1.0E0 | |  | GOTERM\_BP\_5 | cellular response to organonitrogen compound | **RT** |  | 10 | 7.5E-1 | 1.0E0 | |  | GOTERM\_BP\_5 | response to insulin | **RT** |  | 8 | 7.6E-1 | 1.0E0 | |  | GOTERM\_BP\_5 | response to peptide | **RT** |  | 8 | 7.9E-1 | 1.0E0 | |  | GOTERM\_BP\_5 | response to peptide hormone | **RT** |  | 8 | 7.9E-1 | 1.0E0 | | Annotation Cluster 106 | | Enrichment Score: 0.18 |  |  | Count | P\_Value | Benjamini | | --- | --- | --- | --- | --- | --- | --- | --- | |  | GOTERM\_BP\_5 | phospholipid dephosphorylation | **RT** |  | 6 | 3.4E-1 | 9.7E-1 | |  | GOTERM\_BP\_5 | lipid modification | **RT** |  | 9 | 8.5E-1 | 1.0E0 | |  | GOTERM\_BP\_5 | phospholipid metabolic process | **RT** |  | 12 | 9.6E-1 | 1.0E0 | | Annotation Cluster 107 | | Enrichment Score: 0.17 |  |  | Count | P\_Value | Benjamini | | --- | --- | --- | --- | --- | --- | --- | --- | |  | GOTERM\_BP\_5 | regulation of gene silencing by RNA | **RT** |  | 5 | 6.3E-1 | 1.0E0 | |  | GOTERM\_BP\_5 | regulation of gene silencing by miRNA | **RT** |  | 3 | 6.6E-1 | 1.0E0 | |  | GOTERM\_BP\_5 | regulation of posttranscriptional gene silencing | **RT** |  | 3 | 6.6E-1 | 1.0E0 | |  | GOTERM\_BP\_5 | posttranscriptional gene silencing by RNA | **RT** |  | 9 | 7.5E-1 | 1.0E0 | | Annotation Cluster 108 | | Enrichment Score: 0.17 |  |  | Count | P\_Value | Benjamini | | --- | --- | --- | --- | --- | --- | --- | --- | |  | GOTERM\_BP\_5 | cell migration | **RT** |  | 58 | 7.2E-2 | 6.3E-1 | |  | GOTERM\_BP\_5 | epithelial cell differentiation | **RT** |  | 57 | 5.4E-1 | 1.0E0 | |  | GOTERM\_BP\_5 | ameboidal-type cell migration | **RT** |  | 31 | 5.8E-1 | 1.0E0 | |  | GOTERM\_BP\_5 | epithelial cell development | **RT** |  | 47 | 8.1E-1 | 1.0E0 | |  | GOTERM\_BP\_5 | gamete generation | **RT** |  | 120 | 8.4E-1 | 1.0E0 | |  | GOTERM\_BP\_5 | columnar/cuboidal epithelial cell development | **RT** |  | 43 | 8.5E-1 | 1.0E0 | |  | GOTERM\_BP\_5 | ovarian follicle cell development | **RT** |  | 43 | 8.5E-1 | 1.0E0 | |  | GOTERM\_BP\_5 | epithelial cell migration | **RT** |  | 24 | 8.7E-1 | 1.0E0 | |  | GOTERM\_BP\_5 | ovarian follicle cell migration | **RT** |  | 20 | 8.8E-1 | 1.0E0 | |  | GOTERM\_BP\_5 | female gamete generation | **RT** |  | 93 | 9.1E-1 | 1.0E0 | |  | GOTERM\_BP\_5 | border follicle cell migration | **RT** |  | 18 | 9.2E-1 | 1.0E0 | |  | GOTERM\_BP\_5 | germ cell development | **RT** |  | 101 | 9.4E-1 | 1.0E0 | |  | GOTERM\_BP\_5 | oogenesis | **RT** |  | 89 | 9.5E-1 | 1.0E0 | | Annotation Cluster 109 | | Enrichment Score: 0.17 |  |  | Count | P\_Value | Benjamini | | --- | --- | --- | --- | --- | --- | --- | --- | |  | GOTERM\_BP\_5 | regulation of protein localization to nucleus | **RT** |  | 8 | 2.0E-1 | 8.8E-1 | |  | GOTERM\_BP\_5 | regulation of cellular protein localization | **RT** |  | 14 | 2.8E-1 | 9.4E-1 | |  | GOTERM\_BP\_5 | regulation of nucleocytoplasmic transport | **RT** |  | 8 | 4.3E-1 | 9.9E-1 | |  | GOTERM\_BP\_5 | regulation of intracellular protein transport | **RT** |  | 9 | 4.4E-1 | 9.9E-1 | |  | GOTERM\_BP\_5 | regulation of protein import | **RT** |  | 6 | 4.6E-1 | 9.9E-1 | |  | GOTERM\_BP\_5 | regulation of intracellular transport | **RT** |  | 11 | 4.6E-1 | 9.9E-1 | |  | GOTERM\_BP\_5 | regulation of establishment of protein localization | **RT** |  | 15 | 4.9E-1 | 9.9E-1 | |  | GOTERM\_BP\_5 | positive regulation of cellular protein localization | **RT** |  | 7 | 5.2E-1 | 9.9E-1 | |  | GOTERM\_BP\_5 | regulation of protein transport | **RT** |  | 13 | 6.1E-1 | 1.0E0 | |  | GOTERM\_BP\_5 | positive regulation of nucleocytoplasmic transport | **RT** |  | 4 | 6.4E-1 | 1.0E0 | |  | GOTERM\_BP\_5 | positive regulation of establishment of protein localization | **RT** |  | 8 | 6.6E-1 | 1.0E0 | |  | GOTERM\_BP\_5 | positive regulation of intracellular transport | **RT** |  | 5 | 7.7E-1 | 1.0E0 | |  | GOTERM\_BP\_5 | positive regulation of protein transport | **RT** |  | 7 | 7.7E-1 | 1.0E0 | |  | GOTERM\_BP\_5 | positive regulation of intracellular protein transport | **RT** |  | 4 | 8.3E-1 | 1.0E0 | |  | GOTERM\_BP\_5 | single-organism nuclear import | **RT** |  | 8 | 8.6E-1 | 1.0E0 | |  | GOTERM\_BP\_5 | protein import into nucleus | **RT** |  | 8 | 8.6E-1 | 1.0E0 | |  | GOTERM\_BP\_5 | protein targeting to nucleus | **RT** |  | 8 | 8.6E-1 | 1.0E0 | |  | GOTERM\_BP\_5 | nucleocytoplasmic transport | **RT** |  | 14 | 9.6E-1 | 1.0E0 | |  | GOTERM\_BP\_5 | nuclear transport | **RT** |  | 14 | 9.6E-1 | 1.0E0 | |  | GOTERM\_BP\_5 | protein import | **RT** |  | 11 | 9.8E-1 | 1.0E0 | |  | GOTERM\_BP\_5 | single-organism intracellular transport | **RT** |  | 21 | 1.0E0 | 1.0E0 | |  | GOTERM\_BP\_5 | protein targeting | **RT** |  | 17 | 1.0E0 | 1.0E0 | |  | GOTERM\_BP\_5 | establishment of protein localization to organelle | **RT** |  | 13 | 1.0E0 | 1.0E0 | |  | GOTERM\_BP\_5 | protein localization to organelle | **RT** |  | 18 | 1.0E0 | 1.0E0 | |  | GOTERM\_BP\_5 | protein transport | **RT** |  | 47 | 1.0E0 | 1.0E0 | |  | GOTERM\_BP\_5 | intracellular protein transport | **RT** |  | 30 | 1.0E0 | 1.0E0 | | Annotation Cluster 110 | | Enrichment Score: 0.16 |  |  | Count | P\_Value | Benjamini | | --- | --- | --- | --- | --- | --- | --- | --- | |  | GOTERM\_BP\_5 | imaginal disc-derived leg joint morphogenesis | **RT** |  | 3 | 4.9E-1 | 9.9E-1 | |  | GOTERM\_BP\_5 | appendage segmentation | **RT** |  | 3 | 8.2E-1 | 1.0E0 | |  | GOTERM\_BP\_5 | imaginal disc-derived leg segmentation | **RT** |  | 3 | 8.2E-1 | 1.0E0 | | Annotation Cluster 111 | | Enrichment Score: 0.16 |  |  | Count | P\_Value | Benjamini | | --- | --- | --- | --- | --- | --- | --- | --- | |  | GOTERM\_BP\_5 | regulation of insulin secretion | **RT** |  | 3 | 3.9E-1 | 9.8E-1 | |  | GOTERM\_BP\_5 | regulation of protein secretion | **RT** |  | 5 | 7.7E-1 | 1.0E0 | |  | GOTERM\_BP\_5 | positive regulation of protein transport | **RT** |  | 7 | 7.7E-1 | 1.0E0 | |  | GOTERM\_BP\_5 | protein secretion | **RT** |  | 9 | 8.3E-1 | 1.0E0 | |  | GOTERM\_BP\_5 | positive regulation of protein secretion | **RT** |  | 3 | 8.6E-1 | 1.0E0 | | Annotation Cluster 112 | | Enrichment Score: 0.16 |  |  | Count | P\_Value | Benjamini | | --- | --- | --- | --- | --- | --- | --- | --- | |  | GOTERM\_BP\_5 | genital disc development | **RT** |  | 7 | 4.7E-1 | 9.9E-1 | |  | GOTERM\_BP\_5 | imaginal disc-derived male genitalia development | **RT** |  | 5 | 5.1E-1 | 9.9E-1 | |  | GOTERM\_BP\_5 | imaginal disc-derived genitalia development | **RT** |  | 5 | 5.7E-1 | 1.0E0 | |  | GOTERM\_BP\_5 | genitalia development | **RT** |  | 5 | 6.3E-1 | 1.0E0 | |  | GOTERM\_BP\_5 | male sex differentiation | **RT** |  | 4 | 6.4E-1 | 1.0E0 | |  | GOTERM\_BP\_5 | male genitalia morphogenesis | **RT** |  | 3 | 7.8E-1 | 1.0E0 | |  | GOTERM\_BP\_5 | male anatomical structure morphogenesis | **RT** |  | 3 | 7.8E-1 | 1.0E0 | |  | GOTERM\_BP\_5 | male genitalia development | **RT** |  | 3 | 7.8E-1 | 1.0E0 | |  | GOTERM\_BP\_5 | imaginal disc-derived male genitalia morphogenesis | **RT** |  | 3 | 7.8E-1 | 1.0E0 | |  | GOTERM\_BP\_5 | post-embryonic genitalia morphogenesis | **RT** |  | 3 | 8.2E-1 | 1.0E0 | |  | GOTERM\_BP\_5 | genitalia morphogenesis | **RT** |  | 3 | 8.2E-1 | 1.0E0 | |  | GOTERM\_BP\_5 | imaginal disc-derived genitalia morphogenesis | **RT** |  | 3 | 8.2E-1 | 1.0E0 | |  | GOTERM\_BP\_5 | genital disc morphogenesis | **RT** |  | 3 | 8.6E-1 | 1.0E0 | | Annotation Cluster 113 | | Enrichment Score: 0.15 |  |  | Count | P\_Value | Benjamini | | --- | --- | --- | --- | --- | --- | --- | --- | |  | GOTERM\_BP\_5 | intrinsic apoptotic signaling pathway | **RT** |  | 5 | 6.8E-1 | 1.0E0 | |  | GOTERM\_BP\_5 | intrinsic apoptotic signaling pathway in response to DNA damage | **RT** |  | 3 | 7.2E-1 | 1.0E0 | |  | GOTERM\_BP\_5 | apoptotic signaling pathway | **RT** |  | 7 | 7.4E-1 | 1.0E0 | | Annotation Cluster 114 | | Enrichment Score: 0.14 |  |  | Count | P\_Value | Benjamini | | --- | --- | --- | --- | --- | --- | --- | --- | |  | GOTERM\_BP\_5 | regulation of hippo signaling | **RT** |  | 6 | 5.7E-1 | 1.0E0 | |  | GOTERM\_BP\_5 | hippo signaling | **RT** |  | 6 | 7.8E-1 | 1.0E0 | |  | GOTERM\_BP\_5 | positive regulation of hippo signaling | **RT** |  | 3 | 8.2E-1 | 1.0E0 | | Annotation Cluster 115 | | Enrichment Score: 0.14 |  |  | Count | P\_Value | Benjamini | | --- | --- | --- | --- | --- | --- | --- | --- | |  | GOTERM\_BP\_5 | regulation of proteolysis | **RT** |  | 16 | 7.2E-1 | 1.0E0 | |  | GOTERM\_BP\_5 | positive regulation of proteolysis | **RT** |  | 9 | 7.2E-1 | 1.0E0 | |  | GOTERM\_BP\_5 | regulation of peptidase activity | **RT** |  | 7 | 7.4E-1 | 1.0E0 | | Annotation Cluster 116 | | Enrichment Score: 0.13 |  |  | Count | P\_Value | Benjamini | | --- | --- | --- | --- | --- | --- | --- | --- | |  | GOTERM\_BP\_5 | embryonic organ development | **RT** |  | 7 | 5.7E-1 | 1.0E0 | |  | GOTERM\_BP\_5 | embryonic hemopoiesis | **RT** |  | 3 | 8.6E-1 | 1.0E0 | |  | GOTERM\_BP\_5 | hemocyte migration | **RT** |  | 3 | 8.6E-1 | 1.0E0 | | Annotation Cluster 117 | | Enrichment Score: 0.12 |  |  | Count | P\_Value | Benjamini | | --- | --- | --- | --- | --- | --- | --- | --- | |  | GOTERM\_BP\_5 | organelle disassembly | **RT** |  | 5 | 6.8E-1 | 1.0E0 | |  | GOTERM\_BP\_5 | mitochondrion disassembly | **RT** |  | 4 | 7.9E-1 | 1.0E0 | |  | GOTERM\_BP\_5 | mitophagy | **RT** |  | 4 | 7.9E-1 | 1.0E0 | | Annotation Cluster 118 | | Enrichment Score: 0.12 |  |  | Count | P\_Value | Benjamini | | --- | --- | --- | --- | --- | --- | --- | --- | |  | GOTERM\_BP\_5 | glycosphingolipid metabolic process | **RT** |  | 3 | 4.9E-1 | 9.9E-1 | |  | GOTERM\_BP\_5 | glycolipid biosynthetic process | **RT** |  | 5 | 6.3E-1 | 1.0E0 | |  | GOTERM\_BP\_5 | glycolipid metabolic process | **RT** |  | 5 | 6.3E-1 | 1.0E0 | |  | GOTERM\_BP\_5 | membrane lipid biosynthetic process | **RT** |  | 5 | 9.0E-1 | 1.0E0 | |  | GOTERM\_BP\_5 | sphingolipid biosynthetic process | **RT** |  | 3 | 9.1E-1 | 1.0E0 | |  | GOTERM\_BP\_5 | sphingolipid metabolic process | **RT** |  | 4 | 9.4E-1 | 1.0E0 | |  | GOTERM\_BP\_5 | membrane lipid metabolic process | **RT** |  | 6 | 9.4E-1 | 1.0E0 | | Annotation Cluster 119 | | Enrichment Score: 0.12 |  |  | Count | P\_Value | Benjamini | | --- | --- | --- | --- | --- | --- | --- | --- | |  | GOTERM\_BP\_5 | melanotic encapsulation of foreign target | **RT** |  | 6 | 6.7E-1 | 1.0E0 | |  | GOTERM\_BP\_5 | melanin metabolic process | **RT** |  | 7 | 7.7E-1 | 1.0E0 | |  | GOTERM\_BP\_5 | melanization defense response | **RT** |  | 6 | 8.4E-1 | 1.0E0 | | Annotation Cluster 120 | | Enrichment Score: 0.12 |  |  | Count | P\_Value | Benjamini | | --- | --- | --- | --- | --- | --- | --- | --- | |  | GOTERM\_BP\_5 | DNA endoreduplication | **RT** |  | 4 | 6.9E-1 | 1.0E0 | |  | GOTERM\_BP\_5 | cell cycle DNA replication | **RT** |  | 4 | 7.4E-1 | 1.0E0 | |  | GOTERM\_BP\_5 | DNA replication | **RT** |  | 7 | 8.5E-1 | 1.0E0 | | Annotation Cluster 121 | | Enrichment Score: 0.12 |  |  | Count | P\_Value | Benjamini | | --- | --- | --- | --- | --- | --- | --- | --- | |  | GOTERM\_CC\_5 | myofibril | **RT** |  | 7 | 7.0E-1 | 1.0E0 | |  | GOTERM\_CC\_5 | contractile fiber | **RT** |  | 7 | 7.3E-1 | 1.0E0 | |  | GOTERM\_CC\_5 | Z disc | **RT** |  | 5 | 7.6E-1 | 1.0E0 | |  | GOTERM\_CC\_5 | I band | **RT** |  | 5 | 7.6E-1 | 1.0E0 | |  | GOTERM\_CC\_5 | sarcomere | **RT** |  | 6 | 7.9E-1 | 1.0E0 | |  | GOTERM\_CC\_5 | contractile fiber part | **RT** |  | 6 | 8.4E-1 | 1.0E0 | | Annotation Cluster 122 | | Enrichment Score: 0.12 |  |  | Count | P\_Value | Benjamini | | --- | --- | --- | --- | --- | --- | --- | --- | |  | GOTERM\_MF\_5 | translation repressor activity, nucleic acid binding | **RT** |  | 5 | 1.4E-1 | 8.0E-1 | |  | GOTERM\_BP\_5 | negative regulation of translation | **RT** |  | 15 | 6.1E-1 | 1.0E0 | |  | GOTERM\_BP\_5 | negative regulation of cellular amide metabolic process | **RT** |  | 15 | 7.2E-1 | 1.0E0 | |  | GOTERM\_BP\_5 | posttranscriptional regulation of gene expression | **RT** |  | 25 | 9.4E-1 | 1.0E0 | |  | GOTERM\_BP\_5 | regulation of translation | **RT** |  | 22 | 9.6E-1 | 1.0E0 | |  | GOTERM\_BP\_5 | negative regulation of cellular protein metabolic process | **RT** |  | 26 | 9.7E-1 | 1.0E0 | |  | GOTERM\_BP\_5 | negative regulation of protein metabolic process | **RT** |  | 26 | 9.8E-1 | 1.0E0 | |  | GOTERM\_BP\_5 | peptide metabolic process | **RT** |  | 45 | 1.0E0 | 1.0E0 | |  | GOTERM\_BP\_5 | peptide biosynthetic process | **RT** |  | 34 | 1.0E0 | 1.0E0 | |  | GOTERM\_BP\_5 | translation | **RT** |  | 31 | 1.0E0 | 1.0E0 | |  | GOTERM\_BP\_5 | amide biosynthetic process | **RT** |  | 34 | 1.0E0 | 1.0E0 | | Annotation Cluster 123 | | Enrichment Score: 0.11 |  |  | Count | P\_Value | Benjamini | | --- | --- | --- | --- | --- | --- | --- | --- | |  | GOTERM\_BP\_5 | positive regulation of stress-activated protein kinase signaling cascade | **RT** |  | 6 | 4.6E-1 | 9.9E-1 | |  | GOTERM\_BP\_5 | positive regulation of MAPK cascade | **RT** |  | 14 | 7.5E-1 | 1.0E0 | |  | GOTERM\_BP\_5 | stress-activated MAPK cascade | **RT** |  | 13 | 7.6E-1 | 1.0E0 | |  | GOTERM\_BP\_5 | MAPK cascade | **RT** |  | 25 | 7.6E-1 | 1.0E0 | |  | GOTERM\_BP\_5 | signal transduction by protein phosphorylation | **RT** |  | 25 | 7.6E-1 | 1.0E0 | |  | GOTERM\_BP\_5 | stress-activated protein kinase signaling cascade | **RT** |  | 13 | 8.0E-1 | 1.0E0 | |  | GOTERM\_BP\_5 | regulation of stress-activated MAPK cascade | **RT** |  | 8 | 9.1E-1 | 1.0E0 | |  | GOTERM\_BP\_5 | regulation of stress-activated protein kinase signaling cascade | **RT** |  | 8 | 9.1E-1 | 1.0E0 | |  | GOTERM\_BP\_5 | regulation of MAPK cascade | **RT** |  | 18 | 9.4E-1 | 1.0E0 | | Annotation Cluster 124 | | Enrichment Score: 0.11 |  |  | Count | P\_Value | Benjamini | | --- | --- | --- | --- | --- | --- | --- | --- | |  | GOTERM\_BP\_5 | posttranscriptional gene silencing by RNA | **RT** |  | 9 | 7.5E-1 | 1.0E0 | |  | GOTERM\_BP\_5 | response to dsRNA | **RT** |  | 3 | 7.8E-1 | 1.0E0 | |  | GOTERM\_BP\_5 | production of small RNA involved in gene silencing by RNA | **RT** |  | 3 | 7.8E-1 | 1.0E0 | |  | GOTERM\_BP\_5 | cellular response to dsRNA | **RT** |  | 3 | 7.8E-1 | 1.0E0 | | Annotation Cluster 125 | | Enrichment Score: 0.11 |  |  | Count | P\_Value | Benjamini | | --- | --- | --- | --- | --- | --- | --- | --- | |  | GOTERM\_BP\_5 | establishment of monopolar cell polarity | **RT** |  | 3 | 6.6E-1 | 1.0E0 | |  | GOTERM\_BP\_5 | establishment of epithelial cell polarity | **RT** |  | 3 | 7.2E-1 | 1.0E0 | |  | GOTERM\_BP\_5 | establishment or maintenance of apical/basal cell polarity | **RT** |  | 4 | 9.7E-1 | 1.0E0 | | Annotation Cluster 126 | | Enrichment Score: 0.11 |  |  | Count | P\_Value | Benjamini | | --- | --- | --- | --- | --- | --- | --- | --- | |  | GOTERM\_BP\_5 | positive regulation of phosphate metabolic process | **RT** |  | 29 | 3.7E-1 | 9.8E-1 | |  | GOTERM\_BP\_5 | positive regulation of phosphorus metabolic process | **RT** |  | 29 | 3.7E-1 | 9.8E-1 | |  | GOTERM\_BP\_5 | positive regulation of intracellular signal transduction | **RT** |  | 29 | 6.5E-1 | 1.0E0 | |  | GOTERM\_BP\_5 | positive regulation of MAPK cascade | **RT** |  | 14 | 7.5E-1 | 1.0E0 | |  | GOTERM\_BP\_5 | MAPK cascade | **RT** |  | 25 | 7.6E-1 | 1.0E0 | |  | GOTERM\_BP\_5 | signal transduction by protein phosphorylation | **RT** |  | 25 | 7.6E-1 | 1.0E0 | |  | GOTERM\_BP\_5 | regulation of phosphate metabolic process | **RT** |  | 43 | 8.8E-1 | 1.0E0 | |  | GOTERM\_BP\_5 | positive regulation of protein metabolic process | **RT** |  | 38 | 8.8E-1 | 1.0E0 | |  | GOTERM\_BP\_5 | positive regulation of cellular protein metabolic process | **RT** |  | 35 | 9.1E-1 | 1.0E0 | |  | GOTERM\_BP\_5 | positive regulation of protein modification process | **RT** |  | 22 | 9.3E-1 | 1.0E0 | |  | GOTERM\_BP\_5 | ERK1 and ERK2 cascade | **RT** |  | 6 | 9.3E-1 | 1.0E0 | |  | GOTERM\_BP\_5 | regulation of MAPK cascade | **RT** |  | 18 | 9.4E-1 | 1.0E0 | |  | GOTERM\_BP\_5 | regulation of protein metabolic process | **RT** |  | 74 | 1.0E0 | 1.0E0 | |  | GOTERM\_BP\_5 | regulation of protein modification process | **RT** |  | 33 | 1.0E0 | 1.0E0 | |  | GOTERM\_BP\_5 | regulation of cellular protein metabolic process | **RT** |  | 65 | 1.0E0 | 1.0E0 | | Annotation Cluster 127 | | Enrichment Score: 0.11 |  |  | Count | P\_Value | Benjamini | | --- | --- | --- | --- | --- | --- | --- | --- | |  | GOTERM\_BP\_5 | germarium-derived oocyte differentiation | **RT** |  | 6 | 6.7E-1 | 1.0E0 | |  | GOTERM\_BP\_5 | oocyte fate determination | **RT** |  | 6 | 6.7E-1 | 1.0E0 | |  | GOTERM\_BP\_5 | germarium-derived oocyte fate determination | **RT** |  | 5 | 7.7E-1 | 1.0E0 | |  | GOTERM\_BP\_5 | germ-line cyst formation | **RT** |  | 5 | 8.6E-1 | 1.0E0 | |  | GOTERM\_BP\_5 | oocyte dorsal/ventral axis specification | **RT** |  | 4 | 9.7E-1 | 1.0E0 | | Annotation Cluster 128 | | Enrichment Score: 0.11 |  |  | Count | P\_Value | Benjamini | | --- | --- | --- | --- | --- | --- | --- | --- | |  | GOTERM\_BP\_5 | negative regulation of innate immune response | **RT** |  | 5 | 5.7E-1 | 1.0E0 | |  | GOTERM\_BP\_5 | defense response to bacterium | **RT** |  | 20 | 7.3E-1 | 1.0E0 | |  | GOTERM\_BP\_5 | response to bacterium | **RT** |  | 21 | 7.9E-1 | 1.0E0 | |  | GOTERM\_BP\_5 | negative regulation of defense response | **RT** |  | 5 | 8.0E-1 | 1.0E0 | |  | GOTERM\_BP\_5 | defense response to other organism | **RT** |  | 24 | 8.6E-1 | 1.0E0 | |  | GOTERM\_BP\_5 | regulation of innate immune response | **RT** |  | 12 | 8.6E-1 | 1.0E0 | |  | GOTERM\_BP\_5 | defense response to Gram-negative bacterium | **RT** |  | 10 | 8.9E-1 | 1.0E0 | | Annotation Cluster 129 | | Enrichment Score: 0.1 |  |  | Count | P\_Value | Benjamini | | --- | --- | --- | --- | --- | --- | --- | --- | |  | GOTERM\_BP\_5 | rhodopsin metabolic process | **RT** |  | 3 | 6.6E-1 | 1.0E0 | |  | GOTERM\_BP\_5 | eye pigment metabolic process | **RT** |  | 5 | 8.0E-1 | 1.0E0 | |  | GOTERM\_BP\_5 | pigment metabolic process involved in developmental pigmentation | **RT** |  | 5 | 8.0E-1 | 1.0E0 | |  | GOTERM\_BP\_5 | eye pigment biosynthetic process | **RT** |  | 4 | 9.0E-1 | 1.0E0 | | Annotation Cluster 130 | | Enrichment Score: 0.1 |  |  | Count | P\_Value | Benjamini | | --- | --- | --- | --- | --- | --- | --- | --- | |  | GOTERM\_BP\_5 | antimicrobial humoral response | **RT** |  | 13 | 5.5E-1 | 1.0E0 | |  | GOTERM\_BP\_5 | positive regulation of antibacterial peptide biosynthetic process | **RT** |  | 3 | 6.6E-1 | 1.0E0 | |  | GOTERM\_BP\_5 | positive regulation of antibacterial peptide production | **RT** |  | 3 | 6.6E-1 | 1.0E0 | |  | GOTERM\_BP\_5 | positive regulation of defense response to bacterium | **RT** |  | 3 | 7.2E-1 | 1.0E0 | |  | GOTERM\_BP\_5 | regulation of antibacterial peptide biosynthetic process | **RT** |  | 3 | 7.8E-1 | 1.0E0 | |  | GOTERM\_BP\_5 | antibacterial peptide biosynthetic process | **RT** |  | 3 | 7.8E-1 | 1.0E0 | |  | GOTERM\_BP\_5 | antibacterial peptide production | **RT** |  | 3 | 7.8E-1 | 1.0E0 | |  | GOTERM\_BP\_5 | regulation of antibacterial peptide production | **RT** |  | 3 | 7.8E-1 | 1.0E0 | |  | GOTERM\_BP\_5 | regulation of antimicrobial humoral response | **RT** |  | 6 | 7.8E-1 | 1.0E0 | |  | GOTERM\_BP\_5 | positive regulation of antimicrobial peptide biosynthetic process | **RT** |  | 3 | 8.2E-1 | 1.0E0 | |  | GOTERM\_BP\_5 | regulation of defense response to bacterium | **RT** |  | 4 | 8.3E-1 | 1.0E0 | |  | GOTERM\_BP\_5 | regulation of antimicrobial peptide production | **RT** |  | 5 | 8.3E-1 | 1.0E0 | |  | GOTERM\_BP\_5 | Toll signaling pathway | **RT** |  | 4 | 8.6E-1 | 1.0E0 | |  | GOTERM\_BP\_5 | positive regulation of humoral immune response | **RT** |  | 3 | 8.6E-1 | 1.0E0 | |  | GOTERM\_BP\_5 | positive regulation of antimicrobial peptide production | **RT** |  | 3 | 8.6E-1 | 1.0E0 | |  | GOTERM\_BP\_5 | positive regulation of antimicrobial humoral response | **RT** |  | 3 | 8.6E-1 | 1.0E0 | |  | GOTERM\_BP\_5 | positive regulation of production of molecular mediator of immune response | **RT** |  | 3 | 8.6E-1 | 1.0E0 | |  | GOTERM\_BP\_5 | positive regulation of defense response | **RT** |  | 9 | 8.7E-1 | 1.0E0 | |  | GOTERM\_BP\_5 | positive regulation of cellular amide metabolic process | **RT** |  | 7 | 8.7E-1 | 1.0E0 | |  | GOTERM\_BP\_5 | regulation of antimicrobial peptide biosynthetic process | **RT** |  | 4 | 8.8E-1 | 1.0E0 | |  | GOTERM\_BP\_5 | antimicrobial peptide biosynthetic process | **RT** |  | 3 | 8.9E-1 | 1.0E0 | |  | GOTERM\_BP\_5 | antibacterial humoral response | **RT** |  | 3 | 8.9E-1 | 1.0E0 | |  | GOTERM\_BP\_5 | antimicrobial peptide production | **RT** |  | 4 | 9.4E-1 | 1.0E0 | | Annotation Cluster 131 | | Enrichment Score: 0.09 |  |  | Count | P\_Value | Benjamini | | --- | --- | --- | --- | --- | --- | --- | --- | |  | GOTERM\_CC\_5 | MLL3/4 complex | **RT** |  | 3 | 5.1E-1 | 9.9E-1 | |  | GOTERM\_CC\_5 | histone methyltransferase complex | **RT** |  | 6 | 6.5E-1 | 1.0E0 | |  | GOTERM\_CC\_5 | methyltransferase complex | **RT** |  | 6 | 7.3E-1 | 1.0E0 | |  | GOTERM\_BP\_5 | peptidyl-lysine methylation | **RT** |  | 6 | 9.4E-1 | 1.0E0 | |  | GOTERM\_BP\_5 | histone methylation | **RT** |  | 6 | 9.7E-1 | 1.0E0 | |  | GOTERM\_BP\_5 | protein methylation | **RT** |  | 6 | 9.9E-1 | 1.0E0 | |  | GOTERM\_BP\_5 | macromolecule methylation | **RT** |  | 7 | 1.0E0 | 1.0E0 | | Annotation Cluster 132 | | Enrichment Score: 0.09 |  |  | Count | P\_Value | Benjamini | | --- | --- | --- | --- | --- | --- | --- | --- | |  | GOTERM\_BP\_5 | positive regulation of STAT cascade | **RT** |  | 4 | 6.9E-1 | 1.0E0 | |  | GOTERM\_BP\_5 | negative regulation of STAT cascade | **RT** |  | 3 | 8.2E-1 | 1.0E0 | |  | GOTERM\_BP\_5 | regulation of STAT cascade | **RT** |  | 8 | 8.4E-1 | 1.0E0 | |  | GOTERM\_BP\_5 | STAT cascade | **RT** |  | 8 | 8.8E-1 | 1.0E0 | | Annotation Cluster 133 | | Enrichment Score: 0.09 |  |  | Count | P\_Value | Benjamini | | --- | --- | --- | --- | --- | --- | --- | --- | |  | GOTERM\_CC\_5 | ubiquitin ligase complex | **RT** |  | 21 | 6.6E-1 | 1.0E0 | |  | GOTERM\_CC\_5 | cullin-RING ubiquitin ligase complex | **RT** |  | 12 | 7.9E-1 | 1.0E0 | |  | GOTERM\_CC\_5 | SCF ubiquitin ligase complex | **RT** |  | 7 | 8.2E-1 | 1.0E0 | |  | GOTERM\_BP\_5 | proteasomal protein catabolic process | **RT** |  | 16 | 1.0E0 | 1.0E0 | | Annotation Cluster 134 | | Enrichment Score: 0.09 |  |  | Count | P\_Value | Benjamini | | --- | --- | --- | --- | --- | --- | --- | --- | |  | GOTERM\_CC\_5 | lytic vacuole membrane | **RT** |  | 5 | 4.2E-1 | 9.8E-1 | |  | GOTERM\_CC\_5 | lysosomal membrane | **RT** |  | 5 | 4.2E-1 | 9.8E-1 | |  | GOTERM\_CC\_5 | lytic vacuole | **RT** |  | 8 | 9.2E-1 | 1.0E0 | |  | GOTERM\_CC\_5 | late endosome membrane | **RT** |  | 3 | 9.5E-1 | 1.0E0 | |  | GOTERM\_CC\_5 | late endosome | **RT** |  | 7 | 9.8E-1 | 1.0E0 | |  | GOTERM\_CC\_5 | endosome membrane | **RT** |  | 4 | 9.9E-1 | 1.0E0 | |  | GOTERM\_CC\_5 | endosomal part | **RT** |  | 5 | 9.9E-1 | 1.0E0 | |  | GOTERM\_CC\_5 | vacuolar membrane | **RT** |  | 6 | 1.0E0 | 1.0E0 | |  | GOTERM\_CC\_5 | vacuolar part | **RT** |  | 6 | 1.0E0 | 1.0E0 | | Annotation Cluster 135 | | Enrichment Score: 0.09 |  |  | Count | P\_Value | Benjamini | | --- | --- | --- | --- | --- | --- | --- | --- | |  | GOTERM\_BP\_5 | negative regulation of neuron death | **RT** |  | 7 | 4.7E-1 | 9.9E-1 | |  | GOTERM\_BP\_5 | regulation of programmed cell death | **RT** |  | 31 | 7.6E-1 | 1.0E0 | |  | GOTERM\_BP\_5 | negative regulation of programmed cell death | **RT** |  | 14 | 8.7E-1 | 1.0E0 | |  | GOTERM\_BP\_5 | regulation of apoptotic process | **RT** |  | 25 | 9.0E-1 | 1.0E0 | |  | GOTERM\_BP\_5 | negative regulation of cell death | **RT** |  | 16 | 9.1E-1 | 1.0E0 | |  | GOTERM\_BP\_5 | negative regulation of apoptotic process | **RT** |  | 12 | 9.2E-1 | 1.0E0 | |  | GOTERM\_BP\_5 | apoptotic process | **RT** |  | 26 | 9.9E-1 | 1.0E0 | | Annotation Cluster 136 | | Enrichment Score: 0.09 |  |  | Count | P\_Value | Benjamini | | --- | --- | --- | --- | --- | --- | --- | --- | |  | GOTERM\_BP\_5 | establishment of localization by movement along microtubule | **RT** |  | 10 | 6.8E-1 | 1.0E0 | |  | GOTERM\_CC\_5 | cell projection cytoplasm | **RT** |  | 4 | 7.2E-1 | 1.0E0 | |  | GOTERM\_BP\_5 | cytoskeleton-dependent intracellular transport | **RT** |  | 9 | 7.5E-1 | 1.0E0 | |  | GOTERM\_BP\_5 | axo-dendritic transport | **RT** |  | 7 | 7.7E-1 | 1.0E0 | |  | GOTERM\_CC\_5 | axon cytoplasm | **RT** |  | 3 | 8.7E-1 | 1.0E0 | |  | GOTERM\_BP\_5 | axonal transport | **RT** |  | 3 | 9.3E-1 | 1.0E0 | |  | GOTERM\_BP\_5 | single-organism intracellular transport | **RT** |  | 21 | 1.0E0 | 1.0E0 | | Annotation Cluster 137 | | Enrichment Score: 0.09 |  |  | Count | P\_Value | Benjamini | | --- | --- | --- | --- | --- | --- | --- | --- | |  | GOTERM\_BP\_5 | female meiotic division | **RT** |  | 8 | 6.6E-1 | 1.0E0 | |  | GOTERM\_BP\_5 | female meiosis chromosome segregation | **RT** |  | 4 | 8.6E-1 | 1.0E0 | |  | GOTERM\_BP\_5 | meiotic chromosome segregation | **RT** |  | 5 | 9.6E-1 | 1.0E0 | | Annotation Cluster 138 | | Enrichment Score: 0.09 |  |  | Count | P\_Value | Benjamini | | --- | --- | --- | --- | --- | --- | --- | --- | |  | GOTERM\_BP\_5 | activation of innate immune response | **RT** |  | 4 | 6.9E-1 | 1.0E0 | |  | GOTERM\_BP\_5 | activation of immune response | **RT** |  | 4 | 7.4E-1 | 1.0E0 | |  | GOTERM\_BP\_5 | regulation of innate immune response | **RT** |  | 12 | 8.6E-1 | 1.0E0 | |  | GOTERM\_BP\_5 | positive regulation of defense response | **RT** |  | 9 | 8.7E-1 | 1.0E0 | |  | GOTERM\_BP\_5 | positive regulation of innate immune response | **RT** |  | 6 | 9.4E-1 | 1.0E0 | | Annotation Cluster 139 | | Enrichment Score: 0.09 |  |  | Count | P\_Value | Benjamini | | --- | --- | --- | --- | --- | --- | --- | --- | |  | GOTERM\_BP\_5 | hematopoietic or lymphoid organ development | **RT** |  | 15 | 6.7E-1 | 1.0E0 | |  | GOTERM\_BP\_5 | immune system development | **RT** |  | 15 | 6.7E-1 | 1.0E0 | |  | GOTERM\_BP\_5 | hemopoiesis | **RT** |  | 12 | 8.3E-1 | 1.0E0 | |  | GOTERM\_BP\_5 | lymph gland development | **RT** |  | 7 | 8.5E-1 | 1.0E0 | |  | GOTERM\_BP\_5 | post-embryonic hemopoiesis | **RT** |  | 4 | 9.7E-1 | 1.0E0 | |  | GOTERM\_BP\_5 | larval lymph gland hemopoiesis | **RT** |  | 4 | 9.7E-1 | 1.0E0 | | Annotation Cluster 140 | | Enrichment Score: 0.09 |  |  | Count | P\_Value | Benjamini | | --- | --- | --- | --- | --- | --- | --- | --- | |  | GOTERM\_CC\_5 | cytoskeleton | **RT** |  | 92 | 4.4E-1 | 9.8E-1 | |  | GOTERM\_CC\_5 | cytoskeletal part | **RT** |  | 69 | 9.3E-1 | 1.0E0 | |  | GOTERM\_CC\_5 | microtubule cytoskeleton | **RT** |  | 60 | 9.4E-1 | 1.0E0 | |  | GOTERM\_CC\_5 | microtubule associated complex | **RT** |  | 41 | 9.6E-1 | 1.0E0 | |  | GOTERM\_CC\_5 | intracellular non-membrane-bounded organelle | **RT** |  | 162 | 1.0E0 | 1.0E0 | | Annotation Cluster 141 | | Enrichment Score: 0.08 |  |  | Count | P\_Value | Benjamini | | --- | --- | --- | --- | --- | --- | --- | --- | |  | GOTERM\_BP\_5 | positive regulation of nucleobase-containing compound metabolic process | **RT** |  | 58 | 6.1E-1 | 1.0E0 | |  | GOTERM\_BP\_5 | positive regulation of cellular biosynthetic process | **RT** |  | 63 | 7.4E-1 | 1.0E0 | |  | GOTERM\_BP\_5 | positive regulation of RNA metabolic process | **RT** |  | 49 | 8.5E-1 | 1.0E0 | |  | GOTERM\_BP\_5 | positive regulation of macromolecule metabolic process | **RT** |  | 92 | 8.6E-1 | 1.0E0 | |  | GOTERM\_BP\_5 | positive regulation of gene expression | **RT** |  | 56 | 8.8E-1 | 1.0E0 | |  | GOTERM\_BP\_5 | positive regulation of RNA biosynthetic process | **RT** |  | 47 | 9.0E-1 | 1.0E0 | |  | GOTERM\_BP\_5 | positive regulation of transcription, DNA-templated | **RT** |  | 47 | 9.0E-1 | 1.0E0 | |  | GOTERM\_BP\_5 | positive regulation of macromolecule biosynthetic process | **RT** |  | 51 | 9.4E-1 | 1.0E0 | | Annotation Cluster 142 | | Enrichment Score: 0.07 |  |  | Count | P\_Value | Benjamini | | --- | --- | --- | --- | --- | --- | --- | --- | |  | GOTERM\_BP\_5 | regulation of neuroblast proliferation | **RT** |  | 6 | 7.1E-1 | 1.0E0 | |  | GOTERM\_BP\_5 | negative regulation of neuroblast proliferation | **RT** |  | 4 | 8.8E-1 | 1.0E0 | |  | GOTERM\_BP\_5 | negative regulation of neural precursor cell proliferation | **RT** |  | 4 | 8.8E-1 | 1.0E0 | |  | GOTERM\_BP\_5 | negative regulation of stem cell proliferation | **RT** |  | 4 | 9.0E-1 | 1.0E0 | | Annotation Cluster 143 | | Enrichment Score: 0.07 |  |  | Count | P\_Value | Benjamini | | --- | --- | --- | --- | --- | --- | --- | --- | |  | GOTERM\_BP\_5 | gene silencing | **RT** |  | 27 | 8.2E-1 | 1.0E0 | |  | GOTERM\_BP\_5 | regulation of gene expression, epigenetic | **RT** |  | 24 | 8.6E-1 | 1.0E0 | |  | GOTERM\_BP\_5 | negative regulation of gene expression, epigenetic | **RT** |  | 13 | 8.6E-1 | 1.0E0 | |  | GOTERM\_BP\_5 | chromatin silencing | **RT** |  | 13 | 8.6E-1 | 1.0E0 | | Annotation Cluster 144 | | Enrichment Score: 0.07 |  |  | Count | P\_Value | Benjamini | | --- | --- | --- | --- | --- | --- | --- | --- | |  | GOTERM\_BP\_5 | regulation of gene silencing | **RT** |  | 13 | 6.7E-1 | 1.0E0 | |  | GOTERM\_BP\_5 | regulation of chromatin silencing | **RT** |  | 6 | 9.2E-1 | 1.0E0 | |  | GOTERM\_BP\_5 | regulation of chromosome organization | **RT** |  | 10 | 1.0E0 | 1.0E0 | | Annotation Cluster 145 | | Enrichment Score: 0.07 |  |  | Count | P\_Value | Benjamini | | --- | --- | --- | --- | --- | --- | --- | --- | |  | GOTERM\_MF\_5 | guanyl ribonucleotide binding | **RT** |  | 26 | 8.4E-1 | 1.0E0 | |  | GOTERM\_MF\_5 | guanyl nucleotide binding | **RT** |  | 26 | 8.4E-1 | 1.0E0 | |  | GOTERM\_MF\_5 | GTP binding | **RT** |  | 24 | 9.0E-1 | 1.0E0 | | Annotation Cluster 146 | | Enrichment Score: 0.06 |  |  | Count | P\_Value | Benjamini | | --- | --- | --- | --- | --- | --- | --- | --- | |  | GOTERM\_MF\_5 | protein kinase activity | **RT** |  | 47 | 2.9E-1 | 9.3E-1 | |  | GOTERM\_MF\_5 | adenyl ribonucleotide binding | **RT** |  | 86 | 9.8E-1 | 1.0E0 | |  | GOTERM\_MF\_5 | adenyl nucleotide binding | **RT** |  | 86 | 9.8E-1 | 1.0E0 | |  | GOTERM\_MF\_5 | ATP binding | **RT** |  | 85 | 9.8E-1 | 1.0E0 | |  | GOTERM\_MF\_5 | purine ribonucleotide binding | **RT** |  | 110 | 9.9E-1 | 1.0E0 | |  | GOTERM\_MF\_5 | ribonucleotide binding | **RT** |  | 111 | 9.9E-1 | 1.0E0 | |  | GOTERM\_MF\_5 | purine nucleotide binding | **RT** |  | 110 | 9.9E-1 | 1.0E0 | |  | GOTERM\_BP\_5 | phosphorylation | **RT** |  | 81 | 9.9E-1 | 1.0E0 | |  | GOTERM\_MF\_5 | purine ribonucleoside binding | **RT** |  | 107 | 9.9E-1 | 1.0E0 | | Annotation Cluster 147 | | Enrichment Score: 0.06 |  |  | Count | P\_Value | Benjamini | | --- | --- | --- | --- | --- | --- | --- | --- | |  | GOTERM\_BP\_5 | G1/S transition of mitotic cell cycle | **RT** |  | 9 | 1.5E-1 | 8.3E-1 | |  | GOTERM\_BP\_5 | negative regulation of cell cycle G1/S phase transition | **RT** |  | 4 | 5.0E-1 | 9.9E-1 | |  | GOTERM\_BP\_5 | regulation of cell cycle G1/S phase transition | **RT** |  | 5 | 5.1E-1 | 9.9E-1 | |  | GOTERM\_BP\_5 | DNA damage checkpoint | **RT** |  | 10 | 9.5E-1 | 1.0E0 | |  | GOTERM\_BP\_5 | negative regulation of cell cycle process | **RT** |  | 14 | 9.6E-1 | 1.0E0 | |  | GOTERM\_BP\_5 | regulation of mitotic cell cycle | **RT** |  | 22 | 9.6E-1 | 1.0E0 | |  | GOTERM\_BP\_5 | mitotic DNA damage checkpoint | **RT** |  | 9 | 9.7E-1 | 1.0E0 | |  | GOTERM\_BP\_5 | mitotic DNA integrity checkpoint | **RT** |  | 9 | 9.7E-1 | 1.0E0 | |  | GOTERM\_BP\_5 | negative regulation of cell cycle | **RT** |  | 17 | 9.7E-1 | 1.0E0 | |  | GOTERM\_BP\_5 | mitotic G2/M transition checkpoint | **RT** |  | 8 | 9.8E-1 | 1.0E0 | |  | GOTERM\_BP\_5 | cell cycle phase transition | **RT** |  | 18 | 9.8E-1 | 1.0E0 | |  | GOTERM\_BP\_5 | regulation of cell cycle process | **RT** |  | 20 | 9.8E-1 | 1.0E0 | |  | GOTERM\_BP\_5 | negative regulation of cell cycle G2/M phase transition | **RT** |  | 8 | 9.8E-1 | 1.0E0 | |  | GOTERM\_BP\_5 | mitotic cell cycle phase transition | **RT** |  | 17 | 9.9E-1 | 1.0E0 | |  | GOTERM\_BP\_5 | cell cycle checkpoint | **RT** |  | 11 | 9.9E-1 | 1.0E0 | |  | GOTERM\_BP\_5 | negative regulation of mitotic cell cycle phase transition | **RT** |  | 11 | 9.9E-1 | 1.0E0 | |  | GOTERM\_BP\_5 | negative regulation of cell cycle phase transition | **RT** |  | 11 | 9.9E-1 | 1.0E0 | |  | GOTERM\_BP\_5 | mitotic G2 DNA damage checkpoint | **RT** |  | 7 | 9.9E-1 | 1.0E0 | |  | GOTERM\_BP\_5 | G2/M transition of mitotic cell cycle | **RT** |  | 8 | 9.9E-1 | 1.0E0 | |  | GOTERM\_BP\_5 | regulation of cell cycle G2/M phase transition | **RT** |  | 8 | 9.9E-1 | 1.0E0 | |  | GOTERM\_BP\_5 | regulation of mitotic cell cycle phase transition | **RT** |  | 14 | 9.9E-1 | 1.0E0 | |  | GOTERM\_BP\_5 | regulation of cell cycle phase transition | **RT** |  | 14 | 9.9E-1 | 1.0E0 | |  | GOTERM\_BP\_5 | negative regulation of mitotic cell cycle | **RT** |  | 11 | 9.9E-1 | 1.0E0 | |  | GOTERM\_BP\_5 | mitotic cell cycle checkpoint | **RT** |  | 9 | 1.0E0 | 1.0E0 | |  | GOTERM\_BP\_5 | mitotic cell cycle process | **RT** |  | 44 | 1.0E0 | 1.0E0 | | Annotation Cluster 148 | | Enrichment Score: 0.06 |  |  | Count | P\_Value | Benjamini | | --- | --- | --- | --- | --- | --- | --- | --- | |  | GOTERM\_BP\_5 | regulation of mRNA metabolic process | **RT** |  | 15 | 8.1E-1 | 1.0E0 | |  | GOTERM\_BP\_5 | regulation of RNA splicing | **RT** |  | 13 | 9.0E-1 | 1.0E0 | |  | GOTERM\_BP\_5 | regulation of mRNA processing | **RT** |  | 13 | 9.0E-1 | 1.0E0 | | Annotation Cluster 149 | | Enrichment Score: 0.06 |  |  | Count | P\_Value | Benjamini | | --- | --- | --- | --- | --- | --- | --- | --- | |  | GOTERM\_BP\_5 | positive regulation of cellular catabolic process | **RT** |  | 6 | 7.5E-1 | 1.0E0 | |  | GOTERM\_BP\_5 | positive regulation of protein catabolic process | **RT** |  | 6 | 8.4E-1 | 1.0E0 | |  | GOTERM\_BP\_5 | regulation of protein catabolic process | **RT** |  | 8 | 8.6E-1 | 1.0E0 | |  | GOTERM\_BP\_5 | positive regulation of cellular protein catabolic process | **RT** |  | 3 | 9.6E-1 | 1.0E0 | |  | GOTERM\_BP\_5 | regulation of cellular protein catabolic process | **RT** |  | 3 | 9.9E-1 | 1.0E0 | | Annotation Cluster 150 | | Enrichment Score: 0.06 |  |  | Count | P\_Value | Benjamini | | --- | --- | --- | --- | --- | --- | --- | --- | |  | GOTERM\_MF\_5 | flavin adenine dinucleotide binding | **RT** |  | 5 | 8.8E-1 | 1.0E0 | |  | GOTERM\_CC\_5 | microbody | **RT** |  | 6 | 8.8E-1 | 1.0E0 | |  | GOTERM\_CC\_5 | peroxisome | **RT** |  | 6 | 8.8E-1 | 1.0E0 | | Annotation Cluster 151 | | Enrichment Score: 0.05 |  |  | Count | P\_Value | Benjamini | | --- | --- | --- | --- | --- | --- | --- | --- | |  | GOTERM\_BP\_5 | spermatid differentiation | **RT** |  | 15 | 7.9E-1 | 1.0E0 | |  | GOTERM\_BP\_5 | spermatid development | **RT** |  | 13 | 8.4E-1 | 1.0E0 | |  | GOTERM\_BP\_5 | male gamete generation | **RT** |  | 25 | 9.2E-1 | 1.0E0 | |  | GOTERM\_BP\_5 | spermatogenesis | **RT** |  | 25 | 9.2E-1 | 1.0E0 | |  | GOTERM\_BP\_5 | sperm individualization | **RT** |  | 5 | 9.6E-1 | 1.0E0 | | Annotation Cluster 152 | | Enrichment Score: 0.04 |  |  | Count | P\_Value | Benjamini | | --- | --- | --- | --- | --- | --- | --- | --- | |  | GOTERM\_BP\_5 | regulation of cell projection assembly | **RT** |  | 6 | 8.9E-1 | 1.0E0 | |  | GOTERM\_BP\_5 | positive regulation of filopodium assembly | **RT** |  | 3 | 9.1E-1 | 1.0E0 | |  | GOTERM\_BP\_5 | regulation of filopodium assembly | **RT** |  | 4 | 9.2E-1 | 1.0E0 | |  | GOTERM\_BP\_5 | filopodium assembly | **RT** |  | 4 | 9.4E-1 | 1.0E0 | | Annotation Cluster 153 | | Enrichment Score: 0.04 |  |  | Count | P\_Value | Benjamini | | --- | --- | --- | --- | --- | --- | --- | --- | |  | GOTERM\_BP\_5 | centriole assembly | **RT** |  | 4 | 7.9E-1 | 1.0E0 | |  | GOTERM\_BP\_5 | centriole replication | **RT** |  | 4 | 7.9E-1 | 1.0E0 | |  | GOTERM\_BP\_5 | centrosome organization | **RT** |  | 14 | 9.9E-1 | 1.0E0 | |  | GOTERM\_BP\_5 | microtubule organizing center organization | **RT** |  | 14 | 9.9E-1 | 1.0E0 | |  | GOTERM\_BP\_5 | centrosome duplication | **RT** |  | 9 | 9.9E-1 | 1.0E0 | |  | GOTERM\_BP\_5 | centrosome cycle | **RT** |  | 11 | 1.0E0 | 1.0E0 | | Annotation Cluster 154 | | Enrichment Score: 0.03 |  |  | Count | P\_Value | Benjamini | | --- | --- | --- | --- | --- | --- | --- | --- | |  | GOTERM\_BP\_5 | eggshell chorion gene amplification | **RT** |  | 3 | 7.2E-1 | 1.0E0 | |  | GOTERM\_BP\_5 | DNA biosynthetic process | **RT** |  | 4 | 9.2E-1 | 1.0E0 | |  | GOTERM\_BP\_5 | chorion-containing eggshell formation | **RT** |  | 8 | 9.7E-1 | 1.0E0 | |  | GOTERM\_BP\_5 | eggshell formation | **RT** |  | 8 | 9.8E-1 | 1.0E0 | |  | GOTERM\_BP\_5 | eggshell chorion assembly | **RT** |  | 6 | 9.8E-1 | 1.0E0 | |  | GOTERM\_BP\_5 | dorsal appendage formation | **RT** |  | 3 | 1.0E0 | 1.0E0 | | Annotation Cluster 155 | | Enrichment Score: 0.03 |  |  | Count | P\_Value | Benjamini | | --- | --- | --- | --- | --- | --- | --- | --- | |  | GOTERM\_BP\_5 | alpha-amino acid metabolic process | **RT** |  | 8 | 8.6E-1 | 1.0E0 | |  | GOTERM\_BP\_5 | alpha-amino acid catabolic process | **RT** |  | 3 | 9.3E-1 | 1.0E0 | |  | GOTERM\_BP\_5 | carboxylic acid catabolic process | **RT** |  | 4 | 9.6E-1 | 1.0E0 | |  | GOTERM\_BP\_5 | organic acid catabolic process | **RT** |  | 4 | 9.8E-1 | 1.0E0 | | Annotation Cluster 156 | | Enrichment Score: 0.03 |  |  | Count | P\_Value | Benjamini | | --- | --- | --- | --- | --- | --- | --- | --- | |  | GOTERM\_BP\_5 | response to starvation | **RT** |  | 20 | 8.3E-1 | 1.0E0 | |  | GOTERM\_BP\_5 | cellular response to starvation | **RT** |  | 7 | 1.0E0 | 1.0E0 | |  | GOTERM\_BP\_5 | cellular response to nutrient levels | **RT** |  | 7 | 1.0E0 | 1.0E0 | | Annotation Cluster 157 | | Enrichment Score: 0.03 |  |  | Count | P\_Value | Benjamini | | --- | --- | --- | --- | --- | --- | --- | --- | |  | GOTERM\_BP\_5 | male meiosis cytokinesis | **RT** |  | 4 | 8.3E-1 | 1.0E0 | |  | GOTERM\_BP\_5 | meiotic cytokinesis | **RT** |  | 4 | 8.8E-1 | 1.0E0 | |  | GOTERM\_BP\_5 | male meiosis | **RT** |  | 7 | 9.2E-1 | 1.0E0 | |  | GOTERM\_BP\_5 | cytokinetic process | **RT** |  | 3 | 9.7E-1 | 1.0E0 | |  | GOTERM\_BP\_5 | cytoskeleton-dependent cytokinesis | **RT** |  | 8 | 9.9E-1 | 1.0E0 | |  | GOTERM\_BP\_5 | cytokinesis | **RT** |  | 8 | 9.9E-1 | 1.0E0 | |  | GOTERM\_BP\_5 | mitotic cytokinesis | **RT** |  | 4 | 1.0E0 | 1.0E0 | | Annotation Cluster 158 | | Enrichment Score: 0.03 |  |  | Count | P\_Value | Benjamini | | --- | --- | --- | --- | --- | --- | --- | --- | |  | GOTERM\_BP\_5 | tricarboxylic acid cycle | **RT** |  | 5 | 8.8E-1 | 1.0E0 | |  | GOTERM\_BP\_5 | aerobic respiration | **RT** |  | 5 | 9.5E-1 | 1.0E0 | |  | GOTERM\_BP\_5 | cellular respiration | **RT** |  | 5 | 1.0E0 | 1.0E0 | | Annotation Cluster 159 | | Enrichment Score: 0.02 |  |  | Count | P\_Value | Benjamini | | --- | --- | --- | --- | --- | --- | --- | --- | |  | GOTERM\_BP\_5 | glycerophospholipid biosynthetic process | **RT** |  | 4 | 8.8E-1 | 1.0E0 | |  | GOTERM\_BP\_5 | phospholipid biosynthetic process | **RT** |  | 6 | 8.9E-1 | 1.0E0 | |  | GOTERM\_BP\_5 | glycerolipid biosynthetic process | **RT** |  | 4 | 9.6E-1 | 1.0E0 | |  | GOTERM\_BP\_5 | phospholipid metabolic process | **RT** |  | 12 | 9.6E-1 | 1.0E0 | |  | GOTERM\_BP\_5 | glycerophospholipid metabolic process | **RT** |  | 6 | 1.0E0 | 1.0E0 | |  | GOTERM\_BP\_5 | glycerolipid metabolic process | **RT** |  | 7 | 1.0E0 | 1.0E0 | | Annotation Cluster 160 | | Enrichment Score: 0.02 |  |  | Count | P\_Value | Benjamini | | --- | --- | --- | --- | --- | --- | --- | --- | |  | GOTERM\_CC\_5 | transcription factor complex | **RT** |  | 14 | 9.0E-1 | 1.0E0 | |  | GOTERM\_CC\_5 | nuclear transcription factor complex | **RT** |  | 8 | 9.7E-1 | 1.0E0 | |  | GOTERM\_CC\_5 | RNA polymerase II transcription factor complex | **RT** |  | 7 | 9.8E-1 | 1.0E0 | | Annotation Cluster 161 | | Enrichment Score: 0.02 |  |  | Count | P\_Value | Benjamini | | --- | --- | --- | --- | --- | --- | --- | --- | |  | GOTERM\_BP\_5 | alpha-amino acid metabolic process | **RT** |  | 8 | 8.6E-1 | 1.0E0 | |  | GOTERM\_BP\_5 | carboxylic acid metabolic process | **RT** |  | 20 | 1.0E0 | 1.0E0 | |  | GOTERM\_BP\_5 | oxoacid metabolic process | **RT** |  | 20 | 1.0E0 | 1.0E0 | | Annotation Cluster 162 | | Enrichment Score: 0.02 |  |  | Count | P\_Value | Benjamini | | --- | --- | --- | --- | --- | --- | --- | --- | |  | GOTERM\_BP\_5 | negative regulation of transferase activity | **RT** |  | 7 | 8.5E-1 | 1.0E0 | |  | GOTERM\_BP\_5 | regulation of kinase activity | **RT** |  | 13 | 9.6E-1 | 1.0E0 | |  | GOTERM\_BP\_5 | negative regulation of MAPK cascade | **RT** |  | 3 | 9.9E-1 | 1.0E0 | |  | GOTERM\_BP\_5 | negative regulation of phosphate metabolic process | **RT** |  | 9 | 9.9E-1 | 1.0E0 | |  | GOTERM\_BP\_5 | negative regulation of phosphorus metabolic process | **RT** |  | 9 | 9.9E-1 | 1.0E0 | |  | GOTERM\_BP\_5 | negative regulation of protein modification process | **RT** |  | 10 | 9.9E-1 | 1.0E0 | | Annotation Cluster 163 | | Enrichment Score: 0.01 |  |  | Count | P\_Value | Benjamini | | --- | --- | --- | --- | --- | --- | --- | --- | |  | GOTERM\_BP\_5 | sister chromatid segregation | **RT** |  | 12 | 9.3E-1 | 1.0E0 | |  | GOTERM\_BP\_5 | nuclear chromosome segregation | **RT** |  | 16 | 9.4E-1 | 1.0E0 | |  | GOTERM\_BP\_5 | mitotic sister chromatid segregation | **RT** |  | 9 | 9.8E-1 | 1.0E0 | |  | GOTERM\_BP\_5 | mitotic nuclear division | **RT** |  | 22 | 1.0E0 | 1.0E0 | |  | GOTERM\_BP\_5 | nuclear division | **RT** |  | 34 | 1.0E0 | 1.0E0 | | Annotation Cluster 164 | | Enrichment Score: 0.01 |  |  | Count | P\_Value | Benjamini | | --- | --- | --- | --- | --- | --- | --- | --- | |  | GOTERM\_BP\_5 | fatty acid biosynthetic process | **RT** |  | 3 | 9.5E-1 | 1.0E0 | |  | GOTERM\_BP\_5 | fatty acid metabolic process | **RT** |  | 5 | 9.7E-1 | 1.0E0 | |  | GOTERM\_BP\_5 | carboxylic acid biosynthetic process | **RT** |  | 5 | 9.8E-1 | 1.0E0 | |  | GOTERM\_BP\_5 | organic acid biosynthetic process | **RT** |  | 5 | 9.9E-1 | 1.0E0 | | Annotation Cluster 165 | | Enrichment Score: 0 |  |  | Count | P\_Value | Benjamini | | --- | --- | --- | --- | --- | --- | --- | --- | |  | GOTERM\_CC\_5 | endoplasmic reticulum part | **RT** |  | 24 | 9.9E-1 | 1.0E0 | |  | GOTERM\_CC\_5 | endoplasmic reticulum membrane | **RT** |  | 21 | 9.9E-1 | 1.0E0 | |  | GOTERM\_CC\_5 | endoplasmic reticulum | **RT** |  | 37 | 1.0E0 | 1.0E0 | | Annotation Cluster 166 | | Enrichment Score: 0 |  |  | Count | P\_Value | Benjamini | | --- | --- | --- | --- | --- | --- | --- | --- | |  | GOTERM\_BP\_5 | dsRNA transport | **RT** |  | 3 | 9.7E-1 | 1.0E0 | |  | GOTERM\_BP\_5 | mRNA transport | **RT** |  | 3 | 1.0E0 | 1.0E0 | |  | GOTERM\_BP\_5 | RNA transport | **RT** |  | 7 | 1.0E0 | 1.0E0 | |  | GOTERM\_BP\_5 | nucleobase-containing compound transport | **RT** |  | 8 | 1.0E0 | 1.0E0 | | Annotation Cluster 167 | | Enrichment Score: 0 |  |  | Count | P\_Value | Benjamini | | --- | --- | --- | --- | --- | --- | --- | --- | |  | GOTERM\_CC\_5 | histone acetyltransferase complex | **RT** |  | 5 | 9.9E-1 | 1.0E0 | |  | GOTERM\_CC\_5 | protein acetyltransferase complex | **RT** |  | 5 | 1.0E0 | 1.0E0 | |  | GOTERM\_CC\_5 | acetyltransferase complex | **RT** |  | 5 | 1.0E0 | 1.0E0 | | Annotation Cluster 168 | | Enrichment Score: 0 |  |  | Count | P\_Value | Benjamini | | --- | --- | --- | --- | --- | --- | --- | --- | |  | GOTERM\_CC\_5 | chromatin | **RT** |  | 20 | 9.9E-1 | 1.0E0 | |  | GOTERM\_CC\_5 | polytene chromosome | **RT** |  | 23 | 9.9E-1 | 1.0E0 | |  | GOTERM\_CC\_5 | chromosomal region | **RT** |  | 17 | 9.9E-1 | 1.0E0 | |  | GOTERM\_CC\_5 | chromosomal part | **RT** |  | 33 | 1.0E0 | 1.0E0 | |  | GOTERM\_CC\_5 | chromosome | **RT** |  | 40 | 1.0E0 | 1.0E0 | | Annotation Cluster 169 | | Enrichment Score: 0 |  |  | Count | P\_Value | Benjamini | | --- | --- | --- | --- | --- | --- | --- | --- | |  | GOTERM\_MF\_5 | poly(A) RNA binding | **RT** |  | 35 | 9.8E-1 | 1.0E0 | |  | GOTERM\_BP\_5 | mRNA metabolic process | **RT** |  | 42 | 9.9E-1 | 1.0E0 | |  | GOTERM\_CC\_5 | catalytic step 2 spliceosome | **RT** |  | 12 | 9.9E-1 | 1.0E0 | |  | GOTERM\_CC\_5 | precatalytic spliceosome | **RT** |  | 14 | 1.0E0 | 1.0E0 | |  | GOTERM\_CC\_5 | spliceosomal complex | **RT** |  | 15 | 1.0E0 | 1.0E0 | |  | GOTERM\_BP\_5 | RNA processing | **RT** |  | 42 | 1.0E0 | 1.0E0 | |  | GOTERM\_CC\_5 | intracellular ribonucleoprotein complex | **RT** |  | 41 | 1.0E0 | 1.0E0 | | Annotation Cluster 170 | | Enrichment Score: 0 |  |  | Count | P\_Value | Benjamini | | --- | --- | --- | --- | --- | --- | --- | --- | |  | GOTERM\_BP\_5 | phosphorylation | **RT** |  | 81 | 9.9E-1 | 1.0E0 | |  | GOTERM\_BP\_5 | protein modification process | **RT** |  | 173 | 9.9E-1 | 1.0E0 | |  | GOTERM\_BP\_5 | cellular protein modification process | **RT** |  | 173 | 9.9E-1 | 1.0E0 | |  | GOTERM\_BP\_5 | cellular protein metabolic process | **RT** |  | 221 | 1.0E0 | 1.0E0 | | Annotation Cluster 171 | | Enrichment Score: 0 |  |  | Count | P\_Value | Benjamini | | --- | --- | --- | --- | --- | --- | --- | --- | |  | GOTERM\_MF\_5 | thiol-dependent ubiquitin-specific protease activity | **RT** |  | 4 | 9.9E-1 | 1.0E0 | |  | GOTERM\_BP\_5 | protein modification by small protein removal | **RT** |  | 7 | 9.9E-1 | 1.0E0 | |  | GOTERM\_MF\_5 | cysteine-type peptidase activity | **RT** |  | 7 | 1.0E0 | 1.0E0 | | Annotation Cluster 172 | | Enrichment Score: 0 |  |  | Count | P\_Value | Benjamini | | --- | --- | --- | --- | --- | --- | --- | --- | |  | GOTERM\_CC\_5 | transcription elongation factor complex | **RT** |  | 4 | 9.7E-1 | 1.0E0 | |  | GOTERM\_CC\_5 | DNA-directed RNA polymerase II, holoenzyme | **RT** |  | 6 | 1.0E0 | 1.0E0 | |  | GOTERM\_CC\_5 | transferase complex, transferring phosphorus-containing groups | **RT** |  | 10 | 1.0E0 | 1.0E0 | |  | GOTERM\_CC\_5 | DNA-directed RNA polymerase complex | **RT** |  | 6 | 1.0E0 | 1.0E0 | |  | GOTERM\_CC\_5 | nuclear DNA-directed RNA polymerase complex | **RT** |  | 6 | 1.0E0 | 1.0E0 | |  | GOTERM\_CC\_5 | RNA polymerase complex | **RT** |  | 6 | 1.0E0 | 1.0E0 | | Annotation Cluster 173 | | Enrichment Score: 0 |  |  | Count | P\_Value | Benjamini | | --- | --- | --- | --- | --- | --- | --- | --- | |  | GOTERM\_CC\_5 | organellar small ribosomal subunit | **RT** |  | 3 | 9.9E-1 | 1.0E0 | |  | GOTERM\_CC\_5 | mitochondrial small ribosomal subunit | **RT** |  | 3 | 9.9E-1 | 1.0E0 | |  | GOTERM\_CC\_5 | organellar large ribosomal subunit | **RT** |  | 3 | 1.0E0 | 1.0E0 | |  | GOTERM\_CC\_5 | mitochondrial large ribosomal subunit | **RT** |  | 3 | 1.0E0 | 1.0E0 | |  | GOTERM\_CC\_5 | organellar ribosome | **RT** |  | 6 | 1.0E0 | 1.0E0 | |  | GOTERM\_CC\_5 | mitochondrial ribosome | **RT** |  | 6 | 1.0E0 | 1.0E0 | |  | GOTERM\_BP\_5 | mitochondrial translation | **RT** |  | 6 | 1.0E0 | 1.0E0 | |  | GOTERM\_CC\_5 | mitochondrial matrix | **RT** |  | 10 | 1.0E0 | 1.0E0 | |  | GOTERM\_CC\_5 | small ribosomal subunit | **RT** |  | 3 | 1.0E0 | 1.0E0 | |  | GOTERM\_CC\_5 | large ribosomal subunit | **RT** |  | 5 | 1.0E0 | 1.0E0 | |  | GOTERM\_CC\_5 | ribosomal subunit | **RT** |  | 8 | 1.0E0 | 1.0E0 | |  | GOTERM\_CC\_5 | ribosome | **RT** |  | 8 | 1.0E0 | 1.0E0 | | Annotation Cluster 174 | | Enrichment Score: 0 |  |  | Count | P\_Value | Benjamini | | --- | --- | --- | --- | --- | --- | --- | --- | |  | GOTERM\_BP\_5 | proteolysis | **RT** |  | 74 | 9.9E-1 | 1.0E0 | |  | GOTERM\_BP\_5 | cellular macromolecule catabolic process | **RT** |  | 46 | 1.0E0 | 1.0E0 | |  | GOTERM\_BP\_5 | protein catabolic process | **RT** |  | 35 | 1.0E0 | 1.0E0 | |  | GOTERM\_BP\_5 | proteolysis involved in cellular protein catabolic process | **RT** |  | 33 | 1.0E0 | 1.0E0 | |  | GOTERM\_BP\_5 | cellular protein catabolic process | **RT** |  | 33 | 1.0E0 | 1.0E0 | |  | GOTERM\_BP\_5 | proteasomal protein catabolic process | **RT** |  | 16 | 1.0E0 | 1.0E0 | |  | GOTERM\_BP\_5 | modification-dependent macromolecule catabolic process | **RT** |  | 30 | 1.0E0 | 1.0E0 | | Annotation Cluster 175 | | Enrichment Score: 0 |  |  | Count | P\_Value | Benjamini | | --- | --- | --- | --- | --- | --- | --- | --- | |  | GOTERM\_BP\_5 | cellular protein complex assembly | **RT** |  | 23 | 1.0E0 | 1.0E0 | |  | GOTERM\_BP\_5 | protein complex assembly | **RT** |  | 37 | 1.0E0 | 1.0E0 | |  | GOTERM\_BP\_5 | cellular macromolecular complex assembly | **RT** |  | 33 | 1.0E0 | 1.0E0 | | Annotation Cluster 176 | | Enrichment Score: 0 |  |  | Count | P\_Value | Benjamini | | --- | --- | --- | --- | --- | --- | --- | --- | |  | GOTERM\_BP\_5 | glycosyl compound biosynthetic process | **RT** |  | 4 | 1.0E0 | 1.0E0 | |  | GOTERM\_BP\_5 | nucleoside biosynthetic process | **RT** |  | 4 | 1.0E0 | 1.0E0 | |  | GOTERM\_BP\_5 | nucleoside metabolic process | **RT** |  | 10 | 1.0E0 | 1.0E0 | |  | GOTERM\_BP\_5 | purine nucleoside metabolic process | **RT** |  | 7 | 1.0E0 | 1.0E0 | |  | GOTERM\_BP\_5 | nucleoside monophosphate metabolic process | **RT** |  | 6 | 1.0E0 | 1.0E0 | |  | GOTERM\_BP\_5 | ribonucleoside metabolic process | **RT** |  | 8 | 1.0E0 | 1.0E0 | |  | GOTERM\_BP\_5 | nucleoside triphosphate metabolic process | **RT** |  | 5 | 1.0E0 | 1.0E0 | | Annotation Cluster 177 | | Enrichment Score: 0 |  |  | Count | P\_Value | Benjamini | | --- | --- | --- | --- | --- | --- | --- | --- | |  | GOTERM\_CC\_5 | nucleus | **RT** |  | 261 | 1.0E0 | 1.0E0 | |  | GOTERM\_CC\_5 | intracellular organelle | **RT** |  | 479 | 1.0E0 | 1.0E0 | |  | GOTERM\_CC\_5 | intracellular organelle part | **RT** |  | 234 | 1.0E0 | 1.0E0 | |  | GOTERM\_CC\_5 | intracellular membrane-bounded organelle | **RT** |  | 407 | 1.0E0 | 1.0E0 | | Annotation Cluster 178 | | Enrichment Score: 0 |  |  | Count | P\_Value | Benjamini | | --- | --- | --- | --- | --- | --- | --- | --- | |  | GOTERM\_CC\_5 | nuclear chromatin | **RT** |  | 5 | 1.0E0 | 1.0E0 | |  | GOTERM\_CC\_5 | nuclear chromosome part | **RT** |  | 6 | 1.0E0 | 1.0E0 | |  | GOTERM\_CC\_5 | nuclear chromosome | **RT** |  | 8 | 1.0E0 | 1.0E0 | | Annotation Cluster 179 | | Enrichment Score: 0 |  |  | Count | P\_Value | Benjamini | | --- | --- | --- | --- | --- | --- | --- | --- | |  | GOTERM\_CC\_5 | nucleoplasm | **RT** |  | 41 | 1.0E0 | 1.0E0 | |  | GOTERM\_CC\_5 | nucleoplasm part | **RT** |  | 24 | 1.0E0 | 1.0E0 | |  | GOTERM\_CC\_5 | nuclear lumen | **RT** |  | 51 | 1.0E0 | 1.0E0 | |  | GOTERM\_CC\_5 | nuclear part | **RT** |  | 88 | 1.0E0 | 1.0E0 | |  | GOTERM\_CC\_5 | intracellular organelle lumen | **RT** |  | 61 | 1.0E0 | 1.0E0 | | Annotation Cluster 180 | | Enrichment Score: 0 |  |  | Count | P\_Value | Benjamini | | --- | --- | --- | --- | --- | --- | --- | --- | |  | GOTERM\_CC\_5 | organelle envelope | **RT** |  | 25 | 1.0E0 | 1.0E0 | |  | GOTERM\_CC\_5 | mitochondrial protein complex | **RT** |  | 5 | 1.0E0 | 1.0E0 | |  | GOTERM\_CC\_5 | inner mitochondrial membrane protein complex | **RT** |  | 3 | 1.0E0 | 1.0E0 | |  | GOTERM\_CC\_5 | mitochondrial membrane part | **RT** |  | 4 | 1.0E0 | 1.0E0 | |  | GOTERM\_CC\_5 | mitochondrial envelope | **RT** |  | 11 | 1.0E0 | 1.0E0 | |  | GOTERM\_CC\_5 | organelle inner membrane | **RT** |  | 6 | 1.0E0 | 1.0E0 | |  | GOTERM\_CC\_5 | mitochondrial membrane | **RT** |  | 9 | 1.0E0 | 1.0E0 | |  | GOTERM\_CC\_5 | mitochondrial inner membrane | **RT** |  | 5 | 1.0E0 | 1.0E0 | | Annotation Cluster 181 | | Enrichment Score: 0 |  |  | Count | P\_Value | Benjamini | | --- | --- | --- | --- | --- | --- | --- | --- | |  | GOTERM\_CC\_5 | mitochondrial envelope | **RT** |  | 11 | 1.0E0 | 1.0E0 | |  | GOTERM\_CC\_5 | mitochondrial part | **RT** |  | 21 | 1.0E0 | 1.0E0 | |  | GOTERM\_CC\_5 | mitochondrion | **RT** |  | 32 | 1.0E0 | 1.0E0 |   were not clustered. | |  | |
