# Supplementary material for: Enabling cell-type-specific behavioral epigenetics in Drosophila: a modified high-yield INTACT method reveals the impact of social environment on the epigenetic landscape in dopaminergic neurons
Source: BMC Biol. 2019 Apr 10;17:30. doi: 10.1186/s12915-019-0646-4 (PMC6456965; doi:10.1186/s12915-019-0646-4)
Supplement: Supplementary file 10 — Gorilla and DAVID functional analysis. The zip file contains top level html files which may be opened in a browser. These will give the Gorilla functional analysis and DAVID GO analyses referred to in the main text. (ZIP 919 kb) [file 12915_2019_646_MOESM10_ESM.zip › Additional File 10/TPM82_GOLevel5.html]

DAVID: Database for Annotation, Visualization, and Integrated Discovery (Laboratory of Human Retrovirology and Immunoinformatics (LHRI); National Institute of Allergies and Infectious Diseases (NIAID); Leidos Biomedical Research, Inc. (LBR)


|  |  |  |  |  |  |
| --- | --- | --- | --- | --- | --- |
| DAVID Bioinformatics 6.7  |  |  |  |  |  | | --- | --- | --- | --- | --- | | |  |  |  |  | | --- | --- | --- | --- | |  | |  | | --- | | DAVID Bioinformatics Resources 6.8 | | Laboratory of Human Retrovirology and Immunoinformatics (LHRI) | | |   100%  **\*\*\* Welcome to DAVID 6.8 \*\*\* \*\*\* If you are looking for DAVID 6.7, please visit our development site. \*\*\*** |
| |  |  |  |  |  |  |  |  |  |  |  |  |  |  |  |  |  |  |  |  |  |  |  |  |  |  |  |  |  |  |  |  |  |  |  |  |  |  |  |  |  |  |  |  |  |  |  |  |  |  |  |  |  |  |  |  |  |  |  |  |  |  |  |  |  |  |  |  |  |  |  |  |  |  |  |  |  |  |  |  |  |  |  |  |  |  |  |  |  |  |  |  |  |  |  |  |  |  |  |  |  |  |  |  |  |  |  |  |  |  |  |  |  |  |  |  |  |  |  |  |  |  |  |  |  |  |  |  |  |  |  |  |  |  |  |  |  |  |  |  |  |  |  |  |  |  |  |  |  |  |  |  |  |  |  |  |  |  |  |  |  |  |  |  |  |  |  |  |  |  |  |  |  |  |  |  |  |  |  |  |  |  |  |  |  |  |  |  |  |  |  |  |  |  |  |  |  |  |  |  |  |  |  |  |  |  |  |  |  |  |  |  |  |  |  |  |  |  |  |  |  |  |  |  |  |  |  |  |  |  |  |  |  |  |  |  |  |  |  |  |  |  |  |  |  |  |  |  |  |  |  |  |  |  |  |  |  |  |  |  |  |  |  |  |  |  |  |  |  |  |  |  |  |  |  |  |  |  |  |  |  |  |  |  |  |  |  |  |  |  |  |  |  |  |  |  |  |  |  |  |  |  |  |  |  |  |  |  |  |  |  |  |  |  |  |  |  |  |  |  |  |  |  |  |  |  |  |  |  |  |  |  |  |  |  |  |  |  |  |  |  |  |  |  |  |  |  |  |  |  |  |  |  |  |  |  |  |  |  |  |  |  |  |  |  |  |  |  |  |  |  |  |  |  |  |  |  |  |  |  |  |  |  |  |  |  |  |  |  |  |  |  |  |  |  |  |  |  |  |  |  |  |  |  |  |  |  |  |  |  |  |  |  |  |  |  |  |  |  |  |  |  |  |  |  |  |  |  |  |  |  |  |  |  |  |  |  |  |  |  |  |  |  |  |  |  |  |  |  |  |  |  |  |  |  |  |  |  |  |  |  |  |  |  |  |  |  |  |  |  |  |  |  |  |  |  |  |  |  |  |  |  |  |  |  |  |  |  |  |  |  |  |  |  |  |  |  |  |  |  |  |  |  |  |  |  |  |  |  |  |  |  |  |  |  |  |  |  |  |  |  |  |  |  |  |  |  |  |  |  |  |  |  |  |  |  |  |  |  |  |  |  |  |  |  |  |  |  |  |  |  |  |  |  |  |  |  |  |  |  |  |  |  |  |  |  |  |  |  |  |  |  |  |  |  |  |  |  |  |  |  |  |  |  |  |  |  |  |  |  |  |  |  |  |  |  |  |  |  |  |  |  |  |  |  |  |  |  |  |  |  |  |  |  |  |  |  |  |  |  |  |  |  |  |  |  |  |  |  |  |  |  |  |  |  |  |  |  |  |  |  |  |  |  |  |  |  |  |  |  |  |  |  |  |  |  |  |  |  |  |  |  |  |  |  |  |  |  |  |  |  |  |  |  |  |  |  |  |  |  |  |  |  |  |  |  |  |  |  |  |  |  |  |  |  |  |  |  |  |  |  |  |  |  |  |  |  |  |  |  |  |  |  |  |  |  |  |  |  |  |  |  |  |  |  |  |  |  |  |  |  |  |  |  |  |  |  |  |  |  |  |  |  |  |  |  |  |  |  |  |  |  |  |  |  |  |  |  |  |  |  |  |  |  |  |  |  |  |  |  |  |  |  |  |  |  |  |  |  |  |  |  |  |  |  |  |  |  |  |  |  |  |  |  |  |  |  |  |  |  |  |  |  |  |  |  |  |  |  |  |  |  |  |  |  |  |  |  |  |  |  |  |  |  |  |  |  |  |  |  |  |  |  |  |  |  |  |  |  |  |  |  |  |  |  |  |  |  |  |  |  |  |  |  |  |  |  |  |  |  |  |  |  |  |  |  |  |  |  |  |  |  |  |  |  |  |  |  |  |  |  |  |  |  |  |  |  |  |  |  |  |  |  |  |  |  |  |  |  |  |  |  |  |  |  |  |  |  |  |  |  |  |  |  |  |  |  |  |  |  |  |  |  |  |  |  |  |  |  |  |  |  |  |  |  |  |  |  |  |  |  |  |  |  |  |  |  |  |  |  |  |  |  |  |  |  |  |  |  |  |  |  |  |  |  |  |  |  |  |  |  |  |  |  |  |  |  |  |  |  |  |  |  |  |  |  |  |  |  |  |  |  |  |  |  |  |  |  |  |  |  |  |  |  |  |  |  |  |  |  |  |  |  |  |  |  |  |  |  |  |  |  |  |  |  |  |  |  |  |  |  |  |  |  |  |  |  |  |  |  |  |  |  |  |  |  |  |  |  |  |  |  |  |  |  |  |  |  |  |  |  |  |  |  |  |  |  |  |  |  |  |  |  |  |  |  |  |  |  |  |  |  |  |  |  |  |  |  |  |  |  |  |  |  |  |  |  |  |  |  |  |  |  |  |  |  |  |  |  |  |  |  |  |  |  |  |  |  |  |  |  |  |  |  |  |  |  |  |  |  |  |  |  |  |  |  |  |  |  |  |  |  |  |  |  |  |  |  |  |  |  |  |  |  |  |  |  |  |  |  |  |  |  |  |  |  |  |  |  |  |  |  |  |  |  |  |  |  |  |  |  |  |  |  |  |  |  |  |  |  |  |  |  |  |  |  |  |  |  |  |  |  |  |  |  |  |  |  |  |  |  |  |  |  |  |  |  |  |  |  |  |  |  |  |  |  |  |  |  |  |  |  |  |  |  |  |  |  |  |  |  |  |  |  |  |  |  |  |  |  |  |  |  |  |  |  |  |  |  |  |  |  |  |  |  |  |  |  |  |  |  |  |  |  |  |  |  |  |  |  |  |  |  |  |  |  |  |  |  |  |  |  |  |  |  |  |  |  |  |  |  |  |  |  |  |  |  |  |  |  |  |  |  |  |  |  |  |  |  |  |  |  |  |  |  |  |  |  |  |  |  |  |  |  |  |  |  |  |  |  |  |  |  |  |  |  |  |  |  |  |  |  |  |  |  |  |  |  |  |  |  |  |  |  |  |  |  |  |  |  |  |  |  |  |  |  |  |  |  |  |  |  |  |  |  |  |  |  |  |  |  |  |  |  |  |  |  |  |  |  |  |  |  |  |  |  |  |  |  |  |  |  |  |  |  |  |  |  |  |  |  |  |  |  |  |  |  |  |  |  |  |  |  |  |  |  |  |  |  |  |  |  |  |  |  |  |  |  |  |  |  |  |  |  |  |  |  |  |  |  |  |  |  |  |  |  |  |  |  |  |  |  |  |  |  |  |  |  |  |  |  |  |  |  |  |  |  |  |  |  |  |  |  |  |  |  |  |  |  |  |  |  |  |  |  |  |  |  |  |  |  |  |  |  |  |  |  |  |  |  |  |  |  |  |  |  |  |  |  |  |  |  |  |  |  |  |  |  |  |  |  |  |  |  |  |  |  |  |  |  |  |  |  |  |  |  |  |  |  |  |  |  |  |  |  |  |  |  |  |  |  |  |  |  |  |  |  |  |  |  |  |  |  |  |  |  |  |  |  |  |  |  |  |  |  |  |  |  |  |  |  |  |  |  |  |  |  |  |  |  |  |  |  |  |  |  |  |  |  |  |  |  |  |  |  |  |  |  |  |  |  |  |  |  |  |  |  |  |  |  |  |  |  |  |  |  |  |  |  |  |  |  |  |  |  |  |  |  |  |  |  |  |  |  |  |  |  |  |  |  | | --- | --- | --- | --- | --- | --- | --- | --- | --- | --- | --- | --- | --- | --- | --- | --- | --- | --- | --- | --- | --- | --- | --- | --- | --- | --- | --- | --- | --- | --- | --- | --- | --- | --- | --- | --- | --- | --- | --- | --- | --- | --- | --- | --- | --- | --- | --- | --- | --- | --- | --- | --- | --- | --- | --- | --- | --- | --- | --- | --- | --- | --- | --- | --- | --- | --- | --- | --- | --- | --- | --- | --- | --- | --- | --- | --- | --- | --- | --- | --- | --- | --- | --- | --- | --- | --- | --- | --- | --- | --- | --- | --- | --- | --- | --- | --- | --- | --- | --- | --- | --- | --- | --- | --- | --- | --- | --- | --- | --- | --- | --- | --- | --- | --- | --- | --- | --- | --- | --- | --- | --- | --- | --- | --- | --- | --- | --- | --- | --- | --- | --- | --- | --- | --- | --- | --- | --- | --- | --- | --- | --- | --- | --- | --- | --- | --- | --- | --- | --- | --- | --- | --- | --- | --- | --- | --- | --- | --- | --- | --- | --- | --- | --- | --- | --- | --- | --- | --- | --- | --- | --- | --- | --- | --- | --- | --- | --- | --- | --- | --- | --- | --- | --- | --- | --- | --- | --- | --- | --- | --- | --- | --- | --- | --- | --- | --- | --- | --- | --- | --- | --- | --- | --- | --- | --- | --- | --- | --- | --- | --- | --- | --- | --- | --- | --- | --- | --- | --- | --- | --- | --- | --- | --- | --- | --- | --- | --- | --- | --- | --- | --- | --- | --- | --- | --- | --- | --- | --- | --- | --- | --- | --- | --- | --- | --- | --- | --- | --- | --- | --- | --- | --- | --- | --- | --- | --- | --- | --- | --- | --- | --- | --- | --- | --- | --- | --- | --- | --- | --- | --- | --- | --- | --- | --- | --- | --- | --- | --- | --- | --- | --- | --- | --- | --- | --- | --- | --- | --- | --- | --- | --- | --- | --- | --- | --- | --- | --- | --- | --- | --- | --- | --- | --- | --- | --- | --- | --- | --- | --- | --- | --- | --- | --- | --- | --- | --- | --- | --- | --- | --- | --- | --- | --- | --- | --- | --- | --- | --- | --- | --- | --- | --- | --- | --- | --- | --- | --- | --- | --- | --- | --- | --- | --- | --- | --- | --- | --- | --- | --- | --- | --- | --- | --- | --- | --- | --- | --- | --- | --- | --- | --- | --- | --- | --- | --- | --- | --- | --- | --- | --- | --- | --- | --- | --- | --- | --- | --- | --- | --- | --- | --- | --- | --- | --- | --- | --- | --- | --- | --- | --- | --- | --- | --- | --- | --- | --- | --- | --- | --- | --- | --- | --- | --- | --- | --- | --- | --- | --- | --- | --- | --- | --- | --- | --- | --- | --- | --- | --- | --- | --- | --- | --- | --- | --- | --- | --- | --- | --- | --- | --- | --- | --- | --- | --- | --- | --- | --- | --- | --- | --- | --- | --- | --- | --- | --- | --- | --- | --- | --- | --- | --- | --- | --- | --- | --- | --- | --- | --- | --- | --- | --- | --- | --- | --- | --- | --- | --- | --- | --- | --- | --- | --- | --- | --- | --- | --- | --- | --- | --- | --- | --- | --- | --- | --- | --- | --- | --- | --- | --- | --- | --- | --- | --- | --- | --- | --- | --- | --- | --- | --- | --- | --- | --- | --- | --- | --- | --- | --- | --- | --- | --- | --- | --- | --- | --- | --- | --- | --- | --- | --- | --- | --- | --- | --- | --- | --- | --- | --- | --- | --- | --- | --- | --- | --- | --- | --- | --- | --- | --- | --- | --- | --- | --- | --- | --- | --- | --- | --- | --- | --- | --- | --- | --- | --- | --- | --- | --- | --- | --- | --- | --- | --- | --- | --- | --- | --- | --- | --- | --- | --- | --- | --- | --- | --- | --- | --- | --- | --- | --- | --- | --- | --- | --- | --- | --- | --- | --- | --- | --- | --- | --- | --- | --- | --- | --- | --- | --- | --- | --- | --- | --- | --- | --- | --- | --- | --- | --- | --- | --- | --- | --- | --- | --- | --- | --- | --- | --- | --- | --- | --- | --- | --- | --- | --- | --- | --- | --- | --- | --- | --- | --- | --- | --- | --- | --- | --- | --- | --- | --- | --- | --- | --- | --- | --- | --- | --- | --- | --- | --- | --- | --- | --- | --- | --- | --- | --- | --- | --- | --- | --- | --- | --- | --- | --- | --- | --- | --- | --- | --- | --- | --- | --- | --- | --- | --- | --- | --- | --- | --- | --- | --- | --- | --- | --- | --- | --- | --- | --- | --- | --- | --- | --- | --- | --- | --- | --- | --- | --- | --- | --- | --- | --- | --- | --- | --- | --- | --- | --- | --- | --- | --- | --- | --- | --- | --- | --- | --- | --- | --- | --- | --- | --- | --- | --- | --- | --- | --- | --- | --- | --- | --- | --- | --- | --- | --- | --- | --- | --- | --- | --- | --- | --- | --- | --- | --- | --- | --- | --- | --- | --- | --- | --- | --- | --- | --- | --- | --- | --- | --- | --- | --- | --- | --- | --- | --- | --- | --- | --- | --- | --- | --- | --- | --- | --- | --- | --- | --- | --- | --- | --- | --- | --- | --- | --- | --- | --- | --- | --- | --- | --- | --- | --- | --- | --- | --- | --- | --- | --- | --- | --- | --- | --- | --- | --- | --- | --- | --- | --- | --- | --- | --- | --- | --- | --- | --- | --- | --- | --- | --- | --- | --- | --- | --- | --- | --- | --- | --- | --- | --- | --- | --- | --- | --- | --- | --- | --- | --- | --- | --- | --- | --- | --- | --- | --- | --- | --- | --- | --- | --- | --- | --- | --- | --- | --- | --- | --- | --- | --- | --- | --- | --- | --- | --- | --- | --- | --- | --- | --- | --- | --- | --- | --- | --- | --- | --- | --- | --- | --- | --- | --- | --- | --- | --- | --- | --- | --- | --- | --- | --- | --- | --- | --- | --- | --- | --- | --- | --- | --- | --- | --- | --- | --- | --- | --- | --- | --- | --- | --- | --- | --- | --- | --- | --- | --- | --- | --- | --- | --- | --- | --- | --- | --- | --- | --- | --- | --- | --- | --- | --- | --- | --- | --- | --- | --- | --- | --- | --- | --- | --- | --- | --- | --- | --- | --- | --- | --- | --- | --- | --- | --- | --- | --- | --- | --- | --- | --- | --- | --- | --- | --- | --- | --- | --- | --- | --- | --- | --- | --- | --- | --- | --- | --- | --- | --- | --- | --- | --- | --- | --- | --- | --- | --- | --- | --- | --- | --- | --- | --- | --- | --- | --- | --- | --- | --- | --- | --- | --- | --- | --- | --- | --- | --- | --- | --- | --- | --- | --- | --- | --- | --- | --- | --- | --- | --- | --- | --- | --- | --- | --- | --- | --- | --- | --- | --- | --- | --- | --- | --- | --- | --- | --- | --- | --- | --- | --- | --- | --- | --- | --- | --- | --- | --- | --- | --- | --- | --- | --- | --- | --- | --- | --- | --- | --- | --- | --- | --- | --- | --- | --- | --- | --- | --- | --- | --- | --- | --- | --- | --- | --- | --- | --- | --- | --- | --- | --- | --- | --- | --- | --- | --- | --- | --- | --- | --- | --- | --- | --- | --- | --- | --- | --- | --- | --- | --- | --- | --- | --- | --- | --- | --- | --- | --- | --- | --- | --- | --- | --- | --- | --- | --- | --- | --- | --- | --- | --- | --- | --- | --- | --- | --- | --- | --- | --- | --- | --- | --- | --- | --- | --- | --- | --- | --- | --- | --- | --- | --- | --- | --- | --- | --- | --- | --- | --- | --- | --- | --- | --- | --- | --- | --- | --- | --- | --- | --- | --- | --- | --- | --- | --- | --- | --- | --- | --- | --- | --- | --- | --- | --- | --- | --- | --- | --- | --- | --- | --- | --- | --- | --- | --- | --- | --- | --- | --- | --- | --- | --- | --- | --- | --- | --- | --- | --- | --- | --- | --- | --- | --- | --- | --- | --- | --- | --- | --- | --- | --- | --- | --- | --- | --- | --- | --- | --- | --- | --- | --- | --- | --- | --- | --- | --- | --- | --- | --- | --- | --- | --- | --- | --- | --- | --- | --- | --- | --- | --- | --- | --- | --- | --- | --- | --- | --- | --- | --- | --- | --- | --- | --- | --- | --- | --- | --- | --- | --- | --- | --- | --- | --- | --- | --- | --- | --- | --- | --- | --- | --- | --- | --- | --- | --- | --- | --- | --- | --- | --- | --- | --- | --- | --- | --- | --- | --- | --- | --- | --- | --- | --- | --- | --- | --- | --- | --- | --- | --- | --- | --- | --- | --- | --- | --- | --- | --- | --- | --- | --- | --- | --- | --- | --- | --- | --- | --- | --- | --- | --- | --- | --- | --- | --- | --- | --- | --- | --- | --- | --- | --- | --- | --- | --- | --- | --- | --- | --- | --- | --- | --- | --- | --- | --- | --- | --- | --- | --- | --- | --- | --- | --- | --- | --- | --- | --- | --- | --- | --- | --- | --- | --- | --- | --- | --- | --- | --- | --- | --- | --- | --- | --- | --- | --- | --- | --- | --- | --- | --- | --- | --- | --- | --- | --- | --- | --- | --- | --- | --- | --- | --- | --- | --- | --- | --- | --- | --- | --- | --- | --- | --- | --- | --- | --- | --- | --- | --- | --- | --- | --- | --- | --- | --- | --- | --- | --- | --- | --- | --- | --- | --- | --- | --- | --- | --- | --- | --- | --- | --- | --- | --- | --- | --- | --- | --- | --- | --- | --- | --- | --- | --- | --- | --- | --- | --- | --- | --- | --- | --- | --- | --- | --- | --- | --- | --- | --- | --- | --- | --- | --- | --- | --- | --- | --- | --- | --- | --- | --- | --- | --- | --- | --- | --- | --- | --- | --- | --- | --- | --- | --- | --- | --- | --- | --- | --- | --- | --- | --- | --- | --- | --- | --- | --- | --- | --- | --- | --- | --- | --- | --- | --- | --- | --- | --- | --- | --- | --- | --- | --- | --- | --- | --- | --- | --- | --- | --- | --- | --- | --- | --- | --- | --- | --- | --- | --- | --- | --- | --- | --- | --- | --- | --- | --- | --- | --- | --- | --- | --- | --- | --- | --- | --- | --- | --- | --- | --- | --- | --- | --- | --- | --- | --- | --- | --- | --- | --- | --- | --- | --- | --- | --- | --- | --- | --- | --- | --- | --- | --- | --- | --- | --- | --- | --- | --- | --- | --- | --- | --- | --- | --- | --- | --- | --- | --- | --- | --- | --- | --- | --- | --- | --- | --- | --- | --- | --- | --- | --- | --- | --- | --- | --- | --- | --- | --- | --- | --- | --- | --- | --- | --- | --- | --- | --- | --- | --- | --- | --- | --- | --- | --- | --- | --- | --- | --- | --- | --- | --- | --- | --- | --- | --- | --- | --- | --- | --- | --- | --- | --- | --- | --- | --- | --- | --- | --- | --- | --- | --- | --- | --- | --- | --- | --- | --- | --- | --- | --- | --- | --- | --- | --- | --- | --- | --- | --- | --- | --- | --- | --- | --- | --- | --- | --- | --- | --- | --- | --- | | DAVID Functional Annotation Clustering      |  | | --- | | Functional Annotation Clustering | | Help and Manual | | Current Gene List: DavidMay18\_TPM82 | | Current Background: Drosophila melanogaster | | 210 DAVID IDs |  - Options          Classification Stringency    Custom   Lowest   Low   Medium   High   Highest   - |  |  |  |     | --- | --- | --- |     | Kappa Similarity | Similarity Term Overlap 3 4 5 6 7 8 9 10 | Similarity Threshold 0.20 0.25 0.30 0.35 0.40 0.45 0.50 0.55 0.60 0.65 0.70 0.75 0.80 0.85 0.90 0.95 1.00 |     | Classification | Initial Group Membership 2 3 4 5 6 7 8 9 10 | Final Group Membership 2 3 4 5 6 7 8 9 10 | Multiple Linkage Threshold 0.00 0.05 0.10 0.15 0.20 0.25 0.30 0.35 0.40 0.45 0.50 0.55 0.60 0.65 0.70 0.75 0.80 0.85 0.90 0.95 1.00 |  |     | Enrichment Thresholds | EASE |  |  |  |     |     | Display | Fold Change | Bonferroni | Benjamini | FDR | LT,PH,PT |  |  |  |  | | --- | --- | --- | |  |  |  |     |  |  | | --- | --- | | 26 Cluster(s) | Download File |  | Annotation Cluster 1 | | Enrichment Score: 1.04 |  |  | Count | P\_Value | Benjamini | | --- | --- | --- | --- | --- | --- | --- | --- | |  | GOTERM\_BP\_5 | G-protein coupled receptor signaling pathway | **RT** |  | 6 | 7.3E-2 | 1.0E0 | |  | GOTERM\_MF\_5 | neuropeptide receptor activity | **RT** |  | 3 | 7.8E-2 | 1.0E0 | |  | GOTERM\_MF\_5 | G-protein coupled peptide receptor activity | **RT** |  | 3 | 8.8E-2 | 9.9E-1 | |  | GOTERM\_MF\_5 | G-protein coupled receptor activity | **RT** |  | 4 | 1.4E-1 | 9.9E-1 | | Annotation Cluster 2 | | Enrichment Score: 0.97 |  |  | Count | P\_Value | Benjamini | | --- | --- | --- | --- | --- | --- | --- | --- | |  | GOTERM\_CC\_5 | plasma membrane part | **RT** |  | 15 | 3.5E-2 | 1.0E0 | |  | GOTERM\_CC\_5 | integral component of plasma membrane | **RT** |  | 9 | 1.8E-1 | 1.0E0 | |  | GOTERM\_CC\_5 | intrinsic component of plasma membrane | **RT** |  | 9 | 1.9E-1 | 9.9E-1 | | Annotation Cluster 3 | | Enrichment Score: 0.82 |  |  | Count | P\_Value | Benjamini | | --- | --- | --- | --- | --- | --- | --- | --- | |  | GOTERM\_CC\_5 | cytoplasmic, membrane-bounded vesicle | **RT** |  | 5 | 8.5E-2 | 1.0E0 | |  | GOTERM\_CC\_5 | intracellular vesicle | **RT** |  | 5 | 1.1E-1 | 1.0E0 | |  | GOTERM\_CC\_5 | cytoplasmic vesicle | **RT** |  | 5 | 1.1E-1 | 1.0E0 | |  | GOTERM\_BP\_5 | modulation of synaptic transmission | **RT** |  | 4 | 1.3E-1 | 1.0E0 | |  | GOTERM\_CC\_5 | exocytic vesicle | **RT** |  | 3 | 1.6E-1 | 1.0E0 | |  | GOTERM\_BP\_5 | synaptic signaling | **RT** |  | 6 | 1.8E-1 | 1.0E0 | |  | GOTERM\_BP\_5 | trans-synaptic signaling | **RT** |  | 6 | 1.8E-1 | 1.0E0 | |  | GOTERM\_BP\_5 | neurotransmitter transport | **RT** |  | 4 | 1.8E-1 | 1.0E0 | |  | GOTERM\_CC\_5 | secretory vesicle | **RT** |  | 3 | 1.9E-1 | 1.0E0 | |  | GOTERM\_CC\_5 | transport vesicle | **RT** |  | 3 | 2.5E-1 | 1.0E0 | | Annotation Cluster 4 | | Enrichment Score: 0.58 |  |  | Count | P\_Value | Benjamini | | --- | --- | --- | --- | --- | --- | --- | --- | |  | GOTERM\_BP\_5 | embryonic morphogenesis | **RT** |  | 6 | 1.2E-1 | 1.0E0 | |  | GOTERM\_BP\_5 | enzyme linked receptor protein signaling pathway | **RT** |  | 6 | 1.4E-1 | 1.0E0 | |  | GOTERM\_BP\_5 | morphogenesis of embryonic epithelium | **RT** |  | 4 | 1.4E-1 | 1.0E0 | |  | GOTERM\_BP\_5 | cell fate commitment | **RT** |  | 8 | 2.2E-1 | 1.0E0 | |  | GOTERM\_BP\_5 | dorsal closure | **RT** |  | 3 | 2.8E-1 | 1.0E0 | |  | GOTERM\_BP\_5 | cell fate determination | **RT** |  | 3 | 4.2E-1 | 1.0E0 | |  | GOTERM\_BP\_5 | morphogenesis of an epithelium | **RT** |  | 8 | 5.8E-1 | 1.0E0 | |  | GOTERM\_BP\_5 | embryo development ending in birth or egg hatching | **RT** |  | 3 | 6.9E-1 | 1.0E0 | | Annotation Cluster 5 | | Enrichment Score: 0.52 |  |  | Count | P\_Value | Benjamini | | --- | --- | --- | --- | --- | --- | --- | --- | |  | GOTERM\_BP\_5 | cell fate commitment | **RT** |  | 8 | 2.2E-1 | 1.0E0 | |  | GOTERM\_BP\_5 | lateral inhibition | **RT** |  | 4 | 3.5E-1 | 1.0E0 | |  | GOTERM\_BP\_5 | cell-cell signaling involved in cell fate commitment | **RT** |  | 4 | 3.5E-1 | 1.0E0 | | Annotation Cluster 6 | | Enrichment Score: 0.49 |  |  | Count | P\_Value | Benjamini | | --- | --- | --- | --- | --- | --- | --- | --- | |  | GOTERM\_BP\_5 | Malpighian tubule development | **RT** |  | 3 | 1.5E-1 | 1.0E0 | |  | GOTERM\_BP\_5 | renal tubule development | **RT** |  | 3 | 1.5E-1 | 1.0E0 | |  | GOTERM\_BP\_5 | renal system development | **RT** |  | 3 | 1.9E-1 | 1.0E0 | |  | GOTERM\_BP\_5 | urogenital system development | **RT** |  | 3 | 1.9E-1 | 1.0E0 | |  | GOTERM\_BP\_5 | morphogenesis of an epithelium | **RT** |  | 8 | 5.8E-1 | 1.0E0 | |  | GOTERM\_BP\_5 | tube morphogenesis | **RT** |  | 5 | 8.7E-1 | 1.0E0 | |  | GOTERM\_BP\_5 | epithelial tube morphogenesis | **RT** |  | 4 | 9.4E-1 | 1.0E0 | | Annotation Cluster 7 | | Enrichment Score: 0.49 |  |  | Count | P\_Value | Benjamini | | --- | --- | --- | --- | --- | --- | --- | --- | |  | GOTERM\_BP\_5 | transmembrane transport | **RT** |  | 10 | 7.9E-2 | 1.0E0 | |  | GOTERM\_BP\_5 | ion transmembrane transport | **RT** |  | 4 | 2.8E-1 | 1.0E0 | |  | GOTERM\_BP\_5 | ion transport | **RT** |  | 7 | 2.9E-1 | 1.0E0 | |  | GOTERM\_BP\_5 | inorganic ion transmembrane transport | **RT** |  | 3 | 4.5E-1 | 1.0E0 | |  | GOTERM\_BP\_5 | cation transport | **RT** |  | 4 | 5.7E-1 | 1.0E0 | |  | GOTERM\_MF\_5 | cation transmembrane transporter activity | **RT** |  | 4 | 7.4E-1 | 1.0E0 | | Annotation Cluster 8 | | Enrichment Score: 0.46 |  |  | Count | P\_Value | Benjamini | | --- | --- | --- | --- | --- | --- | --- | --- | |  | GOTERM\_BP\_5 | vesicle mediated transport in synapse | **RT** |  | 3 | 3.1E-1 | 1.0E0 | |  | GOTERM\_BP\_5 | establishment of synaptic vesicle localization | **RT** |  | 3 | 3.5E-1 | 1.0E0 | |  | GOTERM\_BP\_5 | synaptic vesicle transport | **RT** |  | 3 | 3.5E-1 | 1.0E0 | |  | GOTERM\_BP\_5 | synaptic vesicle localization | **RT** |  | 3 | 3.6E-1 | 1.0E0 | |  | GOTERM\_BP\_5 | establishment of vesicle localization | **RT** |  | 3 | 3.8E-1 | 1.0E0 | | Annotation Cluster 9 | | Enrichment Score: 0.44 |  |  | Count | P\_Value | Benjamini | | --- | --- | --- | --- | --- | --- | --- | --- | |  | GOTERM\_BP\_5 | positive regulation of cell communication | **RT** |  | 7 | 1.2E-1 | 1.0E0 | |  | GOTERM\_BP\_5 | positive regulation of signal transduction | **RT** |  | 6 | 1.9E-1 | 1.0E0 | |  | GOTERM\_BP\_5 | regulation of signal transduction | **RT** |  | 9 | 4.4E-1 | 1.0E0 | |  | GOTERM\_BP\_5 | positive regulation of intracellular signal transduction | **RT** |  | 3 | 4.8E-1 | 1.0E0 | |  | GOTERM\_BP\_5 | regulation of intracellular signal transduction | **RT** |  | 4 | 6.5E-1 | 1.0E0 | |  | GOTERM\_BP\_5 | intracellular signal transduction | **RT** |  | 6 | 7.6E-1 | 1.0E0 | | Annotation Cluster 10 | | Enrichment Score: 0.41 |  |  | Count | P\_Value | Benjamini | | --- | --- | --- | --- | --- | --- | --- | --- | |  | GOTERM\_BP\_5 | purine nucleoside metabolic process | **RT** |  | 4 | 3.4E-1 | 1.0E0 | |  | GOTERM\_BP\_5 | ribonucleoside metabolic process | **RT** |  | 4 | 3.6E-1 | 1.0E0 | |  | GOTERM\_BP\_5 | nucleoside metabolic process | **RT** |  | 4 | 4.1E-1 | 1.0E0 | |  | GOTERM\_BP\_5 | nucleobase-containing small molecule metabolic process | **RT** |  | 5 | 4.5E-1 | 1.0E0 | | Annotation Cluster 11 | | Enrichment Score: 0.38 |  |  | Count | P\_Value | Benjamini | | --- | --- | --- | --- | --- | --- | --- | --- | |  | GOTERM\_BP\_5 | gland development | **RT** |  | 5 | 2.2E-1 | 1.0E0 | |  | GOTERM\_BP\_5 | salivary gland development | **RT** |  | 4 | 2.9E-1 | 1.0E0 | |  | GOTERM\_BP\_5 | exocrine system development | **RT** |  | 4 | 2.9E-1 | 1.0E0 | |  | GOTERM\_MF\_5 | sequence-specific DNA binding | **RT** |  | 4 | 7.3E-1 | 1.0E0 | |  | GOTERM\_BP\_5 | tube morphogenesis | **RT** |  | 5 | 8.7E-1 | 1.0E0 | | Annotation Cluster 12 | | Enrichment Score: 0.31 |  |  | Count | P\_Value | Benjamini | | --- | --- | --- | --- | --- | --- | --- | --- | |  | GOTERM\_BP\_5 | exocytosis | **RT** |  | 3 | 2.5E-1 | 1.0E0 | |  | GOTERM\_BP\_5 | secretion by cell | **RT** |  | 3 | 6.7E-1 | 1.0E0 | |  | GOTERM\_BP\_5 | secretion | **RT** |  | 3 | 7.0E-1 | 1.0E0 | | Annotation Cluster 13 | | Enrichment Score: 0.31 |  |  | Count | P\_Value | Benjamini | | --- | --- | --- | --- | --- | --- | --- | --- | |  | GOTERM\_MF\_5 | endopeptidase activity | **RT** |  | 7 | 3.1E-1 | 1.0E0 | |  | GOTERM\_BP\_5 | proteolysis | **RT** |  | 10 | 5.3E-1 | 1.0E0 | |  | GOTERM\_MF\_5 | serine-type endopeptidase activity | **RT** |  | 4 | 5.7E-1 | 1.0E0 | |  | GOTERM\_MF\_5 | serine-type peptidase activity | **RT** |  | 4 | 6.3E-1 | 1.0E0 | | Annotation Cluster 14 | | Enrichment Score: 0.28 |  |  | Count | P\_Value | Benjamini | | --- | --- | --- | --- | --- | --- | --- | --- | |  | GOTERM\_BP\_5 | ion transport | **RT** |  | 7 | 2.9E-1 | 1.0E0 | |  | GOTERM\_MF\_5 | gated channel activity | **RT** |  | 3 | 4.9E-1 | 1.0E0 | |  | GOTERM\_MF\_5 | ion channel activity | **RT** |  | 3 | 7.2E-1 | 1.0E0 | |  | GOTERM\_MF\_5 | substrate-specific channel activity | **RT** |  | 3 | 7.4E-1 | 1.0E0 | | Annotation Cluster 15 | | Enrichment Score: 0.23 |  |  | Count | P\_Value | Benjamini | | --- | --- | --- | --- | --- | --- | --- | --- | |  | GOTERM\_BP\_5 | enzyme linked receptor protein signaling pathway | **RT** |  | 6 | 1.4E-1 | 1.0E0 | |  | GOTERM\_BP\_5 | gland development | **RT** |  | 5 | 2.2E-1 | 1.0E0 | |  | GOTERM\_CC\_5 | nucleus | **RT** |  | 28 | 4.3E-1 | 1.0E0 | |  | GOTERM\_BP\_5 | nucleobase-containing compound biosynthetic process | **RT** |  | 16 | 4.7E-1 | 1.0E0 | |  | GOTERM\_BP\_5 | regulation of RNA biosynthetic process | **RT** |  | 13 | 4.8E-1 | 1.0E0 | |  | GOTERM\_BP\_5 | RNA biosynthetic process | **RT** |  | 14 | 4.8E-1 | 1.0E0 | |  | GOTERM\_CC\_5 | intracellular membrane-bounded organelle | **RT** |  | 45 | 4.8E-1 | 1.0E0 | |  | GOTERM\_BP\_5 | negative regulation of cellular macromolecule biosynthetic process | **RT** |  | 6 | 5.4E-1 | 1.0E0 | |  | GOTERM\_BP\_5 | negative regulation of macromolecule biosynthetic process | **RT** |  | 6 | 5.4E-1 | 1.0E0 | |  | GOTERM\_CC\_5 | intracellular organelle | **RT** |  | 52 | 5.5E-1 | 1.0E0 | |  | GOTERM\_BP\_5 | regulation of macromolecule biosynthetic process | **RT** |  | 14 | 5.6E-1 | 1.0E0 | |  | GOTERM\_BP\_5 | negative regulation of cellular biosynthetic process | **RT** |  | 6 | 5.6E-1 | 1.0E0 | |  | GOTERM\_BP\_5 | negative regulation of RNA biosynthetic process | **RT** |  | 5 | 5.7E-1 | 1.0E0 | |  | GOTERM\_BP\_5 | negative regulation of transcription, DNA-templated | **RT** |  | 5 | 5.7E-1 | 1.0E0 | |  | GOTERM\_BP\_5 | regulation of RNA metabolic process | **RT** |  | 13 | 5.8E-1 | 1.0E0 | |  | GOTERM\_BP\_5 | positive regulation of gene expression | **RT** |  | 5 | 5.9E-1 | 1.0E0 | |  | GOTERM\_BP\_5 | regulation of transcription, DNA-templated | **RT** |  | 12 | 6.0E-1 | 1.0E0 | |  | GOTERM\_BP\_5 | negative regulation of RNA metabolic process | **RT** |  | 5 | 6.0E-1 | 1.0E0 | |  | GOTERM\_BP\_5 | negative regulation of nucleobase-containing compound metabolic process | **RT** |  | 5 | 6.4E-1 | 1.0E0 | |  | GOTERM\_BP\_5 | RNA metabolic process | **RT** |  | 19 | 6.4E-1 | 1.0E0 | |  | GOTERM\_BP\_5 | regulation of cellular macromolecule biosynthetic process | **RT** |  | 13 | 6.7E-1 | 1.0E0 | |  | GOTERM\_BP\_5 | transcription, DNA-templated | **RT** |  | 11 | 6.8E-1 | 1.0E0 | |  | GOTERM\_BP\_5 | negative regulation of gene expression | **RT** |  | 6 | 7.0E-1 | 1.0E0 | |  | GOTERM\_BP\_5 | negative regulation of macromolecule metabolic process | **RT** |  | 7 | 7.2E-1 | 1.0E0 | |  | GOTERM\_BP\_5 | nucleic acid metabolic process | **RT** |  | 20 | 7.4E-1 | 1.0E0 | |  | GOTERM\_BP\_5 | positive regulation of macromolecule metabolic process | **RT** |  | 6 | 7.5E-1 | 1.0E0 | |  | GOTERM\_BP\_5 | regulation of gene expression | **RT** |  | 13 | 8.0E-1 | 1.0E0 | |  | GOTERM\_BP\_5 | cellular macromolecule biosynthetic process | **RT** |  | 18 | 9.6E-1 | 1.0E0 | |  | GOTERM\_BP\_5 | regionalization | **RT** |  | 3 | 9.7E-1 | 1.0E0 | |  | GOTERM\_BP\_5 | post-embryonic organ development | **RT** |  | 3 | 9.8E-1 | 1.0E0 | |  | GOTERM\_CC\_5 | cytoplasm | **RT** |  | 34 | 9.9E-1 | 1.0E0 | | Annotation Cluster 16 | | Enrichment Score: 0.23 |  |  | Count | P\_Value | Benjamini | | --- | --- | --- | --- | --- | --- | --- | --- | |  | GOTERM\_CC\_5 | endoplasmic reticulum | **RT** |  | 7 | 3.3E-1 | 1.0E0 | |  | GOTERM\_CC\_5 | endoplasmic reticulum membrane | **RT** |  | 3 | 7.7E-1 | 1.0E0 | |  | GOTERM\_CC\_5 | endoplasmic reticulum part | **RT** |  | 3 | 8.2E-1 | 1.0E0 | | Annotation Cluster 17 | | Enrichment Score: 0.22 |  |  | Count | P\_Value | Benjamini | | --- | --- | --- | --- | --- | --- | --- | --- | |  | GOTERM\_BP\_5 | response to bacterium | **RT** |  | 4 | 4.4E-1 | 1.0E0 | |  | GOTERM\_BP\_5 | defense response to bacterium | **RT** |  | 3 | 6.4E-1 | 1.0E0 | |  | GOTERM\_BP\_5 | defense response to other organism | **RT** |  | 3 | 7.7E-1 | 1.0E0 | | Annotation Cluster 18 | | Enrichment Score: 0.17 |  |  | Count | P\_Value | Benjamini | | --- | --- | --- | --- | --- | --- | --- | --- | |  | GOTERM\_BP\_5 | nucleobase-containing small molecule metabolic process | **RT** |  | 5 | 4.5E-1 | 1.0E0 | |  | GOTERM\_BP\_5 | purine nucleotide metabolic process | **RT** |  | 3 | 7.0E-1 | 1.0E0 | |  | GOTERM\_BP\_5 | nucleotide metabolic process | **RT** |  | 3 | 8.0E-1 | 1.0E0 | |  | GOTERM\_BP\_5 | nucleoside phosphate metabolic process | **RT** |  | 3 | 8.0E-1 | 1.0E0 | | Annotation Cluster 19 | | Enrichment Score: 0.14 |  |  | Count | P\_Value | Benjamini | | --- | --- | --- | --- | --- | --- | --- | --- | |  | GOTERM\_BP\_5 | positive regulation of gene expression | **RT** |  | 5 | 5.9E-1 | 1.0E0 | |  | GOTERM\_BP\_5 | transcription, DNA-templated | **RT** |  | 11 | 6.8E-1 | 1.0E0 | |  | GOTERM\_BP\_5 | positive regulation of transcription, DNA-templated | **RT** |  | 4 | 7.0E-1 | 1.0E0 | |  | GOTERM\_BP\_5 | positive regulation of RNA biosynthetic process | **RT** |  | 4 | 7.0E-1 | 1.0E0 | |  | GOTERM\_BP\_5 | positive regulation of RNA metabolic process | **RT** |  | 4 | 7.2E-1 | 1.0E0 | |  | GOTERM\_BP\_5 | positive regulation of macromolecule metabolic process | **RT** |  | 6 | 7.5E-1 | 1.0E0 | |  | GOTERM\_BP\_5 | positive regulation of macromolecule biosynthetic process | **RT** |  | 4 | 7.6E-1 | 1.0E0 | |  | GOTERM\_BP\_5 | positive regulation of nucleobase-containing compound metabolic process | **RT** |  | 4 | 7.6E-1 | 1.0E0 | |  | GOTERM\_BP\_5 | positive regulation of cellular biosynthetic process | **RT** |  | 4 | 8.2E-1 | 1.0E0 | | Annotation Cluster 20 | | Enrichment Score: 0.14 |  |  | Count | P\_Value | Benjamini | | --- | --- | --- | --- | --- | --- | --- | --- | |  | GOTERM\_BP\_5 | cellular protein catabolic process | **RT** |  | 4 | 6.0E-1 | 1.0E0 | |  | GOTERM\_BP\_5 | protein catabolic process | **RT** |  | 4 | 6.5E-1 | 1.0E0 | |  | GOTERM\_BP\_5 | cellular macromolecule catabolic process | **RT** |  | 4 | 7.8E-1 | 1.0E0 | |  | GOTERM\_BP\_5 | modification-dependent macromolecule catabolic process | **RT** |  | 3 | 7.9E-1 | 1.0E0 | |  | GOTERM\_BP\_5 | proteolysis involved in cellular protein catabolic process | **RT** |  | 3 | 8.2E-1 | 1.0E0 | | Annotation Cluster 21 | | Enrichment Score: 0.12 |  |  | Count | P\_Value | Benjamini | | --- | --- | --- | --- | --- | --- | --- | --- | |  | GOTERM\_CC\_5 | microtubule associated complex | **RT** |  | 5 | 5.1E-1 | 1.0E0 | |  | GOTERM\_CC\_5 | cytoskeletal part | **RT** |  | 6 | 7.5E-1 | 1.0E0 | |  | GOTERM\_CC\_5 | microtubule cytoskeleton | **RT** |  | 5 | 7.9E-1 | 1.0E0 | |  | GOTERM\_CC\_5 | cytoskeleton | **RT** |  | 6 | 8.3E-1 | 1.0E0 | |  | GOTERM\_CC\_5 | intracellular non-membrane-bounded organelle | **RT** |  | 12 | 9.9E-1 | 1.0E0 | | Annotation Cluster 22 | | Enrichment Score: 0.1 |  |  | Count | P\_Value | Benjamini | | --- | --- | --- | --- | --- | --- | --- | --- | |  | GOTERM\_BP\_5 | compound eye development | **RT** |  | 5 | 6.1E-1 | 1.0E0 | |  | GOTERM\_BP\_5 | eye development | **RT** |  | 5 | 6.6E-1 | 1.0E0 | |  | GOTERM\_BP\_5 | sensory organ development | **RT** |  | 5 | 8.2E-1 | 1.0E0 | |  | GOTERM\_BP\_5 | organ morphogenesis | **RT** |  | 8 | 8.8E-1 | 1.0E0 | |  | GOTERM\_BP\_5 | eye morphogenesis | **RT** |  | 3 | 8.8E-1 | 1.0E0 | |  | GOTERM\_BP\_5 | sensory organ morphogenesis | **RT** |  | 3 | 8.8E-1 | 1.0E0 | |  | GOTERM\_BP\_5 | animal organ development | **RT** |  | 12 | 9.2E-1 | 1.0E0 | | Annotation Cluster 23 | | Enrichment Score: 0.09 |  |  | Count | P\_Value | Benjamini | | --- | --- | --- | --- | --- | --- | --- | --- | |  | GOTERM\_MF\_5 | protein kinase activity | **RT** |  | 4 | 5.2E-1 | 1.0E0 | |  | GOTERM\_MF\_5 | GTP binding | **RT** |  | 3 | 5.6E-1 | 1.0E0 | |  | GOTERM\_MF\_5 | guanyl ribonucleotide binding | **RT** |  | 3 | 5.7E-1 | 1.0E0 | |  | GOTERM\_MF\_5 | guanyl nucleotide binding | **RT** |  | 3 | 5.7E-1 | 1.0E0 | |  | GOTERM\_BP\_5 | phosphorylation | **RT** |  | 7 | 6.0E-1 | 1.0E0 | |  | GOTERM\_MF\_5 | pyrophosphatase activity | **RT** |  | 5 | 8.7E-1 | 1.0E0 | |  | GOTERM\_MF\_5 | purine ribonucleoside binding | **RT** |  | 7 | 9.4E-1 | 1.0E0 | |  | GOTERM\_MF\_5 | purine ribonucleotide binding | **RT** |  | 7 | 9.4E-1 | 1.0E0 | |  | GOTERM\_MF\_5 | purine nucleotide binding | **RT** |  | 7 | 9.4E-1 | 1.0E0 | |  | GOTERM\_MF\_5 | ribonucleotide binding | **RT** |  | 7 | 9.4E-1 | 1.0E0 | |  | GOTERM\_BP\_5 | protein modification process | **RT** |  | 9 | 9.7E-1 | 1.0E0 | |  | GOTERM\_BP\_5 | cellular protein modification process | **RT** |  | 9 | 9.7E-1 | 1.0E0 | |  | GOTERM\_MF\_5 | ATP binding | **RT** |  | 4 | 9.9E-1 | 1.0E0 | |  | GOTERM\_MF\_5 | adenyl ribonucleotide binding | **RT** |  | 4 | 9.9E-1 | 1.0E0 | |  | GOTERM\_MF\_5 | adenyl nucleotide binding | **RT** |  | 4 | 9.9E-1 | 1.0E0 | |  | GOTERM\_BP\_5 | cellular protein metabolic process | **RT** |  | 13 | 1.0E0 | 1.0E0 | | Annotation Cluster 24 | | Enrichment Score: 0.06 |  |  | Count | P\_Value | Benjamini | | --- | --- | --- | --- | --- | --- | --- | --- | |  | GOTERM\_CC\_5 | nucleoplasm part | **RT** |  | 4 | 7.3E-1 | 1.0E0 | |  | GOTERM\_CC\_5 | nucleoplasm | **RT** |  | 5 | 7.6E-1 | 1.0E0 | |  | GOTERM\_CC\_5 | nuclear part | **RT** |  | 10 | 9.2E-1 | 1.0E0 | |  | GOTERM\_CC\_5 | nuclear lumen | **RT** |  | 6 | 9.6E-1 | 1.0E0 | |  | GOTERM\_CC\_5 | intracellular organelle lumen | **RT** |  | 6 | 9.9E-1 | 1.0E0 | | Annotation Cluster 25 | | Enrichment Score: 0.04 |  |  | Count | P\_Value | Benjamini | | --- | --- | --- | --- | --- | --- | --- | --- | |  | GOTERM\_BP\_5 | axon guidance | **RT** |  | 3 | 7.4E-1 | 1.0E0 | |  | GOTERM\_BP\_5 | neuron projection guidance | **RT** |  | 3 | 7.6E-1 | 1.0E0 | |  | GOTERM\_BP\_5 | axon development | **RT** |  | 3 | 8.6E-1 | 1.0E0 | |  | GOTERM\_BP\_5 | cell morphogenesis involved in neuron differentiation | **RT** |  | 4 | 8.8E-1 | 1.0E0 | |  | GOTERM\_BP\_5 | cell projection morphogenesis | **RT** |  | 5 | 9.1E-1 | 1.0E0 | |  | GOTERM\_BP\_5 | cell part morphogenesis | **RT** |  | 5 | 9.1E-1 | 1.0E0 | |  | GOTERM\_BP\_5 | animal organ development | **RT** |  | 12 | 9.2E-1 | 1.0E0 | |  | GOTERM\_BP\_5 | cell morphogenesis involved in differentiation | **RT** |  | 4 | 9.2E-1 | 1.0E0 | |  | GOTERM\_BP\_5 | cell morphogenesis | **RT** |  | 6 | 9.3E-1 | 1.0E0 | |  | GOTERM\_BP\_5 | neuron projection morphogenesis | **RT** |  | 4 | 9.4E-1 | 1.0E0 | |  | GOTERM\_BP\_5 | neuron projection development | **RT** |  | 4 | 9.5E-1 | 1.0E0 | |  | GOTERM\_BP\_5 | nervous system development | **RT** |  | 12 | 9.7E-1 | 1.0E0 | |  | GOTERM\_BP\_5 | neuron differentiation | **RT** |  | 5 | 9.8E-1 | 1.0E0 | |  | GOTERM\_BP\_5 | neuron development | **RT** |  | 4 | 9.9E-1 | 1.0E0 | |  | GOTERM\_BP\_5 | neurogenesis | **RT** |  | 9 | 9.9E-1 | 1.0E0 | |  | GOTERM\_BP\_5 | cell development | **RT** |  | 6 | 1.0E0 | 1.0E0 | | Annotation Cluster 26 | | Enrichment Score: 0.03 |  |  | Count | P\_Value | Benjamini | | --- | --- | --- | --- | --- | --- | --- | --- | |  | GOTERM\_BP\_5 | tube morphogenesis | **RT** |  | 5 | 8.7E-1 | 1.0E0 | |  | GOTERM\_BP\_5 | post-embryonic morphogenesis | **RT** |  | 4 | 9.5E-1 | 1.0E0 | |  | GOTERM\_BP\_5 | instar larval or pupal development | **RT** |  | 4 | 9.8E-1 | 1.0E0 |   were not clustered. | |  | |
