# Supplementary material for: Enabling cell-type-specific behavioral epigenetics in Drosophila: a modified high-yield INTACT method reveals the impact of social environment on the epigenetic landscape in dopaminergic neurons
Source: BMC Biol. 2019 Apr 10;17:30. doi: 10.1186/s12915-019-0646-4 (PMC6456965; doi:10.1186/s12915-019-0646-4)
Supplement: Supplementary file 10 — Gorilla and DAVID functional analysis. The zip file contains top level html files which may be opened in a browser. These will give the Gorilla functional analysis and DAVID GO analyses referred to in the main text. (ZIP 919 kb) [file 12915_2019_646_MOESM10_ESM.zip › Additional File 10/TPM85_GOLevel5.html]

DAVID: Database for Annotation, Visualization, and Integrated Discovery (Laboratory of Human Retrovirology and Immunoinformatics (LHRI); National Institute of Allergies and Infectious Diseases (NIAID); Leidos Biomedical Research, Inc. (LBR)


|  |  |  |  |  |  |
| --- | --- | --- | --- | --- | --- |
| DAVID Bioinformatics 6.7  |  |  |  |  |  | | --- | --- | --- | --- | --- | | |  |  |  |  | | --- | --- | --- | --- | |  | |  | | --- | | DAVID Bioinformatics Resources 6.8 | | Laboratory of Human Retrovirology and Immunoinformatics (LHRI) | | |   100%  **\*\*\* Welcome to DAVID 6.8 \*\*\* \*\*\* If you are looking for DAVID 6.7, please visit our development site. \*\*\*** |
| |  |  |  |  |  |  |  |  |  |  |  |  |  |  |  |  |  |  |  |  |  |  |  |  |  |  |  |  |  |  |  |  |  |  |  |  |  |  |  |  |  |  |  |  |  |  |  |  |  |  |  |  |  |  |  |  |  |  |  |  |  |  |  |  |  |  |  |  |  |  |  |  |  |  |  |  |  |  |  |  |  |  |  |  |  |  |  |  |  |  |  |  |  |  |  |  |  |  |  |  |  |  |  |  |  |  |  |  |  |  |  |  |  |  |  |  |  |  |  |  |  |  |  |  |  |  |  |  |  |  |  |  |  |  |  |  |  |  |  |  |  |  |  |  |  |  |  |  |  |  |  |  |  |  |  |  |  |  |  |  |  |  |  |  |  |  |  |  |  |  |  |  |  |  |  |  |  |  |  |  |  |  |  |  |  |  |  |  |  |  |  |  |  |  |  |  |  |  |  |  |  |  |  |  |  |  |  |  |  |  |  |  |  |  |  |  |  |  |  |  |  |  |  |  |  |  |  |  |  |  |  |  |  |  |  |  |  |  |  |  |  |  |  |  |  |  |  |  |  |  |  |  |  |  |  |  |  |  |  |  |  |  |  |  |  |  |  |  |  |  |  |  |  |  |  |  |  |  |  |  |  |  |  |  |  |  |  |  |  |  |  |  |  |  |  |  |  |  |  |  |  |  |  |  |  |  |  |  |  |  |  |  |  |  |  |  |  |  |  |  |  |  |  |  |  |  |  |  |  |  |  |  |  |  |  |  |  |  |  |  |  |  |  |  |  |  |  |  |  |  |  |  |  |  |  |  |  |  |  |  |  |  |  |  |  |  |  |  |  |  |  |  |  |  |  |  |  |  |  |  |  |  |  |  |  |  |  |  |  |  |  |  |  |  |  |  |  |  |  |  |  |  |  |  |  |  |  |  |  |  |  |  |  |  |  |  |  |  |  |  |  |  |  |  |  |  |  |  |  |  |  |  |  |  |  |  |  |  |  |  |  |  |  |  |  |  |  |  |  |  |  |  |  |  |  |  |  |  |  |  |  |  |  |  |  |  |  |  |  |  |  |  |  |  |  |  |  |  |  |  |  |  |  |  |  |  |  |  |  |  |  |  |  |  |  |  |  |  |  |  |  |  |  |  |  |  |  |  |  |  |  |  |  |  |  |  |  |  |  |  |  |  |  |  |  |  |  |  |  |  |  |  |  |  |  |  |  |  |  |  |  |  |  |  |  |  |  |  |  |  |  |  |  |  |  |  |  |  |  |  |  |  |  |  |  |  |  |  |  |  |  |  |  |  |  |  |  |  |  |  |  |  |  |  |  |  |  |  |  |  |  |  |  |  |  |  |  |  |  |  |  |  |  |  |  |  |  |  |  |  |  |  |  |  |  |  |  |  |  |  |  |  |  |  |  |  |  |  |  |  |  |  |  |  |  |  |  |  |  |  |  |  |  |  |  |  |  |  |  |  |  |  |  |  |  |  |  |  |  |  |  |  |  |  |  |  |  |  |  |  |  |  |  |  |  |  |  |  |  |  |  |  |  |  |  |  |  |  |  |  |  |  |  |  |  |  |  |  |  |  |  |  |  |  |  |  |  |  |  |  |  |  |  |  |  |  |  |  |  |  |  |  |  |  |  |  |  |  |  |  |  |  |  |  |  |  |  |  |  |  |  |  |  |  |  |  |  |  |  |  |  |  |  |  |  |  |  |  |  |  |  |  |  |  |  |  |  |  |  |  |  |  |  |  |  |  |  |  |  |  |  |  |  |  |  |  |  |  |  |  |  |  |  |  |  |  |  |  |  |  |  |  |  |  |  |  |  |  |  |  |  |  |  |  |  |  |  |  |  |  |  |  |  |  |  |  |  |  |  |  |  |  |  |  |  |  |  |  |  |  |  |  |  |  |  |  |  |  |  |  |  |  |  |  |  |  |  |  |  |  |  |  |  |  |  |  |  |  |  |  |  |  |  |  |  |  |  |  |  |  |  |  |  |  |  |  |  |  |  |  |  |  |  |  |  |  |  |  |  |  |  |  |  |  |  |  |  |  |  |  |  |  |  |  |  |  |  |  |  |  |  |  |  |  |  |  |  |  |  |  |  |  |  |  |  |  |  |  |  |  |  |  |  |  |  |  |  |  |  |  |  |  |  |  |  |  |  |  |  |  |  |  |  |  |  |  |  |  |  |  |  |  |  |  |  |  |  |  |  |  |  |  |  |  |  |  |  |  |  |  |  |  |  |  |  |  |  |  |  |  |  |  |  |  |  |  |  |  |  |  |  |  |  |  |  |  |  |  |  |  |  |  |  |  |  |  |  |  |  |  |  |  |  |  |  |  |  |  |  |  |  |  |  |  |  |  |  |  |  |  |  |  |  |  |  |  |  |  |  |  |  |  |  |  |  |  |  |  |  |  |  |  |  |  |  |  |  |  |  |  |  |  |  |  |  |  |  |  |  |  |  |  |  |  |  |  |  |  |  |  |  |  |  |  |  |  |  |  |  |  |  |  |  |  |  |  |  |  |  |  |  |  |  |  |  |  |  |  |  |  |  |  |  |  |  |  |  |  |  |  |  |  |  |  |  |  |  |  |  |  |  |  |  |  |  |  |  |  |  |  |  |  |  |  |  |  |  |  |  |  |  |  |  |  |  |  |  |  |  |  |  |  |  |  |  |  |  |  |  |  |  |  |  |  |  |  |  |  |  |  |  |  |  |  |  |  |  |  |  |  |  |  |  |  |  |  |  |  |  |  |  |  |  |  |  |  |  |  |  |  |  |  |  |  |  |  |  |  |  |  |  |  |  |  |  |  |  |  |  |  |  |  |  |  |  |  |  |  |  |  |  |  |  |  |  |  |  |  |  |  |  |  |  |  |  |  |  |  |  |  |  |  |  |  |  |  |  |  |  |  |  |  |  |  |  |  |  |  |  |  |  |  |  |  |  |  |  |  |  |  |  |  |  |  |  |  |  |  |  |  |  |  |  |  |  |  |  |  |  |  |  |  |  |  |  |  |  |  |  |  |  |  |  |  |  |  |  |  |  |  |  |  |  |  |  |  |  |  |  |  |  |  |  |  |  |  |  |  |  |  |  |  |  |  |  |  |  |  |  |  |  |  |  |  |  |  |  |  |  |  |  |  |  |  |  |  |  |  |  |  |  |  |  |  |  |  |  |  |  |  |  |  |  |  |  |  |  |  |  |  |  |  |  |  |  |  |  |  |  |  |  |  |  |  |  |  |  |  |  |  |  |  |  |  |  |  |  |  |  |  |  |  |  |  |  |  |  |  |  |  |  |  |  |  |  |  |  |  |  |  |  |  |  |  |  |  |  |  |  |  |  |  |  |  |  |  |  |  |  |  |  |  |  |  |  |  |  |  |  |  |  |  |  |  |  |  |  |  |  |  |  |  |  |  |  |  |  |  |  |  |  |  |  |  |  |  |  |  |  |  |  |  |  |  |  |  |  |  |  |  |  |  |  |  |  |  |  |  |  |  |  |  |  |  |  |  |  |  |  |  |  |  |  |  |  |  |  |  |  |  |  |  |  |  |  |  |  |  |  |  |  |  |  |  |  |  |  |  |  |  |  |  |  |  |  |  |  |  |  |  |  |  |  |  |  |  |  |  |  |  |  |  |  |  |  |  |  |  |  |  |  |  |  |  |  |  |  |  |  |  |  |  |  |  |  |  |  |  |  |  |  |  |  |  |  |  |  |  |  |  |  |  |  |  |  |  |  |  |  |  |  |  |  |  |  |  |  |  |  |  |  |  |  |  |  |  |  |  |  |  |  |  |  |  |  |  |  |  |  |  |  |  |  |  |  |  |  |  |  |  |  |  |  |  |  |  |  |  |  |  |  |  |  |  |  |  |  |  |  |  |  |  |  |  |  |  |  |  |  |  |  |  |  |  |  |  |  |  |  |  |  |  |  |  |  |  |  |  |  |  |  |  |  |  |  |  |  |  |  |  |  |  |  |  |  |  |  |  |  |  |  |  |  |  |  |  |  |  |  |  |  |  |  |  |  |  |  |  |  |  |  |  |  |  |  |  |  |  |  |  |  |  |  |  |  |  |  |  |  |  |  |  |  |  |  |  |  |  |  |  |  |  |  |  |  |  |  |  |  |  |  |  |  |  |  |  |  |  |  |  |  |  |  |  |  |  |  |  |  |  |  |  |  |  |  |  |  |  |  |  |  |  |  |  |  |  |  |  |  |  |  |  |  |  |  |  |  |  |  |  |  |  |  |  |  |  |  |  |  |  |  |  |  |  |  |  |  |  |  |  |  |  |  |  |  |  |  |  |  |  |  |  |  |  |  |  |  |  |  |  |  |  |  |  |  |  |  |  |  |  |  |  |  |  |  |  |  |  |  |  |  |  |  |  |  |  |  |  |  |  |  |  |  |  |  |  |  |  |  |  |  |  |  |  |  |  |  |  |  |  |  |  |  |  |  |  |  |  |  |  |  |  |  |  |  |  |  |  |  |  |  |  |  |  |  |  |  |  |  |  |  |  |  |  |  |  |  |  |  |  |  |  |  |  |  |  |  |  |  |  |  |  |  |  |  |  |  |  |  |  |  |  |  |  |  |  |  |  |  |  |  |  |  |  |  |  |  |  |  |  |  |  |  |  |  |  |  |  |  |  |  |  |  |  |  |  |  |  |  |  |  |  |  |  |  |  |  |  |  |  |  |  |  |  |  |  |  |  |  |  |  |  |  |  |  |  |  |  |  |  |  |  |  |  |  |  |  |  |  |  |  |  |  |  |  |  |  |  |  |  |  |  |  |  |  |  |  |  |  |  |  |  |  |  |  |  |  |  |  |  |  |  |  |  |  |  |  |  |  |  |  |  |  |  |  |  |  |  |  |  |  |  |  |  |  |  |  |  |  |  |  |  |  |  |  |  |  |  |  |  |  |  |  |  |  |  |  |  |  |  |  |  |  |  |  |  |  |  |  |  |  |  |  |  |  |  |  |  |  |  |  |  |  |  |  |  |  |  |  |  |  |  |  |  |  |  |  |  |  |  |  |  |  |  |  |  |  |  |  |  |  |  |  |  |  |  |  |  |  |  |  |  |  |  |  |  |  |  |  |  |  |  |  |  |  |  |  |  |  |  |  |  |  |  |  |  |  |  |  |  |  |  |  |  |  |  |  |  |  |  |  |  |  |  |  |  |  |  |  |  |  |  |  |  |  |  |  |  |  |  |  |  |  |  |  |  |  |  |  |  |  |  |  |  |  |  |  |  |  |  |  |  |  |  |  |  |  |  |  |  |  |  |  |  |  |  |  |  |  |  |  |  |  |  |  |  |  |  |  |  |  |  |  |  |  |  |  |  |  |  |  |  |  |  |  |  |  |  |  |  |  |  |  |  |  |  |  |  |  |  |  |  |  |  |  |  |  |  |  |  |  |  |  |  |  |  |  |  |  |  |  |  |  |  |  |  |  |  |  |  |  |  |  |  |  |  |  |  |  |  |  |  |  |  |  |  |  |  |  |  |  |  |  |  |  |  |  |  |  |  |  |  |  |  |  |  |  |  |  |  |  |  |  |  |  |  |  |  |  |  |  |  |  |  |  |  |  |  |  |  |  |  |  |  |  |  |  |  |  |  |  |  |  |  |  |  |  |  |  |  |  |  |  |  |  |  |  |  |  |  |  |  |  |  |  |  |  |  |  |  |  |  |  |  |  |  |  |  |  |  |  |  |  |  |  |  |  |  |  |  |  |  |  |  |  |  |  |  |  |  |  |  |  |  |  |  |  |  |  |  |  |  |  |  |  |  |  |  |  |  |  |  |  |  |  |  |  |  |  |  |  |  |  |  |  |  |  |  |  |  |  |  |  |  |  |  |  |  |  |  |  |  |  |  |  |  |  |  |  |  |  |  |  |  |  |  |  |  |  |  |  |  |  |  |  |  |  |  |  |  |  |  |  |  |  |  |  |  |  |  |  |  |  |  |  |  |  |  |  |  |  |  |  |  |  |  |  |  |  |  |  |  |  |  |  |  |  |  |  |  |  |  |  |  |  |  |  |  |  |  |  |  |  |  |  |  |  |  |  |  |  |  |  |  |  |  |  |  |  |  |  |  |  |  |  |  |  |  |  |  |  |  |  |  |  |  |  |  |  |  |  |  |  |  |  |  |  |  |  |  |  |  |  |  |  |  |  |  |  |  |  |  |  |  |  |  |  |  |  |  |  |  |  |  |  |  |  |  |  |  |  |  |  |  |  |  |  |  |  |  |  |  |  |  |  |  |  |  |  |  |  |  |  |  |  |  |  |  |  |  |  |  |  |  |  |  |  |  |  |  |  |  |  |  |  |  |  |  |  |  |  |  |  |  |  |  |  |  |  |  |  |  |  |  |  |  |  |  |  |  |  |  |  |  |  |  |  |  |  |  |  |  |  |  |  |  |  |  |  |  |  |  |  |  |  |  |  |  |  |  |  |  |  |  |  |  |  |  |  |  |  |  |  |  |  |  |  |  |  |  |  |  |  |  |  |  |  |  |  |  |  |  |  |  |  |  |  |  |  |  |  |  |  |  |  |  |  |  |  |  |  |  |  |  |  |  |  |  |  |  |  |  |  |  |  |  |  |  |  |  |  |  |  |  |  |  |  |  |  |  |  |  |  |  |  |  |  |  |  |  |  |  |  |  |  |  |  |  |  |  |  |  |  |  |  |  |  |  |  |  |  |  |  |  |  |  |  |  |  |  |  |  |  |  |  |  |  |  |  |  |  |  |  |  |  |  |  |  |  |  |  |  |  |  |  |  |  |  |  |  |  |  |  |  |  |  |  |  |  |  |  |  |  |  |  |  |  |  |  |  |  |  |  |  |  |  |  |  |  |  |  |  |  |  |  |  |  |  |  |  |  |  |  |  |  |  |  |  |  |  |  |  |  |  |  |  |  |  |  |  |  |  |  |  |  |  |  |  |  |  |  |  |  |  |  |  |  |  |  |  |  |  |  |  |  |  |  |  |  |  |  |  |  |  |  |  |  |  |  |  |  |  |  |  |  |  |  |  |  |  |  |  |  |  |  |  |  |  |  |  |  |  |  |  |  |  |  |  |  |  |  |  |  |  |  |  |  |  |  |  |  |  |  |  |  |  |  |  |  |  |  |  |  |  |  |  |  |  |  |  |  |  |  |  |  |  |  |  |  |  |  |  |  |  |  |  |  |  |  |  |  |  |  |  |  |  |  |  |  |  |  |  |  |  |  |  |  |  |  |  |  |  |  |  |  |  |  |  |  |  |  |  |  |  |  |  |  |  |  |  |  |  |  |  |  |  |  |  |  |  |  |  |  |  |  |  |  |  |  |  |  |  |  |  |  |  |  |  |  |  |  |  |  |  |  |  |  |  |  |  |  |  |  |  |  |  |  |  |  |  |  |  |  |  |  |  |  |  |  |  |  |  |  |  |  |  |  |  |  |  |  |  |  |  |  |  |  |  |  |  |  |  |  |  |  |  |  |  |  |  |  |  |  |  |  |  |  |  |  |  |  |  |  |  |  |  |  |  |  |  |  |  |  |  |  |  |  |  |  |  |  |  |  |  |  |  |  |  |  |  |  |  |  |  |  |  |  |  |  |  |  |  |  |  |  |  |  |  |  |  |  |  |  |  |  |  |  |  |  |  |  |  |  |  |  |  |  |  |  |  |  |  |  |  |  |  |  |  |  |  |  |  |  |  |  |  |  |  |  |  |  |  |  |  |  |  |  |  |  |  |  |  |  |  |  |  |  |  |  |  |  |  |  |  |  |  |  |  |  |  |  |  |  |  |  |  |  |  |  |  |  |  |  |  |  |  |  |  |  |  |  |  |  |  |  |  |  |  |  |  |  |  |  |  |  |  |  |  |  |  |  |  |  |  |  |  |  |  |  |  |  |  |  |  |  |  |  |  |  |  |  |  |  |  |  |  |  |  |  |  |  |  |  |  |  |  |  |  |  |  |  |  |  |  |  |  |  |  |  |  |  |  |  |  |  |  |  |  |  |  |  |  |  |  |  |  |  |  |  |  |  |  |  |  |  |  |  |  |  |  |  |  |  |  |  |  |  |  |  |  |  |  |  |  |  |  |  |  |  |  |  |  |  |  |  |  |  |  |  |  |  |  |  |  |  |  |  |  |  |  |  |  |  |  |  |  |  |  |  |  |  |  |  |  |  |  |  |  |  |  |  |  |  |  |  |  |  |  |  |  |  |  |  |  |  |  |  |  |  |  |  |  |  |  |  |  |  |  |  |  |  |  |  |  |  |  |  |  |  |  |  |  |  |  |  |  |  |  |  |  |  |  |  |  |  |  |  |  |  |  |  |  |  |  |  |  |  |  |  |  |  |  |  |  |  |  |  |  |  |  |  |  |  |  |  |  |  |  |  |  |  |  |  |  |  |  |  |  |  |  |  |  |  |  |  |  |  |  |  |  |  |  |  |  |  |  |  |  |  |  |  |  |  |  |  |  |  |  |  |  |  |  |  |  |  |  |  |  |  |  |  |  |  |  |  |  |  |  |  |  |  |  |  |  |  |  |  |  |  |  |  |  |  |  |  |  |  |  |  |  |  |  |  |  |  |  |  |  |  |  |  |  |  |  |  |  |  |  |  |  |  |  |  |  |  |  |  |  |  |  |  |  |  |  |  |  |  |  |  |  |  |  |  |  |  |  |  |  |  |  |  |  |  |  |  |  |  |  |  |  |  |  |  |  |  |  |  |  |  |  |  |  |  |  |  |  |  |  |  |  |  |  |  |  |  |  |  |  |  |  |  |  |  |  |  |  |  |  |  |  |  |  |  |  |  |  |  |  |  |  |  |  |  |  |  |  |  |  |  |  |  |  |  |  |  |  |  |  |  |  |  |  |  |  |  |  |  |  |  |  |  |  |  |  |  |  |  |  |  |  |  |  |  |  |  |  |  |  |  |  |  |  |  |  |  |  |  |  |  |  |  |  |  |  |  |  |  |  |  |  |  |  |  |  |  |  |  |  |  |  |  |  |  |  |  |  |  |  |  |  |  |  |  |  |  |  |  |  |  |  |  |  |  |  |  |  |  |  |  |  |  |  |  |  |  |  |  |  |  |  |  |  |  |  |  |  |  |  |  |  |  |  |  |  |  |  |  |  |  |  |  |  |  |  |  |  |  |  |  |  |  |  |  |  |  |  |  |  |  |  |  |  |  |  |  |  |  |  |  |  |  |  |  |  |  |  |  |  |  |  |  |  |  |  |  |  |  |  |  |  |  |  |  |  |  |  |  |  |  |  |  |  |  |  |  |  |  |  |  |  |  |  |  |  |  |  |  |  |  |  |  |  |  |  |  |  |  |  |  |  |  |  |  |  |  |  |  |  |  |  |  |  |  |  |  |  |  |  |  |  |  |  |  |  |  |  |  |  |  |  |  |  |  |  |  |  |  |  |  |  |  |  |  |  |  |  |  |  |  |  |  |  |  |  |  |  |  |  |  |  |  |  |  |  |  |  |  |  |  |  |  |  |  |  |  |  |  |  |  |  |  |  |  |  |  |  |  |  |  |  |  |  |  |  |  |  |  |  |  |  |  |  |  |  |  |  |  |  |  |  |  |  |  |  |  |  |  |  |  |  |  |  |  |  |  |  |  |  |  |  |  |  |  |  |  |  |  |  |  |  |  |  |  |  |  |  |  |  |  |  |  |  |  |  |  |  |  |  |  |  |  |  |  |  |  |  |  |  |  |  |  |  |  |  |  |  |  |  |  |  |  |  |  |  |  |  |  |  |  |  |  |  |  |  |  |  |  |  |  |  |  |  |  |  |  |  |  |  |  |  |  |  |  |  |  |  |  |  |  |  |  |  |  |  |  |  |  |  |  |  |  |  |  |  |  |  |  |  |  |  |  |  |  |  |  |  |  |  |  |  |  |  |  |  |  |  |  |  |  |  |  |  |  |  |  |  |  |  |  |  |  |  |  |  |  |  |  |  |  |  |  |  |  |  |  |  |  |  |  |  |  |  |  |  |  |  |  |  |  |  |  |  |  |  |  |  |  |  |  |  |  |  |  |  |  |  |  |  |  |  |  |  |  |  |  |  |  |  |  |  |  |  |  |  |  |  |  |  |  |  |  |  |  |  |  |  |  |  |  |  |  |  |  |  |  |  |  |  |  |  |  |  |  |  |  |  |  |  |  |  |  |  |  |  |  |  |  |  |  |  |  |  |  |  |  |  |  |  |  |  |  |  |  |  |  |  |  |  |  |  |  |  |  |  |  |  |  |  |  |  |  |  |  |  |  |  |  |  |  |  |  |  |  |  |  |  |  |  |  |  |  |  |  |  |  |  |  |  |  |  |  |  |  |  |  |  |  |  |  |  |  |  |  |  |  |  |  |  |  |  |  |  |  |  |  |  |  |  |  |  |  |  |  |  |  |  |  |  |  |  |  |  |  |  |  |  |  |  |  |  |  |  |  |  |  |  |  |  |  |  |  |  |  |  |  |  |  |  |  |  |  |  |  |  |  |  |  |  |  |  |  |  |  |  |  |  |  |  |  |  |  |  |  |  |  |  |  |  |  |  |  |  |  |  |  |  |  |  |  |  |  |  |  |  |  |  |  |  |  |  |  |  |  |  |  |  |  |  |  |  |  |  |  |  |  |  |  |  |  |  |  |  |  |  |  |  |  |  |  |  |  |  |  |  |  |  |  |  |  |  |  |  |  |  |  |  |  |  |  |  |  |  |  |  |  |  |  |  |  |  |  |  |  |  |  |  |  |  |  |  |  |  |  |  |  |  |  |  |  |  |  |  |  |  |  |  |  |  |  |  |  |  |  |  |  |  |  |  |  |  |  |  |  |  |  |  |  |  |  |  |  |  |  |  |  |  |  |  |  |  |  |  |  |  |  |  |  |  |  |  |  |  |  |  |  |  |  |  |  |  |  |  |  |  |  |  |  |  |  |  |  |  |  |  |  |  |  |  |  |  |  |  |  |  |  |  |  |  |  |  |  |  |  |  |  |  |  |  |  |  |  |  |  |  |  |  |  |  |  |  |  |  |  |  |  |  |  |  |  |  |  |  |  |  |  |  |  |  |  |  |  |  |  |  |  |  |  |  |  |  |  |  |  |  |  |  |  |  |  |  |  |  |  |  |  |  |  |  |  |  |  |  |  |  |  |  |  |  |  |  |  |  |  |  |  |  |  |  |  |  |  |  |  |  |  |  |  |  |  |  |  |  |  |  |  |  |  |  |  |  |  |  |  |  |  |  |  |  |  |  |  |  |  |  |  |  |  |  |  |  |  |  |  |  |  |  |  |  |  |  |  |  |  |  |  |  |  |  |  |  |  |  |  |  |  |  |  |  |  |  |  |  |  |  |  |  |  |  |  |  |  |  |  |  |  |  |  |  |  |  |  |  |  |  |  |  |  |  |  |  |  |  |  |  |  |  |  |  |  |  |  |  |  |  |  |  |  |  |  |  |  |  |  |  |  |  |  |  |  |  |  |  |  |  |  |  |  |  |  |  |  |  |  |  |  |  |  |  |  |  |  |  |  |  |  |  |  |  |  |  |  |  |  |  |  |  |  |  |  |  |  |  |  |  |  |  |  |  |  |  |  |  |  |  |  |  |  |  |  |  |  |  |  |  |  |  |  |  |  |  |  |  |  |  |  |  |  |  |  |  |  |  |  |  |  |  |  |  |  |  |  |  |  |  |  |  |  |  |  |  |  |  |  |  |  |  |  |  |  |  |  |  |  |  |  |  |  |  |  |  |  |  |  |  |  |  |  |  |  |  |  |  |  |  |  |  |  |  |  |  |  |  |  |  |  |  |  |  |  |  |  |  |  |  |  |  |  |  |  |  |  |  |  |  |  |  |  |  |  |  |  |  |  |  |  |  |  |  |  |  |  |  |  |  |  |  |  |  |  |  |  |  |  |  |  |  |  |  |  |  |  |  |  |  |  |  |  |  |  |  |  |  |  |  |  |  |  |  |  |  |  |  |  |  |  |  |  |  |  |  |  |  |  |  |  |  |  |  |  |  |  |  |  |  |  |  |  |  |  |  |  |  |  |  |  |  |  |  |  |  |  |  |  |  |  |  |  |  |  |  |  |  |  |  |  |  |  |  |  |  |  |  |  |  |  |  |  |  |  |  |  |  |  |  |  |  |  |  |  |  |  |  |  |  |  |  |  |  |  |  |  |  |  |  |  |  |  |  |  |  |  |  |  |  |  |  |  |  |  |  |  |  |  |  |  |  |  |  |  |  |  |  |  |  |  |  |  |  |  |  |  |  |  |  |  |  |  |  |  |  |  |  |  |  |  |  |  |  |  |  |  |  |  |  |  |  |  |  |  |  |  |  |  |  |  |  |  |  |  |  |  |  |  |  |  |  |  |  |  |  |  |  |  |  |  |  |  |  |  |  |  |  |  |  |  |  |  |  |  |  |  |  |  |  |  |  |  |  |  |  |  |  |  |  |  |  |  |  |  |  |  |  |  |  |  |  |  |  |  |  |  |  |  |  |  |  |  |  |  |  |  |  |  |  |  |  |  |  |  |  |  |  |  |  |  |  |  |  |  |  |  |  |  |  |  |  |  |  |  |  |  |  |  |  |  |  |  |  |  |  |  |  |  |  |  |  |  |  |  |  |  |  |  |  |  |  |  |  |  |  |  |  |  |  |  |  |  |  |  |  |  |  |  |  |  |  |  |  |  |  |  |  |  |  |  |  |  |  |  |  |  |  |  |  |  |  |  |  |  |  |  |  |  |  |  |  |  |  |  |  |  |  |  |  |  |  |  |  |  |  |  |  |  |  |  |  |  |  |  |  |  |  |  |  |  |  |  |  |  |  |  |  |  |  |  |  |  |  |  |  |  |  |  |  |  |  |  |  |  |  |  |  |  |  |  |  |  |  |  |  |  |  |  |  |  |  |  |  |  |  |  |  |  |  |  |  |  |  |  |  |  |  |  |  |  |  |  |  |  |  |  |  |  |  |  |  |  |  |  |  |  |  |  |  |  |  |  |  |  |  |  |  |  |  |  |  |  |  |  |  |  |  |  |  |  |  |  |  |  |  |  |  |  |  |  |  |  |  | | --- | --- | --- | --- | --- | --- | --- | --- | --- | --- | --- | --- | --- | --- | --- | --- | --- | --- | --- | --- | --- | --- | --- | --- | --- | --- | --- | --- | --- | --- | --- | --- | --- | --- | --- | --- | --- | --- | --- | --- | --- | --- | --- | --- | --- | --- | --- | --- | --- | --- | --- | --- | --- | --- | --- | --- | --- | --- | --- | --- | --- | --- | --- | --- | --- | --- | --- | --- | --- | --- | --- | --- | --- | --- | --- | --- | --- | --- | --- | --- | --- | --- | --- | --- | --- | --- | --- | --- | --- | --- | --- | --- | --- | --- | --- | --- | --- | --- | --- | --- | --- | --- | --- | --- | --- | --- | --- | --- | --- | --- | --- | --- | --- | --- | --- | --- | --- | --- | --- | --- | --- | --- | --- | --- | --- | --- | --- | --- | --- | --- | --- | --- | --- | --- | --- | --- | --- | --- | --- | --- | --- | --- | --- | --- | --- | --- | --- | --- | --- | --- | --- | --- | --- | --- | --- | --- | --- | --- | --- | --- | --- | --- | --- | --- | --- | --- | --- | --- | --- | --- | --- | --- | --- | --- | --- | --- | --- | --- | --- | --- | --- | --- | --- | --- | --- | --- | --- | --- | --- | --- | --- | --- | --- | --- | --- | --- | --- | --- | --- | --- | --- | --- | --- | --- | --- | --- | --- | --- | --- | --- | --- | --- | --- | --- | --- | --- | --- | --- | --- | --- | --- | --- | --- | --- | --- | --- | --- | --- | --- | --- | --- | --- | --- | --- | --- | --- | --- | --- | --- | --- | --- | --- | --- | --- | --- | --- | --- | --- | --- | --- | --- | --- | --- | --- | --- | --- | --- | --- | --- | --- | --- | --- | --- | --- | --- | --- | --- | --- | --- | --- | --- | --- | --- | --- | --- | --- | --- | --- | --- | --- | --- | --- | --- | --- | --- | --- | --- | --- | --- | --- | --- | --- | --- | --- | --- | --- | --- | --- | --- | --- | --- | --- | --- | --- | --- | --- | --- | --- | --- | --- | --- | --- | --- | --- | --- | --- | --- | --- | --- | --- | --- | --- | --- | --- | --- | --- | --- | --- | --- | --- | --- | --- | --- | --- | --- | --- | --- | --- | --- | --- | --- | --- | --- | --- | --- | --- | --- | --- | --- | --- | --- | --- | --- | --- | --- | --- | --- | --- | --- | --- | --- | --- | --- | --- | --- | --- | --- | --- | --- | --- | --- | --- | --- | --- | --- | --- | --- | --- | --- | --- | --- | --- | --- | --- | --- | --- | --- | --- | --- | --- | --- | --- | --- | --- | --- | --- | --- | --- | --- | --- | --- | --- | --- | --- | --- | --- | --- | --- | --- | --- | --- | --- | --- | --- | --- | --- | --- | --- | --- | --- | --- | --- | --- | --- | --- | --- | --- | --- | --- | --- | --- | --- | --- | --- | --- | --- | --- | --- | --- | --- | --- | --- | --- | --- | --- | --- | --- | --- | --- | --- | --- | --- | --- | --- | --- | --- | --- | --- | --- | --- | --- | --- | --- | --- | --- | --- | --- | --- | --- | --- | --- | --- | --- | --- | --- | --- | --- | --- | --- | --- | --- | --- | --- | --- | --- | --- | --- | --- | --- | --- | --- | --- | --- | --- | --- | --- | --- | --- | --- | --- | --- | --- | --- | --- | --- | --- | --- | --- | --- | --- | --- | --- | --- | --- | --- | --- | --- | --- | --- | --- | --- | --- | --- | --- | --- | --- | --- | --- | --- | --- | --- | --- | --- | --- | --- | --- | --- | --- | --- | --- | --- | --- | --- | --- | --- | --- | --- | --- | --- | --- | --- | --- | --- | --- | --- | --- | --- | --- | --- | --- | --- | --- | --- | --- | --- | --- | --- | --- | --- | --- | --- | --- | --- | --- | --- | --- | --- | --- | --- | --- | --- | --- | --- | --- | --- | --- | --- | --- | --- | --- | --- | --- | --- | --- | --- | --- | --- | --- | --- | --- | --- | --- | --- | --- | --- | --- | --- | --- | --- | --- | --- | --- | --- | --- | --- | --- | --- | --- | --- | --- | --- | --- | --- | --- | --- | --- | --- | --- | --- | --- | --- | --- | --- | --- | --- | --- | --- | --- | --- | --- | --- | --- | --- | --- | --- | --- | --- | --- | --- | --- | --- | --- | --- | --- | --- | --- | --- | --- | --- | --- | --- | --- | --- | --- | --- | --- | --- | --- | --- | --- | --- | --- | --- | --- | --- | --- | --- | --- | --- | --- | --- | --- | --- | --- | --- | --- | --- | --- | --- | --- | --- | --- | --- | --- | --- | --- | --- | --- | --- | --- | --- | --- | --- | --- | --- | --- | --- | --- | --- | --- | --- | --- | --- | --- | --- | --- | --- | --- | --- | --- | --- | --- | --- | --- | --- | --- | --- | --- | --- | --- | --- | --- | --- | --- | --- | --- | --- | --- | --- | --- | --- | --- | --- | --- | --- | --- | --- | --- | --- | --- | --- | --- | --- | --- | --- | --- | --- | --- | --- | --- | --- | --- | --- | --- | --- | --- | --- | --- | --- | --- | --- | --- | --- | --- | --- | --- | --- | --- | --- | --- | --- | --- | --- | --- | --- | --- | --- | --- | --- | --- | --- | --- | --- | --- | --- | --- | --- | --- | --- | --- | --- | --- | --- | --- | --- | --- | --- | --- | --- | --- | --- | --- | --- | --- | --- | --- | --- | --- | --- | --- | --- | --- | --- | --- | --- | --- | --- | --- | --- | --- | --- | --- | --- | --- | --- | --- | --- | --- | --- | --- | --- | --- | --- | --- | --- | --- | --- | --- | --- | --- | --- | --- | --- | --- | --- | --- | --- | --- | --- | --- | --- | --- | --- | --- | --- | --- | --- | --- | --- | --- | --- | --- | --- | --- | --- | --- | --- | --- | --- | --- | --- | --- | --- | --- | --- | --- | --- | --- | --- | --- | --- | --- | --- | --- | --- | --- | --- | --- | --- | --- | --- | --- | --- | --- | --- | --- | --- | --- | --- | --- | --- | --- | --- | --- | --- | --- | --- | --- | --- | --- | --- | --- | --- | --- | --- | --- | --- | --- | --- | --- | --- | --- | --- | --- | --- | --- | --- | --- | --- | --- | --- | --- | --- | --- | --- | --- | --- | --- | --- | --- | --- | --- | --- | --- | --- | --- | --- | --- | --- | --- | --- | --- | --- | --- | --- | --- | --- | --- | --- | --- | --- | --- | --- | --- | --- | --- | --- | --- | --- | --- | --- | --- | --- | --- | --- | --- | --- | --- | --- | --- | --- | --- | --- | --- | --- | --- | --- | --- | --- | --- | --- | --- | --- | --- | --- | --- | --- | --- | --- | --- | --- | --- | --- | --- | --- | --- | --- | --- | --- | --- | --- | --- | --- | --- | --- | --- | --- | --- | --- | --- | --- | --- | --- | --- | --- | --- | --- | --- | --- | --- | --- | --- | --- | --- | --- | --- | --- | --- | --- | --- | --- | --- | --- | --- | --- | --- | --- | --- | --- | --- | --- | --- | --- | --- | --- | --- | --- | --- | --- | --- | --- | --- | --- | --- | --- | --- | --- | --- | --- | --- | --- | --- | --- | --- | --- | --- | --- | --- | --- | --- | --- | --- | --- | --- | --- | --- | --- | --- | --- | --- | --- | --- | --- | --- | --- | --- | --- | --- | --- | --- | --- | --- | --- | --- | --- | --- | --- | --- | --- | --- | --- | --- | --- | --- | --- | --- | --- | --- | --- | --- | --- | --- | --- | --- | --- | --- | --- | --- | --- | --- | --- | --- | --- | --- | --- | --- | --- | --- | --- | --- | --- | --- | --- | --- | --- | --- | --- | --- | --- | --- | --- | --- | --- | --- | --- | --- | --- | --- | --- | --- | --- | --- | --- | --- | --- | --- | --- | --- | --- | --- | --- | --- | --- | --- | --- | --- | --- | --- | --- | --- | --- | --- | --- | --- | --- | --- | --- | --- | --- | --- | --- | --- | --- | --- | --- | --- | --- | --- | --- | --- | --- | --- | --- | --- | --- | --- | --- | --- | --- | --- | --- | --- | --- | --- | --- | --- | --- | --- | --- | --- | --- | --- | --- | --- | --- | --- | --- | --- | --- | --- | --- | --- | --- | --- | --- | --- | --- | --- | --- | --- | --- | --- | --- | --- | --- | --- | --- | --- | --- | --- | --- | --- | --- | --- | --- | --- | --- | --- | --- | --- | --- | --- | --- | --- | --- | --- | --- | --- | --- | --- | --- | --- | --- | --- | --- | --- | --- | --- | --- | --- | --- | --- | --- | --- | --- | --- | --- | --- | --- | --- | --- | --- | --- | --- | --- | --- | --- | --- | --- | --- | --- | --- | --- | --- | --- | --- | --- | --- | --- | --- | --- | --- | --- | --- | --- | --- | --- | --- | --- | --- | --- | --- | --- | --- | --- | --- | --- | --- | --- | --- | --- | --- | --- | --- | --- | --- | --- | --- | --- | --- | --- | --- | --- | --- | --- | --- | --- | --- | --- | --- | --- | --- | --- | --- | --- | --- | --- | --- | --- | --- | --- | --- | --- | --- | --- | --- | --- | --- | --- | --- | --- | --- | --- | --- | --- | --- | --- | --- | --- | --- | --- | --- | --- | --- | --- | --- | --- | --- | --- | --- | --- | --- | --- | --- | --- | --- | --- | --- | --- | --- | --- | --- | --- | --- | --- | --- | --- | --- | --- | --- | --- | --- | --- | --- | --- | --- | --- | --- | --- | --- | --- | --- | --- | --- | --- | --- | --- | --- | --- | --- | --- | --- | --- | --- | --- | --- | --- | --- | --- | --- | --- | --- | --- | --- | --- | --- | --- | --- | --- | --- | --- | --- | --- | --- | --- | --- | --- | --- | --- | --- | --- | --- | --- | --- | --- | --- | --- | --- | --- | --- | --- | --- | --- | --- | --- | --- | --- | --- | --- | --- | --- | --- | --- | --- | --- | --- | --- | --- | --- | --- | --- | --- | --- | --- | --- | --- | --- | --- | --- | --- | --- | --- | --- | --- | --- | --- | --- | --- | --- | --- | --- | --- | --- | --- | --- | --- | --- | --- | --- | --- | --- | --- | --- | --- | --- | --- | --- | --- | --- | --- | --- | --- | --- | --- | --- | --- | --- | --- | --- | --- | --- | --- | --- | --- | --- | --- | --- | --- | --- | --- | --- | --- | --- | --- | --- | --- | --- | --- | --- | --- | --- | --- | --- | --- | --- | --- | --- | --- | --- | --- | --- | --- | --- | --- | --- | --- | --- | --- | --- | --- | --- | --- | --- | --- | --- | --- | --- | --- | --- | --- | --- | --- | --- | --- | --- | --- | --- | --- | --- | --- | --- | --- | --- | --- | --- | --- | --- | --- | --- | --- | --- | --- | --- | --- | --- | --- | --- | --- | --- | --- | --- | --- | --- | --- | --- | --- | --- | --- | --- | --- | --- | --- | --- | --- | --- | --- | --- | --- | --- | --- | --- | --- | --- | --- | --- | --- | --- | --- | --- | --- | --- | --- | --- | --- | --- | --- | --- | --- | --- | --- | --- | --- | --- | --- | --- | --- | --- | --- | --- | --- | --- | --- | --- | --- | --- | --- | --- | --- | --- | --- | --- | --- | --- | --- | --- | --- | --- | --- | --- | --- | --- | --- | --- | --- | --- | --- | --- | --- | --- | --- | --- | --- | --- | --- | --- | --- | --- | --- | --- | --- | --- | --- | --- | --- | --- | --- | --- | --- | --- | --- | --- | --- | --- | --- | --- | --- | --- | --- | --- | --- | --- | --- | --- | --- | --- | --- | --- | --- | --- | --- | --- | --- | --- | --- | --- | --- | --- | --- | --- | --- | --- | --- | --- | --- | --- | --- | --- | --- | --- | --- | --- | --- | --- | --- | --- | --- | --- | --- | --- | --- | --- | --- | --- | --- | --- | --- | --- | --- | --- | --- | --- | --- | --- | --- | --- | --- | --- | --- | --- | --- | --- | --- | --- | --- | --- | --- | --- | --- | --- | --- | --- | --- | --- | --- | --- | --- | --- | --- | --- | --- | --- | --- | --- | --- | --- | --- | --- | --- | --- | --- | --- | --- | --- | --- | --- | --- | --- | --- | --- | --- | --- | --- | --- | --- | --- | --- | --- | --- | --- | --- | --- | --- | --- | --- | --- | --- | --- | --- | --- | --- | --- | --- | --- | --- | --- | --- | --- | --- | --- | --- | --- | --- | --- | --- | --- | --- | --- | --- | --- | --- | --- | --- | --- | --- | --- | --- | --- | --- | --- | --- | --- | --- | --- | --- | --- | --- | --- | --- | --- | --- | --- | --- | --- | --- | --- | --- | --- | --- | --- | --- | --- | --- | --- | --- | --- | --- | --- | --- | --- | --- | --- | --- | --- | --- | --- | --- | --- | --- | --- | --- | --- | --- | --- | --- | --- | --- | --- | --- | --- | --- | --- | --- | --- | --- | --- | --- | --- | --- | --- | --- | --- | --- | --- | --- | --- | --- | --- | --- | --- | --- | --- | --- | --- | --- | --- | --- | --- | --- | --- | --- | --- | --- | --- | --- | --- | --- | --- | --- | --- | --- | --- | --- | --- | --- | --- | --- | --- | --- | --- | --- | --- | --- | --- | --- | --- | --- | --- | --- | --- | --- | --- | --- | --- | --- | --- | --- | --- | --- | --- | --- | --- | --- | --- | --- | --- | --- | --- | --- | --- | --- | --- | --- | --- | --- | --- | --- | --- | --- | --- | --- | --- | --- | --- | --- | --- | --- | --- | --- | --- | --- | --- | --- | --- | --- | --- | --- | --- | --- | --- | --- | --- | --- | --- | --- | --- | --- | --- | --- | --- | --- | --- | --- | --- | --- | --- | --- | --- | --- | --- | --- | --- | --- | --- | --- | --- | --- | --- | --- | --- | --- | --- | --- | --- | --- | --- | --- | --- | --- | --- | --- | --- | --- | --- | --- | --- | --- | --- | --- | --- | --- | --- | --- | --- | --- | --- | --- | --- | --- | --- | --- | --- | --- | --- | --- | --- | --- | --- | --- | --- | --- | --- | --- | --- | --- | --- | --- | --- | --- | --- | --- | --- | --- | --- | --- | --- | --- | --- | --- | --- | --- | --- | --- | --- | --- | --- | --- | --- | --- | --- | --- | --- | --- | --- | --- | --- | --- | --- | --- | --- | --- | --- | --- | --- | --- | --- | --- | --- | --- | --- | --- | --- | --- | --- | --- | --- | --- | --- | --- | --- | --- | --- | --- | --- | --- | --- | --- | --- | --- | --- | --- | --- | --- | --- | --- | --- | --- | --- | --- | --- | --- | --- | --- | --- | --- | --- | --- | --- | --- | --- | --- | --- | --- | --- | --- | --- | --- | --- | --- | --- | --- | --- | --- | --- | --- | --- | --- | --- | --- | --- | --- | --- | --- | --- | --- | --- | --- | --- | --- | --- | --- | --- | --- | --- | --- | --- | --- | --- | --- | --- | --- | --- | --- | --- | --- | --- | --- | --- | --- | --- | --- | --- | --- | --- | --- | --- | --- | --- | --- | --- | --- | --- | --- | --- | --- | --- | --- | --- | --- | --- | --- | --- | --- | --- | --- | --- | --- | --- | --- | --- | --- | --- | --- | --- | --- | --- | --- | --- | --- | --- | --- | --- | --- | --- | --- | --- | --- | --- | --- | --- | --- | --- | --- | --- | --- | --- | --- | --- | --- | --- | --- | --- | --- | --- | --- | --- | --- | --- | --- | --- | --- | --- | --- | --- | --- | --- | --- | --- | --- | --- | --- | --- | --- | --- | --- | --- | --- | --- | --- | --- | --- | --- | --- | --- | --- | --- | --- | --- | --- | --- | --- | --- | --- | --- | --- | --- | --- | --- | --- | --- | --- | --- | --- | --- | --- | --- | --- | --- | --- | --- | --- | --- | --- | --- | --- | --- | --- | --- | --- | --- | --- | --- | --- | --- | --- | --- | --- | --- | --- | --- | --- | --- | --- | --- | --- | --- | --- | --- | --- | --- | --- | --- | --- | --- | --- | --- | --- | --- | --- | --- | --- | --- | --- | --- | --- | --- | --- | --- | --- | --- | --- | --- | --- | --- | --- | --- | --- | --- | --- | --- | --- | --- | --- | --- | --- | --- | --- | --- | --- | --- | --- | --- | --- | --- | --- | --- | --- | --- | --- | --- | --- | --- | --- | --- | --- | --- | --- | --- | --- | --- | --- | --- | --- | --- | --- | --- | --- | --- | --- | --- | --- | --- | --- | --- | --- | --- | --- | --- | --- | --- | --- | --- | --- | --- | --- | --- | --- | --- | --- | --- | --- | --- | --- | --- | --- | --- | --- | --- | --- | --- | --- | --- | --- | --- | --- | --- | --- | --- | --- | --- | --- | --- | --- | --- | --- | --- | --- | --- | --- | --- | --- | --- | --- | --- | --- | --- | --- | --- | --- | --- | --- | --- | --- | --- | --- | --- | --- | --- | --- | --- | --- | --- | --- | --- | --- | --- | --- | --- | --- | --- | --- | --- | --- | --- | --- | --- | --- | --- | --- | --- | --- | --- | --- | --- | --- | --- | --- | --- | --- | --- | --- | --- | --- | --- | --- | --- | --- | --- | --- | --- | --- | --- | --- | --- | --- | --- | --- | --- | --- | --- | --- | --- | --- | --- | --- | --- | --- | --- | --- | --- | --- | --- | --- | --- | --- | --- | --- | --- | --- | --- | --- | --- | --- | --- | --- | --- | --- | --- | --- | --- | --- | --- | --- | --- | --- | --- | --- | --- | --- | --- | --- | --- | --- | --- | --- | --- | --- | --- | --- | --- | --- | --- | --- | --- | --- | --- | --- | --- | --- | --- | --- | --- | --- | --- | --- | --- | --- | --- | --- | --- | --- | --- | --- | --- | --- | --- | --- | --- | --- | --- | --- | --- | --- | --- | --- | --- | --- | --- | --- | --- | --- | --- | --- | --- | --- | --- | --- | --- | --- | --- | --- | --- | --- | --- | --- | --- | --- | --- | --- | --- | --- | --- | --- | --- | --- | --- | --- | --- | --- | --- | --- | --- | --- | --- | --- | --- | --- | --- | --- | --- | --- | --- | --- | --- | --- | --- | --- | --- | --- | --- | --- | --- | --- | --- | --- | --- | --- | --- | --- | --- | --- | --- | --- | --- | --- | --- | --- | --- | --- | --- | --- | --- | --- | --- | --- | --- | --- | --- | --- | --- | --- | --- | --- | --- | --- | --- | --- | --- | --- | --- | --- | --- | --- | --- | --- | --- | --- | --- | --- | --- | --- | --- | --- | --- | --- | --- | --- | --- | --- | --- | --- | --- | --- | --- | --- | --- | --- | --- | --- | --- | --- | --- | --- | --- | --- | --- | --- | --- | --- | --- | --- | --- | --- | --- | --- | --- | --- | --- | --- | --- | --- | --- | --- | --- | --- | --- | --- | --- | --- | --- | --- | --- | --- | --- | --- | --- | --- | --- | --- | --- | --- | --- | --- | --- | --- | --- | --- | --- | --- | --- | --- | --- | --- | --- | --- | --- | --- | --- | --- | --- | --- | --- | --- | --- | --- | --- | --- | --- | --- | --- | --- | --- | --- | --- | --- | --- | --- | --- | --- | --- | --- | --- | --- | --- | --- | --- | --- | --- | --- | --- | --- | --- | --- | --- | --- | --- | --- | --- | --- | --- | --- | --- | --- | --- | --- | --- | --- | --- | --- | --- | --- | --- | --- | --- | --- | --- | --- | --- | --- | --- | --- | --- | --- | --- | --- | --- | --- | --- | --- | --- | --- | --- | --- | --- | --- | --- | --- | --- | --- | --- | --- | --- | --- | --- | --- | --- | --- | --- | --- | --- | --- | --- | --- | --- | --- | --- | --- | --- | --- | --- | --- | --- | --- | --- | --- | --- | --- | --- | --- | --- | --- | --- | --- | --- | --- | --- | --- | --- | --- | --- | --- | --- | --- | --- | --- | --- | --- | --- | --- | --- | --- | --- | --- | --- | --- | --- | --- | --- | --- | --- | --- | --- | --- | --- | --- | --- | --- | --- | --- | --- | --- | --- | --- | --- | --- | --- | --- | --- | --- | --- | --- | --- | --- | --- | --- | --- | --- | --- | --- | --- | --- | --- | --- | --- | --- | --- | --- | --- | --- | --- | --- | --- | --- | --- | --- | --- | --- | --- | --- | --- | --- | --- | --- | --- | --- | --- | --- | --- | --- | --- | --- | --- | --- | --- | --- | --- | --- | --- | --- | --- | --- | --- | --- | --- | --- | --- | --- | --- | --- | --- | --- | --- | --- | --- | --- | --- | --- | --- | --- | --- | --- | --- | --- | --- | --- | --- | --- | --- | --- | --- | --- | --- | --- | --- | --- | --- | --- | --- | --- | --- | --- | --- | --- | --- | --- | --- | --- | --- | --- | --- | --- | --- | --- | --- | --- | --- | --- | --- | --- | --- | --- | --- | --- | --- | --- | --- | --- | --- | --- | --- | --- | --- | --- | --- | --- | --- | --- | --- | --- | --- | --- | --- | --- | --- | --- | --- | --- | --- | --- | --- | --- | --- | --- | --- | --- | --- | --- | --- | --- | --- | --- | --- | --- | --- | --- | --- | --- | --- | --- | --- | --- | --- | --- | --- | --- | --- | --- | --- | --- | --- | --- | --- | --- | --- | --- | --- | --- | --- | --- | --- | --- | --- | --- | --- | --- | --- | --- | --- | --- | --- | --- | --- | --- | --- | --- | --- | --- | --- | --- | --- | --- | --- | --- | --- | --- | --- | --- | --- | --- | --- | --- | --- | --- | --- | --- | --- | --- | --- | --- | --- | --- | --- | --- | --- | --- | --- | --- | --- | --- | --- | --- | --- | --- | --- | --- | --- | --- | --- | --- | --- | --- | --- | --- | --- | --- | --- | --- | --- | --- | --- | --- | --- | --- | --- | --- | --- | --- | --- | --- | --- | --- | --- | --- | --- | --- | --- | --- | --- | --- | --- | --- | --- | --- | --- | --- | --- | --- | --- | --- | --- | --- | --- | --- | --- | --- | --- | --- | --- | --- | --- | --- | --- | --- | --- | --- | --- | --- | --- | --- | --- | --- | --- | --- | --- | --- | --- | --- | --- | --- | --- | --- | --- | --- | --- | --- | --- | --- | --- | --- | --- | --- | --- | --- | --- | --- | --- | --- | --- | --- | --- | --- | --- | --- | --- | --- | --- | --- | --- | --- | --- | --- | --- | --- | --- | --- | --- | --- | --- | --- | --- | --- | --- | --- | --- | --- | --- | --- | --- | --- | --- | --- | --- | --- | --- | --- | --- | --- | --- | --- | --- | --- | --- | --- | --- | --- | --- | --- | --- | --- | --- | --- | --- | --- | --- | --- | --- | --- | --- | --- | --- | --- | --- | --- | --- | --- | --- | --- | --- | --- | --- | --- | --- | --- | --- | --- | --- | --- | --- | --- | --- | --- | --- | --- | --- | --- | --- | --- | --- | --- | --- | --- | --- | --- | --- | --- | --- | --- | --- | --- | --- | --- | --- | --- | --- | --- | --- | --- | --- | --- | --- | --- | --- | --- | --- | --- | --- | --- | --- | --- | --- | --- | --- | --- | --- | --- | --- | --- | --- | --- | --- | --- | --- | --- | --- | --- | --- | --- | --- | --- | --- | --- | --- | --- | --- | --- | --- | --- | --- | --- | --- | --- | --- | --- | --- | --- | --- | --- | --- | --- | --- | --- | --- | --- | --- | --- | --- | --- | --- | --- | --- | --- | --- | --- | --- | --- | --- | --- | --- | --- | --- | --- | --- | --- | --- | --- | --- | --- | --- | --- | --- | --- | --- | --- | --- | --- | --- | --- | --- | --- | --- | --- | --- | --- | --- | --- | --- | --- | --- | --- | --- | --- | --- | --- | --- | --- | --- | --- | --- | --- | --- | --- | --- | --- | --- | --- | --- | --- | --- | --- | --- | --- | --- | --- | --- | --- | --- | --- | --- | --- | --- | --- | --- | --- | --- | --- | --- | --- | --- | --- | --- | --- | --- | --- | --- | --- | --- | --- | --- | --- | --- | --- | --- | --- | --- | --- | --- | --- | --- | --- | --- | --- | --- | --- | --- | --- | --- | --- | --- | --- | --- | --- | --- | --- | --- | --- | --- | --- | --- | --- | --- | --- | --- | --- | --- | --- | --- | --- | --- | --- | --- | --- | --- | --- | --- | --- | --- | --- | --- | --- | --- | --- | --- | --- | --- | --- | --- | --- | --- | --- | --- | --- | --- | --- | --- | --- | --- | --- | --- | --- | --- | --- | --- | --- | --- | --- | --- | --- | --- | --- | --- | --- | --- | --- | --- | --- | --- | --- | --- | --- | --- | --- | --- | --- | --- | --- | --- | --- | --- | --- | --- | --- | --- | --- | --- | --- | --- | --- | --- | --- | --- | --- | --- | --- | --- | --- | --- | --- | --- | --- | --- | --- | --- | --- | --- | --- | --- | --- | --- | --- | --- | --- | --- | --- | --- | --- | --- | --- | --- | --- | --- | --- | --- | --- | --- | --- | --- | --- | --- | --- | --- | --- | --- | --- | --- | --- | --- | --- | --- | --- | --- | --- | --- | --- | --- | --- | --- | --- | --- | --- | --- | --- | --- | --- | --- | --- | --- | --- | --- | --- | --- | --- | --- | --- | --- | --- | --- | --- | --- | --- | --- | --- | --- | --- | --- | --- | --- | --- | --- | --- | --- | --- | --- | --- | --- | --- | --- | --- | --- | --- | --- | --- | --- | --- | --- | --- | --- | --- | --- | --- | --- | --- | --- | --- | --- | --- | --- | --- | --- | --- | --- | --- | --- | --- | --- | --- | --- | --- | --- | --- | --- | --- | --- | --- | --- | --- | --- | --- | --- | --- | --- | --- | --- | --- | --- | --- | --- | --- | --- | --- | --- | --- | --- | --- | --- | --- | --- | --- | --- | --- | --- | --- | --- | --- | --- | --- | --- | --- | --- | --- | --- | --- | --- | --- | --- | --- | --- | --- | --- | --- | --- | --- | --- | --- | --- | --- | --- | --- | --- | --- | --- | --- | --- | --- | --- | --- | --- | --- | --- | --- | --- | --- | --- | --- | --- | --- | --- | --- | --- | --- | --- | --- | --- | --- | --- | --- | --- | --- | --- | --- | --- | --- | --- | --- | --- | --- | --- | --- | --- | --- | --- | --- | --- | --- | --- | --- | --- | --- | --- | --- | --- | --- | --- | --- | --- | --- | --- | --- | --- | --- | --- | --- | --- | --- | --- | --- | --- | --- | --- | --- | --- | --- | --- | --- | --- | --- | --- | --- | --- | --- | --- | --- | --- | --- | --- | --- | --- | --- | --- | --- | --- | --- | --- | --- | --- | --- | --- | --- | --- | --- | --- | --- | --- | --- | --- | --- | --- | --- | --- | --- | --- | --- | --- | --- | --- | --- | --- | --- | --- | --- | --- | --- | --- | --- | --- | --- | --- | --- | --- | --- | --- | --- | --- | --- | --- | --- | --- | --- | --- | --- | --- | --- | --- | --- | --- | --- | --- | --- | --- | --- | --- | --- | --- | --- | --- | --- | --- | --- | --- | --- | --- | --- | --- | --- | --- | --- | --- | --- | --- | --- | --- | --- | --- | --- | --- | --- | --- | --- | --- | --- | --- | --- | --- | --- | --- | --- | --- | --- | --- | --- | --- | --- | --- | --- | --- | --- | --- | --- | --- | --- | --- | --- | --- | --- | --- | --- | --- | --- | --- | --- | --- | --- | --- | --- | --- | --- | --- | --- | --- | --- | --- | --- | --- | --- | --- | --- | --- | --- | --- | --- | --- | --- | --- | --- | --- | --- | --- | --- | --- | --- | --- | --- | --- | --- | --- | --- | --- | --- | --- | --- | --- | --- | --- | --- | --- | --- | --- | --- | --- | --- | --- | --- | --- | --- | --- | --- | --- | --- | --- | --- | --- | --- | --- | --- | --- | --- | --- | --- | --- | --- | --- | --- | --- | --- | --- | --- | --- | --- | --- | --- | --- | --- | --- | --- | --- | --- | --- | --- | --- | --- | --- | --- | --- | --- | --- | --- | --- | --- | --- | --- | --- | --- | --- | --- | --- | --- | --- | --- | --- | --- | --- | --- | --- | --- | --- | --- | --- | --- | --- | --- | --- | --- | --- | --- | --- | --- | --- | --- | --- | --- | --- | --- | --- | --- | --- | --- | --- | --- | --- | --- | --- | --- | --- | --- | --- | --- | --- | --- | --- | --- | --- | --- | --- | --- | --- | --- | --- | --- | --- | --- | --- | --- | --- | --- | --- | --- | --- | --- | --- | --- | --- | --- | --- | --- | --- | --- | --- | --- | --- | --- | --- | --- | --- | --- | --- | --- | --- | --- | --- | --- | --- | --- | --- | --- | --- | --- | --- | --- | --- | --- | --- | --- | --- | --- | --- | --- | --- | --- | --- | --- | --- | --- | --- | --- | --- | --- | --- | --- | --- | --- | --- | --- | --- | --- | --- | --- | --- | --- | --- | --- | --- | --- | --- | --- | --- | --- | --- | --- | --- | --- | --- | --- | --- | --- | --- | --- | --- | --- | --- | --- | --- | --- | --- | --- | --- | --- | --- | --- | --- | --- | --- | --- | --- | --- | --- | --- | --- | --- | --- | --- | --- | --- | --- | --- | --- | --- | --- | --- | --- | --- | --- | --- | --- | --- | --- | --- | --- | --- | --- | --- | --- | --- | --- | --- | --- | --- | --- | --- | --- | --- | --- | --- | --- | --- | --- | --- | --- | --- | --- | --- | --- | --- | --- | --- | --- | --- | --- | --- | --- | --- | --- | --- | --- | --- | --- | --- | --- | --- | --- | --- | --- | --- | --- | --- | --- | --- | --- | --- | --- | --- | --- | --- | --- | --- | --- | --- | --- | --- | --- | --- | --- | --- | --- | --- | --- | --- | --- | --- | --- | --- | --- | --- | --- | --- | --- | --- | --- | --- | --- | --- | --- | --- | --- | --- | --- | --- | --- | --- | --- | --- | --- | --- | --- | --- | --- | --- | --- | --- | --- | --- | --- | --- | --- | --- | --- | --- | --- | --- | --- | --- | --- | --- | --- | --- | --- | --- | --- | --- | --- | --- | --- | --- | --- | --- | --- | --- | --- | --- | --- | --- | --- | --- | --- | --- | --- | --- | --- | --- | --- | --- | --- | --- | --- | --- | --- | --- | --- | --- | --- | --- | --- | --- | --- | --- | --- | --- | --- | --- | --- | --- | --- | --- | --- | --- | --- | --- | --- | --- | --- | --- | --- | --- | --- | --- | --- | --- | --- | --- | --- | --- | --- | --- | --- | --- | --- | --- | --- | --- | --- | --- | --- | --- | --- | --- | --- | --- | --- | --- | --- | --- | --- | --- | --- | --- | --- | --- | --- | --- | --- | --- | --- | --- | --- | --- | --- | --- | --- | --- | --- | --- | --- | --- | --- | --- | --- | --- | --- | --- | --- | --- | --- | --- | --- | --- | --- | --- | --- | --- | --- | --- | --- | --- | --- | --- | --- | --- | --- | --- | --- | --- | --- | --- | --- | --- | --- | --- | --- | --- | --- | --- | --- | --- | --- | --- | --- | --- | --- | --- | --- | --- | --- | --- | --- | --- | --- | --- | --- | --- | --- | --- | --- | --- | --- | --- | --- | --- | --- | --- | --- | --- | --- | --- | --- | --- | --- | --- | --- | --- | --- | --- | --- | --- | --- | --- | --- | --- | --- | --- | --- | --- | --- | --- | --- | --- | --- | --- | --- | --- | --- | --- | --- | --- | --- | --- | --- | --- | --- | --- | --- | --- | --- | --- | --- | --- | --- | --- | --- | --- | --- | --- | --- | --- | --- | --- | --- | --- | --- | --- | --- | --- | --- | --- | --- | --- | --- | --- | --- | --- | --- | --- | --- | --- | --- | --- | --- | --- | --- | --- | --- | --- | --- | --- | --- | --- | --- | --- | --- | --- | --- | --- | --- | --- | --- | --- | --- | --- | --- | --- | --- | --- | --- | --- | --- | --- | --- | --- | --- | --- | --- | --- | --- | --- | --- | --- | --- | --- | --- | --- | --- | --- | --- | --- | --- | --- | --- | --- | --- | --- | --- | --- | --- | --- | --- | --- | --- | --- | --- | --- | --- | --- | --- | --- | --- | --- | --- | --- | --- | --- | --- | --- | --- | --- | --- | --- | --- | --- | --- | --- | --- | --- | --- | --- | --- | --- | --- | --- | --- | --- | --- | --- | --- | --- | --- | --- | --- | --- | --- | --- | --- | --- | --- | --- | --- | --- | --- | --- | --- | --- | --- | --- | --- | --- | --- | --- | --- | --- | --- | --- | --- | --- | --- | --- | --- | --- | --- | --- | --- | --- | --- | --- | --- | --- | --- | --- | --- | --- | --- | --- | --- | --- | --- | --- | --- | --- | --- | --- | --- | --- | --- | --- | --- | --- | --- | --- | --- | --- | --- | --- | --- | --- | --- | --- | --- | --- | --- | --- | --- | --- | --- | --- | --- | --- | --- | --- | --- | --- | --- | --- | --- | --- | --- | --- | --- | --- | --- | --- | --- | --- | --- | --- | --- | --- | --- | --- | --- | --- | --- | --- | --- | --- | --- | --- | --- | --- | --- | --- | --- | --- | --- | --- | --- | --- | --- | --- | --- | --- | --- | --- | --- | --- | --- | --- | --- | --- | --- | --- | --- | --- | --- | --- | --- | --- | --- | --- | --- | --- | --- | --- | --- | --- | --- | --- | --- | --- | --- | --- | --- | --- | --- | --- | --- | --- | --- | --- | --- | --- | --- | --- | --- | --- | --- | --- | --- | --- | --- | --- | --- | --- | --- | --- | --- | --- | --- | --- | --- | --- | --- | --- | --- | --- | --- | --- | --- | --- | --- | --- | --- | --- | --- | --- | --- | --- | --- | --- | --- | --- | --- | --- | --- | --- | --- | --- | --- | --- | --- | --- | --- | --- | --- | --- | --- | --- | --- | --- | --- | --- | --- | --- | --- | --- | --- | --- | --- | --- | --- | --- | --- | --- | --- | --- | --- | --- | --- | --- | --- | --- | --- | --- | --- | --- | --- | --- | --- | --- | --- | --- | --- | --- | --- | --- | --- | --- | --- | --- | --- | --- | --- | --- | --- | --- | --- | --- | --- | --- | --- | --- | --- | --- | --- | --- | --- | --- | --- | --- | --- | --- | --- | --- | --- | --- | --- | --- | --- | --- | --- | --- | --- | --- | --- | --- | --- | --- | --- | --- | --- | --- | --- | --- | --- | --- | --- | --- | --- | --- | --- | --- | --- | --- | --- | --- | --- | --- | --- | --- | --- | --- | --- | --- | --- | --- | --- | --- | --- | --- | --- | --- | --- | --- | --- | --- | --- | --- | --- | --- | --- | --- | --- | --- | --- | --- | --- | --- | --- | --- | --- | --- | --- | --- | --- | --- | --- | --- | --- | --- | --- | --- | --- | --- | --- | --- | --- | --- | --- | --- | --- | --- | --- | --- | --- | --- | --- | --- | --- | --- | --- | --- | --- | --- | --- | --- | --- | --- | --- | --- | --- | --- | --- | --- | --- | --- | --- | --- | --- | --- | --- | --- | --- | --- | --- | --- | --- | --- | --- | --- | --- | --- | --- | --- | --- | --- | --- | --- | --- | --- | --- | --- | --- | --- | --- | --- | --- | --- | --- | --- | --- | --- | --- | --- | --- | --- | --- | --- | --- | --- | --- | --- | --- | --- | --- | --- | --- | --- | --- | --- | --- | --- | --- | --- | --- | --- | --- | --- | --- | --- | --- | --- | --- | --- | --- | --- | --- | --- | --- | --- | --- | --- | --- | --- | --- | --- | --- | --- | --- | --- | --- | --- | --- | --- | --- | --- | --- | --- | --- | --- | --- | --- | --- | --- | --- | --- | --- | --- | --- | --- | --- | --- | --- | --- | --- | --- | --- | --- | --- | --- | --- | --- | --- | --- | --- | --- | --- | --- | --- | --- | --- | --- | --- | --- | --- | --- | --- | --- | --- | --- | --- | --- | --- | --- | --- | --- | --- | --- | --- | --- | --- | --- | --- | --- | --- | --- | --- | --- | --- | --- | --- | --- | --- | --- | --- | --- | --- | --- | --- | --- | --- | --- | --- | --- | --- | --- | --- | --- | --- | --- | --- | --- | --- | --- | --- | --- | --- | --- | --- | --- | --- | --- | --- | --- | --- | --- | --- | --- | --- | --- | --- | --- | --- | --- | --- | --- | --- | --- | --- | --- | --- | --- | --- | --- | --- | --- | --- | --- | --- | --- | --- | --- | --- | --- | --- | --- | --- | --- | --- | --- | --- | --- | --- | --- | --- | --- | --- | --- | --- | --- | --- | --- | --- | --- | --- | --- | --- | --- | --- | --- | --- | --- | --- | --- | --- | --- | --- | --- | --- | --- | --- | --- | --- | --- | --- | --- | --- | --- | --- | --- | --- | --- | --- | --- | --- | --- | --- | --- | --- | --- | --- | --- | --- | --- | --- | --- | --- | --- | --- | --- | --- | --- | --- | --- | --- | --- | --- | --- | --- | --- | --- | --- | --- | --- | --- | --- | --- | --- | --- | --- | --- | --- | --- | --- | --- | --- | --- | --- | --- | --- | --- | --- | --- | --- | --- | --- | --- | --- | --- | --- | --- | --- | --- | --- | --- | --- | --- | --- | --- | --- | --- | --- | --- | --- | --- | --- | --- | --- | --- | --- | --- | --- | --- | --- | --- | --- | --- | --- | --- | --- | --- | --- | --- | --- | --- | --- | --- | --- | --- | --- | --- | --- | --- | --- | --- | --- | --- | --- | --- | --- | --- | --- | --- | --- | --- | --- | --- | --- | --- | --- | --- | --- | --- | --- | --- | --- | --- | --- | --- | --- | --- | --- | --- | --- | --- | --- | --- | --- | --- | --- | --- | --- | --- | --- | --- | --- | --- | --- | --- | --- | --- | --- | --- | --- | --- | --- | --- | --- | --- | --- | --- | --- | --- | --- | --- | --- | --- | --- | --- | --- | --- | --- | --- | --- | --- | --- | --- | --- | --- | --- | --- | --- | --- | --- | --- | --- | --- | --- | --- | --- | --- | --- | --- | --- | --- | --- | --- | --- | --- | --- | --- | --- | --- | --- | --- | --- | --- | --- | --- | --- | --- | --- | --- | --- | --- | --- | --- | --- | --- | --- | --- | --- | --- | --- | --- | --- | --- | --- | --- | --- | --- | --- | --- | --- | --- | --- | --- | --- | --- | --- | --- | --- | --- | --- | --- | --- | --- | --- | --- | --- | --- | --- | --- | --- | --- | --- | | DAVID Functional Annotation Clustering      |  | | --- | | Functional Annotation Clustering | | Help and Manual | | Current Gene List: DavidMay18\_TPM85 | | Current Background: DavidMay18\_TPM8\_bg | | 494 DAVID IDs |  - Options          Classification Stringency    Custom   Lowest   Low   Medium   High   Highest   - |  |  |  |     | --- | --- | --- |     | Kappa Similarity | Similarity Term Overlap 3 4 5 6 7 8 9 10 | Similarity Threshold 0.20 0.25 0.30 0.35 0.40 0.45 0.50 0.55 0.60 0.65 0.70 0.75 0.80 0.85 0.90 0.95 1.00 |     | Classification | Initial Group Membership 2 3 4 5 6 7 8 9 10 | Final Group Membership 2 3 4 5 6 7 8 9 10 | Multiple Linkage Threshold 0.00 0.05 0.10 0.15 0.20 0.25 0.30 0.35 0.40 0.45 0.50 0.55 0.60 0.65 0.70 0.75 0.80 0.85 0.90 0.95 1.00 |  |     | Enrichment Thresholds | EASE |  |  |  |     |     | Display | Fold Change | Bonferroni | Benjamini | FDR | LT,PH,PT |  |  |  |  | | --- | --- | --- | |  |  |  |     |  |  | | --- | --- | | 88 Cluster(s) | Download File |  | Annotation Cluster 1 | | Enrichment Score: 7.28 |  |  | Count | P\_Value | Benjamini | | --- | --- | --- | --- | --- | --- | --- | --- | |  | GOTERM\_CC\_5 | cytoplasmic part | **RT** |  | 208 | 4.2E-10 | 2.4E-8 | |  | GOTERM\_CC\_5 | cytoplasm | **RT** |  | 257 | 4.2E-9 | 1.6E-7 | |  | GOTERM\_CC\_5 | intracellular organelle | **RT** |  | 279 | 8.3E-5 | 1.5E-3 | | Annotation Cluster 2 | | Enrichment Score: 7.1 |  |  | Count | P\_Value | Benjamini | | --- | --- | --- | --- | --- | --- | --- | --- | |  | GOTERM\_CC\_5 | mitochondrial membrane part | **RT** |  | 37 | 4.6E-13 | 1.8E-10 | |  | GOTERM\_CC\_5 | inner mitochondrial membrane protein complex | **RT** |  | 33 | 3.5E-12 | 6.9E-10 | |  | GOTERM\_CC\_5 | mitochondrial protein complex | **RT** |  | 35 | 7.2E-12 | 9.4E-10 | |  | GOTERM\_CC\_5 | mitochondrial inner membrane | **RT** |  | 38 | 1.7E-10 | 1.3E-8 | |  | GOTERM\_CC\_5 | organelle inner membrane | **RT** |  | 39 | 3.0E-10 | 2.0E-8 | |  | GOTERM\_BP\_5 | nucleoside monophosphate metabolic process | **RT** |  | 32 | 4.9E-10 | 6.7E-7 | |  | GOTERM\_BP\_5 | nucleoside triphosphate metabolic process | **RT** |  | 31 | 1.4E-9 | 9.2E-7 | |  | GOTERM\_CC\_5 | mitochondrial membrane | **RT** |  | 42 | 1.8E-9 | 7.9E-8 | |  | GOTERM\_BP\_5 | ribonucleoside metabolic process | **RT** |  | 33 | 4.7E-9 | 2.1E-6 | |  | GOTERM\_CC\_5 | mitochondrial envelope | **RT** |  | 42 | 6.0E-9 | 2.1E-7 | |  | GOTERM\_CC\_5 | mitochondrial respiratory chain | **RT** |  | 22 | 8.6E-9 | 2.8E-7 | |  | GOTERM\_BP\_5 | nucleoside metabolic process | **RT** |  | 33 | 1.1E-8 | 3.9E-6 | |  | GOTERM\_BP\_5 | purine nucleoside metabolic process | **RT** |  | 30 | 5.1E-8 | 1.4E-5 | |  | GOTERM\_BP\_5 | respiratory electron transport chain | **RT** |  | 19 | 6.1E-7 | 1.4E-4 | |  | GOTERM\_BP\_5 | ribonucleotide metabolic process | **RT** |  | 33 | 6.4E-7 | 1.2E-4 | |  | GOTERM\_BP\_5 | ribose phosphate metabolic process | **RT** |  | 33 | 7.6E-7 | 1.3E-4 | |  | GOTERM\_CC\_5 | mitochondrion | **RT** |  | 67 | 8.7E-7 | 2.1E-5 | |  | GOTERM\_BP\_5 | ATP synthesis coupled electron transport | **RT** |  | 18 | 9.4E-7 | 1.4E-4 | |  | GOTERM\_CC\_5 | mitochondrial part | **RT** |  | 50 | 2.0E-6 | 4.2E-5 | |  | GOTERM\_BP\_5 | nucleotide metabolic process | **RT** |  | 35 | 2.5E-6 | 3.4E-4 | |  | GOTERM\_BP\_5 | nucleoside phosphate metabolic process | **RT** |  | 35 | 2.9E-6 | 3.6E-4 | |  | GOTERM\_BP\_5 | nucleobase-containing small molecule metabolic process | **RT** |  | 37 | 3.0E-6 | 3.4E-4 | |  | GOTERM\_BP\_5 | purine nucleotide metabolic process | **RT** |  | 31 | 3.8E-6 | 3.7E-4 | |  | GOTERM\_CC\_5 | organelle envelope | **RT** |  | 45 | 5.3E-6 | 9.9E-5 | |  | GOTERM\_BP\_5 | cellular respiration | **RT** |  | 21 | 8.4E-6 | 7.1E-4 | |  | GOTERM\_MF\_5 | cation transmembrane transporter activity | **RT** |  | 22 | 2.7E-2 | 5.2E-1 | |  | GOTERM\_BP\_5 | phosphorylation | **RT** |  | 38 | 3.3E-1 | 1.0E0 | | Annotation Cluster 3 | | Enrichment Score: 6.33 |  |  | Count | P\_Value | Benjamini | | --- | --- | --- | --- | --- | --- | --- | --- | |  | GOTERM\_CC\_5 | cytosolic ribosome | **RT** |  | 32 | 1.1E-10 | 1.1E-8 | |  | GOTERM\_CC\_5 | cytosolic small ribosomal subunit | **RT** |  | 20 | 4.4E-10 | 2.2E-8 | |  | GOTERM\_CC\_5 | ribosomal subunit | **RT** |  | 37 | 3.9E-8 | 1.2E-6 | |  | GOTERM\_CC\_5 | cytosolic part | **RT** |  | 33 | 8.2E-8 | 2.3E-6 | |  | GOTERM\_CC\_5 | ribosome | **RT** |  | 37 | 1.0E-7 | 2.6E-6 | |  | GOTERM\_CC\_5 | cytosol | **RT** |  | 73 | 1.4E-6 | 3.1E-5 | |  | GOTERM\_CC\_5 | small ribosomal subunit | **RT** |  | 20 | 1.9E-6 | 4.1E-5 | |  | GOTERM\_BP\_5 | translation | **RT** |  | 53 | 3.5E-6 | 3.7E-4 | |  | GOTERM\_BP\_5 | peptide biosynthetic process | **RT** |  | 54 | 7.4E-6 | 6.7E-4 | |  | GOTERM\_BP\_5 | amide biosynthetic process | **RT** |  | 54 | 1.3E-5 | 1.0E-3 | |  | GOTERM\_BP\_5 | peptide metabolic process | **RT** |  | 56 | 3.8E-5 | 2.9E-3 | |  | GOTERM\_CC\_5 | intracellular ribonucleoprotein complex | **RT** |  | 59 | 1.9E-4 | 3.1E-3 | | Annotation Cluster 4 | | Enrichment Score: 5.37 |  |  | Count | P\_Value | Benjamini | | --- | --- | --- | --- | --- | --- | --- | --- | |  | GOTERM\_CC\_5 | inner mitochondrial membrane protein complex | **RT** |  | 33 | 3.5E-12 | 6.9E-10 | |  | GOTERM\_CC\_5 | mitochondrial respiratory chain | **RT** |  | 22 | 8.6E-9 | 2.8E-7 | |  | GOTERM\_BP\_5 | respiratory electron transport chain | **RT** |  | 19 | 6.1E-7 | 1.4E-4 | |  | GOTERM\_BP\_5 | ATP synthesis coupled electron transport | **RT** |  | 18 | 9.4E-7 | 1.4E-4 | |  | GOTERM\_CC\_5 | NADH dehydrogenase complex | **RT** |  | 12 | 2.4E-4 | 3.7E-3 | |  | GOTERM\_CC\_5 | respiratory chain complex I | **RT** |  | 12 | 2.4E-4 | 3.7E-3 | |  | GOTERM\_CC\_5 | mitochondrial respiratory chain complex I | **RT** |  | 12 | 2.4E-4 | 3.7E-3 | |  | GOTERM\_MF\_5 | NADH dehydrogenase (quinone) activity | **RT** |  | 9 | 3.2E-4 | 2.6E-2 | |  | GOTERM\_BP\_5 | mitochondrial electron transport, NADH to ubiquinone | **RT** |  | 8 | 6.5E-3 | 2.6E-1 | | Annotation Cluster 5 | | Enrichment Score: 3.79 |  |  | Count | P\_Value | Benjamini | | --- | --- | --- | --- | --- | --- | --- | --- | |  | GOTERM\_CC\_5 | intracellular organelle part | **RT** |  | 194 | 5.1E-6 | 9.9E-5 | |  | GOTERM\_CC\_5 | intracellular organelle | **RT** |  | 279 | 8.3E-5 | 1.5E-3 | |  | GOTERM\_CC\_5 | intracellular membrane-bounded organelle | **RT** |  | 237 | 1.0E-2 | 1.1E-1 | | Annotation Cluster 6 | | Enrichment Score: 3.67 |  |  | Count | P\_Value | Benjamini | | --- | --- | --- | --- | --- | --- | --- | --- | |  | GOTERM\_MF\_5 | cytochrome-c oxidase activity | **RT** |  | 8 | 1.3E-5 | 2.2E-3 | |  | GOTERM\_BP\_5 | mitochondrial electron transport, cytochrome c to oxygen | **RT** |  | 6 | 2.5E-4 | 1.7E-2 | |  | GOTERM\_CC\_5 | respiratory chain complex IV | **RT** |  | 6 | 7.8E-4 | 1.1E-2 | |  | GOTERM\_CC\_5 | mitochondrial respiratory chain complex IV | **RT** |  | 6 | 7.8E-4 | 1.1E-2 | | Annotation Cluster 7 | | Enrichment Score: 2.72 |  |  | Count | P\_Value | Benjamini | | --- | --- | --- | --- | --- | --- | --- | --- | |  | GOTERM\_CC\_5 | proteasome complex | **RT** |  | 13 | 9.0E-5 | 1.5E-3 | |  | GOTERM\_CC\_5 | proteasome regulatory particle, lid subcomplex | **RT** |  | 6 | 7.8E-4 | 1.1E-2 | |  | GOTERM\_CC\_5 | proteasome regulatory particle | **RT** |  | 7 | 4.1E-3 | 5.5E-2 | |  | GOTERM\_CC\_5 | proteasome accessory complex | **RT** |  | 7 | 5.6E-3 | 7.3E-2 | |  | GOTERM\_BP\_5 | proteasomal protein catabolic process | **RT** |  | 19 | 1.5E-2 | 4.9E-1 | | Annotation Cluster 8 | | Enrichment Score: 2.1 |  |  | Count | P\_Value | Benjamini | | --- | --- | --- | --- | --- | --- | --- | --- | |  | GOTERM\_CC\_5 | proteasome complex | **RT** |  | 13 | 9.0E-5 | 1.5E-3 | |  | GOTERM\_MF\_5 | threonine-type peptidase activity | **RT** |  | 5 | 9.5E-3 | 3.2E-1 | |  | GOTERM\_CC\_5 | proteasome core complex | **RT** |  | 5 | 1.7E-2 | 1.7E-1 | |  | GOTERM\_MF\_5 | endopeptidase activity | **RT** |  | 11 | 2.8E-1 | 9.4E-1 | | Annotation Cluster 9 | | Enrichment Score: 1.85 |  |  | Count | P\_Value | Benjamini | | --- | --- | --- | --- | --- | --- | --- | --- | |  | GOTERM\_BP\_5 | centrosome duplication | **RT** |  | 13 | 6.0E-3 | 2.6E-1 | |  | GOTERM\_BP\_5 | centrosome organization | **RT** |  | 16 | 6.2E-3 | 2.6E-1 | |  | GOTERM\_BP\_5 | microtubule organizing center organization | **RT** |  | 16 | 6.2E-3 | 2.6E-1 | |  | GOTERM\_CC\_5 | large ribosomal subunit | **RT** |  | 17 | 6.3E-3 | 7.9E-2 | |  | GOTERM\_CC\_5 | cytosolic large ribosomal subunit | **RT** |  | 12 | 8.7E-3 | 9.9E-2 | |  | GOTERM\_BP\_5 | centrosome cycle | **RT** |  | 13 | 2.5E-2 | 6.3E-1 | |  | GOTERM\_BP\_5 | microtubule cytoskeleton organization | **RT** |  | 20 | 3.3E-1 | 1.0E0 | | Annotation Cluster 10 | | Enrichment Score: 1.73 |  |  | Count | P\_Value | Benjamini | | --- | --- | --- | --- | --- | --- | --- | --- | |  | GOTERM\_BP\_5 | hydrogen transport | **RT** |  | 14 | 6.0E-5 | 4.3E-3 | |  | GOTERM\_BP\_5 | proton transport | **RT** |  | 14 | 6.0E-5 | 4.3E-3 | |  | GOTERM\_BP\_5 | nucleoside monophosphate biosynthetic process | **RT** |  | 10 | 3.7E-4 | 2.4E-2 | |  | GOTERM\_CC\_5 | mitochondrial proton-transporting ATP synthase complex | **RT** |  | 8 | 4.2E-4 | 6.3E-3 | |  | GOTERM\_CC\_5 | proton-transporting ATP synthase complex | **RT** |  | 8 | 4.2E-4 | 6.3E-3 | |  | GOTERM\_BP\_5 | nucleoside triphosphate biosynthetic process | **RT** |  | 9 | 1.1E-3 | 6.4E-2 | |  | GOTERM\_BP\_5 | nucleoside biosynthetic process | **RT** |  | 10 | 2.9E-3 | 1.5E-1 | |  | GOTERM\_BP\_5 | glycosyl compound biosynthetic process | **RT** |  | 10 | 2.9E-3 | 1.5E-1 | |  | GOTERM\_CC\_5 | proton-transporting ATP synthase complex, coupling factor F(o) | **RT** |  | 5 | 7.6E-3 | 8.9E-2 | |  | GOTERM\_CC\_5 | mitochondrial proton-transporting ATP synthase complex, coupling factor F(o) | **RT** |  | 5 | 7.6E-3 | 8.9E-2 | |  | GOTERM\_MF\_5 | P-P-bond-hydrolysis-driven transmembrane transporter activity | **RT** |  | 14 | 8.3E-3 | 3.6E-1 | |  | GOTERM\_MF\_5 | ATPase coupled ion transmembrane transporter activity | **RT** |  | 9 | 1.7E-2 | 4.3E-1 | |  | GOTERM\_CC\_5 | mitochondrial proton-transporting ATP synthase complex, catalytic core F(1) | **RT** |  | 4 | 2.4E-2 | 2.3E-1 | |  | GOTERM\_CC\_5 | proton-transporting ATP synthase complex, catalytic core F(1) | **RT** |  | 4 | 2.4E-2 | 2.3E-1 | |  | GOTERM\_BP\_5 | inorganic ion transmembrane transport | **RT** |  | 14 | 2.4E-2 | 6.3E-1 | |  | GOTERM\_CC\_5 | proton-transporting two-sector ATPase complex, catalytic domain | **RT** |  | 6 | 2.5E-2 | 2.4E-1 | |  | GOTERM\_MF\_5 | cation transmembrane transporter activity | **RT** |  | 22 | 2.7E-2 | 5.2E-1 | |  | GOTERM\_BP\_5 | cation transmembrane transport | **RT** |  | 14 | 2.9E-2 | 6.8E-1 | |  | GOTERM\_MF\_5 | active ion transmembrane transporter activity | **RT** |  | 9 | 3.2E-2 | 5.3E-1 | |  | GOTERM\_BP\_5 | ribose phosphate biosynthetic process | **RT** |  | 11 | 4.3E-2 | 7.8E-1 | |  | GOTERM\_BP\_5 | organophosphate biosynthetic process | **RT** |  | 16 | 4.9E-2 | 8.1E-1 | |  | GOTERM\_BP\_5 | nucleotide biosynthetic process | **RT** |  | 12 | 5.0E-2 | 8.1E-1 | |  | GOTERM\_BP\_5 | nucleoside phosphate biosynthetic process | **RT** |  | 12 | 5.4E-2 | 8.2E-1 | |  | GOTERM\_BP\_5 | ion transmembrane transport | **RT** |  | 14 | 5.6E-2 | 8.3E-1 | |  | GOTERM\_CC\_5 | proton-transporting two-sector ATPase complex, proton-transporting domain | **RT** |  | 5 | 6.3E-2 | 4.7E-1 | |  | GOTERM\_MF\_5 | ATPase activity, coupled to transmembrane movement of substances | **RT** |  | 10 | 7.2E-2 | 6.3E-1 | |  | GOTERM\_BP\_5 | purine-containing compound biosynthetic process | **RT** |  | 9 | 1.8E-1 | 9.9E-1 | |  | GOTERM\_BP\_5 | cation transport | **RT** |  | 18 | 2.7E-1 | 1.0E0 | |  | GOTERM\_BP\_5 | transmembrane transport | **RT** |  | 21 | 5.6E-1 | 1.0E0 | |  | GOTERM\_BP\_5 | ion transport | **RT** |  | 20 | 6.1E-1 | 1.0E0 | |  | GOTERM\_BP\_5 | ATP hydrolysis coupled proton transport | **RT** |  | 3 | 6.2E-1 | 1.0E0 | |  | GOTERM\_BP\_5 | ATP hydrolysis coupled transmembrane transport | **RT** |  | 3 | 6.5E-1 | 1.0E0 | |  | GOTERM\_MF\_5 | pyrophosphatase activity | **RT** |  | 22 | 6.6E-1 | 1.0E0 | | Annotation Cluster 11 | | Enrichment Score: 1.7 |  |  | Count | P\_Value | Benjamini | | --- | --- | --- | --- | --- | --- | --- | --- | |  | GOTERM\_BP\_5 | cellular macromolecular complex assembly | **RT** |  | 39 | 1.6E-3 | 9.2E-2 | |  | GOTERM\_BP\_5 | cellular protein complex assembly | **RT** |  | 19 | 4.9E-2 | 8.2E-1 | |  | GOTERM\_BP\_5 | protein complex assembly | **RT** |  | 25 | 9.9E-2 | 9.3E-1 | | Annotation Cluster 12 | | Enrichment Score: 1.34 |  |  | Count | P\_Value | Benjamini | | --- | --- | --- | --- | --- | --- | --- | --- | |  | GOTERM\_CC\_5 | proteasome complex | **RT** |  | 13 | 9.0E-5 | 1.5E-3 | |  | GOTERM\_BP\_5 | proteasomal protein catabolic process | **RT** |  | 19 | 1.5E-2 | 4.9E-1 | |  | GOTERM\_BP\_5 | protein catabolic process | **RT** |  | 26 | 7.7E-2 | 8.9E-1 | |  | GOTERM\_BP\_5 | modification-dependent macromolecule catabolic process | **RT** |  | 23 | 1.3E-1 | 9.7E-1 | |  | GOTERM\_BP\_5 | proteolysis involved in cellular protein catabolic process | **RT** |  | 24 | 1.3E-1 | 9.6E-1 | |  | GOTERM\_BP\_5 | cellular protein catabolic process | **RT** |  | 24 | 1.3E-1 | 9.6E-1 | |  | GOTERM\_BP\_5 | cellular macromolecule catabolic process | **RT** |  | 26 | 3.0E-1 | 1.0E0 | |  | GOTERM\_BP\_5 | proteolysis | **RT** |  | 35 | 3.1E-1 | 1.0E0 | | Annotation Cluster 13 | | Enrichment Score: 1.03 |  |  | Count | P\_Value | Benjamini | | --- | --- | --- | --- | --- | --- | --- | --- | |  | GOTERM\_BP\_5 | ribosome assembly | **RT** |  | 7 | 4.0E-2 | 7.7E-1 | |  | GOTERM\_BP\_5 | ribosomal small subunit assembly | **RT** |  | 5 | 5.9E-2 | 8.4E-1 | |  | GOTERM\_BP\_5 | ribosomal small subunit biogenesis | **RT** |  | 5 | 3.4E-1 | 1.0E0 | | Annotation Cluster 14 | | Enrichment Score: 0.94 |  |  | Count | P\_Value | Benjamini | | --- | --- | --- | --- | --- | --- | --- | --- | |  | GOTERM\_CC\_5 | integral component of organelle membrane | **RT** |  | 7 | 9.7E-2 | 5.9E-1 | |  | GOTERM\_CC\_5 | intrinsic component of mitochondrial membrane | **RT** |  | 4 | 1.3E-1 | 6.7E-1 | |  | GOTERM\_CC\_5 | integral component of mitochondrial membrane | **RT** |  | 4 | 1.3E-1 | 6.7E-1 | | Annotation Cluster 15 | | Enrichment Score: 0.94 |  |  | Count | P\_Value | Benjamini | | --- | --- | --- | --- | --- | --- | --- | --- | |  | GOTERM\_BP\_5 | cellular protein metabolic process | **RT** |  | 132 | 1.7E-3 | 9.2E-2 | |  | GOTERM\_BP\_5 | cellular protein modification process | **RT** |  | 58 | 9.6E-1 | 1.0E0 | |  | GOTERM\_BP\_5 | protein modification process | **RT** |  | 58 | 9.6E-1 | 1.0E0 | | Annotation Cluster 16 | | Enrichment Score: 0.93 |  |  | Count | P\_Value | Benjamini | | --- | --- | --- | --- | --- | --- | --- | --- | |  | GOTERM\_CC\_5 | Z disc | **RT** |  | 6 | 4.7E-2 | 3.9E-1 | |  | GOTERM\_CC\_5 | I band | **RT** |  | 6 | 4.7E-2 | 3.9E-1 | |  | GOTERM\_CC\_5 | sarcomere | **RT** |  | 6 | 1.2E-1 | 6.5E-1 | |  | GOTERM\_CC\_5 | M band | **RT** |  | 3 | 1.4E-1 | 7.1E-1 | |  | GOTERM\_CC\_5 | contractile fiber part | **RT** |  | 6 | 1.5E-1 | 7.1E-1 | |  | GOTERM\_CC\_5 | myofibril | **RT** |  | 6 | 1.5E-1 | 7.1E-1 | |  | GOTERM\_CC\_5 | contractile fiber | **RT** |  | 6 | 1.6E-1 | 7.5E-1 | |  | GOTERM\_CC\_5 | A band | **RT** |  | 3 | 2.6E-1 | 8.7E-1 | | Annotation Cluster 17 | | Enrichment Score: 0.85 |  |  | Count | P\_Value | Benjamini | | --- | --- | --- | --- | --- | --- | --- | --- | |  | GOTERM\_BP\_5 | maturation of LSU-rRNA | **RT** |  | 4 | 6.9E-2 | 8.7E-1 | |  | GOTERM\_BP\_5 | ribosomal large subunit biogenesis | **RT** |  | 6 | 1.4E-1 | 9.7E-1 | |  | GOTERM\_BP\_5 | rRNA processing | **RT** |  | 7 | 2.9E-1 | 1.0E0 | | Annotation Cluster 18 | | Enrichment Score: 0.67 |  |  | Count | P\_Value | Benjamini | | --- | --- | --- | --- | --- | --- | --- | --- | |  | GOTERM\_CC\_5 | endoplasmic reticulum membrane | **RT** |  | 17 | 1.6E-1 | 7.4E-1 | |  | GOTERM\_CC\_5 | endoplasmic reticulum part | **RT** |  | 18 | 2.1E-1 | 8.2E-1 | |  | GOTERM\_CC\_5 | endoplasmic reticulum | **RT** |  | 26 | 2.9E-1 | 8.9E-1 | | Annotation Cluster 19 | | Enrichment Score: 0.64 |  |  | Count | P\_Value | Benjamini | | --- | --- | --- | --- | --- | --- | --- | --- | |  | GOTERM\_CC\_5 | peroxisomal membrane | **RT** |  | 3 | 1.8E-1 | 7.7E-1 | |  | GOTERM\_CC\_5 | microbody membrane | **RT** |  | 3 | 1.8E-1 | 7.7E-1 | |  | GOTERM\_CC\_5 | peroxisomal part | **RT** |  | 3 | 1.8E-1 | 7.7E-1 | |  | GOTERM\_CC\_5 | microbody part | **RT** |  | 3 | 1.8E-1 | 7.7E-1 | |  | GOTERM\_CC\_5 | peroxisome | **RT** |  | 5 | 3.6E-1 | 9.3E-1 | |  | GOTERM\_CC\_5 | microbody | **RT** |  | 5 | 3.6E-1 | 9.3E-1 | | Annotation Cluster 20 | | Enrichment Score: 0.64 |  |  | Count | P\_Value | Benjamini | | --- | --- | --- | --- | --- | --- | --- | --- | |  | GOTERM\_BP\_5 | carboxylic acid biosynthetic process | **RT** |  | 7 | 6.2E-2 | 8.4E-1 | |  | GOTERM\_BP\_5 | cellular amino acid biosynthetic process | **RT** |  | 4 | 8.6E-2 | 9.1E-1 | |  | GOTERM\_BP\_5 | organic acid biosynthetic process | **RT** |  | 7 | 8.8E-2 | 9.1E-1 | |  | GOTERM\_BP\_5 | alpha-amino acid biosynthetic process | **RT** |  | 4 | 1.7E-1 | 9.9E-1 | |  | GOTERM\_BP\_5 | carboxylic acid metabolic process | **RT** |  | 14 | 3.3E-1 | 1.0E0 | |  | GOTERM\_BP\_5 | oxoacid metabolic process | **RT** |  | 14 | 3.3E-1 | 1.0E0 | |  | GOTERM\_BP\_5 | glutamine family amino acid metabolic process | **RT** |  | 3 | 4.0E-1 | 1.0E0 | |  | GOTERM\_BP\_5 | alpha-amino acid metabolic process | **RT** |  | 5 | 4.1E-1 | 1.0E0 | |  | GOTERM\_BP\_5 | fatty acid biosynthetic process | **RT** |  | 3 | 4.3E-1 | 1.0E0 | |  | GOTERM\_BP\_5 | fatty acid metabolic process | **RT** |  | 3 | 7.7E-1 | 1.0E0 | | Annotation Cluster 21 | | Enrichment Score: 0.62 |  |  | Count | P\_Value | Benjamini | | --- | --- | --- | --- | --- | --- | --- | --- | |  | GOTERM\_BP\_5 | Notch receptor processing | **RT** |  | 5 | 1.8E-2 | 5.3E-1 | |  | GOTERM\_CC\_5 | gamma-secretase complex | **RT** |  | 3 | 7.8E-2 | 5.4E-1 | |  | GOTERM\_CC\_5 | recycling endosome | **RT** |  | 5 | 2.5E-1 | 8.7E-1 | |  | GOTERM\_CC\_5 | late endosome | **RT** |  | 5 | 6.9E-1 | 9.9E-1 | |  | GOTERM\_CC\_5 | endosome | **RT** |  | 10 | 8.7E-1 | 1.0E0 | |  | GOTERM\_BP\_5 | regulation of Notch signaling pathway | **RT** |  | 5 | 9.2E-1 | 1.0E0 | | Annotation Cluster 22 | | Enrichment Score: 0.61 |  |  | Count | P\_Value | Benjamini | | --- | --- | --- | --- | --- | --- | --- | --- | |  | GOTERM\_CC\_5 | nuclear DNA-directed RNA polymerase complex | **RT** |  | 10 | 1.3E-1 | 6.7E-1 | |  | GOTERM\_CC\_5 | DNA-directed RNA polymerase complex | **RT** |  | 10 | 1.3E-1 | 6.7E-1 | |  | GOTERM\_CC\_5 | RNA polymerase complex | **RT** |  | 10 | 1.3E-1 | 6.7E-1 | |  | GOTERM\_CC\_5 | DNA-directed RNA polymerase II, holoenzyme | **RT** |  | 8 | 2.7E-1 | 8.8E-1 | |  | GOTERM\_CC\_5 | transcription factor complex | **RT** |  | 10 | 2.8E-1 | 8.8E-1 | |  | GOTERM\_CC\_5 | RNA polymerase II transcription factor complex | **RT** |  | 7 | 3.1E-1 | 9.0E-1 | |  | GOTERM\_CC\_5 | nucleoplasm part | **RT** |  | 23 | 3.2E-1 | 9.0E-1 | |  | GOTERM\_CC\_5 | transferase complex, transferring phosphorus-containing groups | **RT** |  | 11 | 3.3E-1 | 9.1E-1 | |  | GOTERM\_CC\_5 | nuclear transcription factor complex | **RT** |  | 7 | 3.8E-1 | 9.3E-1 | |  | GOTERM\_CC\_5 | transcription factor TFIID complex | **RT** |  | 3 | 4.0E-1 | 9.4E-1 | | Annotation Cluster 23 | | Enrichment Score: 0.56 |  |  | Count | P\_Value | Benjamini | | --- | --- | --- | --- | --- | --- | --- | --- | |  | GOTERM\_MF\_5 | translation factor activity, RNA binding | **RT** |  | 9 | 6.7E-2 | 6.4E-1 | |  | GOTERM\_BP\_5 | formation of translation preinitiation complex | **RT** |  | 4 | 1.9E-1 | 9.9E-1 | |  | GOTERM\_CC\_5 | eukaryotic translation initiation factor 3 complex | **RT** |  | 3 | 3.7E-1 | 9.3E-1 | |  | GOTERM\_CC\_5 | eukaryotic 48S preinitiation complex | **RT** |  | 3 | 3.7E-1 | 9.3E-1 | |  | GOTERM\_CC\_5 | eukaryotic 43S preinitiation complex | **RT** |  | 3 | 4.7E-1 | 9.6E-1 | |  | GOTERM\_CC\_5 | translation preinitiation complex | **RT** |  | 3 | 5.1E-1 | 9.7E-1 | | Annotation Cluster 24 | | Enrichment Score: 0.48 |  |  | Count | P\_Value | Benjamini | | --- | --- | --- | --- | --- | --- | --- | --- | |  | GOTERM\_BP\_5 | DNA replication | **RT** |  | 5 | 3.0E-1 | 1.0E0 | |  | GOTERM\_BP\_5 | DNA endoreduplication | **RT** |  | 3 | 3.3E-1 | 1.0E0 | |  | GOTERM\_BP\_5 | cell cycle DNA replication | **RT** |  | 3 | 3.6E-1 | 1.0E0 | | Annotation Cluster 25 | | Enrichment Score: 0.43 |  |  | Count | P\_Value | Benjamini | | --- | --- | --- | --- | --- | --- | --- | --- | |  | GOTERM\_BP\_5 | chromatin assembly | **RT** |  | 5 | 2.0E-1 | 9.9E-1 | |  | GOTERM\_BP\_5 | chromatin assembly or disassembly | **RT** |  | 5 | 2.2E-1 | 9.9E-1 | |  | GOTERM\_BP\_5 | DNA conformation change | **RT** |  | 8 | 2.6E-1 | 1.0E0 | |  | GOTERM\_BP\_5 | nucleosome assembly | **RT** |  | 3 | 3.6E-1 | 1.0E0 | |  | GOTERM\_BP\_5 | protein-DNA complex assembly | **RT** |  | 5 | 4.1E-1 | 1.0E0 | |  | GOTERM\_BP\_5 | nucleosome organization | **RT** |  | 3 | 7.3E-1 | 1.0E0 | |  | GOTERM\_BP\_5 | chromatin remodeling | **RT** |  | 5 | 8.1E-1 | 1.0E0 | | Annotation Cluster 26 | | Enrichment Score: 0.42 |  |  | Count | P\_Value | Benjamini | | --- | --- | --- | --- | --- | --- | --- | --- | |  | GOTERM\_BP\_5 | RNA processing | **RT** |  | 29 | 2.1E-1 | 9.9E-1 | |  | GOTERM\_CC\_5 | spliceosomal complex | **RT** |  | 15 | 2.5E-1 | 8.7E-1 | |  | GOTERM\_CC\_5 | catalytic step 2 spliceosome | **RT** |  | 10 | 4.1E-1 | 9.4E-1 | |  | GOTERM\_CC\_5 | precatalytic spliceosome | **RT** |  | 11 | 5.1E-1 | 9.7E-1 | |  | GOTERM\_BP\_5 | mRNA metabolic process | **RT** |  | 18 | 7.1E-1 | 1.0E0 | | Annotation Cluster 27 | | Enrichment Score: 0.42 |  |  | Count | P\_Value | Benjamini | | --- | --- | --- | --- | --- | --- | --- | --- | |  | GOTERM\_BP\_5 | cellular macromolecule biosynthetic process | **RT** |  | 115 | 4.0E-3 | 1.9E-1 | |  | GOTERM\_CC\_5 | nuclear lumen | **RT** |  | 51 | 7.9E-2 | 5.4E-1 | |  | GOTERM\_CC\_5 | intracellular organelle lumen | **RT** |  | 62 | 8.5E-2 | 5.6E-1 | |  | GOTERM\_CC\_5 | nuclear part | **RT** |  | 67 | 2.6E-1 | 8.7E-1 | |  | GOTERM\_CC\_5 | nucleoplasm | **RT** |  | 32 | 3.0E-1 | 9.0E-1 | |  | GOTERM\_CC\_5 | nucleoplasm part | **RT** |  | 23 | 3.2E-1 | 9.0E-1 | |  | GOTERM\_BP\_5 | nucleobase-containing compound biosynthetic process | **RT** |  | 68 | 3.3E-1 | 1.0E0 | |  | GOTERM\_BP\_5 | nucleic acid metabolic process | **RT** |  | 88 | 5.6E-1 | 1.0E0 | |  | GOTERM\_BP\_5 | RNA metabolic process | **RT** |  | 80 | 6.4E-1 | 1.0E0 | |  | GOTERM\_BP\_5 | RNA biosynthetic process | **RT** |  | 52 | 7.5E-1 | 1.0E0 | |  | GOTERM\_BP\_5 | transcription, DNA-templated | **RT** |  | 45 | 8.0E-1 | 1.0E0 | |  | GOTERM\_CC\_5 | nucleus | **RT** |  | 118 | 8.8E-1 | 1.0E0 | |  | GOTERM\_BP\_5 | regulation of cellular macromolecule biosynthetic process | **RT** |  | 53 | 9.1E-1 | 1.0E0 | |  | GOTERM\_BP\_5 | regulation of macromolecule biosynthetic process | **RT** |  | 53 | 9.2E-1 | 1.0E0 | |  | GOTERM\_BP\_5 | regulation of transcription, DNA-templated | **RT** |  | 44 | 9.3E-1 | 1.0E0 | |  | GOTERM\_BP\_5 | regulation of RNA biosynthetic process | **RT** |  | 44 | 9.3E-1 | 1.0E0 | |  | GOTERM\_BP\_5 | regulation of RNA metabolic process | **RT** |  | 48 | 9.4E-1 | 1.0E0 | |  | GOTERM\_BP\_5 | regulation of gene expression | **RT** |  | 58 | 9.5E-1 | 1.0E0 | | Annotation Cluster 28 | | Enrichment Score: 0.41 |  |  | Count | P\_Value | Benjamini | | --- | --- | --- | --- | --- | --- | --- | --- | |  | GOTERM\_BP\_5 | mitophagy | **RT** |  | 5 | 3.9E-2 | 7.7E-1 | |  | GOTERM\_BP\_5 | mitochondrion disassembly | **RT** |  | 5 | 3.9E-2 | 7.7E-1 | |  | GOTERM\_BP\_5 | organelle disassembly | **RT** |  | 5 | 5.9E-2 | 8.4E-1 | |  | GOTERM\_BP\_5 | larval midgut cell programmed cell death | **RT** |  | 5 | 8.4E-2 | 9.1E-1 | |  | GOTERM\_BP\_5 | larval midgut histolysis | **RT** |  | 5 | 1.3E-1 | 9.7E-1 | |  | GOTERM\_BP\_5 | autophagosome assembly | **RT** |  | 4 | 2.2E-1 | 9.9E-1 | |  | GOTERM\_BP\_5 | autophagosome organization | **RT** |  | 4 | 2.2E-1 | 9.9E-1 | |  | GOTERM\_BP\_5 | programmed cell death involved in cell development | **RT** |  | 11 | 3.2E-1 | 1.0E0 | |  | GOTERM\_BP\_5 | autophagic cell death | **RT** |  | 7 | 4.2E-1 | 1.0E0 | |  | GOTERM\_BP\_5 | salivary gland cell autophagic cell death | **RT** |  | 7 | 4.2E-1 | 1.0E0 | |  | GOTERM\_BP\_5 | salivary gland histolysis | **RT** |  | 7 | 4.4E-1 | 1.0E0 | |  | GOTERM\_BP\_5 | cellular response to starvation | **RT** |  | 6 | 5.2E-1 | 1.0E0 | |  | GOTERM\_BP\_5 | cellular response to nutrient levels | **RT** |  | 6 | 5.4E-1 | 1.0E0 | |  | GOTERM\_BP\_5 | gland morphogenesis | **RT** |  | 9 | 6.2E-1 | 1.0E0 | |  | GOTERM\_BP\_5 | salivary gland morphogenesis | **RT** |  | 9 | 6.2E-1 | 1.0E0 | |  | GOTERM\_BP\_5 | salivary gland development | **RT** |  | 10 | 7.1E-1 | 1.0E0 | |  | GOTERM\_BP\_5 | exocrine system development | **RT** |  | 10 | 7.1E-1 | 1.0E0 | |  | GOTERM\_BP\_5 | gland development | **RT** |  | 11 | 8.3E-1 | 1.0E0 | |  | GOTERM\_BP\_5 | response to starvation | **RT** |  | 6 | 9.0E-1 | 1.0E0 | |  | GOTERM\_BP\_5 | instar larval or pupal morphogenesis | **RT** |  | 24 | 9.7E-1 | 1.0E0 | |  | GOTERM\_BP\_5 | post-embryonic morphogenesis | **RT** |  | 24 | 9.8E-1 | 1.0E0 | |  | GOTERM\_BP\_5 | imaginal disc development | **RT** |  | 25 | 9.9E-1 | 1.0E0 | |  | GOTERM\_BP\_5 | instar larval or pupal development | **RT** |  | 24 | 1.0E0 | 1.0E0 | |  | GOTERM\_BP\_5 | animal organ development | **RT** |  | 49 | 1.0E0 | 1.0E0 | |  | GOTERM\_BP\_5 | organ morphogenesis | **RT** |  | 25 | 1.0E0 | 1.0E0 | | Annotation Cluster 29 | | Enrichment Score: 0.41 |  |  | Count | P\_Value | Benjamini | | --- | --- | --- | --- | --- | --- | --- | --- | |  | GOTERM\_BP\_5 | cell-cell signaling involved in cell fate commitment | **RT** |  | 14 | 2.5E-1 | 1.0E0 | |  | GOTERM\_BP\_5 | lateral inhibition | **RT** |  | 14 | 2.5E-1 | 1.0E0 | |  | GOTERM\_BP\_5 | cell fate commitment | **RT** |  | 18 | 9.4E-1 | 1.0E0 | | Annotation Cluster 30 | | Enrichment Score: 0.38 |  |  | Count | P\_Value | Benjamini | | --- | --- | --- | --- | --- | --- | --- | --- | |  | GOTERM\_MF\_5 | P-P-bond-hydrolysis-driven protein transmembrane transporter activity | **RT** |  | 5 | 6.6E-2 | 6.7E-1 | |  | GOTERM\_MF\_5 | protein transmembrane transporter activity | **RT** |  | 5 | 1.0E-1 | 7.4E-1 | |  | GOTERM\_BP\_5 | intracellular protein transmembrane transport | **RT** |  | 5 | 2.0E-1 | 9.9E-1 | |  | GOTERM\_BP\_5 | protein transmembrane transport | **RT** |  | 5 | 2.2E-1 | 9.9E-1 | |  | GOTERM\_BP\_5 | mitochondrial transmembrane transport | **RT** |  | 4 | 3.4E-1 | 1.0E0 | |  | GOTERM\_BP\_5 | intracellular protein transmembrane import | **RT** |  | 4 | 3.4E-1 | 1.0E0 | |  | GOTERM\_BP\_5 | intracellular protein transport | **RT** |  | 20 | 4.7E-1 | 1.0E0 | |  | GOTERM\_BP\_5 | establishment of protein localization to mitochondrion | **RT** |  | 4 | 5.4E-1 | 1.0E0 | |  | GOTERM\_BP\_5 | protein targeting to mitochondrion | **RT** |  | 4 | 5.4E-1 | 1.0E0 | |  | GOTERM\_BP\_5 | protein transport | **RT** |  | 26 | 5.7E-1 | 1.0E0 | |  | GOTERM\_BP\_5 | protein targeting | **RT** |  | 11 | 5.8E-1 | 1.0E0 | |  | GOTERM\_BP\_5 | establishment of protein localization to organelle | **RT** |  | 9 | 6.5E-1 | 1.0E0 | |  | GOTERM\_BP\_5 | protein import | **RT** |  | 6 | 6.7E-1 | 1.0E0 | |  | GOTERM\_BP\_5 | protein localization to organelle | **RT** |  | 10 | 8.0E-1 | 1.0E0 | |  | GOTERM\_BP\_5 | single-organism intracellular transport | **RT** |  | 10 | 8.0E-1 | 1.0E0 | |  | GOTERM\_BP\_5 | establishment of protein localization to membrane | **RT** |  | 3 | 8.9E-1 | 1.0E0 | |  | GOTERM\_BP\_5 | protein localization to membrane | **RT** |  | 3 | 9.7E-1 | 1.0E0 | | Annotation Cluster 31 | | Enrichment Score: 0.35 |  |  | Count | P\_Value | Benjamini | | --- | --- | --- | --- | --- | --- | --- | --- | |  | GOTERM\_BP\_5 | mitotic nuclear division | **RT** |  | 15 | 2.6E-1 | 1.0E0 | |  | GOTERM\_BP\_5 | nuclear division | **RT** |  | 19 | 3.9E-1 | 1.0E0 | |  | GOTERM\_BP\_5 | mitotic cell cycle process | **RT** |  | 19 | 8.7E-1 | 1.0E0 | | Annotation Cluster 32 | | Enrichment Score: 0.35 |  |  | Count | P\_Value | Benjamini | | --- | --- | --- | --- | --- | --- | --- | --- | |  | GOTERM\_CC\_5 | nuclear chromosome | **RT** |  | 15 | 1.1E-1 | 6.4E-1 | |  | GOTERM\_CC\_5 | nuclear chromatin | **RT** |  | 8 | 3.9E-1 | 9.4E-1 | |  | GOTERM\_CC\_5 | chromatin | **RT** |  | 13 | 5.3E-1 | 9.7E-1 | |  | GOTERM\_CC\_5 | nuclear chromosome part | **RT** |  | 8 | 6.4E-1 | 9.9E-1 | |  | GOTERM\_CC\_5 | chromosome | **RT** |  | 27 | 6.8E-1 | 9.9E-1 | |  | GOTERM\_CC\_5 | chromosomal part | **RT** |  | 19 | 7.8E-1 | 1.0E0 | | Annotation Cluster 33 | | Enrichment Score: 0.33 |  |  | Count | P\_Value | Benjamini | | --- | --- | --- | --- | --- | --- | --- | --- | |  | GOTERM\_BP\_5 | macromolecule methylation | **RT** |  | 8 | 1.8E-1 | 9.9E-1 | |  | GOTERM\_BP\_5 | peptidyl-lysine methylation | **RT** |  | 5 | 3.2E-1 | 1.0E0 | |  | GOTERM\_BP\_5 | protein methylation | **RT** |  | 6 | 3.2E-1 | 1.0E0 | |  | GOTERM\_BP\_5 | histone methylation | **RT** |  | 5 | 4.1E-1 | 1.0E0 | |  | GOTERM\_BP\_5 | covalent chromatin modification | **RT** |  | 10 | 8.3E-1 | 1.0E0 | |  | GOTERM\_BP\_5 | negative regulation of gene expression, epigenetic | **RT** |  | 4 | 9.0E-1 | 1.0E0 | |  | GOTERM\_BP\_5 | chromatin silencing | **RT** |  | 4 | 9.0E-1 | 1.0E0 | | Annotation Cluster 34 | | Enrichment Score: 0.28 |  |  | Count | P\_Value | Benjamini | | --- | --- | --- | --- | --- | --- | --- | --- | |  | GOTERM\_BP\_5 | regulation of antimicrobial humoral response | **RT** |  | 4 | 3.7E-1 | 1.0E0 | |  | GOTERM\_BP\_5 | antimicrobial peptide production | **RT** |  | 3 | 5.7E-1 | 1.0E0 | |  | GOTERM\_BP\_5 | regulation of antimicrobial peptide production | **RT** |  | 3 | 5.7E-1 | 1.0E0 | |  | GOTERM\_BP\_5 | antimicrobial humoral response | **RT** |  | 5 | 6.0E-1 | 1.0E0 | | Annotation Cluster 35 | | Enrichment Score: 0.27 |  |  | Count | P\_Value | Benjamini | | --- | --- | --- | --- | --- | --- | --- | --- | |  | GOTERM\_BP\_5 | glycerophospholipid metabolic process | **RT** |  | 6 | 3.8E-1 | 1.0E0 | |  | GOTERM\_BP\_5 | glycerolipid metabolic process | **RT** |  | 7 | 3.9E-1 | 1.0E0 | |  | GOTERM\_BP\_5 | glycerophospholipid biosynthetic process | **RT** |  | 3 | 4.9E-1 | 1.0E0 | |  | GOTERM\_BP\_5 | glycerolipid biosynthetic process | **RT** |  | 3 | 6.2E-1 | 1.0E0 | |  | GOTERM\_BP\_5 | phospholipid metabolic process | **RT** |  | 6 | 6.9E-1 | 1.0E0 | |  | GOTERM\_BP\_5 | phospholipid biosynthetic process | **RT** |  | 3 | 7.3E-1 | 1.0E0 | | Annotation Cluster 36 | | Enrichment Score: 0.27 |  |  | Count | P\_Value | Benjamini | | --- | --- | --- | --- | --- | --- | --- | --- | |  | GOTERM\_BP\_5 | eye pigment biosynthetic process | **RT** |  | 3 | 5.2E-1 | 1.0E0 | |  | GOTERM\_BP\_5 | pigment metabolic process involved in developmental pigmentation | **RT** |  | 3 | 5.5E-1 | 1.0E0 | |  | GOTERM\_BP\_5 | eye pigment metabolic process | **RT** |  | 3 | 5.5E-1 | 1.0E0 | | Annotation Cluster 37 | | Enrichment Score: 0.26 |  |  | Count | P\_Value | Benjamini | | --- | --- | --- | --- | --- | --- | --- | --- | |  | GOTERM\_CC\_5 | mitochondrial large ribosomal subunit | **RT** |  | 5 | 4.1E-1 | 9.4E-1 | |  | GOTERM\_CC\_5 | organellar large ribosomal subunit | **RT** |  | 5 | 4.1E-1 | 9.4E-1 | |  | GOTERM\_BP\_5 | mitochondrial translation | **RT** |  | 7 | 5.0E-1 | 1.0E0 | |  | GOTERM\_CC\_5 | mitochondrial ribosome | **RT** |  | 6 | 6.7E-1 | 9.9E-1 | |  | GOTERM\_CC\_5 | organellar ribosome | **RT** |  | 6 | 6.7E-1 | 9.9E-1 | |  | GOTERM\_CC\_5 | mitochondrial matrix | **RT** |  | 9 | 7.9E-1 | 1.0E0 | | Annotation Cluster 38 | | Enrichment Score: 0.21 |  |  | Count | P\_Value | Benjamini | | --- | --- | --- | --- | --- | --- | --- | --- | |  | GOTERM\_BP\_5 | regulation of filopodium assembly | **RT** |  | 3 | 5.5E-1 | 1.0E0 | |  | GOTERM\_BP\_5 | filopodium assembly | **RT** |  | 3 | 5.7E-1 | 1.0E0 | |  | GOTERM\_BP\_5 | regulation of cell projection assembly | **RT** |  | 3 | 7.3E-1 | 1.0E0 | | Annotation Cluster 39 | | Enrichment Score: 0.2 |  |  | Count | P\_Value | Benjamini | | --- | --- | --- | --- | --- | --- | --- | --- | |  | GOTERM\_BP\_5 | positive regulation of defense response | **RT** |  | 5 | 5.1E-1 | 1.0E0 | |  | GOTERM\_BP\_5 | positive regulation of innate immune response | **RT** |  | 4 | 5.6E-1 | 1.0E0 | |  | GOTERM\_BP\_5 | regulation of innate immune response | **RT** |  | 4 | 8.8E-1 | 1.0E0 | | Annotation Cluster 40 | | Enrichment Score: 0.2 |  |  | Count | P\_Value | Benjamini | | --- | --- | --- | --- | --- | --- | --- | --- | |  | GOTERM\_BP\_5 | dsRNA transport | **RT** |  | 3 | 4.9E-1 | 1.0E0 | |  | GOTERM\_BP\_5 | RNA transport | **RT** |  | 6 | 5.9E-1 | 1.0E0 | |  | GOTERM\_BP\_5 | nucleobase-containing compound transport | **RT** |  | 6 | 7.4E-1 | 1.0E0 | |  | GOTERM\_BP\_5 | mRNA transport | **RT** |  | 3 | 7.5E-1 | 1.0E0 | | Annotation Cluster 41 | | Enrichment Score: 0.19 |  |  | Count | P\_Value | Benjamini | | --- | --- | --- | --- | --- | --- | --- | --- | |  | GOTERM\_BP\_5 | regulation of cellular protein catabolic process | **RT** |  | 3 | 6.2E-1 | 1.0E0 | |  | GOTERM\_BP\_5 | regulation of protein catabolic process | **RT** |  | 4 | 6.4E-1 | 1.0E0 | |  | GOTERM\_BP\_5 | regulation of proteolysis | **RT** |  | 6 | 6.9E-1 | 1.0E0 | | Annotation Cluster 42 | | Enrichment Score: 0.18 |  |  | Count | P\_Value | Benjamini | | --- | --- | --- | --- | --- | --- | --- | --- | |  | GOTERM\_BP\_5 | positive regulation of chromosome organization | **RT** |  | 4 | 4.4E-1 | 1.0E0 | |  | GOTERM\_BP\_5 | regulation of chromosome organization | **RT** |  | 7 | 6.5E-1 | 1.0E0 | |  | GOTERM\_BP\_5 | positive regulation of organelle organization | **RT** |  | 7 | 6.5E-1 | 1.0E0 | |  | GOTERM\_BP\_5 | positive regulation of protein modification process | **RT** |  | 5 | 9.9E-1 | 1.0E0 | | Annotation Cluster 43 | | Enrichment Score: 0.17 |  |  | Count | P\_Value | Benjamini | | --- | --- | --- | --- | --- | --- | --- | --- | |  | GOTERM\_BP\_5 | negative regulation of MAPK cascade | **RT** |  | 4 | 3.2E-1 | 1.0E0 | |  | GOTERM\_BP\_5 | negative regulation of protein modification process | **RT** |  | 8 | 3.2E-1 | 1.0E0 | |  | GOTERM\_BP\_5 | negative regulation of stress-activated protein kinase signaling cascade | **RT** |  | 3 | 4.6E-1 | 1.0E0 | |  | GOTERM\_BP\_5 | negative regulation of phosphorus metabolic process | **RT** |  | 6 | 5.7E-1 | 1.0E0 | |  | GOTERM\_BP\_5 | negative regulation of phosphate metabolic process | **RT** |  | 6 | 5.7E-1 | 1.0E0 | |  | GOTERM\_BP\_5 | negative regulation of intracellular signal transduction | **RT** |  | 7 | 5.9E-1 | 1.0E0 | |  | GOTERM\_BP\_5 | negative regulation of protein metabolic process | **RT** |  | 12 | 6.4E-1 | 1.0E0 | |  | GOTERM\_BP\_5 | stress-activated MAPK cascade | **RT** |  | 5 | 7.1E-1 | 1.0E0 | |  | GOTERM\_BP\_5 | stress-activated protein kinase signaling cascade | **RT** |  | 5 | 7.4E-1 | 1.0E0 | |  | GOTERM\_BP\_5 | negative regulation of cellular protein metabolic process | **RT** |  | 11 | 7.4E-1 | 1.0E0 | |  | GOTERM\_BP\_5 | regulation of stress-activated protein kinase signaling cascade | **RT** |  | 3 | 8.8E-1 | 1.0E0 | |  | GOTERM\_BP\_5 | regulation of stress-activated MAPK cascade | **RT** |  | 3 | 8.8E-1 | 1.0E0 | |  | GOTERM\_BP\_5 | regulation of protein modification process | **RT** |  | 14 | 8.8E-1 | 1.0E0 | |  | GOTERM\_BP\_5 | MAPK cascade | **RT** |  | 7 | 9.1E-1 | 1.0E0 | |  | GOTERM\_BP\_5 | signal transduction by protein phosphorylation | **RT** |  | 7 | 9.1E-1 | 1.0E0 | |  | GOTERM\_BP\_5 | regulation of MAPK cascade | **RT** |  | 5 | 9.6E-1 | 1.0E0 | |  | GOTERM\_BP\_5 | regulation of phosphate metabolic process | **RT** |  | 11 | 9.8E-1 | 1.0E0 | | Annotation Cluster 44 | | Enrichment Score: 0.17 |  |  | Count | P\_Value | Benjamini | | --- | --- | --- | --- | --- | --- | --- | --- | |  | GOTERM\_BP\_5 | negative regulation of neural precursor cell proliferation | **RT** |  | 3 | 4.9E-1 | 1.0E0 | |  | GOTERM\_BP\_5 | negative regulation of neuroblast proliferation | **RT** |  | 3 | 4.9E-1 | 1.0E0 | |  | GOTERM\_BP\_5 | negative regulation of stem cell proliferation | **RT** |  | 3 | 5.2E-1 | 1.0E0 | |  | GOTERM\_BP\_5 | regulation of neuroblast proliferation | **RT** |  | 3 | 6.0E-1 | 1.0E0 | |  | GOTERM\_BP\_5 | negative regulation of nervous system development | **RT** |  | 7 | 7.9E-1 | 1.0E0 | |  | GOTERM\_BP\_5 | negative regulation of neurogenesis | **RT** |  | 4 | 8.4E-1 | 1.0E0 | |  | GOTERM\_BP\_5 | negative regulation of cell development | **RT** |  | 4 | 9.0E-1 | 1.0E0 | |  | GOTERM\_BP\_5 | regulation of neurogenesis | **RT** |  | 6 | 1.0E0 | 1.0E0 | | Annotation Cluster 45 | | Enrichment Score: 0.17 |  |  | Count | P\_Value | Benjamini | | --- | --- | --- | --- | --- | --- | --- | --- | |  | GOTERM\_CC\_5 | SAGA-type complex | **RT** |  | 3 | 3.7E-1 | 9.3E-1 | |  | GOTERM\_CC\_5 | histone acetyltransferase complex | **RT** |  | 4 | 7.5E-1 | 1.0E0 | |  | GOTERM\_CC\_5 | protein acetyltransferase complex | **RT** |  | 4 | 8.1E-1 | 1.0E0 | |  | GOTERM\_CC\_5 | acetyltransferase complex | **RT** |  | 4 | 8.1E-1 | 1.0E0 | |  | GOTERM\_BP\_5 | covalent chromatin modification | **RT** |  | 10 | 8.3E-1 | 1.0E0 | | Annotation Cluster 46 | | Enrichment Score: 0.14 |  |  | Count | P\_Value | Benjamini | | --- | --- | --- | --- | --- | --- | --- | --- | |  | GOTERM\_BP\_5 | apoptotic process | **RT** |  | 14 | 4.5E-1 | 1.0E0 | |  | GOTERM\_BP\_5 | regulation of apoptotic process | **RT** |  | 10 | 7.0E-1 | 1.0E0 | |  | GOTERM\_BP\_5 | negative regulation of apoptotic process | **RT** |  | 5 | 7.8E-1 | 1.0E0 | |  | GOTERM\_BP\_5 | regulation of programmed cell death | **RT** |  | 10 | 8.2E-1 | 1.0E0 | |  | GOTERM\_BP\_5 | negative regulation of programmed cell death | **RT** |  | 5 | 8.3E-1 | 1.0E0 | |  | GOTERM\_BP\_5 | negative regulation of cell death | **RT** |  | 5 | 9.2E-1 | 1.0E0 | | Annotation Cluster 47 | | Enrichment Score: 0.14 |  |  | Count | P\_Value | Benjamini | | --- | --- | --- | --- | --- | --- | --- | --- | |  | GOTERM\_BP\_5 | negative regulation of gene silencing | **RT** |  | 3 | 4.3E-1 | 1.0E0 | |  | GOTERM\_BP\_5 | regulation of gene silencing | **RT** |  | 6 | 4.7E-1 | 1.0E0 | |  | GOTERM\_BP\_5 | regulation of chromatin silencing | **RT** |  | 3 | 7.7E-1 | 1.0E0 | |  | GOTERM\_BP\_5 | regulation of gene expression, epigenetic | **RT** |  | 8 | 8.5E-1 | 1.0E0 | |  | GOTERM\_BP\_5 | posttranscriptional gene silencing by RNA | **RT** |  | 3 | 8.5E-1 | 1.0E0 | |  | GOTERM\_BP\_5 | gene silencing | **RT** |  | 8 | 9.0E-1 | 1.0E0 | |  | GOTERM\_BP\_5 | negative regulation of gene expression, epigenetic | **RT** |  | 4 | 9.0E-1 | 1.0E0 | |  | GOTERM\_BP\_5 | chromatin silencing | **RT** |  | 4 | 9.0E-1 | 1.0E0 | | Annotation Cluster 48 | | Enrichment Score: 0.13 |  |  | Count | P\_Value | Benjamini | | --- | --- | --- | --- | --- | --- | --- | --- | |  | GOTERM\_CC\_5 | Golgi membrane | **RT** |  | 9 | 6.4E-1 | 9.9E-1 | |  | GOTERM\_CC\_5 | Golgi apparatus | **RT** |  | 19 | 7.3E-1 | 9.9E-1 | |  | GOTERM\_CC\_5 | Golgi apparatus part | **RT** |  | 11 | 9.0E-1 | 1.0E0 | | Annotation Cluster 49 | | Enrichment Score: 0.13 |  |  | Count | P\_Value | Benjamini | | --- | --- | --- | --- | --- | --- | --- | --- | |  | GOTERM\_CC\_5 | AP-type membrane coat adaptor complex | **RT** |  | 3 | 3.3E-1 | 9.1E-1 | |  | GOTERM\_BP\_5 | vesicle coating | **RT** |  | 3 | 3.6E-1 | 1.0E0 | |  | GOTERM\_BP\_5 | synaptic vesicle budding from presynaptic endocytic zone membrane | **RT** |  | 3 | 4.0E-1 | 1.0E0 | |  | GOTERM\_CC\_5 | coated vesicle | **RT** |  | 6 | 4.1E-1 | 9.4E-1 | |  | GOTERM\_BP\_5 | synaptic vesicle budding | **RT** |  | 3 | 4.3E-1 | 1.0E0 | |  | GOTERM\_CC\_5 | membrane coat | **RT** |  | 4 | 5.5E-1 | 9.8E-1 | |  | GOTERM\_BP\_5 | membrane budding | **RT** |  | 3 | 6.2E-1 | 1.0E0 | |  | GOTERM\_BP\_5 | neurotransmitter secretion | **RT** |  | 9 | 6.3E-1 | 1.0E0 | |  | GOTERM\_BP\_5 | signal release from synapse | **RT** |  | 9 | 6.3E-1 | 1.0E0 | |  | GOTERM\_CC\_5 | coated vesicle membrane | **RT** |  | 3 | 7.6E-1 | 1.0E0 | |  | GOTERM\_BP\_5 | vesicle mediated transport in synapse | **RT** |  | 8 | 7.6E-1 | 1.0E0 | |  | GOTERM\_BP\_5 | neurotransmitter transport | **RT** |  | 9 | 7.7E-1 | 1.0E0 | |  | GOTERM\_BP\_5 | exocytosis | **RT** |  | 6 | 8.3E-1 | 1.0E0 | |  | GOTERM\_BP\_5 | signal release | **RT** |  | 9 | 8.4E-1 | 1.0E0 | |  | GOTERM\_BP\_5 | establishment of synaptic vesicle localization | **RT** |  | 8 | 8.4E-1 | 1.0E0 | |  | GOTERM\_BP\_5 | synaptic vesicle transport | **RT** |  | 8 | 8.4E-1 | 1.0E0 | |  | GOTERM\_BP\_5 | synaptic vesicle recycling | **RT** |  | 4 | 8.4E-1 | 1.0E0 | |  | GOTERM\_BP\_5 | synaptic vesicle exocytosis | **RT** |  | 4 | 8.5E-1 | 1.0E0 | |  | GOTERM\_BP\_5 | synaptic vesicle localization | **RT** |  | 8 | 8.6E-1 | 1.0E0 | |  | GOTERM\_BP\_5 | regulated exocytosis | **RT** |  | 4 | 8.7E-1 | 1.0E0 | |  | GOTERM\_BP\_5 | establishment of vesicle localization | **RT** |  | 8 | 8.8E-1 | 1.0E0 | |  | GOTERM\_CC\_5 | transport vesicle | **RT** |  | 6 | 8.9E-1 | 1.0E0 | |  | GOTERM\_BP\_5 | secretion by cell | **RT** |  | 12 | 9.1E-1 | 1.0E0 | |  | GOTERM\_CC\_5 | secretory vesicle | **RT** |  | 5 | 9.1E-1 | 1.0E0 | |  | GOTERM\_BP\_5 | secretion | **RT** |  | 12 | 9.2E-1 | 1.0E0 | |  | GOTERM\_CC\_5 | synaptic vesicle | **RT** |  | 4 | 9.3E-1 | 1.0E0 | |  | GOTERM\_CC\_5 | cytoplasmic vesicle membrane | **RT** |  | 3 | 9.4E-1 | 1.0E0 | |  | GOTERM\_CC\_5 | exocytic vesicle | **RT** |  | 4 | 9.5E-1 | 1.0E0 | |  | GOTERM\_CC\_5 | intracellular vesicle | **RT** |  | 9 | 9.5E-1 | 1.0E0 | |  | GOTERM\_CC\_5 | cytoplasmic vesicle | **RT** |  | 9 | 9.5E-1 | 1.0E0 | |  | GOTERM\_CC\_5 | cytoplasmic vesicle part | **RT** |  | 3 | 9.5E-1 | 1.0E0 | |  | GOTERM\_CC\_5 | cytoplasmic, membrane-bounded vesicle | **RT** |  | 8 | 9.6E-1 | 1.0E0 | |  | GOTERM\_BP\_5 | trans-synaptic signaling | **RT** |  | 12 | 9.9E-1 | 1.0E0 | |  | GOTERM\_BP\_5 | synaptic signaling | **RT** |  | 12 | 9.9E-1 | 1.0E0 | | Annotation Cluster 50 | | Enrichment Score: 0.12 |  |  | Count | P\_Value | Benjamini | | --- | --- | --- | --- | --- | --- | --- | --- | |  | GOTERM\_BP\_5 | mushroom body development | **RT** |  | 6 | 6.2E-1 | 1.0E0 | |  | GOTERM\_BP\_5 | brain development | **RT** |  | 8 | 7.0E-1 | 1.0E0 | |  | GOTERM\_BP\_5 | central nervous system development | **RT** |  | 9 | 9.9E-1 | 1.0E0 | | Annotation Cluster 51 | | Enrichment Score: 0.12 |  |  | Count | P\_Value | Benjamini | | --- | --- | --- | --- | --- | --- | --- | --- | |  | GOTERM\_BP\_5 | regulation of insulin receptor signaling pathway | **RT** |  | 3 | 4.3E-1 | 1.0E0 | |  | GOTERM\_BP\_5 | cellular response to peptide hormone stimulus | **RT** |  | 3 | 7.8E-1 | 1.0E0 | |  | GOTERM\_BP\_5 | cellular response to peptide | **RT** |  | 3 | 7.8E-1 | 1.0E0 | |  | GOTERM\_BP\_5 | response to insulin | **RT** |  | 3 | 8.0E-1 | 1.0E0 | |  | GOTERM\_BP\_5 | response to peptide | **RT** |  | 3 | 8.1E-1 | 1.0E0 | |  | GOTERM\_BP\_5 | response to peptide hormone | **RT** |  | 3 | 8.1E-1 | 1.0E0 | |  | GOTERM\_BP\_5 | cellular response to organonitrogen compound | **RT** |  | 3 | 8.9E-1 | 1.0E0 | |  | GOTERM\_BP\_5 | cellular response to hormone stimulus | **RT** |  | 4 | 9.3E-1 | 1.0E0 | | Annotation Cluster 52 | | Enrichment Score: 0.11 |  |  | Count | P\_Value | Benjamini | | --- | --- | --- | --- | --- | --- | --- | --- | |  | GOTERM\_CC\_5 | ubiquitin ligase complex | **RT** |  | 9 | 6.8E-1 | 9.9E-1 | |  | GOTERM\_CC\_5 | cullin-RING ubiquitin ligase complex | **RT** |  | 5 | 8.1E-1 | 1.0E0 | |  | GOTERM\_CC\_5 | SCF ubiquitin ligase complex | **RT** |  | 3 | 8.5E-1 | 1.0E0 | | Annotation Cluster 53 | | Enrichment Score: 0.11 |  |  | Count | P\_Value | Benjamini | | --- | --- | --- | --- | --- | --- | --- | --- | |  | GOTERM\_BP\_5 | glycoprotein metabolic process | **RT** |  | 6 | 6.9E-1 | 1.0E0 | |  | GOTERM\_BP\_5 | glycoprotein biosynthetic process | **RT** |  | 6 | 6.9E-1 | 1.0E0 | |  | GOTERM\_BP\_5 | macromolecule glycosylation | **RT** |  | 4 | 8.8E-1 | 1.0E0 | |  | GOTERM\_BP\_5 | glycosylation | **RT** |  | 4 | 8.8E-1 | 1.0E0 | | Annotation Cluster 54 | | Enrichment Score: 0.1 |  |  | Count | P\_Value | Benjamini | | --- | --- | --- | --- | --- | --- | --- | --- | |  | GOTERM\_BP\_5 | negative regulation of protein metabolic process | **RT** |  | 12 | 6.4E-1 | 1.0E0 | |  | GOTERM\_BP\_5 | regulation of protein metabolic process | **RT** |  | 31 | 7.2E-1 | 1.0E0 | |  | GOTERM\_BP\_5 | negative regulation of cellular protein metabolic process | **RT** |  | 11 | 7.4E-1 | 1.0E0 | |  | GOTERM\_BP\_5 | regulation of cellular protein metabolic process | **RT** |  | 28 | 7.8E-1 | 1.0E0 | |  | GOTERM\_BP\_5 | regulation of protein modification process | **RT** |  | 14 | 8.8E-1 | 1.0E0 | |  | GOTERM\_BP\_5 | positive regulation of protein metabolic process | **RT** |  | 12 | 8.9E-1 | 1.0E0 | |  | GOTERM\_BP\_5 | positive regulation of cellular protein metabolic process | **RT** |  | 11 | 9.1E-1 | 1.0E0 | | Annotation Cluster 55 | | Enrichment Score: 0.1 |  |  | Count | P\_Value | Benjamini | | --- | --- | --- | --- | --- | --- | --- | --- | |  | GOTERM\_MF\_5 | thiol-dependent ubiquitin-specific protease activity | **RT** |  | 3 | 7.1E-1 | 1.0E0 | |  | GOTERM\_BP\_5 | protein modification by small protein removal | **RT** |  | 4 | 8.2E-1 | 1.0E0 | |  | GOTERM\_MF\_5 | cysteine-type peptidase activity | **RT** |  | 4 | 8.6E-1 | 1.0E0 | | Annotation Cluster 56 | | Enrichment Score: 0.1 |  |  | Count | P\_Value | Benjamini | | --- | --- | --- | --- | --- | --- | --- | --- | |  | GOTERM\_BP\_5 | regulation of chromosome organization | **RT** |  | 7 | 6.5E-1 | 1.0E0 | |  | GOTERM\_BP\_5 | covalent chromatin modification | **RT** |  | 10 | 8.3E-1 | 1.0E0 | |  | GOTERM\_BP\_5 | chromatin organization | **RT** |  | 15 | 9.3E-1 | 1.0E0 | | Annotation Cluster 57 | | Enrichment Score: 0.09 |  |  | Count | P\_Value | Benjamini | | --- | --- | --- | --- | --- | --- | --- | --- | |  | GOTERM\_BP\_5 | regulation of nucleocytoplasmic transport | **RT** |  | 3 | 6.5E-1 | 1.0E0 | |  | GOTERM\_BP\_5 | regulation of intracellular protein transport | **RT** |  | 3 | 7.3E-1 | 1.0E0 | |  | GOTERM\_BP\_5 | nuclear transport | **RT** |  | 6 | 8.0E-1 | 1.0E0 | |  | GOTERM\_BP\_5 | nucleocytoplasmic transport | **RT** |  | 6 | 8.0E-1 | 1.0E0 | |  | GOTERM\_BP\_5 | regulation of intracellular transport | **RT** |  | 3 | 8.5E-1 | 1.0E0 | |  | GOTERM\_BP\_5 | regulation of establishment of protein localization | **RT** |  | 4 | 8.6E-1 | 1.0E0 | |  | GOTERM\_BP\_5 | regulation of cellular protein localization | **RT** |  | 3 | 9.0E-1 | 1.0E0 | |  | GOTERM\_BP\_5 | regulation of protein transport | **RT** |  | 3 | 9.4E-1 | 1.0E0 | | Annotation Cluster 58 | | Enrichment Score: 0.09 |  |  | Count | P\_Value | Benjamini | | --- | --- | --- | --- | --- | --- | --- | --- | |  | GOTERM\_CC\_5 | microtubule organizing center part | **RT** |  | 3 | 6.5E-1 | 9.9E-1 | |  | GOTERM\_CC\_5 | centrosome | **RT** |  | 5 | 9.0E-1 | 1.0E0 | |  | GOTERM\_CC\_5 | microtubule organizing center | **RT** |  | 5 | 9.1E-1 | 1.0E0 | | Annotation Cluster 59 | | Enrichment Score: 0.09 |  |  | Count | P\_Value | Benjamini | | --- | --- | --- | --- | --- | --- | --- | --- | |  | GOTERM\_BP\_5 | regulation of mRNA processing | **RT** |  | 5 | 8.1E-1 | 1.0E0 | |  | GOTERM\_BP\_5 | regulation of RNA splicing | **RT** |  | 5 | 8.1E-1 | 1.0E0 | |  | GOTERM\_BP\_5 | regulation of mRNA metabolic process | **RT** |  | 5 | 8.4E-1 | 1.0E0 | | Annotation Cluster 60 | | Enrichment Score: 0.08 |  |  | Count | P\_Value | Benjamini | | --- | --- | --- | --- | --- | --- | --- | --- | |  | GOTERM\_BP\_5 | defense response to Gram-negative bacterium | **RT** |  | 4 | 8.1E-1 | 1.0E0 | |  | GOTERM\_BP\_5 | response to bacterium | **RT** |  | 7 | 8.2E-1 | 1.0E0 | |  | GOTERM\_BP\_5 | defense response to other organism | **RT** |  | 8 | 8.5E-1 | 1.0E0 | |  | GOTERM\_BP\_5 | defense response to bacterium | **RT** |  | 6 | 8.7E-1 | 1.0E0 | | Annotation Cluster 61 | | Enrichment Score: 0.07 |  |  | Count | P\_Value | Benjamini | | --- | --- | --- | --- | --- | --- | --- | --- | |  | GOTERM\_MF\_5 | GTP binding | **RT** |  | 8 | 7.9E-1 | 1.0E0 | |  | GOTERM\_MF\_5 | guanyl nucleotide binding | **RT** |  | 8 | 8.1E-1 | 1.0E0 | |  | GOTERM\_MF\_5 | guanyl ribonucleotide binding | **RT** |  | 8 | 8.1E-1 | 1.0E0 | |  | GOTERM\_BP\_5 | small GTPase mediated signal transduction | **RT** |  | 8 | 1.0E0 | 1.0E0 | | Annotation Cluster 62 | | Enrichment Score: 0.07 |  |  | Count | P\_Value | Benjamini | | --- | --- | --- | --- | --- | --- | --- | --- | |  | GOTERM\_BP\_5 | positive regulation of gene expression | **RT** |  | 20 | 7.6E-1 | 1.0E0 | |  | GOTERM\_BP\_5 | positive regulation of macromolecule biosynthetic process | **RT** |  | 19 | 7.6E-1 | 1.0E0 | |  | GOTERM\_BP\_5 | transcription, DNA-templated | **RT** |  | 45 | 8.0E-1 | 1.0E0 | |  | GOTERM\_BP\_5 | positive regulation of cellular biosynthetic process | **RT** |  | 20 | 8.3E-1 | 1.0E0 | |  | GOTERM\_BP\_5 | positive regulation of transcription, DNA-templated | **RT** |  | 16 | 8.4E-1 | 1.0E0 | |  | GOTERM\_BP\_5 | positive regulation of RNA biosynthetic process | **RT** |  | 16 | 8.4E-1 | 1.0E0 | |  | GOTERM\_BP\_5 | positive regulation of RNA metabolic process | **RT** |  | 16 | 8.5E-1 | 1.0E0 | |  | GOTERM\_BP\_5 | positive regulation of nucleobase-containing compound metabolic process | **RT** |  | 16 | 9.2E-1 | 1.0E0 | |  | GOTERM\_BP\_5 | regulation of transcription, DNA-templated | **RT** |  | 44 | 9.3E-1 | 1.0E0 | |  | GOTERM\_BP\_5 | regulation of RNA biosynthetic process | **RT** |  | 44 | 9.3E-1 | 1.0E0 | |  | GOTERM\_BP\_5 | positive regulation of macromolecule metabolic process | **RT** |  | 28 | 9.3E-1 | 1.0E0 | | Annotation Cluster 63 | | Enrichment Score: 0.07 |  |  | Count | P\_Value | Benjamini | | --- | --- | --- | --- | --- | --- | --- | --- | |  | GOTERM\_BP\_5 | dorsal appendage formation | **RT** |  | 3 | 7.3E-1 | 1.0E0 | |  | GOTERM\_BP\_5 | eggshell formation | **RT** |  | 4 | 8.1E-1 | 1.0E0 | |  | GOTERM\_BP\_5 | eggshell chorion assembly | **RT** |  | 3 | 8.7E-1 | 1.0E0 | |  | GOTERM\_BP\_5 | chorion-containing eggshell formation | **RT** |  | 3 | 9.3E-1 | 1.0E0 | |  | GOTERM\_BP\_5 | cellular component assembly involved in morphogenesis | **RT** |  | 4 | 9.7E-1 | 1.0E0 | | Annotation Cluster 64 | | Enrichment Score: 0.06 |  |  | Count | P\_Value | Benjamini | | --- | --- | --- | --- | --- | --- | --- | --- | |  | GOTERM\_BP\_5 | negative regulation of developmental growth | **RT** |  | 6 | 6.2E-1 | 1.0E0 | |  | GOTERM\_BP\_5 | negative regulation of nervous system development | **RT** |  | 7 | 7.9E-1 | 1.0E0 | |  | GOTERM\_BP\_5 | negative regulation of synaptic growth at neuromuscular junction | **RT** |  | 3 | 8.6E-1 | 1.0E0 | |  | GOTERM\_BP\_5 | negative regulation of neuromuscular junction development | **RT** |  | 3 | 8.6E-1 | 1.0E0 | |  | GOTERM\_BP\_5 | negative regulation of synapse assembly | **RT** |  | 3 | 8.6E-1 | 1.0E0 | |  | GOTERM\_BP\_5 | regulation of synaptic growth at neuromuscular junction | **RT** |  | 6 | 9.2E-1 | 1.0E0 | |  | GOTERM\_BP\_5 | regulation of neuromuscular junction development | **RT** |  | 6 | 9.3E-1 | 1.0E0 | |  | GOTERM\_BP\_5 | regulation of synapse assembly | **RT** |  | 6 | 9.4E-1 | 1.0E0 | |  | GOTERM\_BP\_5 | synaptic growth at neuromuscular junction | **RT** |  | 6 | 9.4E-1 | 1.0E0 | |  | GOTERM\_BP\_5 | synapse assembly | **RT** |  | 8 | 9.8E-1 | 1.0E0 | |  | GOTERM\_BP\_5 | regulation of nervous system development | **RT** |  | 12 | 9.9E-1 | 1.0E0 | | Annotation Cluster 65 | | Enrichment Score: 0.05 |  |  | Count | P\_Value | Benjamini | | --- | --- | --- | --- | --- | --- | --- | --- | |  | GOTERM\_CC\_5 | microtubule associated complex | **RT** |  | 19 | 7.9E-1 | 1.0E0 | |  | GOTERM\_CC\_5 | cytoskeletal part | **RT** |  | 28 | 8.9E-1 | 1.0E0 | |  | GOTERM\_CC\_5 | microtubule cytoskeleton | **RT** |  | 24 | 9.1E-1 | 1.0E0 | |  | GOTERM\_CC\_5 | cytoskeleton | **RT** |  | 30 | 9.5E-1 | 1.0E0 | | Annotation Cluster 66 | | Enrichment Score: 0.05 |  |  | Count | P\_Value | Benjamini | | --- | --- | --- | --- | --- | --- | --- | --- | |  | GOTERM\_BP\_5 | negative regulation of intracellular signal transduction | **RT** |  | 7 | 5.9E-1 | 1.0E0 | |  | GOTERM\_BP\_5 | negative regulation of signal transduction | **RT** |  | 12 | 9.6E-1 | 1.0E0 | |  | GOTERM\_BP\_5 | negative regulation of cell communication | **RT** |  | 13 | 9.6E-1 | 1.0E0 | |  | GOTERM\_BP\_5 | regulation of intracellular signal transduction | **RT** |  | 12 | 1.0E0 | 1.0E0 | |  | GOTERM\_BP\_5 | regulation of signal transduction | **RT** |  | 27 | 1.0E0 | 1.0E0 | | Annotation Cluster 67 | | Enrichment Score: 0.05 |  |  | Count | P\_Value | Benjamini | | --- | --- | --- | --- | --- | --- | --- | --- | |  | GOTERM\_CC\_5 | polytene chromosome interband | **RT** |  | 3 | 8.1E-1 | 1.0E0 | |  | GOTERM\_CC\_5 | polytene chromosome puff | **RT** |  | 3 | 8.4E-1 | 1.0E0 | |  | GOTERM\_CC\_5 | chromosomal region | **RT** |  | 8 | 9.3E-1 | 1.0E0 | |  | GOTERM\_CC\_5 | polytene chromosome | **RT** |  | 8 | 9.9E-1 | 1.0E0 | | Annotation Cluster 68 | | Enrichment Score: 0.05 |  |  | Count | P\_Value | Benjamini | | --- | --- | --- | --- | --- | --- | --- | --- | |  | GOTERM\_BP\_5 | regulation of translation | **RT** |  | 9 | 7.7E-1 | 1.0E0 | |  | GOTERM\_BP\_5 | negative regulation of translation | **RT** |  | 3 | 9.7E-1 | 1.0E0 | |  | GOTERM\_BP\_5 | negative regulation of cellular amide metabolic process | **RT** |  | 3 | 9.8E-1 | 1.0E0 | | Annotation Cluster 69 | | Enrichment Score: 0.04 |  |  | Count | P\_Value | Benjamini | | --- | --- | --- | --- | --- | --- | --- | --- | |  | GOTERM\_BP\_5 | male meiosis | **RT** |  | 3 | 8.4E-1 | 1.0E0 | |  | GOTERM\_BP\_5 | meiotic nuclear division | **RT** |  | 5 | 9.3E-1 | 1.0E0 | |  | GOTERM\_BP\_5 | meiotic cell cycle process | **RT** |  | 5 | 9.5E-1 | 1.0E0 | | Annotation Cluster 70 | | Enrichment Score: 0.04 |  |  | Count | P\_Value | Benjamini | | --- | --- | --- | --- | --- | --- | --- | --- | |  | GOTERM\_BP\_5 | dorsal/ventral pattern formation, imaginal disc | **RT** |  | 3 | 8.1E-1 | 1.0E0 | |  | GOTERM\_BP\_5 | wing disc pattern formation | **RT** |  | 3 | 9.0E-1 | 1.0E0 | |  | GOTERM\_BP\_5 | dorsal/ventral pattern formation | **RT** |  | 5 | 9.5E-1 | 1.0E0 | |  | GOTERM\_BP\_5 | imaginal disc pattern formation | **RT** |  | 3 | 9.6E-1 | 1.0E0 | | Annotation Cluster 71 | | Enrichment Score: 0.04 |  |  | Count | P\_Value | Benjamini | | --- | --- | --- | --- | --- | --- | --- | --- | |  | GOTERM\_BP\_5 | actin filament polymerization | **RT** |  | 3 | 8.1E-1 | 1.0E0 | |  | GOTERM\_CC\_5 | actin cytoskeleton | **RT** |  | 5 | 8.3E-1 | 1.0E0 | |  | GOTERM\_BP\_5 | regulation of actin filament polymerization | **RT** |  | 3 | 8.5E-1 | 1.0E0 | |  | GOTERM\_BP\_5 | regulation of protein polymerization | **RT** |  | 3 | 8.8E-1 | 1.0E0 | |  | GOTERM\_BP\_5 | regulation of actin polymerization or depolymerization | **RT** |  | 3 | 8.8E-1 | 1.0E0 | |  | GOTERM\_BP\_5 | regulation of actin filament length | **RT** |  | 3 | 8.8E-1 | 1.0E0 | |  | GOTERM\_BP\_5 | regulation of actin cytoskeleton organization | **RT** |  | 3 | 9.8E-1 | 1.0E0 | |  | GOTERM\_BP\_5 | actin filament organization | **RT** |  | 5 | 9.9E-1 | 1.0E0 | |  | GOTERM\_BP\_5 | regulation of cytoskeleton organization | **RT** |  | 4 | 9.9E-1 | 1.0E0 | |  | GOTERM\_BP\_5 | actin cytoskeleton organization | **RT** |  | 6 | 1.0E0 | 1.0E0 | | Annotation Cluster 72 | | Enrichment Score: 0.03 |  |  | Count | P\_Value | Benjamini | | --- | --- | --- | --- | --- | --- | --- | --- | |  | GOTERM\_BP\_5 | head involution | **RT** |  | 3 | 6.5E-1 | 1.0E0 | |  | GOTERM\_BP\_5 | embryo development ending in birth or egg hatching | **RT** |  | 9 | 8.8E-1 | 1.0E0 | |  | GOTERM\_BP\_5 | dorsal closure | **RT** |  | 4 | 9.6E-1 | 1.0E0 | |  | GOTERM\_BP\_5 | embryonic morphogenesis | **RT** |  | 8 | 9.7E-1 | 1.0E0 | |  | GOTERM\_BP\_5 | morphogenesis of embryonic epithelium | **RT** |  | 4 | 9.8E-1 | 1.0E0 | |  | GOTERM\_BP\_5 | compound eye development | **RT** |  | 7 | 1.0E0 | 1.0E0 | |  | GOTERM\_BP\_5 | sensory organ morphogenesis | **RT** |  | 5 | 1.0E0 | 1.0E0 | |  | GOTERM\_BP\_5 | eye morphogenesis | **RT** |  | 5 | 1.0E0 | 1.0E0 | |  | GOTERM\_BP\_5 | eye development | **RT** |  | 7 | 1.0E0 | 1.0E0 | |  | GOTERM\_BP\_5 | sensory organ development | **RT** |  | 10 | 1.0E0 | 1.0E0 | | Annotation Cluster 73 | | Enrichment Score: 0.03 |  |  | Count | P\_Value | Benjamini | | --- | --- | --- | --- | --- | --- | --- | --- | |  | GOTERM\_BP\_5 | branching morphogenesis of an epithelial tube | **RT** |  | 3 | 9.1E-1 | 1.0E0 | |  | GOTERM\_BP\_5 | morphogenesis of a branching epithelium | **RT** |  | 3 | 9.1E-1 | 1.0E0 | |  | GOTERM\_BP\_5 | branching involved in open tracheal system development | **RT** |  | 3 | 9.1E-1 | 1.0E0 | |  | GOTERM\_BP\_5 | respiratory system development | **RT** |  | 6 | 1.0E0 | 1.0E0 | |  | GOTERM\_BP\_5 | open tracheal system development | **RT** |  | 5 | 1.0E0 | 1.0E0 | | Annotation Cluster 74 | | Enrichment Score: 0.02 |  |  | Count | P\_Value | Benjamini | | --- | --- | --- | --- | --- | --- | --- | --- | |  | GOTERM\_CC\_5 | cullin-RING ubiquitin ligase complex | **RT** |  | 5 | 8.1E-1 | 1.0E0 | |  | GOTERM\_BP\_5 | mitotic cell cycle phase transition | **RT** |  | 6 | 9.4E-1 | 1.0E0 | |  | GOTERM\_BP\_5 | cell cycle checkpoint | **RT** |  | 4 | 9.4E-1 | 1.0E0 | |  | GOTERM\_BP\_5 | cell cycle phase transition | **RT** |  | 6 | 9.4E-1 | 1.0E0 | |  | GOTERM\_BP\_5 | negative regulation of cell cycle phase transition | **RT** |  | 4 | 9.5E-1 | 1.0E0 | |  | GOTERM\_BP\_5 | negative regulation of mitotic cell cycle phase transition | **RT** |  | 4 | 9.5E-1 | 1.0E0 | |  | GOTERM\_BP\_5 | regulation of cell cycle phase transition | **RT** |  | 5 | 9.5E-1 | 1.0E0 | |  | GOTERM\_BP\_5 | regulation of mitotic cell cycle phase transition | **RT** |  | 5 | 9.5E-1 | 1.0E0 | |  | GOTERM\_BP\_5 | negative regulation of mitotic cell cycle | **RT** |  | 4 | 9.6E-1 | 1.0E0 | |  | GOTERM\_BP\_5 | negative regulation of cell cycle | **RT** |  | 5 | 9.6E-1 | 1.0E0 | |  | GOTERM\_BP\_5 | negative regulation of cell cycle process | **RT** |  | 4 | 9.7E-1 | 1.0E0 | |  | GOTERM\_BP\_5 | regulation of mitotic cell cycle | **RT** |  | 6 | 9.7E-1 | 1.0E0 | |  | GOTERM\_BP\_5 | mitotic cell cycle checkpoint | **RT** |  | 3 | 9.8E-1 | 1.0E0 | |  | GOTERM\_BP\_5 | regulation of cell cycle process | **RT** |  | 5 | 9.9E-1 | 1.0E0 | | Annotation Cluster 75 | | Enrichment Score: 0.02 |  |  | Count | P\_Value | Benjamini | | --- | --- | --- | --- | --- | --- | --- | --- | |  | GOTERM\_BP\_5 | columnar/cuboidal epithelial cell development | **RT** |  | 13 | 9.1E-1 | 1.0E0 | |  | GOTERM\_BP\_5 | ovarian follicle cell development | **RT** |  | 13 | 9.1E-1 | 1.0E0 | |  | GOTERM\_BP\_5 | epithelial cell development | **RT** |  | 13 | 9.5E-1 | 1.0E0 | |  | GOTERM\_BP\_5 | epithelial cell differentiation | **RT** |  | 13 | 9.8E-1 | 1.0E0 | |  | GOTERM\_BP\_5 | oogenesis | **RT** |  | 25 | 9.9E-1 | 1.0E0 | |  | GOTERM\_BP\_5 | female gamete generation | **RT** |  | 25 | 9.9E-1 | 1.0E0 | |  | GOTERM\_BP\_5 | germ cell development | **RT** |  | 28 | 9.9E-1 | 1.0E0 | |  | GOTERM\_BP\_5 | gamete generation | **RT** |  | 30 | 1.0E0 | 1.0E0 | | Annotation Cluster 76 | | Enrichment Score: 0.02 |  |  | Count | P\_Value | Benjamini | | --- | --- | --- | --- | --- | --- | --- | --- | |  | GOTERM\_BP\_5 | intracellular mRNA localization involved in anterior/posterior axis specification | **RT** |  | 3 | 8.4E-1 | 1.0E0 | |  | GOTERM\_BP\_5 | intracellular mRNA localization involved in pattern specification process | **RT** |  | 3 | 8.6E-1 | 1.0E0 | |  | GOTERM\_BP\_5 | oocyte anterior/posterior axis specification | **RT** |  | 3 | 9.5E-1 | 1.0E0 | |  | GOTERM\_BP\_5 | anterior/posterior axis specification | **RT** |  | 4 | 9.7E-1 | 1.0E0 | |  | GOTERM\_BP\_5 | tripartite regional subdivision | **RT** |  | 4 | 9.7E-1 | 1.0E0 | |  | GOTERM\_BP\_5 | oocyte axis specification | **RT** |  | 4 | 9.7E-1 | 1.0E0 | |  | GOTERM\_BP\_5 | embryonic axis specification | **RT** |  | 4 | 9.8E-1 | 1.0E0 | |  | GOTERM\_BP\_5 | oocyte construction | **RT** |  | 4 | 9.8E-1 | 1.0E0 | |  | GOTERM\_BP\_5 | axis specification | **RT** |  | 6 | 9.8E-1 | 1.0E0 | |  | GOTERM\_BP\_5 | oocyte differentiation | **RT** |  | 5 | 9.8E-1 | 1.0E0 | |  | GOTERM\_BP\_5 | anterior/posterior pattern specification | **RT** |  | 4 | 9.9E-1 | 1.0E0 | |  | GOTERM\_BP\_5 | cell maturation | **RT** |  | 6 | 9.9E-1 | 1.0E0 | |  | GOTERM\_BP\_5 | oocyte development | **RT** |  | 4 | 9.9E-1 | 1.0E0 | |  | GOTERM\_BP\_5 | blastoderm segmentation | **RT** |  | 5 | 9.9E-1 | 1.0E0 | |  | GOTERM\_BP\_5 | segmentation | **RT** |  | 6 | 9.9E-1 | 1.0E0 | |  | GOTERM\_BP\_5 | embryonic pattern specification | **RT** |  | 5 | 1.0E0 | 1.0E0 | |  | GOTERM\_BP\_5 | regionalization | **RT** |  | 13 | 1.0E0 | 1.0E0 | | Annotation Cluster 77 | | Enrichment Score: 0.01 |  |  | Count | P\_Value | Benjamini | | --- | --- | --- | --- | --- | --- | --- | --- | |  | GOTERM\_BP\_5 | spermatid differentiation | **RT** |  | 4 | 9.3E-1 | 1.0E0 | |  | GOTERM\_BP\_5 | spermatid development | **RT** |  | 3 | 9.7E-1 | 1.0E0 | |  | GOTERM\_BP\_5 | male gamete generation | **RT** |  | 6 | 9.8E-1 | 1.0E0 | |  | GOTERM\_BP\_5 | spermatogenesis | **RT** |  | 6 | 9.8E-1 | 1.0E0 | | Annotation Cluster 78 | | Enrichment Score: 0.01 |  |  | Count | P\_Value | Benjamini | | --- | --- | --- | --- | --- | --- | --- | --- | |  | GOTERM\_BP\_5 | negative regulation of macromolecule metabolic process | **RT** |  | 25 | 9.3E-1 | 1.0E0 | |  | GOTERM\_BP\_5 | negative regulation of macromolecule biosynthetic process | **RT** |  | 14 | 9.7E-1 | 1.0E0 | |  | GOTERM\_BP\_5 | negative regulation of cellular macromolecule biosynthetic process | **RT** |  | 14 | 9.7E-1 | 1.0E0 | |  | GOTERM\_BP\_5 | negative regulation of RNA metabolic process | **RT** |  | 11 | 9.7E-1 | 1.0E0 | |  | GOTERM\_BP\_5 | negative regulation of transcription, DNA-templated | **RT** |  | 10 | 9.7E-1 | 1.0E0 | |  | GOTERM\_BP\_5 | negative regulation of RNA biosynthetic process | **RT** |  | 10 | 9.7E-1 | 1.0E0 | |  | GOTERM\_BP\_5 | negative regulation of cellular biosynthetic process | **RT** |  | 14 | 9.8E-1 | 1.0E0 | |  | GOTERM\_BP\_5 | negative regulation of gene expression | **RT** |  | 17 | 9.8E-1 | 1.0E0 | |  | GOTERM\_BP\_5 | negative regulation of nucleobase-containing compound metabolic process | **RT** |  | 11 | 9.8E-1 | 1.0E0 | | Annotation Cluster 79 | | Enrichment Score: 0.01 |  |  | Count | P\_Value | Benjamini | | --- | --- | --- | --- | --- | --- | --- | --- | |  | GOTERM\_BP\_5 | mitotic sister chromatid segregation | **RT** |  | 3 | 9.6E-1 | 1.0E0 | |  | GOTERM\_BP\_5 | sister chromatid segregation | **RT** |  | 3 | 9.7E-1 | 1.0E0 | |  | GOTERM\_BP\_5 | nuclear chromosome segregation | **RT** |  | 4 | 9.8E-1 | 1.0E0 | | Annotation Cluster 80 | | Enrichment Score: 0.01 |  |  | Count | P\_Value | Benjamini | | --- | --- | --- | --- | --- | --- | --- | --- | |  | GOTERM\_BP\_5 | neurogenesis | **RT** |  | 74 | 7.1E-1 | 1.0E0 | |  | GOTERM\_BP\_5 | axon guidance | **RT** |  | 11 | 9.9E-1 | 1.0E0 | |  | GOTERM\_BP\_5 | neuron projection guidance | **RT** |  | 11 | 9.9E-1 | 1.0E0 | |  | GOTERM\_BP\_5 | cell part morphogenesis | **RT** |  | 27 | 9.9E-1 | 1.0E0 | |  | GOTERM\_BP\_5 | neuron projection morphogenesis | **RT** |  | 25 | 9.9E-1 | 1.0E0 | |  | GOTERM\_BP\_5 | cell projection morphogenesis | **RT** |  | 25 | 1.0E0 | 1.0E0 | |  | GOTERM\_BP\_5 | neuron projection development | **RT** |  | 25 | 1.0E0 | 1.0E0 | |  | GOTERM\_BP\_5 | dendrite development | **RT** |  | 7 | 1.0E0 | 1.0E0 | |  | GOTERM\_BP\_5 | axon development | **RT** |  | 12 | 1.0E0 | 1.0E0 | |  | GOTERM\_BP\_5 | cell morphogenesis | **RT** |  | 30 | 1.0E0 | 1.0E0 | |  | GOTERM\_BP\_5 | cell development | **RT** |  | 61 | 1.0E0 | 1.0E0 | |  | GOTERM\_BP\_5 | cell morphogenesis involved in neuron differentiation | **RT** |  | 17 | 1.0E0 | 1.0E0 | |  | GOTERM\_BP\_5 | neuron development | **RT** |  | 28 | 1.0E0 | 1.0E0 | |  | GOTERM\_BP\_5 | cell morphogenesis involved in differentiation | **RT** |  | 18 | 1.0E0 | 1.0E0 | |  | GOTERM\_BP\_5 | neuron differentiation | **RT** |  | 30 | 1.0E0 | 1.0E0 | | Annotation Cluster 81 | | Enrichment Score: 0.01 |  |  | Count | P\_Value | Benjamini | | --- | --- | --- | --- | --- | --- | --- | --- | |  | GOTERM\_BP\_5 | cellular ion homeostasis | **RT** |  | 3 | 9.8E-1 | 1.0E0 | |  | GOTERM\_BP\_5 | cellular chemical homeostasis | **RT** |  | 3 | 9.9E-1 | 1.0E0 | |  | GOTERM\_BP\_5 | ion homeostasis | **RT** |  | 3 | 9.9E-1 | 1.0E0 | | Annotation Cluster 82 | | Enrichment Score: 0 |  |  | Count | P\_Value | Benjamini | | --- | --- | --- | --- | --- | --- | --- | --- | |  | GOTERM\_CC\_5 | cell cortex | **RT** |  | 6 | 9.8E-1 | 1.0E0 | |  | GOTERM\_CC\_5 | cytoplasmic region | **RT** |  | 6 | 9.9E-1 | 1.0E0 | |  | GOTERM\_BP\_5 | regulation of cell morphogenesis | **RT** |  | 5 | 1.0E0 | 1.0E0 | | Annotation Cluster 83 | | Enrichment Score: 0 |  |  | Count | P\_Value | Benjamini | | --- | --- | --- | --- | --- | --- | --- | --- | |  | GOTERM\_BP\_5 | regulation of cell morphogenesis involved in differentiation | **RT** |  | 3 | 9.9E-1 | 1.0E0 | |  | GOTERM\_BP\_5 | regulation of neuron projection development | **RT** |  | 3 | 1.0E0 | 1.0E0 | |  | GOTERM\_BP\_5 | regulation of neurogenesis | **RT** |  | 6 | 1.0E0 | 1.0E0 | |  | GOTERM\_BP\_5 | regulation of cell morphogenesis | **RT** |  | 5 | 1.0E0 | 1.0E0 | |  | GOTERM\_BP\_5 | regulation of neuron differentiation | **RT** |  | 3 | 1.0E0 | 1.0E0 | | Annotation Cluster 84 | | Enrichment Score: 0 |  |  | Count | P\_Value | Benjamini | | --- | --- | --- | --- | --- | --- | --- | --- | |  | GOTERM\_MF\_5 | purine ribonucleoside binding | **RT** |  | 28 | 9.9E-1 | 1.0E0 | |  | GOTERM\_MF\_5 | purine ribonucleotide binding | **RT** |  | 28 | 9.9E-1 | 1.0E0 | |  | GOTERM\_MF\_5 | purine nucleotide binding | **RT** |  | 28 | 1.0E0 | 1.0E0 | |  | GOTERM\_MF\_5 | ribonucleotide binding | **RT** |  | 28 | 1.0E0 | 1.0E0 | |  | GOTERM\_MF\_5 | ATP binding | **RT** |  | 20 | 1.0E0 | 1.0E0 | |  | GOTERM\_MF\_5 | adenyl ribonucleotide binding | **RT** |  | 20 | 1.0E0 | 1.0E0 | |  | GOTERM\_MF\_5 | adenyl nucleotide binding | **RT** |  | 20 | 1.0E0 | 1.0E0 | | Annotation Cluster 85 | | Enrichment Score: 0 |  |  | Count | P\_Value | Benjamini | | --- | --- | --- | --- | --- | --- | --- | --- | |  | GOTERM\_BP\_5 | instar larval or pupal morphogenesis | **RT** |  | 24 | 9.7E-1 | 1.0E0 | |  | GOTERM\_BP\_5 | post-embryonic morphogenesis | **RT** |  | 24 | 9.8E-1 | 1.0E0 | |  | GOTERM\_BP\_5 | imaginal disc development | **RT** |  | 25 | 9.9E-1 | 1.0E0 | |  | GOTERM\_BP\_5 | instar larval or pupal development | **RT** |  | 24 | 1.0E0 | 1.0E0 | |  | GOTERM\_BP\_5 | wing disc development | **RT** |  | 17 | 1.0E0 | 1.0E0 | |  | GOTERM\_BP\_5 | post-embryonic organ morphogenesis | **RT** |  | 14 | 1.0E0 | 1.0E0 | |  | GOTERM\_BP\_5 | imaginal disc morphogenesis | **RT** |  | 14 | 1.0E0 | 1.0E0 | |  | GOTERM\_BP\_5 | imaginal disc-derived wing morphogenesis | **RT** |  | 9 | 1.0E0 | 1.0E0 | |  | GOTERM\_BP\_5 | epithelial tube morphogenesis | **RT** |  | 15 | 1.0E0 | 1.0E0 | |  | GOTERM\_BP\_5 | tube morphogenesis | **RT** |  | 16 | 1.0E0 | 1.0E0 | |  | GOTERM\_BP\_5 | post-embryonic organ development | **RT** |  | 15 | 1.0E0 | 1.0E0 | |  | GOTERM\_BP\_5 | wing disc morphogenesis | **RT** |  | 9 | 1.0E0 | 1.0E0 | |  | GOTERM\_BP\_5 | post-embryonic appendage morphogenesis | **RT** |  | 9 | 1.0E0 | 1.0E0 | |  | GOTERM\_BP\_5 | appendage morphogenesis | **RT** |  | 9 | 1.0E0 | 1.0E0 | |  | GOTERM\_BP\_5 | imaginal disc-derived appendage morphogenesis | **RT** |  | 9 | 1.0E0 | 1.0E0 | |  | GOTERM\_BP\_5 | imaginal disc-derived appendage development | **RT** |  | 9 | 1.0E0 | 1.0E0 | |  | GOTERM\_BP\_5 | morphogenesis of an epithelium | **RT** |  | 16 | 1.0E0 | 1.0E0 | |  | GOTERM\_BP\_5 | organ morphogenesis | **RT** |  | 25 | 1.0E0 | 1.0E0 | | Annotation Cluster 86 | | Enrichment Score: 0 |  |  | Count | P\_Value | Benjamini | | --- | --- | --- | --- | --- | --- | --- | --- | |  | GOTERM\_BP\_5 | Wnt signaling pathway | **RT** |  | 3 | 1.0E0 | 1.0E0 | |  | GOTERM\_BP\_5 | cell-cell signaling by wnt | **RT** |  | 3 | 1.0E0 | 1.0E0 | |  | GOTERM\_BP\_5 | cell surface receptor signaling pathway involved in cell-cell signaling | **RT** |  | 3 | 1.0E0 | 1.0E0 | | Annotation Cluster 87 | | Enrichment Score: 0 |  |  | Count | P\_Value | Benjamini | | --- | --- | --- | --- | --- | --- | --- | --- | |  | GOTERM\_BP\_5 | positive regulation of intracellular signal transduction | **RT** |  | 3 | 1.0E0 | 1.0E0 | |  | GOTERM\_BP\_5 | positive regulation of signal transduction | **RT** |  | 7 | 1.0E0 | 1.0E0 | |  | GOTERM\_BP\_5 | positive regulation of cell communication | **RT** |  | 8 | 1.0E0 | 1.0E0 | | Annotation Cluster 88 | | Enrichment Score: 0 |  |  | Count | P\_Value | Benjamini | | --- | --- | --- | --- | --- | --- | --- | --- | |  | GOTERM\_CC\_5 | integral component of plasma membrane | **RT** |  | 6 | 1.0E0 | 1.0E0 | |  | GOTERM\_CC\_5 | intrinsic component of plasma membrane | **RT** |  | 6 | 1.0E0 | 1.0E0 | |  | GOTERM\_CC\_5 | plasma membrane part | **RT** |  | 12 | 1.0E0 | 1.0E0 |   were not clustered. | |  | |
