# Supplementary material for: Enabling cell-type-specific behavioral epigenetics in Drosophila: a modified high-yield INTACT method reveals the impact of social environment on the epigenetic landscape in dopaminergic neurons
Source: BMC Biol. 2019 Apr 10;17:30. doi: 10.1186/s12915-019-0646-4 (PMC6456965; doi:10.1186/s12915-019-0646-4)
Supplement: Supplementary file 10 — Gorilla and DAVID functional analysis. The zip file contains top level html files which may be opened in a browser. These will give the Gorilla functional analysis and DAVID GO analyses referred to in the main text. (ZIP 919 kb) [file 12915_2019_646_MOESM10_ESM.zip › Additional File 10/TPM87_GOLevel5.html]

DAVID: Database for Annotation, Visualization, and Integrated Discovery (Laboratory of Human Retrovirology and Immunoinformatics (LHRI); National Institute of Allergies and Infectious Diseases (NIAID); Leidos Biomedical Research, Inc. (LBR)


|  |  |  |  |  |  |
| --- | --- | --- | --- | --- | --- |
| DAVID Bioinformatics 6.7  |  |  |  |  |  | | --- | --- | --- | --- | --- | | |  |  |  |  | | --- | --- | --- | --- | |  | |  | | --- | | DAVID Bioinformatics Resources 6.8 | | Laboratory of Human Retrovirology and Immunoinformatics (LHRI) | | |   100%  **\*\*\* Welcome to DAVID 6.8 \*\*\* \*\*\* If you are looking for DAVID 6.7, please visit our development site. \*\*\*** |
| |  |  |  |  |  |  |  |  |  |  |  |  |  |  |  |  |  |  |  |  |  |  |  |  |  |  |  |  |  |  |  |  |  |  |  |  |  |  |  |  |  |  |  |  |  |  |  |  |  |  |  |  |  |  |  |  |  |  |  |  |  |  |  |  |  |  |  |  |  |  |  |  |  |  |  |  |  |  |  |  |  |  |  |  |  |  |  |  |  |  |  |  |  |  |  |  |  |  |  |  |  |  |  |  |  |  |  |  |  |  |  |  |  |  |  |  |  |  |  |  |  |  |  |  |  |  |  |  |  |  |  |  |  |  |  |  |  |  |  |  |  |  |  |  |  |  |  |  |  |  |  |  |  |  |  |  |  |  |  |  |  |  |  |  |  |  |  |  |  |  |  |  |  |  |  |  |  |  |  |  |  |  |  |  |  |  |  |  |  |  |  |  |  |  |  |  |  |  |  |  |  |  |  |  |  |  |  |  |  |  |  |  |  |  |  |  |  |  |  |  |  |  |  |  |  |  |  |  |  |  |  |  |  |  |  |  |  |  |  |  |  |  |  |  |  |  |  |  |  |  |  |  |  |  |  |  |  |  |  |  |  |  |  |  |  |  |  |  |  |  |  |  |  |  |  |  |  |  |  |  |  |  |  |  |  |  |  |  |  |  |  |  |  |  |  |  |  |  |  |  |  |  |  |  |  |  |  |  |  |  |  |  |  |  |  |  |  |  |  |  |  |  |  |  |  |  |  |  |  |  |  |  |  |  |  |  |  |  |  |  |  |  |  |  |  |  |  |  |  |  |  |  |  |  |  |  |  |  |  |  |  |  |  |  |  |  |  |  |  |  |  |  |  |  |  |  |  |  |  |  |  |  |  |  |  |  |  |  |  |  |  |  |  |  |  |  |  |  |  |  |  |  |  |  |  |  |  |  |  |  |  |  |  |  |  |  |  |  |  |  |  |  |  |  |  |  |  |  |  |  |  |  |  |  |  |  |  |  |  |  |  |  |  |  |  |  |  |  |  |  |  |  |  |  |  |  |  |  |  |  |  |  |  |  |  |  |  |  |  |  |  |  |  |  |  |  |  |  |  |  |  |  |  |  |  |  |  |  |  |  |  |  |  |  |  |  |  |  |  |  |  |  |  |  |  |  |  |  |  |  |  |  |  |  |  |  |  |  |  |  |  |  |  |  |  |  |  |  |  |  |  |  |  |  |  |  |  |  |  |  |  |  |  |  |  |  |  |  |  |  |  |  |  |  |  |  |  |  |  |  |  |  |  |  |  |  |  |  |  |  |  |  |  |  |  |  |  |  |  |  |  |  |  |  |  |  |  |  |  |  |  |  |  |  |  |  |  |  |  |  |  |  |  |  |  |  |  |  |  |  |  |  |  |  |  |  |  |  |  |  |  |  |  |  |  |  |  |  |  |  |  |  |  |  |  |  |  |  |  |  |  |  |  |  |  |  |  |  |  |  |  |  |  |  |  |  |  |  |  |  |  |  |  |  |  |  |  |  |  |  |  |  |  |  |  |  |  |  |  |  |  |  |  |  |  |  |  |  |  |  |  |  |  |  |  |  |  |  |  |  |  |  |  |  |  |  |  |  |  |  |  |  |  |  |  |  |  |  |  |  |  |  |  |  |  |  |  |  |  |  |  |  |  |  |  |  |  |  |  |  |  |  |  |  |  |  |  |  |  |  |  |  |  |  |  |  |  |  |  |  |  |  |  |  |  |  |  |  |  |  |  |  |  |  |  |  |  |  |  |  |  |  |  |  |  |  |  |  |  |  |  |  |  |  |  |  |  |  |  |  |  |  |  |  |  |  |  |  |  |  |  |  |  |  |  |  |  |  |  |  |  |  |  |  |  |  |  |  |  |  |  |  |  |  |  |  |  |  |  |  |  |  |  |  |  |  |  |  |  |  |  |  |  |  |  |  |  |  |  |  |  |  |  |  |  |  |  |  |  |  |  |  |  |  |  |  |  |  |  |  |  |  |  |  |  |  |  |  |  |  |  |  |  |  |  |  |  |  |  |  |  |  |  |  |  |  |  |  |  |  |  |  |  |  |  |  |  |  |  |  |  |  |  |  |  |  |  |  |  |  |  |  |  |  |  |  |  |  |  |  |  |  |  |  |  |  |  |  |  |  |  |  |  |  |  |  |  |  |  |  |  |  |  |  |  |  |  |  |  |  |  |  |  |  |  |  |  |  |  |  |  |  |  |  |  |  |  |  |  |  |  |  |  |  |  |  |  |  |  |  |  |  |  |  |  |  |  |  |  |  |  |  |  |  |  |  |  |  |  |  |  |  |  |  |  |  |  |  |  |  |  |  |  |  |  |  |  |  |  |  |  |  |  |  |  |  |  |  |  |  |  |  |  |  |  |  |  |  |  |  |  |  |  |  |  |  |  |  |  |  |  |  |  |  |  |  |  |  |  |  |  |  |  |  |  |  |  |  |  |  |  |  |  |  |  |  |  |  |  |  |  |  |  |  |  |  |  |  |  |  |  |  |  |  |  |  |  |  |  |  |  |  |  |  |  |  |  |  |  |  |  |  |  |  |  |  |  |  |  |  |  |  |  |  |  |  |  |  |  |  |  |  |  |  |  |  |  |  |  |  |  |  |  |  |  |  |  |  |  |  |  |  |  |  |  |  |  |  |  |  |  |  |  |  |  |  |  |  |  |  |  |  |  |  |  |  |  |  |  |  |  |  |  |  |  |  |  |  |  |  |  |  |  |  |  |  |  |  |  |  |  |  |  |  |  |  |  |  |  |  |  |  |  |  |  |  |  |  |  |  |  |  |  |  |  |  |  |  |  |  |  |  |  |  |  |  |  |  |  |  |  |  |  |  |  |  |  |  |  |  |  |  |  |  |  |  |  |  |  |  |  |  |  |  |  |  |  |  |  |  |  |  |  |  |  |  |  |  |  |  |  |  |  |  |  |  |  |  |  |  |  |  |  |  |  |  |  |  |  |  |  |  |  |  |  |  |  |  |  |  |  |  |  |  |  |  |  |  |  |  |  |  |  |  |  |  |  |  |  |  |  |  |  |  |  |  |  |  |  |  |  |  |  |  |  |  |  |  |  |  |  |  |  |  |  |  |  |  |  |  |  |  |  |  |  |  |  |  |  |  |  |  |  |  |  |  |  |  |  |  |  |  |  |  |  |  |  |  |  |  |  |  |  |  |  |  |  |  |  |  |  |  |  |  |  |  |  |  |  |  |  |  |  |  |  |  |  |  |  |  |  |  |  |  |  |  |  |  |  |  |  |  |  |  |  |  |  |  |  |  |  |  |  |  |  |  |  |  |  |  |  |  |  |  |  |  |  |  |  |  |  |  |  |  |  |  |  |  |  |  |  |  |  |  |  |  |  |  |  |  |  |  |  |  |  |  |  |  |  |  |  |  |  |  |  |  |  |  |  |  |  |  |  |  |  |  |  |  |  |  |  |  |  |  |  |  |  |  |  |  |  |  |  |  |  |  |  |  |  |  |  |  |  |  |  |  |  |  |  |  |  |  |  |  |  |  |  |  |  |  |  |  |  |  |  |  |  |  |  |  |  |  |  |  |  |  |  |  |  |  |  |  |  |  |  |  |  |  |  |  |  |  |  |  |  |  |  |  |  |  |  |  |  |  |  |  |  |  |  |  |  |  |  |  |  |  |  |  |  |  |  |  |  |  |  |  |  |  |  |  |  |  |  |  |  |  |  |  |  |  |  |  |  |  |  |  |  |  |  |  |  |  |  |  |  |  |  |  |  |  |  |  |  |  |  |  |  |  |  |  |  |  |  |  |  |  |  |  |  |  |  |  |  |  |  |  |  |  |  |  |  |  |  |  |  |  |  |  |  |  |  |  |  |  |  |  |  |  |  |  |  |  |  |  |  |  |  |  |  |  |  |  |  |  |  |  |  |  |  |  |  |  |  |  |  |  |  |  |  |  |  |  |  |  |  |  |  |  |  |  |  |  |  |  |  |  |  |  |  |  |  |  |  |  |  |  |  |  |  |  |  |  |  |  |  |  |  |  |  |  |  |  |  |  |  |  |  |  |  |  |  |  |  |  |  |  |  |  |  |  |  |  |  |  |  |  |  |  |  |  |  |  |  |  |  |  |  |  |  |  |  |  |  |  |  |  |  |  |  |  |  |  |  |  |  |  |  |  |  |  |  |  |  |  |  |  |  |  |  |  |  |  |  |  |  |  |  |  |  |  |  |  |  |  |  |  |  |  |  |  |  |  |  |  |  |  |  |  |  |  |  |  |  |  |  |  |  |  |  |  |  |  |  |  |  |  |  |  |  |  |  |  |  |  |  |  |  |  |  |  |  |  |  |  |  |  |  |  |  |  |  |  |  |  |  |  |  |  |  |  |  |  |  |  |  |  |  |  |  |  |  |  |  |  |  |  |  |  |  |  |  |  |  |  |  |  |  |  |  |  |  |  |  |  |  |  |  |  |  |  |  |  |  |  |  |  |  |  |  |  |  |  |  |  |  |  |  |  |  |  |  |  |  |  |  |  |  |  |  |  |  |  |  |  |  |  |  |  |  |  |  |  |  |  |  |  |  |  |  |  |  |  |  |  |  |  |  |  |  |  |  |  |  |  |  |  |  |  |  |  |  |  |  |  |  |  |  |  |  |  |  |  |  |  |  |  |  |  |  |  |  |  |  |  |  |  |  |  |  |  |  |  |  |  |  |  |  |  |  |  |  |  |  |  |  |  |  |  |  |  |  |  |  |  |  |  |  |  |  |  |  |  |  |  |  |  |  |  |  |  |  |  |  |  |  |  |  |  |  |  |  |  |  |  |  |  |  |  |  |  |  |  |  |  |  |  |  |  |  |  |  |  |  |  |  |  |  |  |  |  |  |  |  |  |  |  |  |  |  |  |  |  |  |  |  |  |  |  |  |  |  |  |  |  |  |  |  |  |  |  |  |  |  |  |  |  |  |  |  |  |  |  |  |  |  |  |  |  |  |  |  |  |  |  |  |  |  |  |  |  |  |  |  |  |  |  |  |  |  |  |  |  |  |  |  |  |  |  |  |  |  |  |  |  |  |  |  |  |  |  |  |  |  |  |  |  |  |  |  |  |  |  |  |  |  |  |  |  |  |  |  |  |  |  |  |  |  |  |  |  |  |  |  |  |  |  |  |  |  |  |  |  |  |  |  |  |  |  |  |  |  |  |  |  |  |  |  |  |  |  |  |  |  |  |  |  |  |  |  |  |  |  |  |  |  |  |  |  |  |  |  |  |  |  |  |  |  |  |  |  |  |  |  |  |  |  |  |  |  |  |  |  |  |  |  |  |  |  |  |  |  |  |  |  |  |  |  |  |  |  |  |  |  |  |  |  |  |  |  |  |  |  |  |  |  |  |  |  |  |  |  |  |  |  |  |  |  |  |  |  |  |  |  |  |  |  |  |  |  |  |  |  |  |  |  |  |  |  |  |  |  |  |  |  |  |  |  |  |  |  |  |  |  |  |  |  |  |  |  |  |  |  |  |  |  |  |  |  |  |  |  |  |  |  |  |  |  |  |  |  |  |  |  |  |  |  |  |  |  |  |  |  |  |  |  |  |  |  |  |  |  |  |  |  |  |  |  |  |  |  |  |  |  |  |  |  |  |  |  |  |  |  |  |  |  |  |  |  |  |  |  |  |  |  |  |  |  |  |  |  |  |  |  |  |  |  |  |  |  |  |  |  |  |  |  |  |  |  |  |  |  |  |  |  |  |  |  |  |  |  |  |  |  |  |  |  |  |  |  |  |  |  |  |  |  |  |  |  |  |  |  |  |  |  |  |  |  |  |  |  |  |  |  |  |  |  |  |  |  |  |  |  |  |  |  |  |  |  |  |  |  |  |  |  |  |  |  |  |  |  |  |  |  |  |  |  |  |  |  |  |  |  |  |  |  |  |  |  |  |  |  |  |  |  |  |  |  |  |  |  |  |  |  |  |  |  |  |  |  |  |  |  |  |  |  |  |  |  |  |  |  |  |  |  |  |  |  |  |  |  |  |  |  |  |  |  |  |  |  |  |  |  |  |  |  |  |  |  |  |  |  |  |  |  |  |  |  |  |  |  |  |  |  |  |  |  |  |  |  |  |  |  |  |  |  |  |  |  |  |  |  |  |  |  |  |  |  |  |  |  |  |  |  |  |  |  |  |  |  |  |  |  |  |  |  |  |  |  |  |  |  |  |  |  |  |  |  |  |  |  |  |  |  |  |  |  |  |  |  |  |  |  |  |  |  |  |  |  |  |  |  |  |  |  |  |  |  |  |  |  |  |  |  |  |  |  |  |  |  |  |  |  |  |  |  |  |  |  |  |  |  |  |  |  |  |  |  |  |  |  |  |  |  |  |  |  |  |  |  |  |  |  |  |  |  |  |  |  |  |  |  |  |  |  |  |  |  |  |  |  |  |  |  |  |  |  |  |  |  |  |  |  |  |  |  |  |  |  |  |  |  |  |  |  |  |  |  |  |  |  |  |  |  |  |  |  |  |  |  |  |  |  |  |  |  |  |  |  |  |  |  |  |  |  |  |  |  |  |  |  |  |  |  |  |  |  |  |  |  |  |  |  |  |  |  |  |  |  |  |  |  |  |  |  |  |  |  |  |  |  |  |  |  |  |  |  |  |  |  |  |  |  |  |  |  |  |  |  |  |  |  |  |  |  |  |  |  |  |  |  |  |  |  |  |  |  |  |  |  |  |  |  |  |  |  |  |  |  |  |  |  |  |  |  |  |  |  |  |  |  |  |  |  |  |  |  |  |  |  |  |  |  |  |  |  |  |  |  |  |  |  |  |  |  |  |  |  |  |  |  |  |  |  |  |  |  |  |  |  |  |  |  |  |  |  |  |  |  |  |  |  |  |  |  |  |  |  |  |  |  |  |  |  |  |  |  |  |  |  |  |  |  |  |  |  |  |  |  |  |  |  |  |  |  |  |  |  |  |  |  |  |  |  |  |  |  |  |  |  |  |  |  |  |  |  |  |  |  |  |  |  |  |  |  |  |  |  |  |  |  |  |  |  |  |  |  |  |  |  |  |  |  |  |  |  |  |  |  |  |  |  |  |  |  |  |  |  |  |  |  |  |  |  |  |  |  |  |  |  |  |  |  |  |  |  |  |  |  |  |  |  |  |  |  |  |  |  |  |  |  |  |  |  |  |  |  |  |  |  |  |  |  |  |  |  |  |  |  |  |  |  |  |  |  |  |  |  |  |  |  |  |  |  |  |  |  |  |  |  |  |  |  |  |  |  |  |  |  |  |  |  |  |  |  |  |  |  |  |  |  |  |  |  |  |  |  |  |  |  |  |  |  |  |  |  |  |  |  |  |  |  |  |  |  |  |  |  |  |  |  |  |  |  |  |  |  |  |  |  |  |  |  |  |  |  |  |  |  |  |  |  |  |  |  |  |  |  |  |  |  |  |  |  |  |  |  |  |  |  |  |  |  |  |  |  |  |  |  |  |  |  |  |  |  |  |  |  |  |  |  |  |  |  |  |  |  |  |  |  |  |  |  |  |  |  |  |  |  |  |  |  |  |  |  |  |  |  |  |  |  |  |  |  |  |  |  |  |  |  |  |  |  |  |  |  |  |  |  |  |  |  |  |  |  |  |  |  |  |  |  |  |  |  |  |  |  |  |  |  |  |  |  |  |  |  |  |  |  |  |  |  |  |  |  |  |  |  |  |  |  |  |  |  |  |  |  |  |  |  |  |  |  |  |  |  |  |  |  |  |  |  |  |  |  |  |  |  |  |  |  |  |  |  |  |  |  |  |  |  |  |  |  |  |  |  |  |  |  |  |  |  |  |  |  |  |  |  |  |  |  |  |  |  |  |  |  |  |  |  |  |  |  |  |  |  |  |  |  |  |  |  |  |  |  |  |  |  |  |  |  |  |  |  |  |  |  |  |  |  |  |  |  |  |  |  |  |  |  |  |  |  |  |  |  |  |  |  |  |  |  |  |  |  |  |  |  |  |  |  |  |  |  |  |  |  |  |  |  |  |  |  |  |  |  |  |  |  |  |  |  |  |  |  |  |  |  |  |  |  |  |  |  |  |  |  |  |  |  |  |  |  |  |  |  |  |  |  |  |  |  |  |  |  |  |  |  |  |  |  |  |  |  |  |  |  |  |  |  |  |  |  |  |  |  |  |  |  |  |  |  |  |  |  |  |  |  |  |  |  |  |  |  |  |  |  |  |  |  |  |  |  |  |  |  |  |  |  |  |  |  |  |  |  |  |  |  |  |  |  |  |  |  |  |  |  |  |  |  |  |  |  |  |  |  |  |  |  |  |  |  |  |  |  |  |  |  |  |  |  |  |  |  |  |  |  |  |  |  |  |  |  |  |  |  |  |  |  |  |  |  |  |  |  |  |  |  |  |  |  |  |  |  |  |  |  |  |  |  |  |  |  |  |  |  |  |  |  |  |  |  |  |  |  |  |  |  |  |  |  |  |  |  |  |  |  |  |  |  |  |  |  |  |  |  |  |  |  |  |  |  |  |  |  |  |  |  |  |  |  |  |  |  |  |  |  |  |  |  |  |  |  |  |  |  |  |  |  |  |  |  |  |  |  |  |  |  |  |  |  |  |  |  |  |  |  |  |  |  |  |  |  |  |  |  |  |  |  |  |  |  |  |  |  |  |  |  |  |  |  |  |  |  |  |  |  |  |  |  |  |  |  |  |  |  |  |  |  |  |  |  |  |  |  |  |  |  |  |  |  |  |  |  |  |  |  |  |  |  |  |  |  |  |  |  |  |  |  |  |  |  |  |  |  |  |  |  |  |  |  |  |  |  |  |  |  |  |  |  |  |  |  |  |  |  |  |  |  |  |  |  |  |  |  |  |  |  |  |  |  |  |  |  |  |  |  |  |  |  |  |  |  |  |  |  |  |  |  |  |  |  |  |  |  |  |  |  |  |  |  |  |  |  |  |  |  |  |  |  |  |  |  |  |  |  |  |  |  |  |  |  |  |  |  |  |  |  |  |  |  |  |  |  |  |  |  |  |  |  |  |  |  |  |  |  |  |  |  |  |  |  |  |  |  |  |  |  |  |  |  |  |  |  |  |  |  |  |  |  |  |  |  |  |  |  |  |  |  |  |  |  |  |  |  |  |  |  |  |  |  |  |  |  |  |  |  |  |  |  |  |  |  |  |  |  |  |  |  |  |  |  |  |  |  |  |  |  |  |  |  |  |  |  |  |  |  |  |  |  |  |  |  |  |  |  |  |  |  |  |  |  |  |  |  |  |  |  |  |  |  |  |  |  |  |  |  |  |  |  |  |  |  |  |  |  |  |  |  |  |  |  |  |  |  |  |  |  |  |  |  |  |  |  |  |  |  |  |  |  |  |  |  |  |  |  |  |  |  |  |  |  |  |  |  |  |  |  |  |  |  |  |  |  |  |  |  |  |  |  |  |  |  |  |  |  |  |  |  |  |  |  |  |  |  |  |  |  |  |  |  |  |  |  |  |  |  |  |  |  |  |  |  |  |  |  |  |  |  |  |  |  |  |  |  |  |  |  |  |  |  |  |  |  |  |  |  |  |  |  |  |  |  |  |  |  |  |  |  |  |  |  |  |  |  |  |  |  |  |  |  |  |  |  |  |  |  |  |  |  |  |  |  |  |  |  |  |  |  |  |  |  |  |  |  |  |  |  |  |  |  |  |  |  |  |  |  |  |  |  |  |  |  |  |  |  |  |  |  |  |  |  |  |  |  |  |  |  |  |  |  |  |  |  |  |  |  |  |  |  |  |  |  |  |  |  |  |  |  |  |  |  |  |  |  |  |  |  |  |  |  |  |  |  |  |  |  |  |  |  |  |  |  |  |  |  |  |  |  |  |  |  |  |  |  |  |  |  |  |  |  |  |  |  |  |  |  |  |  |  |  |  |  |  |  |  |  |  |  |  |  |  |  |  |  |  |  |  |  |  |  |  |  |  |  |  |  |  |  |  |  |  |  |  |  |  |  |  |  |  |  |  |  |  |  |  |  |  |  |  |  |  |  |  |  |  |  |  |  |  |  |  |  |  |  |  |  |  |  |  |  |  |  |  |  |  |  |  |  |  |  |  |  |  |  |  |  |  |  |  |  |  |  |  |  |  |  |  |  |  |  |  |  |  |  |  |  |  |  |  |  |  |  |  |  |  |  |  |  |  |  |  |  |  |  |  |  |  |  |  |  |  |  |  |  |  |  |  |  |  |  |  |  |  |  |  |  |  |  |  |  |  |  |  |  |  |  |  |  |  |  |  |  |  |  |  |  |  |  |  |  |  |  |  |  |  |  |  |  |  |  |  |  |  |  |  |  |  |  |  |  |  |  |  |  |  |  |  |  |  |  |  |  |  |  |  |  |  |  |  |  |  |  |  |  |  |  |  |  |  |  |  |  |  |  |  |  |  |  |  |  |  |  |  |  |  |  |  |  |  |  |  |  |  |  |  |  |  |  |  |  |  |  |  |  |  |  |  |  |  |  |  |  |  |  |  |  |  |  |  |  |  |  |  |  |  |  |  |  |  |  |  |  |  |  |  |  |  |  |  |  |  |  |  |  |  |  |  |  |  |  |  |  |  |  |  |  |  |  |  |  |  |  |  |  |  |  |  |  |  |  |  |  |  |  |  |  |  |  |  |  |  |  |  |  |  |  |  |  |  |  |  |  |  |  |  |  |  |  |  |  |  |  |  |  |  |  |  |  |  |  |  |  |  |  |  |  |  |  |  |  |  |  |  |  |  |  |  |  |  |  |  |  |  |  |  |  |  |  |  |  |  |  |  |  |  |  |  |  |  |  |  |  |  |  |  |  |  |  |  |  |  |  |  |  |  |  |  |  |  |  |  |  |  |  |  |  |  |  |  |  |  |  |  |  |  |  |  |  |  |  |  |  |  |  |  |  |  |  |  |  |  |  |  |  |  |  |  |  |  |  |  |  |  |  |  |  |  |  |  |  |  |  |  |  |  |  |  |  |  |  |  |  |  |  |  |  |  |  |  |  |  |  |  |  |  |  |  |  |  |  |  |  |  |  |  |  |  |  |  |  |  |  |  |  |  |  |  |  |  |  |  |  |  |  |  |  |  |  |  |  |  |  |  |  |  |  |  |  |  |  |  |  |  |  |  |  |  |  |  |  |  |  |  |  |  |  |  |  |  |  |  |  |  |  |  |  |  |  |  |  |  |  |  |  |  |  |  |  |  |  |  |  |  |  |  |  |  |  |  |  |  |  |  |  |  |  |  |  |  |  |  |  |  |  |  |  |  |  |  |  |  |  |  |  |  |  |  |  |  |  |  |  |  |  |  |  |  |  |  |  |  |  |  |  |  |  |  |  |  |  |  |  |  |  |  |  |  |  |  |  |  |  |  |  |  |  |  |  |  |  |  |  |  |  |  |  |  |  |  |  |  |  |  |  |  |  |  |  |  |  |  |  |  |  |  |  |  |  |  |  |  |  |  |  |  |  |  |  |  |  |  |  |  |  |  |  |  |  |  |  |  |  |  |  |  |  |  |  |  |  |  |  |  |  |  |  |  |  |  |  |  |  |  |  |  |  |  |  |  |  |  |  |  |  |  |  |  |  |  |  |  |  |  |  |  |  |  |  |  |  |  |  |  |  |  |  |  |  |  |  |  |  |  |  |  |  |  |  |  |  |  |  |  |  |  |  |  |  |  |  |  |  |  |  |  |  |  |  |  |  |  |  |  |  |  |  |  |  |  |  |  |  |  |  |  |  |  |  |  |  |  |  |  |  |  |  |  |  |  |  |  |  |  |  |  |  |  |  |  |  |  |  |  |  |  |  |  |  |  |  |  |  |  |  |  |  |  |  |  |  |  |  |  |  |  |  |  |  |  |  |  |  |  |  |  |  |  |  |  |  |  |  |  |  |  |  |  |  |  |  |  |  |  |  |  |  |  |  |  |  |  |  |  |  |  |  |  |  |  |  |  |  |  |  |  |  |  |  |  |  |  |  |  |  |  |  |  |  |  |  |  |  |  |  |  |  |  |  |  |  |  |  |  |  |  |  |  |  |  |  |  |  |  |  |  |  |  |  |  |  |  |  |  |  |  |  |  |  |  |  |  |  |  |  |  |  |  |  |  |  |  |  |  |  |  |  |  |  |  |  |  |  |  |  |  |  |  |  |  |  |  |  |  |  |  |  |  |  |  |  |  |  |  |  |  |  |  |  |  |  |  |  |  |  |  |  |  |  |  |  |  |  |  |  |  |  |  |  |  |  |  |  |  |  |  |  |  |  |  |  |  |  |  |  |  |  |  |  |  |  |  |  |  |  |  |  |  |  |  |  |  |  |  |  |  |  |  |  |  |  |  |  |  |  |  |  |  |  |  |  |  |  |  |  |  |  |  |  |  |  |  |  |  |  |  |  |  |  |  |  |  |  |  |  |  |  |  |  |  |  |  |  |  |  |  |  |  |  |  |  |  |  |  |  |  |  |  |  |  |  |  |  |  |  |  |  |  |  |  |  |  |  |  |  |  |  |  |  |  |  |  |  |  |  |  |  |  |  |  |  |  |  |  |  |  |  |  |  |  |  |  |  |  |  |  |  |  |  |  |  |  |  |  |  |  |  |  |  |  |  |  |  |  |  |  |  |  |  |  |  |  |  |  |  |  |  |  |  |  |  |  |  |  |  |  |  |  |  |  |  |  |  |  |  |  |  |  |  |  |  |  |  |  |  |  |  |  |  |  |  |  |  |  |  |  |  |  |  |  |  |  |  |  |  |  |  |  |  |  |  |  |  |  |  |  |  |  |  |  |  |  |  |  |  |  |  |  |  |  |  |  |  |  |  |  |  |  |  |  |  |  |  |  |  |  |  |  |  |  |  |  |  |  |  |  |  |  |  |  |  |  |  |  |  |  |  |  |  |  |  |  |  |  |  |  |  |  |  |  |  |  |  |  |  |  |  |  |  |  |  |  |  |  |  |  |  |  |  |  |  |  |  |  |  |  |  |  |  |  |  |  |  |  |  |  |  |  |  |  |  |  |  |  |  |  |  |  |  |  |  |  |  |  |  |  |  |  |  |  |  |  |  |  |  |  |  |  |  |  |  |  |  |  |  |  |  |  |  |  |  |  |  |  |  |  |  |  |  |  |  |  |  |  |  |  |  |  |  |  |  |  |  |  |  |  |  |  |  |  |  |  |  |  |  |  |  |  |  |  |  |  |  |  |  |  |  |  |  |  |  |  |  |  |  |  |  |  |  |  |  |  |  |  |  |  |  |  |  |  |  |  |  |  |  |  |  |  |  |  |  |  |  |  |  |  |  |  |  |  |  |  |  |  |  |  |  |  |  |  |  |  |  |  |  |  |  |  |  |  |  |  |  |  |  |  |  |  |  |  |  |  |  |  |  |  |  |  |  |  |  |  |  |  |  |  |  |  |  |  |  |  |  |  |  |  |  |  |  |  |  |  |  |  |  |  |  |  |  |  |  |  |  |  |  |  |  |  |  |  |  |  |  |  |  |  |  |  |  |  |  |  |  |  |  |  |  |  |  |  |  |  |  |  |  |  |  |  |  |  |  |  |  |  |  |  |  |  |  |  |  |  |  |  |  |  |  |  |  |  |  |  |  |  |  |  |  |  |  |  |  |  |  |  |  |  |  |  |  |  |  |  |  |  |  |  |  |  |  |  |  |  |  |  |  |  |  |  |  |  |  |  |  |  |  |  |  |  |  |  |  |  |  |  |  |  |  |  |  |  |  |  |  |  |  |  |  |  |  |  |  |  |  |  |  |  |  |  |  |  |  |  |  |  |  |  |  |  |  |  |  |  |  |  |  |  |  |  |  |  |  |  |  |  |  |  |  |  |  |  |  |  |  |  |  |  |  |  |  |  |  |  |  |  |  |  |  |  |  |  |  |  |  |  |  |  |  |  |  |  |  |  |  |  |  |  |  |  |  |  |  |  |  |  |  |  |  |  |  |  |  |  |  |  |  |  |  |  |  |  |  |  |  |  |  |  |  |  |  |  |  |  |  |  |  |  |  |  |  |  |  |  |  |  |  |  |  |  |  |  |  |  |  |  |  |  |  |  |  |  |  |  |  |  |  |  |  |  |  |  |  |  |  |  |  |  |  |  |  |  |  |  |  |  |  |  |  |  |  |  |  |  |  |  |  |  |  |  |  |  |  |  |  |  |  |  |  |  |  |  |  |  |  |  |  |  |  |  |  |  |  |  |  |  |  |  |  |  |  |  |  |  |  |  |  |  |  |  |  |  |  |  |  |  |  |  |  |  |  |  |  |  |  |  |  |  |  |  |  |  |  |  |  |  |  |  |  |  |  |  |  |  |  |  |  |  |  |  |  |  |  |  |  |  |  |  |  |  |  |  |  |  |  |  |  |  |  |  |  |  |  |  |  |  |  |  |  |  |  |  |  |  |  |  |  |  |  |  |  |  |  |  |  |  |  |  |  |  |  |  |  |  |  |  |  |  |  |  |  |  |  |  |  |  |  |  |  |  |  |  |  |  |  |  |  |  |  |  |  |  |  |  |  |  |  |  |  |  |  |  |  |  |  |  |  |  |  |  |  |  |  |  |  |  |  |  |  |  |  |  |  |  |  |  |  |  |  |  |  |  |  |  |  |  |  |  |  |  |  |  |  |  |  |  |  |  |  |  |  |  |  |  |  |  |  |  |  |  |  |  |  |  |  |  |  |  |  |  |  |  |  |  |  |  |  |  |  |  |  |  |  |  |  |  |  |  |  |  |  |  |  |  |  |  |  |  |  |  |  |  |  |  |  |  |  |  |  |  |  |  |  |  |  |  |  |  |  |  |  |  |  |  |  |  |  |  |  |  |  |  |  |  |  |  |  |  |  |  |  |  |  |  |  |  |  |  |  |  |  |  |  |  |  |  |  |  |  |  |  |  |  |  |  |  |  |  |  |  |  |  |  |  |  |  |  |  |  |  |  |  |  |  |  |  |  |  |  |  |  |  |  |  |  |  |  |  |  |  |  |  |  |  |  |  |  |  |  |  |  |  |  |  |  |  |  |  |  |  |  |  |  |  |  |  |  |  |  |  |  |  |  |  |  |  |  |  |  |  |  |  |  |  |  |  |  |  |  |  |  |  |  |  |  |  |  |  |  |  |  |  |  |  |  |  |  |  |  |  |  |  |  |  |  |  |  |  |  |  |  |  |  |  |  |  |  |  |  |  |  |  |  |  |  |  |  |  |  |  |  |  |  |  |  |  |  |  |  |  |  |  |  |  |  |  |  |  |  |  |  |  |  |  |  |  |  |  |  |  |  |  |  |  |  |  |  |  |  |  |  |  |  |  |  |  |  |  |  |  |  |  |  |  |  |  |  |  |  |  |  |  |  |  |  |  |  |  |  |  |  |  |  |  |  |  |  |  |  |  |  |  |  |  |  |  |  |  |  |  |  |  |  |  |  |  |  |  |  |  |  |  |  |  |  |  |  |  |  |  |  |  |  |  |  |  |  |  |  |  |  |  |  |  |  |  |  |  |  |  |  |  |  |  |  |  |  |  |  |  |  |  |  |  |  |  |  |  |  |  |  |  |  |  |  |  |  |  |  |  |  |  |  |  |  |  |  |  |  |  |  |  |  |  |  |  |  |  |  |  |  |  |  |  |  |  |  |  |  |  |  |  |  |  |  |  |  |  |  |  |  |  |  |  |  |  |  |  |  |  |  |  |  |  |  |  |  |  |  |  |  |  |  |  |  |  |  |  |  |  |  |  |  |  |  |  |  |  |  |  |  |  |  |  |  |  |  |  |  |  |  |  |  |  |  |  |  |  |  |  |  |  |  |  |  |  |  |  |  |  |  |  |  |  |  |  |  |  |  |  |  |  |  |  |  |  |  |  |  |  |  |  |  |  |  |  |  |  |  |  |  |  |  |  |  |  |  |  |  |  |  |  |  |  |  |  |  |  |  |  |  |  |  |  |  |  |  |  |  |  |  |  |  |  |  |  |  |  |  |  |  |  |  |  |  |  |  |  |  |  |  |  |  |  |  |  |  |  |  |  |  |  |  |  |  |  |  |  |  |  |  |  |  |  |  |  |  |  |  |  |  |  |  |  |  |  |  |  |  |  |  |  |  |  |  |  |  |  |  |  |  |  |  |  |  |  |  |  |  |  |  |  |  |  |  |  |  |  |  |  |  |  |  |  |  |  |  |  |  |  |  |  |  |  |  |  |  |  |  |  |  |  |  |  |  |  |  |  |  |  |  |  |  |  |  |  |  |  |  |  |  |  |  |  |  |  |  |  |  |  |  |  |  |  |  |  |  |  |  |  |  |  |  |  |  |  |  |  |  |  |  |  |  |  |  |  |  |  |  |  |  |  |  |  |  |  |  |  |  |  |  |  |  |  |  |  |  |  |  |  |  |  |  |  |  |  |  |  |  |  |  |  |  |  |  |  |  |  |  |  |  |  |  |  |  |  |  |  |  |  |  |  |  |  |  |  |  |  |  |  |  |  |  |  |  |  |  |  |  |  |  |  |  |  |  |  |  |  |  |  |  |  |  |  |  |  |  |  |  |  |  |  |  |  |  |  |  |  |  |  |  |  |  |  |  |  |  |  |  |  |  |  |  |  |  |  |  |  |  |  |  |  |  |  |  |  |  |  |  |  |  |  |  |  |  |  |  |  |  |  |  |  |  |  |  |  |  |  |  |  |  |  |  |  |  |  |  |  |  |  |  |  |  |  |  |  |  |  |  |  |  |  |  |  |  |  |  |  |  |  |  |  |  |  |  |  |  |  |  |  |  |  |  |  |  |  |  |  |  |  |  |  |  |  |  |  |  |  |  |  |  |  |  |  |  |  |  |  |  |  |  |  |  |  |  |  |  |  |  |  |  |  |  |  |  |  |  |  |  |  |  |  |  |  |  |  |  |  |  |  |  |  |  |  |  |  |  |  |  |  |  |  |  |  |  |  |  |  |  |  |  |  |  |  |  |  |  |  |  |  |  |  |  |  |  |  |  |  |  |  |  |  |  |  |  |  |  |  |  |  |  |  |  |  |  |  |  |  |  |  |  |  |  |  |  |  |  |  |  |  |  |  |  |  |  |  |  |  |  |  |  |  |  |  |  |  |  |  |  |  |  |  |  |  |  |  |  |  |  |  |  |  |  |  |  |  |  |  |  |  |  |  |  |  |  |  |  |  |  |  |  |  |  |  |  |  |  |  |  |  |  |  |  |  |  |  |  |  |  |  |  |  |  |  |  |  |  |  |  |  |  |  |  |  |  |  |  |  |  |  |  |  |  |  |  |  |  |  |  |  |  |  |  |  |  |  |  |  |  |  |  |  |  |  |  |  |  |  |  |  |  |  |  |  |  |  |  |  |  |  |  |  |  |  |  |  |  |  |  |  |  |  |  |  |  |  |  |  |  |  |  |  |  |  |  |  |  |  |  |  |  |  |  |  |  |  |  |  |  |  |  |  |  |  |  |  |  |  |  |  |  |  |  |  |  |  |  |  |  |  |  |  |  |  |  |  |  |  |  |  |  |  |  |  |  |  |  |  |  |  |  |  |  |  |  |  |  |  |  |  |  |  |  |  |  |  |  |  |  |  |  |  |  |  |  |  |  |  |  |  |  |  |  |  |  |  |  |  |  |  |  |  |  |  |  |  |  |  |  |  |  |  |  |  |  |  |  |  |  |  |  |  |  |  |  |  |  |  |  |  |  |  |  |  |  |  |  |  |  |  |  |  |  |  |  |  |  |  |  |  |  |  |  |  |  |  |  |  |  |  |  |  |  |  |  |  |  |  |  |  |  |  |  |  |  |  |  |  |  |  |  |  |  |  |  |  |  |  |  |  |  |  |  |  |  |  |  |  |  |  |  |  |  |  |  |  |  |  |  |  |  |  |  |  |  |  |  |  |  |  |  |  |  |  |  |  |  |  |  |  |  |  |  |  |  |  |  |  |  |  |  |  |  |  |  |  |  |  |  |  |  |  |  |  |  |  |  |  |  |  |  |  |  |  |  |  |  |  |  |  |  |  |  |  |  |  |  |  |  |  |  |  |  |  |  |  |  |  |  |  |  |  |  |  |  |  |  |  |  |  |  |  |  |  |  |  |  |  |  |  |  |  |  |  |  |  |  |  |  |  |  |  |  |  |  |  |  |  |  |  |  |  |  |  |  |  |  |  |  |  |  |  |  |  |  |  |  |  |  |  |  |  |  |  |  |  |  |  |  |  |  |  |  |  |  |  |  |  |  |  |  |  |  |  |  |  |  |  |  |  |  |  |  |  |  |  |  |  |  |  |  |  |  |  |  |  |  |  |  |  |  |  |  |  |  |  |  |  |  |  |  |  |  |  |  |  |  |  |  |  |  |  |  |  |  |  |  |  |  |  |  |  |  |  |  |  |  |  |  |  |  |  |  |  |  |  |  |  |  |  |  |  |  |  |  |  |  |  |  |  |  |  |  |  |  |  |  |  |  |  |  |  |  |  |  |  |  |  |  |  |  |  |  |  |  |  |  |  |  |  |  |  |  |  |  |  |  |  |  |  |  |  |  |  |  |  |  |  |  |  |  |  |  |  |  |  |  |  |  |  |  |  |  |  |  |  |  |  |  |  |  |  |  |  |  |  |  |  |  |  |  |  |  |  |  |  |  |  |  |  |  |  |  |  |  |  |  |  |  |  |  |  |  |  |  |  |  |  |  |  |  |  |  |  |  |  |  |  |  |  |  |  |  |  |  |  |  |  |  |  |  |  |  |  |  |  |  |  |  |  |  |  |  |  |  |  |  |  |  |  |  |  |  |  |  |  |  |  |  |  |  |  |  |  |  |  |  |  |  |  |  |  |  |  |  |  |  |  |  |  |  |  |  |  |  |  |  |  |  |  |  |  |  |  |  |  |  |  |  |  |  |  |  |  |  |  |  |  |  |  |  |  |  |  |  |  |  |  |  |  |  |  |  |  |  |  |  |  |  |  |  |  |  |  |  |  |  |  |  |  |  |  |  |  |  |  |  |  |  |  |  |  |  |  |  |  |  |  |  |  |  |  |  |  |  |  |  |  |  |  |  |  |  |  |  |  |  |  |  |  |  |  |  |  |  |  |  |  |  |  |  |  |  |  |  |  |  |  |  |  |  |  |  |  |  |  |  |  |  |  |  |  |  |  |  |  |  |  |  |  |  |  |  |  |  |  |  |  |  |  |  |  |  |  |  |  |  |  |  |  |  |  |  |  |  |  |  |  |  |  |  |  |  |  |  |  |  |  |  |  |  |  |  |  |  |  |  |  |  |  |  |  |  |  |  |  |  |  |  |  |  |  |  |  |  |  |  |  |  |  |  |  |  |  |  |  |  |  |  |  |  |  |  |  |  |  |  |  |  |  |  |  |  |  |  |  |  |  |  |  |  |  |  |  |  |  |  |  |  |  |  |  |  |  |  |  |  |  |  |  |  |  |  |  |  |  |  |  |  |  |  |  |  |  |  |  |  |  |  |  |  |  |  |  |  |  |  |  |  |  |  |  |  |  |  |  |  |  |  |  |  |  |  |  |  |  |  |  |  |  |  |  |  |  |  |  |  |  |  |  |  |  |  |  |  |  |  |  |  |  |  |  |  |  |  |  |  |  |  |  |  |  |  |  |  |  |  |  |  |  |  |  |  |  |  |  |  |  |  |  |  |  |  |  |  |  |  |  |  |  |  |  |  |  |  |  |  |  |  |  |  |  |  |  |  |  |  |  |  |  |  |  |  |  |  |  |  |  |  |  |  |  |  |  |  |  |  |  |  |  |  |  |  |  |  |  |  |  |  |  |  |  |  |  |  |  |  |  |  |  |  |  |  |  |  |  |  |  |  |  |  |  |  |  |  |  |  |  |  |  |  |  |  |  |  |  |  |  |  |  |  |  |  |  |  |  |  |  |  |  |  |  |  |  |  |  |  |  |  |  |  |  |  |  |  |  |  |  |  |  |  |  |  |  |  |  |  |  |  |  |  |  |  |  |  |  |  |  |  |  |  |  |  |  |  |  |  |  |  |  |  |  |  |  |  |  |  |  |  |  |  |  |  |  |  |  |  |  |  |  |  |  |  |  |  |  |  |  |  |  |  |  |  |  |  |  |  |  |  |  |  |  |  |  |  |  |  |  |  |  |  |  |  |  |  |  |  |  |  |  |  |  |  |  |  |  |  |  |  |  |  |  |  |  |  |  |  |  |  |  |  |  |  |  |  |  |  |  |  |  |  |  |  |  |  |  |  |  |  |  |  |  |  |  |  |  |  |  |  |  |  |  |  |  |  |  |  |  |  |  |  |  |  |  |  |  |  |  |  |  |  |  |  |  |  |  |  |  |  |  |  |  |  |  |  |  |  |  |  |  |  |  |  |  |  |  |  |  |  |  |  |  |  |  |  |  |  |  |  |  |  |  |  |  |  |  |  |  |  |  |  |  |  |  |  |  |  |  |  |  |  |  |  |  |  |  |  |  |  |  |  |  |  |  |  |  |  |  |  |  |  |  |  |  |  |  |  |  |  |  |  |  |  |  |  |  |  |  |  |  |  |  |  |  |  |  |  |  |  |  |  |  |  |  |  |  |  |  |  |  |  |  |  |  |  |  |  |  |  |  |  |  |  |  |  |  |  |  |  |  |  |  |  |  |  |  |  |  |  |  |  |  |  |  |  |  |  |  |  |  |  |  |  |  |  |  |  |  |  |  |  |  |  |  |  |  |  |  |  |  |  |  |  |  |  |  |  |  |  |  |  |  |  |  |  |  |  |  |  |  |  |  |  |  |  |  |  |  |  |  |  |  |  |  |  |  |  |  |  |  |  |  |  |  |  |  |  |  |  |  |  |  |  |  |  |  |  |  |  |  |  |  |  |  |  |  |  |  |  |  |  |  |  |  |  |  |  |  |  |  |  |  |  |  |  |  |  |  |  |  |  |  |  |  |  |  |  |  |  |  |  |  |  |  |  |  |  |  |  |  |  |  |  |  |  |  |  |  |  |  |  |  |  |  |  |  |  |  |  |  |  |  |  |  |  |  |  |  |  |  |  |  |  |  |  |  |  |  |  |  |  |  |  |  |  |  |  |  |  |  |  |  |  |  |  |  |  |  |  |  |  |  |  |  |  |  |  |  |  |  |  |  |  |  |  |  |  |  |  |  |  |  |  |  |  |  |  |  |  |  |  |  |  |  |  |  |  |  |  |  |  |  |  |  |  |  |  |  |  |  |  |  |  |  |  |  |  |  |  |  |  |  |  |  |  |  |  |  |  |  |  |  |  |  |  |  |  |  |  |  |  |  |  |  |  |  |  |  |  |  |  |  |  |  |  |  |  |  |  |  |  |  |  |  |  |  |  |  |  |  |  |  |  |  |  |  |  |  |  |  |  |  |  |  |  |  |  |  |  |  |  |  |  |  |  |  |  |  |  |  |  |  |  |  |  |  |  |  |  |  |  |  |  |  |  |  |  |  |  |  |  |  |  |  |  |  |  |  |  |  |  |  |  |  |  |  |  |  |  |  |  |  |  |  |  |  |  |  |  |  |  |  |  |  |  |  |  |  |  |  |  |  |  |  |  |  |  |  |  |  |  |  |  |  |  |  |  |  |  |  |  |  |  |  |  |  |  |  |  |  |  |  |  |  |  |  |  |  |  |  |  |  |  |  |  |  |  |  |  |  |  |  |  |  |  |  |  |  |  |  |  |  |  |  |  |  |  |  |  |  |  |  |  |  |  |  |  |  |  |  |  |  |  |  |  |  |  |  |  |  |  |  |  |  |  |  |  |  |  |  |  |  |  |  |  |  |  |  |  |  |  |  |  |  |  |  |  |  |  |  |  |  |  |  |  |  |  |  |  |  |  |  |  |  |  |  |  |  |  |  |  |  |  |  |  |  |  |  |  |  |  |  |  |  |  |  |  |  |  |  |  |  |  |  |  |  |  |  |  |  |  |  |  |  |  |  |  |  |  |  |  |  |  |  |  |  |  |  |  |  |  |  |  |  |  |  |  |  |  |  |  |  |  |  |  |  |  |  |  |  |  |  |  |  |  |  |  |  |  |  |  |  |  |  |  |  |  |  |  |  |  |  |  |  |  |  |  |  |  |  |  |  |  |  |  |  |  |  |  |  |  |  |  |  |  |  |  |  |  |  |  |  |  |  |  |  |  |  |  |  |  |  |  |  |  |  |  |  |  |  |  |  |  |  |  |  |  |  |  |  |  |  |  |  |  |  |  |  |  |  |  |  |  |  |  |  |  |  |  |  |  |  |  |  |  |  |  |  |  |  |  |  |  |  |  |  |  |  |  |  |  |  |  |  |  |  |  |  |  |  |  |  |  |  |  |  |  |  |  |  |  |  |  |  |  |  |  |  |  |  |  |  |  |  |  |  |  |  |  |  |  |  |  |  |  |  |  |  |  |  |  |  |  |  |  |  |  |  |  |  |  |  |  |  |  |  |  |  |  |  |  |  |  |  |  |  |  |  |  |  |  |  |  |  |  |  |  |  |  |  |  |  |  |  |  |  |  |  |  |  |  |  |  |  |  |  |  |  |  |  |  |  |  |  |  |  |  |  |  |  |  |  |  |  |  |  |  |  |  |  |  |  |  |  |  |  |  |  |  |  |  |  |  |  |  |  |  |  |  |  |  |  |  |  |  |  |  |  |  |  |  |  |  |  |  |  |  |  |  |  |  |  |  |  |  |  |  |  |  |  |  |  |  |  |  |  |  |  |  |  |  |  |  |  |  |  |  |  |  |  |  |  |  |  |  |  |  |  |  |  |  |  |  |  |  |  |  |  |  |  |  |  |  |  |  |  |  |  |  |  |  |  |  |  |  |  |  |  |  |  |  |  |  |  |  |  |  |  |  |  |  |  |  |  |  |  |  |  |  |  |  |  |  |  |  |  |  |  |  |  |  |  |  |  |  |  |  |  |  |  |  |  |  |  |  |  |  |  |  |  |  |  |  |  |  |  |  |  |  |  |  |  |  |  |  |  |  |  |  |  |  |  |  |  |  |  |  |  |  |  |  |  |  |  |  |  |  |  |  |  |  |  |  |  |  |  |  |  |  |  |  |  |  |  |  |  |  |  |  |  |  |  |  |  |  |  |  |  |  |  |  |  |  |  |  |  |  |  |  |  |  |  |  |  |  |  |  |  |  |  |  |  |  |  |  |  |  |  |  |  |  |  |  |  |  |  |  |  |  |  |  |  |  |  |  |  |  |  |  |  |  |  |  |  |  |  |  |  |  |  |  |  |  |  |  |  |  |  |  |  |  |  |  |  |  |  |  |  |  |  |  |  |  |  |  |  |  |  |  |  |  |  |  |  |  |  |  |  |  |  |  |  |  |  |  |  |  |  |  |  |  |  |  |  |  |  |  |  |  |  |  |  |  |  |  |  |  |  |  |  |  |  |  |  |  |  |  |  |  |  |  |  |  |  |  |  |  |  |  |  |  |  |  |  |  |  |  |  |  |  |  |  |  |  |  |  |  |  |  |  |  |  |  |  |  |  |  |  |  |  |  |  |  |  |  |  |  |  |  |  |  |  |  |  |  |  |  |  |  |  |  |  |  |  |  |  |  |  |  |  |  |  |  |  |  |  |  |  |  |  |  |  |  |  |  |  |  |  |  |  |  |  |  |  |  |  |  |  |  |  |  |  |  |  |  |  |  |  |  |  |  |  |  |  |  |  |  |  |  |  |  |  |  |  |  |  |  |  |  |  |  |  |  |  |  |  |  |  |  |  |  |  |  |  |  |  |  |  |  |  |  |  |  |  |  |  |  |  |  |  |  |  |  |  |  |  |  |  |  |  |  |  |  |  |  |  |  |  |  |  |  |  |  |  |  |  |  |  |  |  |  |  |  |  |  |  |  |  |  |  |  |  |  |  |  |  |  |  |  |  |  |  |  |  |  |  |  |  |  |  |  |  |  |  |  |  |  |  |  |  |  |  |  |  |  |  |  |  |  |  |  |  |  |  |  |  |  |  |  |  |  |  |  |  |  |  |  |  |  |  |  |  |  |  |  |  |  |  |  |  |  |  |  |  |  |  |  |  |  |  |  |  |  |  |  |  |  |  |  |  |  |  |  |  |  |  |  |  |  |  |  |  |  |  |  |  |  |  |  |  |  |  |  |  |  |  |  |  |  |  |  |  |  |  |  |  |  |  |  |  | | --- | --- | --- | --- | --- | --- | --- | --- | --- | --- | --- | --- | --- | --- | --- | --- | --- | --- | --- | --- | --- | --- | --- | --- | --- | --- | --- | --- | --- | --- | --- | --- | --- | --- | --- | --- | --- | --- | --- | --- | --- | --- | --- | --- | --- | --- | --- | --- | --- | --- | --- | --- | --- | --- | --- | --- | --- | --- | --- | --- | --- | --- | --- | --- | --- | --- | --- | --- | --- | --- | --- | --- | --- | --- | --- | --- | --- | --- | --- | --- | --- | --- | --- | --- | --- | --- | --- | --- | --- | --- | --- | --- | --- | --- | --- | --- | --- | --- | --- | --- | --- | --- | --- | --- | --- | --- | --- | --- | --- | --- | --- | --- | --- | --- | --- | --- | --- | --- | --- | --- | --- | --- | --- | --- | --- | --- | --- | --- | --- | --- | --- | --- | --- | --- | --- | --- | --- | --- | --- | --- | --- | --- | --- | --- | --- | --- | --- | --- | --- | --- | --- | --- | --- | --- | --- | --- | --- | --- | --- | --- | --- | --- | --- | --- | --- | --- | --- | --- | --- | --- | --- | --- | --- | --- | --- | --- | --- | --- | --- | --- | --- | --- | --- | --- | --- | --- | --- | --- | --- | --- | --- | --- | --- | --- | --- | --- | --- | --- | --- | --- | --- | --- | --- | --- | --- | --- | --- | --- | --- | --- | --- | --- | --- | --- | --- | --- | --- | --- | --- | --- | --- | --- | --- | --- | --- | --- | --- | --- | --- | --- | --- | --- | --- | --- | --- | --- | --- | --- | --- | --- | --- | --- | --- | --- | --- | --- | --- | --- | --- | --- | --- | --- | --- | --- | --- | --- | --- | --- | --- | --- | --- | --- | --- | --- | --- | --- | --- | --- | --- | --- | --- | --- | --- | --- | --- | --- | --- | --- | --- | --- | --- | --- | --- | --- | --- | --- | --- | --- | --- | --- | --- | --- | --- | --- | --- | --- | --- | --- | --- | --- | --- | --- | --- | --- | --- | --- | --- | --- | --- | --- | --- | --- | --- | --- | --- | --- | --- | --- | --- | --- | --- | --- | --- | --- | --- | --- | --- | --- | --- | --- | --- | --- | --- | --- | --- | --- | --- | --- | --- | --- | --- | --- | --- | --- | --- | --- | --- | --- | --- | --- | --- | --- | --- | --- | --- | --- | --- | --- | --- | --- | --- | --- | --- | --- | --- | --- | --- | --- | --- | --- | --- | --- | --- | --- | --- | --- | --- | --- | --- | --- | --- | --- | --- | --- | --- | --- | --- | --- | --- | --- | --- | --- | --- | --- | --- | --- | --- | --- | --- | --- | --- | --- | --- | --- | --- | --- | --- | --- | --- | --- | --- | --- | --- | --- | --- | --- | --- | --- | --- | --- | --- | --- | --- | --- | --- | --- | --- | --- | --- | --- | --- | --- | --- | --- | --- | --- | --- | --- | --- | --- | --- | --- | --- | --- | --- | --- | --- | --- | --- | --- | --- | --- | --- | --- | --- | --- | --- | --- | --- | --- | --- | --- | --- | --- | --- | --- | --- | --- | --- | --- | --- | --- | --- | --- | --- | --- | --- | --- | --- | --- | --- | --- | --- | --- | --- | --- | --- | --- | --- | --- | --- | --- | --- | --- | --- | --- | --- | --- | --- | --- | --- | --- | --- | --- | --- | --- | --- | --- | --- | --- | --- | --- | --- | --- | --- | --- | --- | --- | --- | --- | --- | --- | --- | --- | --- | --- | --- | --- | --- | --- | --- | --- | --- | --- | --- | --- | --- | --- | --- | --- | --- | --- | --- | --- | --- | --- | --- | --- | --- | --- | --- | --- | --- | --- | --- | --- | --- | --- | --- | --- | --- | --- | --- | --- | --- | --- | --- | --- | --- | --- | --- | --- | --- | --- | --- | --- | --- | --- | --- | --- | --- | --- | --- | --- | --- | --- | --- | --- | --- | --- | --- | --- | --- | --- | --- | --- | --- | --- | --- | --- | --- | --- | --- | --- | --- | --- | --- | --- | --- | --- | --- | --- | --- | --- | --- | --- | --- | --- | --- | --- | --- | --- | --- | --- | --- | --- | --- | --- | --- | --- | --- | --- | --- | --- | --- | --- | --- | --- | --- | --- | --- | --- | --- | --- | --- | --- | --- | --- | --- | --- | --- | --- | --- | --- | --- | --- | --- | --- | --- | --- | --- | --- | --- | --- | --- | --- | --- | --- | --- | --- | --- | --- | --- | --- | --- | --- | --- | --- | --- | --- | --- | --- | --- | --- | --- | --- | --- | --- | --- | --- | --- | --- | --- | --- | --- | --- | --- | --- | --- | --- | --- | --- | --- | --- | --- | --- | --- | --- | --- | --- | --- | --- | --- | --- | --- | --- | --- | --- | --- | --- | --- | --- | --- | --- | --- | --- | --- | --- | --- | --- | --- | --- | --- | --- | --- | --- | --- | --- | --- | --- | --- | --- | --- | --- | --- | --- | --- | --- | --- | --- | --- | --- | --- | --- | --- | --- | --- | --- | --- | --- | --- | --- | --- | --- | --- | --- | --- | --- | --- | --- | --- | --- | --- | --- | --- | --- | --- | --- | --- | --- | --- | --- | --- | --- | --- | --- | --- | --- | --- | --- | --- | --- | --- | --- | --- | --- | --- | --- | --- | --- | --- | --- | --- | --- | --- | --- | --- | --- | --- | --- | --- | --- | --- | --- | --- | --- | --- | --- | --- | --- | --- | --- | --- | --- | --- | --- | --- | --- | --- | --- | --- | --- | --- | --- | --- | --- | --- | --- | --- | --- | --- | --- | --- | --- | --- | --- | --- | --- | --- | --- | --- | --- | --- | --- | --- | --- | --- | --- | --- | --- | --- | --- | --- | --- | --- | --- | --- | --- | --- | --- | --- | --- | --- | --- | --- | --- | --- | --- | --- | --- | --- | --- | --- | --- | --- | --- | --- | --- | --- | --- | --- | --- | --- | --- | --- | --- | --- | --- | --- | --- | --- | --- | --- | --- | --- | --- | --- | --- | --- | --- | --- | --- | --- | --- | --- | --- | --- | --- | --- | --- | --- | --- | --- | --- | --- | --- | --- | --- | --- | --- | --- | --- | --- | --- | --- | --- | --- | --- | --- | --- | --- | --- | --- | --- | --- | --- | --- | --- | --- | --- | --- | --- | --- | --- | --- | --- | --- | --- | --- | --- | --- | --- | --- | --- | --- | --- | --- | --- | --- | --- | --- | --- | --- | --- | --- | --- | --- | --- | --- | --- | --- | --- | --- | --- | --- | --- | --- | --- | --- | --- | --- | --- | --- | --- | --- | --- | --- | --- | --- | --- | --- | --- | --- | --- | --- | --- | --- | --- | --- | --- | --- | --- | --- | --- | --- | --- | --- | --- | --- | --- | --- | --- | --- | --- | --- | --- | --- | --- | --- | --- | --- | --- | --- | --- | --- | --- | --- | --- | --- | --- | --- | --- | --- | --- | --- | --- | --- | --- | --- | --- | --- | --- | --- | --- | --- | --- | --- | --- | --- | --- | --- | --- | --- | --- | --- | --- | --- | --- | --- | --- | --- | --- | --- | --- | --- | --- | --- | --- | --- | --- | --- | --- | --- | --- | --- | --- | --- | --- | --- | --- | --- | --- | --- | --- | --- | --- | --- | --- | --- | --- | --- | --- | --- | --- | --- | --- | --- | --- | --- | --- | --- | --- | --- | --- | --- | --- | --- | --- | --- | --- | --- | --- | --- | --- | --- | --- | --- | --- | --- | --- | --- | --- | --- | --- | --- | --- | --- | --- | --- | --- | --- | --- | --- | --- | --- | --- | --- | --- | --- | --- | --- | --- | --- | --- | --- | --- | --- | --- | --- | --- | --- | --- | --- | --- | --- | --- | --- | --- | --- | --- | --- | --- | --- | --- | --- | --- | --- | --- | --- | --- | --- | --- | --- | --- | --- | --- | --- | --- | --- | --- | --- | --- | --- | --- | --- | --- | --- | --- | --- | --- | --- | --- | --- | --- | --- | --- | --- | --- | --- | --- | --- | --- | --- | --- | --- | --- | --- | --- | --- | --- | --- | --- | --- | --- | --- | --- | --- | --- | --- | --- | --- | --- | --- | --- | --- | --- | --- | --- | --- | --- | --- | --- | --- | --- | --- | --- | --- | --- | --- | --- | --- | --- | --- | --- | --- | --- | --- | --- | --- | --- | --- | --- | --- | --- | --- | --- | --- | --- | --- | --- | --- | --- | --- | --- | --- | --- | --- | --- | --- | --- | --- | --- | --- | --- | --- | --- | --- | --- | --- | --- | --- | --- | --- | --- | --- | --- | --- | --- | --- | --- | --- | --- | --- | --- | --- | --- | --- | --- | --- | --- | --- | --- | --- | --- | --- | --- | --- | --- | --- | --- | --- | --- | --- | --- | --- | --- | --- | --- | --- | --- | --- | --- | --- | --- | --- | --- | --- | --- | --- | --- | --- | --- | --- | --- | --- | --- | --- | --- | --- | --- | --- | --- | --- | --- | --- | --- | --- | --- | --- | --- | --- | --- | --- | --- | --- | --- | --- | --- | --- | --- | --- | --- | --- | --- | --- | --- | --- | --- | --- | --- | --- | --- | --- | --- | --- | --- | --- | --- | --- | --- | --- | --- | --- | --- | --- | --- | --- | --- | --- | --- | --- | --- | --- | --- | --- | --- | --- | --- | --- | --- | --- | --- | --- | --- | --- | --- | --- | --- | --- | --- | --- | --- | --- | --- | --- | --- | --- | --- | --- | --- | --- | --- | --- | --- | --- | --- | --- | --- | --- | --- | --- | --- | --- | --- | --- | --- | --- | --- | --- | --- | --- | --- | --- | --- | --- | --- | --- | --- | --- | --- | --- | --- | --- | --- | --- | --- | --- | --- | --- | --- | --- | --- | --- | --- | --- | --- | --- | --- | --- | --- | --- | --- | --- | --- | --- | --- | --- | --- | --- | --- | --- | --- | --- | --- | --- | --- | --- | --- | --- | --- | --- | --- | --- | --- | --- | --- | --- | --- | --- | --- | --- | --- | --- | --- | --- | --- | --- | --- | --- | --- | --- | --- | --- | --- | --- | --- | --- | --- | --- | --- | --- | --- | --- | --- | --- | --- | --- | --- | --- | --- | --- | --- | --- | --- | --- | --- | --- | --- | --- | --- | --- | --- | --- | --- | --- | --- | --- | --- | --- | --- | --- | --- | --- | --- | --- | --- | --- | --- | --- | --- | --- | --- | --- | --- | --- | --- | --- | --- | --- | --- | --- | --- | --- | --- | --- | --- | --- | --- | --- | --- | --- | --- | --- | --- | --- | --- | --- | --- | --- | --- | --- | --- | --- | --- | --- | --- | --- | --- | --- | --- | --- | --- | --- | --- | --- | --- | --- | --- | --- | --- | --- | --- | --- | --- | --- | --- | --- | --- | --- | --- | --- | --- | --- | --- | --- | --- | --- | --- | --- | --- | --- | --- | --- | --- | --- | --- | --- | --- | --- | --- | --- | --- | --- | --- | --- | --- | --- | --- | --- | --- | --- | --- | --- | --- | --- | --- | --- | --- | --- | --- | --- | --- | --- | --- | --- | --- | --- | --- | --- | --- | --- | --- | --- | --- | --- | --- | --- | --- | --- | --- | --- | --- | --- | --- | --- | --- | --- | --- | --- | --- | --- | --- | --- | --- | --- | --- | --- | --- | --- | --- | --- | --- | --- | --- | --- | --- | --- | --- | --- | --- | --- | --- | --- | --- | --- | --- | --- | --- | --- | --- | --- | --- | --- | --- | --- | --- | --- | --- | --- | --- | --- | --- | --- | --- | --- | --- | --- | --- | --- | --- | --- | --- | --- | --- | --- | --- | --- | --- | --- | --- | --- | --- | --- | --- | --- | --- | --- | --- | --- | --- | --- | --- | --- | --- | --- | --- | --- | --- | --- | --- | --- | --- | --- | --- | --- | --- | --- | --- | --- | --- | --- | --- | --- | --- | --- | --- | --- | --- | --- | --- | --- | --- | --- | --- | --- | --- | --- | --- | --- | --- | --- | --- | --- | --- | --- | --- | --- | --- | --- | --- | --- | --- | --- | --- | --- | --- | --- | --- | --- | --- | --- | --- | --- | --- | --- | --- | --- | --- | --- | --- | --- | --- | --- | --- | --- | --- | --- | --- | --- | --- | --- | --- | --- | --- | --- | --- | --- | --- | --- | --- | --- | --- | --- | --- | --- | --- | --- | --- | --- | --- | --- | --- | --- | --- | --- | --- | --- | --- | --- | --- | --- | --- | --- | --- | --- | --- | --- | --- | --- | --- | --- | --- | --- | --- | --- | --- | --- | --- | --- | --- | --- | --- | --- | --- | --- | --- | --- | --- | --- | --- | --- | --- | --- | --- | --- | --- | --- | --- | --- | --- | --- | --- | --- | --- | --- | --- | --- | --- | --- | --- | --- | --- | --- | --- | --- | --- | --- | --- | --- | --- | --- | --- | --- | --- | --- | --- | --- | --- | --- | --- | --- | --- | --- | --- | --- | --- | --- | --- | --- | --- | --- | --- | --- | --- | --- | --- | --- | --- | --- | --- | --- | --- | --- | --- | --- | --- | --- | --- | --- | --- | --- | --- | --- | --- | --- | --- | --- | --- | --- | --- | --- | --- | --- | --- | --- | --- | --- | --- | --- | --- | --- | --- | --- | --- | --- | --- | --- | --- | --- | --- | --- | --- | --- | --- | --- | --- | --- | --- | --- | --- | --- | --- | --- | --- | --- | --- | --- | --- | --- | --- | --- | --- | --- | --- | --- | --- | --- | --- | --- | --- | --- | --- | --- | --- | --- | --- | --- | --- | --- | --- | --- | --- | --- | --- | --- | --- | --- | --- | --- | --- | --- | --- | --- | --- | --- | --- | --- | --- | --- | --- | --- | --- | --- | --- | --- | --- | --- | --- | --- | --- | --- | --- | --- | --- | --- | --- | --- | --- | --- | --- | --- | --- | --- | --- | --- | --- | --- | --- | --- | --- | --- | --- | --- | --- | --- | --- | --- | --- | --- | --- | --- | --- | --- | --- | --- | --- | --- | --- | --- | --- | --- | --- | --- | --- | --- | --- | --- | --- | --- | --- | --- | --- | --- | --- | --- | --- | --- | --- | --- | --- | --- | --- | --- | --- | --- | --- | --- | --- | --- | --- | --- | --- | --- | --- | --- | --- | --- | --- | --- | --- | --- | --- | --- | --- | --- | --- | --- | --- | --- | --- | --- | --- | --- | --- | --- | --- | --- | --- | --- | --- | --- | --- | --- | --- | --- | --- | --- | --- | --- | --- | --- | --- | --- | --- | --- | --- | --- | --- | --- | --- | --- | --- | --- | --- | --- | --- | --- | --- | --- | --- | --- | --- | --- | --- | --- | --- | --- | --- | --- | --- | --- | --- | --- | --- | --- | --- | --- | --- | --- | --- | --- | --- | --- | --- | --- | --- | --- | --- | --- | --- | --- | --- | --- | --- | --- | --- | --- | --- | --- | --- | --- | --- | --- | --- | --- | --- | --- | --- | --- | --- | --- | --- | --- | --- | --- | --- | --- | --- | --- | --- | --- | --- | --- | --- | --- | --- | --- | --- | --- | --- | --- | --- | --- | --- | --- | --- | --- | --- | --- | --- | --- | --- | --- | --- | --- | --- | --- | --- | --- | --- | --- | --- | --- | --- | --- | --- | --- | --- | --- | --- | --- | --- | --- | --- | --- | --- | --- | --- | --- | --- | --- | --- | --- | --- | --- | --- | --- | --- | --- | --- | --- | --- | --- | --- | --- | --- | --- | --- | --- | --- | --- | --- | --- | --- | --- | --- | --- | --- | --- | --- | --- | --- | --- | --- | --- | --- | --- | --- | --- | --- | --- | --- | --- | --- | --- | --- | --- | --- | --- | --- | --- | --- | --- | --- | --- | --- | --- | --- | --- | --- | --- | --- | --- | --- | --- | --- | --- | --- | --- | --- | --- | --- | --- | --- | --- | --- | --- | --- | --- | --- | --- | --- | --- | --- | --- | --- | --- | --- | --- | --- | --- | --- | --- | --- | --- | --- | --- | --- | --- | --- | --- | --- | --- | --- | --- | --- | --- | --- | --- | --- | --- | --- | --- | --- | --- | --- | --- | --- | --- | --- | --- | --- | --- | --- | --- | --- | --- | --- | --- | --- | --- | --- | --- | --- | --- | --- | --- | --- | --- | --- | --- | --- | --- | --- | --- | --- | --- | --- | --- | --- | --- | --- | --- | --- | --- | --- | --- | --- | --- | --- | --- | --- | --- | --- | --- | --- | --- | --- | --- | --- | --- | --- | --- | --- | --- | --- | --- | --- | --- | --- | --- | --- | --- | --- | --- | --- | --- | --- | --- | --- | --- | --- | --- | --- | --- | --- | --- | --- | --- | --- | --- | --- | --- | --- | --- | --- | --- | --- | --- | --- | --- | --- | --- | --- | --- | --- | --- | --- | --- | --- | --- | --- | --- | --- | --- | --- | --- | --- | --- | --- | --- | --- | --- | --- | --- | --- | --- | --- | --- | --- | --- | --- | --- | --- | --- | --- | --- | --- | --- | --- | --- | --- | --- | --- | --- | --- | --- | --- | --- | --- | --- | --- | --- | --- | --- | --- | --- | --- | --- | --- | --- | --- | --- | --- | --- | --- | --- | --- | --- | --- | --- | --- | --- | --- | --- | --- | --- | --- | --- | --- | --- | --- | --- | --- | --- | --- | --- | --- | --- | --- | --- | --- | --- | --- | --- | --- | --- | --- | --- | --- | --- | --- | --- | --- | --- | --- | --- | --- | --- | --- | --- | --- | --- | --- | --- | --- | --- | --- | --- | --- | --- | --- | --- | --- | --- | --- | --- | --- | --- | --- | --- | --- | --- | --- | --- | --- | --- | --- | --- | --- | --- | --- | --- | --- | --- | --- | --- | --- | --- | --- | --- | --- | --- | --- | --- | --- | --- | --- | --- | --- | --- | --- | --- | --- | --- | --- | --- | --- | --- | --- | --- | --- | --- | --- | --- | --- | --- | --- | --- | --- | --- | --- | --- | --- | --- | --- | --- | --- | --- | --- | --- | --- | --- | --- | --- | --- | --- | --- | --- | --- | --- | --- | --- | --- | --- | --- | --- | --- | --- | --- | --- | --- | --- | --- | --- | --- | --- | --- | --- | --- | --- | --- | --- | --- | --- | --- | --- | --- | --- | --- | --- | --- | --- | --- | --- | --- | --- | --- | --- | --- | --- | --- | --- | --- | --- | --- | --- | --- | --- | --- | --- | --- | --- | --- | --- | --- | --- | --- | --- | --- | --- | --- | --- | --- | --- | --- | --- | --- | --- | --- | --- | --- | --- | --- | --- | --- | --- | --- | --- | --- | --- | --- | --- | --- | --- | --- | --- | --- | --- | --- | --- | --- | --- | --- | --- | --- | --- | --- | --- | --- | --- | --- | --- | --- | --- | --- | --- | --- | --- | --- | --- | --- | --- | --- | --- | --- | --- | --- | --- | --- | --- | --- | --- | --- | --- | --- | --- | --- | --- | --- | --- | --- | --- | --- | --- | --- | --- | --- | --- | --- | --- | --- | --- | --- | --- | --- | --- | --- | --- | --- | --- | --- | --- | --- | --- | --- | --- | --- | --- | --- | --- | --- | --- | --- | --- | --- | --- | --- | --- | --- | --- | --- | --- | --- | --- | --- | --- | --- | --- | --- | --- | --- | --- | --- | --- | --- | --- | --- | --- | --- | --- | --- | --- | --- | --- | --- | --- | --- | --- | --- | --- | --- | --- | --- | --- | --- | --- | --- | --- | --- | --- | --- | --- | --- | --- | --- | --- | --- | --- | --- | --- | --- | --- | --- | --- | --- | --- | --- | --- | --- | --- | --- | --- | --- | --- | --- | --- | --- | --- | --- | --- | --- | --- | --- | --- | --- | --- | --- | --- | --- | --- | --- | --- | --- | --- | --- | --- | --- | --- | --- | --- | --- | --- | --- | --- | --- | --- | --- | --- | --- | --- | --- | --- | --- | --- | --- | --- | --- | --- | --- | --- | --- | --- | --- | --- | --- | --- | --- | --- | --- | --- | --- | --- | --- | --- | --- | --- | --- | --- | --- | --- | --- | --- | --- | --- | --- | --- | --- | --- | --- | --- | --- | --- | --- | --- | --- | --- | --- | --- | --- | --- | --- | --- | --- | --- | --- | --- | --- | --- | --- | --- | --- | --- | --- | --- | --- | --- | --- | --- | --- | --- | --- | --- | --- | --- | --- | --- | --- | --- | --- | --- | --- | --- | --- | --- | --- | --- | --- | --- | --- | --- | --- | --- | --- | --- | --- | --- | --- | --- | --- | --- | --- | --- | --- | --- | --- | --- | --- | --- | --- | --- | --- | --- | --- | --- | --- | --- | --- | --- | --- | --- | --- | --- | --- | --- | --- | --- | --- | --- | --- | --- | --- | --- | --- | --- | --- | --- | --- | --- | --- | --- | --- | --- | --- | --- | --- | --- | --- | --- | --- | --- | --- | --- | --- | --- | --- | --- | --- | --- | --- | --- | --- | --- | --- | --- | --- | --- | --- | --- | --- | --- | --- | --- | --- | --- | --- | --- | --- | --- | --- | --- | --- | --- | --- | --- | --- | --- | --- | --- | --- | --- | --- | --- | --- | --- | --- | --- | --- | --- | --- | --- | --- | --- | --- | --- | --- | --- | --- | --- | --- | --- | --- | --- | --- | --- | --- | --- | --- | --- | --- | --- | --- | --- | --- | --- | --- | --- | --- | --- | --- | --- | --- | --- | --- | --- | --- | --- | --- | --- | --- | --- | --- | --- | --- | --- | --- | --- | --- | --- | --- | --- | --- | --- | --- | --- | --- | --- | --- | --- | --- | --- | --- | --- | --- | --- | --- | --- | --- | --- | --- | --- | --- | --- | --- | --- | --- | --- | --- | --- | --- | --- | --- | --- | --- | --- | --- | --- | --- | --- | --- | --- | --- | --- | --- | --- | --- | --- | --- | --- | --- | --- | --- | --- | --- | --- | --- | --- | --- | --- | --- | --- | --- | --- | --- | --- | --- | --- | --- | --- | --- | --- | --- | --- | --- | --- | --- | --- | --- | --- | --- | --- | --- | --- | --- | --- | --- | --- | --- | --- | --- | --- | --- | --- | --- | --- | --- | --- | --- | --- | --- | --- | --- | --- | --- | --- | --- | --- | --- | --- | --- | --- | --- | --- | --- | --- | --- | --- | --- | --- | --- | --- | --- | --- | --- | --- | --- | --- | --- | --- | --- | --- | --- | --- | --- | --- | --- | --- | --- | --- | --- | --- | --- | --- | --- | --- | --- | --- | --- | --- | --- | --- | --- | --- | --- | --- | --- | --- | --- | --- | --- | --- | --- | --- | --- | --- | --- | --- | --- | --- | --- | --- | --- | --- | --- | --- | --- | --- | --- | --- | --- | --- | --- | --- | --- | --- | --- | --- | --- | --- | --- | --- | --- | --- | --- | --- | --- | --- | --- | --- | --- | --- | --- | --- | --- | --- | --- | --- | --- | --- | --- | --- | --- | --- | --- | --- | --- | --- | --- | --- | --- | --- | --- | --- | --- | --- | --- | --- | --- | --- | --- | --- | --- | --- | --- | --- | --- | --- | --- | --- | --- | --- | --- | --- | --- | --- | --- | --- | --- | --- | --- | --- | --- | --- | --- | --- | --- | --- | --- | --- | --- | --- | --- | --- | --- | --- | --- | --- | --- | --- | --- | --- | --- | --- | --- | --- | --- | --- | --- | --- | --- | --- | --- | --- | --- | --- | --- | --- | --- | --- | --- | --- | --- | --- | --- | --- | --- | --- | --- | --- | --- | --- | --- | --- | --- | --- | --- | --- | --- | --- | --- | --- | --- | --- | --- | --- | --- | --- | --- | --- | --- | --- | --- | --- | --- | --- | --- | --- | --- | --- | --- | --- | --- | --- | --- | --- | --- | --- | --- | --- | --- | --- | --- | --- | --- | --- | --- | --- | --- | --- | --- | --- | --- | --- | --- | --- | --- | --- | --- | --- | --- | --- | --- | --- | --- | --- | --- | --- | --- | --- | --- | --- | --- | --- | --- | --- | --- | --- | --- | --- | --- | --- | --- | --- | --- | --- | --- | --- | --- | --- | --- | --- | --- | --- | --- | --- | --- | --- | --- | --- | --- | --- | --- | --- | --- | --- | --- | --- | --- | --- | --- | --- | --- | --- | --- | --- | --- | --- | --- | --- | --- | --- | --- | --- | --- | --- | --- | --- | --- | --- | --- | --- | --- | --- | --- | --- | --- | --- | --- | --- | --- | --- | --- | --- | --- | --- | --- | --- | --- | --- | --- | --- | --- | --- | --- | --- | --- | --- | --- | --- | --- | --- | --- | --- | --- | --- | --- | --- | --- | --- | --- | --- | --- | --- | --- | --- | --- | --- | --- | --- | --- | --- | --- | --- | --- | --- | --- | --- | --- | --- | --- | --- | --- | --- | --- | --- | --- | --- | --- | --- | --- | --- | --- | --- | --- | --- | --- | --- | --- | --- | --- | --- | --- | --- | --- | --- | --- | --- | --- | --- | --- | --- | --- | --- | --- | --- | --- | --- | --- | --- | --- | --- | --- | --- | --- | --- | --- | --- | --- | --- | --- | --- | --- | --- | --- | --- | --- | --- | --- | --- | --- | --- | --- | --- | --- | --- | --- | --- | --- | --- | --- | --- | --- | --- | --- | --- | --- | --- | --- | --- | --- | --- | --- | --- | --- | --- | --- | --- | --- | --- | --- | --- | --- | --- | --- | --- | --- | --- | --- | --- | --- | --- | --- | --- | --- | --- | --- | --- | --- | --- | --- | --- | --- | --- | --- | --- | --- | --- | --- | --- | --- | --- | --- | --- | --- | --- | --- | --- | --- | --- | --- | --- | --- | --- | --- | --- | --- | --- | --- | --- | --- | --- | --- | --- | --- | --- | --- | --- | --- | --- | --- | --- | --- | --- | --- | --- | --- | --- | --- | --- | --- | --- | --- | --- | --- | --- | --- | --- | --- | --- | --- | --- | --- | --- | --- | --- | --- | --- | --- | --- | --- | --- | --- | --- | --- | --- | --- | --- | --- | --- | --- | --- | --- | --- | --- | --- | --- | --- | --- | --- | --- | --- | --- | --- | --- | --- | --- | --- | --- | --- | --- | --- | --- | --- | --- | --- | --- | --- | --- | --- | --- | --- | --- | --- | --- | --- | --- | --- | --- | --- | --- | --- | --- | --- | --- | --- | --- | --- | --- | --- | --- | --- | --- | --- | --- | --- | --- | --- | --- | --- | --- | --- | --- | --- | --- | --- | --- | --- | --- | --- | --- | --- | --- | --- | --- | --- | --- | --- | --- | --- | --- | --- | --- | --- | --- | --- | --- | --- | --- | --- | --- | --- | --- | --- | --- | --- | --- | --- | --- | --- | --- | --- | --- | --- | --- | --- | --- | --- | --- | --- | --- | --- | --- | --- | --- | --- | --- | --- | --- | --- | --- | --- | --- | --- | --- | --- | --- | --- | --- | --- | --- | --- | --- | --- | --- | --- | --- | --- | --- | --- | --- | --- | --- | --- | --- | --- | --- | --- | --- | --- | --- | --- | --- | --- | --- | --- | --- | --- | --- | --- | --- | --- | --- | --- | --- | --- | --- | --- | --- | --- | --- | --- | --- | --- | --- | --- | --- | --- | --- | --- | --- | --- | --- | --- | --- | --- | --- | --- | --- | --- | --- | --- | --- | --- | --- | --- | --- | --- | --- | --- | --- | --- | --- | --- | --- | --- | --- | --- | --- | --- | --- | --- | --- | --- | --- | --- | --- | --- | --- | --- | --- | --- | --- | --- | --- | --- | --- | --- | --- | --- | --- | --- | --- | --- | --- | --- | --- | --- | --- | --- | --- | --- | --- | --- | --- | --- | --- | --- | --- | --- | --- | --- | --- | --- | --- | --- | --- | --- | --- | --- | --- | --- | --- | --- | --- | --- | --- | --- | --- | --- | --- | --- | --- | --- | --- | --- | --- | --- | --- | --- | --- | --- | --- | --- | --- | --- | --- | --- | --- | --- | --- | --- | --- | --- | --- | --- | --- | --- | --- | --- | --- | --- | --- | --- | --- | --- | --- | --- | --- | --- | --- | --- | --- | --- | --- | --- | --- | --- | --- | --- | --- | --- | --- | --- | --- | --- | --- | --- | --- | --- | --- | --- | --- | --- | --- | --- | --- | --- | --- | --- | --- | --- | --- | --- | --- | --- | --- | --- | --- | --- | --- | --- | --- | --- | --- | --- | --- | --- | --- | --- | --- | --- | --- | --- | --- | --- | --- | --- | --- | --- | --- | --- | --- | --- | --- | --- | --- | --- | --- | --- | --- | --- | --- | --- | --- | --- | --- | --- | --- | --- | --- | --- | --- | --- | --- | --- | --- | --- | --- | --- | --- | --- | --- | --- | --- | --- | --- | --- | --- | --- | --- | --- | --- | --- | --- | --- | --- | --- | --- | --- | --- | --- | --- | --- | --- | --- | --- | --- | --- | --- | --- | --- | --- | --- | --- | --- | --- | --- | --- | --- | --- | --- | --- | --- | --- | --- | --- | --- | --- | --- | --- | --- | --- | --- | --- | --- | --- | --- | --- | --- | --- | --- | --- | --- | --- | --- | --- | --- | --- | --- | --- | --- | --- | --- | --- | --- | --- | --- | --- | --- | --- | --- | --- | --- | --- | --- | --- | --- | --- | --- | --- | --- | --- | --- | --- | --- | --- | --- | --- | --- | --- | --- | --- | --- | --- | --- | --- | --- | --- | --- | --- | --- | --- | --- | --- | --- | --- | --- | --- | --- | --- | --- | --- | --- | --- | --- | --- | --- | --- | --- | --- | --- | --- | --- | --- | --- | --- | --- | --- | --- | --- | --- | --- | --- | --- | --- | --- | --- | --- | --- | --- | --- | --- | --- | --- | --- | --- | --- | --- | --- | --- | --- | --- | --- | --- | --- | --- | --- | --- | --- | --- | --- | --- | --- | --- | --- | --- | --- | --- | --- | --- | --- | --- | --- | --- | --- | --- | --- | --- | --- | --- | --- | --- | --- | --- | --- | --- | --- | --- | --- | --- | --- | --- | --- | --- | --- | --- | --- | --- | --- | --- | --- | --- | --- | --- | --- | --- | --- | --- | --- | --- | --- | --- | --- | --- | --- | --- | --- | --- | --- | --- | --- | --- | --- | --- | --- | --- | --- | --- | --- | --- | --- | --- | --- | --- | --- | --- | --- | --- | --- | --- | --- | --- | --- | --- | --- | --- | --- | --- | --- | --- | --- | --- | --- | --- | --- | --- | --- | --- | --- | --- | --- | --- | --- | --- | --- | --- | --- | --- | --- | --- | --- | --- | --- | --- | --- | --- | --- | --- | --- | --- | --- | --- | --- | --- | --- | --- | --- | --- | --- | --- | --- | --- | --- | --- | --- | --- | --- | --- | --- | --- | --- | --- | --- | --- | --- | --- | --- | --- | --- | --- | --- | --- | --- | --- | --- | --- | --- | --- | --- | --- | --- | --- | --- | --- | --- | --- | --- | --- | --- | --- | --- | --- | --- | --- | --- | --- | --- | --- | --- | --- | --- | --- | --- | --- | --- | --- | --- | --- | --- | --- | --- | --- | --- | --- | --- | --- | --- | --- | --- | --- | --- | --- | --- | --- | --- | --- | --- | --- | --- | --- | --- | --- | --- | --- | --- | --- | --- | --- | --- | --- | --- | --- | --- | --- | --- | --- | --- | --- | --- | --- | --- | --- | --- | --- | --- | --- | --- | --- | --- | --- | --- | --- | --- | --- | --- | --- | --- | --- | --- | --- | --- | --- | --- | --- | --- | --- | --- | --- | --- | --- | --- | --- | --- | --- | --- | --- | --- | --- | --- | --- | --- | --- | --- | --- | --- | --- | --- | --- | --- | --- | --- | --- | --- | --- | --- | --- | --- | --- | --- | --- | --- | --- | --- | --- | --- | --- | --- | --- | --- | --- | --- | --- | --- | --- | --- | --- | --- | --- | --- | --- | --- | --- | --- | --- | --- | --- | --- | --- | --- | --- | --- | --- | --- | --- | --- | --- | --- | --- | --- | --- | --- | --- | --- | --- | --- | --- | --- | --- | --- | --- | --- | --- | --- | --- | --- | --- | --- | --- | --- | --- | --- | --- | --- | --- | --- | --- | --- | --- | --- | --- | --- | --- | --- | --- | --- | --- | --- | --- | --- | --- | --- | --- | --- | --- | --- | --- | --- | --- | --- | --- | --- | --- | --- | --- | --- | --- | --- | --- | --- | --- | --- | --- | --- | --- | --- | --- | --- | --- | --- | --- | --- | --- | --- | --- | --- | --- | --- | --- | --- | --- | --- | --- | --- | --- | --- | --- | --- | --- | --- | --- | --- | --- | --- | --- | --- | --- | --- | --- | --- | --- | --- | --- | --- | --- | --- | --- | --- | --- | --- | --- | --- | --- | --- | --- | --- | --- | --- | --- | --- | --- | --- | --- | --- | --- | --- | --- | --- | --- | --- | --- | --- | --- | --- | --- | --- | --- | --- | --- | --- | --- | --- | --- | --- | --- | --- | --- | --- | --- | --- | --- | --- | --- | --- | --- | --- | --- | --- | --- | --- | --- | --- | --- | --- | --- | --- | --- | --- | --- | --- | --- | --- | --- | --- | --- | --- | --- | --- | --- | --- | --- | --- | --- | --- | --- | --- | --- | --- | --- | --- | --- | --- | --- | --- | --- | --- | --- | --- | --- | --- | --- | --- | --- | --- | --- | --- | --- | --- | --- | --- | --- | --- | --- | --- | --- | --- | --- | --- | --- | --- | --- | --- | --- | --- | --- | --- | --- | --- | --- | --- | --- | --- | --- | --- | --- | --- | --- | --- | --- | --- | --- | --- | --- | --- | --- | --- | --- | --- | --- | --- | --- | --- | --- | --- | --- | --- | --- | --- | --- | --- | --- | --- | --- | --- | --- | --- | --- | --- | --- | --- | --- | --- | --- | --- | --- | --- | --- | --- | --- | --- | --- | --- | --- | --- | --- | --- | --- | --- | --- | --- | --- | --- | --- | --- | --- | --- | --- | --- | --- | --- | --- | --- | --- | --- | --- | --- | --- | --- | --- | --- | --- | --- | --- | --- | --- | --- | --- | --- | --- | --- | --- | --- | --- | --- | --- | --- | --- | --- | --- | --- | --- | --- | --- | --- | --- | --- | --- | --- | --- | --- | --- | --- | --- | --- | --- | --- | --- | --- | --- | --- | --- | --- | --- | --- | --- | --- | --- | --- | --- | --- | --- | --- | --- | --- | --- | --- | --- | --- | --- | --- | --- | --- | --- | --- | --- | --- | --- | --- | --- | --- | --- | --- | --- | --- | --- | --- | --- | --- | --- | --- | --- | --- | --- | --- | --- | --- | --- | --- | --- | --- | --- | --- | --- | --- | --- | --- | --- | --- | --- | --- | --- | --- | --- | --- | --- | --- | --- | --- | --- | --- | --- | --- | --- | --- | --- | --- | --- | --- | --- | --- | --- | --- | --- | --- | --- | --- | --- | --- | --- | --- | --- | --- | --- | --- | --- | --- | --- | --- | --- | --- | --- | --- | --- | --- | --- | --- | --- | --- | --- | --- | --- | --- | --- | --- | --- | --- | --- | --- | --- | --- | --- | --- | --- | --- | --- | --- | --- | --- | --- | --- | --- | --- | --- | --- | --- | --- | --- | --- | --- | --- | --- | --- | --- | --- | --- | --- | --- | --- | --- | --- | --- | --- | --- | --- | --- | --- | --- | --- | --- | --- | --- | --- | --- | --- | --- | --- | --- | --- | --- | --- | --- | --- | --- | --- | --- | --- | --- | --- | --- | --- | --- | --- | --- | --- | --- | --- | --- | --- | --- | --- | --- | --- | --- | --- | --- | --- | --- | --- | --- | --- | --- | --- | --- | --- | --- | --- | --- | --- | --- | --- | --- | --- | --- | --- | --- | --- | --- | --- | --- | --- | --- | --- | --- | --- | --- | --- | --- | --- | --- | --- | --- | --- | --- | --- | --- | --- | --- | --- | --- | --- | --- | --- | --- | --- | --- | --- | --- | --- | --- | --- | --- | --- | --- | --- | --- | --- | --- | --- | --- | --- | --- | --- | --- | --- | --- | --- | --- | --- | --- | --- | --- | --- | --- | --- | --- | --- | --- | --- | --- | --- | --- | --- | --- | --- | --- | --- | --- | --- | --- | --- | --- | --- | --- | --- | --- | --- | --- | --- | --- | --- | --- | --- | --- | --- | --- | --- | --- | --- | --- | --- | --- | --- | --- | --- | --- | --- | --- | --- | --- | --- | --- | --- | --- | --- | --- | --- | --- | --- | --- | --- | --- | --- | --- | --- | --- | --- | --- | --- | --- | --- | --- | --- | --- | --- | --- | --- | --- | --- | --- | --- | --- | --- | --- | --- | --- | --- | --- | --- | --- | --- | --- | --- | --- | --- | --- | --- | --- | --- | --- | --- | --- | --- | --- | --- | --- | --- | --- | --- | --- | --- | --- | --- | --- | --- | --- | --- | --- | --- | --- | --- | --- | --- | --- | --- | --- | --- | --- | --- | --- | --- | --- | --- | --- | --- | --- | --- | --- | --- | --- | --- | --- | --- | --- | --- | --- | --- | --- | --- | --- | --- | --- | --- | --- | --- | --- | --- | --- | --- | --- | --- | --- | --- | --- | --- | --- | --- | --- | --- | --- | --- | --- | --- | --- | --- | --- | --- | --- | --- | --- | --- | --- | --- | --- | --- | --- | --- | --- | --- | --- | --- | --- | --- | --- | --- | --- | --- | --- | --- | --- | --- | --- | --- | --- | --- | --- | --- | --- | --- | --- | --- | --- | --- | --- | --- | --- | --- | --- | --- | --- | --- | --- | --- | --- | --- | --- | --- | --- | --- | --- | --- | --- | --- | --- | --- | --- | --- | --- | --- | --- | --- | --- | --- | --- | --- | --- | --- | --- | --- | --- | --- | --- | --- | --- | --- | --- | --- | --- | --- | --- | --- | --- | --- | --- | --- | --- | --- | --- | --- | --- | --- | --- | --- | --- | --- | --- | --- | --- | --- | --- | --- | --- | --- | --- | --- | --- | --- | --- | --- | --- | --- | --- | --- | --- | --- | --- | --- | --- | --- | --- | --- | --- | --- | --- | --- | --- | --- | --- | --- | --- | --- | --- | --- | --- | --- | --- | --- | --- | --- | --- | --- | --- | --- | --- | --- | --- | --- | --- | --- | --- | --- | --- | --- | --- | --- | --- | --- | --- | --- | --- | --- | --- | --- | --- | --- | --- | --- | --- | --- | --- | --- | --- | --- | --- | --- | --- | --- | --- | --- | --- | --- | --- | --- | --- | --- | --- | --- | --- | --- | --- | --- | --- | --- | --- | --- | --- | --- | --- | --- | --- | --- | --- | --- | --- | --- | --- | --- | --- | --- | --- | --- | --- | --- | --- | --- | --- | --- | --- | --- | --- | --- | --- | --- | --- | --- | --- | --- | --- | --- | --- | --- | --- | --- | --- | --- | --- | --- | --- | --- | --- | --- | --- | --- | --- | --- | --- | --- | --- | --- | --- | --- | --- | --- | --- | --- | --- | --- | --- | --- | --- | --- | --- | --- | --- | --- | --- | --- | --- | --- | --- | --- | --- | --- | --- | --- | --- | --- | --- | --- | --- | --- | --- | --- | --- | --- | --- | --- | --- | --- | --- | --- | --- | --- | --- | --- | --- | --- | --- | --- | --- | --- | --- | --- | --- | --- | --- | --- | --- | --- | --- | --- | --- | --- | --- | --- | --- | --- | --- | --- | --- | --- | --- | --- | --- | --- | --- | --- | --- | --- | --- | --- | --- | --- | --- | --- | --- | --- | --- | --- | --- | --- | --- | --- | --- | --- | --- | --- | --- | --- | --- | --- | --- | --- | --- | --- | --- | --- | --- | --- | --- | --- | --- | --- | --- | --- | --- | --- | --- | --- | --- | --- | --- | --- | --- | --- | --- | --- | --- | --- | --- | --- | --- | --- | --- | --- | --- | --- | --- | --- | --- | --- | --- | --- | --- | --- | --- | --- | --- | --- | --- | --- | --- | --- | --- | --- | --- | --- | --- | --- | --- | --- | --- | --- | --- | --- | --- | --- | --- | --- | --- | --- | --- | --- | --- | --- | --- | --- | --- | --- | --- | --- | --- | --- | --- | --- | --- | --- | --- | --- | --- | --- | --- | --- | --- | --- | --- | --- | --- | --- | --- | --- | --- | --- | --- | --- | --- | --- | --- | --- | --- | --- | --- | --- | --- | --- | --- | --- | --- | --- | --- | --- | --- | --- | --- | --- | --- | --- | --- | --- | --- | --- | --- | --- | --- | --- | --- | --- | --- | --- | --- | --- | --- | --- | --- | --- | --- | --- | --- | --- | --- | --- | --- | --- | --- | --- | --- | --- | --- | --- | --- | --- | --- | --- | --- | --- | --- | --- | --- | --- | --- | --- | --- | --- | --- | --- | --- | --- | --- | --- | --- | --- | --- | --- | --- | --- | --- | --- | --- | --- | --- | --- | --- | --- | --- | --- | --- | --- | --- | --- | --- | --- | --- | --- | --- | --- | --- | --- | --- | --- | --- | --- | --- | --- | --- | --- | --- | --- | --- | --- | --- | --- | --- | --- | --- | --- | --- | --- | --- | --- | --- | --- | --- | --- | --- | --- | --- | --- | --- | --- | --- | --- | --- | --- | --- | --- | --- | --- | --- | --- | --- | --- | --- | --- | --- | --- | --- | --- | --- | --- | --- | --- | --- | --- | --- | --- | --- | --- | --- | --- | --- | --- | --- | --- | --- | --- | --- | --- | --- | --- | --- | --- | --- | --- | --- | --- | --- | --- | --- | --- | --- | --- | --- | --- | --- | --- | --- | --- | --- | --- | --- | --- | --- | --- | --- | --- | --- | --- | --- | --- | --- | --- | --- | --- | --- | --- | --- | --- | --- | --- | --- | --- | --- | --- | --- | --- | --- | --- | --- | --- | --- | --- | --- | --- | --- | --- | --- | --- | --- | --- | --- | --- | --- | --- | --- | --- | --- | --- | --- | --- | --- | --- | --- | --- | --- | --- | --- | --- | --- | --- | --- | --- | --- | --- | --- | --- | --- | --- | --- | --- | --- | --- | --- | --- | --- | --- | --- | --- | --- | --- | --- | --- | --- | --- | --- | --- | --- | --- | --- | --- | --- | --- | --- | --- | --- | --- | --- | --- | --- | --- | --- | --- | --- | --- | --- | --- | --- | --- | --- | --- | --- | --- | --- | --- | --- | --- | --- | --- | --- | --- | --- | --- | --- | --- | --- | --- | --- | --- | --- | --- | --- | --- | --- | --- | --- | --- | --- | --- | --- | --- | --- | --- | --- | --- | --- | --- | --- | --- | --- | --- | --- | --- | --- | --- | --- | --- | --- | --- | --- | --- | --- | --- | --- | --- | --- | --- | --- | --- | --- | --- | --- | --- | --- | --- | --- | --- | --- | --- | --- | --- | --- | --- | --- | --- | --- | --- | --- | --- | --- | --- | --- | --- | --- | --- | --- | --- | --- | --- | --- | --- | --- | --- | --- | --- | --- | --- | --- | --- | --- | --- | --- | --- | --- | --- | --- | --- | --- | --- | --- | --- | --- | --- | --- | --- | --- | --- | --- | --- | --- | --- | --- | --- | --- | --- | --- | --- | --- | --- | --- | --- | --- | --- | --- | --- | --- | --- | --- | --- | --- | --- | --- | --- | --- | --- | --- | --- | --- | --- | --- | --- | --- | --- | --- | --- | --- | --- | --- | --- | --- | --- | --- | --- | --- | --- | --- | --- | --- | --- | --- | --- | --- | --- | --- | --- | --- | --- | --- | --- | --- | --- | --- | --- | --- | --- | --- | --- | --- | --- | --- | --- | --- | --- | --- | --- | --- | --- | --- | --- | --- | --- | --- | --- | --- | --- | --- | --- | --- | --- | --- | --- | --- | --- | --- | --- | --- | --- | --- | --- | --- | --- | --- | --- | --- | --- | --- | --- | --- | --- | --- | --- | --- | --- | --- | --- | --- | --- | --- | --- | --- | --- | --- | --- | --- | --- | --- | --- | --- | --- | --- | --- | --- | --- | --- | --- | --- | --- | --- | --- | --- | --- | --- | --- | --- | --- | --- | --- | --- | --- | --- | --- | --- | --- | --- | --- | --- | --- | --- | --- | --- | --- | --- | --- | --- | --- | --- | --- | --- | --- | --- | --- | --- | --- | --- | --- | --- | --- | --- | --- | --- | --- | --- | --- | --- | --- | --- | --- | --- | --- | --- | --- | --- | --- | --- | --- | --- | --- | --- | --- | --- | --- | --- | --- | --- | --- | --- | --- | --- | --- | --- | --- | --- | --- | --- | --- | --- | --- | --- | --- | --- | --- | --- | --- | --- | --- | --- | --- | --- | --- | --- | --- | --- | --- | --- | --- | --- | --- | --- | --- | --- | --- | --- | --- | --- | --- | --- | --- | --- | --- | --- | --- | --- | --- | --- | --- | --- | --- | --- | --- | --- | --- | --- | --- | --- | --- | --- | --- | --- | --- | --- | --- | --- | --- | --- | --- | --- | --- | --- | --- | --- | --- | --- | --- | --- | --- | --- | --- | --- | --- | --- | --- | --- | --- | --- | --- | --- | --- | --- | --- | --- | --- | --- | --- | --- | --- | --- | --- | --- | --- | --- | --- | --- | --- | --- | --- | --- | --- | --- | --- | --- | --- | --- | --- | --- | --- | --- | --- | --- | --- | --- | --- | --- | --- | --- | --- | --- | --- | --- | --- | --- | --- | --- | --- | --- | --- | --- | --- | --- | --- | --- | --- | --- | --- | --- | --- | --- | --- | --- | --- | --- | --- | --- | --- | --- | --- | --- | --- | --- | --- | --- | --- | --- | --- | --- | --- | --- | --- | --- | --- | --- | --- | --- | --- | --- | --- | --- | --- | --- | --- | --- | --- | --- | --- | --- | --- | --- | --- | --- | --- | --- | --- | --- | --- | --- | --- | --- | --- | --- | --- | --- | --- | --- | --- | --- | --- | --- | --- | --- | --- | --- | --- | --- | --- | --- | --- | --- | --- | --- | --- | --- | --- | --- | --- | --- | --- | --- | --- | --- | --- | --- | --- | --- | --- | --- | --- | --- | --- | --- | --- | --- | --- | --- | --- | --- | --- | --- | --- | --- | --- | --- | --- | --- | --- | --- | --- | --- | --- | --- | --- | --- | --- | --- | --- | --- | --- | --- | --- | --- | --- | --- | --- | --- | --- | --- | --- | --- | --- | --- | --- | --- | --- | --- | --- | --- | --- | --- | --- | --- | --- | --- | --- | --- | --- | --- | --- | --- | --- | --- | --- | --- | --- | --- | --- | --- | --- | --- | --- | --- | --- | --- | --- | --- | --- | --- | --- | --- | --- | --- | --- | --- | --- | --- | --- | --- | --- | --- | --- | --- | --- | --- | --- | --- | --- | --- | --- | --- | --- | --- | --- | --- | --- | --- | --- | --- | --- | --- | --- | --- | --- | --- | --- | --- | --- | --- | --- | --- | --- | --- | --- | --- | --- | --- | --- | --- | --- | --- | --- | --- | --- | --- | --- | --- | --- | --- | --- | --- | --- | --- | --- | --- | --- | --- | --- | --- | --- | --- | --- | --- | --- | --- | --- | --- | --- | --- | --- | --- | --- | --- | --- | --- | --- | --- | --- | --- | --- | --- | --- | --- | --- | --- | --- | --- | --- | --- | --- | --- | --- | --- | --- | --- | --- | --- | --- | --- | --- | --- | --- | --- | --- | --- | --- | --- | --- | --- | --- | --- | --- | --- | --- | --- | --- | --- | --- | --- | --- | --- | --- | --- | --- | --- | --- | --- | --- | --- | --- | --- | --- | --- | --- | --- | --- | --- | --- | --- | --- | --- | --- | --- | --- | --- | --- | --- | --- | --- | --- | --- | --- | --- | --- | --- | --- | --- | --- | --- | --- | --- | --- | --- | --- | --- | --- | --- | --- | --- | --- | --- | --- | --- | --- | --- | --- | --- | --- | --- | --- | --- | --- | --- | --- | --- | --- | --- | --- | --- | --- | --- | --- | --- | --- | --- | --- | --- | --- | --- | --- | --- | --- | --- | --- | --- | --- | --- | --- | --- | --- | --- | --- | --- | --- | --- | --- | --- | --- | --- | --- | --- | --- | --- | --- | --- | --- | --- | --- | --- | --- | --- | --- | --- | --- | --- | --- | --- | --- | --- | --- | --- | --- | --- | --- | --- | --- | --- | --- | --- | --- | --- | --- | --- | --- | --- | --- | --- | --- | --- | --- | --- | --- | --- | --- | --- | --- | --- | --- | --- | --- | --- | --- | --- | --- | --- | --- | --- | --- | --- | --- | --- | --- | --- | --- | --- | --- | --- | --- | --- | --- | --- | --- | --- | --- | --- | --- | --- | --- | --- | --- | --- | --- | --- | --- | --- | --- | --- | --- | --- | --- | --- | --- | --- | --- | --- | --- | --- | --- | --- | --- | --- | --- | --- | --- | --- | --- | --- | --- | --- | --- | --- | --- | --- | --- | --- | --- | --- | --- | --- | --- | --- | --- | --- | --- | --- | --- | --- | --- | --- | --- | --- | --- | --- | --- | --- | --- | --- | --- | --- | --- | --- | --- | --- | --- | --- | --- | --- | --- | --- | --- | --- | --- | --- | --- | --- | --- | --- | --- | --- | --- | --- | --- | --- | --- | --- | --- | --- | --- | --- | --- | --- | --- | --- | --- | --- | --- | --- | --- | --- | --- | --- | --- | --- | --- | --- | --- | --- | --- | --- | --- | --- | --- | --- | --- | --- | --- | --- | --- | --- | --- | --- | --- | --- | --- | --- | --- | --- | --- | --- | --- | --- | --- | --- | --- | --- | --- | --- | --- | --- | --- | --- | --- | --- | --- | --- | --- | --- | --- | --- | --- | --- | --- | --- | --- | --- | --- | --- | --- | --- | --- | --- | --- | --- | --- | --- | --- | --- | --- | --- | --- | --- | --- | --- | --- | --- | --- | --- | --- | --- | --- | --- | --- | --- | --- | --- | --- | --- | --- | --- | --- | --- | --- | --- | --- | --- | --- | --- | --- | --- | --- | --- | --- | --- | --- | --- | --- | --- | --- | --- | --- | --- | --- | --- | --- | --- | --- | --- | --- | --- | --- | --- | --- | --- | --- | --- | --- | --- | --- | --- | --- | --- | --- | --- | --- | --- | --- | --- | --- | --- | --- | --- | --- | --- | --- | --- | --- | --- | --- | --- | --- | --- | --- | --- | --- | --- | --- | --- | --- | --- | --- | --- | --- | --- | --- | --- | --- | --- | --- | --- | --- | --- | --- | --- | --- | --- | --- | --- | --- | --- | --- | --- | --- | --- | --- | --- | --- | --- | --- | --- | --- | --- | --- | --- | --- | --- | --- | --- | --- | --- | --- | --- | --- | --- | --- | --- | --- | --- | --- | --- | --- | --- | --- | --- | --- | --- | --- | --- | --- | --- | --- | --- | --- | --- | --- | --- | --- | --- | --- | --- | --- | --- | --- | --- | --- | --- | --- | --- | --- | --- | --- | --- | --- | --- | --- | --- | --- | --- | --- | --- | --- | --- | --- | --- | --- | --- | --- | --- | --- | --- | --- | --- | --- | --- | --- | --- | --- | --- | --- | --- | --- | --- | --- | --- | --- | --- | --- | --- | --- | --- | --- | --- | --- | --- | --- | --- | --- | --- | --- | --- | --- | --- | --- | --- | --- | --- | --- | --- | --- | --- | --- | --- | --- | --- | --- | --- | --- | --- | --- | --- | --- | --- | --- | --- | --- | --- | --- | --- | --- | --- | --- | --- | --- | --- | --- | --- | --- | --- | --- | --- | --- | --- | --- | --- | --- | --- | --- | --- | --- | --- | --- | --- | --- | --- | --- | --- | --- | --- | --- | --- | --- | --- | --- | --- | --- | --- | --- | --- | --- | --- | --- | --- | --- | --- | --- | --- | --- | --- | --- | --- | --- | --- | --- | --- | --- | --- | --- | --- | --- | --- | --- | --- | --- | --- | --- | --- | --- | --- | --- | --- | --- | --- | --- | --- | --- | --- | --- | --- | --- | --- | --- | --- | --- | --- | --- | --- | --- | --- | --- | --- | --- | --- | --- | --- | --- | --- | --- | --- | --- | --- | --- | --- | --- | --- | --- | --- | --- | --- | --- | --- | --- | --- | --- | --- | --- | --- | --- | --- | --- | --- | --- | --- | --- | --- | --- | --- | --- | --- | --- | --- | --- | --- | --- | --- | --- | --- | --- | --- | --- | --- | --- | --- | --- | --- | --- | --- | --- | --- | --- | --- | --- | --- | --- | --- | --- | --- | --- | --- | --- | --- | --- | --- | --- | --- | --- | --- | --- | --- | --- | --- | --- | --- | --- | --- | --- | --- | --- | --- | --- | --- | --- | --- | --- | --- | --- | --- | --- | --- | --- | --- | --- | --- | --- | --- | --- | --- | --- | --- | --- | --- | --- | --- | --- | --- | --- | --- | --- | --- | --- | --- | --- | --- | --- | --- | --- | --- | --- | --- | --- | --- | --- | --- | --- | --- | --- | --- | --- | --- | --- | --- | --- | --- | --- | --- | --- | --- | --- | --- | --- | --- | --- | --- | --- | --- | --- | --- | --- | --- | --- | --- | --- | --- | --- | --- | --- | --- | --- | --- | --- | --- | --- | --- | --- | --- | --- | --- | --- | --- | --- | --- | --- | --- | --- | --- | --- | --- | --- | --- | --- | --- | --- | --- | --- | --- | --- | --- | --- | --- | --- | --- | --- | --- | --- | --- | --- | --- | --- | --- | --- | --- | --- | --- | --- | --- | --- | --- | --- | --- | --- | --- | --- | --- | --- | --- | --- | --- | --- | --- | --- | --- | --- | --- | --- | --- | --- | --- | --- | --- | --- | --- | --- | --- | --- | --- | --- | --- | --- | --- | --- | --- | --- | --- | --- | --- | --- | --- | --- | --- | --- | --- | --- | --- | --- | --- | --- | --- | --- | --- | --- | --- | --- | --- | --- | --- | --- | --- | --- | --- | --- | --- | --- | --- | --- | --- | --- | --- | --- | --- | --- | --- | --- | --- | --- | --- | --- | --- | --- | --- | --- | --- | --- | --- | --- | --- | --- | --- | --- | --- | --- | --- | --- | --- | --- | --- | --- | --- | --- | --- | --- | --- | --- | --- | --- | --- | --- | --- | --- | --- | --- | --- | --- | --- | --- | --- | --- | --- | --- | --- | --- | --- | --- | --- | --- | --- | --- | --- | --- | --- | --- | --- | --- | --- | --- | --- | --- | --- | --- | --- | --- | --- | --- | --- | --- | --- | --- | --- | --- | --- | --- | --- | --- | --- | --- | --- | --- | --- | --- | --- | --- | --- | --- | --- | --- | --- | --- | --- | --- | --- | --- | --- | --- | --- | --- | --- | --- | --- | --- | --- | --- | --- | --- | --- | --- | --- | --- | --- | --- | --- | --- | --- | --- | --- | --- | --- | --- | --- | --- | --- | --- | --- | --- | --- | --- | --- | --- | --- | --- | --- | --- | --- | --- | --- | --- | --- | --- | --- | --- | --- | --- | --- | --- | --- | --- | --- | --- | --- | --- | --- | --- | --- | --- | --- | --- | --- | --- | --- | --- | --- | --- | --- | --- | --- | --- | --- | --- | --- | --- | --- | --- | --- | --- | --- | --- | --- | --- | --- | --- | --- | --- | --- | --- | --- | --- | --- | --- | --- | --- | --- | --- | --- | --- | --- | --- | --- | --- | --- | --- | --- | --- | --- | --- | --- | --- | --- | --- | --- | --- | --- | --- | --- | --- | --- | --- | --- | --- | --- | --- | --- | --- | --- | --- | --- | --- | --- | --- | --- | --- | --- | --- | --- | --- | --- | --- | --- | --- | --- | --- | --- | --- | --- | --- | --- | --- | --- | --- | --- | --- | --- | --- | --- | --- | --- | --- | --- | --- | --- | --- | --- | --- | --- | --- | --- | --- | --- | --- | --- | --- | --- | --- | --- | --- | --- | --- | --- | --- | --- | --- | --- | --- | --- | --- | --- | --- | --- | --- | --- | --- | --- | --- | --- | --- | --- | --- | --- | --- | --- | --- | --- | --- | --- | --- | --- | --- | --- | --- | --- | --- | --- | --- | --- | --- | --- | --- | --- | --- | --- | --- | --- | --- | --- | --- | --- | --- | --- | --- | --- | --- | --- | --- | --- | --- | --- | --- | --- | --- | --- | --- | --- | --- | --- | --- | --- | --- | --- | --- | --- | --- | --- | --- | --- | --- | --- | --- | --- | --- | --- | --- | --- | --- | --- | --- | --- | --- | --- | --- | --- | --- | --- | --- | --- | --- | --- | --- | --- | --- | --- | --- | --- | --- | --- | --- | --- | --- | --- | --- | --- | --- | --- | --- | --- | --- | --- | --- | --- | --- | --- | --- | --- | --- | --- | --- | --- | --- | --- | --- | --- | --- | --- | --- | --- | --- | --- | --- | --- | --- | --- | --- | --- | --- | --- | --- | --- | --- | --- | --- | --- | --- | --- | --- | --- | --- | --- | --- | --- | --- | --- | --- | --- | --- | --- | --- | --- | --- | --- | --- | --- | --- | --- | --- | --- | --- | --- | --- | --- | --- | --- | --- | --- | --- | --- | --- | --- | --- | --- | --- | --- | --- | --- | --- | --- | --- | --- | --- | --- | --- | --- | --- | --- | --- | --- | --- | --- | --- | --- | --- | --- | --- | --- | --- | --- | --- | --- | --- | --- | --- | --- | --- | --- | --- | --- | --- | --- | --- | --- | --- | --- | --- | --- | --- | --- | --- | --- | --- | --- | --- | --- | --- | --- | --- | --- | --- | --- | --- | --- | --- | --- | --- | --- | --- | --- | --- | --- | --- | --- | --- | --- | --- | --- | --- | --- | --- | --- | --- | --- | --- | --- | --- | --- | --- | --- | --- | --- | --- | --- | --- | --- | --- | --- | --- | --- | --- | --- | --- | --- | --- | --- | --- | --- | --- | --- | --- | --- | --- | --- | --- | --- | --- | --- | --- | --- | --- | --- | --- | --- | --- | --- | --- | --- | --- | --- | --- | --- | --- | --- | --- | --- | --- | --- | --- | --- | --- | --- | --- | --- | --- | --- | --- | --- | --- | --- | --- | --- | --- | --- | --- | --- | --- | --- | --- | --- | --- | --- | --- | --- | --- | --- | --- | --- | --- | --- | --- | --- | --- | --- | --- | --- | --- | --- | --- | --- | --- | --- | --- | --- | --- | --- | --- | --- | --- | --- | --- | --- | --- | --- | --- | --- | --- | --- | --- | --- | --- | --- | --- | --- | --- | --- | --- | --- | --- | --- | --- | --- | --- | --- | --- | --- | --- | --- | --- | --- | --- | --- | --- | --- | --- | --- | --- | --- | --- | --- | --- | --- | --- | --- | --- | --- | --- | --- | --- | --- | --- | --- | --- | --- | --- | --- | --- | --- | --- | --- | --- | --- | --- | --- | --- | --- | --- | --- | --- | --- | --- | --- | --- | --- | --- | --- | --- | --- | --- | --- | --- | --- | --- | --- | --- | --- | --- | --- | --- | --- | --- | --- | --- | --- | --- | --- | --- | --- | --- | --- | --- | --- | --- | --- | --- | --- | --- | --- | --- | --- | --- | --- | --- | --- | --- | --- | --- | --- | --- | --- | --- | --- | --- | --- | --- | --- | --- | --- | --- | --- | --- | --- | --- | --- | --- | --- | --- | --- | --- | --- | --- | --- | --- | --- | --- | --- | --- | --- | --- | --- | --- | --- | --- | --- | --- | --- | --- | --- | --- | --- | --- | --- | --- | --- | --- | --- | --- | --- | --- | --- | --- | --- | --- | --- | --- | --- | --- | --- | --- | --- | --- | --- | --- | --- | --- | --- | --- | --- | --- | --- | --- | --- | --- | --- | --- | --- | --- | --- | --- | --- | --- | --- | --- | --- | --- | --- | --- | --- | --- | --- | --- | --- | --- | --- | --- | --- | --- | --- | --- | --- | --- | --- | --- | --- | --- | --- | --- | --- | --- | --- | --- | --- | --- | --- | --- | --- | --- | --- | --- | --- | --- | --- | --- | --- | --- | --- | --- | --- | --- | --- | --- | --- | --- | --- | --- | --- | --- | --- | --- | --- | --- | --- | --- | --- | --- | --- | --- | --- | --- | --- | --- | --- | --- | --- | --- | --- | --- | --- | --- | --- | --- | --- | --- | --- | --- | --- | --- | --- | --- | --- | --- | --- | --- | --- | --- | --- | --- | --- | --- | --- | --- | --- | --- | --- | --- | --- | --- | --- | --- | --- | --- | --- | --- | --- | --- | --- | --- | --- | --- | --- | --- | --- | --- | --- | --- | --- | --- | --- | --- | --- | --- | --- | --- | --- | --- | --- | --- | --- | --- | --- | --- | --- | --- | --- | --- | --- | --- | --- | --- | --- | --- | --- | --- | --- | --- | --- | --- | --- | --- | --- | --- | --- | --- | --- | --- | --- | --- | --- | --- | --- | --- | --- | --- | --- | --- | --- | --- | --- | --- | --- | --- | --- | --- | --- | --- | --- | --- | --- | --- | --- | --- | --- | --- | --- | --- | --- | --- | --- | --- | --- | --- | --- | --- | --- | --- | --- | --- | --- | --- | --- | --- | --- | --- | --- | --- | --- | --- | --- | --- | --- | --- | --- | --- | --- | --- | --- | --- | --- | --- | --- | --- | --- | --- | --- | --- | --- | --- | --- | --- | --- | --- | --- | --- | --- | --- | --- | --- | --- | --- | --- | --- | --- | --- | --- | --- | --- | --- | --- | --- | --- | --- | --- | --- | --- | --- | --- | --- | --- | --- | --- | --- | --- | --- | --- | --- | --- | --- | --- | --- | --- | --- | --- | --- | --- | --- | --- | --- | --- | --- | --- | --- | --- | --- | --- | --- | --- | --- | --- | --- | --- | --- | --- | --- | --- | --- | --- | --- | --- | --- | --- | --- | --- | --- | --- | --- | --- | --- | --- | --- | --- | --- | --- | --- | --- | --- | --- | --- | --- | --- | --- | --- | --- | --- | --- | --- | --- | --- | --- | --- | --- | --- | --- | --- | --- | --- | --- | --- | --- | --- | --- | --- | --- | --- | --- | --- | --- | --- | --- | --- | --- | --- | --- | --- | --- | --- | --- | --- | --- | --- | --- | --- | --- | --- | --- | --- | --- | --- | --- | --- | --- | --- | --- | --- | --- | --- | --- | --- | --- | --- | --- | --- | --- | --- | --- | --- | --- | --- | --- | --- | --- | --- | --- | --- | --- | --- | --- | --- | --- | --- | --- | --- | --- | --- | --- | --- | --- | --- | --- | --- | --- | --- | --- | --- | --- | --- | --- | --- | --- | --- | --- | --- | --- | --- | --- | --- | --- | --- | --- | --- | --- | --- | --- | --- | --- | --- | --- | --- | --- | --- | --- | --- | --- | --- | --- | --- | --- | --- | --- | --- | --- | --- | --- | --- | --- | --- | --- | --- | --- | --- | --- | --- | --- | --- | --- | --- | --- | --- | --- | --- | --- | --- | --- | --- | --- | --- | --- | --- | --- | --- | --- | --- | --- | --- | --- | --- | --- | --- | --- | --- | --- | --- | --- | --- | --- | --- | --- | --- | --- | --- | --- | --- | --- | --- | --- | --- | --- | --- | --- | --- | --- | --- | --- | --- | --- | --- | --- | --- | --- | --- | --- | --- | --- | --- | --- | --- | --- | --- | --- | --- | --- | --- | --- | --- | --- | --- | --- | --- | --- | --- | --- | --- | --- | --- | --- | --- | --- | --- | --- | --- | --- | --- | --- | --- | --- | --- | --- | --- | --- | --- | --- | --- | --- | --- | --- | --- | --- | --- | --- | --- | --- | --- | --- | --- | --- | --- | --- | --- | --- | --- | --- | --- | --- | --- | --- | --- | --- | --- | --- | --- | --- | --- | --- | --- | --- | --- | --- | --- | --- | --- | --- | --- | --- | --- | --- | --- | --- | --- | --- | --- | --- | --- | --- | --- | --- | --- | --- | --- | --- | --- | --- | --- | --- | --- | --- | --- | --- | --- | --- | --- | --- | --- | --- | --- | --- | --- | --- | --- | --- | --- | --- | --- | --- | --- | --- | --- | --- | --- | --- | --- | --- | --- | --- | --- | --- | --- | --- | --- | --- | --- | --- | --- | --- | --- | --- | --- | --- | --- | --- | --- | --- | --- | --- | --- | --- | --- | --- | --- | --- | --- | --- | --- | --- | --- | --- | --- | --- | --- | --- | --- | --- | --- | --- | --- | --- | --- | --- | --- | --- | --- | --- | --- | --- | --- | --- | --- | --- | --- | --- | --- | --- | --- | --- | --- | --- | --- | --- | --- | --- | --- | --- | --- | --- | --- | --- | --- | --- | --- | --- | --- | --- | --- | --- | --- | --- | --- | --- | --- | --- | --- | --- | --- | --- | --- | --- | --- | --- | --- | --- | --- | --- | --- | --- | --- | --- | --- | --- | --- | --- | --- | --- | --- | --- | --- | --- | --- | --- | --- | --- | --- | --- | --- | --- | --- | --- | --- | --- | --- | --- | --- | --- | --- | --- | --- | --- | --- | --- | --- | --- | --- | --- | --- | --- | --- | --- | --- | --- | --- | --- | --- | --- | --- | --- | --- | --- | --- | --- | --- | --- | --- | --- | --- | --- | --- | --- | --- | --- | --- | --- | --- | --- | --- | --- | --- | --- | --- | --- | --- | --- | --- | --- | --- | --- | --- | --- | --- | --- | --- | --- | --- | --- | --- | --- | --- | --- | --- | --- | --- | --- | --- | --- | --- | --- | --- | --- | --- | --- | --- | --- | --- | --- | --- | --- | --- | --- | --- | --- | --- | --- | --- | --- | --- | --- | --- | --- | --- | --- | --- | --- | --- | --- | --- | --- | --- | --- | --- | --- | --- | --- | --- | --- | --- | --- | --- | --- | --- | --- | --- | --- | --- | --- | --- | --- | --- | --- | --- | --- | --- | --- | --- | --- | --- | --- | --- | --- | --- | --- | --- | --- | --- | --- | --- | --- | --- | --- | --- | --- | --- | --- | --- | --- | --- | --- | --- | --- | --- | --- | --- | --- | --- | --- | --- | --- | --- | --- | --- | --- | --- | --- | --- | --- | --- | --- | --- | --- | --- | --- | --- | --- | --- | --- | --- | --- | --- | --- | --- | --- | --- | --- | --- | --- | --- | --- | --- | --- | --- | --- | --- | --- | --- | --- | --- | --- | --- | --- | --- | --- | --- | --- | --- | --- | --- | --- | --- | --- | --- | --- | --- | --- | --- | --- | --- | --- | --- | --- | --- | --- | --- | --- | --- | --- | --- | --- | --- | --- | --- | --- | --- | --- | --- | --- | --- | --- | --- | --- | --- | --- | --- | --- | --- | --- | --- | --- | --- | --- | --- | --- | --- | --- | --- | --- | --- | --- | --- | --- | --- | --- | --- | --- | --- | --- | --- | --- | --- | --- | --- | --- | --- | --- | --- | --- | --- | --- | --- | --- | --- | --- | --- | --- | --- | --- | --- | --- | --- | --- | --- | --- | --- | --- | --- | --- | --- | --- | --- | --- | --- | --- | --- | --- | --- | --- | --- | --- | --- | --- | --- | --- | --- | --- | --- | --- | --- | --- | --- | --- | --- | --- | --- | --- | --- | --- | --- | --- | --- | --- | --- | --- | --- | --- | --- | --- | --- | --- | --- | --- | --- | --- | --- | --- | --- | --- | --- | --- | --- | --- | --- | --- | --- | --- | --- | --- | --- | --- | --- | --- | --- | --- | --- | --- | --- | --- | --- | --- | --- | --- | --- | --- | --- | --- | --- | --- | --- | --- | --- | --- | --- | --- | --- | --- | --- | --- | --- | --- | --- | --- | --- | --- | --- | --- | --- | --- | --- | --- | --- | --- | --- | --- | --- | --- | --- | --- | --- | --- | --- | --- | --- | --- | --- | --- | --- | --- | --- | --- | --- | --- | --- | --- | --- | --- | --- | --- | --- | --- | --- | --- | --- | --- | --- | --- | --- | --- | --- | --- | --- | --- | --- | --- | --- | --- | --- | --- | --- | --- | --- | --- | --- | --- | --- | --- | --- | --- | --- | --- | --- | --- | --- | --- | --- | --- | --- | --- | --- | --- | --- | --- | --- | --- | --- | --- | --- | --- | --- | --- | --- | --- | --- | --- | --- | --- | --- | --- | --- | --- | --- | --- | --- | --- | --- | --- | --- | --- | --- | --- | --- | --- | --- | --- | --- | --- | --- | --- | --- | --- | --- | --- | --- | --- | --- | --- | --- | --- | --- | --- | --- | --- | --- | --- | --- | --- | --- | --- | --- | --- | --- | --- | --- | --- | --- | --- | --- | --- | --- | --- | --- | --- | --- | --- | --- | --- | --- | --- | --- | --- | --- | --- | --- | --- | --- | --- | --- | --- | --- | --- | --- | --- | --- | --- | --- | --- | --- | --- | --- | --- | --- | --- | --- | --- | --- | --- | --- | --- | --- | --- | --- | --- | --- | --- | --- | --- | --- | --- | --- | --- | --- | --- | --- | --- | --- | --- | --- | --- | --- | --- | --- | --- | --- | --- | --- | --- | --- | --- | --- | --- | --- | --- | --- | --- | --- | --- | --- | --- | --- | --- | --- | --- | --- | --- | --- | --- | --- | --- | --- | --- | --- | --- | --- | --- | --- | --- | --- | --- | --- | --- | --- | --- | --- | --- | --- | --- | --- | --- | --- | --- | --- | --- | --- | --- | --- | --- | --- | --- | --- | --- | --- | --- | --- | --- | --- | --- | --- | --- | --- | --- | --- | --- | --- | --- | --- | --- | --- | --- | --- | --- | --- | --- | --- | --- | --- | --- | --- | --- | --- | --- | --- | --- | --- | --- | --- | --- | --- | --- | --- | --- | --- | --- | --- | --- | --- | --- | --- | --- | --- | --- | --- | --- | --- | --- | --- | --- | --- | --- | --- | --- | --- | --- | --- | --- | --- | --- | --- | --- | --- | --- | --- | --- | --- | --- | --- | --- | --- | --- | --- | --- | --- | --- | --- | --- | --- | --- | --- | --- | --- | --- | --- | --- | --- | --- | --- | --- | --- | --- | --- | --- | --- | --- | --- | --- | --- | --- | --- | --- | --- | --- | --- | --- | --- | --- | --- | --- | --- | --- | --- | --- | --- | --- | --- | --- | --- | --- | --- | --- | --- | --- | --- | --- | --- | --- | --- | --- | --- | --- | --- | --- | --- | --- | --- | --- | --- | --- | --- | --- | --- | --- | --- | --- | --- | --- | --- | --- | --- | --- | --- | --- | --- | --- | --- | --- | --- | --- | --- | --- | --- | --- | --- | --- | --- | --- | --- | --- | --- | --- | --- | --- | --- | --- | --- | --- | --- | --- | --- | --- | --- | --- | --- | --- | --- | --- | --- | --- | --- | --- | --- | --- | --- | --- | --- | --- | --- | --- | --- | --- | --- | --- | --- | --- | --- | --- | --- | --- | --- | --- | --- | --- | --- | --- | --- | --- | --- | --- | --- | --- | --- | --- | --- | --- | --- | --- | --- | --- | --- | --- | --- | --- | --- | --- | --- | --- | --- | --- | --- | --- | --- | --- | --- | --- | --- | --- | --- | --- | --- | --- | --- | --- | --- | --- | --- | --- | --- | --- | --- | --- | --- | --- | --- | --- | --- | --- | | DAVID Functional Annotation Clustering      |  | | --- | | Functional Annotation Clustering | | Help and Manual | | Current Gene List: DavidMay18\_TPM87 | | Current Background: DavidMay18\_TPM8\_bg | | 969 DAVID IDs |  - Options          Classification Stringency    Custom   Lowest   Low   Medium   High   Highest   - |  |  |  |     | --- | --- | --- |     | Kappa Similarity | Similarity Term Overlap 3 4 5 6 7 8 9 10 | Similarity Threshold 0.20 0.25 0.30 0.35 0.40 0.45 0.50 0.55 0.60 0.65 0.70 0.75 0.80 0.85 0.90 0.95 1.00 |     | Classification | Initial Group Membership 2 3 4 5 6 7 8 9 10 | Final Group Membership 2 3 4 5 6 7 8 9 10 | Multiple Linkage Threshold 0.00 0.05 0.10 0.15 0.20 0.25 0.30 0.35 0.40 0.45 0.50 0.55 0.60 0.65 0.70 0.75 0.80 0.85 0.90 0.95 1.00 |  |     | Enrichment Thresholds | EASE |  |  |  |     |     | Display | Fold Change | Bonferroni | Benjamini | FDR | LT,PH,PT |  |  |  |  | | --- | --- | --- | |  |  |  |     |  |  | | --- | --- | | 172 Cluster(s) | Download File |  | Annotation Cluster 1 | | Enrichment Score: 4.43 |  |  | Count | P\_Value | Benjamini | | --- | --- | --- | --- | --- | --- | --- | --- | |  | GOTERM\_BP\_5 | imaginal disc development | **RT** |  | 117 | 7.3E-7 | 1.6E-3 | |  | GOTERM\_BP\_5 | wing disc development | **RT** |  | 95 | 9.2E-7 | 1.0E-3 | |  | GOTERM\_BP\_5 | morphogenesis of an epithelium | **RT** |  | 115 | 1.2E-6 | 9.0E-4 | |  | GOTERM\_BP\_5 | animal organ development | **RT** |  | 208 | 1.3E-6 | 7.1E-4 | |  | GOTERM\_BP\_5 | tube morphogenesis | **RT** |  | 97 | 2.4E-5 | 8.8E-3 | |  | GOTERM\_BP\_5 | epithelial tube morphogenesis | **RT** |  | 93 | 2.5E-5 | 7.9E-3 | |  | GOTERM\_BP\_5 | instar larval or pupal development | **RT** |  | 110 | 3.0E-5 | 8.3E-3 | |  | GOTERM\_BP\_5 | organ morphogenesis | **RT** |  | 143 | 3.1E-5 | 7.6E-3 | |  | GOTERM\_BP\_5 | instar larval or pupal morphogenesis | **RT** |  | 98 | 5.5E-5 | 1.2E-2 | |  | GOTERM\_BP\_5 | post-embryonic morphogenesis | **RT** |  | 99 | 7.6E-5 | 1.4E-2 | |  | GOTERM\_BP\_5 | imaginal disc morphogenesis | **RT** |  | 83 | 8.6E-5 | 1.4E-2 | |  | GOTERM\_BP\_5 | post-embryonic organ morphogenesis | **RT** |  | 83 | 8.6E-5 | 1.4E-2 | |  | GOTERM\_BP\_5 | imaginal disc-derived appendage development | **RT** |  | 75 | 1.2E-4 | 1.7E-2 | |  | GOTERM\_BP\_5 | appendage morphogenesis | **RT** |  | 74 | 1.2E-4 | 1.7E-2 | |  | GOTERM\_BP\_5 | imaginal disc-derived appendage morphogenesis | **RT** |  | 74 | 1.2E-4 | 1.7E-2 | |  | GOTERM\_BP\_5 | post-embryonic appendage morphogenesis | **RT** |  | 72 | 2.0E-4 | 2.5E-2 | |  | GOTERM\_BP\_5 | post-embryonic organ development | **RT** |  | 90 | 3.0E-4 | 3.4E-2 | |  | GOTERM\_BP\_5 | wing disc morphogenesis | **RT** |  | 67 | 4.8E-4 | 4.7E-2 | |  | GOTERM\_BP\_5 | imaginal disc-derived wing morphogenesis | **RT** |  | 65 | 5.9E-4 | 5.5E-2 | | Annotation Cluster 2 | | Enrichment Score: 3.13 |  |  | Count | P\_Value | Benjamini | | --- | --- | --- | --- | --- | --- | --- | --- | |  | GOTERM\_BP\_5 | germ cell development | **RT** |  | 120 | 9.8E-6 | 4.3E-3 | |  | GOTERM\_BP\_5 | oogenesis | **RT** |  | 106 | 6.6E-5 | 1.3E-2 | |  | GOTERM\_BP\_5 | female gamete generation | **RT** |  | 106 | 1.2E-4 | 1.8E-2 | |  | GOTERM\_BP\_5 | cell migration | **RT** |  | 56 | 3.1E-4 | 3.4E-2 | |  | GOTERM\_BP\_5 | gamete generation | **RT** |  | 125 | 3.3E-4 | 3.5E-2 | |  | GOTERM\_BP\_5 | ameboidal-type cell migration | **RT** |  | 38 | 1.0E-3 | 8.6E-2 | |  | GOTERM\_BP\_5 | epithelial cell migration | **RT** |  | 35 | 1.0E-3 | 8.4E-2 | |  | GOTERM\_BP\_5 | ovarian follicle cell migration | **RT** |  | 30 | 2.2E-3 | 1.4E-1 | |  | GOTERM\_BP\_5 | epithelial cell development | **RT** |  | 55 | 2.5E-3 | 1.5E-1 | |  | GOTERM\_BP\_5 | border follicle cell migration | **RT** |  | 28 | 3.8E-3 | 2.0E-1 | |  | GOTERM\_BP\_5 | columnar/cuboidal epithelial cell development | **RT** |  | 51 | 4.3E-3 | 2.1E-1 | |  | GOTERM\_BP\_5 | ovarian follicle cell development | **RT** |  | 51 | 4.3E-3 | 2.1E-1 | |  | GOTERM\_BP\_5 | epithelial cell differentiation | **RT** |  | 58 | 6.0E-3 | 2.4E-1 | | Annotation Cluster 3 | | Enrichment Score: 2.39 |  |  | Count | P\_Value | Benjamini | | --- | --- | --- | --- | --- | --- | --- | --- | |  | GOTERM\_BP\_5 | open tracheal system development | **RT** |  | 41 | 6.9E-4 | 6.2E-2 | |  | GOTERM\_BP\_5 | respiratory system development | **RT** |  | 42 | 1.1E-3 | 8.8E-2 | |  | GOTERM\_BP\_5 | branching morphogenesis of an epithelial tube | **RT** |  | 16 | 1.1E-2 | 3.8E-1 | |  | GOTERM\_BP\_5 | branching involved in open tracheal system development | **RT** |  | 16 | 1.1E-2 | 3.8E-1 | |  | GOTERM\_BP\_5 | morphogenesis of a branching epithelium | **RT** |  | 16 | 1.1E-2 | 3.8E-1 | | Annotation Cluster 4 | | Enrichment Score: 2.28 |  |  | Count | P\_Value | Benjamini | | --- | --- | --- | --- | --- | --- | --- | --- | |  | GOTERM\_BP\_5 | leg disc development | **RT** |  | 21 | 1.6E-3 | 1.2E-1 | |  | GOTERM\_BP\_5 | imaginal disc-derived leg morphogenesis | **RT** |  | 17 | 9.4E-3 | 3.4E-1 | |  | GOTERM\_BP\_5 | leg disc morphogenesis | **RT** |  | 17 | 9.4E-3 | 3.4E-1 | | Annotation Cluster 5 | | Enrichment Score: 2.12 |  |  | Count | P\_Value | Benjamini | | --- | --- | --- | --- | --- | --- | --- | --- | |  | GOTERM\_BP\_5 | cell surface receptor signaling pathway involved in cell-cell signaling | **RT** |  | 29 | 2.2E-3 | 1.4E-1 | |  | GOTERM\_BP\_5 | Wnt signaling pathway | **RT** |  | 28 | 3.2E-3 | 1.7E-1 | |  | GOTERM\_BP\_5 | cell-cell signaling by wnt | **RT** |  | 28 | 3.8E-3 | 2.0E-1 | |  | GOTERM\_BP\_5 | regulation of Wnt signaling pathway | **RT** |  | 17 | 1.3E-1 | 8.1E-1 | | Annotation Cluster 6 | | Enrichment Score: 2.01 |  |  | Count | P\_Value | Benjamini | | --- | --- | --- | --- | --- | --- | --- | --- | |  | GOTERM\_MF\_5 | GTP binding | **RT** |  | 33 | 7.3E-3 | 8.8E-1 | |  | GOTERM\_MF\_5 | guanyl nucleotide binding | **RT** |  | 33 | 1.1E-2 | 8.1E-1 | |  | GOTERM\_MF\_5 | guanyl ribonucleotide binding | **RT** |  | 33 | 1.1E-2 | 8.1E-1 | | Annotation Cluster 7 | | Enrichment Score: 1.89 |  |  | Count | P\_Value | Benjamini | | --- | --- | --- | --- | --- | --- | --- | --- | |  | GOTERM\_BP\_5 | single-organism carbohydrate catabolic process | **RT** |  | 12 | 1.8E-3 | 1.2E-1 | |  | GOTERM\_BP\_5 | oxidoreduction coenzyme metabolic process | **RT** |  | 12 | 1.5E-2 | 4.2E-1 | |  | GOTERM\_BP\_5 | glycolytic process | **RT** |  | 8 | 2.2E-2 | 5.0E-1 | |  | GOTERM\_BP\_5 | pyridine nucleotide metabolic process | **RT** |  | 11 | 2.4E-2 | 5.1E-1 | |  | GOTERM\_BP\_5 | nucleoside diphosphate metabolic process | **RT** |  | 9 | 2.6E-2 | 5.2E-1 | | Annotation Cluster 8 | | Enrichment Score: 1.83 |  |  | Count | P\_Value | Benjamini | | --- | --- | --- | --- | --- | --- | --- | --- | |  | GOTERM\_BP\_5 | regulation of signal transduction | **RT** |  | 117 | 4.3E-3 | 2.1E-1 | |  | GOTERM\_BP\_5 | regulation of intracellular signal transduction | **RT** |  | 64 | 4.9E-3 | 2.3E-1 | |  | GOTERM\_BP\_5 | intracellular signal transduction | **RT** |  | 107 | 6.3E-3 | 2.5E-1 | |  | GOTERM\_BP\_5 | positive regulation of intracellular signal transduction | **RT** |  | 32 | 2.1E-2 | 5.0E-1 | |  | GOTERM\_BP\_5 | positive regulation of signal transduction | **RT** |  | 51 | 3.5E-2 | 5.9E-1 | |  | GOTERM\_BP\_5 | positive regulation of cell communication | **RT** |  | 52 | 1.1E-1 | 7.8E-1 | | Annotation Cluster 9 | | Enrichment Score: 1.78 |  |  | Count | P\_Value | Benjamini | | --- | --- | --- | --- | --- | --- | --- | --- | |  | GOTERM\_BP\_5 | regulation of cell shape | **RT** |  | 29 | 3.2E-3 | 1.8E-1 | |  | GOTERM\_BP\_5 | actin filament organization | **RT** |  | 29 | 3.4E-2 | 5.9E-1 | |  | GOTERM\_BP\_5 | actin cytoskeleton organization | **RT** |  | 43 | 4.1E-2 | 6.2E-1 | | Annotation Cluster 10 | | Enrichment Score: 1.68 |  |  | Count | P\_Value | Benjamini | | --- | --- | --- | --- | --- | --- | --- | --- | |  | GOTERM\_CC\_5 | cytoskeletal part | **RT** |  | 84 | 1.5E-2 | 6.7E-1 | |  | GOTERM\_CC\_5 | microtubule cytoskeleton | **RT** |  | 75 | 1.7E-2 | 6.8E-1 | |  | GOTERM\_CC\_5 | cytoskeleton | **RT** |  | 94 | 1.7E-2 | 6.4E-1 | |  | GOTERM\_CC\_5 | microtubule associated complex | **RT** |  | 54 | 4.5E-2 | 8.0E-1 | | Annotation Cluster 11 | | Enrichment Score: 1.64 |  |  | Count | P\_Value | Benjamini | | --- | --- | --- | --- | --- | --- | --- | --- | |  | GOTERM\_BP\_5 | morphogenesis of embryonic epithelium | **RT** |  | 25 | 1.1E-2 | 3.9E-1 | |  | GOTERM\_BP\_5 | embryonic morphogenesis | **RT** |  | 38 | 1.2E-2 | 3.9E-1 | |  | GOTERM\_BP\_5 | morphogenesis of a polarized epithelium | **RT** |  | 21 | 2.4E-2 | 5.1E-1 | |  | GOTERM\_BP\_5 | embryo development ending in birth or egg hatching | **RT** |  | 33 | 4.3E-2 | 6.3E-1 | |  | GOTERM\_BP\_5 | dorsal closure | **RT** |  | 21 | 4.3E-2 | 6.3E-1 | | Annotation Cluster 12 | | Enrichment Score: 1.58 |  |  | Count | P\_Value | Benjamini | | --- | --- | --- | --- | --- | --- | --- | --- | |  | GOTERM\_CC\_5 | polytene chromosome | **RT** |  | 47 | 1.4E-4 | 3.6E-2 | |  | GOTERM\_CC\_5 | chromosome | **RT** |  | 75 | 6.1E-3 | 5.5E-1 | |  | GOTERM\_CC\_5 | nuclear chromosome part | **RT** |  | 25 | 1.2E-2 | 7.3E-1 | |  | GOTERM\_CC\_5 | chromatin | **RT** |  | 34 | 3.0E-2 | 7.4E-1 | |  | GOTERM\_CC\_5 | nuclear chromatin | **RT** |  | 19 | 4.7E-2 | 7.8E-1 | |  | GOTERM\_CC\_5 | nuclear chromosome | **RT** |  | 28 | 5.8E-2 | 7.9E-1 | |  | GOTERM\_CC\_5 | chromosomal part | **RT** |  | 52 | 8.1E-2 | 8.4E-1 | |  | GOTERM\_CC\_5 | chromosomal region | **RT** |  | 27 | 2.2E-1 | 9.5E-1 | |  | GOTERM\_BP\_5 | chromatin organization | **RT** |  | 45 | 3.8E-1 | 9.6E-1 | | Annotation Cluster 13 | | Enrichment Score: 1.54 |  |  | Count | P\_Value | Benjamini | | --- | --- | --- | --- | --- | --- | --- | --- | |  | GOTERM\_BP\_5 | female meiotic division | **RT** |  | 13 | 5.0E-3 | 2.3E-1 | |  | GOTERM\_BP\_5 | nuclear division | **RT** |  | 51 | 5.4E-3 | 2.3E-1 | |  | GOTERM\_BP\_5 | meiotic cell cycle process | **RT** |  | 25 | 2.4E-2 | 5.1E-1 | |  | GOTERM\_BP\_5 | mitotic nuclear division | **RT** |  | 35 | 2.5E-2 | 5.2E-1 | |  | GOTERM\_BP\_5 | meiotic chromosome segregation | **RT** |  | 11 | 3.1E-2 | 5.7E-1 | |  | GOTERM\_BP\_5 | female meiosis chromosome segregation | **RT** |  | 8 | 3.1E-2 | 5.7E-1 | |  | GOTERM\_BP\_5 | mitotic cell cycle process | **RT** |  | 61 | 3.3E-2 | 5.8E-1 | |  | GOTERM\_BP\_5 | meiotic nuclear division | **RT** |  | 23 | 3.5E-2 | 5.8E-1 | |  | GOTERM\_BP\_5 | nuclear chromosome segregation | **RT** |  | 23 | 3.5E-2 | 5.8E-1 | |  | GOTERM\_BP\_5 | mitotic sister chromatid segregation | **RT** |  | 15 | 1.3E-1 | 8.1E-1 | |  | GOTERM\_BP\_5 | sister chromatid segregation | **RT** |  | 16 | 1.5E-1 | 8.3E-1 | | Annotation Cluster 14 | | Enrichment Score: 1.52 |  |  | Count | P\_Value | Benjamini | | --- | --- | --- | --- | --- | --- | --- | --- | |  | GOTERM\_BP\_5 | cell adhesion involved in heart morphogenesis | **RT** |  | 7 | 5.2E-3 | 2.3E-1 | |  | GOTERM\_BP\_5 | heart morphogenesis | **RT** |  | 9 | 3.5E-2 | 5.8E-1 | |  | GOTERM\_BP\_5 | heart development | **RT** |  | 17 | 5.1E-2 | 6.4E-1 | |  | GOTERM\_BP\_5 | circulatory system development | **RT** |  | 17 | 5.1E-2 | 6.4E-1 | |  | GOTERM\_BP\_5 | cardiovascular system development | **RT** |  | 17 | 5.1E-2 | 6.4E-1 | | Annotation Cluster 15 | | Enrichment Score: 1.38 |  |  | Count | P\_Value | Benjamini | | --- | --- | --- | --- | --- | --- | --- | --- | |  | GOTERM\_BP\_5 | nucleoside phosphate catabolic process | **RT** |  | 6 | 3.9E-3 | 2.0E-1 | |  | GOTERM\_BP\_5 | nucleotide catabolic process | **RT** |  | 5 | 1.5E-2 | 4.2E-1 | |  | GOTERM\_BP\_5 | purine-containing compound catabolic process | **RT** |  | 5 | 7.6E-2 | 7.2E-1 | |  | GOTERM\_BP\_5 | organophosphate catabolic process | **RT** |  | 7 | 1.2E-1 | 7.8E-1 | |  | GOTERM\_BP\_5 | nucleobase-containing compound catabolic process | **RT** |  | 15 | 2.3E-1 | 9.1E-1 | | Annotation Cluster 16 | | Enrichment Score: 1.31 |  |  | Count | P\_Value | Benjamini | | --- | --- | --- | --- | --- | --- | --- | --- | |  | GOTERM\_BP\_5 | cellularization | **RT** |  | 23 | 1.9E-3 | 1.3E-1 | |  | GOTERM\_BP\_5 | sperm individualization | **RT** |  | 12 | 1.5E-2 | 4.2E-1 | |  | GOTERM\_BP\_5 | spermatid differentiation | **RT** |  | 19 | 4.7E-2 | 6.4E-1 | |  | GOTERM\_BP\_5 | spermatid development | **RT** |  | 17 | 6.8E-2 | 7.0E-1 | |  | GOTERM\_BP\_5 | spermatogenesis | **RT** |  | 25 | 3.9E-1 | 9.6E-1 | |  | GOTERM\_BP\_5 | male gamete generation | **RT** |  | 25 | 3.9E-1 | 9.6E-1 | | Annotation Cluster 17 | | Enrichment Score: 1.27 |  |  | Count | P\_Value | Benjamini | | --- | --- | --- | --- | --- | --- | --- | --- | |  | GOTERM\_BP\_5 | eye development | **RT** |  | 60 | 3.8E-2 | 5.9E-1 | |  | GOTERM\_BP\_5 | sensory organ development | **RT** |  | 73 | 4.2E-2 | 6.2E-1 | |  | GOTERM\_BP\_5 | compound eye development | **RT** |  | 57 | 4.4E-2 | 6.2E-1 | |  | GOTERM\_BP\_5 | eye morphogenesis | **RT** |  | 48 | 6.0E-2 | 6.6E-1 | |  | GOTERM\_BP\_5 | sensory organ morphogenesis | **RT** |  | 48 | 6.0E-2 | 6.6E-1 | |  | GOTERM\_BP\_5 | regulation of organ morphogenesis | **RT** |  | 18 | 9.5E-2 | 7.6E-1 | | Annotation Cluster 18 | | Enrichment Score: 1.27 |  |  | Count | P\_Value | Benjamini | | --- | --- | --- | --- | --- | --- | --- | --- | |  | GOTERM\_BP\_5 | cell adhesion involved in heart morphogenesis | **RT** |  | 7 | 5.2E-3 | 2.3E-1 | |  | GOTERM\_BP\_5 | heart morphogenesis | **RT** |  | 9 | 3.5E-2 | 5.8E-1 | |  | GOTERM\_BP\_5 | establishment of blood-brain barrier | **RT** |  | 6 | 4.4E-2 | 6.3E-1 | |  | GOTERM\_BP\_5 | cell-cell junction assembly | **RT** |  | 11 | 6.0E-2 | 6.7E-1 | |  | GOTERM\_BP\_5 | regulation of tube size, open tracheal system | **RT** |  | 10 | 6.0E-2 | 6.6E-1 | |  | GOTERM\_BP\_5 | establishment of glial blood-brain barrier | **RT** |  | 5 | 7.6E-2 | 7.2E-1 | |  | GOTERM\_BP\_5 | glial cell differentiation | **RT** |  | 7 | 1.7E-1 | 8.6E-1 | |  | GOTERM\_BP\_5 | glial cell development | **RT** |  | 6 | 1.8E-1 | 8.7E-1 | | Annotation Cluster 19 | | Enrichment Score: 1.26 |  |  | Count | P\_Value | Benjamini | | --- | --- | --- | --- | --- | --- | --- | --- | |  | GOTERM\_BP\_5 | actin cytoskeleton organization | **RT** |  | 43 | 4.1E-2 | 6.2E-1 | |  | GOTERM\_BP\_5 | regulation of actin cytoskeleton organization | **RT** |  | 19 | 4.7E-2 | 6.4E-1 | |  | GOTERM\_BP\_5 | regulation of cytoskeleton organization | **RT** |  | 24 | 8.5E-2 | 7.5E-1 | | Annotation Cluster 20 | | Enrichment Score: 1.25 |  |  | Count | P\_Value | Benjamini | | --- | --- | --- | --- | --- | --- | --- | --- | |  | GOTERM\_CC\_5 | cell cortex | **RT** |  | 30 | 2.4E-2 | 7.2E-1 | |  | GOTERM\_CC\_5 | cytoplasmic region | **RT** |  | 32 | 4.0E-2 | 7.9E-1 | |  | GOTERM\_CC\_5 | cell cortex part | **RT** |  | 13 | 1.9E-1 | 9.5E-1 | | Annotation Cluster 21 | | Enrichment Score: 1.23 |  |  | Count | P\_Value | Benjamini | | --- | --- | --- | --- | --- | --- | --- | --- | |  | GOTERM\_CC\_5 | intracellular organelle | **RT** |  | 535 | 1.3E-2 | 6.9E-1 | |  | GOTERM\_CC\_5 | intracellular organelle part | **RT** |  | 336 | 4.9E-2 | 7.7E-1 | |  | GOTERM\_CC\_5 | intracellular membrane-bounded organelle | **RT** |  | 449 | 3.2E-1 | 9.5E-1 | | Annotation Cluster 22 | | Enrichment Score: 1.15 |  |  | Count | P\_Value | Benjamini | | --- | --- | --- | --- | --- | --- | --- | --- | |  | GOTERM\_BP\_5 | morphogenesis of a polarized epithelium | **RT** |  | 21 | 2.4E-2 | 5.1E-1 | |  | GOTERM\_BP\_5 | dorsal closure | **RT** |  | 21 | 4.3E-2 | 6.3E-1 | |  | GOTERM\_BP\_5 | establishment of planar polarity | **RT** |  | 17 | 8.9E-2 | 7.6E-1 | |  | GOTERM\_BP\_5 | establishment of ommatidial planar polarity | **RT** |  | 12 | 1.1E-1 | 7.8E-1 | |  | GOTERM\_BP\_5 | ommatidial rotation | **RT** |  | 7 | 1.7E-1 | 8.6E-1 | | Annotation Cluster 23 | | Enrichment Score: 1.14 |  |  | Count | P\_Value | Benjamini | | --- | --- | --- | --- | --- | --- | --- | --- | |  | GOTERM\_BP\_5 | stress-activated protein kinase signaling cascade | **RT** |  | 20 | 5.3E-3 | 2.3E-1 | |  | GOTERM\_BP\_5 | stress-activated MAPK cascade | **RT** |  | 19 | 8.0E-3 | 3.0E-1 | |  | GOTERM\_BP\_5 | positive regulation of intracellular signal transduction | **RT** |  | 32 | 2.1E-2 | 5.0E-1 | |  | GOTERM\_BP\_5 | phosphorylation | **RT** |  | 90 | 2.2E-2 | 5.1E-1 | |  | GOTERM\_BP\_5 | regulation of protein modification process | **RT** |  | 47 | 5.0E-2 | 6.5E-1 | |  | GOTERM\_BP\_5 | MAPK cascade | **RT** |  | 28 | 5.0E-2 | 6.4E-1 | |  | GOTERM\_BP\_5 | signal transduction by protein phosphorylation | **RT** |  | 28 | 5.0E-2 | 6.4E-1 | |  | GOTERM\_BP\_5 | regulation of stress-activated protein kinase signaling cascade | **RT** |  | 13 | 5.8E-2 | 6.6E-1 | |  | GOTERM\_BP\_5 | regulation of stress-activated MAPK cascade | **RT** |  | 13 | 5.8E-2 | 6.6E-1 | |  | GOTERM\_BP\_5 | regulation of MAPK cascade | **RT** |  | 24 | 6.2E-2 | 6.7E-1 | |  | GOTERM\_BP\_5 | regulation of phosphate metabolic process | **RT** |  | 45 | 7.5E-2 | 7.2E-1 | |  | GOTERM\_BP\_5 | regulation of kinase activity | **RT** |  | 19 | 9.0E-2 | 7.5E-1 | |  | GOTERM\_BP\_5 | regulation of cellular protein metabolic process | **RT** |  | 76 | 1.0E-1 | 7.7E-1 | |  | GOTERM\_BP\_5 | regulation of protein metabolic process | **RT** |  | 79 | 1.5E-1 | 8.3E-1 | |  | GOTERM\_BP\_5 | positive regulation of phosphorus metabolic process | **RT** |  | 25 | 1.5E-1 | 8.3E-1 | |  | GOTERM\_BP\_5 | positive regulation of phosphate metabolic process | **RT** |  | 25 | 1.5E-1 | 8.3E-1 | |  | GOTERM\_BP\_5 | positive regulation of MAPK cascade | **RT** |  | 15 | 2.0E-1 | 8.8E-1 | |  | GOTERM\_BP\_5 | positive regulation of transferase activity | **RT** |  | 10 | 2.5E-1 | 9.2E-1 | |  | GOTERM\_BP\_5 | positive regulation of protein modification process | **RT** |  | 24 | 2.6E-1 | 9.2E-1 | |  | GOTERM\_BP\_5 | positive regulation of protein metabolic process | **RT** |  | 36 | 3.0E-1 | 9.4E-1 | |  | GOTERM\_BP\_5 | positive regulation of cellular protein metabolic process | **RT** |  | 34 | 3.2E-1 | 9.5E-1 | | Annotation Cluster 24 | | Enrichment Score: 1.06 |  |  | Count | P\_Value | Benjamini | | --- | --- | --- | --- | --- | --- | --- | --- | |  | GOTERM\_BP\_5 | regulation of Notch signaling pathway | **RT** |  | 22 | 4.7E-2 | 6.4E-1 | |  | GOTERM\_BP\_5 | negative regulation of Notch signaling pathway | **RT** |  | 9 | 7.4E-2 | 7.2E-1 | |  | GOTERM\_BP\_5 | Notch signaling pathway | **RT** |  | 20 | 1.9E-1 | 8.7E-1 | | Annotation Cluster 25 | | Enrichment Score: 1.03 |  |  | Count | P\_Value | Benjamini | | --- | --- | --- | --- | --- | --- | --- | --- | |  | GOTERM\_BP\_5 | positive regulation of macromolecule metabolic process | **RT** |  | 91 | 1.8E-2 | 4.6E-1 | |  | GOTERM\_BP\_5 | positive regulation of nucleobase-containing compound metabolic process | **RT** |  | 57 | 2.0E-2 | 4.9E-1 | |  | GOTERM\_BP\_5 | positive regulation of RNA metabolic process | **RT** |  | 53 | 2.2E-2 | 4.9E-1 | |  | GOTERM\_BP\_5 | positive regulation of transcription, DNA-templated | **RT** |  | 52 | 2.6E-2 | 5.2E-1 | |  | GOTERM\_BP\_5 | positive regulation of RNA biosynthetic process | **RT** |  | 52 | 2.6E-2 | 5.2E-1 | |  | GOTERM\_BP\_5 | positive regulation of cellular biosynthetic process | **RT** |  | 62 | 3.1E-2 | 5.6E-1 | |  | GOTERM\_BP\_5 | regulation of RNA metabolic process | **RT** |  | 140 | 3.8E-2 | 5.9E-1 | |  | GOTERM\_BP\_5 | regulation of gene expression | **RT** |  | 163 | 5.5E-2 | 6.5E-1 | |  | GOTERM\_BP\_5 | positive regulation of macromolecule biosynthetic process | **RT** |  | 54 | 7.7E-2 | 7.2E-1 | |  | GOTERM\_BP\_5 | regulation of transcription, DNA-templated | **RT** |  | 124 | 1.0E-1 | 7.7E-1 | |  | GOTERM\_BP\_5 | regulation of RNA biosynthetic process | **RT** |  | 124 | 1.1E-1 | 7.8E-1 | |  | GOTERM\_BP\_5 | positive regulation of gene expression | **RT** |  | 55 | 1.1E-1 | 7.8E-1 | |  | GOTERM\_BP\_5 | regulation of macromolecule biosynthetic process | **RT** |  | 143 | 1.3E-1 | 8.1E-1 | |  | GOTERM\_BP\_5 | regulation of cellular macromolecule biosynthetic process | **RT** |  | 142 | 1.3E-1 | 8.2E-1 | |  | GOTERM\_BP\_5 | RNA biosynthetic process | **RT** |  | 127 | 2.0E-1 | 8.8E-1 | |  | GOTERM\_BP\_5 | transcription, DNA-templated | **RT** |  | 113 | 2.1E-1 | 8.9E-1 | |  | GOTERM\_CC\_5 | nucleus | **RT** |  | 269 | 2.1E-1 | 9.5E-1 | |  | GOTERM\_BP\_5 | RNA metabolic process | **RT** |  | 182 | 2.9E-1 | 9.3E-1 | |  | GOTERM\_CC\_5 | nuclear part | **RT** |  | 131 | 3.1E-1 | 9.6E-1 | |  | GOTERM\_BP\_5 | nucleobase-containing compound biosynthetic process | **RT** |  | 142 | 3.3E-1 | 9.5E-1 | |  | GOTERM\_BP\_5 | cellular macromolecule biosynthetic process | **RT** |  | 204 | 3.4E-1 | 9.5E-1 | |  | GOTERM\_BP\_5 | nucleic acid metabolic process | **RT** |  | 194 | 3.7E-1 | 9.6E-1 | | Annotation Cluster 26 | | Enrichment Score: 1.03 |  |  | Count | P\_Value | Benjamini | | --- | --- | --- | --- | --- | --- | --- | --- | |  | GOTERM\_BP\_5 | regulation of neurogenesis | **RT** |  | 38 | 4.9E-2 | 6.4E-1 | |  | GOTERM\_BP\_5 | regulation of axonogenesis | **RT** |  | 12 | 4.9E-2 | 6.4E-1 | |  | GOTERM\_BP\_5 | regulation of nervous system development | **RT** |  | 55 | 5.6E-2 | 6.6E-1 | |  | GOTERM\_BP\_5 | regulation of neuron differentiation | **RT** |  | 28 | 1.5E-1 | 8.3E-1 | |  | GOTERM\_BP\_5 | regulation of neuron projection development | **RT** |  | 20 | 1.7E-1 | 8.6E-1 | |  | GOTERM\_BP\_5 | regulation of cell morphogenesis involved in differentiation | **RT** |  | 17 | 1.9E-1 | 8.7E-1 | | Annotation Cluster 27 | | Enrichment Score: 0.98 |  |  | Count | P\_Value | Benjamini | | --- | --- | --- | --- | --- | --- | --- | --- | |  | GOTERM\_BP\_5 | mitotic cytokinesis | **RT** |  | 12 | 8.4E-2 | 7.4E-1 | |  | GOTERM\_BP\_5 | cytokinesis | **RT** |  | 16 | 9.4E-2 | 7.6E-1 | |  | GOTERM\_BP\_5 | cytoskeleton-dependent cytokinesis | **RT** |  | 15 | 1.4E-1 | 8.2E-1 | | Annotation Cluster 28 | | Enrichment Score: 0.98 |  |  | Count | P\_Value | Benjamini | | --- | --- | --- | --- | --- | --- | --- | --- | |  | GOTERM\_BP\_5 | somatic stem cell division | **RT** |  | 11 | 6.0E-2 | 6.7E-1 | |  | GOTERM\_BP\_5 | neuroblast division | **RT** |  | 10 | 7.4E-2 | 7.2E-1 | |  | GOTERM\_BP\_5 | asymmetric neuroblast division | **RT** |  | 10 | 7.4E-2 | 7.2E-1 | |  | GOTERM\_BP\_5 | asymmetric stem cell division | **RT** |  | 12 | 1.3E-1 | 8.2E-1 | |  | GOTERM\_BP\_5 | apical protein localization | **RT** |  | 4 | 3.0E-1 | 9.4E-1 | | Annotation Cluster 29 | | Enrichment Score: 0.96 |  |  | Count | P\_Value | Benjamini | | --- | --- | --- | --- | --- | --- | --- | --- | |  | GOTERM\_BP\_5 | chitin-based cuticle development | **RT** |  | 11 | 6.0E-2 | 6.7E-1 | |  | GOTERM\_BP\_5 | cuticle development involved in chitin-based cuticle molting cycle | **RT** |  | 5 | 1.1E-1 | 7.8E-1 | |  | GOTERM\_BP\_5 | cuticle pattern formation | **RT** |  | 6 | 1.1E-1 | 7.9E-1 | |  | GOTERM\_BP\_5 | chitin-based larval cuticle pattern formation | **RT** |  | 4 | 1.9E-1 | 8.7E-1 | | Annotation Cluster 30 | | Enrichment Score: 0.95 |  |  | Count | P\_Value | Benjamini | | --- | --- | --- | --- | --- | --- | --- | --- | |  | GOTERM\_BP\_5 | regionalization | **RT** |  | 69 | 2.3E-3 | 1.4E-1 | |  | GOTERM\_BP\_5 | segmentation | **RT** |  | 37 | 1.3E-2 | 4.0E-1 | |  | GOTERM\_BP\_5 | pole plasm assembly | **RT** |  | 14 | 2.6E-2 | 5.2E-1 | |  | GOTERM\_BP\_5 | oocyte axis specification | **RT** |  | 21 | 8.1E-2 | 7.4E-1 | |  | GOTERM\_BP\_5 | blastoderm segmentation | **RT** |  | 28 | 8.3E-2 | 7.4E-1 | |  | GOTERM\_BP\_5 | intracellular mRNA localization involved in pattern specification process | **RT** |  | 12 | 8.4E-2 | 7.4E-1 | |  | GOTERM\_BP\_5 | embryonic pattern specification | **RT** |  | 31 | 9.2E-2 | 7.6E-1 | |  | GOTERM\_BP\_5 | anterior/posterior pattern specification | **RT** |  | 23 | 1.0E-1 | 7.7E-1 | |  | GOTERM\_BP\_5 | oocyte construction | **RT** |  | 21 | 1.1E-1 | 7.8E-1 | |  | GOTERM\_BP\_5 | cell maturation | **RT** |  | 30 | 1.3E-1 | 8.1E-1 | |  | GOTERM\_BP\_5 | oocyte anterior/posterior axis specification | **RT** |  | 14 | 1.7E-1 | 8.6E-1 | |  | GOTERM\_BP\_5 | pole plasm RNA localization | **RT** |  | 9 | 1.8E-1 | 8.7E-1 | |  | GOTERM\_BP\_5 | pole plasm mRNA localization | **RT** |  | 9 | 1.8E-1 | 8.7E-1 | |  | GOTERM\_BP\_5 | axis specification | **RT** |  | 27 | 1.8E-1 | 8.7E-1 | |  | GOTERM\_BP\_5 | oocyte development | **RT** |  | 22 | 2.0E-1 | 8.8E-1 | |  | GOTERM\_BP\_5 | intracellular mRNA localization involved in anterior/posterior axis specification | **RT** |  | 10 | 2.2E-1 | 9.0E-1 | |  | GOTERM\_BP\_5 | tripartite regional subdivision | **RT** |  | 18 | 2.5E-1 | 9.2E-1 | |  | GOTERM\_BP\_5 | anterior/posterior axis specification | **RT** |  | 18 | 2.5E-1 | 9.2E-1 | |  | GOTERM\_BP\_5 | oocyte differentiation | **RT** |  | 23 | 3.0E-1 | 9.4E-1 | |  | GOTERM\_BP\_5 | embryonic axis specification | **RT** |  | 18 | 3.3E-1 | 9.5E-1 | |  | GOTERM\_BP\_5 | pole plasm oskar mRNA localization | **RT** |  | 6 | 5.8E-1 | 9.9E-1 | | Annotation Cluster 31 | | Enrichment Score: 0.89 |  |  | Count | P\_Value | Benjamini | | --- | --- | --- | --- | --- | --- | --- | --- | |  | GOTERM\_BP\_5 | positive regulation of intracellular protein transport | **RT** |  | 8 | 2.2E-2 | 5.0E-1 | |  | GOTERM\_BP\_5 | positive regulation of cellular protein localization | **RT** |  | 9 | 4.6E-2 | 6.4E-1 | |  | GOTERM\_BP\_5 | positive regulation of intracellular transport | **RT** |  | 8 | 5.7E-2 | 6.6E-1 | |  | GOTERM\_BP\_5 | regulation of establishment of protein localization | **RT** |  | 16 | 6.2E-2 | 6.7E-1 | |  | GOTERM\_BP\_5 | regulation of protein transport | **RT** |  | 15 | 6.4E-2 | 6.8E-1 | |  | GOTERM\_BP\_5 | regulation of intracellular protein transport | **RT** |  | 10 | 7.4E-2 | 7.2E-1 | |  | GOTERM\_BP\_5 | positive regulation of protein transport | **RT** |  | 10 | 7.4E-2 | 7.2E-1 | |  | GOTERM\_BP\_5 | positive regulation of protein import into nucleus | **RT** |  | 5 | 7.6E-2 | 7.2E-1 | |  | GOTERM\_BP\_5 | positive regulation of protein import | **RT** |  | 5 | 7.6E-2 | 7.2E-1 | |  | GOTERM\_BP\_5 | positive regulation of establishment of protein localization | **RT** |  | 10 | 8.9E-2 | 7.5E-1 | |  | GOTERM\_BP\_5 | regulation of protein import | **RT** |  | 7 | 9.1E-2 | 7.5E-1 | |  | GOTERM\_BP\_5 | regulation of protein localization to nucleus | **RT** |  | 7 | 1.4E-1 | 8.2E-1 | |  | GOTERM\_BP\_5 | nucleocytoplasmic transport | **RT** |  | 19 | 1.5E-1 | 8.3E-1 | |  | GOTERM\_BP\_5 | nuclear transport | **RT** |  | 19 | 1.5E-1 | 8.3E-1 | |  | GOTERM\_BP\_5 | regulation of cellular protein localization | **RT** |  | 12 | 1.7E-1 | 8.6E-1 | |  | GOTERM\_BP\_5 | positive regulation of nucleocytoplasmic transport | **RT** |  | 5 | 1.9E-1 | 8.7E-1 | |  | GOTERM\_BP\_5 | positive regulation of transcription factor import into nucleus | **RT** |  | 4 | 1.9E-1 | 8.7E-1 | |  | GOTERM\_BP\_5 | positive regulation of transport | **RT** |  | 19 | 2.2E-1 | 8.9E-1 | |  | GOTERM\_BP\_5 | regulation of intracellular transport | **RT** |  | 10 | 2.5E-1 | 9.2E-1 | |  | GOTERM\_BP\_5 | regulation of nucleocytoplasmic transport | **RT** |  | 7 | 3.1E-1 | 9.4E-1 | |  | GOTERM\_BP\_5 | protein import into nucleus | **RT** |  | 9 | 3.9E-1 | 9.7E-1 | |  | GOTERM\_BP\_5 | protein targeting to nucleus | **RT** |  | 9 | 3.9E-1 | 9.7E-1 | |  | GOTERM\_BP\_5 | single-organism nuclear import | **RT** |  | 9 | 3.9E-1 | 9.7E-1 | |  | GOTERM\_BP\_5 | protein import | **RT** |  | 14 | 4.2E-1 | 9.7E-1 | | Annotation Cluster 32 | | Enrichment Score: 0.89 |  |  | Count | P\_Value | Benjamini | | --- | --- | --- | --- | --- | --- | --- | --- | |  | GOTERM\_BP\_5 | positive regulation of canonical Wnt signaling pathway | **RT** |  | 6 | 1.7E-2 | 4.5E-1 | |  | GOTERM\_BP\_5 | positive regulation of Wnt signaling pathway | **RT** |  | 9 | 7.4E-2 | 7.2E-1 | |  | GOTERM\_BP\_5 | regulation of canonical Wnt signaling pathway | **RT** |  | 8 | 9.2E-2 | 7.5E-1 | |  | GOTERM\_BP\_5 | regulation of Wnt signaling pathway | **RT** |  | 17 | 1.3E-1 | 8.1E-1 | |  | GOTERM\_CC\_5 | protein phosphatase type 1 complex | **RT** |  | 3 | 4.2E-1 | 9.6E-1 | |  | GOTERM\_BP\_5 | protein complex disassembly | **RT** |  | 6 | 7.9E-1 | 1.0E0 | | Annotation Cluster 33 | | Enrichment Score: 0.87 |  |  | Count | P\_Value | Benjamini | | --- | --- | --- | --- | --- | --- | --- | --- | |  | GOTERM\_BP\_5 | cellular response to starvation | **RT** |  | 15 | 1.3E-1 | 8.1E-1 | |  | GOTERM\_BP\_5 | response to starvation | **RT** |  | 22 | 1.4E-1 | 8.2E-1 | |  | GOTERM\_BP\_5 | cellular response to nutrient levels | **RT** |  | 15 | 1.4E-1 | 8.2E-1 | | Annotation Cluster 34 | | Enrichment Score: 0.86 |  |  | Count | P\_Value | Benjamini | | --- | --- | --- | --- | --- | --- | --- | --- | |  | GOTERM\_BP\_5 | nucleoside triphosphate metabolic process | **RT** |  | 30 | 1.3E-2 | 4.0E-1 | |  | GOTERM\_BP\_5 | purine nucleoside metabolic process | **RT** |  | 32 | 1.4E-2 | 4.1E-1 | |  | GOTERM\_BP\_5 | nucleoside monophosphate metabolic process | **RT** |  | 30 | 1.7E-2 | 4.6E-1 | |  | GOTERM\_BP\_5 | nucleoside phosphate metabolic process | **RT** |  | 44 | 2.2E-2 | 5.0E-1 | |  | GOTERM\_BP\_5 | nucleotide metabolic process | **RT** |  | 43 | 3.1E-2 | 5.7E-1 | |  | GOTERM\_BP\_5 | nucleoside metabolic process | **RT** |  | 33 | 3.5E-2 | 5.8E-1 | |  | GOTERM\_BP\_5 | ribonucleoside metabolic process | **RT** |  | 32 | 3.7E-2 | 5.9E-1 | |  | GOTERM\_BP\_5 | cellular respiration | **RT** |  | 22 | 5.4E-2 | 6.6E-1 | |  | GOTERM\_BP\_5 | purine nucleotide metabolic process | **RT** |  | 36 | 5.4E-2 | 6.5E-1 | |  | GOTERM\_BP\_5 | nucleobase-containing small molecule metabolic process | **RT** |  | 45 | 5.6E-2 | 6.6E-1 | |  | GOTERM\_BP\_5 | ribose phosphate metabolic process | **RT** |  | 36 | 7.6E-2 | 7.2E-1 | |  | GOTERM\_BP\_5 | ribonucleotide metabolic process | **RT** |  | 35 | 1.0E-1 | 7.7E-1 | |  | GOTERM\_BP\_5 | respiratory electron transport chain | **RT** |  | 15 | 1.6E-1 | 8.4E-1 | |  | GOTERM\_BP\_5 | ATP synthesis coupled electron transport | **RT** |  | 14 | 1.7E-1 | 8.6E-1 | |  | GOTERM\_CC\_5 | mitochondrial respiratory chain | **RT** |  | 14 | 2.7E-1 | 9.5E-1 | |  | GOTERM\_CC\_5 | mitochondrial membrane | **RT** |  | 36 | 2.8E-1 | 9.6E-1 | |  | GOTERM\_MF\_5 | NADH dehydrogenase (quinone) activity | **RT** |  | 7 | 3.1E-1 | 9.8E-1 | |  | GOTERM\_CC\_5 | mitochondrial part | **RT** |  | 56 | 3.2E-1 | 9.6E-1 | |  | GOTERM\_CC\_5 | organelle envelope | **RT** |  | 50 | 3.3E-1 | 9.5E-1 | |  | GOTERM\_CC\_5 | organelle inner membrane | **RT** |  | 30 | 3.3E-1 | 9.5E-1 | |  | GOTERM\_CC\_5 | mitochondrial membrane part | **RT** |  | 23 | 3.4E-1 | 9.5E-1 | |  | GOTERM\_CC\_5 | mitochondrial envelope | **RT** |  | 36 | 3.7E-1 | 9.6E-1 | |  | GOTERM\_CC\_5 | mitochondrion | **RT** |  | 80 | 3.8E-1 | 9.6E-1 | |  | GOTERM\_BP\_5 | mitochondrial electron transport, NADH to ubiquinone | **RT** |  | 7 | 3.9E-1 | 9.6E-1 | |  | GOTERM\_CC\_5 | mitochondrial protein complex | **RT** |  | 22 | 4.0E-1 | 9.6E-1 | |  | GOTERM\_CC\_5 | mitochondrial inner membrane | **RT** |  | 27 | 4.4E-1 | 9.6E-1 | |  | GOTERM\_CC\_5 | inner mitochondrial membrane protein complex | **RT** |  | 19 | 4.5E-1 | 9.6E-1 | |  | GOTERM\_CC\_5 | respiratory chain complex I | **RT** |  | 8 | 5.3E-1 | 9.8E-1 | |  | GOTERM\_CC\_5 | mitochondrial respiratory chain complex I | **RT** |  | 8 | 5.3E-1 | 9.8E-1 | |  | GOTERM\_CC\_5 | NADH dehydrogenase complex | **RT** |  | 8 | 5.3E-1 | 9.8E-1 | | Annotation Cluster 35 | | Enrichment Score: 0.86 |  |  | Count | P\_Value | Benjamini | | --- | --- | --- | --- | --- | --- | --- | --- | |  | GOTERM\_BP\_5 | dorsal appendage formation | **RT** |  | 10 | 7.4E-2 | 7.2E-1 | |  | GOTERM\_BP\_5 | eggshell chorion assembly | **RT** |  | 12 | 9.9E-2 | 7.7E-1 | |  | GOTERM\_BP\_5 | chorion-containing eggshell formation | **RT** |  | 13 | 1.6E-1 | 8.4E-1 | |  | GOTERM\_BP\_5 | eggshell formation | **RT** |  | 13 | 1.8E-1 | 8.7E-1 | |  | GOTERM\_BP\_5 | cellular component assembly involved in morphogenesis | **RT** |  | 18 | 2.5E-1 | 9.2E-1 | | Annotation Cluster 36 | | Enrichment Score: 0.84 |  |  | Count | P\_Value | Benjamini | | --- | --- | --- | --- | --- | --- | --- | --- | |  | GOTERM\_CC\_5 | polymeric cytoskeletal fiber | **RT** |  | 25 | 4.6E-2 | 7.9E-1 | |  | GOTERM\_CC\_5 | microtubule | **RT** |  | 18 | 7.4E-2 | 8.3E-1 | |  | GOTERM\_CC\_5 | spindle | **RT** |  | 17 | 3.6E-1 | 9.6E-1 | |  | GOTERM\_CC\_5 | spindle microtubule | **RT** |  | 6 | 3.7E-1 | 9.6E-1 | | Annotation Cluster 37 | | Enrichment Score: 0.8 |  |  | Count | P\_Value | Benjamini | | --- | --- | --- | --- | --- | --- | --- | --- | |  | GOTERM\_BP\_5 | heat shock-mediated polytene chromosome puffing | **RT** |  | 4 | 9.0E-2 | 7.5E-1 | |  | GOTERM\_BP\_5 | polytene chromosome puffing | **RT** |  | 4 | 1.4E-1 | 8.2E-1 | |  | GOTERM\_BP\_5 | cellular response to heat | **RT** |  | 6 | 1.5E-1 | 8.3E-1 | |  | GOTERM\_BP\_5 | response to unfolded protein | **RT** |  | 6 | 3.4E-1 | 9.5E-1 | | Annotation Cluster 38 | | Enrichment Score: 0.76 |  |  | Count | P\_Value | Benjamini | | --- | --- | --- | --- | --- | --- | --- | --- | |  | GOTERM\_BP\_5 | regulation of programmed cell death | **RT** |  | 33 | 5.8E-2 | 6.6E-1 | |  | GOTERM\_BP\_5 | regulation of apoptotic process | **RT** |  | 28 | 1.3E-1 | 8.1E-1 | |  | GOTERM\_BP\_5 | negative regulation of programmed cell death | **RT** |  | 17 | 1.4E-1 | 8.2E-1 | |  | GOTERM\_BP\_5 | negative regulation of cell death | **RT** |  | 18 | 2.7E-1 | 9.2E-1 | |  | GOTERM\_BP\_5 | apoptotic process | **RT** |  | 30 | 3.0E-1 | 9.4E-1 | |  | GOTERM\_BP\_5 | negative regulation of apoptotic process | **RT** |  | 14 | 3.2E-1 | 9.5E-1 | | Annotation Cluster 39 | | Enrichment Score: 0.76 |  |  | Count | P\_Value | Benjamini | | --- | --- | --- | --- | --- | --- | --- | --- | |  | GOTERM\_BP\_5 | pteridine biosynthetic process | **RT** |  | 3 | 9.7E-2 | 7.7E-1 | |  | GOTERM\_BP\_5 | pteridine metabolic process | **RT** |  | 3 | 1.7E-1 | 8.6E-1 | |  | GOTERM\_BP\_5 | pteridine-containing compound biosynthetic process | **RT** |  | 3 | 3.3E-1 | 9.5E-1 | | Annotation Cluster 40 | | Enrichment Score: 0.73 |  |  | Count | P\_Value | Benjamini | | --- | --- | --- | --- | --- | --- | --- | --- | |  | GOTERM\_BP\_5 | dorsal/ventral pattern formation | **RT** |  | 22 | 1.1E-1 | 7.8E-1 | |  | GOTERM\_BP\_5 | imaginal disc pattern formation | **RT** |  | 15 | 1.6E-1 | 8.4E-1 | |  | GOTERM\_BP\_5 | dorsal/ventral pattern formation, imaginal disc | **RT** |  | 10 | 1.7E-1 | 8.6E-1 | |  | GOTERM\_BP\_5 | wing disc pattern formation | **RT** |  | 10 | 4.2E-1 | 9.7E-1 | | Annotation Cluster 41 | | Enrichment Score: 0.73 |  |  | Count | P\_Value | Benjamini | | --- | --- | --- | --- | --- | --- | --- | --- | |  | GOTERM\_BP\_5 | regulation of nurse cell apoptotic process | **RT** |  | 4 | 9.0E-2 | 7.5E-1 | |  | GOTERM\_BP\_5 | positive regulation of nurse cell apoptotic process | **RT** |  | 3 | 1.7E-1 | 8.6E-1 | |  | GOTERM\_BP\_5 | positive regulation of apoptotic process involved in development | **RT** |  | 3 | 1.7E-1 | 8.6E-1 | |  | GOTERM\_BP\_5 | nurse cell apoptotic process | **RT** |  | 4 | 4.7E-1 | 9.8E-1 | | Annotation Cluster 42 | | Enrichment Score: 0.73 |  |  | Count | P\_Value | Benjamini | | --- | --- | --- | --- | --- | --- | --- | --- | |  | GOTERM\_BP\_5 | monosaccharide metabolic process | **RT** |  | 9 | 7.4E-2 | 7.2E-1 | |  | GOTERM\_BP\_5 | hexose metabolic process | **RT** |  | 8 | 9.2E-2 | 7.5E-1 | |  | GOTERM\_BP\_5 | carbohydrate biosynthetic process | **RT** |  | 3 | 9.7E-1 | 1.0E0 | | Annotation Cluster 43 | | Enrichment Score: 0.72 |  |  | Count | P\_Value | Benjamini | | --- | --- | --- | --- | --- | --- | --- | --- | |  | GOTERM\_MF\_5 | purine ribonucleoside binding | **RT** |  | 109 | 7.4E-2 | 9.6E-1 | |  | GOTERM\_MF\_5 | purine ribonucleotide binding | **RT** |  | 109 | 9.3E-2 | 9.5E-1 | |  | GOTERM\_MF\_5 | ribonucleotide binding | **RT** |  | 110 | 9.5E-2 | 9.4E-1 | |  | GOTERM\_MF\_5 | purine nucleotide binding | **RT** |  | 109 | 9.8E-2 | 9.3E-1 | |  | GOTERM\_MF\_5 | protein kinase activity | **RT** |  | 35 | 3.8E-1 | 9.9E-1 | |  | GOTERM\_MF\_5 | ATP binding | **RT** |  | 79 | 3.9E-1 | 9.9E-1 | |  | GOTERM\_MF\_5 | adenyl ribonucleotide binding | **RT** |  | 79 | 4.2E-1 | 9.9E-1 | |  | GOTERM\_MF\_5 | adenyl nucleotide binding | **RT** |  | 79 | 4.2E-1 | 9.9E-1 | | Annotation Cluster 44 | | Enrichment Score: 0.72 |  |  | Count | P\_Value | Benjamini | | --- | --- | --- | --- | --- | --- | --- | --- | |  | GOTERM\_BP\_5 | appendage segmentation | **RT** |  | 5 | 1.5E-1 | 8.3E-1 | |  | GOTERM\_BP\_5 | imaginal disc-derived leg segmentation | **RT** |  | 5 | 1.5E-1 | 8.3E-1 | |  | GOTERM\_BP\_5 | imaginal disc-derived leg joint morphogenesis | **RT** |  | 3 | 3.3E-1 | 9.5E-1 | | Annotation Cluster 45 | | Enrichment Score: 0.71 |  |  | Count | P\_Value | Benjamini | | --- | --- | --- | --- | --- | --- | --- | --- | |  | GOTERM\_BP\_5 | intracellular receptor signaling pathway | **RT** |  | 7 | 5.2E-2 | 6.5E-1 | |  | GOTERM\_BP\_5 | response to ecdysone | **RT** |  | 10 | 1.1E-1 | 7.8E-1 | |  | GOTERM\_BP\_5 | response to sterol | **RT** |  | 10 | 1.1E-1 | 7.8E-1 | |  | GOTERM\_BP\_5 | cellular response to lipid | **RT** |  | 9 | 1.1E-1 | 7.8E-1 | |  | GOTERM\_BP\_5 | cellular response to alcohol | **RT** |  | 7 | 1.2E-1 | 7.8E-1 | |  | GOTERM\_BP\_5 | cellular response to ketone | **RT** |  | 6 | 1.5E-1 | 8.3E-1 | |  | GOTERM\_BP\_5 | cellular response to sterol | **RT** |  | 6 | 1.5E-1 | 8.3E-1 | |  | GOTERM\_BP\_5 | hormone-mediated signaling pathway | **RT** |  | 9 | 2.1E-1 | 8.9E-1 | |  | GOTERM\_BP\_5 | cellular response to hormone stimulus | **RT** |  | 15 | 3.2E-1 | 9.5E-1 | |  | GOTERM\_BP\_5 | cellular response to steroid hormone stimulus | **RT** |  | 6 | 3.4E-1 | 9.5E-1 | |  | GOTERM\_BP\_5 | steroid hormone mediated signaling pathway | **RT** |  | 6 | 3.4E-1 | 9.5E-1 | |  | GOTERM\_BP\_5 | response to steroid hormone | **RT** |  | 6 | 3.4E-1 | 9.5E-1 | |  | GOTERM\_BP\_5 | intracellular steroid hormone receptor signaling pathway | **RT** |  | 4 | 3.6E-1 | 9.6E-1 | |  | GOTERM\_BP\_5 | cellular response to organic cyclic compound | **RT** |  | 7 | 7.7E-1 | 1.0E0 | | Annotation Cluster 46 | | Enrichment Score: 0.71 |  |  | Count | P\_Value | Benjamini | | --- | --- | --- | --- | --- | --- | --- | --- | |  | GOTERM\_BP\_5 | mushroom body development | **RT** |  | 17 | 8.9E-2 | 7.6E-1 | |  | GOTERM\_BP\_5 | brain development | **RT** |  | 21 | 2.6E-1 | 9.2E-1 | |  | GOTERM\_BP\_5 | central nervous system development | **RT** |  | 36 | 3.3E-1 | 9.5E-1 | | Annotation Cluster 47 | | Enrichment Score: 0.71 |  |  | Count | P\_Value | Benjamini | | --- | --- | --- | --- | --- | --- | --- | --- | |  | GOTERM\_BP\_5 | chitin-based cuticle sclerotization | **RT** |  | 8 | 5.7E-2 | 6.6E-1 | |  | GOTERM\_BP\_5 | cuticle pigmentation | **RT** |  | 9 | 5.9E-2 | 6.7E-1 | |  | GOTERM\_BP\_5 | adult chitin-containing cuticle pigmentation | **RT** |  | 5 | 1.9E-1 | 8.7E-1 | |  | GOTERM\_BP\_5 | regulation of cuticle pigmentation | **RT** |  | 4 | 3.6E-1 | 9.6E-1 | |  | GOTERM\_BP\_5 | regulation of adult chitin-containing cuticle pigmentation | **RT** |  | 3 | 4.8E-1 | 9.8E-1 | |  | GOTERM\_BP\_5 | regulation of chitin-based cuticle tanning | **RT** |  | 3 | 5.4E-1 | 9.9E-1 | | Annotation Cluster 48 | | Enrichment Score: 0.7 |  |  | Count | P\_Value | Benjamini | | --- | --- | --- | --- | --- | --- | --- | --- | |  | GOTERM\_BP\_5 | STAT cascade | **RT** |  | 11 | 1.6E-1 | 8.5E-1 | |  | GOTERM\_BP\_5 | regulation of STAT cascade | **RT** |  | 10 | 2.2E-1 | 9.0E-1 | |  | GOTERM\_BP\_5 | positive regulation of STAT cascade | **RT** |  | 5 | 2.3E-1 | 9.0E-1 | | Annotation Cluster 49 | | Enrichment Score: 0.69 |  |  | Count | P\_Value | Benjamini | | --- | --- | --- | --- | --- | --- | --- | --- | |  | GOTERM\_BP\_5 | germ-line sex determination | **RT** |  | 3 | 9.7E-2 | 7.7E-1 | |  | GOTERM\_BP\_5 | female germ-line sex determination | **RT** |  | 3 | 9.7E-2 | 7.7E-1 | |  | GOTERM\_BP\_5 | primary sex determination, germ-line | **RT** |  | 3 | 9.7E-2 | 7.7E-1 | |  | GOTERM\_BP\_5 | primary sex determination | **RT** |  | 4 | 1.9E-1 | 8.7E-1 | |  | GOTERM\_BP\_5 | primary sex determination, soma | **RT** |  | 3 | 3.3E-1 | 9.5E-1 | |  | GOTERM\_BP\_5 | female sex determination | **RT** |  | 3 | 4.0E-1 | 9.7E-1 | |  | GOTERM\_BP\_5 | somatic sex determination | **RT** |  | 3 | 6.6E-1 | 1.0E0 | | Annotation Cluster 50 | | Enrichment Score: 0.67 |  |  | Count | P\_Value | Benjamini | | --- | --- | --- | --- | --- | --- | --- | --- | |  | GOTERM\_BP\_5 | cellular chemical homeostasis | **RT** |  | 17 | 1.9E-1 | 8.7E-1 | |  | GOTERM\_BP\_5 | cellular ion homeostasis | **RT** |  | 16 | 2.0E-1 | 8.8E-1 | |  | GOTERM\_BP\_5 | ion homeostasis | **RT** |  | 18 | 2.5E-1 | 9.2E-1 | | Annotation Cluster 51 | | Enrichment Score: 0.63 |  |  | Count | P\_Value | Benjamini | | --- | --- | --- | --- | --- | --- | --- | --- | |  | GOTERM\_BP\_5 | embryonic organ development | **RT** |  | 10 | 2.2E-2 | 5.1E-1 | |  | GOTERM\_BP\_5 | hemocyte differentiation | **RT** |  | 7 | 9.1E-2 | 7.5E-1 | |  | GOTERM\_BP\_5 | regulation of hemopoiesis | **RT** |  | 4 | 1.4E-1 | 8.2E-1 | |  | GOTERM\_BP\_5 | hemocyte migration | **RT** |  | 5 | 1.9E-1 | 8.7E-1 | |  | GOTERM\_BP\_5 | embryonic hemopoiesis | **RT** |  | 5 | 1.9E-1 | 8.7E-1 | |  | GOTERM\_BP\_5 | hemopoiesis | **RT** |  | 14 | 1.9E-1 | 8.7E-1 | |  | GOTERM\_BP\_5 | immune system development | **RT** |  | 15 | 2.1E-1 | 8.9E-1 | |  | GOTERM\_BP\_5 | hematopoietic or lymphoid organ development | **RT** |  | 15 | 2.1E-1 | 8.9E-1 | |  | GOTERM\_BP\_5 | negative regulation of post-embryonic development | **RT** |  | 3 | 2.5E-1 | 9.2E-1 | |  | GOTERM\_BP\_5 | regulation of lamellocyte differentiation | **RT** |  | 3 | 2.5E-1 | 9.2E-1 | |  | GOTERM\_BP\_5 | negative regulation of lamellocyte differentiation | **RT** |  | 3 | 2.5E-1 | 9.2E-1 | |  | GOTERM\_BP\_5 | regulation of plasmatocyte differentiation | **RT** |  | 3 | 2.5E-1 | 9.2E-1 | |  | GOTERM\_BP\_5 | negative regulation of plasmatocyte differentiation | **RT** |  | 3 | 2.5E-1 | 9.2E-1 | |  | GOTERM\_BP\_5 | regulation of post-embryonic development | **RT** |  | 4 | 3.0E-1 | 9.4E-1 | |  | GOTERM\_BP\_5 | negative regulation of hemopoiesis | **RT** |  | 3 | 3.3E-1 | 9.5E-1 | |  | GOTERM\_BP\_5 | negative regulation of hemocyte differentiation | **RT** |  | 3 | 3.3E-1 | 9.5E-1 | |  | GOTERM\_BP\_5 | larval lymph gland hemopoiesis | **RT** |  | 6 | 5.4E-1 | 9.9E-1 | |  | GOTERM\_BP\_5 | post-embryonic hemopoiesis | **RT** |  | 6 | 5.4E-1 | 9.9E-1 | |  | GOTERM\_BP\_5 | lymph gland development | **RT** |  | 7 | 5.7E-1 | 9.9E-1 | |  | GOTERM\_BP\_5 | regulation of Toll signaling pathway | **RT** |  | 3 | 7.0E-1 | 1.0E0 | | Annotation Cluster 52 | | Enrichment Score: 0.62 |  |  | Count | P\_Value | Benjamini | | --- | --- | --- | --- | --- | --- | --- | --- | |  | GOTERM\_BP\_5 | mitochondrial electron transport, ubiquinol to cytochrome c | **RT** |  | 5 | 1.1E-1 | 7.8E-1 | |  | GOTERM\_CC\_5 | respiratory chain complex III | **RT** |  | 4 | 2.0E-1 | 9.6E-1 | |  | GOTERM\_CC\_5 | mitochondrial respiratory chain complex III | **RT** |  | 4 | 2.0E-1 | 9.6E-1 | |  | GOTERM\_MF\_5 | ubiquinol-cytochrome-c reductase activity | **RT** |  | 4 | 2.0E-1 | 9.8E-1 | |  | GOTERM\_CC\_5 | transmembrane transporter complex | **RT** |  | 10 | 9.3E-1 | 1.0E0 | | Annotation Cluster 53 | | Enrichment Score: 0.61 |  |  | Count | P\_Value | Benjamini | | --- | --- | --- | --- | --- | --- | --- | --- | |  | GOTERM\_BP\_5 | negative regulation of RNA splicing | **RT** |  | 4 | 2.4E-1 | 9.1E-1 | |  | GOTERM\_BP\_5 | negative regulation of mRNA processing | **RT** |  | 4 | 2.4E-1 | 9.1E-1 | |  | GOTERM\_BP\_5 | negative regulation of mRNA metabolic process | **RT** |  | 4 | 2.4E-1 | 9.1E-1 | | Annotation Cluster 54 | | Enrichment Score: 0.61 |  |  | Count | P\_Value | Benjamini | | --- | --- | --- | --- | --- | --- | --- | --- | |  | GOTERM\_BP\_5 | programmed cell death involved in cell development | **RT** |  | 26 | 4.4E-2 | 6.3E-1 | |  | GOTERM\_BP\_5 | salivary gland morphogenesis | **RT** |  | 23 | 1.8E-1 | 8.7E-1 | |  | GOTERM\_BP\_5 | gland morphogenesis | **RT** |  | 23 | 1.8E-1 | 8.7E-1 | |  | GOTERM\_BP\_5 | exocrine system development | **RT** |  | 26 | 2.7E-1 | 9.2E-1 | |  | GOTERM\_BP\_5 | salivary gland development | **RT** |  | 26 | 2.7E-1 | 9.2E-1 | |  | GOTERM\_BP\_5 | gland development | **RT** |  | 31 | 3.0E-1 | 9.4E-1 | |  | GOTERM\_BP\_5 | salivary gland cell autophagic cell death | **RT** |  | 13 | 4.4E-1 | 9.8E-1 | |  | GOTERM\_BP\_5 | autophagic cell death | **RT** |  | 13 | 4.4E-1 | 9.8E-1 | |  | GOTERM\_BP\_5 | salivary gland histolysis | **RT** |  | 13 | 4.7E-1 | 9.8E-1 | | Annotation Cluster 55 | | Enrichment Score: 0.61 |  |  | Count | P\_Value | Benjamini | | --- | --- | --- | --- | --- | --- | --- | --- | |  | GOTERM\_BP\_5 | purine-containing compound catabolic process | **RT** |  | 5 | 7.6E-2 | 7.2E-1 | |  | GOTERM\_BP\_5 | nucleoside catabolic process | **RT** |  | 3 | 4.0E-1 | 9.7E-1 | |  | GOTERM\_BP\_5 | glycosyl compound catabolic process | **RT** |  | 3 | 4.8E-1 | 9.8E-1 | | Annotation Cluster 56 | | Enrichment Score: 0.6 |  |  | Count | P\_Value | Benjamini | | --- | --- | --- | --- | --- | --- | --- | --- | |  | GOTERM\_BP\_5 | regulation of lipid biosynthetic process | **RT** |  | 5 | 1.9E-1 | 8.7E-1 | |  | GOTERM\_BP\_5 | acylglycerol metabolic process | **RT** |  | 5 | 2.3E-1 | 9.0E-1 | |  | GOTERM\_BP\_5 | neutral lipid metabolic process | **RT** |  | 5 | 2.3E-1 | 9.0E-1 | |  | GOTERM\_BP\_5 | glycerolipid metabolic process | **RT** |  | 13 | 3.9E-1 | 9.7E-1 | | Annotation Cluster 57 | | Enrichment Score: 0.6 |  |  | Count | P\_Value | Benjamini | | --- | --- | --- | --- | --- | --- | --- | --- | |  | GOTERM\_BP\_5 | retinal cell programmed cell death | **RT** |  | 7 | 9.1E-2 | 7.5E-1 | |  | GOTERM\_BP\_5 | regulation of compound eye retinal cell programmed cell death | **RT** |  | 4 | 3.0E-1 | 9.4E-1 | |  | GOTERM\_BP\_5 | negative regulation of compound eye retinal cell programmed cell death | **RT** |  | 3 | 3.3E-1 | 9.5E-1 | |  | GOTERM\_BP\_5 | negative regulation of retinal cell programmed cell death | **RT** |  | 3 | 3.3E-1 | 9.5E-1 | |  | GOTERM\_BP\_5 | regulation of retinal cell programmed cell death | **RT** |  | 4 | 3.6E-1 | 9.6E-1 | | Annotation Cluster 58 | | Enrichment Score: 0.59 |  |  | Count | P\_Value | Benjamini | | --- | --- | --- | --- | --- | --- | --- | --- | |  | GOTERM\_BP\_5 | developmental cell growth | **RT** |  | 21 | 5.4E-3 | 2.3E-1 | |  | GOTERM\_BP\_5 | axon extension | **RT** |  | 16 | 1.8E-2 | 4.6E-1 | |  | GOTERM\_BP\_5 | neuron projection extension | **RT** |  | 16 | 2.2E-2 | 5.0E-1 | |  | GOTERM\_BP\_5 | regulation of axonogenesis | **RT** |  | 12 | 4.9E-2 | 6.4E-1 | |  | GOTERM\_BP\_5 | positive regulation of cell development | **RT** |  | 17 | 1.0E-1 | 7.7E-1 | |  | GOTERM\_MF\_5 | frizzled binding | **RT** |  | 3 | 1.8E-1 | 9.8E-1 | |  | GOTERM\_BP\_5 | regulation of axon extension | **RT** |  | 6 | 3.0E-1 | 9.4E-1 | |  | GOTERM\_BP\_5 | regulation of extent of cell growth | **RT** |  | 6 | 3.0E-1 | 9.4E-1 | |  | GOTERM\_BP\_5 | positive regulation of neurogenesis | **RT** |  | 12 | 3.7E-1 | 9.6E-1 | |  | GOTERM\_BP\_5 | positive regulation of axon guidance | **RT** |  | 3 | 4.0E-1 | 9.7E-1 | |  | GOTERM\_BP\_5 | regulation of axon guidance | **RT** |  | 5 | 4.2E-1 | 9.7E-1 | |  | GOTERM\_BP\_5 | positive regulation of axonogenesis | **RT** |  | 3 | 4.8E-1 | 9.8E-1 | |  | GOTERM\_BP\_5 | positive regulation of chemotaxis | **RT** |  | 3 | 4.8E-1 | 9.8E-1 | |  | GOTERM\_BP\_5 | positive regulation of axon extension | **RT** |  | 3 | 4.8E-1 | 9.8E-1 | |  | GOTERM\_BP\_5 | positive regulation of cell growth | **RT** |  | 7 | 5.0E-1 | 9.8E-1 | |  | GOTERM\_BP\_5 | positive regulation of cell projection organization | **RT** |  | 9 | 5.5E-1 | 9.9E-1 | |  | GOTERM\_BP\_5 | positive regulation of cellular component movement | **RT** |  | 4 | 5.7E-1 | 9.9E-1 | |  | GOTERM\_BP\_5 | positive regulation of neuron differentiation | **RT** |  | 9 | 5.8E-1 | 9.9E-1 | |  | GOTERM\_BP\_5 | regulation of cell size | **RT** |  | 14 | 5.9E-1 | 9.9E-1 | |  | GOTERM\_BP\_5 | positive regulation of cell morphogenesis involved in differentiation | **RT** |  | 5 | 6.7E-1 | 1.0E0 | |  | GOTERM\_BP\_5 | positive regulation of nervous system development | **RT** |  | 14 | 7.5E-1 | 1.0E0 | |  | GOTERM\_BP\_5 | positive regulation of neuron projection development | **RT** |  | 5 | 7.9E-1 | 1.0E0 | |  | GOTERM\_BP\_5 | neuron recognition | **RT** |  | 10 | 9.8E-1 | 1.0E0 | | Annotation Cluster 59 | | Enrichment Score: 0.59 |  |  | Count | P\_Value | Benjamini | | --- | --- | --- | --- | --- | --- | --- | --- | |  | GOTERM\_BP\_5 | cell development | **RT** |  | 220 | 1.8E-4 | 2.3E-2 | |  | GOTERM\_BP\_5 | cell morphogenesis | **RT** |  | 111 | 1.6E-1 | 8.4E-1 | |  | GOTERM\_BP\_5 | neuron differentiation | **RT** |  | 121 | 1.7E-1 | 8.6E-1 | |  | GOTERM\_BP\_5 | nervous system development | **RT** |  | 202 | 2.5E-1 | 9.2E-1 | |  | GOTERM\_BP\_5 | cell morphogenesis involved in differentiation | **RT** |  | 77 | 3.3E-1 | 9.5E-1 | |  | GOTERM\_BP\_5 | neurogenesis | **RT** |  | 170 | 3.6E-1 | 9.6E-1 | |  | GOTERM\_BP\_5 | neuron development | **RT** |  | 103 | 3.9E-1 | 9.7E-1 | |  | GOTERM\_BP\_5 | neuron projection development | **RT** |  | 85 | 4.5E-1 | 9.8E-1 | |  | GOTERM\_BP\_5 | axon development | **RT** |  | 50 | 4.6E-1 | 9.8E-1 | |  | GOTERM\_BP\_5 | neuron projection morphogenesis | **RT** |  | 80 | 4.7E-1 | 9.8E-1 | |  | GOTERM\_BP\_5 | cell part morphogenesis | **RT** |  | 84 | 4.7E-1 | 9.8E-1 | |  | GOTERM\_BP\_5 | cell projection morphogenesis | **RT** |  | 81 | 5.6E-1 | 9.9E-1 | |  | GOTERM\_BP\_5 | cell morphogenesis involved in neuron differentiation | **RT** |  | 67 | 5.9E-1 | 9.9E-1 | |  | GOTERM\_BP\_5 | dendrite development | **RT** |  | 31 | 7.0E-1 | 1.0E0 | |  | GOTERM\_BP\_5 | neuron projection guidance | **RT** |  | 36 | 8.0E-1 | 1.0E0 | |  | GOTERM\_BP\_5 | axon guidance | **RT** |  | 34 | 8.5E-1 | 1.0E0 | | Annotation Cluster 60 | | Enrichment Score: 0.59 |  |  | Count | P\_Value | Benjamini | | --- | --- | --- | --- | --- | --- | --- | --- | |  | GOTERM\_CC\_5 | U12-type spliceosomal complex | **RT** |  | 4 | 5.8E-2 | 8.1E-1 | |  | GOTERM\_CC\_5 | spliceosomal snRNP complex | **RT** |  | 9 | 3.0E-1 | 9.6E-1 | |  | GOTERM\_CC\_5 | small nuclear ribonucleoprotein complex | **RT** |  | 9 | 4.3E-1 | 9.6E-1 | |  | GOTERM\_CC\_5 | U2-type spliceosomal complex | **RT** |  | 4 | 6.0E-1 | 9.8E-1 | | Annotation Cluster 61 | | Enrichment Score: 0.56 |  |  | Count | P\_Value | Benjamini | | --- | --- | --- | --- | --- | --- | --- | --- | |  | GOTERM\_BP\_5 | regulation of embryonic cell shape | **RT** |  | 6 | 1.8E-1 | 8.7E-1 | |  | GOTERM\_BP\_5 | unidimensional cell growth | **RT** |  | 5 | 1.9E-1 | 8.7E-1 | |  | GOTERM\_BP\_5 | initiation of dorsal closure | **RT** |  | 3 | 6.0E-1 | 9.9E-1 | | Annotation Cluster 62 | | Enrichment Score: 0.56 |  |  | Count | P\_Value | Benjamini | | --- | --- | --- | --- | --- | --- | --- | --- | |  | GOTERM\_BP\_5 | actin-mediated cell contraction | **RT** |  | 5 | 1.9E-1 | 8.7E-1 | |  | GOTERM\_BP\_5 | epithelial cell morphogenesis | **RT** |  | 5 | 2.8E-1 | 9.3E-1 | |  | GOTERM\_BP\_5 | epithelial cell morphogenesis involved in gastrulation | **RT** |  | 3 | 3.3E-1 | 9.5E-1 | |  | GOTERM\_BP\_5 | apical constriction involved in gastrulation | **RT** |  | 3 | 3.3E-1 | 9.5E-1 | | Annotation Cluster 63 | | Enrichment Score: 0.56 |  |  | Count | P\_Value | Benjamini | | --- | --- | --- | --- | --- | --- | --- | --- | |  | GOTERM\_CC\_5 | cell cortex part | **RT** |  | 13 | 1.9E-1 | 9.5E-1 | |  | GOTERM\_CC\_5 | apical cortex | **RT** |  | 8 | 2.5E-1 | 9.6E-1 | |  | GOTERM\_CC\_5 | cell cortex region | **RT** |  | 9 | 3.0E-1 | 9.6E-1 | |  | GOTERM\_BP\_5 | establishment or maintenance of polarity of embryonic epithelium | **RT** |  | 3 | 4.0E-1 | 9.7E-1 | | Annotation Cluster 64 | | Enrichment Score: 0.56 |  |  | Count | P\_Value | Benjamini | | --- | --- | --- | --- | --- | --- | --- | --- | |  | GOTERM\_BP\_5 | actin cytoskeleton reorganization | **RT** |  | 7 | 2.4E-1 | 9.1E-1 | |  | GOTERM\_BP\_5 | positive regulation of wound healing | **RT** |  | 4 | 2.4E-1 | 9.1E-1 | |  | GOTERM\_BP\_5 | regulation of wound healing | **RT** |  | 4 | 2.4E-1 | 9.1E-1 | |  | GOTERM\_BP\_5 | positive regulation of response to wounding | **RT** |  | 4 | 4.2E-1 | 9.7E-1 | | Annotation Cluster 65 | | Enrichment Score: 0.55 |  |  | Count | P\_Value | Benjamini | | --- | --- | --- | --- | --- | --- | --- | --- | |  | GOTERM\_BP\_5 | regulation of antimicrobial humoral response | **RT** |  | 8 | 1.6E-1 | 8.5E-1 | |  | GOTERM\_BP\_5 | negative regulation of antimicrobial humoral response | **RT** |  | 3 | 3.3E-1 | 9.5E-1 | |  | GOTERM\_BP\_5 | negative regulation of humoral immune response | **RT** |  | 3 | 4.0E-1 | 9.7E-1 | | Annotation Cluster 66 | | Enrichment Score: 0.55 |  |  | Count | P\_Value | Benjamini | | --- | --- | --- | --- | --- | --- | --- | --- | |  | GOTERM\_CC\_5 | BAF-type complex | **RT** |  | 6 | 9.8E-2 | 8.9E-1 | |  | GOTERM\_CC\_5 | brahma complex | **RT** |  | 5 | 2.0E-1 | 9.5E-1 | |  | GOTERM\_CC\_5 | SWI/SNF superfamily-type complex | **RT** |  | 8 | 3.6E-1 | 9.6E-1 | |  | GOTERM\_BP\_5 | chromatin remodeling | **RT** |  | 10 | 8.6E-1 | 1.0E0 | | Annotation Cluster 67 | | Enrichment Score: 0.55 |  |  | Count | P\_Value | Benjamini | | --- | --- | --- | --- | --- | --- | --- | --- | |  | GOTERM\_BP\_5 | negative regulation of cell fate commitment | **RT** |  | 6 | 1.8E-1 | 8.7E-1 | |  | GOTERM\_BP\_5 | regulation of cell fate specification | **RT** |  | 6 | 2.6E-1 | 9.2E-1 | |  | GOTERM\_BP\_5 | negative regulation of cell fate specification | **RT** |  | 5 | 2.8E-1 | 9.3E-1 | |  | GOTERM\_BP\_5 | cell fate specification | **RT** |  | 9 | 4.9E-1 | 9.8E-1 | | Annotation Cluster 68 | | Enrichment Score: 0.55 |  |  | Count | P\_Value | Benjamini | | --- | --- | --- | --- | --- | --- | --- | --- | |  | GOTERM\_BP\_5 | negative regulation of nervous system development | **RT** |  | 23 | 8.1E-2 | 7.3E-1 | |  | GOTERM\_BP\_5 | synaptic growth at neuromuscular junction | **RT** |  | 24 | 1.4E-1 | 8.2E-1 | |  | GOTERM\_BP\_5 | negative regulation of neuromuscular junction development | **RT** |  | 10 | 2.7E-1 | 9.3E-1 | |  | GOTERM\_BP\_5 | negative regulation of synaptic growth at neuromuscular junction | **RT** |  | 10 | 2.7E-1 | 9.3E-1 | |  | GOTERM\_BP\_5 | negative regulation of synapse assembly | **RT** |  | 10 | 2.7E-1 | 9.3E-1 | |  | GOTERM\_BP\_5 | negative regulation of developmental growth | **RT** |  | 14 | 3.4E-1 | 9.5E-1 | |  | GOTERM\_BP\_5 | synapse assembly | **RT** |  | 31 | 3.5E-1 | 9.5E-1 | |  | GOTERM\_BP\_5 | regulation of neuromuscular junction development | **RT** |  | 20 | 4.3E-1 | 9.7E-1 | |  | GOTERM\_BP\_5 | regulation of synaptic growth at neuromuscular junction | **RT** |  | 19 | 4.7E-1 | 9.8E-1 | |  | GOTERM\_BP\_5 | regulation of synapse assembly | **RT** |  | 19 | 5.8E-1 | 9.9E-1 | | Annotation Cluster 69 | | Enrichment Score: 0.55 |  |  | Count | P\_Value | Benjamini | | --- | --- | --- | --- | --- | --- | --- | --- | |  | GOTERM\_BP\_5 | lytic vacuole organization | **RT** |  | 7 | 5.2E-2 | 6.5E-1 | |  | GOTERM\_BP\_5 | lysosomal transport | **RT** |  | 6 | 1.5E-1 | 8.3E-1 | |  | GOTERM\_CC\_5 | vacuolar membrane | **RT** |  | 18 | 1.7E-1 | 9.4E-1 | |  | GOTERM\_BP\_5 | endosome to lysosome transport | **RT** |  | 4 | 1.9E-1 | 8.7E-1 | |  | GOTERM\_BP\_5 | endosomal transport | **RT** |  | 17 | 2.1E-1 | 8.9E-1 | |  | GOTERM\_CC\_5 | vacuolar part | **RT** |  | 18 | 2.4E-1 | 9.6E-1 | |  | GOTERM\_CC\_5 | endosome membrane | **RT** |  | 10 | 2.5E-1 | 9.5E-1 | |  | GOTERM\_CC\_5 | late endosome membrane | **RT** |  | 6 | 2.8E-1 | 9.6E-1 | |  | GOTERM\_CC\_5 | lysosomal membrane | **RT** |  | 5 | 3.0E-1 | 9.6E-1 | |  | GOTERM\_CC\_5 | lytic vacuole membrane | **RT** |  | 5 | 3.0E-1 | 9.6E-1 | |  | GOTERM\_CC\_5 | endosomal part | **RT** |  | 10 | 4.7E-1 | 9.6E-1 | |  | GOTERM\_CC\_5 | lytic vacuole | **RT** |  | 10 | 5.3E-1 | 9.8E-1 | |  | GOTERM\_CC\_5 | vacuole | **RT** |  | 37 | 5.6E-1 | 9.8E-1 | |  | GOTERM\_CC\_5 | late endosome | **RT** |  | 9 | 7.5E-1 | 9.9E-1 | |  | GOTERM\_CC\_5 | endosome | **RT** |  | 23 | 7.7E-1 | 9.9E-1 | | Annotation Cluster 70 | | Enrichment Score: 0.55 |  |  | Count | P\_Value | Benjamini | | --- | --- | --- | --- | --- | --- | --- | --- | |  | GOTERM\_BP\_5 | regulation of intrinsic apoptotic signaling pathway | **RT** |  | 3 | 1.7E-1 | 8.6E-1 | |  | GOTERM\_BP\_5 | regulation of apoptotic signaling pathway | **RT** |  | 5 | 1.9E-1 | 8.7E-1 | |  | GOTERM\_BP\_5 | apoptotic signaling pathway | **RT** |  | 8 | 2.5E-1 | 9.2E-1 | |  | GOTERM\_BP\_5 | intrinsic apoptotic signaling pathway | **RT** |  | 5 | 4.2E-1 | 9.7E-1 | |  | GOTERM\_BP\_5 | intrinsic apoptotic signaling pathway in response to DNA damage | **RT** |  | 3 | 5.4E-1 | 9.9E-1 | | Annotation Cluster 71 | | Enrichment Score: 0.55 |  |  | Count | P\_Value | Benjamini | | --- | --- | --- | --- | --- | --- | --- | --- | |  | GOTERM\_BP\_5 | regulation of mRNA metabolic process | **RT** |  | 16 | 2.4E-1 | 9.1E-1 | |  | GOTERM\_BP\_5 | regulation of RNA splicing | **RT** |  | 15 | 2.8E-1 | 9.3E-1 | |  | GOTERM\_BP\_5 | regulation of mRNA processing | **RT** |  | 15 | 2.8E-1 | 9.3E-1 | |  | GOTERM\_BP\_5 | mRNA metabolic process | **RT** |  | 44 | 3.5E-1 | 9.6E-1 | | Annotation Cluster 72 | | Enrichment Score: 0.54 |  |  | Count | P\_Value | Benjamini | | --- | --- | --- | --- | --- | --- | --- | --- | |  | GOTERM\_BP\_5 | imaginal disc-derived wing hair organization | **RT** |  | 9 | 1.8E-1 | 8.7E-1 | |  | GOTERM\_BP\_5 | epidermal cell differentiation | **RT** |  | 9 | 2.1E-1 | 8.9E-1 | |  | GOTERM\_BP\_5 | establishment of imaginal disc-derived wing hair orientation | **RT** |  | 4 | 6.2E-1 | 1.0E0 | | Annotation Cluster 73 | | Enrichment Score: 0.54 |  |  | Count | P\_Value | Benjamini | | --- | --- | --- | --- | --- | --- | --- | --- | |  | GOTERM\_BP\_5 | regulation of gastrulation | **RT** |  | 4 | 5.2E-2 | 6.5E-1 | |  | GOTERM\_BP\_5 | establishment or maintenance of cytoskeleton polarity involved in gastrulation | **RT** |  | 3 | 1.7E-1 | 8.6E-1 | |  | GOTERM\_BP\_5 | establishment or maintenance of cytoskeleton polarity involved in ameboidal cell migration | **RT** |  | 3 | 1.7E-1 | 8.6E-1 | |  | GOTERM\_BP\_5 | establishment of cell polarity involved in ameboidal cell migration | **RT** |  | 3 | 1.7E-1 | 8.6E-1 | |  | GOTERM\_BP\_5 | mesectoderm development | **RT** |  | 3 | 2.5E-1 | 9.2E-1 | |  | GOTERM\_BP\_5 | convergent extension involved in gastrulation | **RT** |  | 3 | 2.5E-1 | 9.2E-1 | |  | GOTERM\_CC\_5 | extrinsic component of plasma membrane | **RT** |  | 8 | 3.2E-1 | 9.6E-1 | |  | GOTERM\_CC\_5 | heterotrimeric G-protein complex | **RT** |  | 4 | 3.2E-1 | 9.6E-1 | |  | GOTERM\_BP\_5 | regulation of myosin II filament organization | **RT** |  | 3 | 3.3E-1 | 9.5E-1 | |  | GOTERM\_BP\_5 | convergent extension | **RT** |  | 3 | 4.0E-1 | 9.7E-1 | |  | GOTERM\_BP\_5 | establishment or maintenance of cytoskeleton polarity | **RT** |  | 9 | 4.2E-1 | 9.7E-1 | |  | GOTERM\_CC\_5 | extrinsic component of cytoplasmic side of plasma membrane | **RT** |  | 5 | 6.3E-1 | 9.8E-1 | |  | GOTERM\_CC\_5 | cytoplasmic side of plasma membrane | **RT** |  | 6 | 6.8E-1 | 9.9E-1 | |  | GOTERM\_BP\_5 | cell migration involved in gastrulation | **RT** |  | 3 | 7.0E-1 | 1.0E0 | | Annotation Cluster 74 | | Enrichment Score: 0.54 |  |  | Count | P\_Value | Benjamini | | --- | --- | --- | --- | --- | --- | --- | --- | |  | GOTERM\_BP\_5 | melanization defense response | **RT** |  | 8 | 2.2E-1 | 9.0E-1 | |  | GOTERM\_BP\_5 | melanin metabolic process | **RT** |  | 8 | 2.9E-1 | 9.3E-1 | |  | GOTERM\_BP\_5 | melanotic encapsulation of foreign target | **RT** |  | 6 | 3.8E-1 | 9.6E-1 | | Annotation Cluster 75 | | Enrichment Score: 0.54 |  |  | Count | P\_Value | Benjamini | | --- | --- | --- | --- | --- | --- | --- | --- | |  | GOTERM\_BP\_5 | regulation of lipid biosynthetic process | **RT** |  | 5 | 1.9E-1 | 8.7E-1 | |  | GOTERM\_BP\_5 | positive regulation of lipid biosynthetic process | **RT** |  | 3 | 3.3E-1 | 9.5E-1 | |  | GOTERM\_BP\_5 | positive regulation of lipid metabolic process | **RT** |  | 3 | 4.0E-1 | 9.7E-1 | | Annotation Cluster 76 | | Enrichment Score: 0.54 |  |  | Count | P\_Value | Benjamini | | --- | --- | --- | --- | --- | --- | --- | --- | |  | GOTERM\_BP\_5 | phospholipid metabolic process | **RT** |  | 18 | 9.5E-2 | 7.6E-1 | |  | GOTERM\_BP\_5 | glycerolipid metabolic process | **RT** |  | 13 | 3.9E-1 | 9.7E-1 | |  | GOTERM\_BP\_5 | glycerophospholipid metabolic process | **RT** |  | 9 | 6.6E-1 | 1.0E0 | | Annotation Cluster 77 | | Enrichment Score: 0.53 |  |  | Count | P\_Value | Benjamini | | --- | --- | --- | --- | --- | --- | --- | --- | |  | GOTERM\_BP\_5 | cellular protein modification process | **RT** |  | 156 | 2.2E-1 | 9.0E-1 | |  | GOTERM\_BP\_5 | protein modification process | **RT** |  | 156 | 2.2E-1 | 9.0E-1 | |  | GOTERM\_BP\_5 | cellular protein metabolic process | **RT** |  | 229 | 5.0E-1 | 9.8E-1 | | Annotation Cluster 78 | | Enrichment Score: 0.53 |  |  | Count | P\_Value | Benjamini | | --- | --- | --- | --- | --- | --- | --- | --- | |  | GOTERM\_BP\_5 | development of primary female sexual characteristics | **RT** |  | 4 | 1.9E-1 | 8.7E-1 | |  | GOTERM\_BP\_5 | female gonad development | **RT** |  | 4 | 1.9E-1 | 8.7E-1 | |  | GOTERM\_BP\_5 | reproductive system development | **RT** |  | 10 | 3.0E-1 | 9.4E-1 | |  | GOTERM\_BP\_5 | reproductive structure development | **RT** |  | 10 | 3.0E-1 | 9.4E-1 | |  | GOTERM\_BP\_5 | development of primary sexual characteristics | **RT** |  | 6 | 3.8E-1 | 9.6E-1 | |  | GOTERM\_BP\_5 | gonad development | **RT** |  | 6 | 3.8E-1 | 9.6E-1 | |  | GOTERM\_BP\_5 | female sex differentiation | **RT** |  | 4 | 4.2E-1 | 9.7E-1 | | Annotation Cluster 79 | | Enrichment Score: 0.52 |  |  | Count | P\_Value | Benjamini | | --- | --- | --- | --- | --- | --- | --- | --- | |  | GOTERM\_BP\_5 | organophosphate ester transport | **RT** |  | 5 | 2.3E-1 | 9.0E-1 | |  | GOTERM\_BP\_5 | nucleotide transport | **RT** |  | 3 | 3.3E-1 | 9.5E-1 | |  | GOTERM\_BP\_5 | carbohydrate derivative transport | **RT** |  | 4 | 3.6E-1 | 9.6E-1 | | Annotation Cluster 80 | | Enrichment Score: 0.52 |  |  | Count | P\_Value | Benjamini | | --- | --- | --- | --- | --- | --- | --- | --- | |  | GOTERM\_BP\_5 | vacuole fusion | **RT** |  | 6 | 1.5E-1 | 8.3E-1 | |  | GOTERM\_BP\_5 | lysosomal transport | **RT** |  | 6 | 1.5E-1 | 8.3E-1 | |  | GOTERM\_BP\_5 | single-organism membrane fusion | **RT** |  | 12 | 2.1E-1 | 9.0E-1 | |  | GOTERM\_BP\_5 | vesicle fusion | **RT** |  | 10 | 2.7E-1 | 9.3E-1 | |  | GOTERM\_BP\_5 | autophagosome maturation | **RT** |  | 5 | 2.8E-1 | 9.3E-1 | |  | GOTERM\_BP\_5 | organelle membrane fusion | **RT** |  | 10 | 3.3E-1 | 9.5E-1 | |  | GOTERM\_CC\_5 | SNARE complex | **RT** |  | 7 | 3.4E-1 | 9.5E-1 | |  | GOTERM\_BP\_5 | vesicle docking involved in exocytosis | **RT** |  | 4 | 7.4E-1 | 1.0E0 | |  | GOTERM\_BP\_5 | vesicle docking | **RT** |  | 4 | 7.7E-1 | 1.0E0 | | Annotation Cluster 81 | | Enrichment Score: 0.51 |  |  | Count | P\_Value | Benjamini | | --- | --- | --- | --- | --- | --- | --- | --- | |  | GOTERM\_BP\_5 | regulation of smoothened signaling pathway | **RT** |  | 8 | 1.4E-1 | 8.2E-1 | |  | GOTERM\_BP\_5 | positive regulation of smoothened signaling pathway | **RT** |  | 4 | 1.9E-1 | 8.7E-1 | |  | GOTERM\_BP\_5 | smoothened signaling pathway | **RT** |  | 9 | 4.2E-1 | 9.7E-1 | |  | GOTERM\_BP\_5 | negative regulation of smoothened signaling pathway | **RT** |  | 3 | 8.2E-1 | 1.0E0 | | Annotation Cluster 82 | | Enrichment Score: 0.5 |  |  | Count | P\_Value | Benjamini | | --- | --- | --- | --- | --- | --- | --- | --- | |  | GOTERM\_BP\_5 | hexose metabolic process | **RT** |  | 8 | 9.2E-2 | 7.5E-1 | |  | GOTERM\_BP\_5 | glycogen metabolic process | **RT** |  | 4 | 3.6E-1 | 9.6E-1 | |  | GOTERM\_BP\_5 | glucan metabolic process | **RT** |  | 4 | 3.6E-1 | 9.6E-1 | |  | GOTERM\_BP\_5 | cellular glucan metabolic process | **RT** |  | 4 | 3.6E-1 | 9.6E-1 | |  | GOTERM\_BP\_5 | energy reserve metabolic process | **RT** |  | 4 | 3.6E-1 | 9.6E-1 | |  | GOTERM\_BP\_5 | cellular polysaccharide metabolic process | **RT** |  | 4 | 6.2E-1 | 1.0E0 | | Annotation Cluster 83 | | Enrichment Score: 0.5 |  |  | Count | P\_Value | Benjamini | | --- | --- | --- | --- | --- | --- | --- | --- | |  | GOTERM\_BP\_5 | positive regulation of MAPK cascade | **RT** |  | 15 | 2.0E-1 | 8.8E-1 | |  | GOTERM\_BP\_5 | positive regulation of small GTPase mediated signal transduction | **RT** |  | 11 | 2.8E-1 | 9.3E-1 | |  | GOTERM\_BP\_5 | ERK1 and ERK2 cascade | **RT** |  | 7 | 5.7E-1 | 9.9E-1 | | Annotation Cluster 84 | | Enrichment Score: 0.49 |  |  | Count | P\_Value | Benjamini | | --- | --- | --- | --- | --- | --- | --- | --- | |  | GOTERM\_BP\_5 | negative regulation of cell motility | **RT** |  | 3 | 2.5E-1 | 9.2E-1 | |  | GOTERM\_BP\_5 | negative regulation of cell migration | **RT** |  | 3 | 2.5E-1 | 9.2E-1 | |  | GOTERM\_BP\_5 | negative regulation of cellular component movement | **RT** |  | 3 | 5.4E-1 | 9.9E-1 | | Annotation Cluster 85 | | Enrichment Score: 0.48 |  |  | Count | P\_Value | Benjamini | | --- | --- | --- | --- | --- | --- | --- | --- | |  | GOTERM\_BP\_5 | negative regulation of stress-activated protein kinase signaling cascade | **RT** |  | 6 | 2.2E-1 | 9.0E-1 | |  | GOTERM\_BP\_5 | negative regulation of MAPK cascade | **RT** |  | 7 | 2.4E-1 | 9.1E-1 | |  | GOTERM\_BP\_5 | negative regulation of intracellular signal transduction | **RT** |  | 16 | 3.5E-1 | 9.5E-1 | |  | GOTERM\_BP\_5 | negative regulation of transferase activity | **RT** |  | 8 | 3.9E-1 | 9.7E-1 | |  | GOTERM\_BP\_5 | negative regulation of phosphorus metabolic process | **RT** |  | 13 | 3.9E-1 | 9.7E-1 | |  | GOTERM\_BP\_5 | negative regulation of phosphate metabolic process | **RT** |  | 13 | 3.9E-1 | 9.7E-1 | |  | GOTERM\_BP\_5 | negative regulation of protein modification process | **RT** |  | 14 | 4.2E-1 | 9.7E-1 | | Annotation Cluster 86 | | Enrichment Score: 0.44 |  |  | Count | P\_Value | Benjamini | | --- | --- | --- | --- | --- | --- | --- | --- | |  | GOTERM\_BP\_5 | single-organism intracellular transport | **RT** |  | 30 | 1.4E-1 | 8.3E-1 | |  | GOTERM\_BP\_5 | protein transport | **RT** |  | 59 | 3.2E-1 | 9.5E-1 | |  | GOTERM\_BP\_5 | protein import into nucleus, translocation | **RT** |  | 3 | 3.3E-1 | 9.5E-1 | |  | GOTERM\_BP\_5 | protein targeting | **RT** |  | 25 | 3.5E-1 | 9.5E-1 | |  | GOTERM\_BP\_5 | protein targeting to nucleus | **RT** |  | 9 | 3.9E-1 | 9.7E-1 | |  | GOTERM\_BP\_5 | single-organism nuclear import | **RT** |  | 9 | 3.9E-1 | 9.7E-1 | |  | GOTERM\_BP\_5 | protein import into nucleus | **RT** |  | 9 | 3.9E-1 | 9.7E-1 | |  | GOTERM\_BP\_5 | establishment of protein localization to organelle | **RT** |  | 21 | 4.1E-1 | 9.7E-1 | |  | GOTERM\_BP\_5 | intracellular protein transport | **RT** |  | 42 | 4.1E-1 | 9.7E-1 | |  | GOTERM\_BP\_5 | protein import | **RT** |  | 14 | 4.2E-1 | 9.7E-1 | |  | GOTERM\_BP\_5 | protein localization to organelle | **RT** |  | 24 | 6.2E-1 | 9.9E-1 | | Annotation Cluster 87 | | Enrichment Score: 0.44 |  |  | Count | P\_Value | Benjamini | | --- | --- | --- | --- | --- | --- | --- | --- | |  | GOTERM\_BP\_5 | positive regulation of proteolysis | **RT** |  | 10 | 2.2E-1 | 9.0E-1 | |  | GOTERM\_BP\_5 | positive regulation of cellular catabolic process | **RT** |  | 7 | 2.7E-1 | 9.3E-1 | |  | GOTERM\_BP\_5 | regulation of proteolysis | **RT** |  | 15 | 3.5E-1 | 9.5E-1 | |  | GOTERM\_BP\_5 | positive regulation of protein catabolic process | **RT** |  | 7 | 3.9E-1 | 9.6E-1 | |  | GOTERM\_BP\_5 | positive regulation of cellular protein catabolic process | **RT** |  | 5 | 4.2E-1 | 9.7E-1 | |  | GOTERM\_BP\_5 | regulation of cellular protein catabolic process | **RT** |  | 6 | 4.6E-1 | 9.8E-1 | |  | GOTERM\_BP\_5 | regulation of protein catabolic process | **RT** |  | 8 | 5.6E-1 | 9.9E-1 | | Annotation Cluster 88 | | Enrichment Score: 0.43 |  |  | Count | P\_Value | Benjamini | | --- | --- | --- | --- | --- | --- | --- | --- | |  | GOTERM\_BP\_5 | peptide metabolic process | **RT** |  | 76 | 2.5E-1 | 9.2E-1 | |  | GOTERM\_BP\_5 | amide biosynthetic process | **RT** |  | 70 | 2.5E-1 | 9.2E-1 | |  | GOTERM\_BP\_5 | peptide biosynthetic process | **RT** |  | 68 | 2.9E-1 | 9.4E-1 | |  | GOTERM\_CC\_5 | ribosome | **RT** |  | 32 | 4.1E-1 | 9.6E-1 | |  | GOTERM\_BP\_5 | translation | **RT** |  | 62 | 4.5E-1 | 9.8E-1 | |  | GOTERM\_CC\_5 | ribosomal subunit | **RT** |  | 30 | 5.0E-1 | 9.7E-1 | |  | GOTERM\_CC\_5 | intracellular ribonucleoprotein complex | **RT** |  | 76 | 5.6E-1 | 9.8E-1 | | Annotation Cluster 89 | | Enrichment Score: 0.42 |  |  | Count | P\_Value | Benjamini | | --- | --- | --- | --- | --- | --- | --- | --- | |  | GOTERM\_CC\_5 | intracellular organelle lumen | **RT** |  | 112 | 2.9E-1 | 9.5E-1 | |  | GOTERM\_CC\_5 | nucleoplasm | **RT** |  | 62 | 3.1E-1 | 9.6E-1 | |  | GOTERM\_CC\_5 | nuclear part | **RT** |  | 131 | 3.1E-1 | 9.6E-1 | |  | GOTERM\_CC\_5 | nuclear lumen | **RT** |  | 88 | 3.8E-1 | 9.5E-1 | |  | GOTERM\_CC\_5 | nucleoplasm part | **RT** |  | 38 | 7.4E-1 | 9.9E-1 | | Annotation Cluster 90 | | Enrichment Score: 0.41 |  |  | Count | P\_Value | Benjamini | | --- | --- | --- | --- | --- | --- | --- | --- | |  | GOTERM\_BP\_5 | regulation of antimicrobial humoral response | **RT** |  | 8 | 1.6E-1 | 8.5E-1 | |  | GOTERM\_BP\_5 | antimicrobial peptide production | **RT** |  | 7 | 2.0E-1 | 8.9E-1 | |  | GOTERM\_BP\_5 | regulation of antimicrobial peptide production | **RT** |  | 7 | 2.0E-1 | 8.9E-1 | |  | GOTERM\_BP\_5 | antimicrobial peptide biosynthetic process | **RT** |  | 5 | 2.3E-1 | 9.0E-1 | |  | GOTERM\_BP\_5 | regulation of antimicrobial peptide biosynthetic process | **RT** |  | 6 | 2.6E-1 | 9.2E-1 | |  | GOTERM\_BP\_5 | antimicrobial humoral response | **RT** |  | 12 | 2.6E-1 | 9.2E-1 | |  | GOTERM\_BP\_5 | positive regulation of antimicrobial peptide biosynthetic process | **RT** |  | 4 | 3.6E-1 | 9.6E-1 | |  | GOTERM\_BP\_5 | regulation of defense response to bacterium | **RT** |  | 5 | 3.7E-1 | 9.6E-1 | |  | GOTERM\_BP\_5 | positive regulation of antimicrobial peptide production | **RT** |  | 4 | 4.2E-1 | 9.7E-1 | |  | GOTERM\_BP\_5 | positive regulation of production of molecular mediator of immune response | **RT** |  | 4 | 4.2E-1 | 9.7E-1 | |  | GOTERM\_BP\_5 | positive regulation of antimicrobial humoral response | **RT** |  | 4 | 4.2E-1 | 9.7E-1 | |  | GOTERM\_BP\_5 | positive regulation of humoral immune response | **RT** |  | 4 | 4.2E-1 | 9.7E-1 | |  | GOTERM\_BP\_5 | antibacterial peptide production | **RT** |  | 3 | 6.0E-1 | 9.9E-1 | |  | GOTERM\_BP\_5 | regulation of antibacterial peptide production | **RT** |  | 3 | 6.0E-1 | 9.9E-1 | |  | GOTERM\_BP\_5 | antibacterial peptide biosynthetic process | **RT** |  | 3 | 6.0E-1 | 9.9E-1 | |  | GOTERM\_BP\_5 | regulation of antibacterial peptide biosynthetic process | **RT** |  | 3 | 6.0E-1 | 9.9E-1 | |  | GOTERM\_BP\_5 | antibacterial humoral response | **RT** |  | 3 | 7.5E-1 | 1.0E0 | |  | GOTERM\_BP\_5 | positive regulation of cellular amide metabolic process | **RT** |  | 5 | 8.9E-1 | 1.0E0 | | Annotation Cluster 91 | | Enrichment Score: 0.41 |  |  | Count | P\_Value | Benjamini | | --- | --- | --- | --- | --- | --- | --- | --- | |  | GOTERM\_CC\_5 | ATG1/ULK1 kinase complex | **RT** |  | 3 | 1.8E-1 | 9.5E-1 | |  | GOTERM\_BP\_5 | larval midgut cell programmed cell death | **RT** |  | 5 | 5.1E-1 | 9.9E-1 | |  | GOTERM\_BP\_5 | larval midgut histolysis | **RT** |  | 5 | 6.4E-1 | 1.0E0 | | Annotation Cluster 92 | | Enrichment Score: 0.41 |  |  | Count | P\_Value | Benjamini | | --- | --- | --- | --- | --- | --- | --- | --- | |  | GOTERM\_BP\_5 | unidimensional cell growth | **RT** |  | 5 | 1.9E-1 | 8.7E-1 | |  | GOTERM\_BP\_5 | digestive tract morphogenesis | **RT** |  | 10 | 1.9E-1 | 8.8E-1 | |  | GOTERM\_BP\_5 | digestive tract development | **RT** |  | 12 | 2.6E-1 | 9.2E-1 | |  | GOTERM\_BP\_5 | digestive system development | **RT** |  | 12 | 2.6E-1 | 9.2E-1 | |  | GOTERM\_BP\_5 | hindgut development | **RT** |  | 8 | 2.9E-1 | 9.3E-1 | |  | GOTERM\_BP\_5 | hindgut morphogenesis | **RT** |  | 8 | 2.9E-1 | 9.3E-1 | |  | GOTERM\_BP\_5 | renal tubule morphogenesis | **RT** |  | 6 | 3.8E-1 | 9.6E-1 | |  | GOTERM\_BP\_5 | Malpighian tubule morphogenesis | **RT** |  | 6 | 3.8E-1 | 9.6E-1 | |  | GOTERM\_BP\_5 | embryonic hindgut morphogenesis | **RT** |  | 6 | 5.0E-1 | 9.9E-1 | |  | GOTERM\_BP\_5 | renal tubule development | **RT** |  | 6 | 7.1E-1 | 1.0E0 | |  | GOTERM\_BP\_5 | Malpighian tubule development | **RT** |  | 6 | 7.1E-1 | 1.0E0 | |  | GOTERM\_BP\_5 | renal system development | **RT** |  | 6 | 8.3E-1 | 1.0E0 | |  | GOTERM\_BP\_5 | urogenital system development | **RT** |  | 6 | 8.3E-1 | 1.0E0 | | Annotation Cluster 93 | | Enrichment Score: 0.4 |  |  | Count | P\_Value | Benjamini | | --- | --- | --- | --- | --- | --- | --- | --- | |  | GOTERM\_BP\_5 | positive regulation of cell death | **RT** |  | 12 | 2.9E-1 | 9.3E-1 | |  | GOTERM\_BP\_5 | positive regulation of apoptotic process | **RT** |  | 9 | 3.3E-1 | 9.5E-1 | |  | GOTERM\_BP\_5 | positive regulation of programmed cell death | **RT** |  | 10 | 4.2E-1 | 9.7E-1 | |  | GOTERM\_BP\_5 | regulation of peptidase activity | **RT** |  | 6 | 6.2E-1 | 9.9E-1 | | Annotation Cluster 94 | | Enrichment Score: 0.4 |  |  | Count | P\_Value | Benjamini | | --- | --- | --- | --- | --- | --- | --- | --- | |  | GOTERM\_BP\_5 | negative regulation of signal transduction | **RT** |  | 40 | 3.4E-1 | 9.5E-1 | |  | GOTERM\_BP\_5 | negative regulation of intracellular signal transduction | **RT** |  | 16 | 3.5E-1 | 9.5E-1 | |  | GOTERM\_BP\_5 | negative regulation of cell communication | **RT** |  | 40 | 5.4E-1 | 9.9E-1 | | Annotation Cluster 95 | | Enrichment Score: 0.39 |  |  | Count | P\_Value | Benjamini | | --- | --- | --- | --- | --- | --- | --- | --- | |  | GOTERM\_CC\_5 | SWI/SNF superfamily-type complex | **RT** |  | 8 | 3.6E-1 | 9.6E-1 | |  | GOTERM\_CC\_5 | DNA helicase complex | **RT** |  | 3 | 4.2E-1 | 9.6E-1 | |  | GOTERM\_CC\_5 | Ino80 complex | **RT** |  | 3 | 4.2E-1 | 9.6E-1 | |  | GOTERM\_CC\_5 | INO80-type complex | **RT** |  | 3 | 4.2E-1 | 9.6E-1 | | Annotation Cluster 96 | | Enrichment Score: 0.39 |  |  | Count | P\_Value | Benjamini | | --- | --- | --- | --- | --- | --- | --- | --- | |  | GOTERM\_BP\_5 | regulation of filopodium assembly | **RT** |  | 7 | 1.7E-1 | 8.6E-1 | |  | GOTERM\_BP\_5 | filopodium assembly | **RT** |  | 7 | 2.0E-1 | 8.9E-1 | |  | GOTERM\_BP\_5 | lamellipodium assembly | **RT** |  | 5 | 2.8E-1 | 9.3E-1 | |  | GOTERM\_BP\_5 | regulation of cell projection assembly | **RT** |  | 8 | 2.9E-1 | 9.3E-1 | |  | GOTERM\_BP\_5 | positive regulation of lamellipodium assembly | **RT** |  | 3 | 4.8E-1 | 9.8E-1 | |  | GOTERM\_BP\_5 | positive regulation of lamellipodium organization | **RT** |  | 3 | 4.8E-1 | 9.8E-1 | |  | GOTERM\_BP\_5 | positive regulation of filopodium assembly | **RT** |  | 4 | 5.2E-1 | 9.9E-1 | |  | GOTERM\_BP\_5 | positive regulation of cell projection organization | **RT** |  | 9 | 5.5E-1 | 9.9E-1 | |  | GOTERM\_BP\_5 | regulation of lamellipodium assembly | **RT** |  | 3 | 8.4E-1 | 1.0E0 | |  | GOTERM\_BP\_5 | regulation of lamellipodium organization | **RT** |  | 3 | 8.4E-1 | 1.0E0 | | Annotation Cluster 97 | | Enrichment Score: 0.39 |  |  | Count | P\_Value | Benjamini | | --- | --- | --- | --- | --- | --- | --- | --- | |  | GOTERM\_CC\_5 | cytosolic large ribosomal subunit | **RT** |  | 14 | 2.0E-1 | 9.5E-1 | |  | GOTERM\_CC\_5 | large ribosomal subunit | **RT** |  | 20 | 3.1E-1 | 9.6E-1 | |  | GOTERM\_CC\_5 | ribosome | **RT** |  | 32 | 4.1E-1 | 9.6E-1 | |  | GOTERM\_CC\_5 | cytosolic ribosome | **RT** |  | 20 | 4.5E-1 | 9.6E-1 | |  | GOTERM\_CC\_5 | ribosomal subunit | **RT** |  | 30 | 5.0E-1 | 9.7E-1 | |  | GOTERM\_CC\_5 | cytosolic part | **RT** |  | 22 | 8.1E-1 | 1.0E0 | | Annotation Cluster 98 | | Enrichment Score: 0.39 |  |  | Count | P\_Value | Benjamini | | --- | --- | --- | --- | --- | --- | --- | --- | |  | GOTERM\_BP\_5 | negative regulation of neurogenesis | **RT** |  | 13 | 2.4E-1 | 9.2E-1 | |  | GOTERM\_BP\_5 | regulation of neuroblast proliferation | **RT** |  | 6 | 4.2E-1 | 9.7E-1 | |  | GOTERM\_BP\_5 | negative regulation of neuroblast proliferation | **RT** |  | 5 | 4.7E-1 | 9.8E-1 | |  | GOTERM\_BP\_5 | negative regulation of neural precursor cell proliferation | **RT** |  | 5 | 4.7E-1 | 9.8E-1 | |  | GOTERM\_BP\_5 | negative regulation of stem cell proliferation | **RT** |  | 5 | 5.1E-1 | 9.9E-1 | | Annotation Cluster 99 | | Enrichment Score: 0.38 |  |  | Count | P\_Value | Benjamini | | --- | --- | --- | --- | --- | --- | --- | --- | |  | GOTERM\_CC\_5 | plasma membrane proton-transporting V-type ATPase complex | **RT** |  | 6 | 1.3E-1 | 9.2E-1 | |  | GOTERM\_CC\_5 | proton-transporting V-type ATPase, V0 domain | **RT** |  | 4 | 1.5E-1 | 9.3E-1 | |  | GOTERM\_CC\_5 | vacuolar proton-transporting V-type ATPase, V0 domain | **RT** |  | 4 | 1.5E-1 | 9.3E-1 | |  | GOTERM\_MF\_5 | ATPase coupled ion transmembrane transporter activity | **RT** |  | 12 | 1.8E-1 | 9.8E-1 | |  | GOTERM\_MF\_5 | P-P-bond-hydrolysis-driven transmembrane transporter activity | **RT** |  | 19 | 2.5E-1 | 9.9E-1 | |  | GOTERM\_MF\_5 | ATPase activity, coupled to transmembrane movement of substances | **RT** |  | 16 | 2.7E-1 | 9.9E-1 | |  | GOTERM\_MF\_5 | active ion transmembrane transporter activity | **RT** |  | 12 | 3.1E-1 | 9.8E-1 | |  | GOTERM\_BP\_5 | ATP hydrolysis coupled transmembrane transport | **RT** |  | 7 | 3.1E-1 | 9.4E-1 | |  | GOTERM\_CC\_5 | proton-transporting V-type ATPase complex | **RT** |  | 6 | 3.3E-1 | 9.5E-1 | |  | GOTERM\_CC\_5 | proton-transporting two-sector ATPase complex, proton-transporting domain | **RT** |  | 5 | 4.0E-1 | 9.6E-1 | |  | GOTERM\_CC\_5 | vacuolar proton-transporting V-type ATPase complex | **RT** |  | 5 | 4.0E-1 | 9.6E-1 | |  | GOTERM\_BP\_5 | ATP hydrolysis coupled proton transport | **RT** |  | 6 | 4.6E-1 | 9.8E-1 | |  | GOTERM\_BP\_5 | cation transmembrane transport | **RT** |  | 17 | 5.2E-1 | 9.9E-1 | |  | GOTERM\_BP\_5 | hydrogen transport | **RT** |  | 9 | 6.1E-1 | 9.9E-1 | |  | GOTERM\_BP\_5 | proton transport | **RT** |  | 9 | 6.1E-1 | 9.9E-1 | |  | GOTERM\_BP\_5 | ion transmembrane transport | **RT** |  | 17 | 6.8E-1 | 1.0E0 | |  | GOTERM\_BP\_5 | inorganic ion transmembrane transport | **RT** |  | 15 | 7.0E-1 | 1.0E0 | |  | GOTERM\_MF\_5 | cation transmembrane transporter activity | **RT** |  | 30 | 8.1E-1 | 1.0E0 | |  | GOTERM\_BP\_5 | cation transport | **RT** |  | 28 | 8.1E-1 | 1.0E0 | |  | GOTERM\_CC\_5 | proton-transporting two-sector ATPase complex, catalytic domain | **RT** |  | 3 | 9.0E-1 | 1.0E0 | |  | GOTERM\_BP\_5 | ion transport | **RT** |  | 36 | 9.2E-1 | 1.0E0 | |  | GOTERM\_BP\_5 | transmembrane transport | **RT** |  | 36 | 9.5E-1 | 1.0E0 | | Annotation Cluster 100 | | Enrichment Score: 0.38 |  |  | Count | P\_Value | Benjamini | | --- | --- | --- | --- | --- | --- | --- | --- | |  | GOTERM\_CC\_5 | clathrin vesicle coat | **RT** |  | 5 | 1.2E-1 | 9.1E-1 | |  | GOTERM\_CC\_5 | clathrin-coated vesicle membrane | **RT** |  | 5 | 2.0E-1 | 9.5E-1 | |  | GOTERM\_CC\_5 | clathrin coat | **RT** |  | 5 | 2.5E-1 | 9.6E-1 | |  | GOTERM\_CC\_5 | trans-Golgi network transport vesicle | **RT** |  | 4 | 2.6E-1 | 9.6E-1 | |  | GOTERM\_CC\_5 | trans-Golgi network transport vesicle membrane | **RT** |  | 3 | 2.6E-1 | 9.5E-1 | |  | GOTERM\_CC\_5 | clathrin coat of trans-Golgi network vesicle | **RT** |  | 3 | 2.6E-1 | 9.5E-1 | |  | GOTERM\_BP\_5 | synaptic vesicle budding from presynaptic endocytic zone membrane | **RT** |  | 5 | 3.2E-1 | 9.5E-1 | |  | GOTERM\_CC\_5 | vesicle coat | **RT** |  | 7 | 3.4E-1 | 9.5E-1 | |  | GOTERM\_CC\_5 | transport vesicle membrane | **RT** |  | 7 | 3.4E-1 | 9.5E-1 | |  | GOTERM\_CC\_5 | clathrin coat of coated pit | **RT** |  | 3 | 3.4E-1 | 9.5E-1 | |  | GOTERM\_BP\_5 | synaptic vesicle budding | **RT** |  | 5 | 3.7E-1 | 9.6E-1 | |  | GOTERM\_CC\_5 | cytoplasmic vesicle membrane | **RT** |  | 11 | 3.8E-1 | 9.6E-1 | |  | GOTERM\_CC\_5 | trans-Golgi network | **RT** |  | 6 | 4.6E-1 | 9.6E-1 | |  | GOTERM\_CC\_5 | coated vesicle membrane | **RT** |  | 7 | 4.6E-1 | 9.6E-1 | |  | GOTERM\_CC\_5 | cytoplasmic vesicle part | **RT** |  | 11 | 4.7E-1 | 9.6E-1 | |  | GOTERM\_BP\_5 | vesicle coating | **RT** |  | 4 | 5.2E-1 | 9.9E-1 | |  | GOTERM\_CC\_5 | membrane coat | **RT** |  | 7 | 5.4E-1 | 9.8E-1 | |  | GOTERM\_CC\_5 | Golgi-associated vesicle membrane | **RT** |  | 4 | 5.5E-1 | 9.8E-1 | |  | GOTERM\_CC\_5 | cytoplasmic, membrane-bounded vesicle | **RT** |  | 25 | 5.7E-1 | 9.8E-1 | |  | GOTERM\_CC\_5 | exocytic vesicle | **RT** |  | 13 | 5.7E-1 | 9.8E-1 | |  | GOTERM\_CC\_5 | Golgi-associated vesicle | **RT** |  | 5 | 5.9E-1 | 9.8E-1 | |  | GOTERM\_CC\_5 | transport vesicle | **RT** |  | 16 | 6.1E-1 | 9.8E-1 | |  | GOTERM\_CC\_5 | secretory vesicle | **RT** |  | 14 | 6.2E-1 | 9.8E-1 | |  | GOTERM\_CC\_5 | cytoplasmic vesicle | **RT** |  | 26 | 6.2E-1 | 9.8E-1 | |  | GOTERM\_CC\_5 | intracellular vesicle | **RT** |  | 26 | 6.2E-1 | 9.8E-1 | |  | GOTERM\_CC\_5 | synaptic vesicle | **RT** |  | 12 | 6.3E-1 | 9.8E-1 | |  | GOTERM\_CC\_5 | coated vesicle | **RT** |  | 9 | 6.5E-1 | 9.8E-1 | |  | GOTERM\_BP\_5 | membrane budding | **RT** |  | 5 | 6.7E-1 | 1.0E0 | | Annotation Cluster 101 | | Enrichment Score: 0.37 |  |  | Count | P\_Value | Benjamini | | --- | --- | --- | --- | --- | --- | --- | --- | |  | GOTERM\_BP\_5 | protein localization to membrane | **RT** |  | 15 | 2.1E-1 | 8.9E-1 | |  | GOTERM\_BP\_5 | plasma membrane organization | **RT** |  | 9 | 4.5E-1 | 9.8E-1 | |  | GOTERM\_BP\_5 | protein localization to cell periphery | **RT** |  | 7 | 5.3E-1 | 9.9E-1 | |  | GOTERM\_BP\_5 | protein localization to plasma membrane | **RT** |  | 6 | 6.2E-1 | 9.9E-1 | | Annotation Cluster 102 | | Enrichment Score: 0.37 |  |  | Count | P\_Value | Benjamini | | --- | --- | --- | --- | --- | --- | --- | --- | |  | GOTERM\_BP\_5 | regulation of striated muscle cell differentiation | **RT** |  | 4 | 1.9E-1 | 8.7E-1 | |  | GOTERM\_BP\_5 | regulation of syncytium formation by plasma membrane fusion | **RT** |  | 3 | 3.3E-1 | 9.5E-1 | |  | GOTERM\_BP\_5 | regulation of myotube differentiation | **RT** |  | 3 | 3.3E-1 | 9.5E-1 | |  | GOTERM\_BP\_5 | regulation of myoblast fusion | **RT** |  | 3 | 3.3E-1 | 9.5E-1 | |  | GOTERM\_BP\_5 | syncytium formation by plasma membrane fusion | **RT** |  | 7 | 6.6E-1 | 1.0E0 | |  | GOTERM\_BP\_5 | syncytium formation | **RT** |  | 7 | 6.6E-1 | 1.0E0 | |  | GOTERM\_BP\_5 | myoblast fusion | **RT** |  | 4 | 9.4E-1 | 1.0E0 | | Annotation Cluster 103 | | Enrichment Score: 0.36 |  |  | Count | P\_Value | Benjamini | | --- | --- | --- | --- | --- | --- | --- | --- | |  | GOTERM\_BP\_5 | proximal/distal pattern formation | **RT** |  | 4 | 3.6E-1 | 9.6E-1 | |  | GOTERM\_BP\_5 | leg disc pattern formation | **RT** |  | 3 | 4.8E-1 | 9.8E-1 | |  | GOTERM\_BP\_5 | proximal/distal pattern formation, imaginal disc | **RT** |  | 3 | 4.8E-1 | 9.8E-1 | | Annotation Cluster 104 | | Enrichment Score: 0.36 |  |  | Count | P\_Value | Benjamini | | --- | --- | --- | --- | --- | --- | --- | --- | |  | GOTERM\_BP\_5 | protein localization to membrane | **RT** |  | 15 | 2.1E-1 | 8.9E-1 | |  | GOTERM\_BP\_5 | protein targeting to membrane | **RT** |  | 6 | 4.2E-1 | 9.7E-1 | |  | GOTERM\_BP\_5 | establishment of protein localization to endoplasmic reticulum | **RT** |  | 3 | 6.0E-1 | 9.9E-1 | |  | GOTERM\_BP\_5 | establishment of protein localization to membrane | **RT** |  | 8 | 6.8E-1 | 1.0E0 | | Annotation Cluster 105 | | Enrichment Score: 0.34 |  |  | Count | P\_Value | Benjamini | | --- | --- | --- | --- | --- | --- | --- | --- | |  | GOTERM\_BP\_5 | negative regulation of transmembrane receptor protein serine/threonine kinase signaling pathway | **RT** |  | 6 | 2.2E-1 | 9.0E-1 | |  | GOTERM\_BP\_5 | negative regulation of synaptic growth at neuromuscular junction | **RT** |  | 10 | 2.7E-1 | 9.3E-1 | |  | GOTERM\_BP\_5 | negative regulation of neuromuscular junction development | **RT** |  | 10 | 2.7E-1 | 9.3E-1 | |  | GOTERM\_BP\_5 | negative regulation of synapse assembly | **RT** |  | 10 | 2.7E-1 | 9.3E-1 | |  | GOTERM\_BP\_5 | negative regulation of BMP signaling pathway | **RT** |  | 4 | 5.2E-1 | 9.9E-1 | |  | GOTERM\_BP\_5 | regulation of transmembrane receptor protein serine/threonine kinase signaling pathway | **RT** |  | 7 | 6.3E-1 | 1.0E0 | |  | GOTERM\_BP\_5 | BMP signaling pathway | **RT** |  | 5 | 6.7E-1 | 1.0E0 | |  | GOTERM\_BP\_5 | regulation of BMP signaling pathway | **RT** |  | 5 | 7.1E-1 | 1.0E0 | |  | GOTERM\_BP\_5 | response to BMP | **RT** |  | 5 | 7.1E-1 | 1.0E0 | |  | GOTERM\_BP\_5 | cellular response to growth factor stimulus | **RT** |  | 8 | 8.5E-1 | 1.0E0 | | Annotation Cluster 106 | | Enrichment Score: 0.33 |  |  | Count | P\_Value | Benjamini | | --- | --- | --- | --- | --- | --- | --- | --- | |  | GOTERM\_CC\_5 | precatalytic spliceosome | **RT** |  | 23 | 3.2E-1 | 9.5E-1 | |  | GOTERM\_BP\_5 | mRNA metabolic process | **RT** |  | 44 | 3.5E-1 | 9.6E-1 | |  | GOTERM\_MF\_5 | poly(A) RNA binding | **RT** |  | 36 | 4.4E-1 | 9.9E-1 | |  | GOTERM\_CC\_5 | spliceosomal complex | **RT** |  | 25 | 4.8E-1 | 9.6E-1 | |  | GOTERM\_BP\_5 | RNA processing | **RT** |  | 51 | 6.2E-1 | 1.0E0 | |  | GOTERM\_CC\_5 | catalytic step 2 spliceosome | **RT** |  | 16 | 6.9E-1 | 9.9E-1 | | Annotation Cluster 107 | | Enrichment Score: 0.33 |  |  | Count | P\_Value | Benjamini | | --- | --- | --- | --- | --- | --- | --- | --- | |  | GOTERM\_BP\_5 | ketone biosynthetic process | **RT** |  | 5 | 1.1E-1 | 7.8E-1 | |  | GOTERM\_BP\_5 | ecdysteroid biosynthetic process | **RT** |  | 3 | 4.0E-1 | 9.7E-1 | |  | GOTERM\_BP\_5 | steroid biosynthetic process | **RT** |  | 3 | 6.6E-1 | 1.0E0 | |  | GOTERM\_BP\_5 | ecdysteroid metabolic process | **RT** |  | 3 | 6.6E-1 | 1.0E0 | |  | GOTERM\_BP\_5 | hormone biosynthetic process | **RT** |  | 3 | 7.0E-1 | 1.0E0 | |  | GOTERM\_BP\_5 | cellular hormone metabolic process | **RT** |  | 3 | 7.8E-1 | 1.0E0 | | Annotation Cluster 108 | | Enrichment Score: 0.32 |  |  | Count | P\_Value | Benjamini | | --- | --- | --- | --- | --- | --- | --- | --- | |  | GOTERM\_BP\_5 | positive regulation of endocytosis | **RT** |  | 7 | 2.0E-1 | 8.9E-1 | |  | GOTERM\_BP\_5 | positive regulation of receptor-mediated endocytosis | **RT** |  | 3 | 6.6E-1 | 1.0E0 | |  | GOTERM\_BP\_5 | regulation of receptor-mediated endocytosis | **RT** |  | 3 | 8.2E-1 | 1.0E0 | | Annotation Cluster 109 | | Enrichment Score: 0.32 |  |  | Count | P\_Value | Benjamini | | --- | --- | --- | --- | --- | --- | --- | --- | |  | GOTERM\_BP\_5 | nucleobase-containing compound transport | **RT** |  | 17 | 2.3E-1 | 9.0E-1 | |  | GOTERM\_BP\_5 | RNA transport | **RT** |  | 13 | 4.2E-1 | 9.7E-1 | |  | GOTERM\_BP\_5 | mRNA transport | **RT** |  | 7 | 5.0E-1 | 9.8E-1 | |  | GOTERM\_BP\_5 | RNA export from nucleus | **RT** |  | 6 | 5.8E-1 | 9.9E-1 | |  | GOTERM\_BP\_5 | mRNA-containing ribonucleoprotein complex export from nucleus | **RT** |  | 4 | 6.6E-1 | 1.0E0 | |  | GOTERM\_BP\_5 | mRNA export from nucleus | **RT** |  | 4 | 6.6E-1 | 1.0E0 | | Annotation Cluster 110 | | Enrichment Score: 0.31 |  |  | Count | P\_Value | Benjamini | | --- | --- | --- | --- | --- | --- | --- | --- | |  | GOTERM\_BP\_5 | phospholipid biosynthetic process | **RT** |  | 11 | 3.1E-2 | 5.7E-1 | |  | GOTERM\_BP\_5 | glycerolipid biosynthetic process | **RT** |  | 6 | 4.6E-1 | 9.8E-1 | |  | GOTERM\_BP\_5 | glycerophospholipid metabolic process | **RT** |  | 9 | 6.6E-1 | 1.0E0 | |  | GOTERM\_BP\_5 | lipoprotein biosynthetic process | **RT** |  | 6 | 6.8E-1 | 1.0E0 | |  | GOTERM\_BP\_5 | glycerophospholipid biosynthetic process | **RT** |  | 4 | 7.0E-1 | 1.0E0 | |  | GOTERM\_BP\_5 | membrane lipid biosynthetic process | **RT** |  | 5 | 7.1E-1 | 1.0E0 | |  | GOTERM\_BP\_5 | membrane lipid metabolic process | **RT** |  | 6 | 7.7E-1 | 1.0E0 | |  | GOTERM\_BP\_5 | glycolipid metabolic process | **RT** |  | 3 | 8.4E-1 | 1.0E0 | |  | GOTERM\_BP\_5 | glycolipid biosynthetic process | **RT** |  | 3 | 8.4E-1 | 1.0E0 | | Annotation Cluster 111 | | Enrichment Score: 0.31 |  |  | Count | P\_Value | Benjamini | | --- | --- | --- | --- | --- | --- | --- | --- | |  | GOTERM\_BP\_5 | organic acid catabolic process | **RT** |  | 9 | 1.3E-1 | 8.1E-1 | |  | GOTERM\_BP\_5 | alpha-amino acid catabolic process | **RT** |  | 6 | 1.5E-1 | 8.3E-1 | |  | GOTERM\_BP\_5 | carboxylic acid catabolic process | **RT** |  | 7 | 2.7E-1 | 9.3E-1 | |  | GOTERM\_BP\_5 | glutamine family amino acid metabolic process | **RT** |  | 5 | 3.2E-1 | 9.5E-1 | |  | GOTERM\_BP\_5 | fatty acid oxidation | **RT** |  | 3 | 5.4E-1 | 9.9E-1 | |  | GOTERM\_BP\_5 | fatty acid catabolic process | **RT** |  | 3 | 5.4E-1 | 9.9E-1 | |  | GOTERM\_BP\_5 | lipid oxidation | **RT** |  | 3 | 5.4E-1 | 9.9E-1 | |  | GOTERM\_BP\_5 | alpha-amino acid metabolic process | **RT** |  | 8 | 5.6E-1 | 9.9E-1 | |  | GOTERM\_BP\_5 | cellular lipid catabolic process | **RT** |  | 4 | 5.7E-1 | 9.9E-1 | |  | GOTERM\_BP\_5 | cellular amino acid catabolic process | **RT** |  | 3 | 6.6E-1 | 1.0E0 | |  | GOTERM\_BP\_5 | fatty acid metabolic process | **RT** |  | 6 | 7.1E-1 | 1.0E0 | |  | GOTERM\_BP\_5 | lipid modification | **RT** |  | 7 | 8.1E-1 | 1.0E0 | |  | GOTERM\_BP\_5 | alpha-amino acid biosynthetic process | **RT** |  | 3 | 8.4E-1 | 1.0E0 | |  | GOTERM\_BP\_5 | carboxylic acid biosynthetic process | **RT** |  | 4 | 9.6E-1 | 1.0E0 | |  | GOTERM\_BP\_5 | organic acid biosynthetic process | **RT** |  | 4 | 9.8E-1 | 1.0E0 | | Annotation Cluster 112 | | Enrichment Score: 0.29 |  |  | Count | P\_Value | Benjamini | | --- | --- | --- | --- | --- | --- | --- | --- | |  | GOTERM\_CC\_5 | condensed nuclear chromosome | **RT** |  | 4 | 3.8E-1 | 9.6E-1 | |  | GOTERM\_BP\_5 | mitotic sister chromatid cohesion | **RT** |  | 3 | 4.8E-1 | 9.8E-1 | |  | GOTERM\_CC\_5 | cohesin complex | **RT** |  | 3 | 5.0E-1 | 9.7E-1 | |  | GOTERM\_BP\_5 | sister chromatid cohesion | **RT** |  | 4 | 5.2E-1 | 9.9E-1 | |  | GOTERM\_CC\_5 | condensed chromosome | **RT** |  | 6 | 7.4E-1 | 9.9E-1 | | Annotation Cluster 113 | | Enrichment Score: 0.29 |  |  | Count | P\_Value | Benjamini | | --- | --- | --- | --- | --- | --- | --- | --- | |  | GOTERM\_CC\_5 | I band | **RT** |  | 6 | 4.1E-1 | 9.6E-1 | |  | GOTERM\_CC\_5 | Z disc | **RT** |  | 6 | 4.1E-1 | 9.6E-1 | |  | GOTERM\_CC\_5 | M band | **RT** |  | 3 | 4.2E-1 | 9.6E-1 | |  | GOTERM\_CC\_5 | myofibril | **RT** |  | 7 | 5.4E-1 | 9.8E-1 | |  | GOTERM\_CC\_5 | contractile fiber part | **RT** |  | 7 | 5.4E-1 | 9.8E-1 | |  | GOTERM\_CC\_5 | contractile fiber | **RT** |  | 7 | 5.7E-1 | 9.8E-1 | |  | GOTERM\_CC\_5 | A band | **RT** |  | 3 | 6.2E-1 | 9.8E-1 | |  | GOTERM\_CC\_5 | sarcomere | **RT** |  | 6 | 6.5E-1 | 9.8E-1 | | Annotation Cluster 114 | | Enrichment Score: 0.28 |  |  | Count | P\_Value | Benjamini | | --- | --- | --- | --- | --- | --- | --- | --- | |  | GOTERM\_MF\_5 | double-stranded DNA binding | **RT** |  | 16 | 4.8E-1 | 9.9E-1 | |  | GOTERM\_MF\_5 | sequence-specific DNA binding | **RT** |  | 33 | 4.9E-1 | 9.9E-1 | |  | GOTERM\_MF\_5 | regulatory region DNA binding | **RT** |  | 19 | 6.1E-1 | 1.0E0 | | Annotation Cluster 115 | | Enrichment Score: 0.28 |  |  | Count | P\_Value | Benjamini | | --- | --- | --- | --- | --- | --- | --- | --- | |  | GOTERM\_CC\_5 | pre-autophagosomal structure | **RT** |  | 5 | 3.0E-1 | 9.6E-1 | |  | GOTERM\_BP\_5 | autophagosome assembly | **RT** |  | 4 | 7.0E-1 | 1.0E0 | |  | GOTERM\_BP\_5 | autophagosome organization | **RT** |  | 4 | 7.0E-1 | 1.0E0 | | Annotation Cluster 116 | | Enrichment Score: 0.27 |  |  | Count | P\_Value | Benjamini | | --- | --- | --- | --- | --- | --- | --- | --- | |  | GOTERM\_MF\_5 | cysteine-type peptidase activity | **RT** |  | 15 | 2.4E-1 | 9.9E-1 | |  | GOTERM\_BP\_5 | protein modification by small protein removal | **RT** |  | 9 | 7.3E-1 | 1.0E0 | |  | GOTERM\_MF\_5 | thiol-dependent ubiquitin-specific protease activity | **RT** |  | 5 | 8.6E-1 | 1.0E0 | | Annotation Cluster 117 | | Enrichment Score: 0.27 |  |  | Count | P\_Value | Benjamini | | --- | --- | --- | --- | --- | --- | --- | --- | |  | GOTERM\_CC\_5 | transport vesicle membrane | **RT** |  | 7 | 3.4E-1 | 9.5E-1 | |  | GOTERM\_CC\_5 | exocytic vesicle membrane | **RT** |  | 3 | 6.8E-1 | 9.9E-1 | |  | GOTERM\_CC\_5 | synaptic vesicle membrane | **RT** |  | 3 | 6.8E-1 | 9.9E-1 | | Annotation Cluster 118 | | Enrichment Score: 0.27 |  |  | Count | P\_Value | Benjamini | | --- | --- | --- | --- | --- | --- | --- | --- | |  | GOTERM\_BP\_5 | actomyosin structure organization | **RT** |  | 9 | 3.9E-1 | 9.7E-1 | |  | GOTERM\_BP\_5 | striated muscle cell development | **RT** |  | 7 | 4.6E-1 | 9.8E-1 | |  | GOTERM\_BP\_5 | striated muscle cell differentiation | **RT** |  | 13 | 5.0E-1 | 9.8E-1 | |  | GOTERM\_BP\_5 | muscle cell development | **RT** |  | 7 | 5.0E-1 | 9.8E-1 | |  | GOTERM\_BP\_5 | muscle cell differentiation | **RT** |  | 14 | 5.2E-1 | 9.9E-1 | |  | GOTERM\_CC\_5 | myofibril | **RT** |  | 7 | 5.4E-1 | 9.8E-1 | |  | GOTERM\_CC\_5 | contractile fiber part | **RT** |  | 7 | 5.4E-1 | 9.8E-1 | |  | GOTERM\_BP\_5 | myofibril assembly | **RT** |  | 5 | 5.6E-1 | 9.9E-1 | |  | GOTERM\_CC\_5 | contractile fiber | **RT** |  | 7 | 5.7E-1 | 9.8E-1 | |  | GOTERM\_BP\_5 | sarcomere organization | **RT** |  | 4 | 6.2E-1 | 1.0E0 | |  | GOTERM\_BP\_5 | skeletal muscle organ development | **RT** |  | 5 | 8.9E-1 | 1.0E0 | | Annotation Cluster 119 | | Enrichment Score: 0.26 |  |  | Count | P\_Value | Benjamini | | --- | --- | --- | --- | --- | --- | --- | --- | |  | GOTERM\_CC\_5 | transcription factor complex | **RT** |  | 17 | 3.8E-1 | 9.5E-1 | |  | GOTERM\_CC\_5 | RNA polymerase II transcription factor complex | **RT** |  | 10 | 6.1E-1 | 9.8E-1 | |  | GOTERM\_CC\_5 | nuclear transcription factor complex | **RT** |  | 10 | 7.2E-1 | 9.9E-1 | | Annotation Cluster 120 | | Enrichment Score: 0.26 |  |  | Count | P\_Value | Benjamini | | --- | --- | --- | --- | --- | --- | --- | --- | |  | GOTERM\_BP\_5 | hippo signaling | **RT** |  | 6 | 5.0E-1 | 9.9E-1 | |  | GOTERM\_BP\_5 | regulation of hippo signaling | **RT** |  | 5 | 5.1E-1 | 9.9E-1 | |  | GOTERM\_BP\_5 | positive regulation of hippo signaling | **RT** |  | 3 | 6.6E-1 | 1.0E0 | | Annotation Cluster 121 | | Enrichment Score: 0.25 |  |  | Count | P\_Value | Benjamini | | --- | --- | --- | --- | --- | --- | --- | --- | |  | GOTERM\_BP\_5 | exocytosis | **RT** |  | 18 | 3.1E-1 | 9.4E-1 | |  | GOTERM\_BP\_5 | synaptic vesicle recycling | **RT** |  | 12 | 3.7E-1 | 9.6E-1 | |  | GOTERM\_BP\_5 | vesicle mediated transport in synapse | **RT** |  | 21 | 3.7E-1 | 9.6E-1 | |  | GOTERM\_CC\_5 | cytoplasmic vesicle membrane | **RT** |  | 11 | 3.8E-1 | 9.6E-1 | |  | GOTERM\_BP\_5 | establishment of vesicle localization | **RT** |  | 24 | 3.9E-1 | 9.6E-1 | |  | GOTERM\_BP\_5 | establishment of synaptic vesicle localization | **RT** |  | 22 | 4.5E-1 | 9.8E-1 | |  | GOTERM\_BP\_5 | synaptic vesicle transport | **RT** |  | 22 | 4.5E-1 | 9.8E-1 | |  | GOTERM\_CC\_5 | cytoplasmic vesicle part | **RT** |  | 11 | 4.7E-1 | 9.6E-1 | |  | GOTERM\_BP\_5 | synaptic vesicle localization | **RT** |  | 22 | 5.0E-1 | 9.9E-1 | |  | GOTERM\_CC\_5 | cytoplasmic, membrane-bounded vesicle | **RT** |  | 25 | 5.7E-1 | 9.8E-1 | |  | GOTERM\_BP\_5 | secretion | **RT** |  | 34 | 5.7E-1 | 9.9E-1 | |  | GOTERM\_CC\_5 | exocytic vesicle | **RT** |  | 13 | 5.7E-1 | 9.8E-1 | |  | GOTERM\_BP\_5 | regulated exocytosis | **RT** |  | 11 | 5.9E-1 | 9.9E-1 | |  | GOTERM\_CC\_5 | transport vesicle | **RT** |  | 16 | 6.1E-1 | 9.8E-1 | |  | GOTERM\_CC\_5 | secretory vesicle | **RT** |  | 14 | 6.2E-1 | 9.8E-1 | |  | GOTERM\_CC\_5 | cytoplasmic vesicle | **RT** |  | 26 | 6.2E-1 | 9.8E-1 | |  | GOTERM\_CC\_5 | intracellular vesicle | **RT** |  | 26 | 6.2E-1 | 9.8E-1 | |  | GOTERM\_CC\_5 | synaptic vesicle | **RT** |  | 12 | 6.3E-1 | 9.8E-1 | |  | GOTERM\_BP\_5 | secretion by cell | **RT** |  | 32 | 6.6E-1 | 1.0E0 | |  | GOTERM\_BP\_5 | synaptic vesicle exocytosis | **RT** |  | 10 | 6.7E-1 | 1.0E0 | |  | GOTERM\_BP\_5 | signal release from synapse | **RT** |  | 18 | 6.8E-1 | 1.0E0 | |  | GOTERM\_BP\_5 | neurotransmitter secretion | **RT** |  | 18 | 6.8E-1 | 1.0E0 | |  | GOTERM\_BP\_5 | neurotransmitter transport | **RT** |  | 19 | 7.9E-1 | 1.0E0 | |  | GOTERM\_BP\_5 | signal release | **RT** |  | 20 | 8.3E-1 | 1.0E0 | |  | GOTERM\_BP\_5 | synaptic signaling | **RT** |  | 39 | 8.4E-1 | 1.0E0 | |  | GOTERM\_BP\_5 | trans-synaptic signaling | **RT** |  | 39 | 8.4E-1 | 1.0E0 | | Annotation Cluster 122 | | Enrichment Score: 0.25 |  |  | Count | P\_Value | Benjamini | | --- | --- | --- | --- | --- | --- | --- | --- | |  | GOTERM\_BP\_5 | courtship behavior | **RT** |  | 14 | 3.2E-1 | 9.5E-1 | |  | GOTERM\_BP\_5 | mating | **RT** |  | 15 | 6.0E-1 | 9.9E-1 | |  | GOTERM\_BP\_5 | male courtship behavior | **RT** |  | 10 | 6.2E-1 | 1.0E0 | |  | GOTERM\_BP\_5 | mating behavior | **RT** |  | 14 | 6.7E-1 | 1.0E0 | |  | GOTERM\_BP\_5 | male mating behavior | **RT** |  | 10 | 7.2E-1 | 1.0E0 | | Annotation Cluster 123 | | Enrichment Score: 0.25 |  |  | Count | P\_Value | Benjamini | | --- | --- | --- | --- | --- | --- | --- | --- | |  | GOTERM\_BP\_5 | ribosome assembly | **RT** |  | 8 | 3.2E-1 | 9.5E-1 | |  | GOTERM\_BP\_5 | ribosomal large subunit assembly | **RT** |  | 3 | 6.6E-1 | 1.0E0 | |  | GOTERM\_BP\_5 | ribosomal large subunit biogenesis | **RT** |  | 5 | 8.8E-1 | 1.0E0 | | Annotation Cluster 124 | | Enrichment Score: 0.24 |  |  | Count | P\_Value | Benjamini | | --- | --- | --- | --- | --- | --- | --- | --- | |  | GOTERM\_BP\_5 | establishment of mitotic spindle localization | **RT** |  | 5 | 4.7E-1 | 9.8E-1 | |  | GOTERM\_BP\_5 | establishment of mitotic spindle orientation | **RT** |  | 4 | 4.7E-1 | 9.8E-1 | |  | GOTERM\_BP\_5 | establishment of spindle localization | **RT** |  | 5 | 6.4E-1 | 1.0E0 | |  | GOTERM\_BP\_5 | spindle localization | **RT** |  | 5 | 6.4E-1 | 1.0E0 | |  | GOTERM\_BP\_5 | establishment of spindle orientation | **RT** |  | 4 | 6.6E-1 | 1.0E0 | | Annotation Cluster 125 | | Enrichment Score: 0.24 |  |  | Count | P\_Value | Benjamini | | --- | --- | --- | --- | --- | --- | --- | --- | |  | GOTERM\_BP\_5 | genital disc development | **RT** |  | 6 | 3.8E-1 | 9.6E-1 | |  | GOTERM\_BP\_5 | genital disc morphogenesis | **RT** |  | 4 | 4.2E-1 | 9.7E-1 | |  | GOTERM\_BP\_5 | imaginal disc-derived male genitalia development | **RT** |  | 4 | 5.2E-1 | 9.9E-1 | |  | GOTERM\_BP\_5 | imaginal disc-derived genitalia development | **RT** |  | 4 | 5.7E-1 | 9.9E-1 | |  | GOTERM\_BP\_5 | male genitalia morphogenesis | **RT** |  | 3 | 6.0E-1 | 9.9E-1 | |  | GOTERM\_BP\_5 | imaginal disc-derived male genitalia morphogenesis | **RT** |  | 3 | 6.0E-1 | 9.9E-1 | |  | GOTERM\_BP\_5 | male anatomical structure morphogenesis | **RT** |  | 3 | 6.0E-1 | 9.9E-1 | |  | GOTERM\_BP\_5 | male genitalia development | **RT** |  | 3 | 6.0E-1 | 9.9E-1 | |  | GOTERM\_BP\_5 | genitalia development | **RT** |  | 4 | 6.2E-1 | 1.0E0 | |  | GOTERM\_BP\_5 | imaginal disc-derived genitalia morphogenesis | **RT** |  | 3 | 6.6E-1 | 1.0E0 | |  | GOTERM\_BP\_5 | genitalia morphogenesis | **RT** |  | 3 | 6.6E-1 | 1.0E0 | |  | GOTERM\_BP\_5 | post-embryonic genitalia morphogenesis | **RT** |  | 3 | 6.6E-1 | 1.0E0 | |  | GOTERM\_BP\_5 | male sex differentiation | **RT** |  | 3 | 7.0E-1 | 1.0E0 | | Annotation Cluster 126 | | Enrichment Score: 0.24 |  |  | Count | P\_Value | Benjamini | | --- | --- | --- | --- | --- | --- | --- | --- | |  | GOTERM\_BP\_5 | organophosphate biosynthetic process | **RT** |  | 28 | 6.9E-2 | 7.0E-1 | |  | GOTERM\_BP\_5 | nucleoside triphosphate biosynthetic process | **RT** |  | 6 | 5.4E-1 | 9.9E-1 | |  | GOTERM\_BP\_5 | nucleoside monophosphate biosynthetic process | **RT** |  | 6 | 6.2E-1 | 9.9E-1 | |  | GOTERM\_BP\_5 | nucleoside phosphate biosynthetic process | **RT** |  | 13 | 7.6E-1 | 1.0E0 | |  | GOTERM\_BP\_5 | nucleoside biosynthetic process | **RT** |  | 6 | 8.3E-1 | 1.0E0 | |  | GOTERM\_BP\_5 | glycosyl compound biosynthetic process | **RT** |  | 6 | 8.3E-1 | 1.0E0 | |  | GOTERM\_BP\_5 | nucleotide biosynthetic process | **RT** |  | 12 | 8.3E-1 | 1.0E0 | |  | GOTERM\_BP\_5 | ribose phosphate biosynthetic process | **RT** |  | 10 | 8.6E-1 | 1.0E0 | |  | GOTERM\_BP\_5 | purine-containing compound biosynthetic process | **RT** |  | 10 | 8.6E-1 | 1.0E0 | | Annotation Cluster 127 | | Enrichment Score: 0.23 |  |  | Count | P\_Value | Benjamini | | --- | --- | --- | --- | --- | --- | --- | --- | |  | GOTERM\_BP\_5 | axo-dendritic transport | **RT** |  | 7 | 4.6E-1 | 9.8E-1 | |  | GOTERM\_BP\_5 | cytoskeleton-dependent intracellular transport | **RT** |  | 8 | 5.6E-1 | 9.9E-1 | |  | GOTERM\_BP\_5 | establishment of localization by movement along microtubule | **RT** |  | 7 | 7.7E-1 | 1.0E0 | | Annotation Cluster 128 | | Enrichment Score: 0.23 |  |  | Count | P\_Value | Benjamini | | --- | --- | --- | --- | --- | --- | --- | --- | |  | GOTERM\_BP\_5 | regulation of chromosome organization | **RT** |  | 19 | 1.5E-1 | 8.3E-1 | |  | GOTERM\_BP\_5 | regulation of chromatin silencing | **RT** |  | 9 | 2.1E-1 | 8.9E-1 | |  | GOTERM\_BP\_5 | negative regulation of macromolecule metabolic process | **RT** |  | 68 | 5.0E-1 | 9.8E-1 | |  | GOTERM\_BP\_5 | regulation of gene silencing | **RT** |  | 11 | 5.1E-1 | 9.9E-1 | |  | GOTERM\_BP\_5 | negative regulation of gene expression | **RT** |  | 53 | 5.3E-1 | 9.9E-1 | |  | GOTERM\_BP\_5 | gene silencing | **RT** |  | 23 | 5.5E-1 | 9.9E-1 | |  | GOTERM\_BP\_5 | negative regulation of gene expression, epigenetic | **RT** |  | 12 | 5.8E-1 | 9.9E-1 | |  | GOTERM\_BP\_5 | chromatin silencing | **RT** |  | 12 | 5.8E-1 | 9.9E-1 | |  | GOTERM\_BP\_5 | negative regulation of RNA metabolic process | **RT** |  | 33 | 7.2E-1 | 1.0E0 | |  | GOTERM\_BP\_5 | regulation of gene expression, epigenetic | **RT** |  | 19 | 7.5E-1 | 1.0E0 | |  | GOTERM\_BP\_5 | negative regulation of nucleobase-containing compound metabolic process | **RT** |  | 33 | 8.3E-1 | 1.0E0 | |  | GOTERM\_BP\_5 | negative regulation of cellular macromolecule biosynthetic process | **RT** |  | 38 | 8.4E-1 | 1.0E0 | |  | GOTERM\_BP\_5 | negative regulation of macromolecule biosynthetic process | **RT** |  | 38 | 8.4E-1 | 1.0E0 | |  | GOTERM\_BP\_5 | negative regulation of cellular biosynthetic process | **RT** |  | 39 | 8.7E-1 | 1.0E0 | |  | GOTERM\_BP\_5 | negative regulation of transcription, DNA-templated | **RT** |  | 27 | 9.1E-1 | 1.0E0 | |  | GOTERM\_BP\_5 | negative regulation of RNA biosynthetic process | **RT** |  | 27 | 9.1E-1 | 1.0E0 | | Annotation Cluster 129 | | Enrichment Score: 0.23 |  |  | Count | P\_Value | Benjamini | | --- | --- | --- | --- | --- | --- | --- | --- | |  | GOTERM\_CC\_5 | pole plasm | **RT** |  | 7 | 2.7E-1 | 9.5E-1 | |  | GOTERM\_CC\_5 | ribonucleoprotein granule | **RT** |  | 9 | 5.9E-1 | 9.8E-1 | |  | GOTERM\_CC\_5 | P granule | **RT** |  | 4 | 6.0E-1 | 9.8E-1 | |  | GOTERM\_CC\_5 | germ plasm | **RT** |  | 4 | 6.9E-1 | 9.9E-1 | |  | GOTERM\_CC\_5 | cytoplasmic ribonucleoprotein granule | **RT** |  | 8 | 7.1E-1 | 9.9E-1 | |  | GOTERM\_CC\_5 | cytoplasmic mRNA processing body | **RT** |  | 4 | 8.7E-1 | 1.0E0 | | Annotation Cluster 130 | | Enrichment Score: 0.23 |  |  | Count | P\_Value | Benjamini | | --- | --- | --- | --- | --- | --- | --- | --- | |  | GOTERM\_BP\_5 | actomyosin contractile ring assembly | **RT** |  | 3 | 4.8E-1 | 9.8E-1 | |  | GOTERM\_BP\_5 | assembly of actomyosin apparatus involved in cytokinesis | **RT** |  | 3 | 5.4E-1 | 9.9E-1 | |  | GOTERM\_BP\_5 | cortical actin cytoskeleton organization | **RT** |  | 6 | 5.4E-1 | 9.9E-1 | |  | GOTERM\_BP\_5 | cytokinetic process | **RT** |  | 4 | 7.0E-1 | 1.0E0 | |  | GOTERM\_BP\_5 | cortical cytoskeleton organization | **RT** |  | 6 | 7.1E-1 | 1.0E0 | | Annotation Cluster 131 | | Enrichment Score: 0.23 |  |  | Count | P\_Value | Benjamini | | --- | --- | --- | --- | --- | --- | --- | --- | |  | GOTERM\_BP\_5 | regulation of actin cytoskeleton organization | **RT** |  | 19 | 4.7E-2 | 6.4E-1 | |  | GOTERM\_BP\_5 | positive regulation of cytoskeleton organization | **RT** |  | 7 | 4.6E-1 | 9.8E-1 | |  | GOTERM\_BP\_5 | regulation of actin filament polymerization | **RT** |  | 8 | 5.6E-1 | 9.9E-1 | |  | GOTERM\_BP\_5 | actin filament polymerization | **RT** |  | 7 | 6.3E-1 | 1.0E0 | |  | GOTERM\_BP\_5 | regulation of actin filament length | **RT** |  | 8 | 6.5E-1 | 1.0E0 | |  | GOTERM\_BP\_5 | regulation of protein polymerization | **RT** |  | 8 | 6.5E-1 | 1.0E0 | |  | GOTERM\_BP\_5 | regulation of actin polymerization or depolymerization | **RT** |  | 8 | 6.5E-1 | 1.0E0 | |  | GOTERM\_BP\_5 | negative regulation of protein polymerization | **RT** |  | 3 | 7.0E-1 | 1.0E0 | |  | GOTERM\_BP\_5 | positive regulation of actin filament polymerization | **RT** |  | 4 | 7.4E-1 | 1.0E0 | |  | GOTERM\_BP\_5 | negative regulation of protein complex assembly | **RT** |  | 3 | 7.5E-1 | 1.0E0 | |  | GOTERM\_BP\_5 | negative regulation of cytoskeleton organization | **RT** |  | 4 | 8.3E-1 | 1.0E0 | |  | GOTERM\_BP\_5 | positive regulation of protein polymerization | **RT** |  | 4 | 8.3E-1 | 1.0E0 | |  | GOTERM\_BP\_5 | cellular protein complex assembly | **RT** |  | 22 | 8.8E-1 | 1.0E0 | |  | GOTERM\_BP\_5 | negative regulation of organelle organization | **RT** |  | 8 | 8.9E-1 | 1.0E0 | |  | GOTERM\_BP\_5 | positive regulation of protein complex assembly | **RT** |  | 4 | 9.7E-1 | 1.0E0 | | Annotation Cluster 132 | | Enrichment Score: 0.22 |  |  | Count | P\_Value | Benjamini | | --- | --- | --- | --- | --- | --- | --- | --- | |  | GOTERM\_CC\_5 | prespliceosome | **RT** |  | 4 | 4.4E-1 | 9.6E-1 | |  | GOTERM\_CC\_5 | U2-type spliceosomal complex | **RT** |  | 4 | 6.0E-1 | 9.8E-1 | |  | GOTERM\_BP\_5 | spliceosomal complex assembly | **RT** |  | 4 | 8.0E-1 | 1.0E0 | | Annotation Cluster 133 | | Enrichment Score: 0.2 |  |  | Count | P\_Value | Benjamini | | --- | --- | --- | --- | --- | --- | --- | --- | |  | GOTERM\_BP\_5 | negative regulation of cellular protein metabolic process | **RT** |  | 27 | 4.5E-1 | 9.8E-1 | |  | GOTERM\_BP\_5 | negative regulation of protein metabolic process | **RT** |  | 27 | 4.7E-1 | 9.8E-1 | |  | GOTERM\_BP\_5 | negative regulation of cellular amide metabolic process | **RT** |  | 12 | 6.3E-1 | 1.0E0 | |  | GOTERM\_BP\_5 | negative regulation of translation | **RT** |  | 11 | 6.6E-1 | 1.0E0 | |  | GOTERM\_BP\_5 | regulation of translation | **RT** |  | 19 | 7.9E-1 | 1.0E0 | |  | GOTERM\_BP\_5 | posttranscriptional regulation of gene expression | **RT** |  | 20 | 8.5E-1 | 1.0E0 | | Annotation Cluster 134 | | Enrichment Score: 0.2 |  |  | Count | P\_Value | Benjamini | | --- | --- | --- | --- | --- | --- | --- | --- | |  | GOTERM\_CC\_5 | PRC1 complex | **RT** |  | 3 | 5.6E-1 | 9.8E-1 | |  | GOTERM\_CC\_5 | nuclear ubiquitin ligase complex | **RT** |  | 4 | 6.5E-1 | 9.8E-1 | |  | GOTERM\_CC\_5 | PcG protein complex | **RT** |  | 5 | 7.0E-1 | 9.9E-1 | | Annotation Cluster 135 | | Enrichment Score: 0.19 |  |  | Count | P\_Value | Benjamini | | --- | --- | --- | --- | --- | --- | --- | --- | |  | GOTERM\_BP\_5 | DNA conformation change | **RT** |  | 15 | 2.1E-1 | 8.9E-1 | |  | GOTERM\_BP\_5 | chromatin assembly | **RT** |  | 5 | 7.7E-1 | 1.0E0 | |  | GOTERM\_BP\_5 | nucleosome assembly | **RT** |  | 3 | 7.8E-1 | 1.0E0 | |  | GOTERM\_BP\_5 | chromatin assembly or disassembly | **RT** |  | 5 | 7.9E-1 | 1.0E0 | |  | GOTERM\_BP\_5 | nucleosome organization | **RT** |  | 5 | 8.2E-1 | 1.0E0 | |  | GOTERM\_BP\_5 | protein-DNA complex assembly | **RT** |  | 6 | 8.5E-1 | 1.0E0 | | Annotation Cluster 136 | | Enrichment Score: 0.16 |  |  | Count | P\_Value | Benjamini | | --- | --- | --- | --- | --- | --- | --- | --- | |  | GOTERM\_BP\_5 | positive regulation of cell cycle | **RT** |  | 6 | 5.8E-1 | 9.9E-1 | |  | GOTERM\_BP\_5 | positive regulation of mitotic cell cycle | **RT** |  | 4 | 7.4E-1 | 1.0E0 | |  | GOTERM\_BP\_5 | positive regulation of cell cycle process | **RT** |  | 3 | 7.8E-1 | 1.0E0 | | Annotation Cluster 137 | | Enrichment Score: 0.15 |  |  | Count | P\_Value | Benjamini | | --- | --- | --- | --- | --- | --- | --- | --- | |  | GOTERM\_BP\_5 | ribosome assembly | **RT** |  | 8 | 3.2E-1 | 9.5E-1 | |  | GOTERM\_CC\_5 | 90S preribosome | **RT** |  | 3 | 5.6E-1 | 9.8E-1 | |  | GOTERM\_BP\_5 | ribosomal small subunit biogenesis | **RT** |  | 7 | 6.3E-1 | 1.0E0 | |  | GOTERM\_BP\_5 | ribosomal small subunit assembly | **RT** |  | 4 | 6.6E-1 | 1.0E0 | |  | GOTERM\_CC\_5 | preribosome | **RT** |  | 5 | 7.4E-1 | 9.9E-1 | |  | GOTERM\_BP\_5 | maturation of SSU-rRNA | **RT** |  | 4 | 7.4E-1 | 1.0E0 | |  | GOTERM\_CC\_5 | small ribosomal subunit | **RT** |  | 10 | 8.7E-1 | 1.0E0 | |  | GOTERM\_BP\_5 | rRNA processing | **RT** |  | 8 | 8.7E-1 | 1.0E0 | |  | GOTERM\_CC\_5 | cytosolic small ribosomal subunit | **RT** |  | 6 | 9.1E-1 | 1.0E0 | |  | GOTERM\_BP\_5 | ncRNA metabolic process | **RT** |  | 14 | 9.9E-1 | 1.0E0 | | Annotation Cluster 138 | | Enrichment Score: 0.15 |  |  | Count | P\_Value | Benjamini | | --- | --- | --- | --- | --- | --- | --- | --- | |  | GOTERM\_MF\_5 | protein transmembrane transporter activity | **RT** |  | 6 | 4.6E-1 | 9.9E-1 | |  | GOTERM\_BP\_5 | protein transmembrane transport | **RT** |  | 6 | 6.2E-1 | 9.9E-1 | |  | GOTERM\_BP\_5 | protein import into mitochondrial matrix | **RT** |  | 4 | 6.2E-1 | 1.0E0 | |  | GOTERM\_BP\_5 | establishment of protein localization to mitochondrion | **RT** |  | 6 | 7.4E-1 | 1.0E0 | |  | GOTERM\_BP\_5 | protein targeting to mitochondrion | **RT** |  | 6 | 7.4E-1 | 1.0E0 | |  | GOTERM\_MF\_5 | P-P-bond-hydrolysis-driven protein transmembrane transporter activity | **RT** |  | 4 | 7.7E-1 | 1.0E0 | |  | GOTERM\_BP\_5 | intracellular protein transmembrane transport | **RT** |  | 5 | 7.7E-1 | 1.0E0 | |  | GOTERM\_BP\_5 | mitochondrial transmembrane transport | **RT** |  | 4 | 8.5E-1 | 1.0E0 | |  | GOTERM\_BP\_5 | intracellular protein transmembrane import | **RT** |  | 4 | 8.5E-1 | 1.0E0 | | Annotation Cluster 139 | | Enrichment Score: 0.15 |  |  | Count | P\_Value | Benjamini | | --- | --- | --- | --- | --- | --- | --- | --- | |  | GOTERM\_BP\_5 | microtubule cytoskeleton organization | **RT** |  | 37 | 5.6E-1 | 9.9E-1 | |  | GOTERM\_BP\_5 | centrosome duplication | **RT** |  | 11 | 7.5E-1 | 1.0E0 | |  | GOTERM\_BP\_5 | microtubule organizing center organization | **RT** |  | 15 | 7.5E-1 | 1.0E0 | |  | GOTERM\_BP\_5 | centrosome organization | **RT** |  | 15 | 7.5E-1 | 1.0E0 | |  | GOTERM\_BP\_5 | centrosome cycle | **RT** |  | 13 | 7.6E-1 | 1.0E0 | | Annotation Cluster 140 | | Enrichment Score: 0.14 |  |  | Count | P\_Value | Benjamini | | --- | --- | --- | --- | --- | --- | --- | --- | |  | GOTERM\_CC\_5 | axon | **RT** |  | 25 | 6.2E-1 | 9.8E-1 | |  | GOTERM\_CC\_5 | terminal bouton | **RT** |  | 9 | 7.0E-1 | 9.9E-1 | |  | GOTERM\_CC\_5 | presynaptic membrane | **RT** |  | 3 | 7.2E-1 | 9.9E-1 | |  | GOTERM\_CC\_5 | axon part | **RT** |  | 13 | 7.3E-1 | 9.9E-1 | |  | GOTERM\_CC\_5 | axon terminus | **RT** |  | 9 | 7.7E-1 | 9.9E-1 | |  | GOTERM\_CC\_5 | neuron projection terminus | **RT** |  | 9 | 7.7E-1 | 9.9E-1 | | Annotation Cluster 141 | | Enrichment Score: 0.14 |  |  | Count | P\_Value | Benjamini | | --- | --- | --- | --- | --- | --- | --- | --- | |  | GOTERM\_BP\_5 | establishment or maintenance of cytoskeleton polarity | **RT** |  | 9 | 4.2E-1 | 9.7E-1 | |  | GOTERM\_BP\_5 | establishment or maintenance of microtubule cytoskeleton polarity | **RT** |  | 4 | 9.4E-1 | 1.0E0 | |  | GOTERM\_BP\_5 | oocyte microtubule cytoskeleton organization | **RT** |  | 3 | 9.5E-1 | 1.0E0 | | Annotation Cluster 142 | | Enrichment Score: 0.14 |  |  | Count | P\_Value | Benjamini | | --- | --- | --- | --- | --- | --- | --- | --- | |  | GOTERM\_BP\_5 | regulation of innate immune response | **RT** |  | 11 | 6.1E-1 | 9.9E-1 | |  | GOTERM\_BP\_5 | defense response to bacterium | **RT** |  | 16 | 6.1E-1 | 9.9E-1 | |  | GOTERM\_BP\_5 | response to bacterium | **RT** |  | 17 | 6.5E-1 | 1.0E0 | |  | GOTERM\_BP\_5 | defense response to Gram-negative bacterium | **RT** |  | 9 | 7.1E-1 | 1.0E0 | |  | GOTERM\_BP\_5 | defense response to other organism | **RT** |  | 18 | 8.3E-1 | 1.0E0 | |  | GOTERM\_BP\_5 | positive regulation of defense response | **RT** |  | 7 | 8.3E-1 | 1.0E0 | |  | GOTERM\_BP\_5 | positive regulation of innate immune response | **RT** |  | 5 | 8.9E-1 | 1.0E0 | | Annotation Cluster 143 | | Enrichment Score: 0.13 |  |  | Count | P\_Value | Benjamini | | --- | --- | --- | --- | --- | --- | --- | --- | |  | GOTERM\_BP\_5 | regulation of sister chromatid segregation | **RT** |  | 5 | 5.6E-1 | 9.9E-1 | |  | GOTERM\_BP\_5 | regulation of mitotic nuclear division | **RT** |  | 6 | 6.2E-1 | 9.9E-1 | |  | GOTERM\_BP\_5 | regulation of mitotic sister chromatid segregation | **RT** |  | 4 | 6.6E-1 | 1.0E0 | |  | GOTERM\_BP\_5 | regulation of nuclear division | **RT** |  | 6 | 6.8E-1 | 1.0E0 | |  | GOTERM\_BP\_5 | metaphase/anaphase transition of mitotic cell cycle | **RT** |  | 3 | 8.2E-1 | 1.0E0 | |  | GOTERM\_BP\_5 | regulation of metaphase/anaphase transition of cell cycle | **RT** |  | 3 | 8.2E-1 | 1.0E0 | |  | GOTERM\_BP\_5 | chromosome separation | **RT** |  | 4 | 8.3E-1 | 1.0E0 | |  | GOTERM\_BP\_5 | metaphase/anaphase transition of cell cycle | **RT** |  | 3 | 8.4E-1 | 1.0E0 | |  | GOTERM\_BP\_5 | mitotic sister chromatid separation | **RT** |  | 3 | 8.7E-1 | 1.0E0 | | Annotation Cluster 144 | | Enrichment Score: 0.13 |  |  | Count | P\_Value | Benjamini | | --- | --- | --- | --- | --- | --- | --- | --- | |  | GOTERM\_MF\_5 | N-methyltransferase activity | **RT** |  | 5 | 5.5E-1 | 1.0E0 | |  | GOTERM\_BP\_5 | histone methylation | **RT** |  | 7 | 7.2E-1 | 1.0E0 | |  | GOTERM\_MF\_5 | protein methyltransferase activity | **RT** |  | 5 | 7.4E-1 | 1.0E0 | |  | GOTERM\_BP\_5 | peptidyl-lysine methylation | **RT** |  | 6 | 7.7E-1 | 1.0E0 | |  | GOTERM\_BP\_5 | macromolecule methylation | **RT** |  | 9 | 8.1E-1 | 1.0E0 | |  | GOTERM\_BP\_5 | protein methylation | **RT** |  | 7 | 8.5E-1 | 1.0E0 | |  | GOTERM\_MF\_5 | S-adenosylmethionine-dependent methyltransferase activity | **RT** |  | 5 | 8.6E-1 | 1.0E0 | | Annotation Cluster 145 | | Enrichment Score: 0.12 |  |  | Count | P\_Value | Benjamini | | --- | --- | --- | --- | --- | --- | --- | --- | |  | GOTERM\_BP\_5 | spindle assembly | **RT** |  | 6 | 7.1E-1 | 1.0E0 | |  | GOTERM\_BP\_5 | mitotic spindle assembly | **RT** |  | 3 | 7.8E-1 | 1.0E0 | |  | GOTERM\_BP\_5 | microtubule cytoskeleton organization involved in mitosis | **RT** |  | 3 | 7.8E-1 | 1.0E0 | | Annotation Cluster 146 | | Enrichment Score: 0.12 |  |  | Count | P\_Value | Benjamini | | --- | --- | --- | --- | --- | --- | --- | --- | |  | GOTERM\_CC\_5 | transferase complex, transferring phosphorus-containing groups | **RT** |  | 17 | 6.7E-1 | 9.9E-1 | |  | GOTERM\_CC\_5 | DNA-directed RNA polymerase II, holoenzyme | **RT** |  | 10 | 7.8E-1 | 9.9E-1 | |  | GOTERM\_CC\_5 | DNA-directed RNA polymerase complex | **RT** |  | 11 | 7.9E-1 | 9.9E-1 | |  | GOTERM\_CC\_5 | RNA polymerase complex | **RT** |  | 11 | 7.9E-1 | 9.9E-1 | |  | GOTERM\_CC\_5 | nuclear DNA-directed RNA polymerase complex | **RT** |  | 11 | 7.9E-1 | 9.9E-1 | | Annotation Cluster 147 | | Enrichment Score: 0.12 |  |  | Count | P\_Value | Benjamini | | --- | --- | --- | --- | --- | --- | --- | --- | |  | GOTERM\_CC\_5 | mitochondrial matrix | **RT** |  | 22 | 4.6E-1 | 9.6E-1 | |  | GOTERM\_CC\_5 | organellar ribosome | **RT** |  | 11 | 7.3E-1 | 9.9E-1 | |  | GOTERM\_CC\_5 | mitochondrial ribosome | **RT** |  | 11 | 7.3E-1 | 9.9E-1 | |  | GOTERM\_CC\_5 | organellar large ribosomal subunit | **RT** |  | 6 | 8.2E-1 | 1.0E0 | |  | GOTERM\_CC\_5 | mitochondrial large ribosomal subunit | **RT** |  | 6 | 8.2E-1 | 1.0E0 | |  | GOTERM\_BP\_5 | mitochondrial translation | **RT** |  | 10 | 8.8E-1 | 1.0E0 | |  | GOTERM\_CC\_5 | organellar small ribosomal subunit | **RT** |  | 4 | 8.9E-1 | 1.0E0 | |  | GOTERM\_CC\_5 | mitochondrial small ribosomal subunit | **RT** |  | 4 | 8.9E-1 | 1.0E0 | | Annotation Cluster 148 | | Enrichment Score: 0.11 |  |  | Count | P\_Value | Benjamini | | --- | --- | --- | --- | --- | --- | --- | --- | |  | GOTERM\_BP\_5 | negative regulation of neuron differentiation | **RT** |  | 6 | 5.4E-1 | 9.9E-1 | |  | GOTERM\_BP\_5 | negative regulation of neuron projection development | **RT** |  | 3 | 9.1E-1 | 1.0E0 | |  | GOTERM\_BP\_5 | negative regulation of cell projection organization | **RT** |  | 3 | 9.4E-1 | 1.0E0 | | Annotation Cluster 149 | | Enrichment Score: 0.11 |  |  | Count | P\_Value | Benjamini | | --- | --- | --- | --- | --- | --- | --- | --- | |  | GOTERM\_BP\_5 | organ formation | **RT** |  | 7 | 5.7E-1 | 9.9E-1 | |  | GOTERM\_BP\_5 | formation of anatomical boundary | **RT** |  | 6 | 7.9E-1 | 1.0E0 | |  | GOTERM\_BP\_5 | salivary gland boundary specification | **RT** |  | 3 | 8.7E-1 | 1.0E0 | |  | GOTERM\_BP\_5 | formation of organ boundary | **RT** |  | 4 | 9.3E-1 | 1.0E0 | | Annotation Cluster 150 | | Enrichment Score: 0.11 |  |  | Count | P\_Value | Benjamini | | --- | --- | --- | --- | --- | --- | --- | --- | |  | GOTERM\_MF\_5 | ubiquitin protein ligase activity | **RT** |  | 13 | 3.7E-1 | 9.9E-1 | |  | GOTERM\_CC\_5 | ubiquitin ligase complex | **RT** |  | 19 | 5.6E-1 | 9.8E-1 | |  | GOTERM\_BP\_5 | proteolysis involved in cellular protein catabolic process | **RT** |  | 34 | 8.7E-1 | 1.0E0 | |  | GOTERM\_BP\_5 | cellular protein catabolic process | **RT** |  | 34 | 8.8E-1 | 1.0E0 | |  | GOTERM\_BP\_5 | protein catabolic process | **RT** |  | 35 | 8.8E-1 | 1.0E0 | |  | GOTERM\_BP\_5 | cellular macromolecule catabolic process | **RT** |  | 42 | 8.9E-1 | 1.0E0 | |  | GOTERM\_BP\_5 | modification-dependent macromolecule catabolic process | **RT** |  | 31 | 9.1E-1 | 1.0E0 | |  | GOTERM\_BP\_5 | proteasomal protein catabolic process | **RT** |  | 18 | 9.3E-1 | 1.0E0 | |  | GOTERM\_BP\_5 | proteolysis | **RT** |  | 54 | 9.8E-1 | 1.0E0 | | Annotation Cluster 151 | | Enrichment Score: 0.11 |  |  | Count | P\_Value | Benjamini | | --- | --- | --- | --- | --- | --- | --- | --- | |  | GOTERM\_BP\_5 | cellular response to hormone stimulus | **RT** |  | 15 | 3.2E-1 | 9.5E-1 | |  | GOTERM\_BP\_5 | cellular response to peptide | **RT** |  | 5 | 8.8E-1 | 1.0E0 | |  | GOTERM\_BP\_5 | cellular response to peptide hormone stimulus | **RT** |  | 5 | 8.8E-1 | 1.0E0 | |  | GOTERM\_BP\_5 | response to peptide | **RT** |  | 5 | 9.0E-1 | 1.0E0 | |  | GOTERM\_BP\_5 | response to peptide hormone | **RT** |  | 5 | 9.0E-1 | 1.0E0 | |  | GOTERM\_BP\_5 | cellular response to organonitrogen compound | **RT** |  | 6 | 9.1E-1 | 1.0E0 | |  | GOTERM\_BP\_5 | response to insulin | **RT** |  | 4 | 9.6E-1 | 1.0E0 | | Annotation Cluster 152 | | Enrichment Score: 0.11 |  |  | Count | P\_Value | Benjamini | | --- | --- | --- | --- | --- | --- | --- | --- | |  | GOTERM\_CC\_5 | intrinsic component of mitochondrial membrane | **RT** |  | 3 | 7.7E-1 | 9.9E-1 | |  | GOTERM\_CC\_5 | integral component of mitochondrial membrane | **RT** |  | 3 | 7.7E-1 | 9.9E-1 | |  | GOTERM\_CC\_5 | integral component of organelle membrane | **RT** |  | 6 | 8.2E-1 | 1.0E0 | | Annotation Cluster 153 | | Enrichment Score: 0.11 |  |  | Count | P\_Value | Benjamini | | --- | --- | --- | --- | --- | --- | --- | --- | |  | GOTERM\_CC\_5 | trans-Golgi network | **RT** |  | 6 | 4.6E-1 | 9.6E-1 | |  | GOTERM\_CC\_5 | Golgi apparatus part | **RT** |  | 23 | 9.3E-1 | 1.0E0 | |  | GOTERM\_CC\_5 | organelle subcompartment | **RT** |  | 10 | 9.3E-1 | 1.0E0 | |  | GOTERM\_CC\_5 | Golgi subcompartment | **RT** |  | 9 | 9.5E-1 | 1.0E0 | | Annotation Cluster 154 | | Enrichment Score: 0.09 |  |  | Count | P\_Value | Benjamini | | --- | --- | --- | --- | --- | --- | --- | --- | |  | GOTERM\_BP\_5 | nuclear migration | **RT** |  | 4 | 7.0E-1 | 1.0E0 | |  | GOTERM\_BP\_5 | establishment of nucleus localization | **RT** |  | 4 | 8.5E-1 | 1.0E0 | |  | GOTERM\_BP\_5 | ovarian nurse cell to oocyte transport | **RT** |  | 4 | 8.9E-1 | 1.0E0 | | Annotation Cluster 155 | | Enrichment Score: 0.09 |  |  | Count | P\_Value | Benjamini | | --- | --- | --- | --- | --- | --- | --- | --- | |  | GOTERM\_BP\_5 | protein complex assembly | **RT** |  | 38 | 6.9E-1 | 1.0E0 | |  | GOTERM\_BP\_5 | cellular macromolecular complex assembly | **RT** |  | 45 | 8.7E-1 | 1.0E0 | |  | GOTERM\_BP\_5 | cellular protein complex assembly | **RT** |  | 22 | 8.8E-1 | 1.0E0 | | Annotation Cluster 156 | | Enrichment Score: 0.08 |  |  | Count | P\_Value | Benjamini | | --- | --- | --- | --- | --- | --- | --- | --- | |  | GOTERM\_BP\_5 | regulation of ERBB signaling pathway | **RT** |  | 8 | 7.9E-1 | 1.0E0 | |  | GOTERM\_BP\_5 | regulation of epidermal growth factor receptor signaling pathway | **RT** |  | 8 | 7.9E-1 | 1.0E0 | |  | GOTERM\_BP\_5 | negative regulation of ERBB signaling pathway | **RT** |  | 5 | 8.8E-1 | 1.0E0 | |  | GOTERM\_BP\_5 | negative regulation of epidermal growth factor receptor signaling pathway | **RT** |  | 5 | 8.8E-1 | 1.0E0 | | Annotation Cluster 157 | | Enrichment Score: 0.08 |  |  | Count | P\_Value | Benjamini | | --- | --- | --- | --- | --- | --- | --- | --- | |  | GOTERM\_BP\_5 | positive regulation of nucleotide biosynthetic process | **RT** |  | 3 | 7.5E-1 | 1.0E0 | |  | GOTERM\_BP\_5 | positive regulation of nucleotide metabolic process | **RT** |  | 3 | 7.8E-1 | 1.0E0 | |  | GOTERM\_BP\_5 | regulation of nucleotide biosynthetic process | **RT** |  | 3 | 8.9E-1 | 1.0E0 | |  | GOTERM\_BP\_5 | regulation of nucleotide metabolic process | **RT** |  | 3 | 9.5E-1 | 1.0E0 | | Annotation Cluster 158 | | Enrichment Score: 0.07 |  |  | Count | P\_Value | Benjamini | | --- | --- | --- | --- | --- | --- | --- | --- | |  | GOTERM\_BP\_5 | rhodopsin mediated signaling pathway | **RT** |  | 3 | 7.5E-1 | 1.0E0 | |  | GOTERM\_BP\_5 | sensory perception of light stimulus | **RT** |  | 6 | 7.7E-1 | 1.0E0 | |  | GOTERM\_BP\_5 | phototransduction, visible light | **RT** |  | 3 | 7.8E-1 | 1.0E0 | |  | GOTERM\_BP\_5 | cellular response to light stimulus | **RT** |  | 4 | 8.5E-1 | 1.0E0 | |  | GOTERM\_BP\_5 | detection of visible light | **RT** |  | 3 | 9.2E-1 | 1.0E0 | |  | GOTERM\_BP\_5 | phototransduction | **RT** |  | 5 | 9.5E-1 | 1.0E0 | |  | GOTERM\_BP\_5 | detection of light stimulus | **RT** |  | 5 | 9.8E-1 | 1.0E0 | | Annotation Cluster 159 | | Enrichment Score: 0.06 |  |  | Count | P\_Value | Benjamini | | --- | --- | --- | --- | --- | --- | --- | --- | |  | GOTERM\_BP\_5 | regulation of cell cycle process | **RT** |  | 22 | 5.0E-1 | 9.9E-1 | |  | GOTERM\_BP\_5 | regulation of mitotic cell cycle | **RT** |  | 20 | 7.4E-1 | 1.0E0 | |  | GOTERM\_BP\_5 | mitotic cell cycle phase transition | **RT** |  | 17 | 7.7E-1 | 1.0E0 | |  | GOTERM\_BP\_5 | regulation of mitotic cell cycle phase transition | **RT** |  | 15 | 7.9E-1 | 1.0E0 | |  | GOTERM\_BP\_5 | regulation of cell cycle phase transition | **RT** |  | 15 | 7.9E-1 | 1.0E0 | |  | GOTERM\_BP\_5 | cell cycle phase transition | **RT** |  | 17 | 8.0E-1 | 1.0E0 | |  | GOTERM\_BP\_5 | regulation of cell cycle G2/M phase transition | **RT** |  | 9 | 8.4E-1 | 1.0E0 | |  | GOTERM\_BP\_5 | G2/M transition of mitotic cell cycle | **RT** |  | 8 | 9.1E-1 | 1.0E0 | |  | GOTERM\_BP\_5 | cell cycle checkpoint | **RT** |  | 10 | 9.1E-1 | 1.0E0 | |  | GOTERM\_BP\_5 | G1/S transition of mitotic cell cycle | **RT** |  | 3 | 9.3E-1 | 1.0E0 | |  | GOTERM\_BP\_5 | mitotic DNA integrity checkpoint | **RT** |  | 7 | 9.4E-1 | 1.0E0 | |  | GOTERM\_BP\_5 | mitotic DNA damage checkpoint | **RT** |  | 7 | 9.4E-1 | 1.0E0 | |  | GOTERM\_BP\_5 | DNA damage checkpoint | **RT** |  | 7 | 9.5E-1 | 1.0E0 | |  | GOTERM\_BP\_5 | negative regulation of mitotic cell cycle phase transition | **RT** |  | 9 | 9.6E-1 | 1.0E0 | |  | GOTERM\_BP\_5 | negative regulation of cell cycle phase transition | **RT** |  | 9 | 9.6E-1 | 1.0E0 | |  | GOTERM\_BP\_5 | mitotic G2 DNA damage checkpoint | **RT** |  | 6 | 9.6E-1 | 1.0E0 | |  | GOTERM\_BP\_5 | mitotic cell cycle checkpoint | **RT** |  | 8 | 9.6E-1 | 1.0E0 | |  | GOTERM\_BP\_5 | negative regulation of cell cycle | **RT** |  | 12 | 9.7E-1 | 1.0E0 | |  | GOTERM\_BP\_5 | mitotic G2/M transition checkpoint | **RT** |  | 6 | 9.7E-1 | 1.0E0 | |  | GOTERM\_BP\_5 | negative regulation of cell cycle G2/M phase transition | **RT** |  | 6 | 9.7E-1 | 1.0E0 | |  | GOTERM\_BP\_5 | negative regulation of mitotic cell cycle | **RT** |  | 9 | 9.7E-1 | 1.0E0 | |  | GOTERM\_BP\_5 | negative regulation of cell cycle process | **RT** |  | 9 | 9.8E-1 | 1.0E0 | | Annotation Cluster 160 | | Enrichment Score: 0.05 |  |  | Count | P\_Value | Benjamini | | --- | --- | --- | --- | --- | --- | --- | --- | |  | GOTERM\_BP\_5 | regulation of exocytosis | **RT** |  | 4 | 7.0E-1 | 1.0E0 | |  | GOTERM\_BP\_5 | regulation of protein secretion | **RT** |  | 4 | 7.4E-1 | 1.0E0 | |  | GOTERM\_BP\_5 | regulation of secretion | **RT** |  | 8 | 9.7E-1 | 1.0E0 | |  | GOTERM\_BP\_5 | positive regulation of secretion by cell | **RT** |  | 3 | 9.8E-1 | 1.0E0 | |  | GOTERM\_BP\_5 | positive regulation of secretion | **RT** |  | 3 | 9.8E-1 | 1.0E0 | |  | GOTERM\_BP\_5 | regulation of secretion by cell | **RT** |  | 7 | 9.8E-1 | 1.0E0 | | Annotation Cluster 161 | | Enrichment Score: 0.05 |  |  | Count | P\_Value | Benjamini | | --- | --- | --- | --- | --- | --- | --- | --- | |  | GOTERM\_BP\_5 | detection of temperature stimulus involved in sensory perception | **RT** |  | 5 | 8.8E-1 | 1.0E0 | |  | GOTERM\_BP\_5 | detection of temperature stimulus involved in sensory perception of pain | **RT** |  | 5 | 8.8E-1 | 1.0E0 | |  | GOTERM\_BP\_5 | sensory perception of temperature stimulus | **RT** |  | 5 | 8.8E-1 | 1.0E0 | |  | GOTERM\_BP\_5 | detection of stimulus involved in sensory perception | **RT** |  | 6 | 9.8E-1 | 1.0E0 | | Annotation Cluster 162 | | Enrichment Score: 0.04 |  |  | Count | P\_Value | Benjamini | | --- | --- | --- | --- | --- | --- | --- | --- | |  | GOTERM\_BP\_5 | eye pigment biosynthetic process | **RT** |  | 3 | 9.1E-1 | 1.0E0 | |  | GOTERM\_BP\_5 | pigment metabolic process involved in developmental pigmentation | **RT** |  | 3 | 9.2E-1 | 1.0E0 | |  | GOTERM\_BP\_5 | eye pigment metabolic process | **RT** |  | 3 | 9.2E-1 | 1.0E0 | | Annotation Cluster 163 | | Enrichment Score: 0.03 |  |  | Count | P\_Value | Benjamini | | --- | --- | --- | --- | --- | --- | --- | --- | |  | GOTERM\_CC\_5 | Golgi apparatus | **RT** |  | 35 | 9.1E-1 | 1.0E0 | |  | GOTERM\_CC\_5 | Golgi apparatus part | **RT** |  | 23 | 9.3E-1 | 1.0E0 | |  | GOTERM\_CC\_5 | Golgi membrane | **RT** |  | 12 | 9.8E-1 | 1.0E0 | | Annotation Cluster 164 | | Enrichment Score: 0.03 |  |  | Count | P\_Value | Benjamini | | --- | --- | --- | --- | --- | --- | --- | --- | |  | GOTERM\_BP\_5 | positive regulation of developmental growth | **RT** |  | 11 | 9.3E-1 | 1.0E0 | |  | GOTERM\_BP\_5 | positive regulation of multicellular organism growth | **RT** |  | 4 | 9.4E-1 | 1.0E0 | |  | GOTERM\_BP\_5 | regulation of multicellular organism growth | **RT** |  | 5 | 9.5E-1 | 1.0E0 | | Annotation Cluster 165 | | Enrichment Score: 0.02 |  |  | Count | P\_Value | Benjamini | | --- | --- | --- | --- | --- | --- | --- | --- | |  | GOTERM\_BP\_5 | macromolecule glycosylation | **RT** |  | 8 | 9.3E-1 | 1.0E0 | |  | GOTERM\_BP\_5 | glycosylation | **RT** |  | 8 | 9.3E-1 | 1.0E0 | |  | GOTERM\_BP\_5 | glycoprotein metabolic process | **RT** |  | 8 | 9.8E-1 | 1.0E0 | |  | GOTERM\_BP\_5 | glycoprotein biosynthetic process | **RT** |  | 8 | 9.8E-1 | 1.0E0 | | Annotation Cluster 166 | | Enrichment Score: 0.02 |  |  | Count | P\_Value | Benjamini | | --- | --- | --- | --- | --- | --- | --- | --- | |  | GOTERM\_CC\_5 | endoplasmic reticulum part | **RT** |  | 22 | 9.5E-1 | 1.0E0 | |  | GOTERM\_CC\_5 | endoplasmic reticulum | **RT** |  | 37 | 9.6E-1 | 1.0E0 | |  | GOTERM\_CC\_5 | endoplasmic reticulum membrane | **RT** |  | 18 | 9.8E-1 | 1.0E0 | | Annotation Cluster 167 | | Enrichment Score: 0.01 |  |  | Count | P\_Value | Benjamini | | --- | --- | --- | --- | --- | --- | --- | --- | |  | GOTERM\_BP\_5 | protein processing | **RT** |  | 6 | 9.3E-1 | 1.0E0 | |  | GOTERM\_BP\_5 | protein maturation | **RT** |  | 6 | 9.6E-1 | 1.0E0 | |  | GOTERM\_MF\_5 | serine-type peptidase activity | **RT** |  | 4 | 9.9E-1 | 1.0E0 | |  | GOTERM\_MF\_5 | metallopeptidase activity | **RT** |  | 5 | 1.0E0 | 1.0E0 | | Annotation Cluster 168 | | Enrichment Score: 0.01 |  |  | Count | P\_Value | Benjamini | | --- | --- | --- | --- | --- | --- | --- | --- | |  | GOTERM\_BP\_5 | positive regulation of synaptic growth at neuromuscular junction | **RT** |  | 3 | 9.7E-1 | 1.0E0 | |  | GOTERM\_BP\_5 | positive regulation of neuromuscular junction development | **RT** |  | 3 | 9.7E-1 | 1.0E0 | |  | GOTERM\_BP\_5 | positive regulation of synapse assembly | **RT** |  | 3 | 9.8E-1 | 1.0E0 | | Annotation Cluster 169 | | Enrichment Score: 0.01 |  |  | Count | P\_Value | Benjamini | | --- | --- | --- | --- | --- | --- | --- | --- | |  | GOTERM\_MF\_5 | gated channel activity | **RT** |  | 9 | 9.8E-1 | 1.0E0 | |  | GOTERM\_MF\_5 | ion channel activity | **RT** |  | 12 | 9.9E-1 | 1.0E0 | |  | GOTERM\_MF\_5 | substrate-specific channel activity | **RT** |  | 12 | 9.9E-1 | 1.0E0 | |  | GOTERM\_CC\_5 | cation channel complex | **RT** |  | 3 | 9.9E-1 | 1.0E0 | |  | GOTERM\_CC\_5 | ion channel complex | **RT** |  | 5 | 1.0E0 | 1.0E0 | | Annotation Cluster 170 | | Enrichment Score: 0 |  |  | Count | P\_Value | Benjamini | | --- | --- | --- | --- | --- | --- | --- | --- | |  | GOTERM\_CC\_5 | plasma membrane part | **RT** |  | 66 | 9.8E-1 | 1.0E0 | |  | GOTERM\_CC\_5 | intrinsic component of plasma membrane | **RT** |  | 28 | 1.0E0 | 1.0E0 | |  | GOTERM\_CC\_5 | integral component of plasma membrane | **RT** |  | 26 | 1.0E0 | 1.0E0 | | Annotation Cluster 171 | | Enrichment Score: 0 |  |  | Count | P\_Value | Benjamini | | --- | --- | --- | --- | --- | --- | --- | --- | |  | GOTERM\_CC\_5 | protein acetyltransferase complex | **RT** |  | 4 | 1.0E0 | 1.0E0 | |  | GOTERM\_CC\_5 | acetyltransferase complex | **RT** |  | 4 | 1.0E0 | 1.0E0 | |  | GOTERM\_CC\_5 | histone acetyltransferase complex | **RT** |  | 3 | 1.0E0 | 1.0E0 | | Annotation Cluster 172 | | Enrichment Score: 0 |  |  | Count | P\_Value | Benjamini | | --- | --- | --- | --- | --- | --- | --- | --- | |  | GOTERM\_MF\_5 | neuropeptide receptor activity | **RT** |  | 3 | 9.9E-1 | 1.0E0 | |  | GOTERM\_MF\_5 | G-protein coupled peptide receptor activity | **RT** |  | 3 | 1.0E0 | 1.0E0 | |  | GOTERM\_MF\_5 | G-protein coupled receptor activity | **RT** |  | 4 | 1.0E0 | 1.0E0 |   were not clustered. | |  | |
