# Supplementary material for: Enabling cell-type-specific behavioral epigenetics in Drosophila: a modified high-yield INTACT method reveals the impact of social environment on the epigenetic landscape in dopaminergic neurons
Source: BMC Biol. 2019 Apr 10;17:30. doi: 10.1186/s12915-019-0646-4 (PMC6456965; doi:10.1186/s12915-019-0646-4)
Supplement: Supplementary file 10 — Gorilla and DAVID functional analysis. The zip file contains top level html files which may be opened in a browser. These will give the Gorilla functional analysis and DAVID GO analyses referred to in the main text. (ZIP 919 kb) [file 12915_2019_646_MOESM10_ESM.zip › Additional File 10/TPM83_GOLevel5.html]

DAVID: Database for Annotation, Visualization, and Integrated Discovery (Laboratory of Human Retrovirology and Immunoinformatics (LHRI); National Institute of Allergies and Infectious Diseases (NIAID); Leidos Biomedical Research, Inc. (LBR)


|  |  |  |  |  |  |
| --- | --- | --- | --- | --- | --- |
| DAVID Bioinformatics 6.7  |  |  |  |  |  | | --- | --- | --- | --- | --- | | |  |  |  |  | | --- | --- | --- | --- | |  | |  | | --- | | DAVID Bioinformatics Resources 6.8 | | Laboratory of Human Retrovirology and Immunoinformatics (LHRI) | | |   100%  **\*\*\* Welcome to DAVID 6.8 \*\*\* \*\*\* If you are looking for DAVID 6.7, please visit our development site. \*\*\*** |
| |  |  |  |  |  |  |  |  |  |  |  |  |  |  |  |  |  |  |  |  |  |  |  |  |  |  |  |  |  |  |  |  |  |  |  |  |  |  |  |  |  |  |  |  |  |  |  |  |  |  |  |  |  |  |  |  |  |  |  |  |  |  |  |  |  |  |  |  |  |  |  |  |  |  |  |  |  |  |  |  |  |  |  |  |  |  |  |  |  |  |  |  |  |  |  |  |  |  |  |  |  |  |  |  |  |  |  |  |  |  |  |  |  |  |  |  |  |  |  |  |  |  |  |  |  |  |  |  |  |  |  |  |  |  |  |  |  |  |  |  |  |  |  |  |  |  |  |  |  |  |  |  |  |  |  |  |  |  |  |  |  |  |  |  |  |  |  |  |  |  |  |  |  |  |  |  |  |  |  |  |  |  |  |  |  |  |  |  |  |  |  |  |  |  |  |  |  |  |  |  |  |  |  |  |  |  |  |  |  |  |  |  |  |  |  |  |  |  |  |  |  |  |  |  |  |  |  |  |  |  |  |  |  |  |  |  |  |  |  |  |  |  |  |  |  |  |  |  |  |  |  |  |  |  |  |  |  |  |  |  |  |  |  |  |  |  |  |  |  |  |  |  |  |  |  |  |  |  |  |  |  |  |  |  |  |  |  |  |  |  |  |  |  |  |  |  |  |  |  |  |  |  |  |  |  |  |  |  |  |  |  |  |  |  |  |  |  |  |  |  |  |  |  |  |  |  |  |  |  |  |  |  |  |  |  |  |  |  |  |  |  |  |  |  |  |  |  |  |  |  |  |  |  |  |  |  |  |  |  |  |  |  |  |  |  |  |  |  |  |  |  |  |  |  |  |  |  |  |  |  |  |  |  |  |  |  |  |  |  |  |  |  |  |  |  |  |  |  |  |  |  |  |  |  |  |  |  |  |  |  |  |  |  |  |  |  |  |  |  |  |  |  |  |  |  |  |  |  |  |  |  |  |  |  |  |  |  |  |  |  |  |  |  |  |  |  |  |  |  |  |  |  |  |  |  |  |  |  |  |  |  |  |  |  |  |  |  |  |  |  |  |  |  |  |  |  |  |  |  |  |  |  |  |  |  |  |  |  |  |  |  |  |  |  |  |  |  |  |  |  |  |  |  |  |  |  |  |  |  |  |  |  |  |  |  |  |  |  |  |  |  |  |  |  |  |  |  |  |  |  |  |  |  |  |  |  |  |  |  |  |  |  |  |  |  |  |  |  |  |  |  |  |  |  |  |  |  |  |  |  |  |  |  |  |  |  |  |  |  |  |  |  |  |  |  |  |  |  |  |  |  |  |  |  |  |  |  |  |  |  |  |  |  |  |  |  |  |  |  |  |  |  |  |  |  |  |  |  |  |  |  |  |  |  |  |  |  |  |  |  |  |  |  |  |  |  |  |  |  |  |  |  |  |  |  |  |  |  |  |  |  |  |  |  |  |  |  |  |  |  |  |  |  |  |  |  |  |  |  |  |  |  |  |  |  |  |  |  |  |  |  |  |  |  |  |  |  |  |  |  |  |  |  |  |  |  |  |  |  |  |  |  |  |  |  |  |  |  |  |  |  |  |  |  |  |  |  |  |  |  |  |  |  |  |  |  |  |  |  |  |  |  |  |  |  |  |  |  |  |  |  |  |  |  |  |  |  |  |  |  |  |  |  |  |  |  |  |  |  |  |  |  |  |  |  |  |  |  |  |  |  |  |  |  |  |  |  |  |  |  |  |  |  |  |  |  |  |  |  |  |  |  |  |  |  |  |  |  |  |  |  |  |  |  |  |  |  |  |  |  |  |  |  |  |  |  |  |  |  |  |  |  |  |  |  |  |  |  |  |  |  |  |  |  |  |  |  |  |  |  |  |  |  |  |  |  |  |  |  |  |  |  |  |  |  |  |  |  |  |  |  |  |  |  |  |  |  |  |  |  |  |  |  |  |  |  |  |  |  |  |  |  |  |  |  |  |  |  |  |  |  |  |  |  |  |  |  |  |  |  |  |  |  |  |  |  |  |  |  |  |  |  |  |  |  |  |  |  |  |  |  |  |  |  |  |  |  |  |  |  |  |  |  |  |  |  |  |  |  |  |  |  |  |  |  |  |  |  |  |  |  |  |  |  |  |  |  |  |  |  |  |  |  |  |  |  |  |  |  |  |  |  |  |  |  |  |  |  |  |  |  |  |  |  |  |  |  |  |  |  |  |  |  |  |  |  |  |  |  |  |  |  |  |  |  |  |  |  |  |  |  |  |  |  |  |  |  |  |  |  |  |  |  |  |  |  |  |  |  |  |  |  |  |  |  |  |  |  |  |  |  |  |  |  |  |  |  |  |  |  |  |  |  |  |  |  |  |  |  |  |  |  |  |  |  |  |  |  |  |  |  |  |  |  |  |  |  |  |  |  |  |  |  |  |  |  |  |  |  |  |  |  |  |  |  |  |  |  |  |  |  |  |  |  |  |  |  |  |  |  |  |  |  |  |  |  |  |  |  |  |  |  |  |  |  |  |  |  |  |  |  |  |  |  |  |  |  |  |  |  |  |  |  |  |  |  |  |  |  |  |  |  |  |  |  |  |  |  |  |  |  |  |  |  |  |  |  |  |  |  |  |  |  |  |  |  |  |  |  |  |  |  |  |  |  |  |  |  |  |  |  |  |  |  |  |  |  |  |  |  |  |  |  |  |  |  |  |  | | --- | --- | --- | --- | --- | --- | --- | --- | --- | --- | --- | --- | --- | --- | --- | --- | --- | --- | --- | --- | --- | --- | --- | --- | --- | --- | --- | --- | --- | --- | --- | --- | --- | --- | --- | --- | --- | --- | --- | --- | --- | --- | --- | --- | --- | --- | --- | --- | --- | --- | --- | --- | --- | --- | --- | --- | --- | --- | --- | --- | --- | --- | --- | --- | --- | --- | --- | --- | --- | --- | --- | --- | --- | --- | --- | --- | --- | --- | --- | --- | --- | --- | --- | --- | --- | --- | --- | --- | --- | --- | --- | --- | --- | --- | --- | --- | --- | --- | --- | --- | --- | --- | --- | --- | --- | --- | --- | --- | --- | --- | --- | --- | --- | --- | --- | --- | --- | --- | --- | --- | --- | --- | --- | --- | --- | --- | --- | --- | --- | --- | --- | --- | --- | --- | --- | --- | --- | --- | --- | --- | --- | --- | --- | --- | --- | --- | --- | --- | --- | --- | --- | --- | --- | --- | --- | --- | --- | --- | --- | --- | --- | --- | --- | --- | --- | --- | --- | --- | --- | --- | --- | --- | --- | --- | --- | --- | --- | --- | --- | --- | --- | --- | --- | --- | --- | --- | --- | --- | --- | --- | --- | --- | --- | --- | --- | --- | --- | --- | --- | --- | --- | --- | --- | --- | --- | --- | --- | --- | --- | --- | --- | --- | --- | --- | --- | --- | --- | --- | --- | --- | --- | --- | --- | --- | --- | --- | --- | --- | --- | --- | --- | --- | --- | --- | --- | --- | --- | --- | --- | --- | --- | --- | --- | --- | --- | --- | --- | --- | --- | --- | --- | --- | --- | --- | --- | --- | --- | --- | --- | --- | --- | --- | --- | --- | --- | --- | --- | --- | --- | --- | --- | --- | --- | --- | --- | --- | --- | --- | --- | --- | --- | --- | --- | --- | --- | --- | --- | --- | --- | --- | --- | --- | --- | --- | --- | --- | --- | --- | --- | --- | --- | --- | --- | --- | --- | --- | --- | --- | --- | --- | --- | --- | --- | --- | --- | --- | --- | --- | --- | --- | --- | --- | --- | --- | --- | --- | --- | --- | --- | --- | --- | --- | --- | --- | --- | --- | --- | --- | --- | --- | --- | --- | --- | --- | --- | --- | --- | --- | --- | --- | --- | --- | --- | --- | --- | --- | --- | --- | --- | --- | --- | --- | --- | --- | --- | --- | --- | --- | --- | --- | --- | --- | --- | --- | --- | --- | --- | --- | --- | --- | --- | --- | --- | --- | --- | --- | --- | --- | --- | --- | --- | --- | --- | --- | --- | --- | --- | --- | --- | --- | --- | --- | --- | --- | --- | --- | --- | --- | --- | --- | --- | --- | --- | --- | --- | --- | --- | --- | --- | --- | --- | --- | --- | --- | --- | --- | --- | --- | --- | --- | --- | --- | --- | --- | --- | --- | --- | --- | --- | --- | --- | --- | --- | --- | --- | --- | --- | --- | --- | --- | --- | --- | --- | --- | --- | --- | --- | --- | --- | --- | --- | --- | --- | --- | --- | --- | --- | --- | --- | --- | --- | --- | --- | --- | --- | --- | --- | --- | --- | --- | --- | --- | --- | --- | --- | --- | --- | --- | --- | --- | --- | --- | --- | --- | --- | --- | --- | --- | --- | --- | --- | --- | --- | --- | --- | --- | --- | --- | --- | --- | --- | --- | --- | --- | --- | --- | --- | --- | --- | --- | --- | --- | --- | --- | --- | --- | --- | --- | --- | --- | --- | --- | --- | --- | --- | --- | --- | --- | --- | --- | --- | --- | --- | --- | --- | --- | --- | --- | --- | --- | --- | --- | --- | --- | --- | --- | --- | --- | --- | --- | --- | --- | --- | --- | --- | --- | --- | --- | --- | --- | --- | --- | --- | --- | --- | --- | --- | --- | --- | --- | --- | --- | --- | --- | --- | --- | --- | --- | --- | --- | --- | --- | --- | --- | --- | --- | --- | --- | --- | --- | --- | --- | --- | --- | --- | --- | --- | --- | --- | --- | --- | --- | --- | --- | --- | --- | --- | --- | --- | --- | --- | --- | --- | --- | --- | --- | --- | --- | --- | --- | --- | --- | --- | --- | --- | --- | --- | --- | --- | --- | --- | --- | --- | --- | --- | --- | --- | --- | --- | --- | --- | --- | --- | --- | --- | --- | --- | --- | --- | --- | --- | --- | --- | --- | --- | --- | --- | --- | --- | --- | --- | --- | --- | --- | --- | --- | --- | --- | --- | --- | --- | --- | --- | --- | --- | --- | --- | --- | --- | --- | --- | --- | --- | --- | --- | --- | --- | --- | --- | --- | --- | --- | --- | --- | --- | --- | --- | --- | --- | --- | --- | --- | --- | --- | --- | --- | --- | --- | --- | --- | --- | --- | --- | --- | --- | --- | --- | --- | --- | --- | --- | --- | --- | --- | --- | --- | --- | --- | --- | --- | --- | --- | --- | --- | --- | --- | --- | --- | --- | --- | --- | --- | --- | --- | --- | --- | --- | --- | --- | --- | --- | --- | --- | --- | --- | --- | --- | --- | --- | --- | --- | --- | --- | --- | --- | --- | --- | --- | --- | --- | --- | --- | --- | --- | --- | --- | --- | --- | --- | --- | --- | --- | --- | --- | --- | --- | --- | --- | --- | --- | --- | --- | --- | --- | --- | --- | --- | --- | --- | --- | --- | --- | --- | --- | --- | --- | --- | --- | --- | --- | --- | --- | --- | --- | --- | --- | --- | --- | --- | --- | --- | --- | --- | --- | --- | --- | --- | --- | --- | --- | --- | --- | --- | --- | --- | --- | --- | --- | --- | --- | --- | --- | --- | --- | --- | --- | --- | --- | --- | --- | --- | --- | --- | --- | --- | --- | --- | --- | --- | --- | --- | --- | --- | --- | --- | --- | --- | --- | --- | --- | --- | --- | --- | --- | --- | --- | --- | --- | --- | --- | --- | --- | --- | --- | --- | --- | --- | --- | --- | --- | --- | --- | --- | --- | --- | --- | --- | --- | --- | --- | --- | --- | --- | --- | --- | --- | --- | --- | --- | --- | --- | --- | --- | --- | --- | --- | --- | --- | --- | --- | --- | --- | --- | --- | --- | --- | --- | --- | --- | --- | --- | --- | --- | --- | --- | --- | --- | --- | --- | --- | --- | --- | --- | --- | --- | --- | --- | --- | --- | --- | --- | --- | --- | --- | --- | --- | --- | --- | --- | --- | --- | --- | --- | --- | --- | --- | --- | --- | --- | --- | --- | --- | --- | --- | --- | --- | --- | --- | --- | --- | --- | --- | --- | --- | --- | --- | --- | --- | --- | --- | --- | --- | --- | --- | --- | --- | --- | --- | --- | --- | --- | --- | --- | --- | --- | --- | --- | --- | --- | --- | --- | --- | --- | --- | --- | --- | --- | --- | --- | --- | --- | --- | --- | --- | --- | --- | --- | --- | --- | --- | --- | --- | --- | --- | --- | --- | --- | --- | --- | --- | --- | --- | --- | --- | --- | --- | --- | --- | --- | --- | --- | --- | --- | --- | --- | --- | --- | --- | --- | --- | --- | --- | --- | --- | --- | --- | --- | --- | --- | --- | --- | --- | --- | --- | --- | --- | --- | --- | --- | --- | --- | --- | --- | --- | --- | --- | --- | --- | --- | --- | --- | --- | --- | --- | --- | --- | --- | --- | --- | --- | --- | --- | --- | --- | --- | --- | --- | --- | --- | --- | --- | --- | --- | --- | --- | --- | --- | --- | --- | --- | --- | --- | --- | --- | --- | --- | --- | --- | --- | --- | --- | --- | --- | --- | --- | --- | --- | --- | --- | --- | --- | --- | --- | --- | --- | --- | --- | --- | --- | --- | --- | --- | --- | --- | --- | --- | --- | --- | --- | --- | --- | --- | --- | --- | --- | --- | --- | --- | --- | --- | --- | --- | --- | --- | --- | --- | --- | --- | --- | --- | --- | --- | --- | --- | --- | --- | --- | --- | | DAVID Functional Annotation Clustering      |  | | --- | | Functional Annotation Clustering | | Help and Manual | | Current Gene List: DavidMay18\_TPM83 | | Current Background: DavidMay18\_TPM8\_bg | | 361 DAVID IDs |  - Options          Classification Stringency    Custom   Lowest   Low   Medium   High   Highest   - |  |  |  |     | --- | --- | --- |     | Kappa Similarity | Similarity Term Overlap 3 4 5 6 7 8 9 10 | Similarity Threshold 0.20 0.25 0.30 0.35 0.40 0.45 0.50 0.55 0.60 0.65 0.70 0.75 0.80 0.85 0.90 0.95 1.00 |     | Classification | Initial Group Membership 2 3 4 5 6 7 8 9 10 | Final Group Membership 2 3 4 5 6 7 8 9 10 | Multiple Linkage Threshold 0.00 0.05 0.10 0.15 0.20 0.25 0.30 0.35 0.40 0.45 0.50 0.55 0.60 0.65 0.70 0.75 0.80 0.85 0.90 0.95 1.00 |  |     | Enrichment Thresholds | EASE |  |  |  |     |     | Display | Fold Change | Bonferroni | Benjamini | FDR | LT,PH,PT |  |  |  |  | | --- | --- | --- | |  |  |  |     |  |  | | --- | --- | | 22 Cluster(s) | Download File |  | Annotation Cluster 1 | | Enrichment Score: 2.25 |  |  | Count | P\_Value | Benjamini | | --- | --- | --- | --- | --- | --- | --- | --- | |  | GOTERM\_BP\_DIRECT | mitochondrial translation | **RT** |  | 15 | 2.0E-5 | 1.3E-2 | |  | GOTERM\_CC\_DIRECT | mitochondrial small ribosomal subunit | **RT** |  | 8 | 7.5E-4 | 7.5E-2 | |  | GOTERM\_MF\_DIRECT | structural constituent of ribosome | **RT** |  | 21 | 2.6E-3 | 5.1E-1 | |  | GOTERM\_BP\_DIRECT | translation | **RT** |  | 20 | 8.6E-3 | 7.7E-1 | |  | GOTERM\_CC\_DIRECT | mitochondrial large ribosomal subunit | **RT** |  | 7 | 2.1E-2 | 5.8E-1 | |  | UP\_KEYWORDS | Ribonucleoprotein | **RT** |  | 18 | 3.1E-2 | 8.4E-1 | |  | UP\_KEYWORDS | Ribosomal protein | **RT** |  | 16 | 4.5E-2 | 8.6E-1 | |  | KEGG\_PATHWAY | Ribosome | **RT** |  | 14 | 1.1E-1 | 7.9E-1 | | Annotation Cluster 2 | | Enrichment Score: 1.77 |  |  | Count | P\_Value | Benjamini | | --- | --- | --- | --- | --- | --- | --- | --- | |  | GOTERM\_BP\_DIRECT | cellular response to amino acid stimulus | **RT** |  | 4 | 4.9E-3 | 8.1E-1 | |  | GOTERM\_CC\_DIRECT | Ragulator complex | **RT** |  | 3 | 2.5E-2 | 5.8E-1 | |  | GOTERM\_BP\_DIRECT | positive regulation of TOR signaling | **RT** |  | 4 | 4.0E-2 | 1.0E0 | | Annotation Cluster 3 | | Enrichment Score: 1.4 |  |  | Count | P\_Value | Benjamini | | --- | --- | --- | --- | --- | --- | --- | --- | |  | UP\_SEQ\_FEATURE | transit peptide:Mitochondrion | **RT** |  | 9 | 1.6E-2 | 9.8E-1 | |  | UP\_KEYWORDS | Transit peptide | **RT** |  | 10 | 6.2E-2 | 8.9E-1 | |  | UP\_KEYWORDS | Mitochondrion | **RT** |  | 15 | 6.4E-2 | 8.5E-1 | | Annotation Cluster 4 | | Enrichment Score: 1.38 |  |  | Count | P\_Value | Benjamini | | --- | --- | --- | --- | --- | --- | --- | --- | |  | GOTERM\_CC\_DIRECT | proteasome complex | **RT** |  | 8 | 3.1E-3 | 1.5E-1 | |  | KEGG\_PATHWAY | Proteasome | **RT** |  | 8 | 6.5E-3 | 3.5E-1 | |  | GOTERM\_BP\_DIRECT | proteasome-mediated ubiquitin-dependent protein catabolic process | **RT** |  | 10 | 7.2E-3 | 8.0E-1 | |  | UP\_KEYWORDS | Proteasome | **RT** |  | 7 | 9.3E-3 | 8.0E-1 | |  | GOTERM\_CC\_DIRECT | proteasome regulatory particle | **RT** |  | 4 | 8.9E-2 | 9.1E-1 | |  | GOTERM\_CC\_DIRECT | proteasome regulatory particle, base subcomplex | **RT** |  | 3 | 9.7E-2 | 9.0E-1 | |  | GOTERM\_MF\_DIRECT | endopeptidase activity | **RT** |  | 4 | 1.0E-1 | 1.0E0 | |  | GOTERM\_CC\_DIRECT | proteasome core complex | **RT** |  | 3 | 1.4E-1 | 9.3E-1 | |  | GOTERM\_MF\_DIRECT | threonine-type endopeptidase activity | **RT** |  | 3 | 1.5E-1 | 1.0E0 | |  | INTERPRO | Proteasome, subunit alpha/beta | **RT** |  | 3 | 1.6E-1 | 1.0E0 | |  | UP\_KEYWORDS | Threonine protease | **RT** |  | 3 | 1.7E-1 | 9.6E-1 | | Annotation Cluster 5 | | Enrichment Score: 1.01 |  |  | Count | P\_Value | Benjamini | | --- | --- | --- | --- | --- | --- | --- | --- | |  | SMART | Sm | **RT** |  | 3 | 3.4E-2 | 9.7E-1 | |  | INTERPRO | Ribonucleoprotein LSM domain | **RT** |  | 3 | 5.3E-2 | 1.0E0 | |  | INTERPRO | Like-Sm (LSM) domain | **RT** |  | 3 | 7.1E-2 | 1.0E0 | |  | GOTERM\_CC\_DIRECT | spliceosomal complex | **RT** |  | 4 | 2.2E-1 | 9.6E-1 | |  | GOTERM\_CC\_DIRECT | small nuclear ribonucleoprotein complex | **RT** |  | 3 | 3.2E-1 | 9.8E-1 | | Annotation Cluster 6 | | Enrichment Score: 0.9 |  |  | Count | P\_Value | Benjamini | | --- | --- | --- | --- | --- | --- | --- | --- | |  | GOTERM\_MF\_DIRECT | flavin adenine dinucleotide binding | **RT** |  | 4 | 1.2E-1 | 1.0E0 | |  | UP\_KEYWORDS | FAD | **RT** |  | 4 | 1.2E-1 | 9.5E-1 | |  | UP\_KEYWORDS | Flavoprotein | **RT** |  | 4 | 1.5E-1 | 9.5E-1 | | Annotation Cluster 7 | | Enrichment Score: 0.85 |  |  | Count | P\_Value | Benjamini | | --- | --- | --- | --- | --- | --- | --- | --- | |  | INTERPRO | TCP-1-like chaperonin intermediate domain | **RT** |  | 3 | 9.0E-2 | 1.0E0 | |  | INTERPRO | Chaperonin TCP-1, conserved site | **RT** |  | 3 | 9.0E-2 | 1.0E0 | |  | INTERPRO | Chaperone tailless complex polypeptide 1 (TCP-1) | **RT** |  | 3 | 9.0E-2 | 1.0E0 | |  | INTERPRO | GroEL-like apical domain | **RT** |  | 3 | 1.3E-1 | 1.0E0 | |  | INTERPRO | Chaperonin Cpn60/TCP-1 | **RT** |  | 3 | 1.3E-1 | 1.0E0 | |  | INTERPRO | GroEL-like equatorial domain | **RT** |  | 3 | 1.3E-1 | 1.0E0 | |  | UP\_KEYWORDS | Chaperone | **RT** |  | 5 | 1.9E-1 | 9.4E-1 | |  | GOTERM\_BP\_DIRECT | protein folding | **RT** |  | 6 | 4.3E-1 | 1.0E0 | | Annotation Cluster 8 | | Enrichment Score: 0.6 |  |  | Count | P\_Value | Benjamini | | --- | --- | --- | --- | --- | --- | --- | --- | |  | GOTERM\_MF\_DIRECT | translation initiation factor activity | **RT** |  | 5 | 1.9E-1 | 1.0E0 | |  | GOTERM\_BP\_DIRECT | regulation of translational initiation | **RT** |  | 3 | 2.1E-1 | 1.0E0 | |  | UP\_KEYWORDS | Initiation factor | **RT** |  | 4 | 2.9E-1 | 9.8E-1 | |  | UP\_KEYWORDS | Protein biosynthesis | **RT** |  | 6 | 3.4E-1 | 9.8E-1 | | Annotation Cluster 9 | | Enrichment Score: 0.59 |  |  | Count | P\_Value | Benjamini | | --- | --- | --- | --- | --- | --- | --- | --- | |  | SMART | PINT | **RT** |  | 3 | 1.2E-1 | 9.7E-1 | |  | INTERPRO | Proteasome component (PCI) domain | **RT** |  | 3 | 1.8E-1 | 1.0E0 | |  | INTERPRO | Winged helix-turn-helix DNA-binding domain | **RT** |  | 4 | 7.9E-1 | 1.0E0 | | Annotation Cluster 10 | | Enrichment Score: 0.55 |  |  | Count | P\_Value | Benjamini | | --- | --- | --- | --- | --- | --- | --- | --- | |  | SMART | AAA | **RT** |  | 6 | 5.9E-2 | 8.8E-1 | |  | INTERPRO | AAA+ ATPase domain | **RT** |  | 6 | 1.3E-1 | 1.0E0 | |  | INTERPRO | ATPase, AAA-type, conserved site | **RT** |  | 3 | 2.3E-1 | 1.0E0 | |  | INTERPRO | ATPase, AAA-type, core | **RT** |  | 3 | 3.5E-1 | 1.0E0 | |  | INTERPRO | ABC transporter, conserved site | **RT** |  | 3 | 3.8E-1 | 1.0E0 | |  | INTERPRO | ABC transporter-like | **RT** |  | 3 | 3.8E-1 | 1.0E0 | |  | GOTERM\_MF\_DIRECT | ATPase activity | **RT** |  | 6 | 4.5E-1 | 1.0E0 | |  | INTERPRO | P-loop containing nucleoside triphosphate hydrolase | **RT** |  | 12 | 9.2E-1 | 1.0E0 | | Annotation Cluster 11 | | Enrichment Score: 0.29 |  |  | Count | P\_Value | Benjamini | | --- | --- | --- | --- | --- | --- | --- | --- | |  | GOTERM\_CC\_DIRECT | spliceosomal complex | **RT** |  | 4 | 2.2E-1 | 9.6E-1 | |  | GOTERM\_BP\_DIRECT | mRNA splicing, via spliceosome | **RT** |  | 10 | 4.1E-1 | 1.0E0 | |  | KEGG\_PATHWAY | Spliceosome | **RT** |  | 7 | 4.7E-1 | 9.9E-1 | |  | GOTERM\_CC\_DIRECT | precatalytic spliceosome | **RT** |  | 8 | 5.1E-1 | 9.9E-1 | |  | UP\_KEYWORDS | mRNA splicing | **RT** |  | 3 | 5.7E-1 | 1.0E0 | |  | GOTERM\_BP\_DIRECT | regulation of alternative mRNA splicing, via spliceosome | **RT** |  | 4 | 6.8E-1 | 1.0E0 | |  | UP\_KEYWORDS | mRNA processing | **RT** |  | 3 | 7.6E-1 | 1.0E0 | |  | GOTERM\_CC\_DIRECT | catalytic step 2 spliceosome | **RT** |  | 5 | 8.1E-1 | 1.0E0 | | Annotation Cluster 12 | | Enrichment Score: 0.27 |  |  | Count | P\_Value | Benjamini | | --- | --- | --- | --- | --- | --- | --- | --- | |  | GOTERM\_MF\_DIRECT | RNA polymerase II transcription cofactor activity | **RT** |  | 4 | 1.4E-1 | 1.0E0 | |  | GOTERM\_CC\_DIRECT | mediator complex | **RT** |  | 4 | 1.5E-1 | 9.2E-1 | |  | UP\_KEYWORDS | Activator | **RT** |  | 7 | 3.7E-1 | 9.8E-1 | |  | GOTERM\_BP\_DIRECT | transcription from RNA polymerase II promoter | **RT** |  | 4 | 4.7E-1 | 1.0E0 | |  | UP\_KEYWORDS | Repressor | **RT** |  | 4 | 7.2E-1 | 1.0E0 | |  | UP\_KEYWORDS | Transcription | **RT** |  | 13 | 8.4E-1 | 1.0E0 | |  | GOTERM\_BP\_DIRECT | regulation of transcription from RNA polymerase II promoter | **RT** |  | 5 | 9.2E-1 | 1.0E0 | |  | UP\_KEYWORDS | Transcription regulation | **RT** |  | 11 | 9.2E-1 | 1.0E0 | |  | GOTERM\_BP\_DIRECT | transcription, DNA-templated | **RT** |  | 8 | 9.7E-1 | 1.0E0 | |  | GOTERM\_BP\_DIRECT | positive regulation of transcription from RNA polymerase II promoter | **RT** |  | 4 | 9.9E-1 | 1.0E0 | | Annotation Cluster 13 | | Enrichment Score: 0.16 |  |  | Count | P\_Value | Benjamini | | --- | --- | --- | --- | --- | --- | --- | --- | |  | GOTERM\_BP\_DIRECT | mitochondrial electron transport, NADH to ubiquinone | **RT** |  | 3 | 5.2E-1 | 1.0E0 | |  | UP\_KEYWORDS | Ubiquinone | **RT** |  | 3 | 6.8E-1 | 1.0E0 | |  | GOTERM\_CC\_DIRECT | mitochondrial respiratory chain complex I | **RT** |  | 3 | 6.8E-1 | 1.0E0 | |  | KEGG\_PATHWAY | Oxidative phosphorylation | **RT** |  | 4 | 9.7E-1 | 1.0E0 | | Annotation Cluster 14 | | Enrichment Score: 0.15 |  |  | Count | P\_Value | Benjamini | | --- | --- | --- | --- | --- | --- | --- | --- | |  | UP\_KEYWORDS | WD repeat | **RT** |  | 4 | 5.3E-1 | 9.9E-1 | |  | INTERPRO | WD40 repeat, conserved site | **RT** |  | 4 | 5.3E-1 | 1.0E0 | |  | SMART | WD40 | **RT** |  | 4 | 7.0E-1 | 1.0E0 | |  | INTERPRO | WD40 repeat | **RT** |  | 4 | 8.5E-1 | 1.0E0 | |  | INTERPRO | WD40/YVTN repeat-like-containing domain | **RT** |  | 5 | 8.7E-1 | 1.0E0 | |  | INTERPRO | WD40-repeat-containing domain | **RT** |  | 4 | 9.0E-1 | 1.0E0 | | Annotation Cluster 15 | | Enrichment Score: 0.13 |  |  | Count | P\_Value | Benjamini | | --- | --- | --- | --- | --- | --- | --- | --- | |  | SMART | RING | **RT** |  | 4 | 4.5E-1 | 1.0E0 | |  | GOTERM\_MF\_DIRECT | ubiquitin-protein transferase activity | **RT** |  | 7 | 6.7E-1 | 1.0E0 | |  | GOTERM\_BP\_DIRECT | protein ubiquitination | **RT** |  | 6 | 7.4E-1 | 1.0E0 | |  | INTERPRO | Zinc finger, RING-type | **RT** |  | 4 | 8.4E-1 | 1.0E0 | |  | KEGG\_PATHWAY | Ubiquitin mediated proteolysis | **RT** |  | 4 | 8.6E-1 | 1.0E0 | |  | INTERPRO | Zinc finger, RING/FYVE/PHD-type | **RT** |  | 5 | 9.7E-1 | 1.0E0 | | Annotation Cluster 16 | | Enrichment Score: 0.06 |  |  | Count | P\_Value | Benjamini | | --- | --- | --- | --- | --- | --- | --- | --- | |  | SMART | SM00868 | **RT** |  | 3 | 6.4E-1 | 1.0E0 | |  | INTERPRO | Zinc finger, AD-type | **RT** |  | 3 | 7.8E-1 | 1.0E0 | |  | SMART | ZnF\_C2H2 | **RT** |  | 5 | 9.1E-1 | 1.0E0 | |  | GOTERM\_MF\_DIRECT | nucleic acid binding | **RT** |  | 9 | 9.6E-1 | 1.0E0 | |  | INTERPRO | Zinc finger, C2H2-like | **RT** |  | 5 | 9.7E-1 | 1.0E0 | |  | INTERPRO | Zinc finger C2H2-type/integrase DNA-binding domain | **RT** |  | 4 | 9.7E-1 | 1.0E0 | |  | INTERPRO | Zinc finger, C2H2 | **RT** |  | 5 | 9.9E-1 | 1.0E0 | | Annotation Cluster 17 | | Enrichment Score: 0.05 |  |  | Count | P\_Value | Benjamini | | --- | --- | --- | --- | --- | --- | --- | --- | |  | GOTERM\_MF\_DIRECT | ATP binding | **RT** |  | 21 | 8.4E-1 | 1.0E0 | |  | UP\_KEYWORDS | ATP-binding | **RT** |  | 17 | 8.5E-1 | 1.0E0 | |  | UP\_KEYWORDS | Nucleotide-binding | **RT** |  | 18 | 9.8E-1 | 1.0E0 | | Annotation Cluster 18 | | Enrichment Score: 0.03 |  |  | Count | P\_Value | Benjamini | | --- | --- | --- | --- | --- | --- | --- | --- | |  | UP\_SEQ\_FEATURE | transmembrane region | **RT** |  | 13 | 8.6E-1 | 1.0E0 | |  | UP\_SEQ\_FEATURE | glycosylation site:N-linked (GlcNAc...) | **RT** |  | 8 | 9.2E-1 | 1.0E0 | |  | UP\_KEYWORDS | Glycoprotein | **RT** |  | 8 | 9.9E-1 | 1.0E0 | | Annotation Cluster 19 | | Enrichment Score: 0.03 |  |  | Count | P\_Value | Benjamini | | --- | --- | --- | --- | --- | --- | --- | --- | |  | UP\_KEYWORDS | Transmembrane helix | **RT** |  | 74 | 9.1E-1 | 1.0E0 | |  | UP\_KEYWORDS | Transmembrane | **RT** |  | 74 | 9.2E-1 | 1.0E0 | |  | UP\_KEYWORDS | Membrane | **RT** |  | 80 | 9.7E-1 | 1.0E0 | |  | GOTERM\_CC\_DIRECT | integral component of membrane | **RT** |  | 67 | 9.7E-1 | 1.0E0 | | Annotation Cluster 20 | | Enrichment Score: 0.02 |  |  | Count | P\_Value | Benjamini | | --- | --- | --- | --- | --- | --- | --- | --- | |  | SMART | S\_TKc | **RT** |  | 4 | 9.0E-1 | 1.0E0 | |  | GOTERM\_MF\_DIRECT | protein serine/threonine kinase activity | **RT** |  | 4 | 9.3E-1 | 1.0E0 | |  | INTERPRO | Serine/threonine-protein kinase, active site | **RT** |  | 4 | 9.3E-1 | 1.0E0 | |  | INTERPRO | Protein kinase-like domain | **RT** |  | 7 | 9.4E-1 | 1.0E0 | |  | INTERPRO | Protein kinase, ATP binding site | **RT** |  | 4 | 9.6E-1 | 1.0E0 | |  | UP\_KEYWORDS | Kinase | **RT** |  | 7 | 9.6E-1 | 1.0E0 | |  | INTERPRO | Protein kinase, catalytic domain | **RT** |  | 5 | 9.8E-1 | 1.0E0 | |  | GOTERM\_MF\_DIRECT | protein kinase activity | **RT** |  | 3 | 9.9E-1 | 1.0E0 | |  | GOTERM\_BP\_DIRECT | protein phosphorylation | **RT** |  | 5 | 9.9E-1 | 1.0E0 | | Annotation Cluster 21 | | Enrichment Score: 0.02 |  |  | Count | P\_Value | Benjamini | | --- | --- | --- | --- | --- | --- | --- | --- | |  | INTERPRO | Nucleotide-binding, alpha-beta plait | **RT** |  | 5 | 9.3E-1 | 1.0E0 | |  | INTERPRO | RNA recognition motif domain | **RT** |  | 4 | 9.5E-1 | 1.0E0 | |  | SMART | RRM | **RT** |  | 3 | 9.5E-1 | 1.0E0 | |  | GOTERM\_MF\_DIRECT | nucleotide binding | **RT** |  | 5 | 9.7E-1 | 1.0E0 | |  | GOTERM\_MF\_DIRECT | mRNA binding | **RT** |  | 4 | 9.9E-1 | 1.0E0 | | Annotation Cluster 22 | | Enrichment Score: 0.02 |  |  | Count | P\_Value | Benjamini | | --- | --- | --- | --- | --- | --- | --- | --- | |  | UP\_KEYWORDS | Zinc | **RT** |  | 15 | 9.6E-1 | 1.0E0 | |  | UP\_KEYWORDS | Zinc-finger | **RT** |  | 10 | 9.6E-1 | 1.0E0 | |  | UP\_KEYWORDS | Metal-binding | **RT** |  | 25 | 9.7E-1 | 1.0E0 |   were not clustered. | |  | |
