# Supplementary material for: Enabling cell-type-specific behavioral epigenetics in Drosophila: a modified high-yield INTACT method reveals the impact of social environment on the epigenetic landscape in dopaminergic neurons
Source: BMC Biol. 2019 Apr 10;17:30. doi: 10.1186/s12915-019-0646-4 (PMC6456965; doi:10.1186/s12915-019-0646-4)
Supplement: Supplementary file 10 — Gorilla and DAVID functional analysis. The zip file contains top level html files which may be opened in a browser. These will give the Gorilla functional analysis and DAVID GO analyses referred to in the main text. (ZIP 919 kb) [file 12915_2019_646_MOESM10_ESM.zip › Additional File 10/FDR20Function_files/top.html]

GOrilla


|  |  |  |  |  |  |  |  |
| --- | --- | --- | --- | --- | --- | --- | --- |
| Process |  | Function |  | Component |  | Back to GOrilla |  |
