# Supplementary material for: Enabling cell-type-specific behavioral epigenetics in Drosophila: a modified high-yield INTACT method reveals the impact of social environment on the epigenetic landscape in dopaminergic neurons
Source: BMC Biol. 2019 Apr 10;17:30. doi: 10.1186/s12915-019-0646-4 (PMC6456965; doi:10.1186/s12915-019-0646-4)
Supplement: Supplementary file 10 — Gorilla and DAVID functional analysis. The zip file contains top level html files which may be opened in a browser. These will give the Gorilla functional analysis and DAVID GO analyses referred to in the main text. (ZIP 919 kb) [file 12915_2019_646_MOESM10_ESM.zip › Additional File 10/FDR20Function_files/GOResultsPROCESS.html]

Results


*P-value color scale*

|  |  |  |  |  |
| --- | --- | --- | --- | --- |
| > 10-3 | 10-3 to 10-5 | 10-5 to 10-7 | 10-7 to 10-9 | < 10-9 |


|  |  |  |  |  |  |
| --- | --- | --- | --- | --- | --- |
| **GO term** | **Description** | **P-value** | **FDR q-value** | **Enrichment (N, B, n, b)** | **Genes** |
| GO:0061733 | peptide-lysine-N-acetyltransferase activity | 4.77E-4 | 8.54E-1 | 2.28 (3385,18,1073,13) | [-] Hide genes  nej - nejire  dom - domino  enok - enoki mushroom  Nipped-A - cg33554 gene product from transcript cg33554-re  Taf12 - tbp-associated factor 12  d4 - cg2682 gene product from transcript cg2682-rb  E(Pc) - enhancer of polycomb  Naa60 - n(alpha)-acetyltransferase 60  CG3967 - cg3967 gene product from transcript cg3967-rc  Gas41 - cg9207 gene product from transcript cg9207-ra  CG8677 - cg8677 gene product from transcript cg8677-rb  CG9293 - cg9293 gene product from transcript cg9293-rb  Taf10b - tbp-associated factor 10b |

Species used: Drosophila melanogaster

The system has recognized 5116 genes out of 5691 gene terms entered by the user.  
 5116 genes were recognized by gene symbol and 0 genes by other gene IDs .  
1236 duplicate genes were removed (keeping the highest ranking instance of each gene) leaving a total of 3880 genes.  
Only 3385 of these genes are associated with a GO term.

The GOrilla database is periodically updated using the GO database and other sources.  
The GOrilla database was last updated on Aug 4, 2018

This results page will be available on this site for one month from now (until
Sep 9, 2018
). You can bookmark this page and come back to it later.

  
**'P-value'** is the enrichment p-value computed according to the mHG or HG model. This p-value is not corrected for multiple testing of 1789 GO terms.  
  
**'FDR q-value'** is the correction of the above p-value for multiple testing using the Benjamini and Hochberg (1995) method.   
Namely, for the ith term (ranked according to p-value) the FDR q-value is (p-value \* number of GO terms) / i.   
  
**Enrichment (N, B, n, b)** is defined as follows:  
N - is the total number of genes  
B - is the total number of genes associated with a specific GO term  
n - is the number of genes in the top of the user's input list or in the target set when appropriate  
b - is the number of genes in the intersection  
Enrichment = (b/n) / (B/N)  
  
**Genes:** For each GO term you can see the list of associated genes that appear in the optimal top of the list.  
Each gene name is specified by gene symbol followed by a short description of the gene   

Back to the GOrilla main page
